# Supplementary material for: Cascade cyclization of alkene-tethered acylsilanes and allylic sulfones enabled by unproductive energy transfer photocatalysis
Source: Nat Commun. 2022 Oct 16;13:6111. doi: 10.1038/s41467-022-33730-x (PMC9573877; doi:10.1038/s41467-022-33730-x)
Supplement: Supplementary file 1 — Supplementary Information [file 41467_2022_33730_MOESM1_ESM.pdf]

Supplementary Information

Cascade Cyclization of Alkene-tethered Acylsilanes and Allylic Sulfones Enabled by Unproductive Energy Transfer Photocatalysis

Yunxiao Zhang[1,2#], Yizhi Zhang[1#], Chen Ye[3,4], Xiaotian Qi[5*], Li-Zhu Wu[3,4*] and Xiao Shen[1,2*]

[1]The Institute for Advanced Studies, Engineering Research Center of Organosilicon Compounds & Materials, Ministry of Education, Wuhan University, Wuhan, China; [2]Shenzhen Research Institute of Wuhan University, Wuhan University, Shenzhen, China. [3]Key Laboratory of Photochemical Conversion and Optoelectronic Materials, Technical Institute of Physics and Chemistry, Chinese Academy of Sciences, Beijing, China. [4]School of Future Technology, University of Chinese Academy of Sciences, Beijing, China. [5]Engineering Research Center of Organosilicon Compounds & Materials, Ministry of Education, College of Chemistry and Molecular Sciences, Wuhan University, Wuhan, China. [#]These authors contributed equally to this work.

*E-mail: xiaoshen@whu.edu.cn; qi7xiaotian@whu.edu.cn; lzwu@mail.ipc.ac.cn

Table of contents

1. **General information** S1
2. **Synthesis of substrates** S1
3. **Substrate scope** S5
4. **Down-stream transformations of products** S41
5. **Mechanism study** S42
6. **Computational Details** S62
7. **Spectroscopic Data** S128
8. **Supplementary References** S237

1. General information

Chromatography: HaiLang Silica Flash P60 size 40~45 μm (300~400 mesh), TLC: HaiLang silica gel 60 (0.25mm). Visualization of the chromatogram was performed by UV, phosphomolybdic acid and $KMnO_4$ staining. Mass spectra were recorded on Bruker UltiMate 3000 & Compact, Thermo ISQ LT, LTQ XL and VELOS pro & ORBITRIP mass spectrometers. ^{1}H, ^{13}C, ^{19}F, ^{29}Si were recorded on Bruker 400, Bruker 600 and JNM–ECZ 400 using $CDCl_3$ or DMSO-d_6 as solvent. Chemical shift values are reported in ppm with the solvent resonance as the internal standard ($CDCl_3$: δ 7.26 for ^{1}H, δ 77.16 for ^{13}C). Data are reported as follows: chemical shifts, multiplicity (s = singlet, bs = broad singlet, d = doublet, dd = doublet of doublets, t = triplet, td = triplet of doublets, m = multiplet), coupling constants (Hz), and integration. Infrared spectra were recorded on an Agilent Technologies Cary 630 FTIR and wavelengths are reported in cm^{-1}. Melting point was measured by INESA SGW X–4. All reagents were used as received and solvents were dried and degassed according to standard procedure. If no special description, all reactions were conducted under nitrogen. Eosin Y (neutral eosin Y CAS: 15086-94-9), 4,4'-di-tert-butyl-2,2'-bipyridine was purchased from laajoo, $IrCl_3{\cdot}xH_2O$ was purchased from adamas, 1,3-Dithiane was purchased from bide, 1,3-Dithiane was purchased from bide, all of the Chlorosilane and $FeCl_3$ were purchased from Heowns, $PhSO_2Na$, $K_2S_2O_8$, Chloramine T, Acetophenone were purchased from adamas, KOPiv was purchased from Ark, collidine (2,4,6-triMethypyridine), *n*-BuLi were purchased from Energy chemical.

2. Synthesis of substrates

General procedure for preparation of Alkene-tethered Acylsilanes.

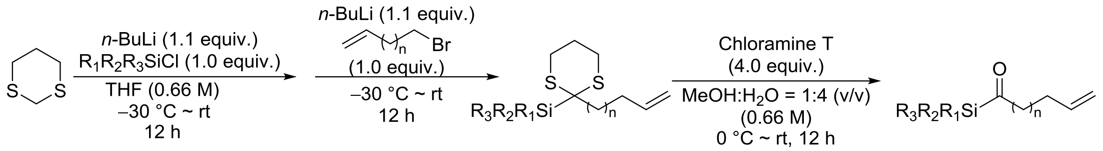

Alkene-tethered acylsilanes **1a~1f** are all known compounds and they were synthesized according to reported procedures.[1] To a solution of 1,3-dithiane (4.8 g, 40 mmol) in dry THF (60 mL) was added *n*-butyllithium (17.6 mL, 2.5 M in hexane, 44 mmol) dropwise at –30 ºC. After the mixture was stirred for 0.5 h, chlorosilane (40 mmol) was slowly added, and the reaction was warmed to ambient temperature. After being stirred for additional 12 h, the reaction mixture was cooled to –30 ºC, and then *n*-butyllithium (17.6 mL, 2.5 M in hexane, 44 mmol) was added to the reaction mixture. After the mixture was stirred for 0.5 h, bromoalkyl olefin (40 mmol) was added dropwise at –30 ºC. The reaction mixture was warmed to ambient temperature over 12 h, and then the reaction was quenched with H_2O (100 mL). The aqueous layers were extracted with EA (70 mL×3), and the combined organic layers were washed with brine, dried over anhydrous Na_2SO_4, and concentrated in vacuo. Purification by flash silica gel column chromatography using PE/EA (v/v = 100:1) as an eluent gave crude product substituted dithiolane. To a solution of substituted dithiolane in MeOH/H_2O solution (v/v = 4:1, 75 mL) was added chloramine T trihydrate (36.3 g, 160 mmol) in one portion at 0 °C, and then the reaction mixture was allowed to warm to ambient temperature. After the resulting mixture was stirred for additional 12 h. The aqueous layers were extracted with hexane (150 mL×3), and the combined organic layers were washed with brine, dried over anhydrous Na_2SO_4, and concentrated in vacuo carefully. The crude product was purified via distillation to give the alkene-tethered acylsilanes.

1-(Trimethylsilyl)hex-5-en-1-one 1a

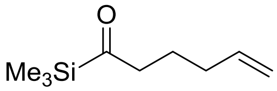

Yellow oil. (2.5 g, 3 steps total yield: 36%), NMR Spectroscopy: ^{1}H NMR (400 MHz, $CDCl_3$, 25 ℃) δ 5.79–5.68 (m, 1H), 5.02–4.91 (m, 2H), 2.59 (td, *J* = 7.3, 0.9 Hz, 2H), 2.05–1.95 (m, 2H), 1.65–1.56 (m, 2H), 0.18 (s, 9H). The characterization data is consistent to the reported data.[1]

1-(Triethylsilyl)hex-5-en-1-one 1b

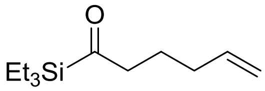

Yellow oil. (3.1 g, 3 steps total yield: 36%), NMR Spectroscopy: ^{1}H NMR (600 MHz, $CDCl_3$, 25 ℃) ^{1}H NMR (600 MHz, $CDCl_3$) δ 5.79–5.70 (m, 1H), 5.01–4.92 (m, 2H), 2.56 (t, *J* = 7.2 Hz, 2H), 2.04–1.98 (m, 2H), 1.64–1.58 (m, 2H), 0.96 (t, *J* = 7.9 Hz, 9H), 0.72 (q, *J* = 7.9 Hz, 6H). The characterization data is consistent to the reported data.[1]

1-(*tert*-Butyldimethylsilyl)hex-5-en-1-one 1c

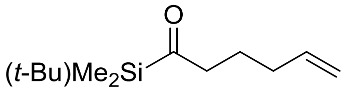

Yellow oil. (3.1 g, 3 steps total yield: 36%), NMR Spectroscopy: ^{1}H NMR (600 MHz, $CDCl_3$, 25 ℃) δ 5.76–5.66 (m, 1H), 4.99–4.89 (m, 2H), 2.57 (t, *J* = 7.2 Hz, 2H), 2.02–1.95 (m, 2H), 1.62–1.55 (m, 2H), 0.89 (s, 9H), 0.14 (s, 6H). The characterization data is consistent to the reported data.[1]

1-(Dimethyl(phenyl)silyl)hex-5-en-1-one 1d

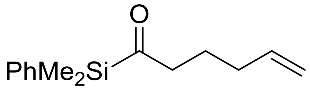

Yellow oil. (3.3 g, 3 steps total yield: 36%), NMR Spectroscopy: ^{1}H NMR (600 MHz, $CDCl_3$, 25 ℃) δ 7.56–7.53 (m, 2H), 7.42–7.37 (m, 3H), 5.71–5.63 (m, 1H), 4.94–4.88 (m, 2H), 2.57 (t, J = 7.2 Hz, 2H), 1.98–1.90 (m, 2H), 1.62–1.49 (m, 2H), 0.48 (s, 6H). The characterization data is consistent to the reported data.[1]

1-(Methyldiphenylsilyl)hex-5-en-1-one 1e

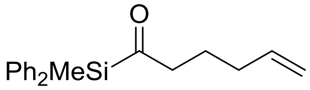

R_f = 0.45 (PE/EA = 10/1 (v/v)). Yellow oil. (4.4 g, 3 steps total yield: 37%), NMR Spectroscopy: ^{1}H NMR (600 MHz, $CDCl_3$, 25 ℃) δ 7.46–7.42 (m, 4H), 7.42–7.38 (m, 6H), 5.71–5.63 (m, 1H), 4.94–4.85 (m, 2H), 2.67 (t, J = 7.2 Hz, 2H), 1.98–1.92 (m, 2H), 1.62–1.53 (m, 2H), 0.76 (s, 3H). The characterization data is consistent to the reported data.[1]

1-(Trimethylsilyl)pent-4-en-1-one 1f

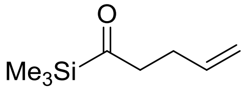

Yellow oil. (2.4 g, 3 steps total yield: 38%). NMR Spectroscopy: ^{1}H NMR (400 MHz, $CDCl_3$, 25 ℃) δ 5.84–5.69 (m, 1H), 5.02–4.91 (m, 2H), 2.74–2.64 (m, 2H), 2.32–2.20 (m, 2H), 0.19 (s, 9H). The characterization data is consistent to the reported data.[2]

General procedure for preparation of allylic sulfone.

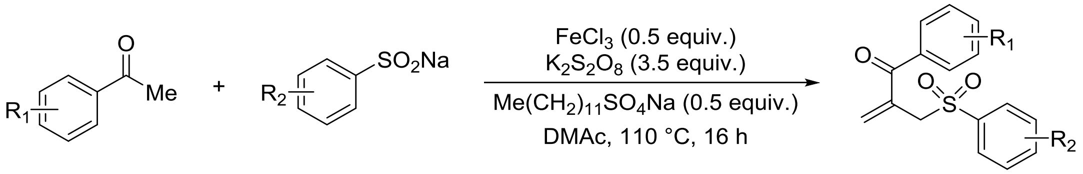

Allylic sulfones **2** were synthesized according to reported procedures. [3,4] A 250 mL oven-dried reaction vessel was charged with $K_2S_2O_8$ (9.45 g, 35 mmol), sodium dodecyl (1.74 g, 5 mmol), $FeCl_3$ (0.81 g, 5 mmol), acetophenone (1.2 g, 10 mmol), Sodium benzenesulfinate (4.5 g, 25 mmol), and DMAc (50 mL) under air. The sealed reaction vessel was stirred at 110 ºC for 12 h. After cooling to ambient temperature, the reaction was diluted with EA (100 mL) and washed with saturated sodium chloride solution. The organic layer was separated, and the aqueous layer was extracted with EA for three times. The combined organic layer was dried over magnesium sulfate and the volatiles were removed under reduced pressure. The crude product was purified with column chromatography on silica gel (200~300 mesh) with PE/EA = 10/1 (v/v) as eluent to afford the title compound as a white solid.

1-(2-Bromophenyl)-2-((phenylsulfonyl)methyl)prop-2-en-1-one 2l

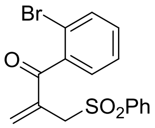

R_f = 0.35 (PE/EA = 5/1 (v/v)). White solid, mp = 124.1–125.1 ℃. (0.89 g, 81% yield). NMR Spectroscopy: ^{1}H NMR (400 MHz, $CDCl_3$, 25 ℃) δ 7.98–7.91 (m, 2H), 7.69–7.63 (m, 1H), 7.60–7.55 (m, 3H), 7.37–7.28 (m, 2H), 7.11–7.05 (m, 1H), 6.55–6.50 (m, 1H), 6.03 (s, 1H), 4.36 (d, *J* = 0.8 Hz, 2H). ^{13}C NMR (151 MHz, $CDCl_3$, 25 ℃) δ 194.4, 139.0, 138.8, 138.2, 136.0, 134.1, 133.3, 131.5, 129.3, 129.0, 128.7, 127.1, 119.4, 55.1. IR (ATR): ν 3063, 2922, 1669, 1446, 1297, 1136, 1084, 980, 902, 708 cm^{-1}.HRMS (APCI, m/z): calcd for $C_{16}H_{14}BrO_3S$ $(M+H)^+$: 364.9842; found: 364.9855.

2-((Phenylsulfonyl)methyl)-1-(2-(trifluoromethyl)phenyl)prop-2-en-1-one 2n

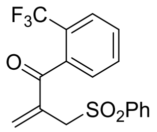

R_f = 0.36 (PE/EA = 5/1 (v/v)). White solid, mp = 123.9–124.7 ℃. (0.89 g, 83% yield). NMR Spectroscopy: ^{1}H NMR (600 MHz, $CDCl_3$, 25 ℃) δ 7.98–7.93 (m, 2H), 7.75–7.64 (m, 3H), 7.61–7.56 (m, 4H), 6.52 (d, *J* = 0.9 Hz, 1H), 5.93 (s, 1H), 4.36 (s, 2H). ^{13}C NMR (151 MHz, $CDCl_3$, 25 ℃) δ 194.2, 138.9, 138.1, 136.7, 136.6 (q, J = 2.1 Hz), 134.2, 131.5, 130.3, 129.4, 128.6, 128.5, 126.8 (q, *J* = 4.6 Hz), 123.5 (q, *J* = 273.9 Hz), 55.2. ^{19}F NMR (565 MHz, $CDCl_3$, 25 ℃) δ −58.3 (s, 3F). IR (ATR): ν 3063, 2922, 1669, 1446, 1297, 1136, 1084, 980, 902, 708 cm^{-1}. HRMS (APCI, m/z): calcd for $C_{17}H_{14}F_3O_3S$ $(M+H)^+$: 355.0610; found: 355.0619.

1-Phenyl-2-((o-tolylsulfonyl)methyl)prop-2-en-1-one 2ac

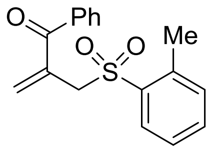

R_f = 0.37 (PE/EA = 5/1 (v/v)). mp = 62.2–63.6 ºC. (1.8 g, 60 % yield). NMR Spectroscopy: ^{1}H NMR (600 MHz, $CDCl_3$, 25 ºC) δ 7.95 (dd, *J* = 7.9, 1.4 Hz, 1H), 7.62–7.58 (m, 2H), 7.55–7.51 (m, 1H), 7.47–7.36 (m, 3H), 7.31–7.27 (m, 2H), 6.33 (s, 1H), 6.02 (s, 1H), 4.40 (d, *J* = 0.9 Hz, 2H), 2.76 (s, 3H). ^{13}C NMR (151 MHz, $CDCl_3$, 25 ºC) δ 195.0, 139.0, 137.0, 136.2, 135.6, 134.5, 134.1, 133.0, 132.8, 130.7, 129.7, 128.4, 126.5, 56.7, 20.5. IR (ATR): ν 3060, 2993, 2929, 1654, 1468, 1397, 1304, 1248, 1148, 984, 756, 708. cm^{-1}. HRMS (APCI, m/z): calcd for $C_{17}H_{17}O_3S$ $(M+H)^+$: 301.0893; found: 301.0893.

2-(([1,1'-Biphenyl]-4-ylsulfonyl)methyl)-1-phenylprop-2-en-1-one 2ai

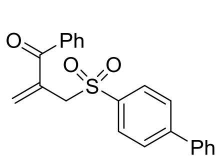

R_f = 0.38 (PE/EA = 5/1 (v/v)). White solid, mp = 87.5–88.1 ℃. (1.4 g, 40% yield). NMR Spectroscopy: ^{1}H NMR (600 MHz, $CDCl_3$, 25 ℃) δ 7.98–7.95 (m, 2H), 7.72–7.67 (m, 4H), 7.57–7.53 (m, 3H), 7.49–7.45 (m, 2H), 7.44–7.40 (m, 3H), 6.31 (d, *J* = 0.9 Hz, 1H), 6.06 (d, *J* = 0.6 Hz, 1H), 4.41 (d, *J* = 0.9 Hz, 2H). ^{13}C NMR (151 MHz, $CDCl_3$, 25 ℃)) δ 194.8, 146.9, 139.0, 137.3, 136.1, 135.6, 134.0, 132.7, 129.7, 129.1, 128.9, 128.7, 128.3, 127.8, 127.4, 57.9. IR (ATR): ν 3063, 1654, 1595, 1479, 1446, 1394, 1304, 1203, 1092, 969, 756 cm^{-1}. HRMS (APCI, m/z): calcd for $C_{22}H_{19}O_3S$ $(M+H)^+$: 363.1044; found: 363.1049.

1-Phenyl-2-(((4-(trifluoromethoxy)phenyl)sulfonyl)methyl)prop-2-en-1-one 2aj

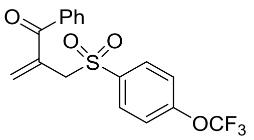

R_f = 0.40 (PE/EA = 4/1 (v/v)). White solid, mp = 84.3–85.9 ℃. (1.5 g, 41% yield). NMR Spectroscopy: ^{1}H NMR (600 MHz, $CDCl_3$, 25 ℃) δ 7.98–7.93 (m, 2H), 7.67–7.62 (m, 2H), 7.58–7.54 (m, 1H), 7.46–7.41 (m, 2H), 7.32 (d, *J* = 8.4 Hz, 2H), 6.35 (d, *J* = 1.4 Hz, 1H), 6.09 (s, 1H), 4.38 (s, 2H). ^{13}C NMR (151 MHz, $CDCl_3$, 25 ℃) δ 194.7, 153.3, 137.1, 136.1, 135.4, 134.7, 133.0, 130.9, 129.7, 128.5, 121.1, 120.3 (q, *J* = 260.0 Hz), 58.0. ^{19}F NMR (565 MHz, $CDCl_3$, 25 ℃) δ –57.7 (s, 3F). IR (ATR): ν 3052, 1654, 1587, 1490, 1446, 1297, 1256, 1203, 1148, 1084, 984, 741, 693 cm^{-1}. HRMS (APCI, m/z): calcd for $C_{17}H_{14}F_3O_4S$ $(M+H)^+$: 371.0551; found: 371.0559.

3. Substrate scope

3.1 Reaction setup

20 mL reaction vials are placed at the hole of photoreactive plant. Two parallel LED lamps (total 24 W) are placed perpendicularly to the sidewall of reaction vials (at approximately 1 cm away from the light source), so that the reactions vials can be equally exposed to the LEDs (about 6W was distributed to each hole). A clip fan at one end of the plant had been kept working during the reaction, offsetting the heat generated from the LED light and to stabilize reaction temperature for reproducible results.

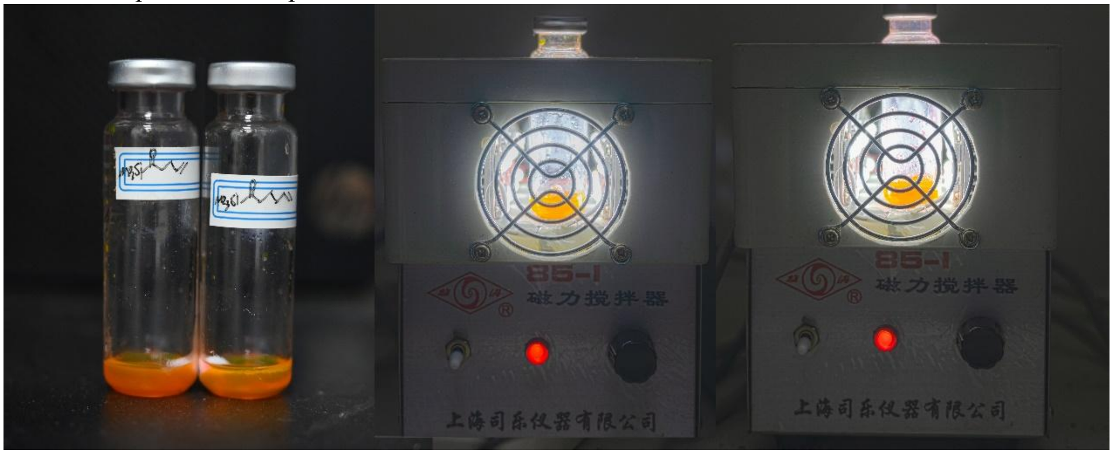

Supplementary Figure 1: The device of photo-catalysis reaction

White LEDs were used for light irradiations (see figure S3).

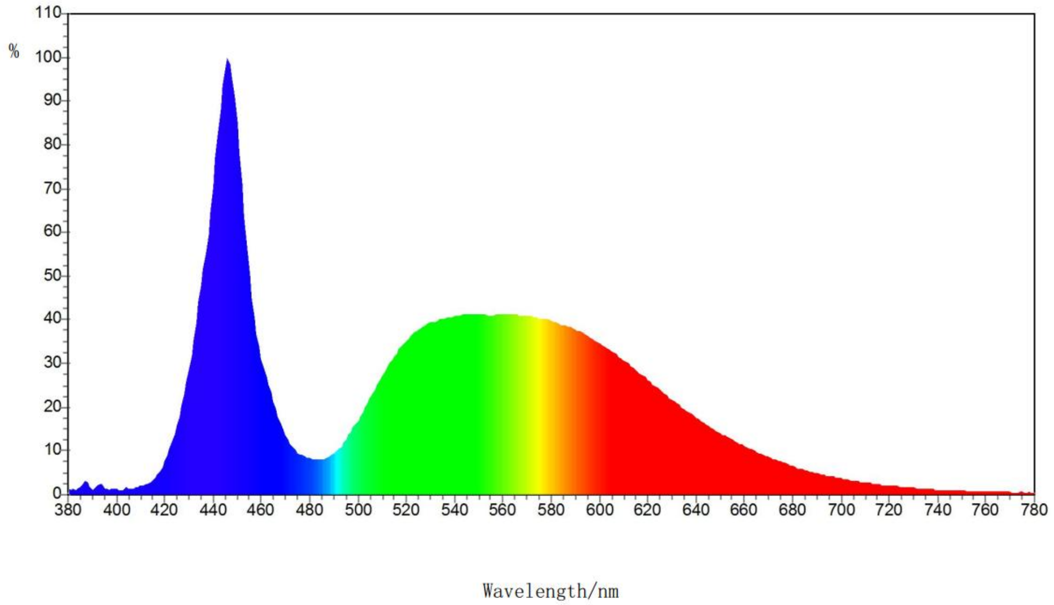

Supplementary Figure 2: Emission spectra of the white LEDs used in this work

3.2 Investigation of the reaction conditions.

Supplementary Table 1: Optimization of solvent.

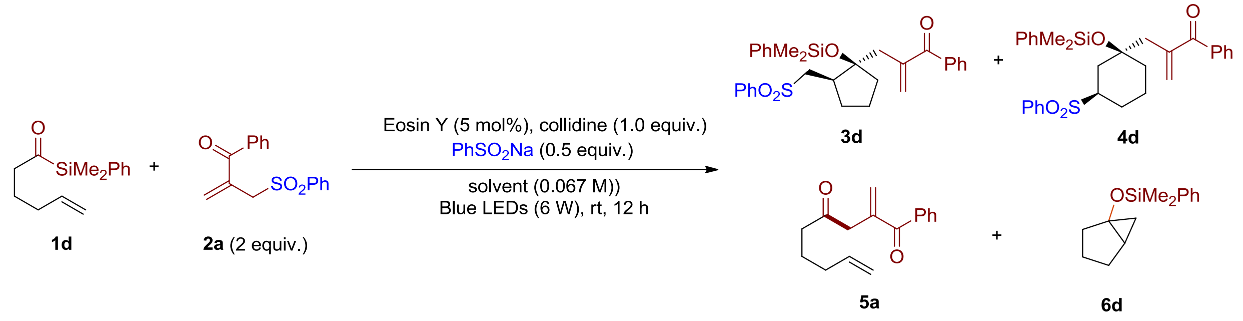

entry	solvent	Yield of **3d**/% (dr)	Yield of **4d**/% (dr)	Yield of **5a**/% (dr)	Yield of **6d**/% (dr)
1	DMF	36 (85/15)	0	0	0
2	MeCN	52 (88/12)	0	0	0
3	THF	44 (87/13)	0	0	0
4	DMSO	18 (87/13)	0	0	0
5	DCM	40 (87/13)	0	0	0
6	MeOH	58 (86/14)	0	0	0
7	H_2O	20 (80/20)	0	0	0
8	MeCN/H_2O 1/1	60 (90/10)	0	0	0
9	MeOH/H_2O 1/1	60 (90/10)	0	0	0
10	MeCN/MeOH 1/1	50 (88/12)	0	0	0

[a]N_2, Reactions were run on 0.1 mmol scale in 1.5 mL dry solvent for 12 h. The yield was determined by ^1H NMR spectroscopy with $BrCH_2CH_2Br$ as an internal standard of the **3d**.

Supplementary Table 2 Optimization of catalyst and light source.

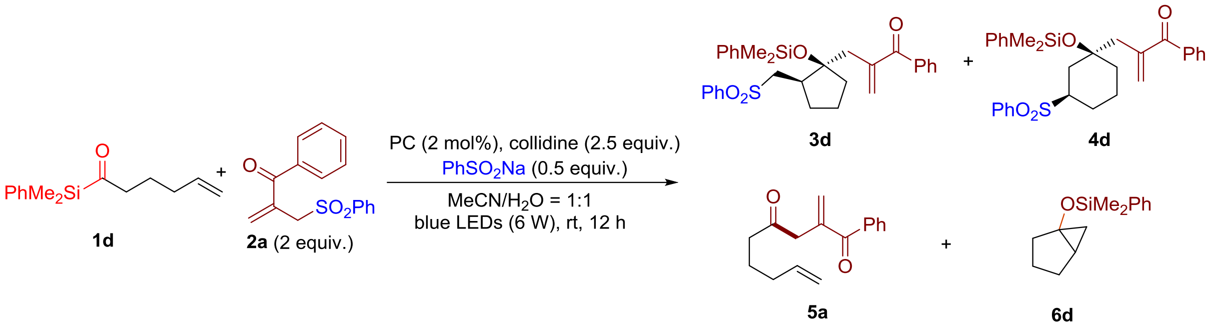

Entry	PC	Yield of **3d**/% (dr)	Yield of **4d**/% (dr)	Yield of **5a**/% (dr)	Yield of **6d**/% (dr)
1	$[Ir(dF(CF_3)ppy)_2(dtbbpy)]PF_6$	4 (87/13)	0	4	0
2	$[Ir(dF(CF_3)ppy)_2(5,5'\text{-}d(CF_3)bpy)]PF_6$	28 (87/13)	0	12	0
3	4CzIPN	36 (90/10)	0	4	0
4	Eosin Y (5 mol%)[b]	60 (90/10)	0	0	0
5	Eosin Y (5 mol%)[c]	58 (90/10)	0	0	0
6	Eosin Y (5 mol%)[d]	64 (90/10)	0	0	0

[a] N_2, Reactions were run on 0.1 mmol scale in 1.5 mL dry solvent for 12 h. The yield was determined by ^1H NMR spectroscopy with $BrCH_2CH_2Br$ as an internal standard of the **3d**. [b] $PhSO_2Na$ (1 equiv.) was used. [c] green LEDs was used. [d] white LED was used.

Supplementary Table 3 Optimization of base.

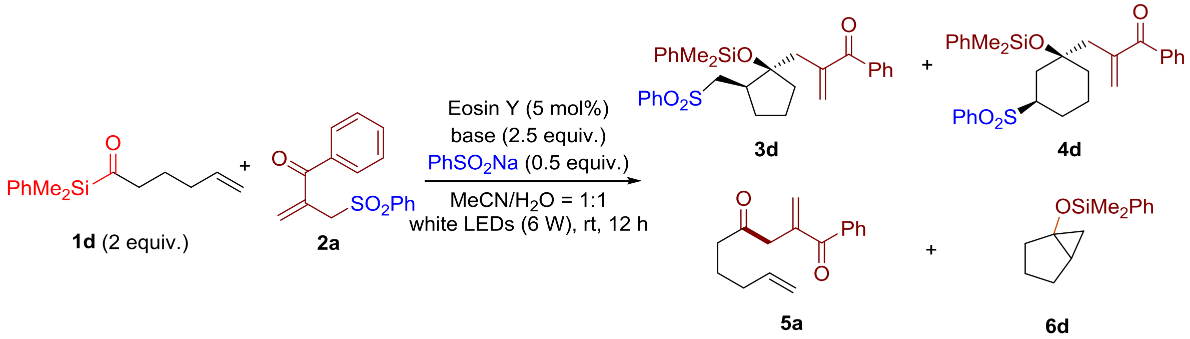

Entry	base	Yield of **3d**/% (dr)	Yield of **4d**/% (dr)	Yield of **5a**/% (dr)	Yield of **6d**/%
1	Cs_2CO_3	56 (89/11)	0	0	0
2	CsOAc	58 (89/11)	0	0	0
3	K_3PO_4	64 (90/10)	0	0	0
4	*t*BuOK	24 (91/9)	0	0	0
5	KOPiv	66 (90/10)	0	0	0
6	collidine	64 (89/11)	0	0	0
7	KOPiv (1 equiv.)	66 (90/10)	0	0	0

[a] N_2, Reactions were run on 0.1 mmol scale in 1.5 mL dry solvent for 12 h. The yield was determined by ^1H NMR spectroscopy with $BrCH_2CH_2Br$ as an internal standard of the **3d**. [b] **1d** (2 equiv.), **2a** (1 equiv.) was used.

Supplementary Table 4 Optimization of ratio of MeCN/H_2O.

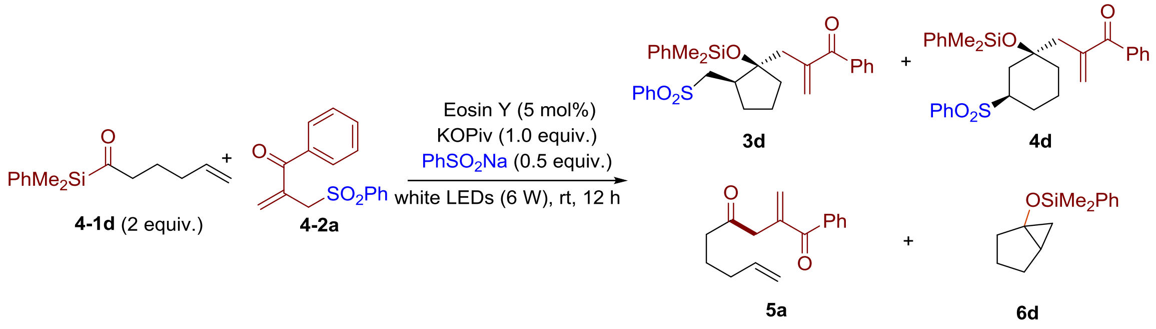

Entry	solvent	Yield of **3d**/% (dr)	Yield of **4d**/% (dr)	Yield of **5a**/% (dr)	Yield of **6d**/%
1	MeCN/H_2O = 3:1	46 (88/12)	0	0	0
2	MeCN/H_2O = 1:1	70 (90/10)	0	0	0
3	MeCN/H_2O = 1:2	70 (89/12)	0	0	0
4	MeCN/H_2O = 1:3	83 (88/12)82[b]	0	0	0
5	MeCN/H_2O = 1:5	60 (88/12)	0	0	0
6	MeCN/H_2O = 1:8	40 (87/13)	0	0	0

[a]N_2, Reactions were run on 0.1 mmol scale in 1.5 mL dry solvent for 12 h. The yield was determined by ^1H NMR spectroscopy with $BrCH_2CH_2Br$ as an internal standard of the **3d**. [b]Yield of isolated product is given within parentheses.

Supplementary Table 5 Optimization of influence of silyl group.

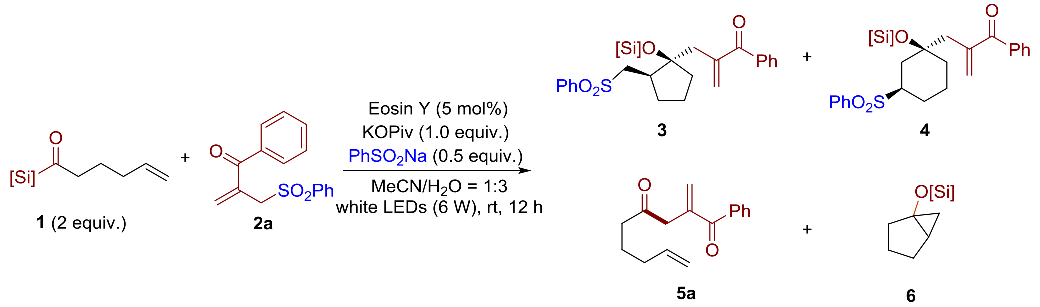

entry	[Si]	Yield of **3**/% (dr)	Yield of **4**/% (dr)	Yield of **5a**/% (dr)	Yield of **6**/% (dr)
1	TMS	86 (90/10) 82[b]	0	0	0
2	TES	66 (86/14) 63[b]	0	0	0
3	TBS	44 (86/14) 50[b]	0	0	0
4	$SiMe_2Ph$	83 (88/12) 82[b]	0	0	0
5	$SiMePh_2$	73 (87/13) 71[b]	0	0	0

[a]N_2, Reactions were run on 0.1 mmol scale in 1.5 mL dry solvent for 12 h. The yield was determined by ^1H NMR spectroscopy with $BrCH_2CH_2Br$ as an internal standard of the **3**. [b]Yield of isolated product is given within parentheses.

Supplementary Table 6 Control experiments.

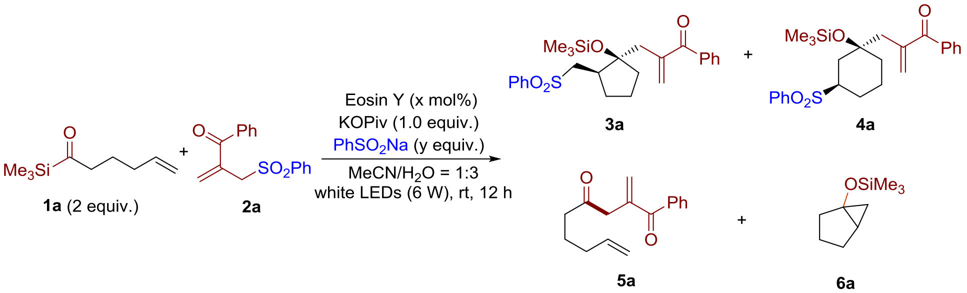

Entry	Eosin Y	$PhSO_2Na$	Yield of **3a**/% (dr)	Yield of **4a**/% (dr)	Yield of **5a**/% (dr)	Yield of **6a**/% (dr)
1	5 mol%	0.2 equiv.	82 (90/10)	0	0	0
2	5 mol%	0.1 equiv.	80 (90/10)	0	0	0
3	5 mol%	0.05 equiv.	68 (90/10)	0	0	0
4	2 mol%	0.2 equiv.	89 (90/10)	0	0	0
5	1 mol%	0.2 equiv.	89 (90/10) 82[b]	0	0	0
6	0.5 mol%	0.2 equiv.	84 (90/10)	0	0	0
7	0.2 mol%	0.2 equiv.	82 (90/10)	0	0	0
8[c]	1 mol%	0.2 equiv.	78 (90/10)	0	0	0
9[d]	1 mol%	0.2 equiv.	0	0	0	0
10[e]	0	0.2 equiv.	14 (87/13)	0	0	0
11[f]	1 mol%	0.2 equiv.	10 (87/13)	0	0	0
12[g]	1 mol%	0.2 equiv.	84 (90/10)	0	0	0

[a]N_2, Reactions were run on 0.1 mmol scale in 1.5 mL dry solvent for 12 h. The yield was determined by ^{1}H NMR spectroscopy with $BrCH_2CH_2Br$ as an internal standard of the **3a**. [b]Yield of isolated product is given within parentheses. [c]no KOPiv was used, [d]no light. [e]no Eosin Y. [f]Air atmosphere. [g]reaction concentration (0.025 M).

3.3 Scope for the *β*-substituent cyclopentanol derivatives

1-Phenyl-2-((2-((phenylsulfonyl)methyl)-1-((trimethylsilyl)oxy)cyclopentyl)methyl)prop-2-en-1-one (3a)

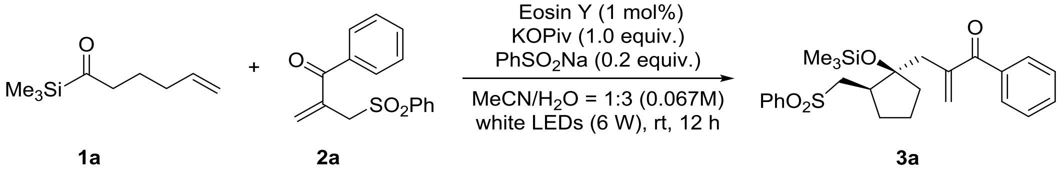

In a glovebox, to an oven-dried 10 mL tube was added **2a** (28.6 mg, 0.1 mmol), $PhSO_2Na$ (3.2 mg, 0.02 mmol, 0.2 equiv.), Eosin Y (0.65 mg, 0.001 mmol, 1 mol%), KOPiv (14.2 mg, 0.1 mmol, 1 equiv.), MeCN/H_2O =1:3 (0.067 M) and **1a** (34.0 mg, 0.2 mmol, 2 equiv.) sequentially. The tube was sealed, then irradiated with 6 W white LED lamps. The mixture was stirred under white light irradiation at ambient temperature for the 12 h. Then the light was turned off. The resulting mixture was filtered through a thin silica gel plug with EA (30 mL) as the eluent. The organic phase was concentrated under reduced pressure. The dr was determined by the analysis of the unpurified crude mixture by ^{1}H NMR. The crude product was purified with column chromatography on silica gel (300~400 mesh) with PE/EA = 5/1 (v/v) as eluent to afford the title compound as a colorless oil (37.5 mg, 82 % yield in total, a mixture of two disteraosiomers).

Charaterization of the major isomer: R_f = 0.48 (PE/EA = 5/1 (v/v)). NMR Spectroscopy: ^{1}H NMR (600 MHz, $CDCl_3$, 25 ℃) δ 7.93–7.88 (m, 2H), 7.65–7.62 (m, 2H), 7.58–7.52 (m, 2H), 7.51–7.47 (m, 2H), 7.44–7.41 (m, 2H), 5.77 (d, *J*

= 1.0 Hz, 1H), 5.65 (d, J = 0.9 Hz, 1H), 3.45 (dd, J = 14.3, 2.0 Hz, 1H), 3.02 (dd, J = 14.3, 10.6 Hz, 1H), 2.84 (dd, J = 13.2, 0.9 Hz, 1H), 2.75 (dd, J = 13.1, 0.9 Hz, 1H), 2.12–1.91 (m, 2H), 1.77–1.66 (m, 2H), 1.65–1.54 (m, 2H), 1.55–1.45 (m, 1H), 0.08 (s, 9H). ^{13}C NMR (151 MHz, $CDCl_3$, 25 ℃) δ 197.3, 144.1, 140.1, 137.2, 133.5, 132.5, 130.6, 129.8, 129.2, 128.4, 128.1, 85.7, 57.8, 41.0, 40.2, 37.0, 29.6, 21.6, 2.2. ^{29}Si NMR (119 MHz, $CDCl_3$, 25 ℃) δ 10.7. IR (ATR): ν 3063, 2959, 1654, 1446, 1304, 1252, 1148, 1069, 842, 752 cm^{-1}. HRMS (ESI, m/z): calcd for $C_{25}H_{33}O_4SSi$ $(M+H)^+$: 457.1863; found: 457.1862.

2-((1-Hydroxy-2-((phenylsulfonyl)methyl)cyclopentyl)methyl)-1-phenylprop-2-en-1-one (3a')

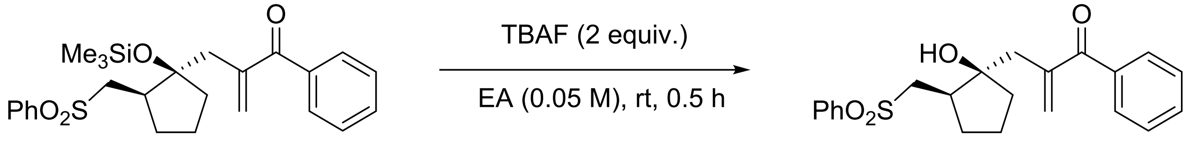

To a 4 mL tube was added **3a** (22.8 mg, 0.05 mmol), EA (0.1 M) and TBAF (0.1 mL, 0.1 mmol, 1 M in THF, 2 equiv.) sequentially. The mixture was stirred at ambient temperature for the 0.5 h. The resulting mixture was filtered through a thin silica gel plug with EA (20 mL) as the eluent. The organic phase was concentrated under reduced pressure. The crude product was purified with column chromatography on silica gel (300~400 mesh) with PE/EA = 2/1 (v/v) as eluent to afford the title compound as a colorless oil (18.8 mg, 97 % yield).

Charaterization of the major isomer: R_f = 0.22 (PE/EA = 2/1 (v/v)). NMR Spectroscopy: ^{1}H NMR (600 MHz, $CDCl_3$, 25 ℃) δ 7.94–7.91 (m, 2H), 7.76–7.73 (m, 2H), 7.65–7.62 (m, 1H), 7.60–7.54 (m, 3H), 7.47–7.43 (m, 2H), 6.03 (d, J = 1.0 Hz, 1H), 5.79 (d, J = 0.7 Hz, 1H), 3.90 (s, 1H), 3.40 (dd, J = 14.3, 2.9 Hz, 1H), 3.17 (dd, J = 14.3, 9.7 Hz, 1H), 2.81 (d, J = 13.7 Hz, 1H), 2.54 (d, J = 13.7 Hz, 1H), 2.26–2.21 (m, 1H), 2.11–2.05 (m, 1H), 1.82–1.75 (m, 1H), 1.72–1.54 (m, 4H). ^{13}C NMR (151 MHz, $CDCl_3$, 25 ℃) δ 200.5, 144.1, 140.4, 137.0, 133.7, 133.1, 131.6, 130.1, 129.4, 128.5, 128.0, 81.6, 57.9, 43.3, 43.2, 38.9, 31.2, 21.7. IR (ATR): ν 3541, 3063, 2967, 2873, 1654, 1601, 1446, 1304, 1144, 1084, 909, 738, 689 cm^{-1}. HRMS (ESI, m/z): calcd for $C_{22}H_{25}O_4S$ $(M+H)^+$: 385.1468; found: 385.1463.

1-Phenyl-2-((2-((phenylsulfonyl)methyl)-1-((triethylsilyl)oxy)cyclopentyl)methyl)prop-2-en-1-one (3b)

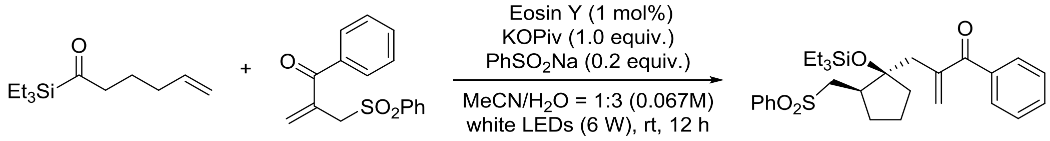

In a glovebox, to an oven-dried 10 mL tube was added **2a** (57.2 mg, 0.2 mmol), $PhSO_2Na$ (6.4 mg, 0.04 mmol, 0.2 equiv.), Eosin Y (1.3 mg, 0.002 mmol, 1 mol%), KOPiv (28.4 mg, 0.2 mmol, 1 equiv.), $MeCN/H_2O$ =1:3 (0.067 M) and **1b** (82.8 mg, 0.4 mmol, 2 equiv.) sequentially. The tube was sealed, then irradiated with 6 W white LED lamps. The mixture was stirred under white light irradiation at ambient temperature for the 12 h. Then the light was turned off. The resulting mixture was filtered through a thin silica gel plug with EA (30 mL) as the eluent. The organic phase was concentrated under reduced pressure. The crude product was purified with column chromatography on silica gel (300~400 mesh) with PE/EA = 5/1 (v/v) as eluent to afford the title compound as a colorless oil (69.4 mg, 69 % yield in total, a mixture of two disteraosiomers).

Charaterization of the major isomer: R_f = 0.45 (PE/EA = 5/1 (v/v)). NMR Spectroscopy: ^{1}H NMR (600 MHz, $CDCl_3$, 25 ℃) δ 7.92–7.89 (m, 2H), 7.61 (dd, *J* = 8.3, 1.4 Hz, 2H), 7.57–7.51 (m, 2H), 7.50–7.45 (m, 2H), 7.45–7.40 (m, 2H), 5.78 (d, *J* = 0.9 Hz, 1H), 5.67 (d, *J* = 0.7 Hz, 1H), 3.47 (dd, *J* = 14.3, 1.7 Hz, 1H), 3.09 (dd, *J* = 14.3, 10.2 Hz, 1H), 2.95 (dd, *J* = 13.2, 0.9 Hz, 1H), 2.70 (dd, *J* = 13.0, 0.8 Hz, 1H), 2.01–1.95 (m, 2H), 1.78–1.68 (m, 1H), 1.66–1.57 (m, 2H), 1.56–1.44 (m, 2H), 0.93 (t, *J* = 7.9 Hz, 9H), 0.66–0.54 (m, 6H). ^{13}C NMR (151 MHz, $CDCl_3$, 25 ℃) δ 197.3, 143.9, 140.1, 137.2, 133.5, 132.5, 131.2, 129.7, 129.2, 128.4, 128.0, 85.5, 57.7, 40.8, 39.3, 37.1, 29.6, 21.5, 7.3, 6.7. ^{29}Si NMR (119 MHz, $CDCl_3$, 25 ℃) δ 13.5. IR (ATR): ν 2981, 2929, 2862, 1610, 1513, 1356, 1248, 1170, 1080, 1002, 834 cm^{-1}. HRMS (ESI, m/z): calcd for $C_{28}H_{39}O_4SSi$ $(M+H)^+$: 499.2333; found: 499.2332.

2-((1-((Tert-butyldimethylsilyl)oxy)-2-((phenylsulfonyl)methyl)cyclopentyl)methyl)-1-phenylprop-2-en-1-one (3c)

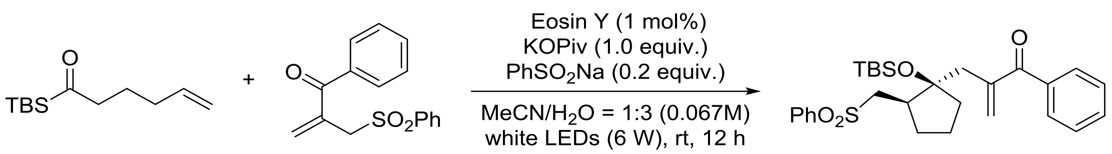

1c 2a 3c

In a glovebox, to an oven-dried 10 mL tube was added **2a** (57.2 mg, 0.2 mmol), $PhSO_2Na$ (6.4 mg, 0.04 mmol, 0.2 equiv.), Eosin Y (1.3 mg, 0.002 mmol, 1 mol%), KOPiv (28.4 mg, 0.2 mmol, 1 equiv.), MeCN/H_2O =1:3 (0.067 M) and **1c** (82.8 mg, 0.4 mmol, 2 equiv.) sequentially. The tube was sealed, then irradiated with 6 W white LED lamps. The mixture was stirred under white light irradiation at ambient temperature for the 12 h. Then the light was turned off. The resulting mixture was filtered through a thin silica gel plug with EA (30 mL) as the eluent. The organic phase was concentrated under reduced pressure. The crude product was purified with column chromatography on silica gel (300~400 mesh) with PE/EA = 5/1 (v/v) as eluent to afford the title compound as a colorless oil (69.1 mg, 69 % yield in total, a mixture of two disteraosiomers).

Charaterization of the major isomer: R_f = 0.44 (PE/EA = 5/1 (v/v)). NMR Spectroscopy: ^{1}H NMR (600 MHz, $CDCl_3$, 25 ℃) δ 7.96–7.88 (m, 2H), 7.64–7.59 (m, 2H), 7.58–7.51 (m, 2H), 7.50–7.46 (m, 2H), 7.45–7.42 (m, 2H), 5.80 (d, *J* = 0.9 Hz, 1H), 5.68 (d, *J* = 0.7 Hz, 1H), 3.47 (dd, *J* = 14.4, 1.8 Hz, 1H), 3.11 (dd, *J* = 14.3, 10.5 Hz, 1H), 3.01 (dd, *J* = 13.2, 0.9 Hz, 1H), 2.67 (dd, *J* = 13.1, 0.8 Hz, 1H), 2.01–1.93 (m, 2H), 1.77–1.69 (m, 1H), 1.66–1.61 (m, 1H), 1.60–1.55 (m, 1H), 1.53–1.44 (m, 2H), 0.84 (s, 9H), 0.15 (s, 3H), 0.13 (s, 3H). ^{13}C NMR (151 MHz, $CDCl_3$, 25 ℃) δ 197.3, 143.8, 140.1, 137.2, 133.5, 132.5, 131.5, 129.7, 129.2, 128.4, 128.1, 85.8, 57.7, 41.0, 38.8, 36.5, 29.6, 26.0, 21.3, 18.5, –1.9, –2.6. ^{29}Si NMR (119 MHz, $CDCl_3$, 25 ℃) δ 12.5. IR (ATR): ν 2952, 2929, 2858, 1654, 1446, 1308, 1259, 1146, 1066, 913, 838, 775, 738, 711 cm^{-1}. HRMS (ESI, m/z): calcd for $C_{28}H_{39}O_4SSi$ $(M+H)^+$: 499.2333; found: 499.2332.

2-((1-((Dimethyl(phenyl)silyl)oxy)-2-((phenylsulfonyl)methyl)cyclopentyl)methyl)-1-phenylprop-2-en-1-one (3d)

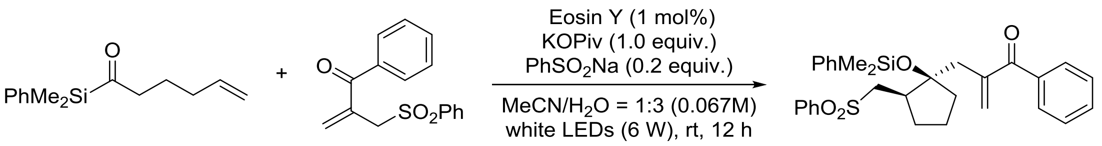

1d 2a 3d

In a glovebox, to an oven-dried 10 mL tube was added **2a** (28.6 mg, 0.1 mmol), $PhSO_2Na$ (3.2 mg, 0.02 mmol, 0.2 equiv.), Eosin Y (0.65 mg, 0.001 mmol, 1 mol%), KOPiv (14.2 mg, 0.1 mmol, 1 equiv.), MeCN/H_2O =1:3 (0.067 M) and **1d** (46.4 mg, 0.2 mmol, 2 equiv.) sequentially. The tube was sealed, then irradiated with 6 W white LED lamps.

The mixture was stirred under white light irradiation at ambient temperature for the 12 h. Then the light was turned off. The resulting mixture was filtered through a thin silica gel plug with EA (30 mL) as the eluent. The organic phase was concentrated under reduced pressure. The crude product was purified with column chromatography on silica gel (300~400 mesh) with PE/EA = 5/1 (v/v) as eluent to afford the title compound as a colorless oil (34.2 mg, 66 % yield in total, a mixture of two disteraosiomers).

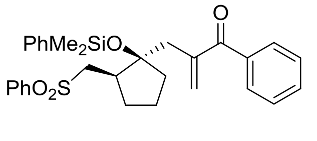

3d

Charaterization of the major isomer: R_f = 0.44 (PE/EA = 5/1 (v/v)). NMR Spectroscopy: ^{1}H NMR (600 MHz, $CDCl_3$, 25 ℃) δ 7.92–7.87 (m, 2H), 7.61 (dd, *J* = 8.3, 1.4 Hz, 2H), 7.56–7.53 (m, 2H), 7.53–7.46 (m, 4H), 7.44–7.40 (m, 2H), 7.39–7.35 (m, 1H), 7.35–7.31 (m, 2H), 5.76–5.74 (m, 1H), 5.65 (d, *J* = 0.7 Hz, 1H), 3.50 (dd, *J* = 14.3, 2.0 Hz, 1H), 3.07 (dd, *J* = 14.3, 10.6 Hz, 1H), 2.91–2.84 (m, 1H), 2.77 (d, *J* = 12.9 Hz, 1H), 2.10–1.95 (m, 2H), 1.70–1.64 (m, 1H), 1.64–1.56 (m, 2H), 1.56–1.50 (m, 1H), 1.50–1.40 (m, 1H), 0.41 (s, 3H), 0.37 (s, 3H). ^{13}C NMR (151 MHz, $CDCl_3$, 25 ℃) δ 197.2, 143.9, 140.1, 139.2, 137.2, 133.5, 133.2, 132.4, 130.9, 129.7, 129.6, 129.2, 128.4, 128.0, 128.0, 86.4, 57.7, 41.0, 39.9, 36.9, 29.6, 21.4, 0.9, 0.8. ^{29}Si NMR (119 MHz, $CDCl_3$, 25 ℃) δ 0.4. IR (ATR): ν 3062, 2959, 2858, 1654, 1446, 1304, 1252, 1192, 1144, 1058, 827, 786 cm^{-1}. HRMS (ESI, m/z): calcd for $C_{30}H_{35}O_4SSi$ $(M+H)^+$: 519.2120; found: 519.2135.

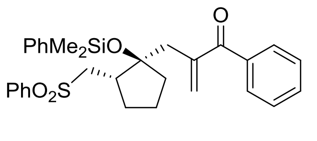

3d'

Charaterization of the minor isomer: R_f = 0.40 (PE/EA = 5/1 (v/v)). NMR Spectroscopy: ^{1}H NMR (600 MHz, $CDCl_3$, 25 ºC) δ 7.86–7.82 (m, 2H), 7.67–7.61 (m, 3H), 7.52 (dt, *J* = 13.5, 7.6 Hz, 3H), 7.49–7.45 (m, 2H), 7.38 (dt, *J* = 10.0, 7.6 Hz, 3H), 7.34 (dd, *J* = 7.8, 6.5 Hz, 2H), 5.87 (s, 1H), 5.68 (d, *J* = 1.3 Hz, 1H), 3.43 (dd, *J* = 13.6, 1.9 Hz, 1H), 3.00 (dd, *J* = 13.5, 11.9 Hz, 1H), 2.57 (d, *J* = 13.1 Hz, 1H), 2.43–2.38 (m, 1H), 2.36 (d, *J* = 13.2 Hz, 1H), 2.13–2.05 (m, 1H), 1.71–1.64 (m, 1H), 1.61–1.57 (m, 3H), 1.47–1.37 (m, 1H), 0.21 (s, 3H), 0.20 (s, 3H). ^{13}C NMR (151 MHz, $CDCl_3$, 25 ºC) δ 197.6, 144.2, 140.0, 139.0, 137.1, 133.7, 133.5, 132.4, 130.0, 129.6, 129.5, 129.4, 128.3, 128.1, 127.9, 85.5, 57.4, 44.6, 36.4, 35.6, 27.2, 19.8, 0.8, 0.6. IR (ATR): ν 3060, 2925, 2929, 1654, 1595, 1446, 1308, 1256, 1148, 834, 745, 700 cm^{-1}. HRMS (ESI, m/z): calcd for $C_{30}H_{35}O_4SSi$ $(M+H)^+$: 519.2120; found: 519.2021.

2-((1-((Methyldiphenylsilyl)oxy)-2-((phenylsulfonyl)methyl)cyclopentyl)methyl)-1-phenylprop-2-en-1-one (3e)

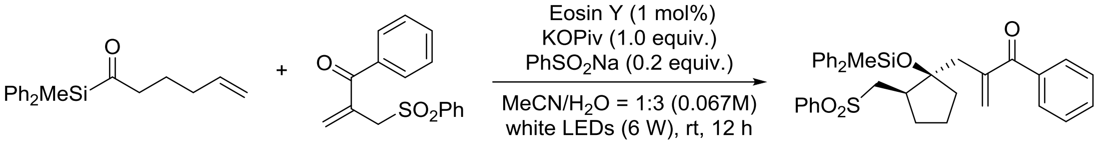

1e 2a 3e

In a glovebox, to an oven-dried 10 mL tube was added **2a** (28.6 mg, 0.1 mmol), $PhSO_2Na$ (3.2 mg, 0.02 mmol, 0.2 equiv.), Eosin Y (0.65 mg, 0.001 mmol, 1 mol%), KOPiv (14.2 mg, 0.1 mmol, 1 equiv.), $MeCN/H_2O$ =1:3 (0.067 M) and **1e** (58.8 mg, 0.2 mmol, 2 equiv.) sequentially. The tube was sealed, then irradiated with 6 W white LED lamps. The mixture was stirred under white light irradiation at ambient temperature for the 12 h. Then the light was turned off. The resulting mixture was filtered through a thin silica gel plug with EA (30 mL) as the eluent. The organic phase was concentrated under reduced pressure. The crude product was purified with column chromatography on silica gel (300~400 mesh) with PE/EA = 5/1 (v/v) as eluent to afford the title compound as a colorless oil (30.1 mg, 52 % yield in total, a mixture of two disteraosiomers).

Charaterization of the major isomer: R_f = 0.44 (PE/EA = 5/1 (v/v)). NMR Spectroscopy: ^{1}H NMR (600 MHz, $CDCl_3$, 25 ℃) δ 7.93–7.87 (m, 2H), 7.59–7.56 (m, 2H), 7.56–7.50 (m, 6H), 7.50–7.46 (m, 2H), 7.43–7.36 (m, 4H), 7.36–

7.30 (m, 4H), 5.73 (d, *J* = 0.9 Hz, 1H), 5.65 (d, *J* = 0.7 Hz, 1H), 3.55 (dd, *J* = 14.2, 1.7 Hz, 1H), 3.20 (dd, *J* = 14.2, 10.3 Hz, 1H), 2.94 (dd, *J* = 13.3, 0.8 Hz, 1H), 2.77 (dd, *J* = 13.2, 0.8 Hz, 1H), 2.04 (ddt, *J* = 14.4, 11.5, 5.0 Hz, 2H), 1.73–1.64 (m, 2H), 1.58–1.53 (m, 1H), 1.53–1.47 (m, 1H), 1.46–1.39 (m, 1H), 0.75 (s, 3H). ^{13}C NMR (151 MHz, $CDCl_3$, 25 ℃) δ 197.2, 143.7, 140.1, 137.6, 137.4, 137.2, 134.3, 133.5, 132.4, 131.5, 129.9, 129.9, 129.7, 129.2, 128.4, 128.1, 128.0, 128.0, 87.3, 57.7, 41.2, 39.5, 36.9, 29.7, 21.3, –0.3. ^{29}Si NMR (119 MHz, $CDCl_3$, 25 ℃) δ –10.1. IR (ATR): ν 3067, 2963, 2926, 2873, 1654, 1446, 1304, 1259, 1058, 915,738 cm^{-1}. HRMS (ESI, m/z): calcd for $C_{35}H_{37}O_4SSi$ $(M+H)^+$: 581.2176; found: 581.2180.

2-((2-((Phenylsulfonyl)methyl)-1-((trimethylsilyl)oxy)cyclopentyl)methyl)-1-(p-tolyl)prop-2-en-1-one (3f)

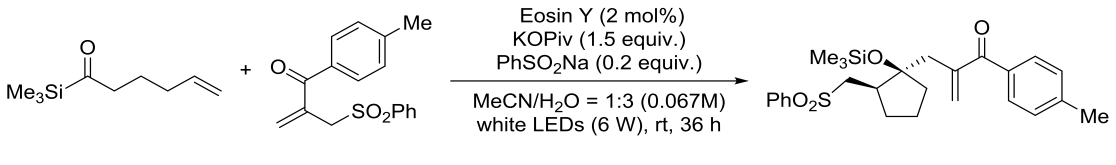

1a **2f** **3f**

In a glovebox, to an oven-dried 10 mL tube was added **2f** (60 mg, 0.2 mmol), $PhSO_2Na$ (6.4 mg, 0.04 mmol, 0.2 equiv.), Eosin Y (2.6 mg, 0.004 mmol, 2 mol%), KOPiv (14.2 mg, 0.3 mmol, 1.5 equiv.), $MeCN/H_2O$ =1:3 (0.067 M) and **1a** (68 mg, 0.4 mmol, 2 equiv.) sequentially. The tube was sealed, then irradiated with 6 W white LED lamps. The mixture was stirred under white light irradiation at ambient temperature for the 12 h. Then the light was turned off. The resulting mixture was filtered through a thin silica gel plug with EA (30 mL) as the eluent. The organic phase was concentrated under reduced pressure. The crude product was purified with column chromatography on silica gel (300~400 mesh) with PE/EA = 5/1 (v/v) as eluent to afford the title compound as a colorless oil (51.9 mg, 55 % yield in total, a mixture of two disteraosiomers).

Charaterization of the major isomer: R_f = 0.43 (PE/EA = 5/1 (v/v)). NMR Spectroscopy: ^{1}H NMR (600 MHz, $CDCl_3$, 25 ℃) δ 7.94–7.90 (m, 2H), 7.60–7.55 (m, 3H), 7.54–7.49 (m, 2H), 7.25–7.21 (m, 2H), 5.71 (d, *J* = 1.0 Hz, 1H), 5.61 (d, *J* = 0.9 Hz, 1H), 3.44 (dd, *J* = 14.4, 2.1 Hz, 1H), 3.02 (dd, *J* = 14.4, 10.8 Hz, 1H), 2.86 (dd, *J* = 13.2, 0.9 Hz, 1H), 2.72 (dd, *J* = 13.2, 0.9 Hz, 1H), 2.42 (s, 3H), 2.08–2.03 (m, 1H), 2.02–1.96 (m, 1H), 1.76–1.65 (m, 2H), 1.60–1.52 (m, 2H), 1.52–1.45 (m, 1H), 0.08 (s, 9H). ^{13}C NMR (151 MHz, $CDCl_3$, 25 ℃) δ 197.0, 144.2, 143.3, 140.2, 134.4, 133.5, 130.0, 129.7, 129.3, 129.1, 128.1, 85.7, 57.8, 40.9, 40.4, 37.0, 29.6, 21.8, 21.6, 2.3. ^{29}Si NMR (119 MHz, $CDCl_3$, 25 ℃) δ 10.6. IR (ATR): ν 3063, 2959, 1654, 1606, 1446, 1408, 1304, 1252, 1148, 1066, 916, 842, 698 cm^{-1}. HRMS (ESI, m/z): calcd for $C_{26}H_{35}O_4SSi$ $(M+H)^+$: 471.2020; found: 471.2025.

2-((2-((Phenylsulfonyl)methyl)-1-((trimethylsilyl)oxy)cyclopentyl)methyl)-1-(m-tolyl)prop-2-en-1-one (3g)

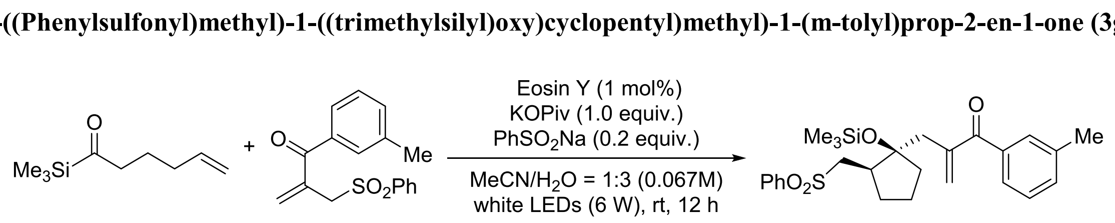

1a **2g** **3g**

In a glovebox, to an oven-dried 10 mL tube was added **2g** (90 mg, 0.3 mmol), $PhSO_2Na$ (9.84 mg, 0.06 mmol, 0.2 equiv.), Eosin Y (1.9 mg, 0.003 mmol, 1 mol%), KOPiv (42.6 mg, 0.3 mmol, 1 equiv.), $MeCN/H_2O$ =1:3 (0.067 M) and **1a** (102 mg, 0.6 mmol, 2 equiv.) sequentially. The tube was sealed, then irradiated with 6 W white LED lamps. The mixture was stirred under white light irradiation at ambient temperature for the 12 h. Then the light was turned off. The resulting mixture was filtered through a thin silica gel plug with EA (30 mL) as the eluent. The organic phase

was concentrated under reduced pressure. The crude product was purified with column chromatography on silica gel (300~400 mesh) with PE/EA = 5/1 (v/v) as eluent to afford the title compound as a colorless oil (98.7 mg, 70 % yield in total, a mixture of two disteraosiomers).

Charaterization of the major isomer: R_f = 0.44 (PE/EA = 5/1 (v/v)). NMR Spectroscopy: ^{1}H NMR (400 MHz, $CDCl_3$, 25 ℃) δ 7.94–7.89 (m, 2H), 7.58–7.53 (m, 1H), 7.52–7.46 (m, 3H), 7.42–7.34 (m, 2H), 7.32–7.26 (m, 1H), 5.76 (q, *J* = 0.9 Hz, 1H), 5.65 (d, *J* = 0.9 Hz, 1H), 3.46 (dd, *J* = 14.3, 1.9 Hz, 1H), 3.03 (dd, *J* = 14.4, 10.5 Hz, 1H), 2.85 (dd, *J* = 13.3, 0.9 Hz, 1H), 2.72 (dd, *J* = 13.3, 0.9 Hz, 1H), 2.41 (d, *J* = 0.7 Hz, 3H), 2.11–1.92 (m, 2H), 1.77–1.64 (m, 2H), 1.62–1.44 (m, 3H), 0.09 (s, 9H).^{13}C NMR (151 MHz, $CDCl_3$, 25 ℃) δ 197.5, 144.2, 140.2, 138.3, 137.3, 133.5, 133.2, 130.6, 130.2, 129.2, 128.2, 128.1, 127.1, 85.8, 57.8, 41.0, 40.1, 37.0, 29.7, 21.6, 21.5, 2.3. ^{29}Si NMR (119 MHz, $CDCl_3$, 25 ℃) δ 10.6. IR (ATR): ν 3063, 2955, 1654, 1446, 1304, 1252, 1144, 1066, 838, 749, 689 cm^{-1}. HRMS (ESI, m/z): calcd for $C_{26}H_{35}O_4SSi$ $(M+H)^+$: 471.2120; found: 471.2119.

2-((2-((Phenylsulfonyl)methyl)-1-((trimethylsilyl)oxy)cyclopentyl)methyl)-1-(o-tolyl)prop-2-en-1-one (3h)

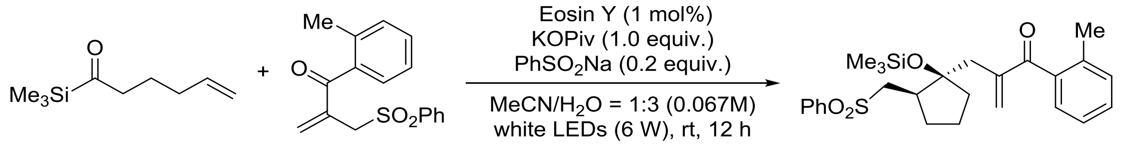

1a **2h** **3h**

In a glovebox, to an oven-dried 10 mL tube was added **2h** (90 mg, 0.3 mmol), $PhSO_2Na$ (9.84 mg, 0.06 mmol, 0.2 equiv.), Eosin Y (1.9 mg, 0.003 mmol, 1 mol%), KOPiv (42.6 mg, 0.3 mmol, 1 equiv.), MeCN/H_2O =1:3 (0.067 M) and **1a** (102 mg, 0.6 mmol, 2 equiv.) sequentially. The tube was sealed, then irradiated with 6 W white LED lamps. The mixture was stirred under white light irradiation at ambient temperature for the 12 h. Then the light was turned off. The resulting mixture was filtered through a thin silica gel plug with EA (30 mL) as the eluent. The organic phase was concentrated under reduced pressure. The crude product was purified with column chromatography on silica gel (300~400 mesh) with PE/EA = 5/1 (v/v) as eluent to afford the title compound as a colorless oil (105.0 mg, 75 % yield in total, a mixture of two disteraosiomers).

Charaterization of the major isomer: R_f = 0.43 (PE/EA = 5/1 (v/v)). NMR Spectroscopy: ^{1}H NMR (600 MHz, $CDCl_3$, 25 ℃) δ 7.93–7.88 (m, 2H), 7.55–7.49 (m, 1H), 7.48–7.44 (m, 2H), 7.34–7.31 (m, 1H), 7.22–7.21 (m, 1H), 7.18–7.15 (m, 1H), 7.08 (dd, *J* = 7.6, 1.4 Hz, 1H), 5.87 (d, *J* = 0.9 Hz, 1H), 5.65 (d, *J* = 0.8 Hz, 1H), 3.54 (dd, *J* = 14.3, 1.9 Hz, 1H), 3.05 (dd, *J* = 14.3, 10.6 Hz, 1H), 2.79 (dd, *J* = 13.1, 0.8 Hz, 1H), 2.72 (dd, *J* = 13.1, 0.8 Hz, 1H), 2.28 (s, 3H), 2.06–1.92 (m, 2H), 1.79–1.69 (m, 2H), 1.66–1.59 (m, 2H), 1.57–1.50 (m, 1H), 0.12 (s, 9H). ^{13}C NMR (151 MHz, $CDCl_3$, 25 ℃) δ 199.4, 145.3, 140.1, 138.2, 136.8, 134.0, 133.4, 131.0, 130.1, 129.2, 128.4, 128.1, 125.1, 86.0, 57.8, 41.0, 37.7, 36.6, 29.5, 21.4, 19.9, 2.3. ^{29}Si NMR (119 MHz, $CDCl_3$, 25 ℃) δ 10.4. IR (ATR): ν 3063, 3019, 2955, 1654, 1446, 1304, 1144, 834, 730 cm^{-1}. HRMS (ESI, m/z): calcd for $C_{26}H_{35}O_4SSi$ $(M+H)^+$: 471.2120; found: 471.2122.

1-(4-Fluorophenyl)-2-((2-((phenylsulfonyl)methyl)-1-((trimethylsilyl)oxy)cyclopentyl)methyl)prop-2-en-1-one (3i)

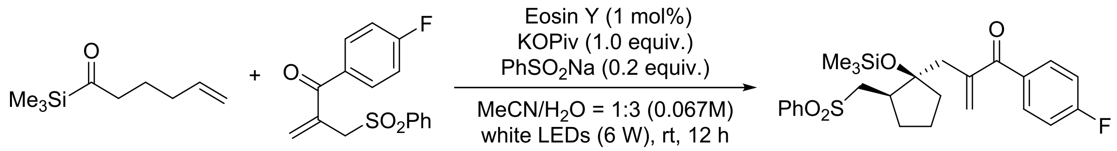

1a **2i** **3i**

In a glovebox, to an oven-dried 10 mL tube was added **2i** (91.2 mg, 0.3 mmol), $PhSO_2Na$ (9.8 mg, 0.06 mmol, 0.2 equiv.), Eosin Y (1.9 mg, 0.003 mmol, 1 mol%), KOPiv (42.6 mg, 0.3 mmol, 1 equiv.), $MeCN/H_2O$ =1:3 (0.067 M) and **1a** (102 mg, 0.6 mmol, 2 equiv.) sequentially. The tube was sealed, then irradiated with 6 W white LED lamps. The mixture was stirred under white light irradiation at ambient temperature for the 12 h. Then the light was turned off. The resulting mixture was filtered through a thin silica gel plug with EA (30 mL) as the eluent. The organic phase was concentrated under reduced pressure. The crude product was purified with column chromatography on silica gel (300~400 mesh) with PE/EA = 5/1 (v/v) as eluent to afford the title compound as a colorless oil (101.2 mg, 72 % yield in total, a mixture of two disteraosiomers).

Charaterization of the major isomer: R_f = 0.44 (PE/EA = 5/1 (v/v)). NMR Spectroscopy: ^1H NMR (600 MHz, $CDCl_3$, 25 ℃) δ 7.95–7.86 (m, 2H), 7.74–7.69 (m, 2H), 7.63–7.57 (m, 1H), 7.54–7.50 (m, 2H), 7.15–7.08 (m, 2H), 5.78 (d, *J* = 0.9 Hz, 1H), 5.63 (d, *J* = 0.8 Hz, 1H), 3.43 (dd, *J* = 14.3, 2.1 Hz, 1H), 2.99 (dd, *J* = 14.3, 10.9 Hz, 1H), 2.83–2.76 (m, 2H), 2.10–2.05 (m, 1H), 2.03–1.94 (m, 1H), 1.77–1.66 (m, 2H), 1.64–1.54 (m, 2H), 1.54–1.45 (m, 1H), 0.07 (s, 9H). ^{13}C NMR (151 MHz, $CDCl_3$, 25 ℃) δ 195.8, 165.5 (d, *J* = 254.0 Hz), 144.1, 140.3, 133.6, 133.4 (d, *J* = 3.1 Hz), 132.4 (d, *J* = 8.9 Hz), 129.9, 129.3, 128.1, 115.6 (d, *J* = 21.8 Hz), 85.7, 57.8, 40.9, 40.6, 37.1, 29.6, 21.6, 2.2. ^{29}Si NMR (119 MHz, $CDCl_3$, 25 ℃) δ 10.83. ^{19}F NMR (565 MHz, $CDCl_3$, 25 ℃) δ –106.2—106.7 (m, 1F). IR (ATR): ν 3067, 2939, 1654, 1599, 1505, 1446, 1408, 1304, 1252, 1151, 1066, 842 cm^{-1}. HRMS (ESI, m/z): calcd for $C_{25}H_{32}FO_4SSi$ $(M+H)^+$: 475.1769; found: 475.1771.

1-(2-Fluorophenyl)-2-((2-((phenylsulfonyl)methyl)-1-((trimethylsilyl)oxy)cyclopentyl)methyl) prop-2-en-1-one (3j) 9-147

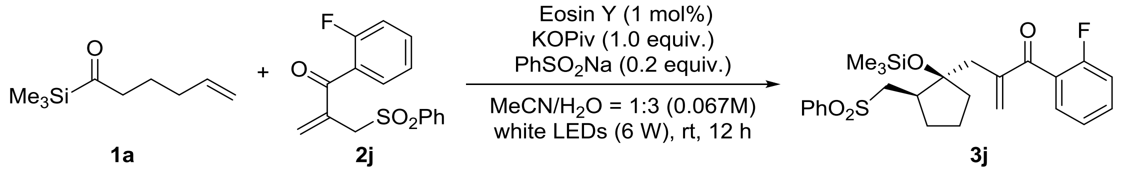

1a **2j** **3j**

In a glovebox, to an oven-dried 10 mL tube was added **2j** (91.2 mg, 0.3 mmol), $PhSO_2Na$ (9.84 mg, 0.06 mmol, 0.2 equiv.), Eosin Y (1.9 mg, 0.003 mmol, 1 mol%), KOPiv (42.6 mg, 0.3 mmol, 1 equiv.), $MeCN/H_2O$ =1:3 (0.067 M) and **1a** (102 mg, 0.6 mmol, 2 equiv.) sequentially. The tube was sealed, then irradiated with 6 W white LED lamps. The mixture was stirred under white light irradiation at ambient temperature for the 12 h. Then the light was turned off. The resulting mixture was filtered through a thin silica gel plug with EA (30 mL) as the eluent. The organic phase was concentrated under reduced pressure. The crude product was purified with column chromatography on silica gel (300~400 mesh) with PE/EA = 5/1 (v/v) as eluent to afford the title compound as a colorless oil (109.1 mg, 77 % yield in total, a mixture of two disteraosiomers).

Charaterization of the major isomer: R_f = 0.44 (PE/EA = 5/1 (v/v)). NMR Spectroscopy: ^1H NMR (600 MHz, $CDCl_3$, 25 ℃) δ 7.92–7.88 (m, 2H), 7.49–7.41 (m, 4H), 7.38–7.36 (m, 1H), 7.23–7.17 (m, 1H), 7.07–7.04 (m, 1H), 5.85 (s, 1H), 5.72 (d, *J* = 2.1 Hz, 1H), 3.50 (dd, *J* = 14.5, 2.1 Hz, 1H), 3.05 (dd, *J* = 14.4, 10.8 Hz, 1H), 2.75 (s, 2H), 2.04–1.95 (m, 1H), 1.94–1.87 (m, 1H), 1.78–1.57 (m, 4H), 1.57–1.48 (m, 1H), 0.11 (s, 9H). ^{13}C NMR (151 MHz, $CDCl_3$, 25 ℃) δ 194.4, 159.7 (d, *J* = 251.4 Hz), 145.0, 139.9, 133.3, 132.9 (d, *J* = 2.3 Hz), 132.9, 130.6 (d, *J* = 2.9 Hz), 129.1, 128.1, 126.8 (d, *J* = 14.7 Hz), 124.4 (d, *J* = 3.6 Hz), 116.2 (d, *J* = 22.0 Hz), 85.9, 57.7, 40.8, 38.1, 36.5, 29.6, 21.4, 2.3. ^{19}F NMR (565 MHz, $CDCl_3$, 25 ℃) δ –111.36—111.4 (m, 1F). ^{29}Si NMR (119 MHz, $CDCl_3$, 25 ℃) δ 10.3. IR (ATR): ν 3302, 3064, 2949, 1659, 1610, 1484, 1446, 1305, 1252, 1144, 1062, 987, 913, 835, 730 cm^{-1}. HRMS (ESI, m/z): calcd for $C_{25}H_{32}FO_4SSi$ $(M+H)^+$: 475.1769; found: 475.1770.

1-(2-Chlorophenyl)-2-((2-((phenylsulfonyl)methyl)-1-((trimethylsilyl)oxy)cyclopentyl)methyl)prop-2-en-1-one (3k)

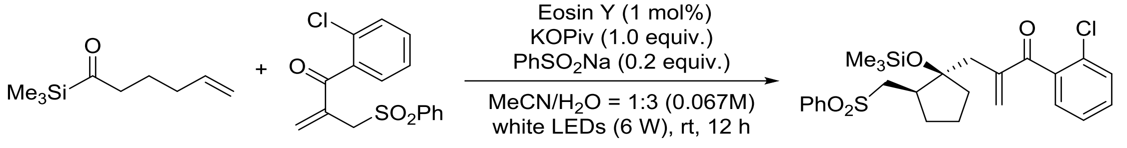

1a **2k** **3k**

In a glovebox, to an oven-dried 10 mL tube was added **2k** (96 mg, 0.3 mmol), $PhSO_2Na$ (9.84 mg, 0.06 mmol, 0.2 equiv.), Eosin Y (1.9 mg, 0.003 mmol, 1 mol%), KOPiv (42.6 mg, 0.3 mmol, 1 equiv.), MeCN/H_2O =1:3 (0.067 M) and **1a** (102 mg, 0.6 mmol, 2 equiv.) sequentially. The tube was sealed, then irradiated with 6 W white LED lamps. The mixture was stirred under white light irradiation at ambient temperature for the 12 h. Then the light was turned off. The resulting mixture was filtered through a thin silica gel plug with EA (30 mL) as the eluent. The organic phase was concentrated under reduced pressure. The crude product was purified with column chromatography on silica gel (300~400 mesh) with PE/EA = 5/1 (v/v) as eluent to afford the title compound as a colorless oil (101.4 mg, 69 % yield in total, a mixture of two disteraosiomers).

Charaterization of the major isomer: R_f = 0.44 (PE/EA = 5/1 (v/v)). NMR Spectroscopy: ^{1}H NMR (600 MHz, $CDCl_3$, 25 ℃) δ 7.94–7.91 (m, 2H), 7.57–7.52 (m, 1H), 7.51–7.46 (m, 2H), 7.41–7.35 (m, 2H), 7.32–7.29 (m, 1H), 7.23–7.21 (m, 1H), 5.99 (s, 1H), 5.72 (s, 1H), 3.55 (dd, J = 14.3, 1.8 Hz, 1H), 3.05 (dd, J = 14.3, 10.5 Hz, 1H), 2.85–2.76 (m, 1H), 2.74–2.68 (m, 1H), 2.09–1.91 (m, 2H), 1.79–1.65 (m, 3H), 1.62–1.49 (m, 2H), 0.13 (s, 9H). ^{13}C NMR (151 MHz, $CDCl_3$, 25 ℃) δ 196.4, 144.2, 140.2, 138.4, 135.5, 133.5, 131.1, 131.0, 130.1, 129.3, 129.2, 128.2, 126.7, 86.1, 57.9, 40.9, 37.2, 36.6, 29.8, 29.7, 21.4, 2.4. ^{29}Si NMR (119 MHz, $CDCl_3$, 25 ℃) δ 10.4. IR (ATR): ν 3060, 2955, 1666, 1617, 1591, 1446, 1304, 1252, 1146, 1062, 1025, 987, 842, 732 cm^{-1}. HRMS (ESI, m/z): calcd for $C_{25}H_{32}ClO_4SSi$ $(M+H)^+$: 491.1474; found: 491.1471.

1-(2-Bromophenyl)-2-((2-((phenylsulfonyl)methyl)-1-((trimethylsilyl)oxy)cyclopentyl)methyl)prop-2-en-1-one (3l)

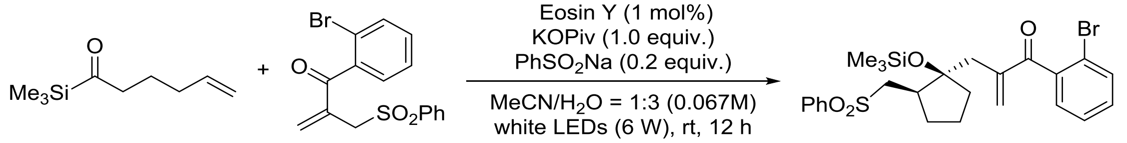

1a **2l** **3l**

In a glovebox, to an oven-dried 10 mL tube was added **2l** (108.9 mg, 0.3 mmol), $PhSO_2Na$ (9.84 mg, 0.06 mmol, 0.2 equiv.), Eosin Y (1.9 mg, 0.003 mmol, 1 mol%), KOPiv (42.6 mg, 0.3 mmol, 1 equiv.), MeCN/H_2O =1:3 (0.067 M) and **1a** (102 mg, 0.6 mmol, 2 equiv.) sequentially. The tube was sealed, then irradiated with 6 W white LED lamps. The mixture was stirred under white light irradiation at ambient temperature for the 12 h. Then the light was turned off. The resulting mixture was filtered through a thin silica gel plug with EA (30 mL) as the eluent. The organic phase was concentrated under reduced pressure. The crude product was purified with column chromatography on silica gel (300~400 mesh) with PE/EA = 5/1 (v/v) as eluent to afford the title compound as a colorless oil (111.9 mg, 70 % yield in total, a mixture of two disteraosiomers).

Charaterization of the major isomer: R_f = 0.44 (PE/EA = 5/1 (v/v)). NMR Spectroscopy: ^{1}H NMR (600 MHz, $CDCl_3$, 25 ℃) δ 7.95–7.91 (m, 2H), 7.60–7.53 (m, 2H), 7.51–7.47 (m, 2H), 7.36–7.33 (m, 1H), 7.30–7.28 (m, 1H), 7.18 (dd, J = 7.5, 1.7 Hz, 1H), 6.01 (s, 1H), 5.72 (s, 1H), 3.56 (dd, J = 14.3, 2.0 Hz, 1H), 3.04 (dd, J = 14.3, 10.5 Hz, 1H), 2.79

(dd, J = 13.3, 0.8 Hz, 1H), 2.75–2.68 (m, 1H), 2.07–1.96 (m, 2H), 1.79–1.66 (m, 3H), 1.66–1.48 (m, 2H), 0.13 (s, 9H). ^{13}C NMR (151 MHz, $CDCl_3$, 25 ℃) δ 197.0, 143.8, 140. 5, 140. 2, 135.9, 133.5, 133.2, 131.0, 129.3, 129.1, 128.1, 127.2, 119.5, 86.1, 57.8, 40.9, 37.1, 36.6, 29.7, 21.4, 2.4. ^{29}Si NMR (119 MHz, $CDCl_3$, 25 ℃) δ 10.4. IR (ATR): ν 3064, 2959, 1666, 1588, 1446, 1305, 1252, 1144, 1062, 1025, 984, 913, 835, 730 cm^{-1}. HRMS (ESI, m/z): calcd for $C_{25}H_{32}BrO_4SSi$ $(M+H)^+$: 535.0969; found: 535.0969.

1-(4-Iodophenyl)-2-((2-((phenylsulfonyl)methyl)-1-((trimethylsilyl)oxy)cyclopentyl)methyl)prop-2-en-1-one (3m)

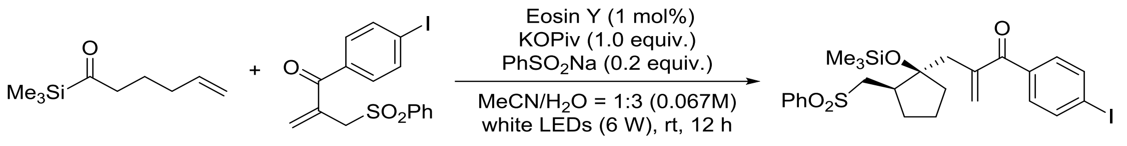

In a glovebox, to an oven-dried 10 mL tube was added **2m** (123.3 mg, 0.3 mmol), $PhSO_2Na$ (9.84 mg, 0.06 mmol, 0.2 equiv.), Eosin Y (1.9 mg, 0.003 mmol, 1 mol%), KOPiv (42.6 mg, 0.3 mmol, 1 equiv.), $MeCN/H_2O$ =1:3 (0.067 M) and **1a** (102 mg, 0.6 mmol, 2 equiv.) sequentially. The tube was sealed, then irradiated with 6 W white LED lamps. The mixture was stirred under white light irradiation at ambient temperature for the 12 h. Then the light was turned off. The resulting mixture was filtered through a thin silica gel plug with EA (30 mL) as the eluent. The organic phase was concentrated under reduced pressure. The crude product was purified with column chromatography on silica gel (300~400 mesh) with PE/EA = 5/1 (v/v) as eluent to afford the title compound as a colorless oil (138.7 mg, 81 % yield in total, a mixture of two disteraosiomers).

Charaterization of the major isomer: R_f = 0.44 (PE/EA = 5/1 (v/v)). NMR Spectroscopy: ^{1}H NMR (600 MHz, $CDCl_3$, 25 ℃) δ 7.92–7.88 (m, 2H), 7.81–7.77 (m, 2H), 7.60–7.55 (m, 1H), 7.53–7.48 (m, 2H), 7.39–7.34 (m, 2H), 5.80–5.78 (m, 1H), 5.63 (d, J = 0.7 Hz, 1H), 3.41 (dd, J = 14.3, 1.9 Hz, 1H), 2.99 (dd, J = 14.3, 10.6 Hz, 1H), 2.77 (dd, J = 2.1, 0.9 Hz, 2H), 2.06–1.94 (m, 2H), 1.76–1.65 (m, 2H), 1.62–1.45 (m, 3H), 0.07 (s, 9H). ^{13}C NMR (151 MHz, $CDCl_3$, 25 ℃) δ 196.4, 143.9, 140.1, 137.7, 136.5, 133.5, 131.2, 130.5, 129.3, 128.0, 100.1, 85.6, 57.7, 40.9, 40.3, 37.0, 29.6, 21.5, 2.2. ^{29}Si NMR (119 MHz, $CDCl_3$, 25 ℃) δ 10.82. IR (ATR): ν 3063, 2955, 1654, 1580, 1446, 1304, 1148, 1062, 987, 842, 703 cm^{-1}. HRMS (ESI, m/z): calcd for $C_{25}H_{32}IO_4SSi$ $(M+H)^+$: 583.0830; found: 583.0838.

2-((2-((Phenylsulfonyl)methyl)-1-((trimethylsilyl)oxy)cyclopentyl)methyl)-1-(2-(trifluoromethyl)phenyl)prop-2-en-1-one (3n)

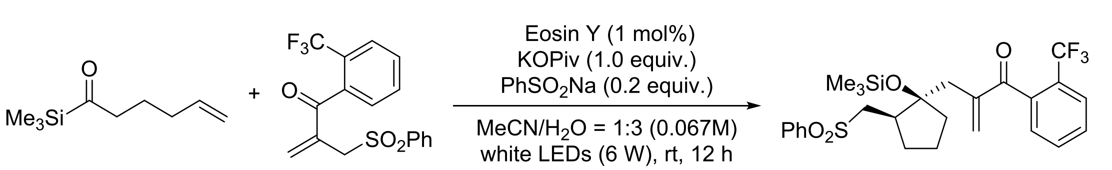

In a glovebox, to an oven-dried 10 mL tube was added **2n** (70.8 mg, 0.2 mmol), $PhSO_2Na$ (6.4 mg, 0.04 mmol, 0.2 equiv.), Eosin Y (1.3 mg, 0.002 mmol, 1 mol%), KOPiv (28.4 mg, 0.2 mmol, 1 equiv.), $MeCN/H_2O$ =1:3 (0.067 M) and **1a** (68 mg, 0.4 mmol, 2 equiv.) sequentially. The tube was sealed, then irradiated with 6 W white LED lamps. The mixture was stirred under white light irradiation at ambient temperature for the 12 h. Then the light was turned off. The resulting mixture was filtered through a thin silica gel plug with EA (30 mL) as the eluent. The organic phase was concentrated under reduced pressure. The crude product was purified with column chromatography on silica gel (300~400 mesh) with PE/EA = 5/1 (v/v) as eluent to afford the title compound as a colorless oil (68.3 mg, 65 % yield

in total, a mixture of two disteraosiomers).

Charaterization of the major isomer: R_f = 0.44 (PE/EA = 5/1 (v/v)). NMR Spectroscopy: ^{1}H NMR (600 MHz, $CDCl_3$, 25 ℃) δ 7.97–7.87 (m, 2H), 7.74–7.68 (m, 1H), 7.59–7.56 (m, 4H), 7.52–7.50 (m, 2H), 7.30–7.26 (m, 1H), 6.07 (s, 1H), 5.65 (s, 1H), 3.55 (dd, J = 14.3, 1.6 Hz, 1H), 3.02 (dd, J = 14.3, 10.4 Hz, 1H), 2.85–2.66 (m, 2H), 2.15–1.96 (m, 2H), 1.80–1.71 (m, 2H), 1.70–1.62 (m, 1H), 1.62–1.53 (m, 2H), 0.13 (s, 9H). ^{13}C NMR (151 MHz, $CDCl_3$, 25 ℃) δ 196.8, 144.7, 140.3, 138.1, 136.1, 133.5, 131.4, 129.8, 129.3, 128.4, 128.2 (q, J = 31.7 Hz), 128.1, 126.7 (q, J = 4.5 Hz), 123.7 (q, J = 273.6 Hz), 86.1, 57.9, 40.9, 37.1, 36.6, 29.8, 21.4, 2.4. ^{19}F NMR (565 MHz, $CDCl_3$, 25 ℃) δ –58.1 (s, 3F). ^{29}Si NMR (119 MHz, $CDCl_3$, 25 ℃) δ 10.5. IR (ATR): ν 3068, 2959, 1670, 1580, 1446, 1409, 1312, 1252, 1133, 1059, 984, 913, 838, 730 cm^{-1}. HRMS (ESI, m/z): calcd for $C_{26}H_{32}F_3O_4SSi$ $(M+H)^+$: 525.1737; found: 525.1740.

1-(4-Methoxyphenyl)-2-((2-((phenylsulfonyl)methyl)-1-((trimethylsilyl)oxy)cyclopentyl)methyl)prop-2-en-1-one (3o)

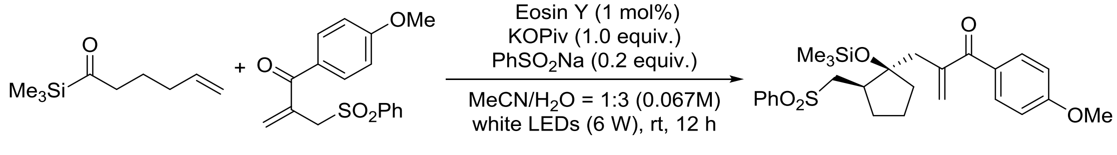

1a **2o** **3o**

In a glovebox, to an oven-dried 10 mL tube was added **2o** (63.2 mg, 0.2 mmol), Eosin Y (1.3 mg, 0.002 mmol, 1 mol%), KOPiv (28.4 mg, 0.2 mmol, 1 equiv.), $MeCN/H_2O$ =1:3 (0.067 M) and **1a** (68 mg, 0.4 mmol, 2 equiv.) sequentially. The tube was sealed, then irradiated with 6 W white LED lamps. The mixture was stirred under white light irradiation at ambient temperature for the 12 h. Then the light was turned off. The resulting mixture was filtered through a thin silica gel plug with EA (30 mL) as the eluent. The organic phase was concentrated under reduced pressure. The crude product was purified with column chromatography on silica gel (300~400 mesh) with PE/EA = 5/1 (v/v) as eluent to afford the title compound as a colorless oil (70.4 mg, 72 % yield in total, a mixture of two disteraosiomers).

Charaterization of the major isomer: R_f = 0.40 (PE/EA = 5/1 (v/v)). NMR Spectroscopy: ^{1}H NMR (600 MHz, $CDCl_3$, 25 ℃) δ 7.96–7.89 (m, 2H), 7.72–7.67 (m, 2H), 7.62–7.57 (m, 1H), 7.55–7.49 (m, 2H), 6.99–6.86 (m, 2H), 5.67 (d, J = 1.0 Hz, 1H), 5.58 (d, J = 0.9 Hz, 1H), 3.88 (s, 3H), 3.43 (dd, J = 14.3, 2.1 Hz, 1H), 3.01 (dd, J = 14.3, 10.9 Hz, 1H), 2.85 (dd, J = 13.2, 0.9 Hz, 1H), 2.73 (dd, J = 13.2, 0.9 Hz, 1H), 2.13–2.05 (m, 1H), 2.03–1.93 (m, 1H), 1.74–1.64 (m, 2H), 1.60–1.52 (m, 1H), 1.51–1.44 (m, 1H), 0.07 (s, 9H). ^{13}C NMR (151 MHz, $CDCl_3$, 25 ℃) δ 196.0, 163.3, 144.3, 140.3, 133.5, 132.2, 129.6, 129.3, 128.6, 128.1, 113.7, 85.7, 57.8, 55.6, 40.9, 37.1, 29.6, 21.6, 2.2. ^{29}Si NMR (119 MHz, $CDCl_3$, 25 ℃) δ 10.6. IR (ATR): ν 3064, 2959, 1647, 1599, 1510, 1446, 1305, 1252, 1144, 1066, 1029, 987, 838, 790, 730 cm^{-1}. HRMS (ESI, m/z): calcd for $C_{26}H_{35}O_5SSi$ $(M+H)^+$: 487.1969; found: 487.1971.

1-Cyclopropyl-2-((2-((phenylsulfonyl)methyl)-1-((trimethylsilyl)oxy)cyclopentyl)methyl)prop-2-en-1-one (3p)

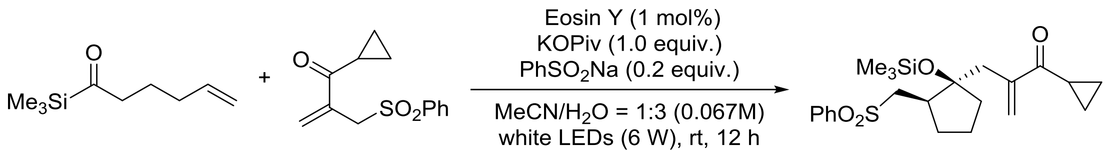

1a **2p** **3p**

In a glovebox, to an oven-dried 10 mL tube was added **2p** (75 mg, 0.3 mmol), $PhSO_2Na$ (9.84 mg, 0.06 mmol, 0.2 equiv.), Eosin Y (1.9 mg, 0.003 mmol, 1 mol%), KOPiv (42.6 mg, 0.3 mmol, 1 equiv.), $MeCN/H_2O$ =1:3 (0.067 M) and **1a** (102 mg, 0.6 mmol, 2 equiv.) sequentially. The tube was sealed, then irradiated with 6 W white LED lamps.

The mixture was stirred under white light irradiation at ambient temperature for the 12 h. Then the light was turned off. The resulting mixture was filtered through a thin silica gel plug with EA (30 mL) as the eluent. The organic phase was concentrated under reduced pressure. The crude product was purified with column chromatography on silica gel (300~400 mesh) with PE/EA = 5/1 (v/v) as eluent to afford the title compound as a colorless oil (112.0 mg, 88 % yield in total, a mixture of two disteraosiomers).

Charaterization of the major isomer: R_f = 0.45 (PE/EA = 5/1 (v/v)). NMR Spectroscopy: ^{1}H NMR (600 MHz, $CDCl_3$, 25 ℃) δ 8.00–7.90 (m, 2H), 7.69–7.62 (m, 1H), 7.61–7.54 (m, 2H), 6.20 (s, 1H), 5.70 (s, 1H), 3.43 (dd, *J* = 14.3, 1.7 Hz, 1H), 2.97 (dd, *J* = 14.3, 10.2 Hz, 1H), 2.69 (dd, *J* = 13.1, 0.9 Hz, 1H), 2.59–2.49 (m, 1H), 2.39–2.29 (m, 1H), 2.01–1.89 (m, 2H), 1.73–1.64 (m, 1H), 1.63–1.57 (m, 1H), 1.56–1.41 (m, 3H), 1.18–1.11 (m, 1H), 1.07–1.01 (m, 1H), 0.97–0.86 (m, 2H), 0.09 (s, 9H). ^{13}C NMR (151 MHz, $CDCl_3$, 25 ℃) δ 201.8, 145.9, 140.6, 133.5, 129.3, 128.1, 128.0, 86.0, 57.8, 40.8, 38.3, 36.4, 29.7, 21.5, 16.6, 11.6, 11.6, 2.3. ^{29}Si NMR (119 MHz, $CDCl_3$, 25 ℃) δ 10.0. IR (ATR): ν 3086, 3004, 2959, 1662, 1621, 1446, 1394, 1304, 1252, 1148, 1066, 1025, 842 cm^{-1}. HRMS (ESI, m/z): calcd for $C_{22}H_{33}O_4SSi$ $(M+H)^+$: 421.1863; found: 421.1872.

1-(Furan-2-yl)-2-((2-((phenylsulfonyl)methyl)-1-((trimethylsilyl)oxy)cyclopentyl)methyl)prop-2-en-1-one (3q)

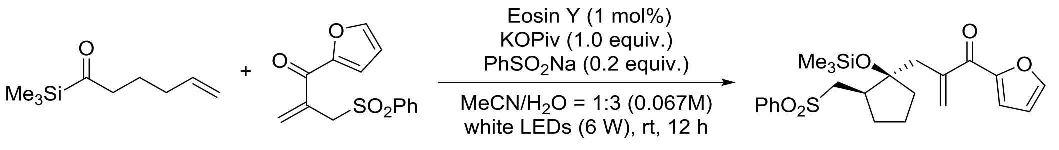

1a **2q** **3q**

In a glovebox, to an oven-dried 10 mL tube was added **2q** (82.8mg, 0.3 mmol), $PhSO_2Na$ (9.84 mg, 0.06 mmol, 0.2 equiv.), Eosin Y (1.9 mg, 0.003 mmol, 1 mol%), KOPiv (42.6 mg, 0.3 mmol, 1 equiv.), $MeCN/H_2O$ =1:3 (0.067 M) and **1a** (102 mg, 0.6 mmol, 2 equiv.) sequentially. The tube was sealed, then irradiated with 6 W white LED lamps. The mixture was stirred under white light irradiation at ambient temperature for the 12 h. Then the light was turned off. The resulting mixture was filtered through a thin silica gel plug with EA (30 mL) as the eluent. The organic phase was concentrated under reduced pressure. The crude product was purified with column chromatography on silica gel (300~400 mesh) with PE/EA = 5/1 (v/v) as eluent to afford the title compound as a colorless oil (109.1 mg, 82 % yield in total, a mixture of two disteraosiomers).

Charaterization of the major isomer: R_f = 0.44 (PE/EA = 5/1 (v/v)). NMR Spectroscopy: ^{1}H NMR (600 MHz, $CDCl_3$, 25 ℃) δ 7.96–7.89 (m, 2H), 7.67–7.63 (m, 1H), 7.61 (dd, *J* = 1.7, 0.8 Hz, 1H), 7.59–7.55 (m, 2H), 7.09 (dd, *J* = 3.5, 0.8 Hz, 1H), 6.53 (dd, *J* = 3.5, 1.7 Hz, 1H), 5.92 (d, *J* = 0.8 Hz, 1H), 5.65 (q, *J* = 1.0 Hz, 1H), 3.42 (dd, *J* = 14.3, 2.0 Hz, 1H), 2.93 (dd, *J* = 14.3, 11.0 Hz, 1H), 2.79 (dd, *J* = 13.3, 1.0 Hz, 1H), 2.70 (dd, *J* = 13.2, 0.9 Hz, 1H), 2.12–2.07 (m, 1H), 1.99–1.94 (m, 1H), 1.72–1.65 (m, 2H), 1.62–1.56 (m, 1H), 1.55–1.40 (m, 2H), 0.04 (s, 9H). ^{13}C NMR (151 MHz, $CDCl_3$, 25 ℃) δ 183.7, 151.8, 147.2, 143.9, 140.4, 133.6, 129.3, 128.1, 127.6, 120.1, 112.2, 85.4, 58.0, 41.6, 41.2, 37.5, 30.2, 21.9, 2.1. ^{29}Si NMR (119 MHz, $CDCl_3$, 25 ℃) δ 10.7. IR (ATR): ν 3131, 3067, 2955, 1647, 1561, 1464, 1390, 1304, 1252, 1144, 1066, 838, 749 cm^{-1}. HRMS (ESI, m/z): calcd for $C_{23}H_{31}O_5SSi$ $(M+H)^+$: 447.1656; found: 447.1660.

2-((2-((Phenylsulfonyl)methyl)-1-((trimethylsilyl)oxy)cyclopentyl)methyl)-1-(thiophen-2-yl)prop-2-en-1-one (3r)

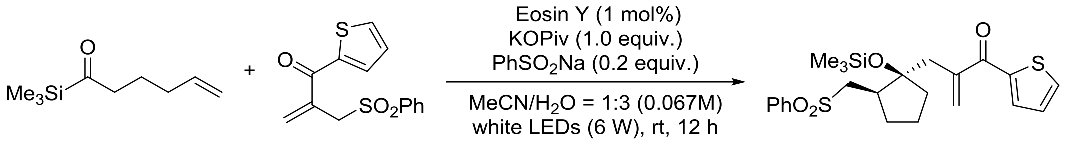

1a **2r** **3r**

In a glovebox, to an oven-dried 10 mL tube was added **2r** (87.6 mg, 0.3 mmol), $PhSO_2Na$ (9.84 mg, 0.06 mmol, 0.2 equiv.), Eosin Y (1.9 mg, 0.003 mmol, 1 mol%), KOPiv (42.6 mg, 0.3 mmol, 1 equiv.), $MeCN/H_2O$ =1:3 (0.067 M) and **1a** (102 mg, 0.6 mmol, 2 equiv.) sequentially. The tube was sealed, then irradiated with 6 W white LED lamps. The mixture was stirred under white light irradiation at ambient temperature for the 12 h. Then the light was turned off. The resulting mixture was filtered through a thin silica gel plug with EA (30 mL) as the eluent. The organic phase was concentrated under reduced pressure. The crude product was purified with column chromatography on silica gel (300~400 mesh) with PE/EA = 5/1 (v/v) as eluent to afford the title compound as a colorless oil (117.7 mg, 85 % yield in total, a mixture of two disteraosiomers).

Charaterization of the major isomer: R_f = 0.40 (PE/EA = 5/1 (v/v)). NMR Spectroscopy: ^{1}H NMR (600 MHz, $CDCl_3$, 25 ℃) δ 7.99–7.91 (m, 2H), 7.67–7.61 (m, 2H), 7.59–7.52 (m, 3H), 7.11 (dd, *J* = 5.0, 3.8 Hz, 1H), 5.83 (d, *J* = 0.8 Hz, 1H), 5.64 (d, *J* = 1.0 Hz, 1H), 3.39 (dd, *J* = 14.3, 2.1 Hz, 1H), 2.96 (dd, *J* = 14.3, 11.0 Hz, 1H), 2.83–2.64 (m, 2H), 2.14–2.09 (m, 1H), 2.01–1.90 (m, 1H), 1.74–1.64 (m, 2H), 1.60–1.51 (m, 2H), 1.50–1.40 (m, 1H), 0.05 (s, 9H). ^{13}C NMR (151 MHz, $CDCl_3$, 25 ℃) δ 188.9, 144.5, 143.2, 140.4, 134.3, 134.0, 133.6, 129.3, 128.1, 127.6, 85.5, 57.9, 41.5, 41.0, 37.3, 29.9, 21.7, 2.1. ^{29}Si NMR (119 MHz, $CDCl_3$, 25 ℃) δ 10.7. IR (ATR): ν 3093, 2955, 2877, 1636, 1513, 1412, 1304, 1148, 1066, 842, 749 cm^{-1}. HRMS (ESI, m/z): calcd for $C_{23}H_{31}O_4S_2Si$ $(M+H)^+$: 463.1428; found: 463.1423.

Ethyl 2-((2-((phenylsulfonyl)methyl)-1-((trimethylsilyl)oxy)cyclopentyl)methyl)acrylate (3s)

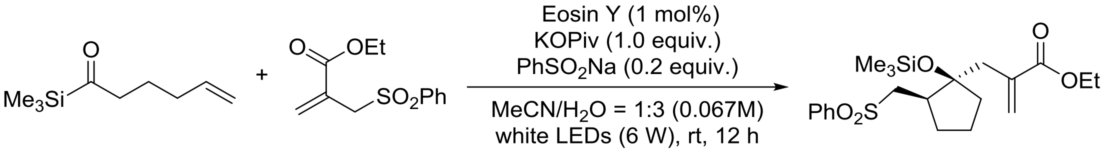

1a **2s** **3s**

In a glovebox, to an oven-dried 10 mL tube was added **2s** (76.2 mg, 0.3 mmol), $PhSO_2Na$ (9.84 mg, 0.06 mmol, 0.2 equiv.), Eosin Y (1.9 mg, 0.003 mmol, 1 mol%), KOPiv (42.6 mg, 0.3 mmol, 1 equiv.), $MeCN/H_2O$ =1:3 (0.067 M) and **1a** (102 mg, 0.6 mmol, 2 equiv.) sequentially. The tube was sealed, then irradiated with 6 W white LED lamps. The mixture was stirred under white light irradiation at ambient temperature for the 12 h. Then the light was turned off. The resulting mixture was filtered through a thin silica gel plug with EA (30 mL) as the eluent. The organic phase was concentrated under reduced pressure. The crude product was purified with column chromatography on silica gel (300~400 mesh) with PE/EA = 5/1 (v/v) as eluent to afford the title compound as a colorless oil (91.6 mg, 72 % yield in total, a mixture of two disteraosiomers).

Charaterization of the major isomer: R_f = 0.50 (PE/EA = 5/1 (v/v)). NMR Spectroscopy: ^{1}H NMR (600 MHz, $CDCl_3$, 25 ℃) δ 7.98–7.91 (m, 2H), 7.68–7.63 (m, 1H), 7.61–7.53 (m, 2H), 6.15 (d, *J* = 1.4 Hz, 1H), 5.40 (d, *J* = 1.2 Hz, 1H), 4.27–4.16 (m, 2H), 3.44 (dd, *J* = 14.4, 1.9 Hz, 1H), 2.98 (dd, *J* = 14.4, 10.6 Hz, 1H), 2.68 (dd, *J* = 13.4, 0.8 Hz, 1H), 2.52 (dd, *J* = 13.3, 0.9 Hz, 1H), 2.03–1.95 (m, 1H), 1.96–1.88 (m, 1H), 1.74–1.63 (m, 2H), 1.56–1.46 (m, 3H), 1.30

(t, J = 7.1 Hz, 3H), 0.09 (s, 9H). ^{13}C NMR (151 MHz, $CDCl_3$, 25 ℃) δ 167.6, 140.5, 136.8, 133.6, 129.4, 129.0, 128.1, 86.1, 61.2, 58.0, 40.8, 39.3, 36.4, 29.9, 21.6, 14.3, 2.3. ^{29}Si NMR (119 MHz, $CDCl_3$, 25 ℃) δ 10.1. IR (ATR): ν 3064, 2959, 1715, 1629, 1588, 1446, 1305, 1252, 1148, 1066, 1025, 947, 838, 749 cm^{-1}. HRMS (ESI, m/z): calcd for $C_{21}H_{33}O_5SSi$ $(M+H)^+$: 425.1813; found: 425.1814.

2-Cyanoethyl 2-((2-((phenylsulfonyl)methyl)-1-((trimethylsilyl)oxy)cyclopentyl)methyl)acrylate (3t)

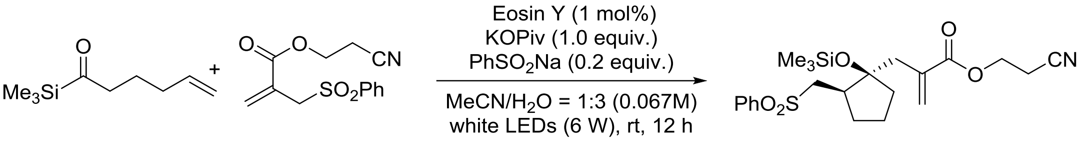

1a **2t** **3t**

In a glovebox, to an oven-dried 10 mL tube was added **2t** (27.9 mg, 0.1 mmol), $PhSO_2Na$ (3.2 mg, 0.02 mmol, 0.2 equiv.), Eosin Y (0.65 mg, 0.001 mmol, 1 mol%), KOPiv (14.2 mg, 0.1 mmol, 1 equiv.), $MeCN/H_2O$ =1:3 (0.067 M) and **1a** (34 mg, 0.2 mmol, 2 equiv.) sequentially. The tube was sealed, then irradiated with 6 W white LED lamps. The mixture was stirred under white light irradiation at ambient temperature for the 12 h. Then the light was turned off. The resulting mixture was filtered through a thin silica gel plug with EA (30 mL) as the eluent. The organic phase was concentrated under reduced pressure. The crude product was purified with column chromatography on silica gel (300~400 mesh) with PE/EA = 5/1 (v/v) as eluent to afford the title compound as a colorless oil (43.1 mg, 95 % yield in total, a mixture of two disteraosiomers).

Charaterization of the major isomer: R_f = 0.63 (PE/EA = 4/1 (v/v)). NMR Spectroscopy: ^{1}H NMR (600 MHz, $CDCl_3$, 25 ℃) δ 7.96–7.91 (m, 2H), 7.69–7.63 (m, 1H), 7.60–7.54 (m, 2H), 6.27 (d, J = 1.1 Hz, 1H), 5.57 (d, J = 1.1 Hz, 1H), 4.45–4.41 (m, 1H), 4.38–4.34 (m, 1H), 3.44 (dd, J = 14.2, 1.6 Hz, 1H), 2.94 (dd, J = 14.3, 10.4 Hz, 1H), 2.85–2.72 (m, 2H), 2.62 (t, J = 1.1 Hz, 2H), 2.00–1.90 (m, 2H), 1.76–1.65 (m, 2H), 1.65–1.57 (m, 1H), 1.56–1.42 (m, 2H), 0.09 (s, 9H). ^{13}C NMR (151 MHz, $CDCl_3$, 25 ℃) δ 166.9, 140.5, 135.8, 133.6, 130.4, 129.4, 128.0, 117.0, 86.0, 59.5, 57.9, 40.8, 39.3, 36.4, 30.0, 21.4, 18.1, 2.3. ^{29}Si NMR (119 MHz, $CDCl_3$, 25 ℃) δ 10.3. IR (ATR): ν 3063, 2959, 2877, 2251, 1722, 1625, 1587, 1446, 1408, 1304, 1252, 1144, 1066, 1028, 842, 749, 689 cm^{-1}. HRMS (ESI, m/z): calcd for $C_{22}H_{32}O_5NSSi$ $(M+H)^+$: 450.1765; found: 450.1757.

Cyclobutyl 2-((2-((phenylsulfonyl)methyl)-1-((trimethylsilyl)oxy)cyclopentyl)methyl)acrylate (3u)

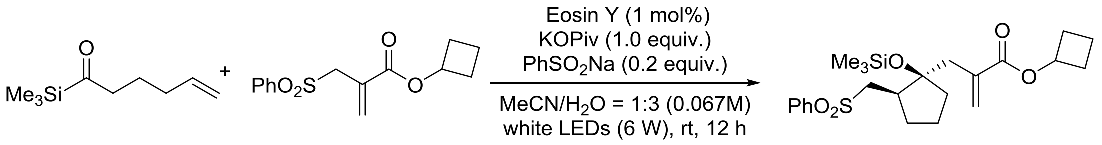

1a **2u** **3u**

In a glovebox, to an oven-dried 10 mL tube was added **2u** (28 mg, 0.1 mmol), $PhSO_2Na$ (3.2 mg, 0.06 mmol, 0.2 equiv.), Eosin Y (0.65 mg, 0.001 mmol, 1 mol%), KOPiv (14.2 mg, 0.1 mmol, 1 equiv.), $MeCN/H_2O$ =1:3 (0.067 M) and **1a** (34 mg, 0.2 mmol, 2 equiv.) sequentially. The tube was sealed, then irradiated with 6 W white LED lamps. The mixture was stirred under white light irradiation at ambient temperature for the 12 h. Then the light was turned off. The resulting mixture was filtered through a thin silica gel plug with EA (30 mL) as the eluent. The organic phase was concentrated under reduced pressure. The crude product was purified with column chromatography on silica gel (300~400 mesh) with PE/EA = 5/1 (v/v) as eluent to afford the title compound as a colorless oil (41.1 mg, 91 % yield in total, a mixture of two disteraosiomers).

Charaterization of the major isomer: R_f = 0.50 (PE/EA = 5/1 (v/v)). NMR Spectroscopy: ^{1}H NMR (600 MHz, $CDCl_3$, 25 ℃) δ 7.96–7.91 (m, 2H), 7.67–7.63 (m, 1H), 7.60–7.55 (m, 2H), 6.16 (d, *J* = 1.4 Hz, 1H), 5.39 (d, *J* = 1.3 Hz, 1H), 5.07–4.96 (m, 1H), 3.42 (dd, *J* = 14.4, 2.0 Hz, 1H), 2.98 (dd, *J* = 14.4, 10.6 Hz, 1H), 2.68 (dd, *J* = 13.4, 0.8 Hz, 1H), 2.47 (dd, *J* = 13.4, 0.9 Hz, 1H), 2.42–2.32 (m, 2H), 2.15–2.04 (m, 2H), 2.03–1.96 (m, 1H), 1.96–1.88 (m, 1H), 1.85–1.77 (m, 1H), 1.72–1.60 (m, 3H), 1.55–1.43 (m, 3H), 0.09 (s, 9H). ^{13}C NMR (151 MHz, $CDCl_3$, 25 ℃) δ 166.9, 140.5, 136.7, 133.6, 129.4, 129.2, 128.1, 86.0, 69.5, 58.0, 40.7, 39.1, 36.4, 30.4, 29.8, 21.6, 13.7, 2.3. ^{29}Si NMR (119 MHz, $CDCl_3$, 25 ℃) δ 10.1. IR (ATR): ν 3093, 3063, 2955, 1714, 1625, 1446, 1405, 1304, 1252, 1148, 1066, 1025, 946, 842, 749, 689 cm^{-1}. HRMS (ESI, m/z): calcd for $C_{23}H_{35}O_5SSi$ $(M+H)^+$: 451.1969; found: 451.1964.

Naphthalen-1-ylmethyl 2-((2-((phenylsulfonyl)methyl)-1-((trimethylsilyl)oxy)cyclopent yl)methyl)acrylate (3v)

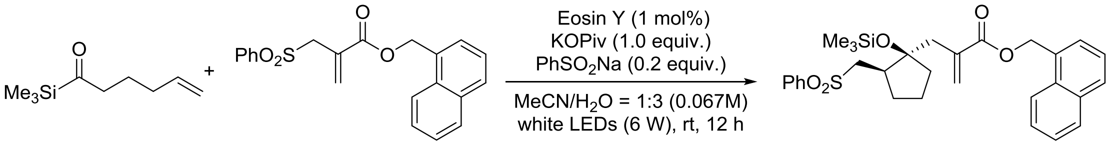

1a **2v** **3v**

In a glovebox, to an oven-dried 10 mL tube was added **2v** (36.6 mg, 0.1 mmol), $PhSO_2Na$ (3.2 mg, 0.02 mmol, 0.2 equiv.), Eosin Y (0.65 mg, 0.001 mmol, 1 mol%), KOPiv (14.2 mg, 0.1 mmol, 1 equiv.), MeCN/H_2O =1:3 (0.067 M) and **1a** (34 mg, 0.2 mmol, 2 equiv.) sequentially. The tube was sealed, then irradiated with 6 W white LED lamps. The mixture was stirred under white light irradiation at ambient temperature for the 12 h. Then the light was turned off. The resulting mixture was filtered through a thin silica gel plug with EA (30 mL) as the eluent. The organic phase was concentrated under reduced pressure. The crude product was purified with column chromatography on silica gel (300~400 mesh) with PE/EA = 5/1 (v/v) as eluent to afford the title compound as a colorless oil (35.1 mg, 65 % yield in total, a mixture of two disteraosiomers).

Charaterization of the major isomer: R_f = 0.48 (PE/EA = 5/1 (v/v)). NMR Spectroscopy: ^{1}H NMR (600 MHz, $CDCl_3$, 25 ℃) δ 8.04–8.02 (m, 1H), 7.94–7.91 (m, 2H), 7.91–7.88 (m, 1H), 7.89–7.84 (m, 1H), 7.63–7.59 (m, 1H), 7.58–7.49 (m, 5H), 7.46 (dd, *J* = 8.2, 7.0 Hz, 1H), 6.14 (d, *J* = 1.3 Hz, 1H), 5.71–5.61 (m, 2H), 5.40 (d, *J* = 1.2 Hz, 1H), 3.45 (dd, *J* = 14.4, 1.9 Hz, 1H), 2.98 (dd, *J* = 14.4, 10.6 Hz, 1H), 2.66 (dd, *J* = 13.3, 0.8 Hz, 1H), 2.54 (dd, *J* = 13.4, 0.8 Hz, 1H), 2.03–1.97 (m, 1H), 1.98–1.89 (m, 1H), 1.71–1.57 (m, 2H), 1.56–1.39 (m, 3H), 0.03 (s, 9H). ^{13}C NMR (151 MHz, $CDCl_3$, 25 ℃) δ 167.5, 140.4, 136.5, 133.9, 133.5, 131.9, 131.5, 129.5, 129.5, 129.3, 128.8, 128.1, 127.8, 126.7, 126.1, 125.4, 123.7, 86.0, 65.4, 58.0, 40.8, 39.4, 36.5, 29.9, 21.5, 2.2. ^{29}Si NMR (119 MHz, $CDCl_3$, 25 ℃) δ 10.2. IR (ATR): ν 3060, 2955, 1714, 1625, 1599, 1513, 1446, 1405, 1304, 1252, 1148, 1066, 957, 738, 689 cm^{-1}. HRMS (ESI, m/z): calcd for $C_{30}H_{37}O_5SSi$ $(M+H)^+$: 537.2126; found: 537.2120.

6-Hydroxyhexyl 2-((2-((phenylsulfonyl)methyl)-1-((trimethylsilyl)oxy)cyclopent yl)methyl)acrylate (3w)

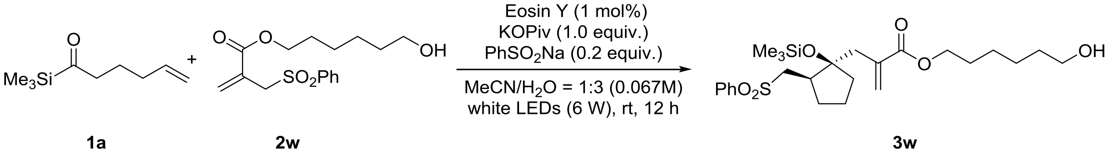

1a **2w** **3w**

In a glovebox, to an oven-dried 10 mL tube was added **2w** (32.6 mg, 0.1 mmol), $PhSO_2Na$ (3.2 mg, 0.02 mmol, 0.2 equiv.), Eosin Y (0.65 mg, 0.001 mmol, 1 mol%), KOPiv (14.2 mg, 0.1 mmol, 1 equiv.), MeCN/H_2O =1:3 (0.067 M) and **1a** (34 mg, 0.2 mmol, 2 equiv.) sequentially. The tube was sealed, then irradiated with 6 W white LED lamps. The mixture was stirred under white light irradiation at ambient temperature for the 12 h. Then the light was turned

off. The resulting mixture was filtered through a thin silica gel plug with EA (30 mL) as the eluent. The organic phase was concentrated under reduced pressure. The crude product was purified with column chromatography on silica gel (300~400 mesh) with PE/EA = 5/1 (v/v) as eluent to afford the title compound as a colorless oil (41.1 mg, 82 % yield in total, a mixture of two disteraosiomers).

Charaterization of the major isomer: R_f = 0.2 (PE/EA = 2/1 (v/v)). NMR Spectroscopy: ^{1}H NMR (600 MHz, $CDCl_3$, 25 ℃) δ 7.96–7.92 (m, 2H), 7.67–7.62 (m, 1H), 7.60–7.55 (m, 2H), 6.15 (d, *J* = 1.4 Hz, 1H), 5.42 (d, *J* = 1.3 Hz, 1H), 4.18–4.12 (m, 2H), 3.69–3.60 (m, 2H), 3.44 (dd, *J* = 14.4, 2.0 Hz, 1H), 2.98 (dd, *J* = 14.3, 10.6 Hz, 1H), 2.67 (dd, *J* = 13.3, 0.7 Hz, 1H), 2.52 (dd, *J* = 13.3, 0.9 Hz, 1H), 2.03–1.96 (m, 1H), 1.97–1.89 (m, 1H), 1.73–1.64 (m, 4H), 1.60–1.54 (m, 2H), 1.54–1.45 (m, 2H), 1.44–1.39 (m, 4H), 1.33 (d, *J* = 5.0 Hz, 1H), 0.09 (s, 9H). ^{13}C NMR (151 MHz, $CDCl_3$, 25 ℃) δ 167.7, 140.5, 136.8, 133.6, 129.4, 129.0, 128.1, 86.0, 65.2, 63.0, 58.0, 40.8, 39.4, 36.4, 32.7, 29.9, 28.7, 25.9, 25.5, 21.6, 2.3. ^{29}Si NMR (119 MHz, $CDCl_3$, 25 ℃) δ 10.1. IR (ATR): ν 3544, 2940, 2862, 1714, 1625, 1446, 1408, 1446, 1408, 1304, 1252, 1148, 1066, 1025, 957, 842, 749, 689 cm^{-1}. HRMS (ESI, m/z): calcd for $C_{25}H_{41}O_6SSi$ $(M+H)^+$: 497.2388; found: 497.2384.

3-Hydroxy-3-methylbutyl 2-((2-((phenylsulfonyl)methyl)-1-((trimethylsilyl)oxy)cyclopentyl)methyl)acrylate (3x)

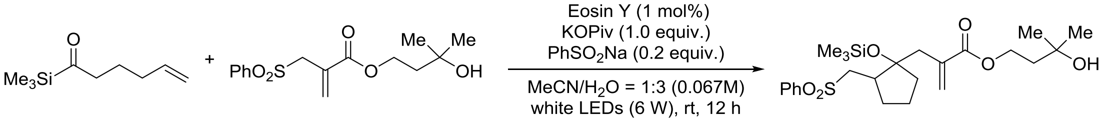

1a **2x** **3x**

In a glovebox, to an oven-dried 10 mL tube was added **2x** (31.2 mg, 0.1 mmol), $PhSO_2Na$ (3.2 mg, 0.02 mmol, 0.2 equiv.), Eosin Y (0.65 mg, 0.001 mmol, 1 mol%), KOPiv (14.2 mg, 0.1 mmol, 1 equiv.), $MeCN/H_2O$ =1:3 (0.067 M) and **1a** (34 mg, 0.2 mmol, 2 equiv.) sequentially. The tube was sealed, then irradiated with 6 W white LED lamps. The mixture was stirred under white light irradiation at ambient temperature for the 12 h. Then the light was turned off. The resulting mixture was filtered through a thin silica gel plug with EA (30 mL) as the eluent. The organic phase was concentrated under reduced pressure. The crude product was purified with column chromatography on silica gel (300~400 mesh) with PE/EA = 5/1 (v/v) as eluent to afford the title compound as a colorless oil (42.5 mg, 88 % yield in total, a mixture of two disteraosiomers).

Charaterization of the major isomer: R_f = 0.38 (PE/EA = 2/1 (v/v)). NMR Spectroscopy: ^{1}H NMR (600 MHz, $CDCl_3$, 25 ℃) δ 7.98–7.90 (m, 2H), 7.67–7.61 (m, 1H), 7.58–7.54 (m, 2H), 6.14 (d, *J* = 1.3 Hz, 1H), 5.45 (d, *J* = 1.2 Hz, 1H), 4.40 (dt, *J* = 11.2, 6.9 Hz, 1H), 4.29 (dt, *J* = 11.2, 6.9 Hz, 1H), 3.46 (dd, *J* = 14.3, 1.6 Hz, 1H), 2.95 (dd, *J* = 14.2, 10.4 Hz, 1H), 2.68–2.54 (m, 2H), 2.01–1.91 (m, 2H), 1.89 (td, *J* = 6.9, 0.9 Hz, 2H), 1.73–1.63 (m, 2H), 1.61–1.54 (m, 1H), 1.53–1.45 (m, 2H), 1.27 (d, *J* = 3.0 Hz, 6H), 0.08 (s, 9H). ^{13}C NMR (151 MHz, $CDCl_3$, 25 ℃) δ 167.6, 140.5, 136.8, 133.6, 129.3, 128.9, 128.0, 86.0, 70.0, 62.2, 57.9, 41.7, 40.8, 39.3, 36.3, 29.9, 29.8, 29.8, 21.4, 2.3. ^{29}Si NMR (119 MHz, $CDCl_3$, 25 ℃) δ 10.1. IR (ATR): ν 3511, 3064, 2963, 1711, 1625, 1446, 1304, 1252, 1141, 1066, 1025, 943, 839, 745 cm^{-1}. HRMS (ESI, m/z): calcd for $C_{24}H_{39}O_6SSi$ $(M+H)^+$: 483.2231; found: 483.2231.

6-Oxohexyl 2-((2-((phenylsulfonyl)methyl)-1-((trimethylsilyl)oxy)cyclopentyl)methyl)acrylate (3y)

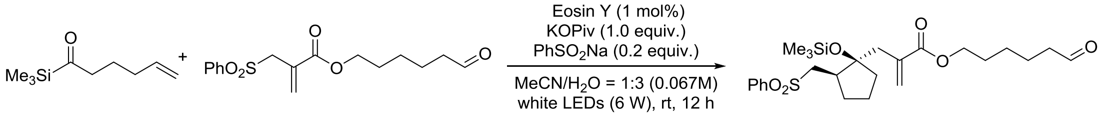

1a **2y** **3y**

In a glovebox, to an oven-dried 10 mL tube was added **2y** (34 mg, 0.1 mmol), $PhSO_2Na$ (3.2 mg, 0.02 mmol, 0.2 equiv.), Eosin Y (0.65 mg, 0.001 mmol, 1 mol%), KOPiv (14.2 mg, 0.1 mmol, 1 equiv.), MeCN/H_2O =1:3 (0.067 M) and **1a** (34 mg, 0.2 mmol, 2 equiv.) sequentially. The tube was sealed, then irradiated with 6 W white LED lamps. The mixture was stirred under white light irradiation at ambient temperature for the 12 h. Then the light was turned off. The resulting mixture was filtered through a thin silica gel plug with EA (30 mL) as the eluent. The organic phase was concentrated under reduced pressure. The crude product was purified with column chromatography on silica gel (300~400 mesh) with PE/EA = 5/1 (v/v) as eluent to afford the title compound as a colorless oil (39.6 mg, 80 % yield in total, a mixture of two disteraosiomers).

Charaterization of the major isomer: R_f = 0.43 (PE/EA = 3/1 (v/v)). NMR Spectroscopy: ^1H NMR (600 MHz, $CDCl_3$, 25 ℃) δ 9.77 (t, *J* = 1.7 Hz, 1H), 7.95–7.92 (m, 2H), 7.67–7.63 (m, 1H), 7.59–7.54 (m, 2H), 6.15 (d, *J* = 1.3 Hz, 1H), 5.43 (d, *J* = 1.3 Hz, 1H), 4.20–4.11 (m, 2H), 3.44 (dd, *J* = 14.4, 1.9 Hz, 1H), 2.97 (dd, *J* = 14.4, 10.5 Hz, 1H), 2.68–2.64 (m, 1H), 2.53 (dd, *J* = 13.3, 0.8 Hz, 1H), 2.46 (td, *J* = 7.3, 1.7 Hz, 2H), 2.02–1.96 (m, 1H), 1.95–1.90 (m, 1H), 1.74–1.63 (m, 6H), 1.58–1.52 (m, 1H), 1.49 (ddt, *J* = 9.2, 7.8, 6.0 Hz, 2H), 1.45–1.37 (m, 2H), 0.09 (s, 9H). ^{13}C NMR (151 MHz, $CDCl_3$, 25 ℃) δ 202.4, 167.6, 140.5, 136.8, 133.6, 129.4, 129.0, 128.1, 86.0, 64.9, 58.0, 43.8, 40.8, 39.3, 36.4, 29.9, 28.5, 25.7, 21.8, 21.5, 2.3. ^{29}Si NMR (119 MHz, $CDCl_3$, 25 ℃) δ 10.1. IR (ATR): ν 3063, 2955, 2870, 2721, 1718, 1628, 1446, 1408, 1304, 1252, 1148, 1069, 1025, 957, 842 cm^{-1}. HRMS (ESI, m/z): calcd for $C_{25}H_{39}O_6SSi$ $(M+H)^+$: 495.2231; found: 495.2227.

(1R,2S,5R)-2-Isopropyl-5-methylcyclohexyl-2-(((1S)-2-((phenylsulfonyl)methyl)-1-((trimethyllsilyl)oxy)cyclopentyl)methyl)acrylate (3z)

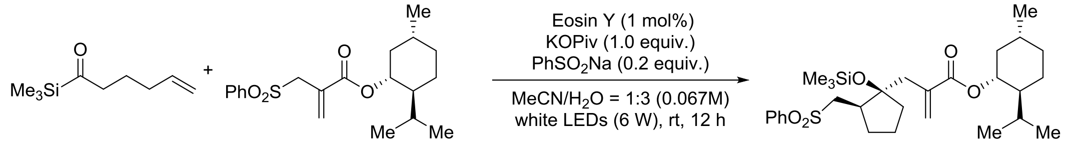

1a **2z** **3z**

In a glovebox, to an oven-dried 10 mL tube was added **2z** (35.7 mg, 0.1 mmol), $PhSO_2Na$ (3.2 mg, 0.02 mmol, 0.2 equiv.), Eosin Y (0.65 mg, 0.001 mmol, 1 mol%), KOPiv (14.2 mg, 0.1 mmol, 1 equiv.), MeCN/H_2O =1:3 (0.067 M) and **1a** (34 mg, 0.2 mmol, 2 equiv.) sequentially. The tube was sealed, then irradiated with 6 W white LED lamps. The mixture was stirred under white light irradiation at ambient temperature for the 12 h. Then the light was turned off. The resulting mixture was filtered through a thin silica gel plug with EA (30 mL) as the eluent.The organic phase was concentrated under reduced pressure. The crude product was purified with column chromatography on silica gel (300~400 mesh) with PE/EA = 5/1 (v/v) as eluent to afford the title compound as a colorless oil (30.3 mg, 56 % yield in total, a mixture of two disteraosiomers).

Charaterization of the major isomer: R_f = 0.44 (PE/EA = 5/1 (v/v)). NMR Spectroscopy [mixture of rotamers]: ^1H NMR (600 MHz, $CDCl_3$, 25 ℃) δ 7.96–7.91 (m, 2H), 7.68–7.63 (m, 1H), 7.61–7.55 (m, 2H), 6.12 (dd, *J* = 10.8, 1.4 Hz, 1H), 5.34 (t, *J* = 1.2 Hz, 1H), 4.74–4.68 (m, 1H), 3.42–3.33 (m, 1H), 3.04–2.99 (m, 1H), 2.76 (dt, *J* = 13.3, 1.0 Hz, 1H), 2.40 (ddd, *J* = 30.4, 13.3, 0.8 Hz, 1H), 2.08–1.96 (m, 2H), 1.92–1.82 (m, 2H), 1.74–1.63 (m, 4H), 1.55–1.39 (m, 5H), 1.10–1.03 (m, 1H), 1.02–0.94 (m, 1H), 0.91–0.88 (m, 8H), 0.74 (dd, *J* = 6.9, 1.3 Hz, 3H), 0.10 (d, *J* = 2.7 Hz, 9H). ^{13}C NMR (151 MHz, $CDCl_3$, 25 ℃) δ 167.1, 167.0 (C'), 140.4, 140.3(C'), 137.1, 137.0(C'), 133.7, 133.6(C'), 129.4, 129.4(C'), 129.0, 128.8(C'), 128.2, 128.1(C'), 86.0, 86.0(C'), 75.1, 75.1(C'), 58.2, 58.1(C'), 47.4, 47.3(C'), 41.0, 40.9, 40.6(C'), 39.1, 39.0(C'), 36.4, 36.3(C'), 34.4, 34.4(C'), 31.6, 31.5(C'), 29.9, 29.8(C'), 26.6, 26.4(C'), 23.6, 23.5(C'), 22.2, 21.7, 21.7(C'), 21.0, 20.9(C'), 16.5, 16.4(C'), 2.3, 2.3(C'). ^{29}Si NMR (119 MHz,

CDCl3, 25 ℃) δ 10.2, 10.1(Si’). IR (ATR): ν 2959, 2929, 2873, 1710, 1628, 1446, 1408, 1371, 1308, 1252, 1174, 1148, 1066, 1025, 842, 749, 689 cm^{-1}. HRMS (ESI, m/z): calcd for $C_{29}H_{47}O_5SSi$ $(M+H)^+$: 535.2908; found: 535.2913.

(8R,9S,10R,13S,14S,17S)-10,13-dimethyl-3-oxo-2,3,6,7,8,9,10,11,12,13,14,15,16,17-tetradecahydro-1H-cyclopenta[a]phenanthren-17-yl 2-((2-((phenylsulfonyl)methyl)-1-((trimethylsilyll)oxy)cyclopentyl)methyl)acrylate (3aa)

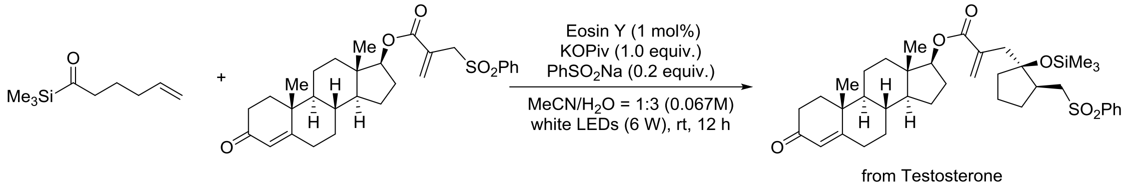

1a **2aa** from Testosterone **3aa**

In a glovebox, to an oven-dried 10 mL tube was added **2aa** (49.6 mg, 0.1 mmol), $PhSO_2Na$ (3.2 mg, 0.02 mmol, 0.2 equiv.), Eosin Y (0.65 mg, 0.001 mmol, 1 mol%), KOPiv (14.2 mg, 0.1 mmol, 1 equiv.), MeCN/H_2O =1:3 (0.067 M) and **1a** (34 mg, 0.2 mmol, 2 equiv.) sequentially. The tube was sealed, then irradiated with 6 W white LED lamps. The mixture was stirred under white light irradiation at ambient temperature for the 12 h. Then the light was turned off. The resulting mixture was filtered through a thin silica gel plug with EA (30 mL) as the eluent. The organic phase was concentrated under reduced pressure. The crude product was purified with column chromatography on silica gel (300~400 mesh) with PE/EA = 5/1 (v/v) as eluent to afford the title compound as a colorless oil (34.1 mg, 52 % yield in total, a mixture of two disteraosiomers).

Charaterization of the major isomer: R_f = 0.22 (PE/EA = 4/1 (v/v)). NMR Spectroscopy [mixture of rotamers]: ^{1}H NMR (600 MHz, CDCl3, 25 ℃) δ 7.97–7.91 (m, 2H), 7.68–7.62 (m, 1H), 7.60–7.53 (m, 2H), 6.15 (dd, *J* = 10.9, 1.4 Hz, 1H), 5.73 (t, *J* = 2.3 Hz, 1H), 5.43 (dd, *J* = 4.0, 1.3 Hz, 1H), 4.66–4.61 (m, 1H), 3.44–3.40 (m, 1H), 2.97 (dd, *J* = 14.4, 10.7 Hz, 1H), 2.67 (d, *J* = 13.3 Hz, 1H), 2.52–2.46 (m, 1H), 2.45–2.34 (m, 3H), 2.34–2.26 (m, 1H), 2.25–2.18 (m, 1H), 2.04–2.00 (m, 2H), 1.98–1.91 (m, 1H), 1.89–1.83 (m, 1H), 1.81–1.77 (m, 1H), 1.73–1.69 (m, 4H), 1.64–1.45 (m, 7H), 1.43–1.33 (m, 2H), 1.27–1.21 (m, 1H), 1.20–1.18 (m, 3H), 1.13–1.00 (m, 2H), 0.98–0.93 (m, 1H), 0.88 (d, *J* = 4.3 Hz, 3H), 0.08 (s, 9H). ^{13}C NMR (151 MHz, CDCl3, 25 ℃) δ 199.6, 199.6(C’), 171.0, 167.5, 167.5(C’), 140.5, 140.5(C’), 137.0, 137.0(C’), 133.6, 133.6(C’), 129.4, 128.9, 128.7(C’), 128.1, 128.1(C’), 124.1, 86.0, 86.0(C’), 83.3, 83.2(C’), 58.1, 58.0(C’), 53.8, 53.8(C’), 50.4, 50.3(C’), 43.0, 42.8(C’), 41.1, 40.9(C’), 39.6, 39.4(C’), 38.7, 36.9, 36.8(C’), 36.7, 36.5(C’), 35.8, 35.8(C’), 35.6, 35.5(C’), 34.1, 32.9, 31.6, 30.0, 29.9(C’), 27.7, 27.6(C’), 23.7, 23.7(C’), 21.7, 21.6(C’), 20.7, 20.7(C’), 17.6, 12.4, 12.4(C’), 2.4, 2.3(C’). ^{29}Si NMR (119 MHz, CDCl3, 25 ℃) δ 10.0. IR (ATR): ν 2944, 2877, 2851, 1714, 1673, 1617, 1446, 1416, 1308, 1252, 1170, 1147, 1069, 842, 689 cm^{-1}. HRMS (ESI, m/z): calcd for $C_{38}H_{55}O_6SSi$ $(M+H)^+$: 667.3483; found: 667.3480.

2-Methylene-1-phenylnon-8-ene-1,4-dione 5a

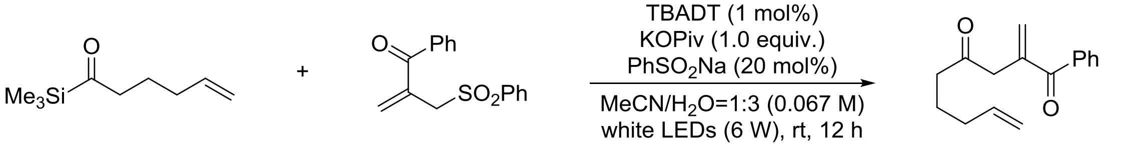

1a **2a** **5a**

In a glovebox, to an oven-dried 10 mL tube was added **2a** (57.2 mg, 0.2 mmol), sodium benzenesulfinate (3.6 mg, 0.04 mmol, 20 mol%), TBADT (6.6 mg, 0.002 mmol, 1 mol%), KOPiv (28.4 mg, 0.2 mmol, 1 equiv.), MeCN/H_2O

=1:3 (0.067 M) and **1a** (92.8 mg, 0.4 mmol, 2 equiv.) sequentially. The tube was sealed, then irradiated with 6 W white LED lamps. The mixture was stirred under white light irradiation at ambient temperature for the 12 h. Then the light was turned off. The resulting mixture was filtered through a thin silica gel plug with EA (30 mL) as the eluent. The organic phase was concentrated under reduced pressure. The crude product was purified with column chromatography on silica gel (200~300 mesh) with PE/EA = 10/1 (v/v) as eluent to afford the title compound as a colorless oil (7.7 mg, 14 % yield).

R_f = 0.48 (PE/EA = 10/1 (v/v)). NMR Spectroscopy: ^{1}H NMR (600 MHz, $CDCl_3$, 25 °C) δ 7.83–7.80 (m, 2H), 7.56–7.52 (m, 1H), 7.46–7.42 (m, 2H), 5.92 (q, *J* = 1.0 Hz, 1H), 5.78 (s, 1H), 5.77–5.72 (m, 1H), 5.05–4.95 (m, 2H), 3.61 (d, *J* = 1.0 Hz, 2H), 2.54 (t, *J* = 7.4 Hz, 2H), 2.09–2.04 (m, 2H), 1.70 (p, *J* = 7.4 Hz, 2H). ^{13}C NMR (151 MHz, $CDCl_3$, 25 °C) δ 207.8, 197.5, 142.1, 138.1, 137.4, 132.4, 129.9, 129.3, 128.3, 115.4, 46.6, 41.9, 33.1, 22.7. IR (ATR): ν 2933, 2875, 1714, 1654, 1446, 1341, 1216, 1170, 1118, 1077, 984, 752, 708 cm^{-1}. HRMS (APCI+, m/z): calcd for $C_{16}H_{18}O_2$ $(M+H)^+$: 243.1380; found: 243.1377.

3.4 Scope for the *γ*-substituted cyclopentanol derivatives

1-Phenyl-2-((3-(phenylsulfonyl)-1-((trimethylsilyl)oxy)cyclopentyl)methyl)prop-2-en-1-one (7a)

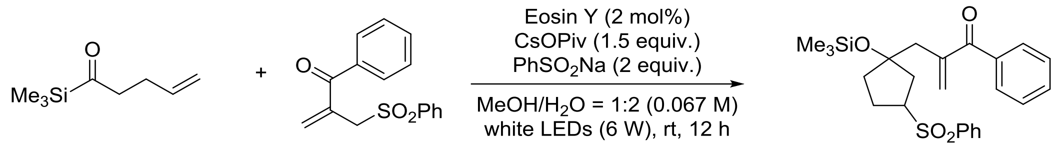

1f **2a** **7a**

In a glovebox, to an oven-dried 10 mL tube was added **2a** (28.6 mg, 0.1 mmol), $PhSO_2Na$ (32.8 mg, 0.2 mmol, 2 equiv.), Eosin Y (1.3 mg, 0.002 mmol, 2 mol%), CsOPiv (35.1 mg, 0.15 mmol, 1.5 equiv.), $MeOH/H_2O$ =1:2 (0.067 M) and **1f** (31.2 mg, 0.2 mmol, 2 equiv.) sequentially. The tube was sealed, then irradiated with 6 W white LED lamps. The mixture was stirred under white light irradiation at ambient temperature for the 12 h. Then the light was turned off. The resulting mixture was filtered through a thin silica gel plug with EA (30 mL) as the eluent. The organic phase was concentrated under reduced pressure. The crude product was purified with column chromatography on silica gel (300~400 mesh) with PE/EA = 5/1 (v/v) as eluent to afford the title compound as a colorless oil (27.5 mg, 62 % yield in total, a mixture of two disteraosiomers).

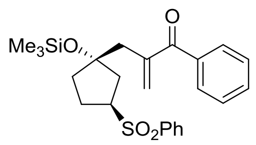

7a

R_f = 0.50 (PE/EA = 5/1 (v/v)). NMR Spectroscopy: ^{1}H NMR (600 MHz, $CDCl_3$, 25 ℃) δ 7.85–7.81 (m, 2H), 7.80–7.77 (m, 2H), 7.63–7.60 (m, 1H), 7.56–7.49 (m, 3H), 7.46–7.41 (m, 2H), 5.87 (q, *J* = 1.0 Hz, 1H), 5.64 (d, *J* = 1.0 Hz, 1H), 3.76–3.63 (m, 1H), 2.95–2.83 (m, 2H), 2.20–2.09 (m, 2H), 2.07–1.98 (m, 1H), 1.97–1.93 (m, 1H), 1.83–1.74 (m, 2H), 0.00 (s, 9H). ^{13}C NMR (151 MHz, $CDCl_3$, 25 ℃) δ 197.8, 145.1, 138.7, 137.3, 133.7, 132.5, 130.1, 129.3, 128.4, 128.3, 128.0, 84.9, 62.8, 42.7, 40.0, 38.3, 24.9, 2.0. ^{29}Si NMR (119 MHz, $CDCl_3$, 25 ℃) δ 10.5. IR (ATR): ν 3063, 2952, 1654, 1599, 1446, 1304, 1252, 1200, 1144, 1056, 838, 699 cm^{-1}. HRMS (ESI, m/z): calcd for $C_{24}H_{31}O_4SSi$ $(M+H)^+$: 443.1707; found: 443.1704.

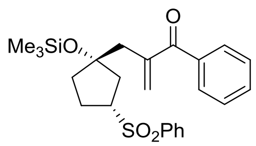

7a'

R_f = 0.35 (PE/EA = 5/1 (v/v)). NMR Spectroscopy: ^{1}H NMR (600 MHz, $CDCl_3$, 25 ℃) δ 7.90–7.85 (m, 2H), 7.73–7.70 (m, 2H), 7.63–7.57 (m, 1H), 7.55–7.49 (m, 3H), 7.43–7.38 (m, 2H), 5.82 (d, *J* = 1.1 Hz, 1H), 5.63 (d, *J* = 1.1 Hz, 1H), 3.73–3.45 (m, 1H), 2.78–2.51 (m, 2H), 2.27–2.14 (m, 2H), 2.03 (ddd, *J* = 13.3, 8.3, 1.5 Hz, 1H), 1.97–1.87 (m, 2H), 1.82–1.75 (m, 1H), 0.01 (s, 9H). ^{13}C NMR (151 MHz, $CDCl_3$, 25 ℃) δ 198.0, 144.6, 138.4, 137.2, 133.7, 132.4, 130.0, 129.3, 128.6, 128.2, 83.3, 61.4, 42.5, 39.0, 37.9, 24.1, 2.1. ^{29}Si NMR (119 MHz, $CDCl_3$, 25 ℃) δ

10.9. IR (ATR): ν 3302, 3063, 2952, 1658, 1446, 1304, 1248, 1144, 1103, 1084, 838, 699 cm^{-1}. HRMS (ESI, m/z): calcd for $C_{24}H_{31}O_4SSi$ $(M+H)^+$: 443.1707; found: 443.1707.

2-((1-Hydroxy-3-(phenylsulfonyl)cyclopentyl)methyl)-1-phenylprop-2-en-1-one (7a')

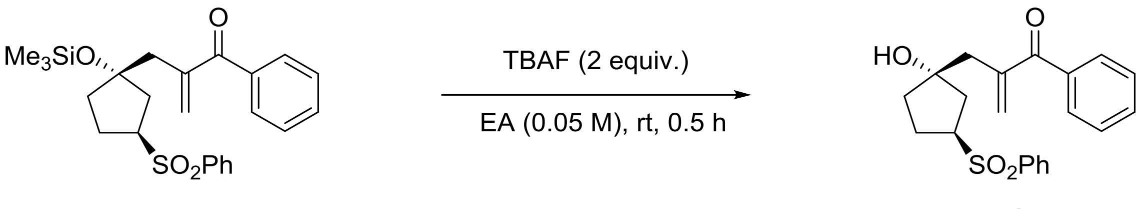

To a 4 mL tube was added **3a** (44.2 mg, 0.1 mmol), EA (0.1 M) and TBAF (0.2 mL, 0.2 mmol, 1 M in THF, 2 equiv.) sequentially. The mixture was stirred at ambient temperature for the 0.5 h. The resulting mixture was filtered through a thin silica gel plug with EA (20 mL) as the eluent. The organic phase was concentrated under reduced pressure. The crude product was purified with column chromatography on silica gel (300~400 mesh) with PE/EA = 2/1 (v/v) as eluent to afford the title compound as a colorless oil (36.4 mg, 98 % yield in total, a mixture of two disteraosiomers). R_f = 0.15 (PE/EA = 2/1 (v/v)). NMR Spectroscopy: ^{1}H NMR (600 MHz, $CDCl_3$, 25 ℃) δ 7.90–7.88 (m, 2H), 7.77–7.75 (m, 2H), 7.65–7.62 (m, 1H), 7.59–7.53 (m, 3H), 7.47–7.43 (m, 2H), 6.05 (s, 1H), 5.79 (s, 1H), 3.88–3.82 (m, 2H), 2.86–2.78 (m, 2H), 2.20 (dddd, *J* = 13.3, 9.4, 5.7, 3.4 Hz, 1H), 2.16–2.09 (m, 2H), 1.95 (ddd, *J* = 13.3, 7.8, 2.3 Hz, 1H), 1.88–1.81 (m, 1H), 1.76 (dddd, *J* = 13.2, 8.0, 3.3, 2.3 Hz, 1H). ^{13}C NMR (151 MHz, $CDCl_3$, 25 ℃) δ 200.5, 144.2, 139.1, 137.0, 133.7, 133.1, 131.2, 130.1, 129.4, 128.5, 128.4, 81.4, 62.9, 43.7, 40.3, 38.9, 24.9. IR (ATR): ν 3496, 3067, 2937, 2858, 1654, 1607, 1446, 1304, 1207, 1148, 1084, 991, 838, 730, 687 cm^{-1}. HRMS (ESI, m/z): calcd for $C_{21}H_{23}O_4S$ $(M+H)^+$: 371.1312; found: 371.1300.

2-((3-(phenylsulfonyl)-1-((trimethylsilyl)oxy)cyclopentyl)methyl)-1-(o-tolyl)prop-2-en-1-one (7b)

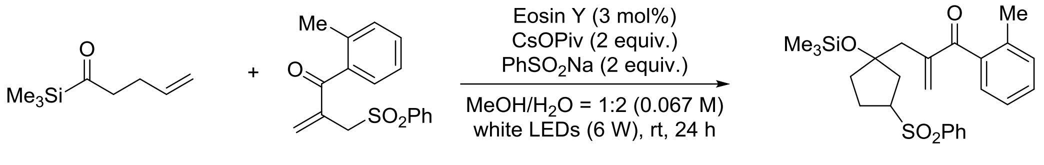

In a glovebox, to an oven-dried 10 mL tube was added **2h** (30 mg, 0.1 mmol), $PhSO_2Na$ (32.8 mg, 0.2 mmol, 2 equiv.), Eosin Y (1.9 mg, 0.003 mmol, 3 mol%), CsOPiv (46.8 mg, 0.2 mmol, 2.0 equiv.), $MeOH/H_2O$ =1:2 (0.067 M) and **1f** (46.8 mg, 0.3 mmol, 3 equiv.) sequentially. The tube was sealed, then irradiated with 6 W white LED lamps. The mixture was stirred under white light irradiation at ambient temperature for the 24 h. Then the light was turned off. The resulting mixture was filtered through a thin silica gel plug with EA (30 mL) as the eluent. The organic phase was concentrated under reduced pressure. The crude product was purified with column chromatography on silica gel (300~400 mesh) with PE/EA = 5/1 (v/v) as eluent to afford the title compound as a colorless oil (26.3 mg, 58 % yield in total, a mixture of two disteraosiomers).

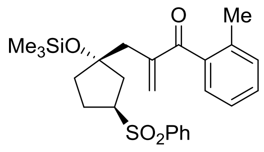

7b

R_f = 0.47 (PE/EA = 5/1 (v/v)). NMR Spectroscopy: ^{1}H NMR (600 MHz, $CDCl_3$, 25 ºC) δ 7.90–7.85 (m, 2H), 7.66–7.63 (m, 1H), 7.58–7.53 (m, 2H), 7.33 (td, *J* = 7.5, 1.5 Hz, 1H), 7.29 (dd, *J* = 7.6, 1.4 Hz, 1H), 7.24–7.18 (m, 2H), 6.02 (d, *J* = 1.0 Hz, 1H), 5.69 (d, *J* = 1.0 Hz, 1H), 3.78–3.67 (m, 1H), 2.94–2.82 (m, 2H), 2.33 (s, 3H), 2.24–2.15 (m, 1H), 2.14–2.02 (m, 2H), 1.97 (ddd, *J* = 13.4, 7.8, 1.9 Hz, 1H), 1.87–1.76 (m, 2H), 0.09 (s, 9H). ^{13}C NMR (151 MHz, $CDCl_3$, 25 ºC) δ 199.9, 146.3, 138.9, 138.5, 136.9, 133.7, 132.3, 131.0, 130.2, 129.4, 128.8, 128.5, 125.2, 85.1, 62.9, 40.3, 40.1, 38.3, 24.8, 20.1, 2.2. ^{29}Si NMR (119 MHz, $CDCl_3$, 25 ºC) δ 10.3. IR (ATR): ν 3063, 2952, 1662, 1446, 1408, 1304, 1252, 1196, 1148, 1069, 987, 913, 842, 760, 734 cm^{-1}. HRMS (ESI, m/z): calcd for $C_{25}H_{33}O_4SSi$ $(M+H)^+$: 457.1863; found: 457.1856.

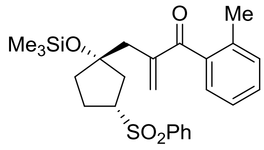

7b'

R_f = 0.38 (PE/EA = 5/1 (v/v)). NMR Spectroscopy: ^{1}H NMR (600 MHz, $CDCl_3$, 25 ºC) δ 7.94–7.88 (m, 2H), 7.66–7.60 (m, 1H), 7.58–7.52 (m, 2H), 7.31 (ddd, *J* = 7.7, 5.1, 3.7 Hz, 1H), 7.23–7.18 (m, 1H), 7.18–7.15 (m, 2H), 6.07 (d, *J* = 1.1 Hz, 1H), 5.72 (d, *J* = 1.2 Hz, 1H), 3.79–3.63 (m, 1H), 2.77–2.48 (m, 2H), 2.33–2.24 (m, 1H), 2.23–2.16 (m, 4H), 2.05–1.91 (m, 3H), 1.84–1.71 (m, 1H), 0.11 (s, 9H). ^{13}C NMR (151 MHz, $CDCl_3$, 25 ºC) δ 200.6, 145.3, 138.7, 138.6, 136.6, 133.7, 133.5, 131.0, 130.1, 129.3, 128.6, 128.5, 125.1, 83.3, 61.3, 39.4, 39.3, 38.2, 23.8, 19.9, 2.3. ^{29}Si NMR (119 MHz, $CDCl_3$, 25 ºC) δ 10.7. IR (ATR): ν 3056, 2959, 1658, 1446, 1304, 1252, 1181, 1148, 1103, 1084, 965, 913, 864, 842, 760 cm^{-1}. HRMS (ESI, m/z): calcd for $C_{25}H_{33}O_4SSi$ $(M+H)^+$: 457.1863; found: 457.1861.

1-(2-Fluorophenyl)-2-((3-(phenylsulfonyl)-1-((trimethylsilyl)oxy)cyclopentyl)methyl)prop-2-en-1-one (7c)

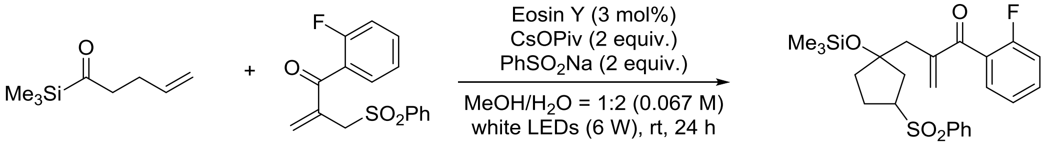

1f **2j** **7c**

In a glovebox, to an oven-dried 10 mL tube was added **2j** (30.4 mg, 0.1 mmol), $PhSO_2Na$ (32.8 mg, 0.2 mmol, 2 equiv.), Eosin Y (1.9 mg, 0.003 mmol, 3 mol%), CsOPiv (46.8 mg, 0.2 mmol, 2 equiv.), $MeOH/H_2O$ =1:2 (0.067 M) and **1f** (46.8 mg, 0.3 mmol, 3 equiv.) sequentially. The tube was sealed, then irradiated with 6 W white LED lamps. The mixture was stirred under white light irradiation at ambient temperature for the 24 h. Then the light was turned off. The resulting mixture was filtered through a thin silica gel plug with EA (30 mL) as the eluent. The organic phase was concentrated under reduced pressure. The crude product was purified with column chromatography on silica gel (300~400 mesh) with PE/EA = 5/1 (v/v) as eluent to afford the title compound as a colorless oil (22.1 mg, 48 % yield in total, a mixture of two disteraosiomers).

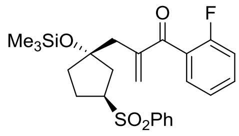

7c

R_f = 0.51 (PE/EA = 5/1 (v/v)). NMR Spectroscopy: ^{1}H NMR (600 MHz, $CDCl_3$, 25 ºC) δ 7.91–7.86 (m, 2H), 7.67–7.61 (m, 1H), 7.58–7.53 (m, 2H), 7.49–7.43 (m, 2H), 7.24–7.17 (m, 1H), 7.14–7.07 (m, 1H), 6.04 (d, *J* = 1.0 Hz, 1H), 5.80–5.76 (m, 1H), 3.77–3.68 (m, 1H), 2.92–2.82 (m, 2H), 2.25–2.15 (m, 1H), 2.11–2.01 (m, 2H), 1.97 (ddd, *J* = 13.5, 7.8, 2.0 Hz, 1H), 1.86–1.76 (m, 2H), 0.07 (s, 9H). ^{13}C NMR (151 MHz, $CDCl_3$, 25 ºC) δ 194.8, 160.2 (d, *J* = 252.8 Hz), 145.8, 138.9, 133.7, 132.9 (d, *J* = 8.5 Hz), 131.5, 130.8 (d, *J* = 2.8 Hz), 129.4, 128.6, 127.0 (d, *J* = 14.3 Hz), 124.1 (d, *J* = 3.7 Hz), 116.4 (d, *J* = 21.8 Hz), 84.8, 63.0, 40.4, 40.2, 38.2, 24.8, 2.1. ^{19}F NMR (565 MHz, $CDCl_3$, 25 ºC) δ −112.0 (dd, *J* = 10.9, 5.3 Hz, 1F). ^{29}Si NMR (119 MHz, $CDCl_3$, 25 ºC) δ 10.3. IR (ATR): ν 2959, 1666, 1610, 1580, 1483, 1449, 1408, 1304, 1252, 1226, 1196, 1148, 1069, 991, 838, 730 cm^{-1}. HRMS (ESI, m/z):

calcd for $C_{24}H_{30}FO_4SSi$ $(M+H)^+$: 461.1613; found: 461.1617.

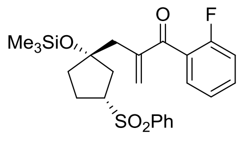

7c'

R_f = 0.45 (PE/EA = 5/1 (v/v)). NMR Spectroscopy: ^{1}H NMR (600 MHz, $CDCl_3$, 25 ℃) δ 7.93–7.88 (m, 2H), 7.65–7.59 (m, 1H), 7.57–7.52 (m, 2H), 7.45 (dddd, *J* = 8.3, 7.2, 5.2, 1.8 Hz, 1H), 7.39–7.34 (m, 1H), 7.18 (td, *J* = 7.5, 1.0 Hz, 1H), 7.08 (ddd, *J* = 9.6, 8.4, 1.1 Hz, 1H), 6.06 (d, *J* = 1.0 Hz, 1H), 5.80 (dd, *J* = 1.9, 0.9 Hz, 1H), 3.67–3.59 (m, 1H), 2.68–2.56 (m, 2H), 2.32–2.22 (m, 1H), 2.19 (ddd, *J* = 13.4, 9.9, 0.9 Hz, 1H), 2.04–1.88 (m, 3H), 1.82–1.73 (m, 1H), 0.10 (s, 9H). ^{13}C NMR (151 MHz, $CDCl_3$, 25 ℃) δ 195.3, 159.9 (d, *J* = 252.1 Hz), 145.0, 138.7, 133.8, 132.8 (d, *J* = 8.2 Hz), 132.4, 130.6 (d, *J* = 3.1 Hz), 129.3, 128.7, 127.0 (d, *J* = 14.9 Hz), 124.2 (d, *J* = 3.5 Hz), 116.3 (d, *J* = 21.8 Hz), 83.2, 61.4, 39.9, 39.1, 38.0, 24.0, 2.2. ^{19}F NMR (565 MHz, $CDCl_3$, 25 ℃) δ –111.89—111.92 (m, 1F). ^{29}Si NMR (119 MHz, $CDCl_3$, 25 ℃) δ 10.7. IR (ATR): ν 3067, 2963, 1662, 1610, 1483, 1449, 1304, 1252, 1148, 1110, 965, 913, 864, 760 cm^{-1}. HRMS (ESI, m/z): calcd for $C_{24}H_{30}FO_4SSi$ $(M+H)^+$: 461.1613; found: 461.1611.

1-(4-Iodophenyl)-2-((3-(phenylsulfonyl)-1-((trimethylsilyl)oxy)cyclopentyl)methyl)prop-2-en-1-one (7d)

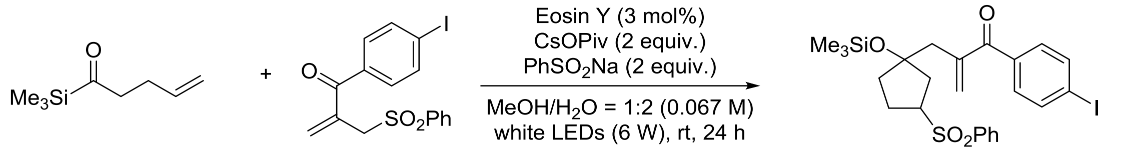

1f **2m** **7d**

In a glovebox, to an oven-dried 10 mL tube was added **2m** (41.1 mg, 0.1 mmol), $PhSO_2Na$ (32.8 mg, 0.2 mmol, 2 equiv.), Eosin Y (1.9 mg, 0.003 mmol, 3 mol%), CsOPiv (46.8 mg, 0.2 mmol, 2 equiv.), $MeOH/H_2O$ =1:2 (0.067 M) and **1f** (46.8 mg, 0.3 mmol, 3 equiv.) sequentially. The tube was sealed, then irradiated with 6 W white LED lamps. The mixture was stirred under white light irradiation at ambient temperature for the 24 h. Then the light was turned off. The resulting mixture was filtered through a thin silica gel plug with EA (30 mL) as the eluent. The organic phase was concentrated under reduced pressure. The crude product was purified with column chromatography on silica gel (300~400 mesh) with PE/EA = 5/1 (v/v) as eluent to afford the title compound as a colorless oil (35.3 mg, 62 % yield in total, a mixture of two disteraosiomers).

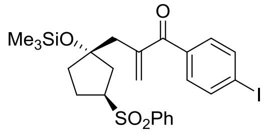

7d

R_f = 0.45 (PE/EA = 5/1 (v/v)). NMR Spectroscopy: ^{1}H NMR (600 MHz, $CDCl_3$, 25 ºC) δ 7.87–7.83 (m, 2H), 7.82–7.78 (m, 2H), 7.67–7.62 (m, 1H), 7.56–7.50 (m, 4H), 5.87 (d, *J* = 0.9 Hz, 1H), 5.60 (d, *J* = 0.9 Hz, 1H), 3.74–3.65 (m, 1H), 2.93–2.85 (m, 2H), 2.20–2.09 (m, 2H), 2.04–1.98 (m, 1H), 1.97–1.92 (m, 1H), 1.82–1.76 (m, 2H), 0.00 (s, 9H). ^{13}C NMR (151 MHz, $CDCl_3$, 25 ºC) δ 197.0, 145.1, 138.8, 137.7, 136.6, 133.8, 131.5, 129.4, 128.5, 128.0, 100.4, 84.9, 62.8, 42.8, 39.9, 38.4, 25.0, 2.0. ^{29}Si NMR (119 MHz, $CDCl_3$, 25 ºC) δ 10.7. IR (ATR): ν 3063, 2955, 2862, 1658, 1621, 1580, 1479, 1446, 1390, 1304, 1252, 1203, 1148, 1069, 842, 730cm^{-1}. HRMS (ESI, m/z): calcd for $C_{24}H_{30}IO_4SSi$ $(M+H)^+$: 569.0673; found: 569.0676.

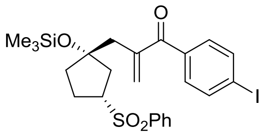

7d'

R_f = 0.38 (PE/EA = 5/1 (v/v)). NMR Spectroscopy: ^{1}H NMR (600 MHz, $CDCl_3$, 25 ℃) δ 7.90–7.87 (m, 2H), 7.80–7.76 (m, 2H), 7.66–7.59 (m, 1H), 7.56–7.52 (m, 2H), 7.47–7.42 (m, 2H), 5.81 (d, *J* = 1.0 Hz, 1H), 5.59 (d, *J* = 1.0 Hz, 1H), 3.59–3.51 (m, 1H), 2.68–2.56 (m, 2H), 2.31–2.20 (m, 1H), 2.18 (ddd, *J* = 13.3, 10.2, 0.9 Hz, 1H), 2.01 (ddd, *J* = 13.3, 8.2, 1.6 Hz, 1H), 1.97–1.85 (m, 2H), 1.80–1.75 (m, 1H), 0.01 (s, 9H). ^{13}C NMR (151 MHz, $CDCl_3$, 25 ℃) δ 197.2, 144.5, 138.5, 137.6, 136.6, 133.8, 131.5, 129.4, 128.7, 128.1, 100.2, 83.3, 61.5, 42.6, 39.0, 37.9, 24.1, 2.1. ^{29}Si NMR (119 MHz, $CDCl_3$, 25 ℃) δ 11.2. IR (ATR): ν 3063, 2959, 1662, 1580, 1429, 1446, 1390,

1304, 1252, 1148, 1103, 1084, 1058, 987, 913, 838, 730 cm^{-1}. HRMS (ESI, m/z): calcd for $C_{24}H_{30}IO_4SSi$ $(M+H)^+$: 569.0673; found: 569.0676.

2-((3-(Phenylsulfonyl)-1-((trimethylsilyl)oxy)cyclopentyl)methyl)-1-(2-(trifluoromethyl)phenyl)-prop-2-en-1-one (7e)

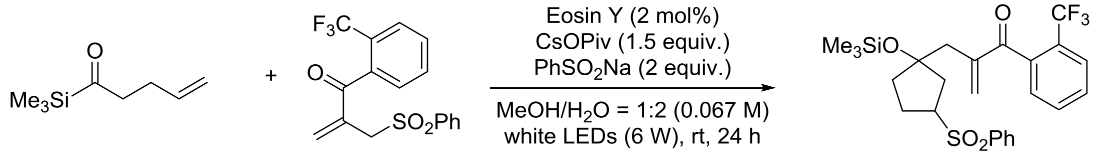

1f **2n** **7e**

In a glovebox, to an oven-dried 10 mL tube was added **2n** (70.8 mg, 0.2 mmol), $PhSO_2Na$ (65.6 mg, 0.4 mmol, 2 equiv.), Eosin Y (2.6 mg, 0.004 mmol, 2 mol%), CsOPiv (70.2 mg, 0.3 mmol, 1.5 equiv.), $MeOH/H_2O$ =1:2 (0.067 M) and **1f** (62.4 mg, 0.4 mmol, 2 equiv.) sequentially. The tube was sealed, then irradiated with 6 W white LED lamps. The mixture was stirred under white light irradiation at ambient temperature for the 24 h. Then the light was turned off. The resulting mixture was filtered through a thin silica gel plug with EA (30 mL) as the eluent. The organic phase was concentrated under reduced pressure. The crude product was purified with column chromatography on silica gel (300~400 mesh) with PE/EA = 5/1 (v/v) as eluent to afford the title compound as a colorless oil (53.8 mg, 52 % yield in total, a mixture of two disteraosiomers).

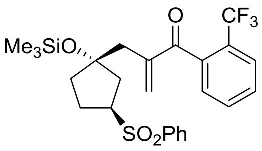

7e

R_f = 0.51 (PE/EA = 5/1 (v/v)). NMR Spectroscopy: ^{1}H NMR (600 MHz, $CDCl_3$, 25 ℃) δ 7.91–7.86 (m, 2H), 7.75–7.70 (m, 1H), 7.68–7.62 (m, 1H), 7.61–7.53 (m, 4H), 7.40–7.36 (m, 1H), 6.20 (d, *J* = 1.1 Hz, 1H), 5.68 (s, 1H), 3.76–3.71 (m, 1H), 2.91–2.83 (m, 2H), 2.24–2.21 (m, 1H), 2.14–2.03 (m, 2H), 1.97 (ddd, *J* = 13.5, 7.8, 2.2 Hz, 1H), 1.89–1.75 (m, 2H), 0.10 (s, 9H). ^{13}C NMR (151 MHz, $CDCl_3$, 25 ℃) δ 197.0, 145.5, 138.8, 138.4, 134.6, 133.7, 131.4, 129.9, 129.4, 128.6, 128.3 (q, *J* = 31.7 Hz), 128.5, 126.7 (q, *J* = 6.1 Hz), 123.7 (q, *J* = 273.3 Hz), 84.9, 63.0, 40.2, 39.3, 38.3, 24.8, 2.2. ^{19}F NMR (565 MHz, $CDCl_3$, 25 ℃) δ –58.2 (s, 3F). ^{29}Si NMR (119 MHz, $CDCl_3$, 25 ℃) δ 10.5. IR (ATR): ν 3068, 2959, 1670, 1580, 1446, 1409, 1312, 1252, 1133, 1059, 984, 913, 838, 730 cm^{-1}. HRMS (ESI, m/z): calcd for $C_{25}H_{30}F_3O_4SSi$ $(M+H)^+$: 511.1581; found: 511.1568.

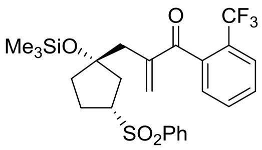

7e'

R_f = 0.41 (PE/EA = 5/1 (v/v)). NMR Spectroscopy: ^{1}H NMR (600 MHz, $CDCl_3$, 25 ℃) δ 7.94–7.88 (m, 2H), 7.71–7.68 (m, 1H), 7.66–7.61 (m, 1H), 7.58–7.52 (m, 4H), 7.25–7.21 (m, 1H), 6.27 (d, *J* = 1.0 Hz, 1H), 5.69 (d, *J* = 0.8 Hz, 1H), 3.74–3.66 (m, 1H), 2.65 (d, *J* = 14.1 Hz, 1H), 2.58–2.53 (m, 1H), 2.33–2.25 (m, 1H), 2.19 (ddd, *J* = 13.2, 10.0, 1.2 Hz, 1H), 2.06–1.95 (m, 2H), 1.92 (ddd, *J* = 13.2, 8.2, 1.7 Hz, 1H), 1.81–1.74 (m, 1H), 0.13 (s, 9H). ^{13}C NMR (151 MHz, $CDCl_3$, 25 ℃) δ 197.8, 144.6, 138.7, 138.2, 135.8, 133.7, 131.3, 129.8, 129.4, 128.6, 128.4, 128.2 (q, *J* = 31.7 Hz),126.7 (q, *J* = 4.5 Hz), 123.6 (q, *J* = 273.3 Hz), 83.3, 61.2, 39.2, 38.5, 38.3, 23.7, 2.3. ^{19}F NMR (565 MHz, $CDCl_3$, 25 ℃) δ –58.2. ^{29}Si NMR (119 MHz, $CDCl_3$, 25 ℃) δ 10.8. IR (ATR): ν 3071, 2959, 1669, 1625, 1584, 1446, 1408, 1315, 1252, 1148, 1062, 1036, 987, 913, 864, 742 cm^{-1}. HRMS (ESI, m/z): calcd for $C_{25}H_{30}F_3O_4SSi$ $(M+H)^+$: 511.1581; found: 511.1580.

1-(4-Methoxyphenyl)-2-((3-(phenylsulfonyl)-1-((trimethylsilyl)oxy)cyclopentyl)methyl)prop-2-en-1-one (7f)

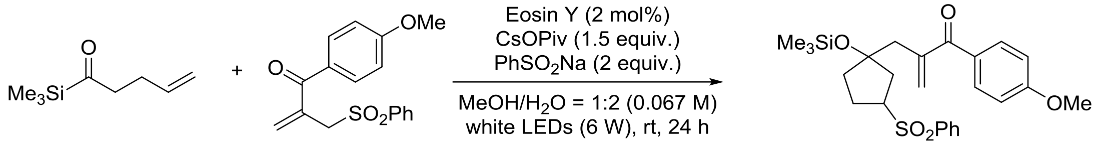

1f **2o** **7f**

In a glovebox, to an oven-dried 10 mL tube was added **2o** (63.2 mg, 0.2 mmol), $PhSO_2Na$ (65.6 mg, 0.4 mmol, 2 equiv.), Eosin Y (2.6 mg, 0.004 mmol, 2 mol%), CsOPiv (70.2 mg, 0.3 mmol, 1.5 equiv.), $MeOH/H_2O$ =1:2 (0.067 M) and **1f** (62.4 mg, 0.4 mmol, 2 equiv.) sequentially. The tube was sealed, then irradiated with 6 W white LED lamps. The mixture was stirred under white light irradiation at ambient temperature for the 24 h. Then the light was turned off. The resulting mixture was filtered through a thin silica gel plug with EA (30 mL) as the eluent. The organic phase was concentrated under reduced pressure. The crude product was purified with column chromatography on silica gel (300~400 mesh) with PE/EA = 5/1 (v/v) as eluent to afford the title compound as a colorless oil (26.3 mg, 58 % yield in total, a mixture of two disteraosiomers).

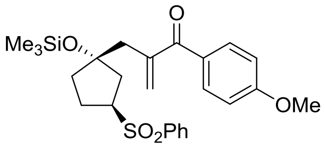

7f

R_f = 0.50 (PE/EA = 4/1 (v/v)). NMR Spectroscopy: ^1H NMR (600 MHz, $CDCl_3$, 25 ℃) δ 7.86–7.76 (m, 4H), 7.64–7.61 (m, 1H), 7.55–7.49 (m, 2H), 6.96–6.90 (m, 2H), 5.78 (d, *J* = 1.1 Hz, 1H), 5.58 (d, *J* = 1.1 Hz, 1H), 3.87 (s, 3H), 3.73–3.65 (m, 1H), 3.00–2.81 (m, 2H), 2.19–2.08 (m, 2H), 2.06–1.97 (m, 1H), 1.93 (ddd, *J* = 13.7, 7.9, 1.4 Hz, 1H), 1.79 (ddd, *J* = 8.5, 6.9, 1.7 Hz, 2H), 0.00 (s, 9H). ^{13}C NMR (151 MHz, $CDCl_3$, 25 ℃) δ 196.5, 163.4, 145.3, 138.8, 133.7, 132.5, 132.2, 129.8, 129.4, 128.5, 126.4, 113.6, 85.0, 62.9, 55.6, 43.2, 40.1, 38.3, 24.9, 2.0. ^{29}Si NMR (119 MHz, $CDCl_3$, 25 ℃) δ 10.5. IR (ATR): ν 3071, 2955, 2844, 1651, 1602, 1509, 1446, 1420, 1304, 1252, 1148, 1069, 1025, 957, 842, 700 cm^{-1}. HRMS (ESI, m/z): calcd for $C_{25}H_{33}O_5SSi$ $(M+H)^+$: 473.1813; found: 473.1807.

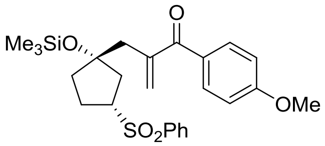

7f'

R_f = 0.38 (PE/EA = 4/1 (v/v)). NMR Spectroscopy: ^1H NMR (600 MHz, $CDCl_3$, 25 ℃) δ 7.90–7.85 (m, 2H), 7.80–7.73 (m, 2H), 7.65–7.59 (m, 1H), 7.55–7.50 (m, 2H), 6.94–6.88 (m, 2H), 5.73 (d, *J* = 1.2 Hz, 1H), 5.57 (d, *J* = 1.3 Hz, 1H), 3.87 (s, 3H), 3.60–3.50 (m, 1H), 2.74–2.57 (m, 2H), 2.29–2.13 (m, 2H), 2.04 (ddd, *J* = 13.3, 8.3, 1.5 Hz, 1H), 1.95–1.83 (m, 2H), 1.78 (ddd, *J* = 9.8, 5.8, 1.5 Hz, 1H), 0.01 (s, 9H). ^{13}C NMR (151 MHz, $CDCl_3$, 25 ℃) δ 196.8, 163.3, 144.8, 138.5, 133.7, 132.5, 129.8, 129.3, 128.7, 126.4, 113.5, 83.3, 61.6, 55.6, 43.2, 39.1, 37.9, 24.2, 2.1. ^{29}Si NMR (119 MHz, $CDCl_3$, 25 ℃) δ 10.8. IR (ATR): ν 2955, 1654, 1602, 1572, 1509, 1446, 1420, 1304, 1259, 1166, 1148, 1107, 1084, 842, 700 cm^{-1}. HRMS (ESI, m/z): calcd for $C_{25}H_{33}O_5SSi$ $(M+H)^+$: 473.1813; found: 473.1810.

2-((3-(Phenylsulfonyl)-1-((trimethylsilyl)oxy)cyclopentyl)methyl)-1-(thiophen-2-yl)prop-2-en-1-one (7g)

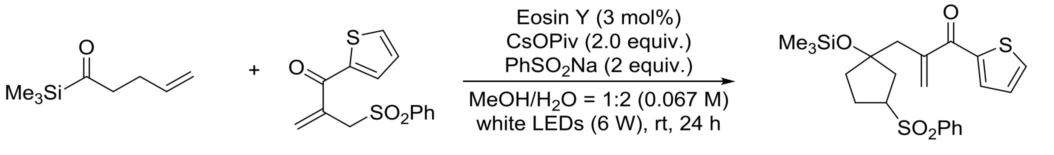

1f **2r** **7g**

In a glovebox, to an oven-dried 10 mL tube was added **2r** (29.2 mg, 0.1 mmol), $PhSO_2Na$ (32.8 mg, 0.2 mmol, 2 equiv.), Eosin Y (1.9 mg, 0.003 mmol, 3 mol%), CsOPiv (46.8 mg, 0.2 mmol, 2 equiv.), $MeOH/H_2O$ =1:2 (0.067 M) and **1f** (46.8 mg, 0.3 mmol, 3 equiv.) sequentially. The tube was sealed, then irradiated with 6 W white LED lamps.

The mixture was stirred under white light irradiation at ambient temperature for the 24 h. Then the light was turned off. The resulting mixture was filtered through a thin silica gel plug with EA (30 mL) as the eluent. The organic phase was concentrated under reduced pressure. The crude product was purified with column chromatography on silica gel (300~400 mesh) with PE/EA = 5/1 (v/v) as eluent to afford the title compound as a colorless oil (32.1 mg, 71 % yield in total, a mixture of two disteraosiomers).

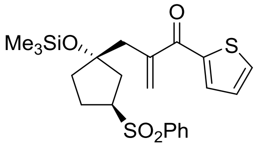

7g

R_f = 0.48 (PE/EA = 5/1 (v/v)). NMR Spectroscopy: ^{1}H NMR (600 MHz, $CDCl_3$, 25 ℃) δ 7.83–7.78 (m, 2H), 7.71–7.65 (m, 2H), 7.66–7.60 (m, 1H), 7.56–7.50 (m, 2H), 7.13 (dd, *J* = 4.9, 3.8 Hz, 1H), 5.83 (d, *J* = 1.0 Hz, 1H), 5.76 (d, *J* = 1.0 Hz, 1H), 3.71–3.63 (m, 1H), 2.93–2.81 (m, 2H), 2.19–2.12 (m, 1H), 2.08 (dd, *J* = 13.6, 9.5 Hz, 1H), 2.06–1.97 (m, 1H), 1.94–1.88 (m, 1H), 1.85–1.77 (m, 2H), 0.00 (s, 9H). ^{13}C NMR (151 MHz, $CDCl_3$, 25 ℃) δ 189.5, 145.5, 143.5, 138.8, 134.6, 134.3, 133.7, 129.4, 128.5, 128.1, 125.7, 84.8, 62.9, 43.4, 40.1, 38.3, 24.84, 1.9. ^{29}Si NMR (119 MHz, $CDCl_3$, 25 ℃) δ 10.6. IR (ATR): ν 3090, 2955, 1640, 1513, 1446, 1412, 1356, 1304, 1252, 1200, 1148, 1069, 842, 699 cm^{-1}. HRMS (ESI, m/z): calcd for $C_{22}H_{29}O_4S_2Si$ $(M+H)^+$: 449.1271; found: 449.1278.

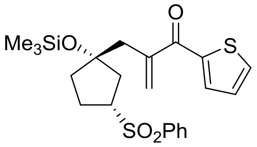

7g'

R_f = 0.42 (PE/EA = 5/1 (v/v)). NMR Spectroscopy: ^{1}H NMR (600 MHz, $CDCl_3$, 25 ℃) δ 7.90–7.86 (m, 2H), 7.68–7.59 (m, 3H), 7.57–7.51 (m, 2H), 7.11 (dd, *J* = 4.9, 3.8 Hz, 1H), 5.84 (d, *J* = 1.1 Hz, 1H), 5.71 (d, *J* = 1.1 Hz, 1H), 3.57–3.48 (m, 1H), 2.69–2.54 (m, 2H), 2.27–2.12 (m, 2H), 2.03 (ddd, *J* = 13.4, 8.3, 1.5 Hz, 1H), 1.95–1.84 (m, 2H), 1.81–1.74 (m, 1H), 0.01 (s, 9H). ^{13}C NMR (151 MHz, $CDCl_3$, 25 ℃) δ 189.6, 145.0, 143.4, 138.5, 134.3, 134.1, 133.8, 129.4, 128.7, 127.9, 125.9, 83.2, 61.5, 43.1, 39.0, 37.8, 24.1, 2.0. ^{29}Si NMR (119 MHz, $CDCl_3$, 25 ℃) δ 10.9. IR (ATR): ν 3082, 2955, 1640, 1513, 1446, 1412, 1304, 1103, 972, 913, 838, 723 cm^{-1}. HRMS (ESI, m/z): calcd for $C_{25}H_{33}O_4SSi$ $(M+H)^+$: 449.1271; found: 449.1280.

1-(Naphthalen-2-yl)-2-((3-(phenylsulfonyl)-1-((trimethylsilyl)oxy)cyclopentyl)methyl)prop-2-en-1-one (7h)

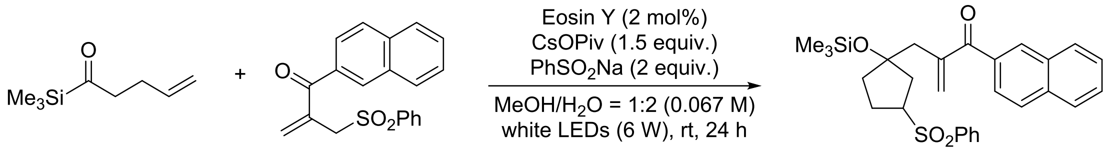

1f **2h'** **7h**

In a glovebox, to an oven-dried 10 mL tube was added **2h'** (67.6 mg, 0.2 mmol), $PhSO_2Na$ (65.6 mg, 0.4 mmol, 2 equiv.), Eosin Y (2.6 mg, 0.004 mmol, 2 mol%), CsOPiv (70.2 mg, 0.3 mmol, 1.5 equiv.), $MeOH/H_2O$ =1:2 (0.067 M) and **1f** (62.4 mg, 0.4 mmol, 2 equiv.) sequentially. The tube was sealed, then irradiated with 6 W white LED lamps. The mixture was stirred under white light irradiation at ambient temperature for the 24 h. Then the light was turned off. The resulting mixture was filtered through a thin silica gel plug with EA (30 mL) as the eluent. The organic phase was concentrated under reduced pressure. The crude product was purified with column chromatography on silica gel (300~400 mesh) with PE/EA = 5/1 (v/v) as eluent to afford the title compound as a colorless oil (50.7 mg, 51 % yield in total, a mixture of two disteraosiomers).

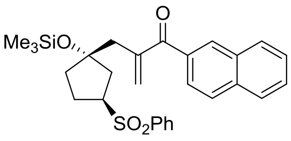

7h

R_f = 0.48 (PE/EA = 5/1 (v/v)). NMR Spectroscopy: ^{1}H NMR (600 MHz, $CDCl_3$, 25 ºC) δ 8.35–8.34 (m, 1H), 7.98 (dd, *J* = 8.0, 1.3 Hz, 1H), 7.89 (dd, *J* = 12.6, 1.0 Hz, 3H), 7.85–7.82 (m, 2H), 7.63–7.52 (m, 3H), 7.51–7.45 (m, 2H), 5.92 (d, *J* = 1.0 Hz, 1H), 5.72 (d, *J* = 1.0 Hz, 1H), 3.77–3.67 (m, 1H), 2.98 (d, *J* = 0.9 Hz, 2H), 2.25–2.13 (m, 2H), 2.09–2.02 (m, 1H), 2.02–1.96 (m, 1H), 1.90–1.79 (m, 2H), 0.04 (s, 9H). ^{13}C NMR

(151 MHz, $CDCl_3$, 25 ºC) δ 197.9, 145.4, 138.8, 135.5, 134.5, 133.7, 132.4, 132.0, 129.8, 129.3, 128.4, 128.4, 128.3, 128.0, 127.9, 126.9, 125.8, 85.1, 62.9, 42.8, 40.0, 38.4, 25.0, 2.1. ^{29}Si NMR (119 MHz, $CDCl_3$, 25 ºC) δ 10.5. IR (ATR): ν 3060, 2955, 1654, 1625, 1595, 1446, 1409, 1304, 1252, 1148, 1069, 998, 913, 842, 700 cm^{-1}. HRMS (ESI, m/z): calcd for $C_{28}H_{33}O_4SSi$ $(M+H)^+$: 493.1863; found: 493.1861.

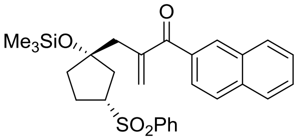

7h'

R_f = 0.30 (PE/EA = 5/1 (v/v)). NMR Spectroscopy: ^{1}H NMR (600 MHz, $CDCl_3$, 25 ºC) δ 8.23 (dd, J = 1.5, 0.8 Hz, 1H), 7.97–7.85 (m, 5H), 7.83 (dd, J = 8.5, 1.7 Hz, 1H), 7.61 (ddd, J = 8.2, 6.9, 1.4 Hz, 1H), 7.58–7.52 (m, 2H), 7.52–7.46 (m, 2H), 5.88 (d, J = 1.1 Hz, 1H), 5.71 (d, J = 1.1 Hz, 1H), 3.66–3.54 (m, 1H), 2.81–2.65 (m, 2H), 2.31–2.16 (m, 2H), 2.08 (ddd, J = 13.3, 8.3, 1.5 Hz, 1H), 2.01–1.90 (m, 2H), 1.87–1.80 (m, 1H), 0.04 (s, 9H). ^{13}C NMR (151 MHz, $CDCl_3$, 25 ºC) δ 198.1, 144.8, 138.5, 135.4, 134.5, 133.7, 132.3, 131.8, 129.6, 129.3, 128.6, 128.4, 128.3, 128.2, 127.9, 126.9, 125.8, 83.4, 61.6, 42.7, 39.3, 38.0, 24.1, 2.2. ^{29}Si NMR (119 MHz, $CDCl_3$, 25 ºC) δ 10.9. IR (ATR): ν 3060, 2955, 2873, 1654, 1625, 1446, 1304, 1252, 1148, 1125, 1103, 1084, 913, 842, 700 cm^{-1}. HRMS (ESI, m/z): calcd for $C_{28}H_{33}O_4SSi$ $(M+H)^+$: 493.1863; found: 493.1860.

Ethyl 2-((3-(phenylsulfonyl)-1-((trimethylsilyl)oxy)cyclopentyl)methyl)acrylate (7i)

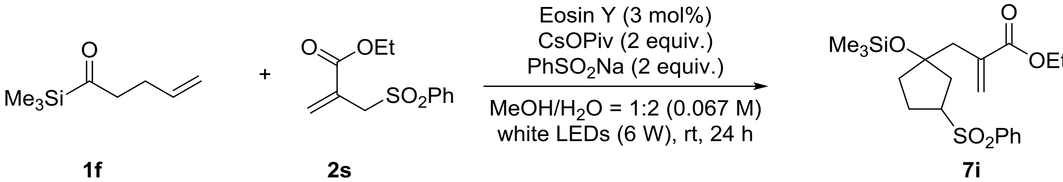

In a glovebox, to an oven-dried 10 mL tube was added **2s** (25.4 mg, 0.1 mmol), $PhSO_2Na$ (32.8 mg, 0.2 mmol, 2 equiv.), Eosin Y (1.9 mg, 0.003 mmol, 3 mol%), CsOPiv (46.8 mg, 0.2 mmol, 2 equiv.), $MeOH/H_2O$ =1:2 (0.067 M) and **1f** (46.8 mg, 0.3 mmol, 3 equiv.) sequentially. The tube was sealed, then irradiated with 6 W white LED lamps. The mixture was stirred under white light irradiation at ambient temperature for the 24 h. Then the light was turned off. The resulting mixture was filtered through a thin silica gel plug with EA (30 mL) as the eluent. The organic phase was concentrated under reduced pressure. The crude product was purified with column chromatography on silica gel (300~400 mesh) with PE/EA = 5/1 (v/v) as eluent to afford the title compound as a colorless oil (19.8 mg, 48 % yield in total, a mixture of two disteraosiomers).

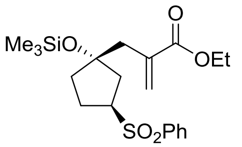

7i

R_f = 0.51 (PE/EA = 5/1 (v/v)). NMR Spectroscopy: ^{1}H NMR (600 MHz, $CDCl_3$, 25 ℃) δ 7.89–7.85 (m, 2H), 7.67–7.62 (m, 1H), 7.58–7.52 (m, 2H), 6.25 (d, J = 1.5 Hz, 1H), 5.63 (d, J = 1.4 Hz, 1H), 4.19 (q, J = 7.1 Hz, 2H), 3.75–3.66 (m, 1H), 2.71 (dd, J = 2.4, 1.0 Hz, 2H), 2.19–2.14 (m, 1H), 2.09–2.02 (m, 1H), 1.99 (dd, J = 13.4, 9.9 Hz, 1H), 1.87 (ddd, J = 13.4, 7.7, 2.3 Hz, 1H), 1.79–1.66 (m, 2H), 1.30 (t, J = 7.1 Hz, 3H), 0.09 (s, 9H). ^{13}C NMR (151 MHz, $CDCl_3$, 25 ℃) δ 167.5, 138.9, 137.1, 133.7, 129.4, 128.5, 128.4, 84.9, 62.9, 61.0, 41.0, 40.1, 37.8, 24.5, 14.4, 2.2. ^{29}Si NMR (119 MHz, $CDCl_3$, 25 ℃) δ 10.0. IR (ATR): ν 3075, 2955, 1718, 1628, 1446, 1304, 1252, 1177, 1148, 1069, 1002, 957, 913, 842, 730 cm^{-1}. HRMS (ESI, m/z): calcd for $C_{20}H_{31}O_5SSi$ $(M+H)^+$: 411.1656; found: 411.1667.

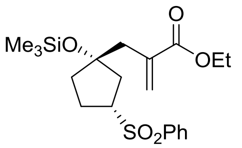

7i'

R_f = 0.46 (PE/EA = 5/1 (v/v)). NMR Spectroscopy: ^{1}H NMR (600 MHz, $CDCl_3$, 25 ℃) δ 7.92–7.86 (m, 2H), 7.66–7.62 (m, 1H), 7.59–7.53 (m, 2H), 6.25 (d, J = 1.8 Hz, 1H), 5.65 (d, J = 1.8 Hz, 1H), 4.13 (q, J = 7.1 Hz, 2H), 3.57 (dtd, J = 9.5, 8.4, 6.1 Hz, 1H), 2.54–2.41 (m, 2H), 2.30–2.20 (m, 1H), 2.13 (ddd, J = 13.3, 9.8, 0.9 Hz, 1H), 1.96–1.82 (m, 3H), 1.75–1.69 (m, 1H), 1.25 (t, J = 7.1 Hz, 3H), 0.11 (s, 9H). ^{13}C NMR (151 MHz, $CDCl_3$, 25 ℃) δ 168.0, 138.7, 136.5, 133.7, 129.3, 128.8, 128.7, 83.2, 61.4, 61.0, 40.7, 39.1, 37.9, 23.9, 14.3, 2.2. ^{29}Si NMR (119 MHz,

CDCl$_3$, 25 ℃) δ 10.5. IR (ATR): ν 2963, 1714, 1628, 1543, 1446, 1304, 1252, 1148, 1088, 1028, 842, 726 cm^{-1}. HRMS (ESI, m/z): calcd for $C_{21}H_{31}O_4SSi$ (M+H)$^+$: 411.1656; found: 411.1661.

3.5 Scope for the synthesis different sulfonyl group-substituted cyclopentyl siloxanes.

1-Phenyl-2-((2-(tosylmethyl)-1-((trimethylsilyl)oxy)cyclopentyl)methyl)prop-2-en-1-one 3ab

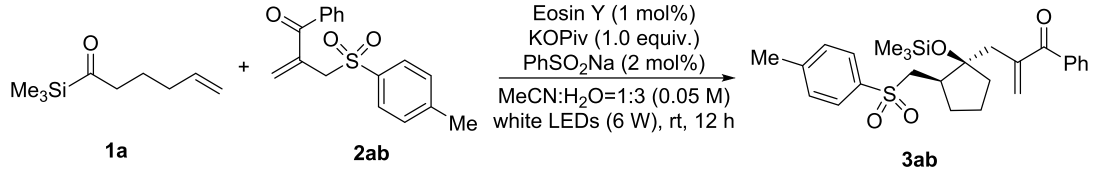

In a glovebox, to an oven-dried 10 mL tube was added **2ab** (60.0 mg, 0.2 mmol), PhSO$_2$Na (0.7 mg, 0.004 mmol, 2 mol%), Eosin Y (1.3 mg, 0.002 mmol, 1 mol%), KOPiv (28.0 mg, 0.2 mmol, 1 equiv.), MeCN/H$_2$O =1:3 (0.05 M) and **1a** (68.0 mg, 0.4 mmol, 2 equiv.) sequentially. The tube was sealed, then irradiated with 6 W white LED lamps. The mixture was stirred under white light irradiation at ambient temperature for the 12 h. Then the light was turned off. The resulting mixture was filtered through a thin silica gel plug with EA (30 mL) as the eluent. The organic phase was concentrated under reduced pressure. The crude product was purified with column chromatography on silica gel (200~300 mesh) with PE/EA = 5/1 (v/v) as eluent to afford the title compound as a colorless oil (75.1 mg, 80 % yield in total, a mixture of two disteraosiomers).

Charaterization of the major isomer: R_f = 0.55 (PE/EA = 5/1 (v/v)). NMR Spectroscopy: ^{1}H NMR (600 MHz, CDCl$_3$, 25 ℃) δ 7.81–7.75 (m, 2H), 7.67–7.61 (m, 2H), 7.57–7.53 (m, 1H), 7.45–7.41 (m, 2H), 7.30–7.26 (m, 2H), 5.79 (d, J = 1.0 Hz, 1H), 5.65 (d, J = 0.8 Hz, 1H), 3.43 (dd, J = 14.4, 2.0 Hz, 1H), 3.01 (dd, J = 14.4, 10.7 Hz, 1H), 2.89–2.82 (m, 1H), 2.74 (dd, J = 13.0, 0.8 Hz, 1H), 2.39 (s, 3H), 2.09–1.91 (m, 2H), 1.76–1.65 (m, 2H), 1.61–1.46 (m, 3H), 0.08 (s, 9H). ^{13}C NMR (151 MHz, CDCl$_3$, 25 ℃) δ 197.4, 144.4, 144.1, 137.2, 137.2, 132.5, 130.7, 129.9, 129.8, 128.4, 128.1, 85.7, 57.9, 41.0, 40.1, 37.0, 29.6, 21.8, 21.6. ^{29}Si NMR (119 MHz, CDCl$_3$, 25 ℃) δ 10.6. IR (ATR): ν 3086, 3063, 2959, 1654, 1599, 1446, 1312, 1252, 1144, 1066, 842, 730 cm^{-1}. HRMS (ESI, m/z): calcd for $C_{26}H_{35}O_4SSi$ (M+H)$^+$: 471.2020; found: 471.2023.

1-Phenyl-2-((2-((o-tolylsulfonyl)methyl)-1-((trimethylsilyl)oxy)cyclopentyl)methyl)prop-2-en-1-one 3ac

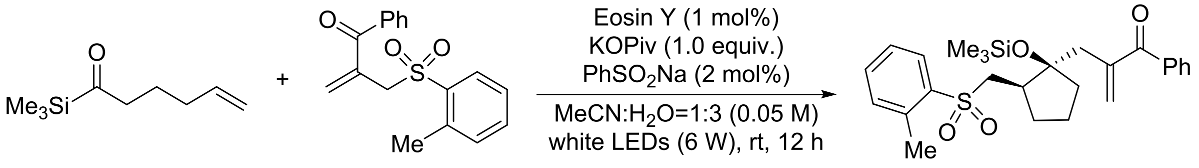

In a glovebox, to an oven-dried 10 mL tube was added **2ac** (60.0 mg, 0.2 mmol), PhSO$_2$Na (0.7 mg, 0.004 mmol, 2 mol%), Eosin Y (1.3 mg, 0.002 mmol, 1 mol%), KOPiv (28.0 mg, 0.2 mmol, 1 equiv.), MeCN/H$_2$O =1/3 (0.05 M) and **1a** (68.0 mg, 0.4 mmol, 2 equiv.) sequentially. The tube was sealed, then irradiated with 6 W white LED lamps. The mixture was stirred under white light irradiation at ambient temperature for the 12 h. Then the light was turned off. The resulting mixture was filtered through a thin silica gel plug with EA (30 mL) as the eluent. The organic phase was concentrated under reduced pressure. The crude product was purified with column chromatography on silica gel (200~300 mesh) with PE/EA = 5/1 (v/v) as eluent to afford the title compound as a colorless oil (81.6 mg, 87 % yield in total, a mixture of two disteraosiomers).

Charaterization of the major isomer: R_f = 0.52 (PE/EA = 5/1 (v/v)). NMR Spectroscopy: ^{1}H NMR (600 MHz, $CDCl_3$, 25 ℃) δ 7.99 (dd, *J* = 7.9, 1.4 Hz, 1H), 7.69–7.66 (m, 2H), 7.57–7.52 (m, 1H), 7.48 – 7.40 (m, 3H), 7.35 (t, *J* = 7.6 Hz, 1H), 7.30 (d, *J* = 7.6 Hz, 1H), 5.82 (s, 1H), 5.68 (s, 1H), 3.45 (dd, *J* = 14.4, 2.1 Hz, 1H), 3.04 (dd, *J* = 14.3, 10.8 Hz, 1H), 2.91 (d, *J* = 13.2 Hz, 1H), 2.72 (d, *J* = 5.6 Hz, 4H), 2.18 (dtd, *J* = 10.8, 8.6, 2.1 Hz, 1H), 1.96 (dtd, *J* = 12.5, 8.5, 3.7 Hz, 1H), 1.69 (qq, *J* = 7.7, 3.7, 3.1 Hz, 2H), 1.62–1.46 (m, 3H), 0.08 (s, 9H). ^{13}C NMR (151 MHz, $CDCl_3$, 25 ℃) δ 197.4, 144.1, 138.2, 138.1, 137.1, 133.6, 132.8, 132.5, 130.3, 130.1, 129.8, 128.4, 126.6, 85.7, 56.8, 40.7, 40.3, 37.1, 29.7, 21.6, 20.6, 2.2. ^{29}Si NMR (119 MHz, $CDCl_3$, 25 ℃) δ 10.73. IR (ATR): ν 3063, 2952, 1654, 1446, 1304, 1252, 1148, 1058, 838, 730 cm^{-1}. HRMS (ESI, m/z): calcd for $C_{26}H_{35}O_4SSi$ $(M+H)^+$: 471.2020; found: 471.2016.

2-((2-(((4-(*tert*-Butyl)phenyl)sulfonyl)methyl)-1-((trimethylsilyl)oxy)cyclopentyl)methyl)-1-phenylprop-2-en-1-one 3ad

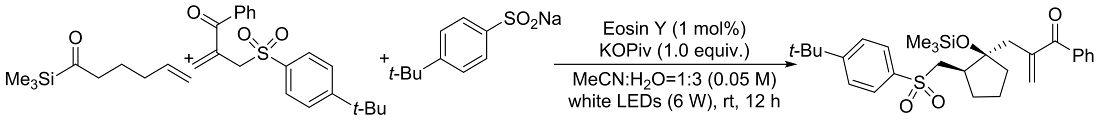

1a **2ad** **3ad**

In a glovebox, to an oven-dried 10 mL tube was added **2ad** (68.4 mg, 0.2 mmol), sodium 4-(tert-butyl)benzenesulfinate (9.9 mg, 0.04 mmol, 20 mol%), Eosin Y (1.3 mg, 0.002 mmol, 1 mol%), KOPiv (28.0 mg, 0.2 mmol, 1 equiv.), MeCN/H_2O =1:3 (0.05 M) and **1a** (68.0 mg, 0.4 mmol, 2 equiv.) sequentially. The tube was sealed, then irradiated with 6 W white LED lamps. The mixture was stirred under white light irradiation at ambient temperature for the 12 h. Then the light was turned off. The resulting mixture was filtered through a thin silica gel plug with EA (30 mL) as the eluent. The organic phase was concentrated under reduced pressure. The crude product was purified with column chromatography on silica gel (200~300 mesh) with PE/EA = 5/1 (v/v) as eluent to afford the title compound as a colorless oil (86.0 mg, 84 % yield in total, a mixture of two disteraosiomers).

Charaterization of the major isomer: R_f = 0.58 (PE/EA = 5/1 (v/v)). NMR Spectroscopy: ^{1}H NMR (600 MHz, $CDCl_3$, 25 ºC) δ 7.88–7.83 (m, 2H), 7.71–7.67 (m, 2H), 7.59–7.52 (m, 3H), 7.47–7.41 (m, 2H), 5.70 (d, *J* = 0.9 Hz, 1H), 5.62 (d, *J* = 0.8 Hz, 1H), 3.43 (dd, *J* = 14.3, 2.0 Hz, 1H), 3.00 (dd, *J* = 14.3, 10.9 Hz, 1H), 2.90 (dd, *J* = 13.2, 0.9 Hz, 1H), 2.69 (dd, *J* = 13.2, 0.9 Hz, 1H), 2.23–2.12 (m, 1H), 2.07–1.98 (m, 1H), 1.75–1.66 (m, 2H), 1.56–1.47 (m, 2H), 1.33 (s, 9H), 1.28–1.23 (m, 1H), 0.07 (s, 9H). ^{13}C NMR (151 MHz, $CDCl_3$, 25 ºC) δ 197.5, 157.5, 144.1, 137.3, 137.2, 132.5, 130.3, 129.8, 128.4, 127.9, 126.4, 85.7, 57.9, 40.8, 40.3, 37.1, 35.4, 31.2, 29.8, 21.7, 2.2. ^{29}Si NMR (119 MHz, $CDCl_3$, 25 ºC) δ 10.64. IR (ATR): ν 3060, 2959, 1654, 1595, 1446, 1401, 1312, 1252, 1151, 1107, 842, 731 cm^{-1}. HRMS (ESI, m/z): calcd for $C_{29}H_{41}O_4SSi$ $(M+H)^+$: 513.2489; found: 513.2492.

2-((2-(((4-Methoxyphenyl)sulfonyl)methyl)-1-((trimethylsilyl)oxy)cyclopentyl)methyl)-1-phenylprop-2-en-1-one 3ae

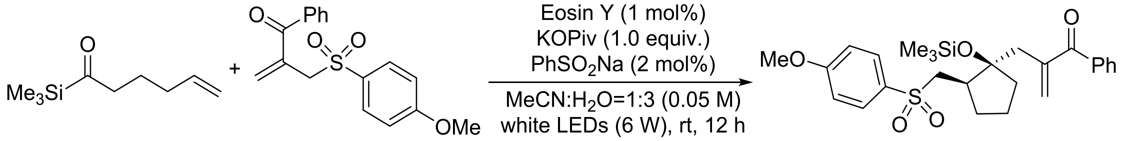

1a **2ae** **3ae**

In a glovebox, to an oven-dried 10 mL tube was added **2ae** (63.2 mg, 0.2 mmol), $PhSO_2Na$ (0.7 mg, 0.004 mmol, 2 mol%), Eosin Y (1.3 mg, 0.002 mmol, 1 mol%), KOPiv (28.0 mg, 0.2 mmol, 1 equiv.), MeCN/H_2O =1:3 (0.05 M) and **1a** (68.0 mg, 0.4 mmol, 2 equiv.) sequentially. The tube was sealed, then irradiated with 6 W white LED lamps.

The mixture was stirred under white light irradiation at ambient temperature for the 12 h. Then the light was turned off. The resulting mixture was filtered through a thin silica gel plug with EA (30 mL) as the eluent. The organic phase was concentrated under reduced pressure. The crude product was purified with column chromatography on silica gel (200~300 mesh) with PE/EA = 5/1 (v/v) as eluent to afford the title compound as a colorless oil (80.0 mg, 82 % yield in total, a mixture of two disteraosiomers).

Charaterization of the major isomer: R_f = 0.32 (PE/EA = 5/1 (v/v)). NMR Spectroscopy: ^{1}H NMR (600 MHz, $CDCl_3$, 25 ℃) ^{1}H NMR (600 MHz, Chloroform-*d*) δ 7.85–7.79 (m, 2H), 7.65–7.61 (m, 2H), 7.56–7.52 (m, 1H), 7.45 – 7.40 (m, 2H), 6.96 – 6.92 (m, 2H), 5.81 (d, *J* = 1.0 Hz, 1H), 5.67 (d, *J* = 0.8 Hz, 1H), 3.84 (s, 3H), 3.43 (dd, *J* = 14.3, 2.0 Hz, 1H), 3.00 (dd, *J* = 14.3, 10.6 Hz, 1H), 2.87 – 2.82 (m, 1H), 2.75 (dd, *J* = 13.2, 0.9 Hz, 1H), 2.02–1.93 (m, 2H), 1.77–1.64 (m, 2H), 1.62 – 1.53 (m, 3H), 1.53–1.46 (m, 1H), 0.08 (s, 9H). ^{13}C NMR (151 MHz, $CDCl_3$, 25 ºC) δ 197.4, 163.6, 144.1, 137.2, 132.5, 131.7, 130.7, 130.2, 129.7, 128.4, 114.4, 85.7, 58.0, 55.7, 41.1, 40.1, 37.0, 29.6, 21.6, 2.3. ^{29}Si NMR (119 MHz, $CDCl_3$, 25 ºC) δ 10.6. IR (ATR): ν 3097, 3063, 2955, 1654, 1595, 1498, 1446, 1412, 1297, 1259, 1140, 1088, 1028, 838, 730 cm^{-1}. HRMS (APCI, m/z): calcd for $C_{26}H_{35}O_5SSi$ $(M+H)^+$: 487.1969; found: 487.1963.

2-((2-(((4-Fluorophenyl)sulfonyl)methyl)-1-((trimethylsilyl)oxy)cyclopentyl)methyl)-1-phenylprop-2-en-1-one 3af

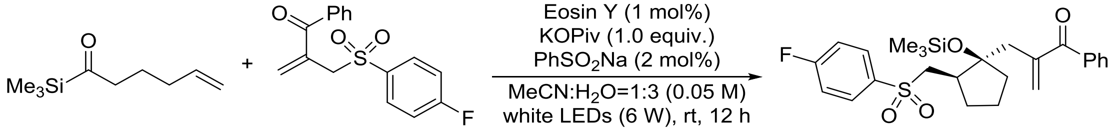

1a **2af** **3af**

In a glovebox, to an oven-dried 10 mL tube was added **2af** (60.8 mg, 0.2 mmol), $PhSO_2Na$ (0.7 mg, 0.004 mmol, 2 mol%), Eosin Y (1.3 mg, 0.002 mmol, 1 mol%), KOPiv (28.0 mg, 0.2 mmol, 1 equiv.), $MeCN/H_2O$ =1:3 (0.05 M) and **1a** (68.0 mg, 0.4 mmol, 2 equiv.) sequentially. The tube was sealed, then irradiated with 6 W white LED lamps. The mixture was stirred under white light irradiation at ambient temperature for the 12 h. Then the light was turned off. The resulting mixture was filtered through a thin silica gel plug with EA (30 mL) as the eluent. The organic phase was concentrated under reduced pressure. The crude product was purified with column chromatography on silica gel (200~300 mesh) with PE/EA = 5/1 (v/v) as eluent to afford the title compound as a colorless oil (80.5 mg, 85 % yield in total, a mixture of two disteraosiomers).

Charaterization of the major isomer: R_f = 0.56 (PE/EA = 5/1 (v/v)). NMR Spectroscopy: ^{1}H NMR (600 MHz, $CDCl_3$, 25 ℃) δ 7.91–7.84 (m, 2H), 7.60–7.58 (m, 2H), 7.57 (d, J = 1.4 Hz, 1H), 7.43 (t, *J* = 7.7 Hz, 2H), 7.11–7.04 (m, 2H), 5.85 (d, *J* = 1.1 Hz, 1H), 5.70 (d, J = 0.9 Hz, 1H). 5.85 (d, *J* = 1.1 Hz, 1H), 5.70 (d, *J* = 0.9 Hz, 1H), 3.47 (dd, *J* = 14.3, 2.2 Hz, 1H), 3.03 (dd, *J* = 14.3, 11.0 Hz, 1H), 2.84 (d, *J* = 13.2 Hz, 1H), 2.75 (d, *J* = 13.2 Hz, 1H), 1.98 (dtd, *J* = 12.5, 8.4, 3.5 Hz, 1H), 1.86 (dtd, *J* = 10.7, 8.9, 8.1, 2.2 Hz, 1H), 1.80–1.59 (m, 5H), 1.56–1.48 (m, 1H), 0.09 (s, 9H). ^{13}C NMR (151 MHz, $CDCl_3$, 25 ℃) δ 197.3, 166.5, 164.8, 144.1, 137.1, 135.9 (d, *J* = 3.2 Hz), 132.6, 131.1, 130.9 (d, *J* = 9.5 Hz), 129.0 (d, *J* = 191.7 Hz), 116.4 (d, *J* = 22.4 Hz), 85.8, 57.7, 41.1, 39.9, 36.6, 29.4, 21.4, 2.3. ^{19}F NMR (565 MHz, $CDCl_3$, 25 ℃) δ –103.73 (tt, *J* = 9.1, 4.8 Hz). ^{29}Si NMR (119 MHz, $CDCl_3$, 25 ℃) δ 10.7. IR (ATR): ν 3071, 2955, 1654, 1591, 1490, 1446, 1405, 1315, 1252, 1140, 1060, 913, 838, 752 cm^{-1}. HRMS (ESI, m/z): calcd for $C_{25}H_{32}FO_4SSi$ $(M+H)^+$:475.1769; found: 475.1758.

2-((2-(((4-Chlorophenyl)sulfonyl)methyl)-1-((trimethylsilyl)oxy)cyclopentyl)methyl)-1-phenylprop-2-en-1-one 3ag

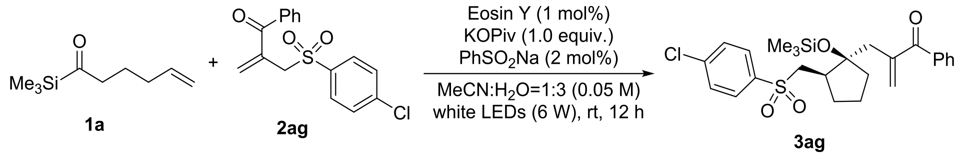

In a glovebox, to an oven-dried 10 mL tube was added **2ag** (64.0 mg, 0.2 mmol), $PhSO_2Na$ (1.6 mg, 0.01 mmol, 5 mol%), Eosin Y (1.3 mg, 0.002 mmol, 1 mol%), KOPiv (28.4 mg, 0.2 mmol, 1 equiv.), MeCN/H_2O =1:3 (0.05 M) and **1a** (68.0 mg, 0.4 mmol, 2 equiv.) sequentially. The tube was sealed, then irradiated with 6 W white LED lamps. The mixture was stirred under white light irradiation at ambient temperature for the 12 h. Then the light was turned off. The resulting mixture was filtered through a thin silica gel plug with EA (30 mL) as the eluent. The organic phase was concentrated under reduced pressure. The crude product was purified with column chromatography on silica gel (200~300 mesh) with PE/EA = 5/1 (v/v) as eluent to afford the title compound as a colorless oil (61.1 mg, 62 % yield in total, a mixture of two disteraosiomers).

Charaterization of the major isomer: R_f = 0.55 (PE/EA = 5/1 (v/v)). NMR Spectroscopy: ^{1}H NMR (600 MHz, $CDCl_3$, 25 ºC) δ 7.83–7.78 (m, 2H), 7.63–7.58 (m, 2H), 7.58–7.55 (m, 1H) 7.46–7.41 (m, 2H), 7.41–7.36 (m, 2H), 5.85 (d, *J* = 1.0 Hz, 1H), 5.71 (d, *J* = 0.8 Hz, 1H), 3.48 (dd, *J* = 14.4, 2.2 Hz, 1H), 3.03 (dd, *J* = 14.3, 11.0 Hz, 1H), 2.84 (dd, *J* = 13.2, 0.9 Hz, 1H), 2.79 – 2.73 (m, 1H), 1.98 (dtd, *J* = 16.8, 8.4, 3.5 Hz, 1H), 1.91–1.85 (m, 1H), 1.77–1.62 (m, 4H), 1.57–1.47 (m, 1H), 0.10 (s, 9H). ^{13}C NMR (151 MHz, $CDCl_3$, 25 ºC) δ 197.3, 144.1, 140.2, 138.5, 137.1, 132.6, 131.1, 129.6, 129.6, 129.5, 128.44, 85.8, 57.7, 41.0, 39.9, 36.7, 29.4, 21.4, 2.3. ^{29}Si NMR (119 MHz, $CDCl_3$, 25 ºC) δ 10.75. IR (ATR): ν 3086, 3060, 3030, 2955, 1654, 1580, 1476, 1446, 1394, 1312, 1252, 1148, 1088, 838, 752 cm^{-1}. HRMS (ESI, m/z): calcd for $C_{25}H_{32}ClO_4SSi$ $(M+H)^+$: 491.1474; found: 491.1469.

2-((2-(((4-Bromophenyl)sulfonyl)methyl)-1-((trimethylsilyl)oxy)cyclopentyl)methyl)-1-phenylprop-2-en-1-one 3ah

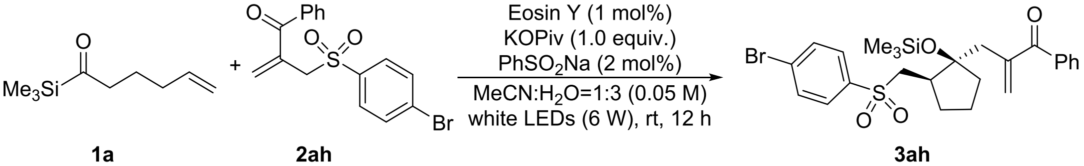

In a glovebox, to an oven-dried 10 mL tube was added **2ah** (73.0 mg, 0.2 mmol), $PhSO_2Na$ (1.6 mg, 0.01 mmol, 5 mol%), Eosin Y (1.3 mg, 0.002 mmol, 1 mol%), KOPiv (28.0 mg, 0.2 mmol, 1 equiv.), MeCN/H_2O =1:3 (0.05 M) and **1a** (68.0 mg, 0.4 mmol, 2 equiv.) sequentially. The tube was sealed, then irradiated with 6 W white LED lamps. The mixture was stirred under white light irradiation at ambient temperature for the 12 h. Then the light was turned off. The resulting mixture was filtered through a thin silica gel plug with EA (30 mL) as the eluent. The organic phase was concentrated under reduced pressure. The crude product was purified with column chromatography on silica gel (200~300 mesh) with PE/EA = 5/1 (v/v) as eluent to afford the title compound as a colorless oil (58.4 mg, 54 % yield in total, a mixture of two disteraosiomers).

Charaterization of the major isomer: R_f = 0.53 (PE/EA = 5/1 (v/v)). NMR Spectroscopy: ^{1}H NMR (600 MHz, $CDCl_3$, 25 ºC) δ 7.76–7.70 (m, 2H), 7.63–7.53 (m, 5H), 7.48–7.42 (m, 2H), 5.85 (d, *J* = 1.0 Hz, 1H), 5.71 (d, *J* = 0.8 Hz, 1H), 3.48 (dd, *J* = 14.3, 2.3 Hz, 1H), 3.03 (dd, *J* = 14.3, 11.0 Hz, 1H), 2.84 (dd, *J* = 13.2, 0.9 Hz, 1H), 2.77 (dd, *J* = 13.1, 0.9 Hz, 1H), 1.98 (dtd, *J* = 12.4, 8.4, 3.3 Hz, 1H), 1.92–1.86 (m, 1H), 1.80–1.63 (m, 4H), 1.57–1.47 (m, 1H), 0.10 (s, 9H). ^{13}C NMR (151 MHz, $CDCl_3$, 25 ºC) δ 197.4, 144.0, 139.0, 137.1, 132.7, 132.5, 131.1, 129.7, 129.7, 128.8, 128.5,

85.8, 57.6, 41.0, 39.9, 36.7, 29.4, 21.4, 2.3. ^{29}Si NMR (119 MHz, $CDCl_3$, 25 ºC) δ 10.76. IR (ATR): ν 3086, 3060, 2955, 1654, 1572, 1468, 1390, 1315, 1252, 1148, 1066, 913, 842, 752 cm^{-1}. HRMS (ESI, m/z): calcd for $C_{25}H_{32}BrO_4SSi$ $(M+H)^+$: 535.0969; found: 535.0965.

2-((2-(([1,1'-Biphenyl]-4-ylsulfonyl)methyl)-1-((trimethylsilyl)oxy)cyclopentyl)methyl)-1-phenylprop-2-en-1-one 3ai

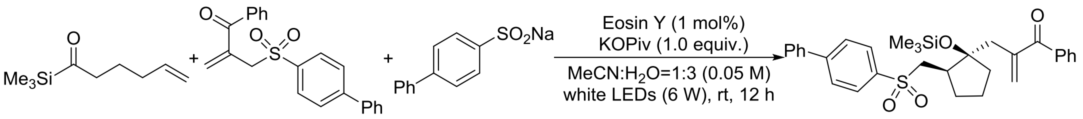

1a **2ai** **3ai**

In a glovebox, to an oven-dried 10 mL tube was added **2ai** (72.4 mg, 0.2 mmol), sodium [1,1'-biphenyl]-4-sulfinate (9.6 mg, 0.04 mmol, 20 mol%), Eosin Y (1.3 mg, 0.002 mmol, 1 mol%), KOPiv (28.0 mg, 0.2 mmol, 1 equiv.), MeCN/H_2O =1:3 (0.05 M) and **1a** (68.0 mg, 0.4 mmol, 2 equiv.) sequentially. The tube was sealed, then irradiated with 6 W white LED lamps. The mixture was stirred under white light irradiation at ambient temperature for the 12 h. Then the light was turned off. The resulting mixture was filtered through a thin silica gel plug with EA (30 mL) as the eluent. The organic phase was concentrated under reduced pressure. The crude product was purified with column chromatography on silica gel (200~300 mesh) with PE/EA = 5/1 (v/v) as eluent to afford the title compound as a colorless oil (30.8 mg, 58 % yield in total, a mixture of two disteraosiomers).

Charaterization of the major isomer: R_f = 0.56 (PE/EA = 5/1 (v/v)). NMR Spectroscopy: ^{1}H NMR (600 MHz, $CDCl_3$, 25 ℃) δ 7.99–7.94 (m, 2H), 7.73–7.68 (m, 2H), 7.67–7.62 (m, 2H), 7.61–7.56 (m, 2H), 7.51–7.43 (m, 4H), 7.37 (t, *J* = 7.6 Hz, 2H), 5.81 (s, 1H), 5.67 (s, 1H), 3.50 (dd, *J* = 14.3, 2.0 Hz, 1H), 3.07 (dd, *J* = 14.3, 10.6 Hz, 1H), 2.86 (d, *J* = 13.2 Hz, 1H), 2.77 (d, *J* = 13.2 Hz, 1H), 2.12–1.99 (m, 2H), 1.78–1.63 (m, 3H), 1.58–1.47 (m, 2H), 0.09 (s, 9H). ^{13}C NMR (151 MHz, $CDCl_3$, 25 ℃) δ 197.4, 146.4, 144.1, 139.3, 138.7, 137.2, 132.5, 130.7, 129.7, 129.2, 128.8, 128.6, 128.4, 127.9, 127.5, 85.7, 57.9, 41.0, 40.2, 37.0, 29.7, 21.6, 2.3. ^{29}Si NMR (119 MHz, $CDCl_3$, 25 ℃) δ 10.7. IR (ATR): ν 3063, 2955, 1654, 1595, 1446, 1304, 1252, 1144, 1066, 842, 752 cm^{-1}. HRMS (ESI, m/z): calcd for $C_{31}H_{37}O_4SSi$ $(M+H)^+$: 533.2176; found: 533.2174.

1-Phenyl-2-((2-(((4-(trifluoromethoxy)phenyl)sulfonyl)methyl)-1-((trimethylsilyl)oxy)cyclo-pent yl)methyl)prop-2-en-1-one 3aj

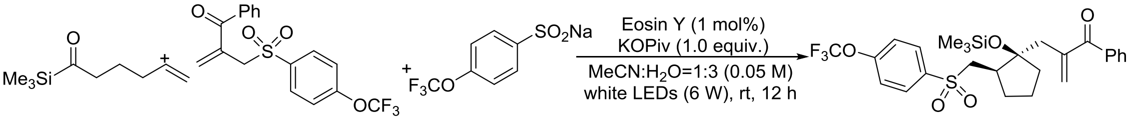

1a **2aj** **3aj**

In a glovebox, to an oven-dried 10 mL tube was added **2aj** (74.0 mg, 0.2 mmol), sodium 4-(trifluoromethoxy)benzenesulfinate (9.6 mg, 0.04 mmol, 20 mol%), Eosin Y (1.3 mg, 0.002 mmol, 1 mol%), KOPiv (28.0 mg, 0.2 mmol, 1 equiv.), MeCN/H_2O =1:3 (0.05 M) and **1a** (68.0 mg, 0.4 mmol, 2 equiv.) sequentially. The tube was sealed, then irradiated with 6 W white LED lamps. The mixture was stirred under white light irradiation at ambient temperature for the 12 h. Then the light was turned off. The resulting mixture was filtered through a thin silica gel plug with EA (30 mL) as the eluent. The organic phase was concentrated under reduced pressure. The crude product was purified with column chromatography on silica gel (200~300 mesh) with PE/EA = 5/1 (v/v) as eluent to afford the title compound as a colorless oil (31.7 mg, 58 % yield in total, a mixture of two disteraosiomers).

Charaterization of the major isomer: R_f = 0.48 (PE/EA = 5/1 (v/v)). NMR Spectroscopy: ^{1}H NMR (600 MHz, $CDCl_3$, 25 ℃) δ 7.96–7.90 (m, 2H), 7.66–7.60 (m, 2H), 7.57–7.54 (m, 1H), 7.45–7.42 (m, 2H), 7.28 (t, *J* = 1.2 Hz, 1H), 5.84

(d, J = 1.0 Hz, 1H), 5.71 (d, J = 0.8 Hz, 1H), 3.49 (dd, J = 14.3, 2.2 Hz, 1H), 3.03 (dd, J = 14.3, 10.9 Hz, 1H), 2.84–2.77 (m, 2H), 2.02–1.94 (m, 2H), 1.76–1.68 (m, 2H), 1.67–1.62 (m, 2H), 1.56–1.51 (m, 1H), 0.10 (s, 9H). ^{13}C NMR (151 MHz, CDCl$_3$, 25 ℃) δ 197.4, 152.8, 144.1, 138.4, 137.1, 132.6, 131.0, 130.4, 129.7, 128.4, 120.9, 120.3 (q, J = 257.8 Hz), 85.8, 57.7, 41.0, 40.0, 36.7, 29.5, 21.5, 2.3. ^{19}F NMR (565 MHz, CDCl$_3$, 25 ℃) δ –57.59. ^{29}Si NMR (119 MHz, CDCl$_3$, 25 ℃) δ 10.8. IR (ATR): ν 3052, 1654, 1587, 1490, 1446, 1297, 1256, 1203, 1148, 1084, 984, 741, 693 cm^{-1}. HRMS (ESI, m/z): calcd for $C_{26}H_{32}F_3O_5SSi$ (M+H)$^+$: 541.1686; found: 541.1678.

1-Phenyl-2-((3-tosyl-1-((trimethylsilyl)oxy)cyclopentyl)methyl)prop-2-en-1-one 7j

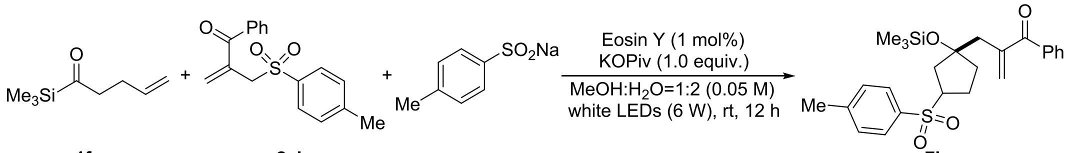

1f **2ab** **7j**

In a glovebox, to an oven-dried 10 mL tube was added **2ab** (60.0 mg, 0.2 mmol), sodium 4-methylbenzenesulfinate (71.2 mg, 0.4 mmol, 2 equiv.), Eosin Y (1.3 mg, 0.002 mmol, 1 mol%), KOPiv (28.0 mg, 0.2 mmol, 1 equiv.), MeOH/H_2O =1:2 (0.075 M) and **1f** (62.4 mg, 0.4 mmol, 2 equiv.) sequentially. The tube was sealed, then irradiated with 6 W white LED lamps. The mixture was stirred under white light irradiation at ambient temperature for the 12 h. Then the light was turned off. The resulting mixture was filtered through a thin silica gel plug with EA (30 mL) as the eluent. The organic phase was concentrated under reduced pressure. The crude product was purified with column chromatography on silica gel (200~300 mesh) with PE/EA = 5/1 (v/v) as eluent to afford the title compound as a colorless oil (47.0 mg, 52 % yield in total, a mixture of two disteraosiomers).

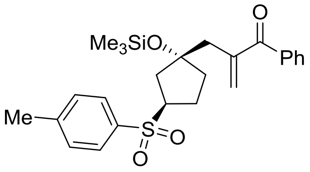

7j

R_f = 0.50 (PE/EA = 5/1 (v/v)). NMR Spectroscopy: ^{1}H NMR (600 MHz, CDCl$_3$, 25 ℃) δ 7.80 (dt, J = 7.3, 1.3 Hz, 2H), 7.73–7.67 (m, 2H), 7.57–7.52 (m, 1H), 7.46–7.43 (m, 2H), 7.31 (d, J = 8.0 Hz, 2H), 5.87 (d, J = 1.2 Hz, 1H), 5.64 (s, 1H), 3.68 (tdd, J = 9.6, 8.0, 5.7 Hz, 1H), 3.00–2.81 (m, 2H), 2.43 (s, 3H), 2.12 (ddd, J = 27.6, 13.9, 8.4 Hz, 2H), 2.07–1.91 (m, 2H), 1.80 (dd, J = 8.7, 6.8 Hz, 2H), 0.00 (s, 9H). ^{13}C NMR (151 MHz, CDCl$_3$, 25 ℃) δ 197.9, 145.2, 144.7, 137.3, 135.7, 132.6, 130.1, 130.0, 128.5, 128.3, 128.0, 85.0, 62.9, 42.7, 40.1, 38.3, 25.0, 21.8, 2.0. ^{29}Si NMR (119 MHz, CDCl$_3$, 25 ℃) δ 10.5. IR (ATR): ν 3060, 2952, 1654, 1595, 1446, 1300, 1252, 1144, 1066, 838, 701 cm^{-1}. HRMS (ESI, m/z): calcd for $C_{25}H_{33}O_4SSi$ (M+H)$^+$: 457.1863; found: 457.1866.

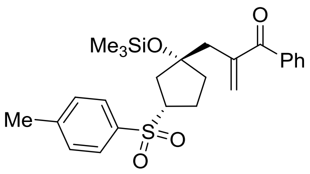

7j'

R_f = 0.47 (PE/EA = 5/1 (v/v)). NMR Spectroscopy: ^{1}H NMR (600 MHz, CDCl$_3$, 25 ℃) δ 7.78–7.70 (m, 4H), 7.53 (ddt, J = 8.7, 7.0, 1.3 Hz, 1H), 7.44–7.38 (m, 2H), 7.33–7.29 (m, 2H), 5.82 (t, J = 1.1 Hz, 1H), 5.63 (d, J = 1.1 Hz, 1H), 3.55 (tdd, J = 9.9, 8.3, 6.3 Hz, 1H), 2.69–2.60 (m, 2H), 2.42 (s, 3H), 2.27–2.13 (m, 2H), 2.03 (ddd, J = 13.3, 8.3, 1.5 Hz, 1H), 1.95–1.85 (m, 2H), 1.84–1.73 (m, 1H), 0.01 (s, 9H). ^{13}C NMR (151 MHz, CDCl$_3$, 25 ℃) δ 198.1, 144.7, 144.6, 137.3, 135.4, 132.5, 130.1, 130.0, 128.7, 128.2, 128.2, 83.3, 61.5, 42.5, 39.1, 37.9, 24.1, 21.8, 2.1. ^{29}Si NMR (119 MHz, CDCl$_3$, 25 ℃) δ 10.9. IR (ATR): ν3056, 2952, 1654, 1595, 1446, 1300, 1252, 1144, 838, 701 cm^{-1}. HRMS (ESI, m/z): calcd for $C_{25}H_{33}O_4SSi$ (M+H)$^+$: 457.1863; found: 457.1864.

2-((3-((4-Fluorophenyl)sulfonyl)-1-((trimethylsilyl)oxy)cyclopentyl)methyl)-1-phenylprop-2-en-1-one 7k

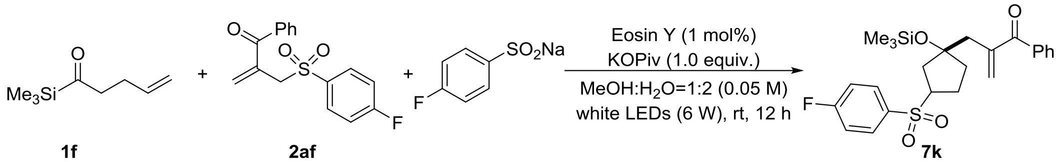

1f 2af 7k

In a glovebox, to an oven-dried 10 mL tube was added **2af** (60.8 mg, 0.2 mmol), sodium 4-fluorobenzenesulfinate (72.8 mg, 0.4 mmol, 2 equiv.), Eosin Y (1.3 mg, 0.002 mmol, 1 mol%), KOPiv (28.0 mg, 0.2 mmol, 1 equiv.), MeOH/H_2O =1:2 (0.075 M) and **1b** (62.4 mg, 0.4 mmol, 2 equiv.) sequentially. The tube was sealed, then irradiated with 6 W white LED lamps. The mixture was stirred under white light irradiation at ambient temperature for the 12 h. Then the light was turned off. The resulting mixture was filtered through a thin silica gel plug with EA (30 mL) as the eluent. The organic phase was concentrated under reduced pressure. The crude product was purified with column chromatography on silica gel (200~300 mesh) with PE/EA = 5/1 (v/v) as eluent to afford the title compound as a colorless oil (51.5 mg, 56 % yield in total, a mixture of two disteraosiomers).

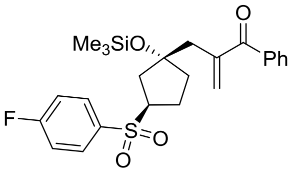

7k

R_f = 0.53 (PE/EA = 5/1 (v/v)). NMR Spectroscopy: ^{1}H NMR (600 MHz, $CDCl_3$, 25 ℃) δ 7.86–7.76 (m, 4H), 7.57–7.54 (m, 1H), 7.48–7.41 (m, 2H), 7.22–7.15 (m, 2H), 5.88 (d, *J* = 1.0 Hz, 1H), 5.65 (d, *J* = 1.0 Hz, 1H), 3.68 (tdd, *J* = 9.7, 7.9, 5.7 Hz, 1H), 2.96–2.86 (m, 2H), 2.15 (ddt, *J* = 13.4, 7.5, 5.5 Hz, 1H), 2.10–2.00 (m, 2H), 1.93 (ddd, *J* = 13.5, 7.9, 1.5 Hz, 1H), 1.86–1.77 (m, 2H), 0.02 (s, 9H). ^{13}C NMR (151 MHz, $CDCl_3$, 25 ℃) δ 197.81, 166.71, 165.01, 145.05, 137.21, 134.78 (d, *J* = 3.3 Hz), 132.66, 131.29 (d, *J* = 9.6 Hz), 129.23 (d, *J* = 257.9 Hz), 128.19, 116.71 (d, *J* = 22.4 Hz), 84.93, 63.06, 42.60, 40.18, 38.27, 24.85, 2.04. ^{29}Si NMR (119 MHz, $CDCl_3$, 25 ℃) δ 10.7. ^{19}F NMR (565 MHz, $CDCl_3$, 25 ℃) δ –102.4—108.2 (m). IR (ATR): ν3075, 2952, 1654, 1591, 1490, 1312, 1248, 1144, 1060, 834, 730 cm^{-1}. HRMS (ESI, m/z): calcd for $C_{24}H_{30}FO_4SSi$ $(M+H)^+$: 461.1613; found: 461.1612.

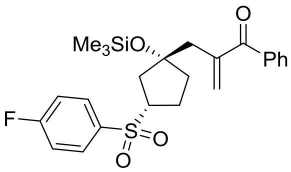

7k'

R_f = 0.55 (PE/EA = 5/1 (v/v)). NMR Spectroscopy: ^{1}H NMR (600 MHz, $CDCl_3$, 25 ℃) δ 7.86–7.82 (m, 2H), 7.69–7.65 (m, 2H), 7.53–7.48 (m, 1H), 7.43–7.35 (m, 2H), 7.19–7.11 (m, 2H), 5.81 (d, *J* = 1.0 Hz, 1H), 5.62 (d, *J* = 1.1 Hz, 1H), 3.58–3.48 (m, 1H), 2.61 (s, 2H), 2.24–2.15 (m, 1H), 2.11 (dd, *J* = 13.3, 9.9 Hz, 1H), 1.98–1.85 (m, 3H), 1.82–1.72 (m, 1H), 0.00 (s, 9H). ^{13}C NMR (151 MHz, $CDCl_3$, 25 ℃) δ 196.0, 164.6, 162.9, 142.3, 135.1, 132.4 (d, *J* = 3.2 Hz), 129.3 (d, *J* = 9.5 Hz), 127.0 (d, *J* = 259.9 Hz), 126.5, 114.5 (d, *J* = 22.7 Hz), 81.1, 59.4, 40.2, 37.0, 35.8, 21.8, –0.01. ^{19}F NMR (565 MHz, $CDCl_3$, 25 ℃) δ –99.1—106.6 (m). ^{29}Si NMR (119 MHz, $CDCl_3$, 25 ℃) δ 11.0. IR (ATR): ν 3071, 2955, 1654, 1591, 1490, 1312, 1248, 1144, 1084, 834, 752 cm^{-1}. HRMS (ESI, m/z): calcd for $C_{24}H_{30}FO_4SSi$ $(M+H)^+$: 461.1613; found: 461.1613.

2-((3-((4-Methoxyphenyl)sulfonyl)-1-((trimethylsilyl)oxy)cyclopentyl)methyl)-1-phenylprop-2-en-1-one 7l

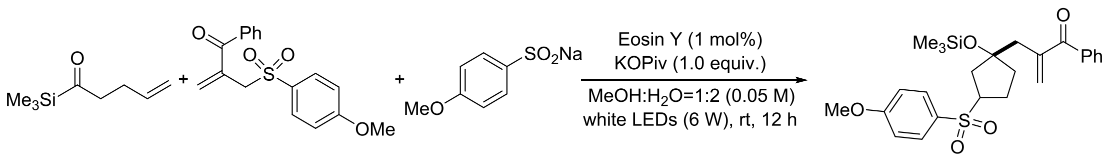

1f 2ae 7l

In a glovebox, to an oven-dried 10 mL tube was added **2ae** (63.2 mg, 0.2 mmol), sodium 4-fluorobenzenesulfinate (77.6 mg, 0.4 mmol, 2 equiv.), Eosin Y (1.3 mg, 0.002 mmol, 1 mol%), KOPiv (28.0 mg, 0.2 mmol, 1 equiv.),

MeOH/H_2O =1:2 (0.075 M) and **1f** (62.4 mg, 0.4 mmol, 2 equiv.) sequentially. The tube was sealed, then irradiated with 6 W white LED lamps. The mixture was stirred under white light irradiation at ambient temperature for the 12 h. Then the light was turned off. The resulting mixture was filtered through a thin silica gel plug with EA (30 mL) as the eluent. The organic phase was concentrated under reduced pressure. The crude product was purified with column chromatography on silica gel (200~300 mesh) with PE/EA = 5/1 (v/v) as eluent to afford the title compound as a colorless oil (55.8 mg, 59 % yield in total, a mixture of two disteraosiomers).

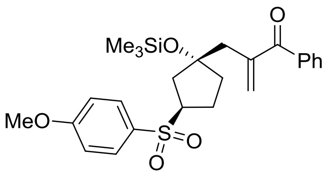

7l

R_f = 0.28 (PE/EA = 5/1 (v/v)). NMR Spectroscopy: ^{1}H NMR (600 MHz, $CDCl_3$, 25 ℃) δ 7.81–7.78 (m, 2H), 7.76–7.72 (m, 2H), 7.56–7.53 (m, 1H), 7.47–7.42 (m, 2H), 6.99–6.96 (m, 2H), 5.87 (d, *J* = 1.0 Hz, 1H), 5.63 (d, *J* = 1.0 Hz, 1H), 3.87 (s, 3H), 3.67 (tdd, *J* = 9.6, 8.0, 5.8 Hz, 1H), 2.99–2.83 (m, 2H), 2.17–2.00 (m, 3H), 1.96 (ddd, *J* = 13.6, 8.0, 1.6 Hz, 1H), 1.83–1.77 (m, 2H), 0.01 (s, 9H). ^{13}C NMR (151 MHz, $CDCl_3$, 25 ℃) δ 197.9, 163.7, 145.2, 137.3, 132.6, 130.6, 130.2, 130.1, 128.3, 128.0, 114.5, 85.0, 63.1, 55.8, 42.7, 40.2, 38.3, 25.0, 2.0. ^{29}Si NMR (119 MHz, $CDCl_3$, 25 ℃) δ 10.5. IR (ATR): ν 3063, 2955, 2844, 1658, 1595, 1498, 1461, 1297, 1259, 1140, 1088, 1025, 834, 699 cm^{-1}. HRMS (ESI, m/z): calcd for $C_{25}H_{33}O_5SSi$ $(M+H)^+$: 473.1813; found: 473.1822.

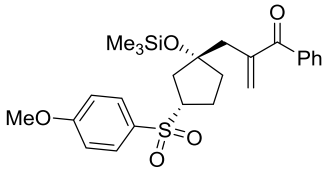

7l'

R_f = 0.30 (PE/EA = 5/1 (v/v)). NMR Spectroscopy: ^{1}H NMR (600 MHz, $CDCl_3$, 25 ºC) δ 7.82–7.77 (m, 2H), 7.76–7.71 (m, 2H), 7.55–7.51 (m, 1H), 7.45–7.39 (m, 2H), 7.00–6.95 (m, 2H), 5.82 (d, *J* = 1.2 Hz, 1H), 5.63 (d, *J* = 1.1 Hz, 1H), 3.86 (s, 3H), 3.53 (dtd, *J* = 9.9, 8.6, 6.2 Hz, 1H), 2.64 (q, *J* = 13.4 Hz, 2H), 2.26 – 2.12 (m, 2H), 2.04 (ddd, *J* = 13.3, 8.3, 1.5 Hz, 1H), 1.95–1.86 (m, 2H), 1.83–1.74 (m, 1H), 0.01 (s, 9H). ^{13}C NMR (151 MHz, $CDCl_3$, 25 ºC) δ 198.1, 163.8, 144.7, 137.3, 132.5, 130.8, 130.1, 129.9, 128.2, 128.2, 114.5, 83.3, 61.7, 55.8, 42.5, 39.2, 37.9, 24.1, 2.1. ^{29}Si NMR (119 MHz, $CDCl_3$, 25 ºC) δ 10.85. IR (ATR): ν 3060, 2922, 2847, 1651, 1595, 1498, 1461, 1297, 1259, 1148, 1107, 1025, 838, 699 cm^{-1}. HRMS (ESI, m/z): calcd for $C_{25}H_{33}O_5SSi$ $(M+H)^+$: 473.1813; found: 473.1812.

4. Down-stream transformations of products

Phenyl(7-(phenylsulfonyl)-3a-((trimethylsilyl)oxy)octahydro-1H-inden-5-yl)methanone (8)

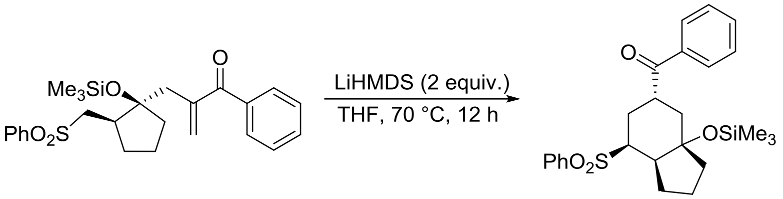

3a **8**

In a glovebox, to an oven-dried 10 mL Schlenk tube equipped with a magnetic stir bar was added **3a** (0.05 mmol, 22.8 mg)，THF (1 mL, dry)，LiHDMS (0.1 mL, 1 M in THF, 2.0 equiv.) The tube was then sealed and moved out of the glovebox，and the resulting mixture was stirred at 70 ºC in a heating block for 12 h, after which the mixture was cooled to room temperature. The reaction mixture was filtered through a thin silica gel plug with EA (30 mL) as the eluent. The organic phase was concentrated under reduced pressure. The crude product was purified with column chromatography on silica gel (300~400 mesh) with PE/EA=5/1 (v/v) as eluent to afford the title compound as a colorless oil (16.4 mg, 82 % yield).

R_f = 0.38 (PE/EA = 5/1 (v/v)). NMR Spectroscopy: ^{1}H NMR (600 MHz, $CDCl_3$, 25 ℃) δ 8.02– 7.97 (m, 2H), 7.91–

7.85 (m, 2H), 7.65–7.61 (m, 1H), 7.61–7.56 (m, 2H), 7.54–7.49 (m, 1H), 7.44–7.39 (m, 2H), 3.88 (td, J = 10.7, 3.7 Hz, 1H), 3.59–3.53 (m, 1H), 2.69–2.57 (m, 1H), 2.22–2.14 (m, 1H), 2.12–2.05 (m, 1H), 1.88 (dd, J = 14.0, 7.8 Hz, 1H), 1.83–1.76 (m, 1H), 1.75–1.61 (m, 4H), 1.62–1.48 (m, 2H), −0.24 (s, 9H). ^{13}C NMR (151 MHz, $CDCl_3$, 25 ℃) δ 200.3, 138.8, 135.0, 133.5, 133.0, 129.2, 129.0, 128.6, 128.6, 82.7, 60.3, 48.0, 40.5, 40.1, 37.0, 27.5, 27.4, 20.9, 1.21. ^{29}Si NMR (119 MHz, $CDCl_3$, 25 ℃) δ 10.4. IR (ATR): ν 3615, 3533, 3339, 3063, 2952, 2881, 1681, 1595, 1446, 1289 cm^{-1}. HRMS (ESI, m/z): calcd for $C_{25}H_{33}O_4SSi$ $(M+H)^+$: 457.1863; found: 457.1871.

Phenyl((1S,5S)-1-(phenylsulfonyl)-5-((trimethylsilyl)oxy)bicyclo[3.2.1]octan-3-yl)methanone (9)

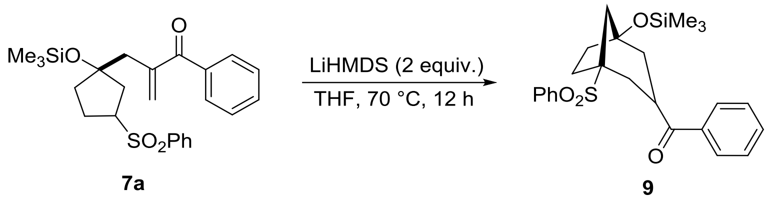

In a glovebox, to an oven-dried 10 mL Schlenk tube equipped with a magnetic stir bar was added **7a** (0.05 mmol, 22.1 mg)， THF (1 mL, dry)， LiHDMS (0.1 mL, 1 M in THF, 2.0 equiv.) The tube was then sealed and moved out of the glovebox， and the resulting mixture was stirred at 70 ºC in a heating block for 12 h, after which the mixture was cooled to room temperature. The reaction mixture was filtered through a thin silica gel plug with EA (30 mL) as the eluent. The organic phase was concentrated under reduced pressure. The crude product was purified with column chromatography on silica gel (300~400 mesh) with PE/EA=5/1 (v/v) as eluent to afford the title compound as a colorless oil (16.3 mg, 74 % yield).

Rf = 0.40 (PE/EA = 5/1 (v/v)). NMR Spectroscopy: ^{1}H NMR (600 MHz, $CDCl_3$, 25 ℃) δ 7.87–7.82 (m, 2H), 7.73–7.68 (m, 2H), 7.68–7.62 (m, 1H), 7.57–7.51 (m, 3H), 7.46–7.41 (m, 2H), 3.71–3.65 (m, 1H), 2.50–2.44 (m, 2H), 2.41–2.31 (m, 2H), 2.12 (ddd, J = 13.5, 9.4, 2.2 Hz, 1H), 2.07–2.01 (m, 1H), 1.76 (ddd, J = 8.2, 6.6, 2.6 Hz, 2H), 1.65–1.50 (m, 2H), 0.08 (s, 9H). ^{13}C NMR (151 MHz, $CDCl_3$, 25 ℃) δ 202.3, 136.2, 136.1, 133.9, 132.9, 130.0, 129.1, 128.9, 128.5, 80.0, 68.1, 44.9, 40.2, 39.7, 35.5, 30.6, 27.0, 2.3. ^{29}Si NMR (119 MHz, $CDCl_3$, 25 ℃) δ 12.1. IR (ATR): ν 3063, 2959, 1684, 1595, 1446, 1345, 1304, 1252, 1222, 1148, 842 cm^{-1}. HRMS (ESI, m/z): calcd for $C_{24}H_{31}O_4SSi$ $(M+H)^+$: 443.1707; found: 443.1717.

5. Mechanism study

5.1 Reaction of 1a and 2a in the presence of TEMPO.

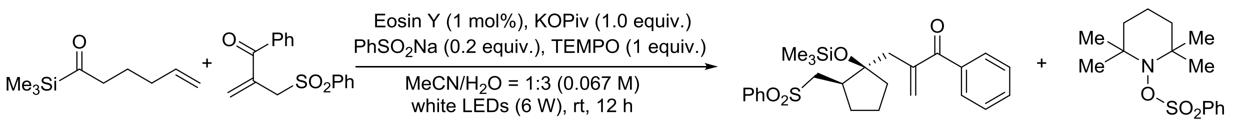

1a (2 equiv.) **2a** 40% conv. of **2a**, 0% yield of **3a** **X**, detected by HRMS

In a glovebox, to an oven-dried 10 mL tube was added **2a** (28.6 mg, 0.1 mmol), $PhSO_2Na$ (3.2 mg, 0.02 mmol, 0.2 equiv.), Eosin Y (0.65 mg, 0.001 mmol, 1 mol%), KOPiv (14.2 mg, 0.1 mmol, 1 equiv.), TEMPO (15.6 mg, 0.1 mmol, 1 equiv.). MeCN/H_2O =1:3 (0.067 M) and **1a** (34.0 mg, 0.2 mmol, 2 equiv.) sequentially. The tube was sealed, then irradiated with 6 W white LED lamps. The mixture was stirred under white light irradiation at ambient temperature for the 12 h. Then the light was turned off. The resulting mixture was filtered through a thin silica gel plug with EA

(30 mL) as the eluent. The organic phase was concentrated under reduced pressure. The conversion of **2a** and yield of **3a** were determined by ^{1}H NMR analysis of the unpurified mixture with $BrCH_2CH_2Br$ as an internal standard.

5.2 Time profile of the transformation with the light ON/OFF over time.

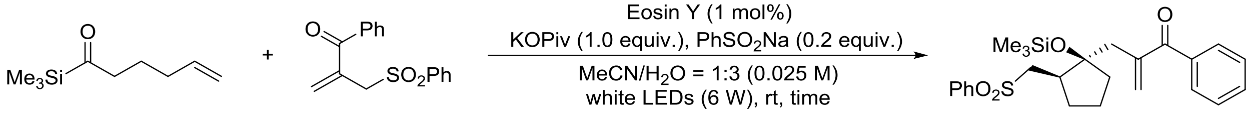

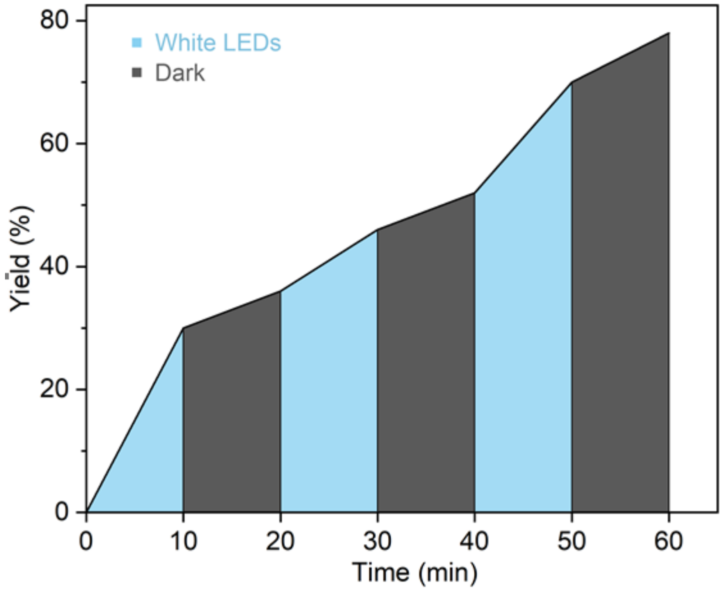

Supplementary Figure 3: Time profile of the transformation with the light ON/OFF over time of **3a**.

In a glovebox, to 6 identical oven-dried 10 mL tubes respectively add **2a** (28.6 mg, 0.1 mmol), $PhSO_2Na$ (3.2 mg, 0.02 mmol, 0.2 equiv.), Eosin Y (0.65 mg, 0.001 mmol, 1 mol%), KOPiv (14.2 mg, 0.1 mmol, 1 equiv.). $MeCN/H_2O$ =1:3 (0.067 M) and **1a** (34.0 mg, 0.2 mmol, 2 equiv.) sequentially. The tube was sealed, then irradiated with 6 W white LED lamps. All the reactions were stirred under blue light irradiation at ambient temperature for the 10 minutes. Then the light was turned off. Remove one of the tubes. The resulting mixture was filtered through a thin silica gel plug with EA (30 mL) as the eluent. The organic phase was concentrated under reduced pressure. The remaining mixture was stirred in the absence of light for an additional 10 minutes. Then remove one of the tubes. The resulting mixture was filtered through a thin silica gel plug with EA (30 mL) as the eluent. NMR analysis was performed every 10 minutes until the reaction time reached 1 h. The conversion of **2a** and yield of **3a** were determined by ^{1}H NMR analysis of the unpurified mixture with $BrCH_2CH_2Br$ as an internal standard.

variation	conv. of **2a**	yield of **3a**
10 min light	35%	22% (dr = 90/10)
10 min light, then 1 h dark	50%	31% (dr = 90/10)
10 min light, then 2 h dark	78%	52% (dr = 90/10)

Supplementary Figure 4: Light and darkness control experiment

In a glovebox, to three identical oven-dried 10 mL tubes respectively added **2a** (28.6 mg, 0.1 mmol), $PhSO_2Na$ (3.2 mg, 0.02 mmol, 0.2 equiv.), Eosin Y (0.65 mg, 0.001 mmol, 1 mol%), KOPiv (14.2 mg, 0.1 mmol, 1 equiv.). MeCN/H_2O =1:3 (0.067 M) and **1a** (34.0 mg, 0.2 mmol, 2 equiv.) sequentially. The tube was sealed, then irradiated with 6 W white LED lamps. All the reactions were stirred under white light irradiation at ambient temperature for the 10 minutes. Then the light was turned off. Remove one of the tubes. The resulting mixture of reaction 1 was filtered through a thin silica gel plug with EA (30 mL) as the eluent. The organic phase was concentrated under reduced pressure. The remaining mixtures of reactions 2 and 3 were stirred in the absence of light for an additional 1 hour and 2 hours. Then remove the tube. The resulting mixture was filtered through a thin silica gel plug with EA (30 mL) as the eluent. The conversion of **2a** and yield of **3a** were determined by ^{1}H NMR analysis of the unpurified mixture with $BrCH_2CH_2Br$ as an internal standard.

5.3 Determination of Quantum Yield

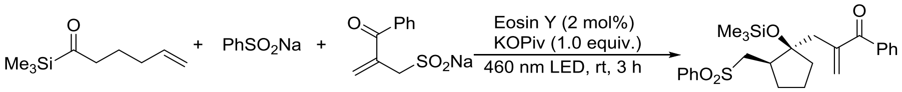

1a **2a** **3a**

In a glovebox, to an oven-dried 10 mL tube was added **2a** (28.6 mg, 0.1 mmol), Eosin Y (1.3 mg, 0.002 mmol, 2 mol%), dry DCM (2 mL) and KOPiv (14.2 mg, 0.1 mmol, 1.0 equiv.) and MeCN (1 mL) and **1a** (34.0 mg, 0.2 mmol, 2 equiv.) and H_2O (3 mL), sequentially. The tube was sealed, the sample was irradiated (λ = 460 nm, slit width = 3.0 mm, slit height 5.0 mm with intensity of 0.662 mW•cm^{-2}) for 10800 s. After irradiation, the yield of product formed was determined by ^{1}H NMR with $BrCH_2CH_2Br$ as an internal standard. The quantum yield was determined as follows.

ϕ = Mole number for product/Mole number for absorption of photons = 1.46

$$\Phi = \frac{n_{3a} N_A/t}{f P \lambda/hc}$$

n**3a**: the mole number of the product **3a**; t: reaction time (10800 s); NA: 6.02×10^{23}/mol; f: $1\text{-}10^{-A}$ (460 nm, A = 4.8); P: P = E*S (E: illumination intensity, E = 0.662 mW/cm^2; S: the area that irradiated S = 0.15 cm^2); λ: wavelength (λ = 4.6×10^{-7}m); h: planck constant (h = 6.626×10^{-34} J*s); c: velocity of light (c = 3×10^{8} m/s). This result reveals that the radical chain process is not main pathway.

5.4 Experiments on the light-induced decomposition of 1a

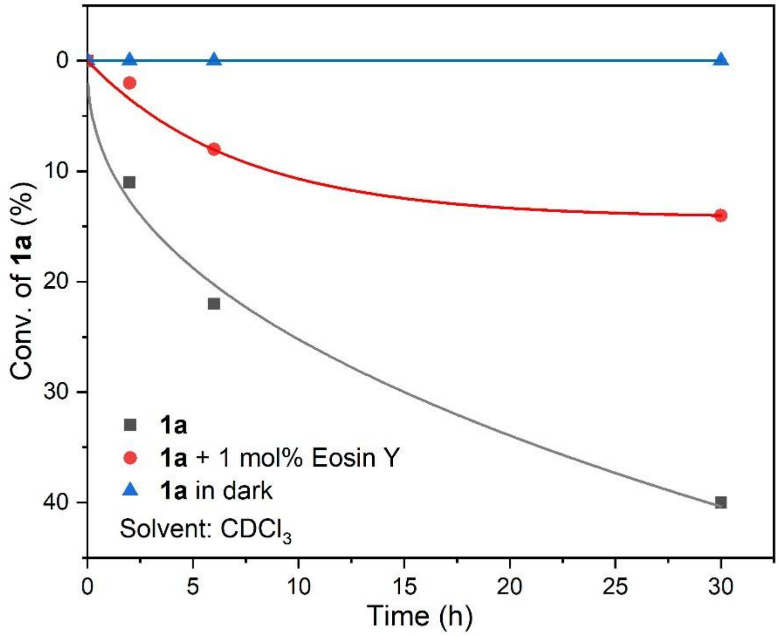

Supplementary Figure 5: Experiments on the light-induced decomposition of **1a**

In a glovebox, 0.5 mL $CDCl_3$ was added to the NMR tubes labeled 1, 2, and 3, followed by **1a** (0.05 mmol, 8.5 mg) and $BrCH_2CH_2Br$ (0.025 mmol, 4.7 mg). The No. 1 NMR tube is completely covered with tin foil. Eosin Y (0.0005 mmol, 0.32 mg) was added to the No. 2 NMR tube. The No. 3 NMR tube is only added by **1a** (0.05 mmol, 8.5 mg) and $BrCH_2CH_2Br$ (0.025 mmol, 4.7 mg). Illuminate the NMR tubes with a white LED lamp. The conversion of **1a** was measured at 2 h, 6 h and 30 h respectively. The conversion of **1a** were determined by ^{1}H NMR analysis of the unpurified mixture with $BrCH_2CH_2Br$ as an internal standard.

5.5 Luminescence Quenching Experiments

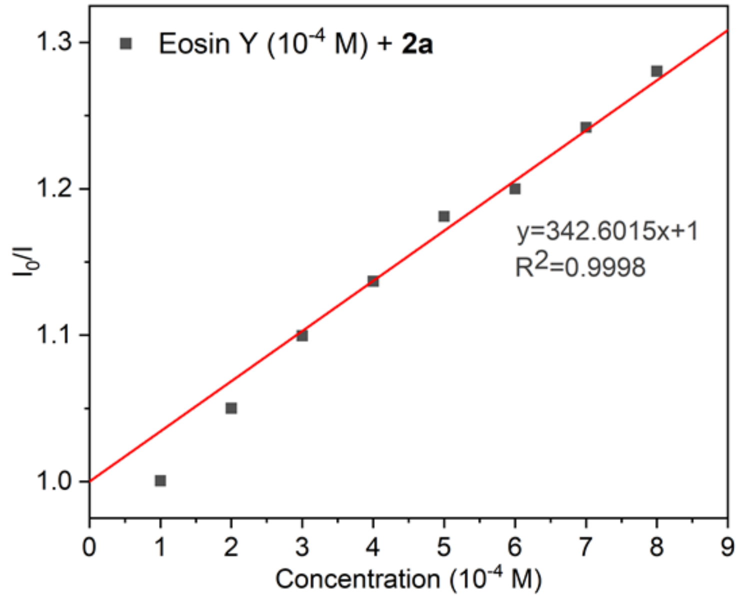

Supplementary Figure 6: Eosin Y emission quenching by **2a**

Fluorescence spectra was collected on Agilent Fluorescence Spectrophotometer G9800AS24 for all experiments. All Eosin Y solutions were excited at 350 nm and the emission intensity was collected at 550 nm. In a typical experiment, the emission spectrum of a 1×10^{-4} M solution of Eosin Y in MeOH/H_2O=1/2 (v/v) was collected. The significant decrease of Eosin Y luminescence could be observed in the presence of substrate **2a**.

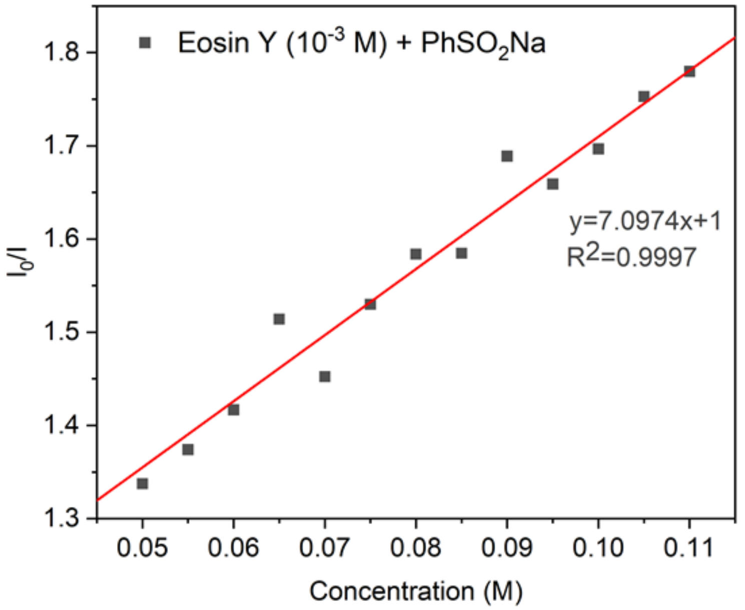

Supplementary Figure 7: Eosin Y emission quenching by $PhSO_2Na$

Fluorescence spectra was collected on Agilent Fluorescence Spectrophotometer G9800AS24 for all experiments. All Eosin Y solutions were excited at 350 nm and the emission intensity was collected at 550 nm. In a typical experiment, the emission spectrum of a 1×10^{-3} M solution of Eosin Y in MeCN/H_2O=1/2 (v/v) was collected. The significant decrease of Eosin Y luminescence could be observed in the presence of substrate $PhSO_2Na$.

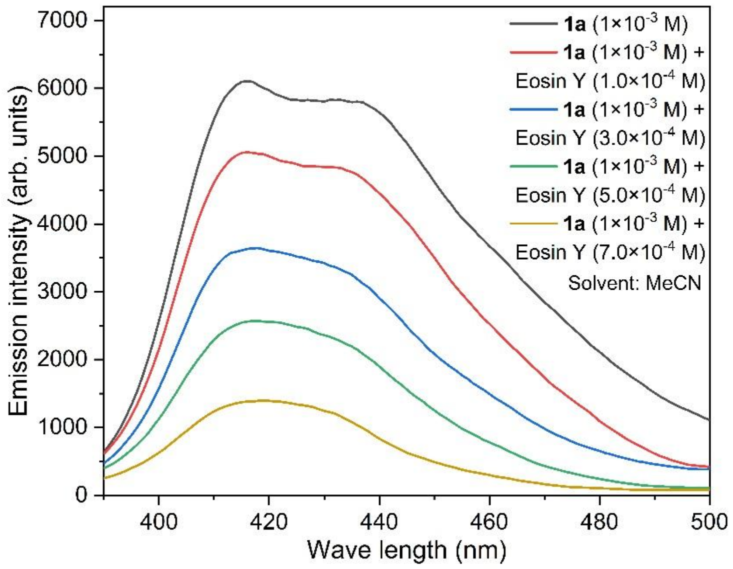

Supplementary Figure 8:**1a** emission quenching by Eosin Y

Fluorescence spectra was collected on Agilent Fluorescence Spectrophotometer G9800AS24 for all experiments. All **1a** solutions were excited at 350 nm and the emission intensity was collected at 416 nm. In a typical experiment, the emission spectrum of a 1×10^{-3} M solution of **1a** in MeCN was collected. The significant decrease of **1a** luminescence could be observed in the presence of substrate Eosin Y.

5.6 Cyclic voltammograms

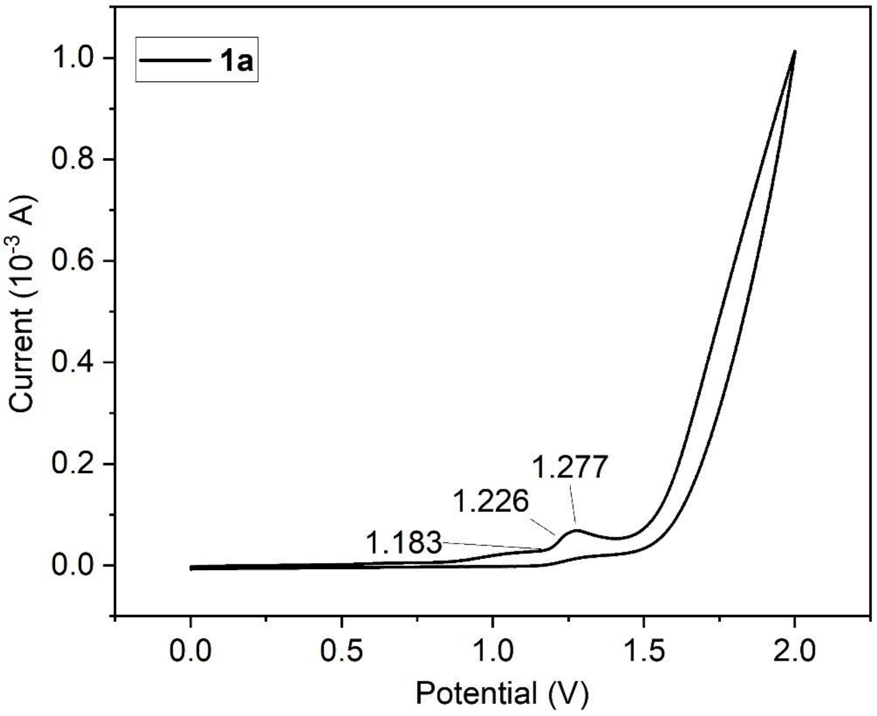

	SCE
E_p	**1.183 V**
$E_{p/2}$	**1.226 V**
E_s	**1.277 V**

Supplementary Figure 9: Cyclic voltammogram study for **1a** in MeCN

Cyclic voltammetry experiments were performed on a CH Instruments Electrochemical Analyzer (CHI660E model) at room temperature under nitrogen atmosphere. The 0.1 M MeCN solution of **1a** was prepared with 0.1 M tetrabutylammonium hexafluorophosphate as the supporting electrolyte, using a glassy carbon working electrode, a Pt counter electrode, and an SCE reference electrode. Scan Rate (V/s) = 0.10.

5.7 UV/Vis of 1a and Eosin Y.

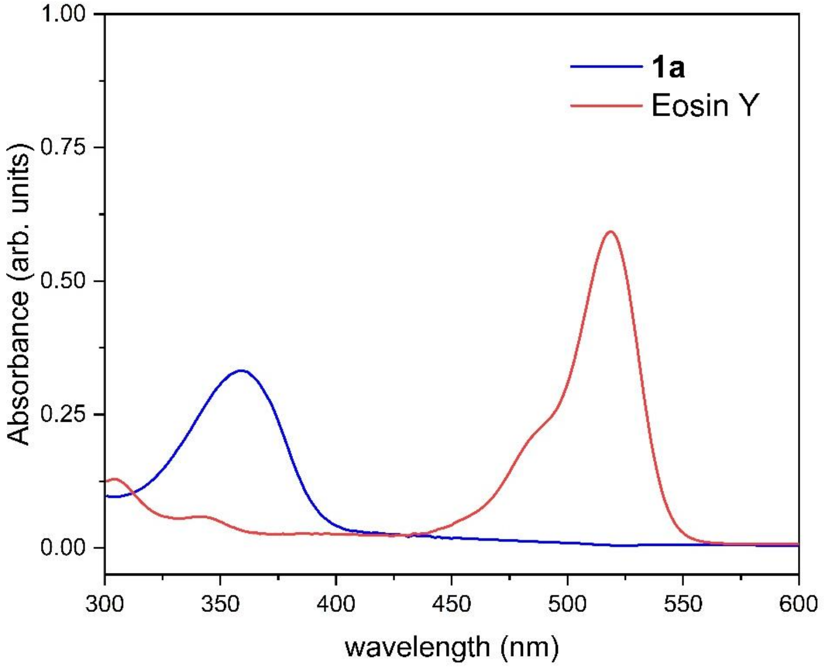

Supplementary Figure 10: Individual absorption Spectra of acylsilane 1a, EosinY (0.005 M in MeCN/H2O = 1/3).

The UV-Vis spectra of 1a and 2a in DCM were collected by using the following parameter set: scan rate 600 nm·min^{-1}, band width 2.0 nm, baseline correction.

5.8 Reaction in the presence of E-stilbene instead of Eosin Y.

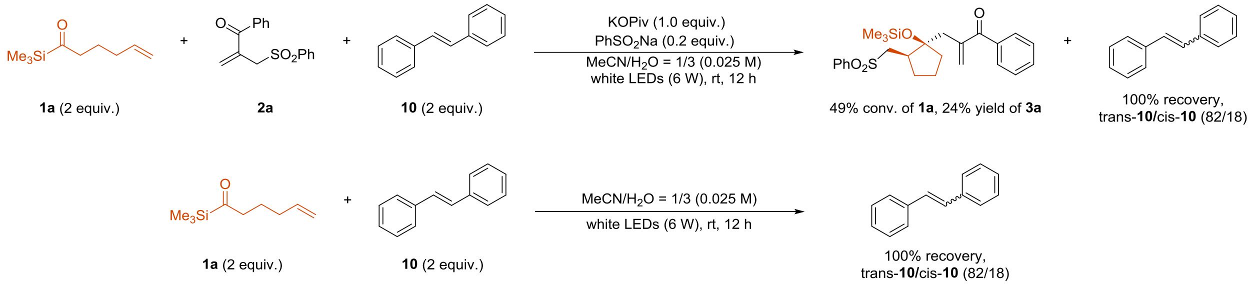

In a glovebox, to an oven-dried 10 mL tube was added **2a** (28.6 mg, 0.1 mmol), $PhSO_2Na$ (3.2 mg, 0.02 mmol, 0.2 equiv.), E-stilbene (18.0 mg, 0.1 mmol, 1 equiv.), KOPiv (14.2 mg, 0.1 mmol, 1 equiv.), MeCN (1 mL) and **1a** (34.0 mg, 0.2 mmol, 2 equiv.) and H_2O (3 mL) sequentially. The tube was sealed, then irradiated with 6 W white LED lamps. The mixture was stirred under white light irradiation at ambient temperature for the 12 h. Then the light was turned off. The resulting mixture was filtered through a thin silica gel plug with EA (30 mL) as the eluent. The organic phase was concentrated under reduced pressure. The conversion of **2a** and stereochemical scrambling/conversion to Z-stilbene were determined by ^1H NMR analysis of the unpurified mixture with $BrCH_2CH_2Br$ as an internal standard. In a glovebox, to an oven-dried 10 mL tube was added **2a** (28.6 mg, 0.1 mmol), E-stilbene (18.0 mg, 0.1 mmol, 1

equiv.), MeCN (1 mL) and **1a** (34.0 mg, 0.2 mmol, 2 equiv.) and H_2O (3 mL) sequentially. The tube was sealed, then irradiated with 6 W white LED lamps. The mixture was stirred under white light irradiation at ambient temperature for the 12 h. Then the light was turned off. The resulting mixture was filtered through a thin silica gel plug with EA (30 mL) as the eluent. The organic phase was concentrated under reduced pressure. The conversion of **2a** and stereochemical scrambling/conversion to Z-stilbene were determined by ^{1}H NMR analysis of the unpurified mixture with $BrCH_2CH_2Br$ as an internal standard.

5.9 Transient absorption spectra of 1a/Eosin Y/1a+ Eosin Y.

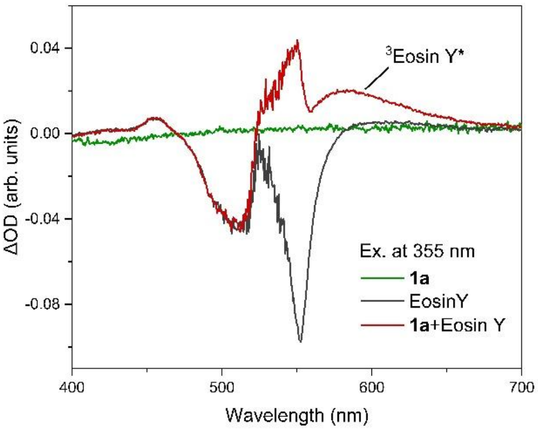

Supplementary Figure 11: Transient absorption spectra of **1a**/Eosin Y/**1a**+ Eosin Y.

The nanosecond transient absorption spectra were measured using Laser Flash Photolysis Spectrometer (LP980-KS, Edinburgh Instruments, UK). The samples were pumped by the third harmonic output of a frequency-doubled Q-switched Nd:YAG laser at 355 nm (LAB 190, Spectraphysics), with pulse energy at 10 mJ/pulse. The samples were probed by a pulsed Xe lamp. The transient absorption spectra were recorded by an iStar CCD camera (Andor Technology). The transient absorption kinetics were recorded by a R928 PMT (Hamamatsu Photonics) connected to a 500 MHz 5 GS/s oscilloscope (Tektronix TDS 3052). All samples were carried out at room temperature, in a standard 1.0 cm path length quartz cell cuvette with screwed cap and degassed with Ar for 30 min before the measurement.

Transient absorption spectra observed after laser excitation (λex = 355 nm) of a) **1a** (1.0×10^{-3} M); b) Eosin Y (1.0×10^{-4} M); c) **1a** (1×10^{-3} M)+Eosin Y (1.0×10^{-4} M)

5.10 Proposed mechanism of the synthesis of γ-substituted cyclopentanol derivatives.

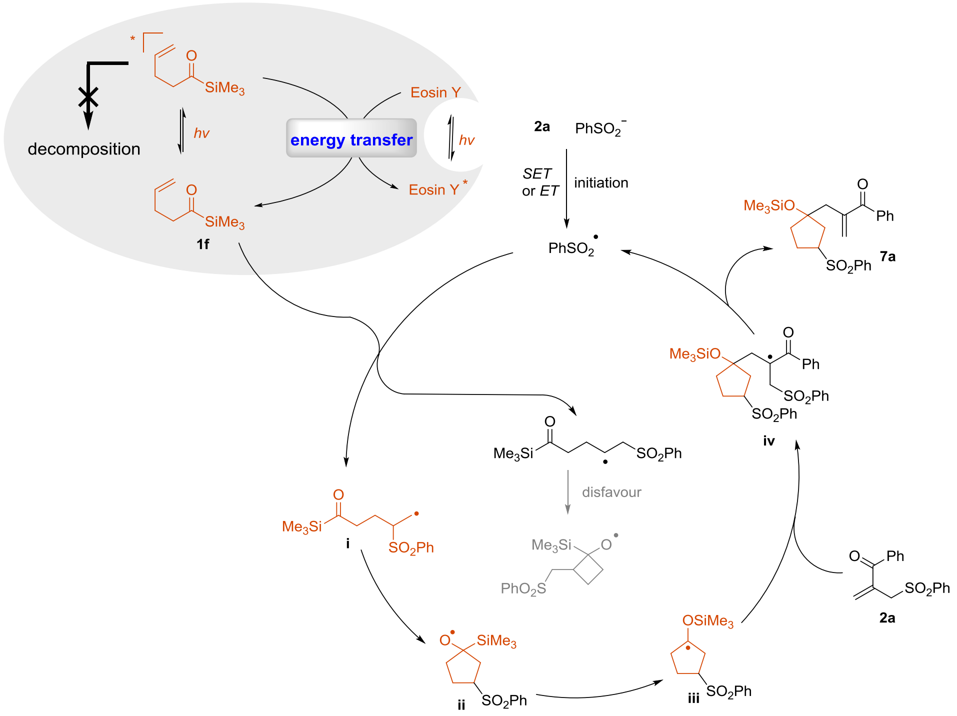

Supplementary Figure 12: Proposed mechanism of the synthesis of γ-substituted cyclopentanol derivatives.

5.10 NOE of 7a

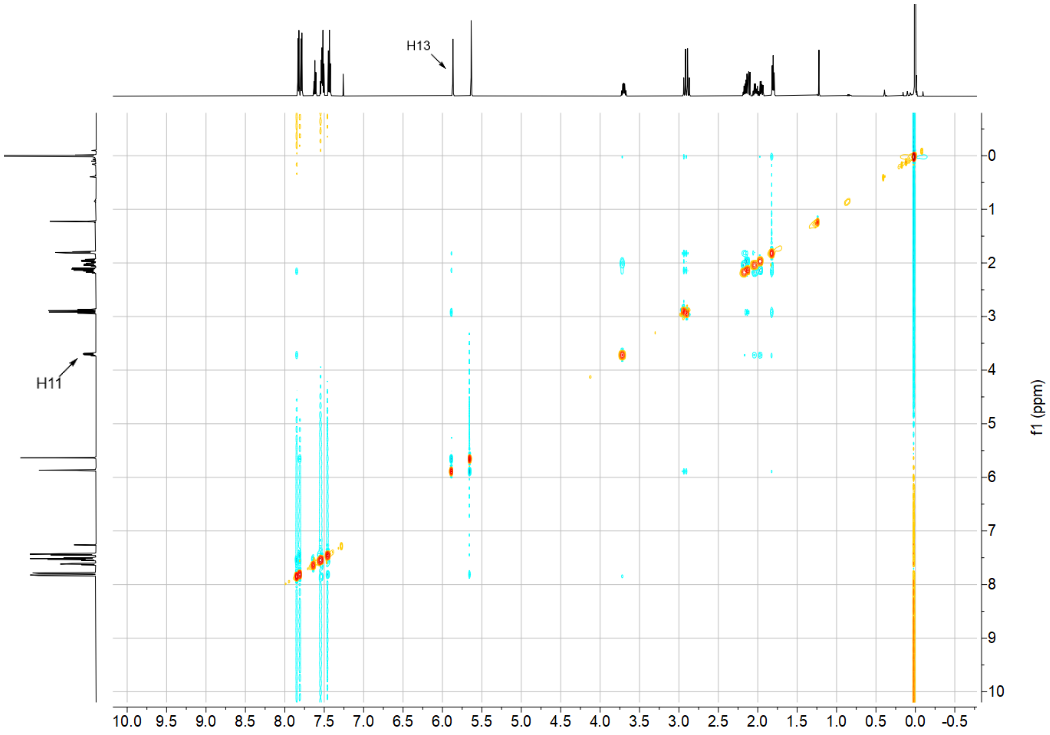

Supplementary Figure 13: NOE spectra of **7a**

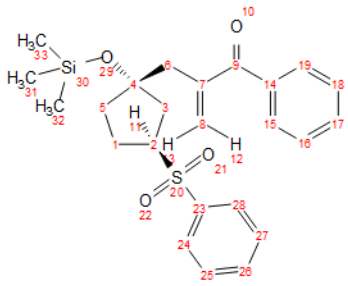

H11 is not correlated with H13, suggesting that the two H atomsare not on the same plane, indicating that the configuration of the product is trans.

5.11 NOE of 7a’

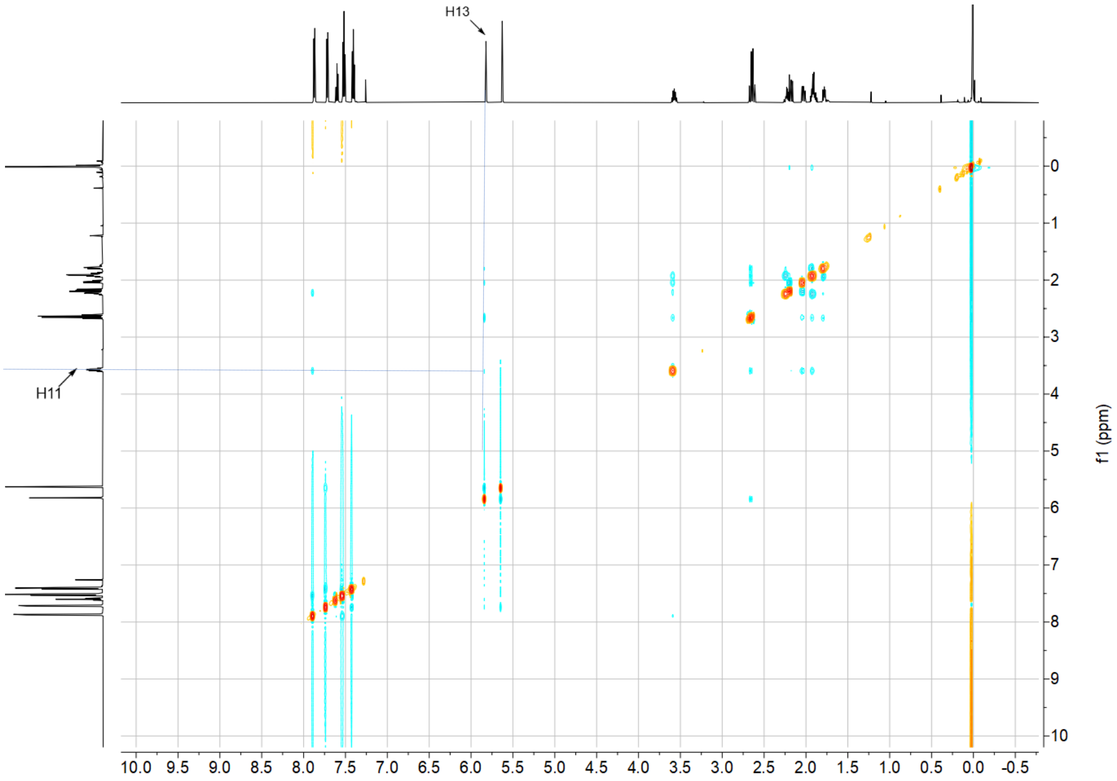

Supplementary Figure 14: NOE spectra of **7a’**

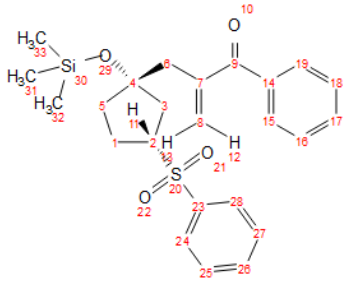

H11 is correlated with H13, suggesting that these H atoms are located on the same plane, indicating that the product is a cis-structure.

5.12 COSY of 8.

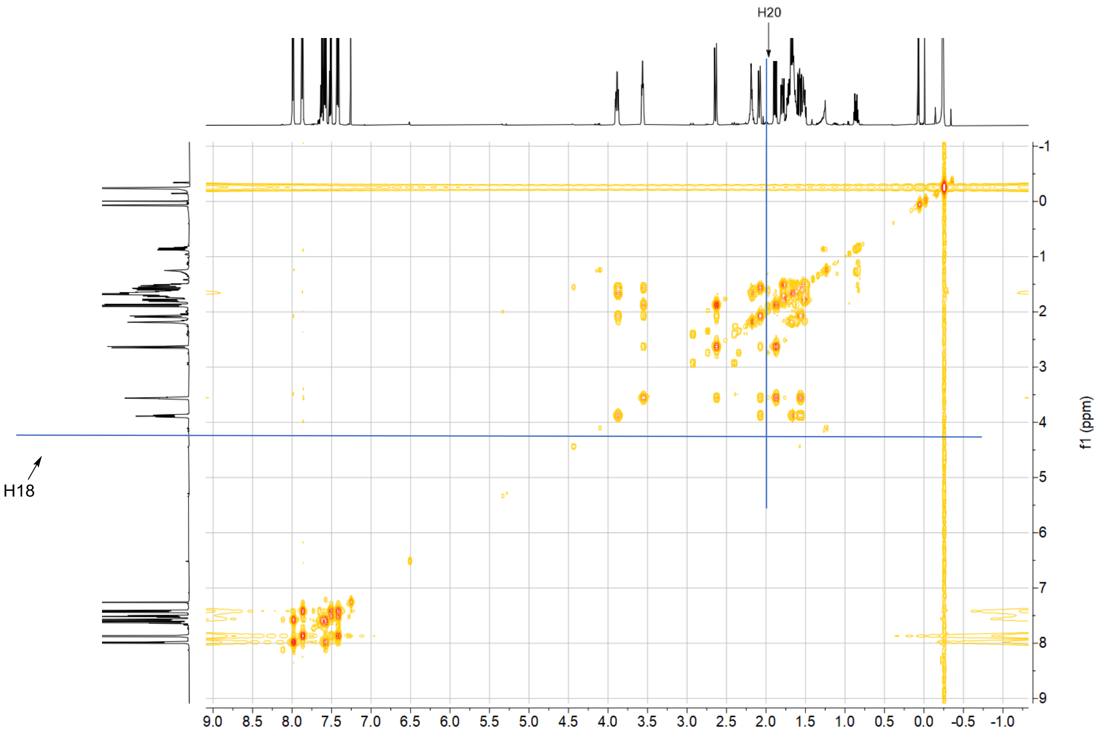

Supplementary Figure 15: COSY spectra of **8**

5.13 NOE of 8.

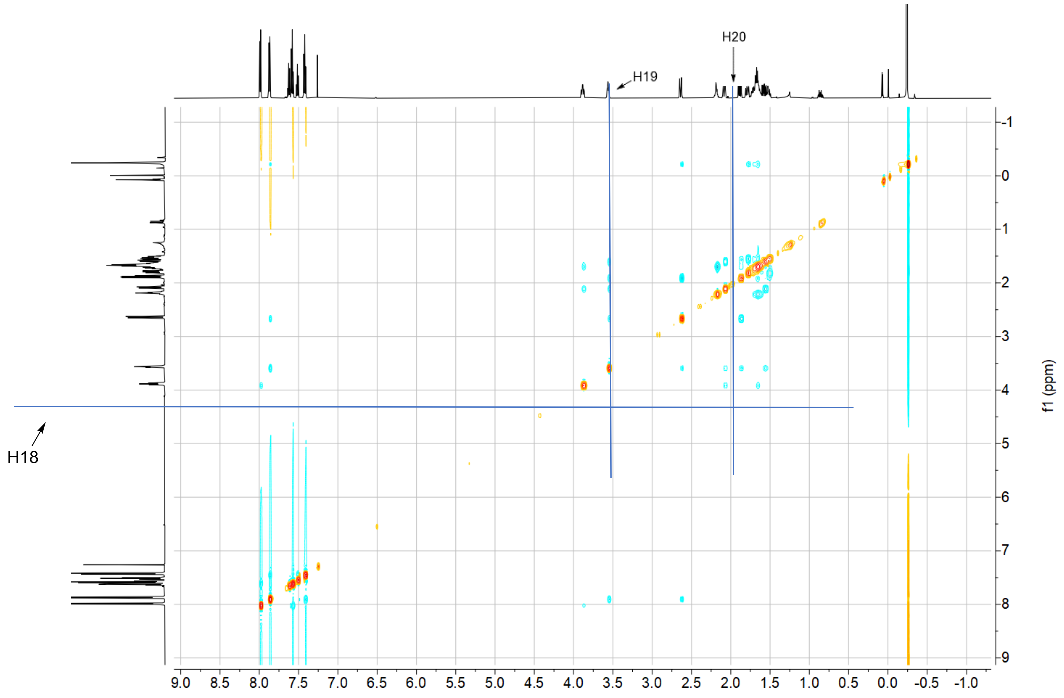

Supplementary Figure 16: NOE spectra of **8**

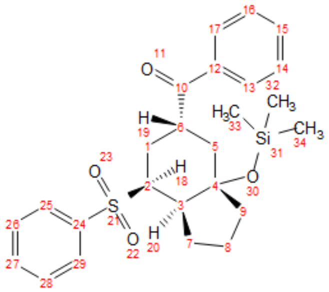

According to the COSY: H18 is correlated with H20.

According to the NOE: H18 is not correlated with H19, suggesting that these H atoms are located on the different plane. H18 is correlated with H20, suggesting that these two H atoms are located on the same plane.

5.14 ORTEP diagram of compound 3j.

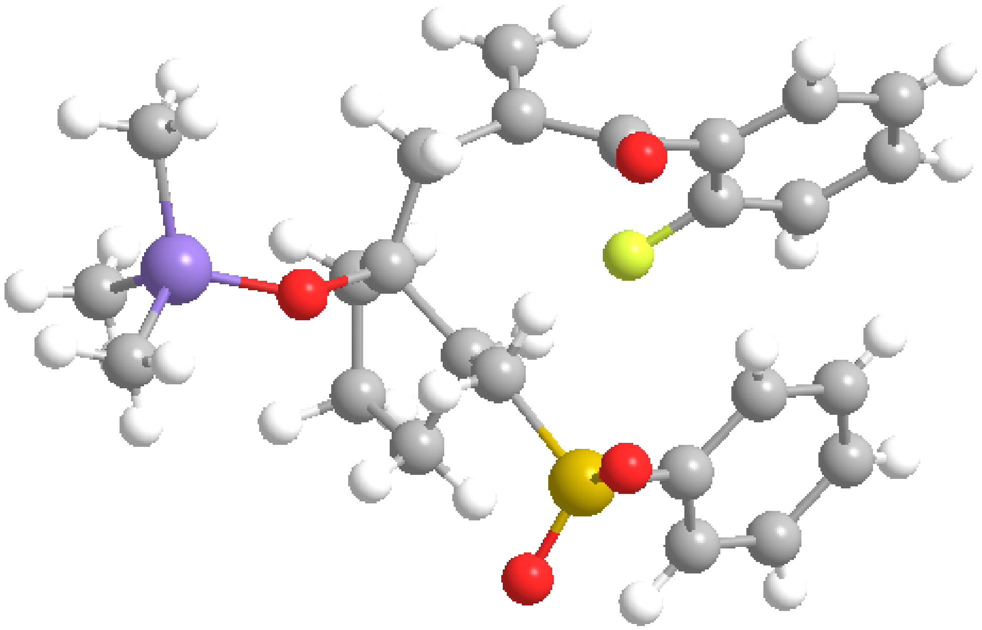

Supplementary Figure 17: ORTEP diagram of compound 3j. (CCDC 2076884)

Table 1. Crystal data and structure refinement for final-zyx-9-147-250k-20210113.

Identification code	zyx-9-147-250k-20210113	
Empirical formula	C25 H31 F O4 S Si	
Formula weight	474.65	
Temperature	249.98(10) K	
Wavelength	1.54184 Å	
Crystal system	Triclinic	
Space group	P-1	
Unit cell dimensions	a = 9.46640(10) Å	α= 78.518(2) °.
	b = 11.3418(2) Å	β= 68.875(2) °.
	c = 12.7977(2) Å	γ = 78.0470(10) °.

Volume	1242.20(4) Å^3
Z	2
Density (calculated)	1.269 Mg/m^3
Absorption coefficient	1.919 mm^{-1}
F(000)	504
Crystal size	0.08 x 0.06 x 0.04 mm^3
Theta range for data collection	3.738 to 75.298 °.
Index ranges	-11<=h<=11, -14<=k<=14, -15<=l<=15
Reflections collected	44512
Independent reflections	4930 [R(int) = 0.0468]
Completeness to theta = 67.684 °	98.3 %
Absorption correction	Semi-empirical from equivalents
Max. and min. transmission	1.00000 and 0.84796
Refinement method	Full-matrix least-squares on F^2
Data / restraints / parameters	4930 / 0 / 413
Goodness-of-fit on F^2	1.053
Final R indices [I>2sigma(I)]	R1 = 0.0441, wR2 = 0.1180
R indices (all data)	R1 = 0.0454, wR2 = 0.1189
Extinction coefficient	n/a
Largest diff. peak and hole	0.389 and -0.604 e.Å^{-3}

Table 2. Atomic coordinates (x 10^4) and equivalent isotropic displacement parameters (Å^{2}x 10^3) for final-zyx-9-147-250k-20210113. U(eq) is defined as one third of the trace of the orthogonalized U^{ij} tensor.

	x	y	z	U(eq)
S(1)	6001(1)	8203(1)	7538(1)	49(1)
Si(1)	-306(1)	10674(1)	7987(1)	43(1)
O(2)	1145(1)	9560(1)	7860(1)	45(1)
F(1)	3334(2)	5249(1)	6449(1)	69(1)
O(1)	2887(2)	5723(1)	9480(1)	58(1)
O(3)	6505(2)	9155(1)	6627(1)	65(1)
O(4)	6435(2)	8108(2)	8520(2)	72(1)
C(16)	3970(2)	8340(2)	7995(2)	44(1)
C(2)	3286(2)	8079(2)	7173(1)	40(1)
C(1)	1520(2)	8333(1)	7604(1)	40(1)
C(10)	3172(2)	4285(2)	8293(2)	47(1)

C(9)	2412(2)	5430(2)	8816(2)	46(1)
C(6)	710(2)	7506(2)	8672(2)	47(1)
C(5)	1152(2)	8236(2)	6560(2)	49(1)
C(7)	1048(2)	6163(2)	8562(2)	46(1)
C(17)	6703(2)	6804(2)	6984(2)	52(1)
C(11)	3602(2)	4228(2)	7151(2)	51(1)
C(15)	3552(2)	3231(2)	8964(2)	57(1)
C(3)	3702(2)	8829(2)	5997(2)	53(1)
C(13)	4699(2)	2151(2)	7350(2)	67(1)
C(14)	4294(2)	2172(2)	8502(2)	68(1)
C(24)	-1947(3)	10321(2)	9274(2)	67(1)
C(22)	6543(2)	5720(2)	7705(2)	62(1)
C(12)	4364(2)	3191(2)	6663(2)	62(1)
C(8)	111(2)	5595(2)	8351(2)	64(1)
C(18)	7425(3)	6818(2)	5828(2)	68(1)
C(25)	457(3)	11999(2)	8115(3)	68(1)
C(4)	2414(3)	8769(3)	5555(2)	67(1)
C(23)	-985(3)	11063(2)	6756(2)	66(1)
C(21)	7111(3)	4631(2)	7244(3)	83(1)
C(20)	7817(3)	4647(3)	6098(4)	94(1)
C(19)	7984(3)	5725(3)	5381(3)	88(1)

Table 3. Bond lengths [Å] and angles [°] for final-zyx-9-147-250k-20210113.

S(1)-O(3)	1.4360(14)	C(13)-C(14)	1.387(4)
S(1)-O(4)	1.4352(15)	C(13)-C(12)	1.378(3)
S(1)-C(16)	1.7793(18)	C(13)-H(13)	0.97(3)
S(1)-C(17)	1.769(2)	C(14)-H(14)	0.88(3)
Si(1)-O(2)	1.6470(12)	C(24)-H(24A)	0.99(3)
Si(1)-C(24)	1.854(2)	C(24)-H(24B)	1.01(3)
Si(1)-C(25)	1.854(2)	C(24)-H(24C)	1.03(4)
Si(1)-C(23)	1.851(2)	C(22)-C(21)	1.396(4)
O(2)-C(1)	1.4361(19)	C(22)-H(22)	0.91(2)
F(1)-C(11)	1.357(2)	C(12)-H(12)	0.94(3)
O(1)-C(9)	1.218(2)	C(8)-H(8A)	0.95(3)
C(16)-C(2)	1.519(2)	C(8)-H(8B)	0.94(3)
C(16)-H(16A)	0.99(2)	C(18)-C(19)	1.391(4)
C(16)-H(16B)	0.92(2)	C(18)-H(18)	0.99(3)
C(2)-C(1)	1.543(2)	C(25)-H(25A)	0.96(3)
C(2)-C(3)	1.532(2)	C(25)-H(25B)	0.91(3)
C(2)-H(2)	0.974(19)	C(25)-H(25C)	1.00(3)
C(1)-C(6)	1.539(2)	C(4)-H(4A)	0.96(3)
C(1)-C(5)	1.526(2)	C(4)-H(4B)	1.05(3)
C(10)-C(9)	1.504(2)	C(23)-H(23A)	0.94(3)
C(10)-C(11)	1.380(3)	C(23)-H(23B)	0.96(3)
C(10)-C(15)	1.390(3)	C(23)-H(23C)	0.97(3)
C(9)-C(7)	1.489(2)	C(21)-C(20)	1.373(5)
C(6)-C(7)	1.514(2)	C(21)-H(21)	0.87(3)
C(6)-H(6A)	0.98(2)	C(20)-C(19)	1.375(5)
C(6)-H(6B)	1.04(3)	C(20)-H(20)	0.93(3)
C(5)-C(4)	1.526(3)	C(19)-H(19)	0.98(3)
C(5)-H(5A)	0.98(2)		
C(5)-H(5B)	0.98(2)	O(3)-S(1)-C(16)	109.38(9)
C(7)-C(8)	1.324(3)	O(3)-S(1)-C(17)	107.48(10)
C(17)-C(22)	1.382(3)	O(4)-S(1)-O(3)	117.96(10)
C(17)-C(18)	1.387(3)	O(4)-S(1)-C(16)	107.60(9)
C(11)-C(12)	1.382(3)	O(4)-S(1)-C(17)	107.92(10)
C(15)-C(14)	1.385(3)	C(17)-S(1)-C(16)	105.86(8)
C(15)-H(15)	0.94(2)	O(2)-Si(1)-C(24)	111.02(9)
C(3)-C(4)	1.536(3)	O(2)-Si(1)-C(25)	104.16(9)
C(3)-H(3A)	1.01(2)	O(2)-Si(1)-C(23)	114.24(10)
C(3)-H(3B)	0.95(3)	C(25)-Si(1)-C(24)	110.11(13)

C(23)-Si(1)-C(24)	108.54(13)	H(5A)-C(5)-H(5B)	109.4(18)
C(23)-Si(1)-C(25)	108.65(13)	C(9)-C(7)-C(6)	118.28(16)
C(1)-O(2)-Si(1)	137.92(10)	C(8)-C(7)-C(9)	118.61(17)
S(1)-C(16)-H(16A)	104.2(12)	C(8)-C(7)-C(6)	122.82(18)
S(1)-C(16)-H(16B)	102.2(14)	C(22)-C(17)-S(1)	119.90(18)
C(2)-C(16)-S(1)	116.50(12)	C(22)-C(17)-C(18)	121.0(2)
C(2)-C(16)-H(16A)	112.1(12)	C(18)-C(17)-S(1)	119.07(16)
C(2)-C(16)-H(16B)	110.8(14)	F(1)-C(11)-C(10)	119.16(16)
H(16A)-C(16)-H(16B)	110.5(18)	F(1)-C(11)-C(12)	117.21(19)
C(16)-C(2)-C(1)	112.40(13)	C(10)-C(11)-C(12)	123.56(19)
C(16)-C(2)-C(3)	116.87(15)	C(10)-C(15)-H(15)	118.0(14)
C(16)-C(2)-H(2)	108.3(11)	C(14)-C(15)-C(10)	121.1(2)
C(1)-C(2)-H(2)	109.1(11)	C(14)-C(15)-H(15)	120.9(14)
C(3)-C(2)-C(1)	102.77(13)	C(2)-C(3)-C(4)	104.77(16)
C(3)-C(2)-H(2)	107.1(11)	C(2)-C(3)-H(3A)	108.1(13)
O(2)-C(1)-C(2)	105.07(13)	C(2)-C(3)-H(3B)	107.6(16)
O(2)-C(1)-C(6)	108.05(13)	C(4)-C(3)-H(3A)	115.5(13)
O(2)-C(1)-C(5)	111.58(13)	C(4)-C(3)-H(3B)	111.8(17)
C(6)-C(1)-C(2)	116.08(14)	H(3A)-C(3)-H(3B)	109(2)
C(5)-C(1)-C(2)	101.73(13)	C(14)-C(13)-H(13)	121.2(15)
C(5)-C(1)-C(6)	113.97(15)	C(12)-C(13)-C(14)	119.7(2)
C(11)-C(10)-C(9)	123.52(16)	C(12)-C(13)-H(13)	119.2(15)
C(11)-C(10)-C(15)	116.71(18)	C(15)-C(14)-C(13)	120.4(2)
C(15)-C(10)-C(9)	119.67(18)	C(15)-C(14)-H(14)	121.8(17)
O(1)-C(9)-C(10)	119.45(16)	C(13)-C(14)-H(14)	117.7(16)
O(1)-C(9)-C(7)	120.52(17)	Si(1)-C(24)-H(24A)	109.2(19)
C(7)-C(9)-C(10)	119.97(16)	Si(1)-C(24)-H(24B)	111.4(17)
C(1)-C(6)-H(6A)	109.6(12)	Si(1)-C(24)-H(24C)	110.3(18)
C(1)-C(6)-H(6B)	106.2(13)	H(24A)-C(24)-H(24B)	110(2)
C(7)-C(6)-C(1)	114.87(14)	H(24A)-C(24)-H(24C)	109(3)
C(7)-C(6)-H(6A)	109.4(12)	H(24B)-C(24)-H(24C)	107(3)
C(7)-C(6)-H(6B)	108.6(13)	C(17)-C(22)-C(21)	118.6(3)
H(6A)-C(6)-H(6B)	107.9(17)	C(17)-C(22)-H(22)	119.3(13)
C(1)-C(5)-H(5A)	105.7(12)	C(21)-C(22)-H(22)	122.0(13)
C(1)-C(5)-H(5B)	111.3(12)	C(11)-C(12)-H(12)	119.3(17)
C(4)-C(5)-C(1)	105.40(16)	C(13)-C(12)-C(11)	118.6(2)
C(4)-C(5)-H(5A)	108.8(12)	C(13)-C(12)-H(12)	122.1(17)
C(4)-C(5)-H(5B)	115.8(13)	C(7)-C(8)-H(8A)	121.0(15)

C(7)-C(8)-H(8B)	121.7(15)
H(8A)-C(8)-H(8B)	117(2)
C(17)-C(18)-C(19)	119.7(3)
C(17)-C(18)-H(18)	121.6(16)
C(19)-C(18)-H(18)	118.7(16)
Si(1)-C(25)-H(25A)	114.5(17)
Si(1)-C(25)-H(25B)	103.0(17)
Si(1)-C(25)-H(25C)	110.9(16)
H(25A)-C(25)-H(25B)	104(2)
H(25A)-C(25)-H(25C)	110(2)
H(25B)-C(25)-H(25C)	114(2)
C(5)-C(4)-C(3)	106.57(16)
C(5)-C(4)-H(4A)	110.0(17)
C(5)-C(4)-H(4B)	109.2(18)
C(3)-C(4)-H(4A)	108.6(17)
C(3)-C(4)-H(4B)	110.5(18)
H(4A)-C(4)-H(4B)	112(2)
Si(1)-C(23)-H(23A)	106.3(17)
Si(1)-C(23)-H(23B)	115.5(18)
Si(1)-C(23)-H(23C)	107.8(19)
H(23A)-C(23)-H(23B)	101(2)
H(23A)-C(23)-H(23C)	112(3)
H(23B)-C(23)-H(23C)	114(3)
C(22)-C(21)-H(21)	117.8(19)
C(20)-C(21)-C(22)	120.3(3)
C(20)-C(21)-H(21)	121.8(18)
C(21)-C(20)-C(19)	121.2(3)
C(21)-C(20)-H(20)	122.2(19)
C(19)-C(20)-H(20)	116.6(19)
C(18)-C(19)-H(19)	124.6(19)
C(20)-C(19)-C(18)	119.2(3)
C(20)-C(19)-H(19)	116.1(18)

Symmetry transformations used to generate equivalent atoms: :

Table 4. Anisotropic displacement parameters ($Å^2$x 10^3) for final-zyx-9-147-250k-20210113. The anisotropic

displacement factor exponent takes the form: $-2\pi^2[h^2 a^{*2}U^{11} + ... + 2 h k a^* b^* U^{12}]$

	U^{11}	U^{22}	U^{33}	U^{23}	U^{13}	U^{12}
S(1)	46(1)	43(1)	65(1)	1(1)	-27(1)	-13(1)
Si(1)	41(1)	38(1)	55(1)	-5(1)	-22(1)	-3(1)
O(2)	44(1)	39(1)	53(1)	-9(1)	-20(1)	0(1)
F(1)	95(1)	53(1)	56(1)	-2(1)	-30(1)	1(1)
O(1)	64(1)	58(1)	58(1)	-2(1)	-29(1)	-10(1)
O(3)	58(1)	50(1)	83(1)	9(1)	-20(1)	-22(1)
O(4)	74(1)	79(1)	86(1)	-5(1)	-52(1)	-20(1)
C(16)	46(1)	43(1)	44(1)	-3(1)	-18(1)	-8(1)
C(2)	37(1)	41(1)	39(1)	-5(1)	-13(1)	-4(1)
C(1)	40(1)	36(1)	44(1)	-6(1)	-14(1)	-2(1)
C(10)	38(1)	43(1)	57(1)	1(1)	-17(1)	-8(1)
C(9)	42(1)	45(1)	46(1)	5(1)	-12(1)	-12(1)
C(6)	41(1)	44(1)	50(1)	-4(1)	-9(1)	-4(1)
C(5)	52(1)	49(1)	53(1)	-8(1)	-27(1)	-3(1)
C(7)	39(1)	43(1)	52(1)	-1(1)	-11(1)	-7(1)
C(17)	36(1)	48(1)	76(1)	-1(1)	-26(1)	-6(1)
C(11)	50(1)	44(1)	61(1)	-5(1)	-21(1)	-4(1)
C(15)	45(1)	53(1)	67(1)	7(1)	-20(1)	-8(1)
C(3)	52(1)	60(1)	42(1)	4(1)	-14(1)	-10(1)
C(13)	45(1)	48(1)	99(2)	-12(1)	-15(1)	-2(1)
C(14)	48(1)	44(1)	103(2)	15(1)	-28(1)	-6(1)
C(24)	51(1)	67(1)	72(1)	-14(1)	-10(1)	-1(1)
C(22)	44(1)	49(1)	94(2)	4(1)	-29(1)	-8(1)
C(12)	56(1)	55(1)	74(1)	-16(1)	-17(1)	-5(1)
C(8)	48(1)	50(1)	98(2)	-2(1)	-32(1)	-10(1)
C(18)	52(1)	65(1)	81(2)	-11(1)	-21(1)	4(1)
C(25)	66(1)	48(1)	101(2)	-12(1)	-41(1)	-8(1)
C(4)	62(1)	96(2)	42(1)	-2(1)	-22(1)	-9(1)
C(23)	74(2)	56(1)	78(2)	-4(1)	-46(1)	2(1)

C(21)	52(1)	48(1)	145(3)	3(1)	-37(2)	-7(1)
C(20)	58(1)	69(2)	152(3)	-37(2)	-29(2)	8(1)
C(19)	64(2)	92(2)	100(2)	-32(2)	-22(1)	12(1)

Table 5. Hydrogen coordinates (x 10^4) and isotropic displacement parameters (Å^{2}x 10 ^{3}) for final-zyx-9-147-250k-20210113.

	x	y	z	U(eq)
H(6A)	970(20)	7633(18)	9313(17)	47(5)
H(2)	3620(20)	7226(18)	7066(15)	43(5)
H(5A)	1260(20)	7360(20)	6535(17)	52(5)
H(16A)	3700(20)	7782(19)	8720(18)	54(5)
H(15)	3290(30)	3260(20)	9744(19)	59(6)
H(5B)	100(30)	8620(20)	6619(18)	58(6)
H(16B)	3670(30)	9140(20)	8126(18)	60(6)
H(6B)	-460(30)	7790(20)	8840(20)	70(7)
H(14)	4480(30)	1480(20)	8930(20)	72(7)
H(13)	5230(30)	1420(20)	7010(20)	73(7)
H(3A)	4770(30)	8470(20)	5533(19)	65(6)
H(12)	4650(30)	3220(30)	5870(20)	85(8)
H(3B)	3720(30)	9640(30)	6090(20)	80(8)
H(23A)	-100(30)	11110(20)	6110(20)	83(8)
H(24A)	-1590(40)	10100(30)	9940(30)	104(10)
H(23B)	-1400(30)	10430(30)	6610(20)	91(9)
H(23C)	-1660(40)	11840(30)	6830(30)	108(10)
H(24B)	-2790(40)	11030(30)	9400(20)	99(9)
H(24C)	-2400(40)	9610(30)	9190(30)	110(10)
H(22)	6130(20)	5736(18)	8468(18)	47(6)
H(25A)	990(30)	11840(30)	8650(20)	90(9)
H(18)	7560(30)	7590(30)	5310(20)	85(8)
H(25B)	1210(30)	12120(20)	7440(20)	75(8)
H(21)	6970(30)	3950(30)	7700(20)	80(8)
H(4A)	2810(30)	8240(30)	4980(20)	86(8)
H(20)	8190(40)	3940(30)	5760(30)	99(10)
H(25C)	-370(30)	12710(30)	8290(20)	84(8)

H(4B)	1980(40)	9650(30)	5250(30)	106(10)
H(19)	8540(40)	5660(30)	4580(30)	95(9)
H(8A)	-820(30)	6020(20)	8250(20)	72(7)
H(8B)	330(30)	4750(20)	8300(20)	70(7)

6. Computational Details

Computational methods

All density functional theory (DFT) calculations were carried out using the Gaussian 16 software package.[5] All geometries were optimized using the M06-2X functional[6] with a basis set of 6-31G(d) for all atoms. Frequencies were calculated for all the stationary points to confirm if each optimized structure is a local minimum on the respective potential energy surface or a transition state structure with only one imaginary frequency. Solvation energy correction was calculated in water solvent with the SMD continuum solvation model[7] based on the gas phase optimized geometries. The M06-2X functional with a basis set of 6-311+G(d,p) for all atoms was used for single point energy calculations. The 3D structures were plot using CYLview.[8]

DFT study of 1f involved Cascade Cyclization of Alkene-tethered Acylsilanes and Allyic Sulfones

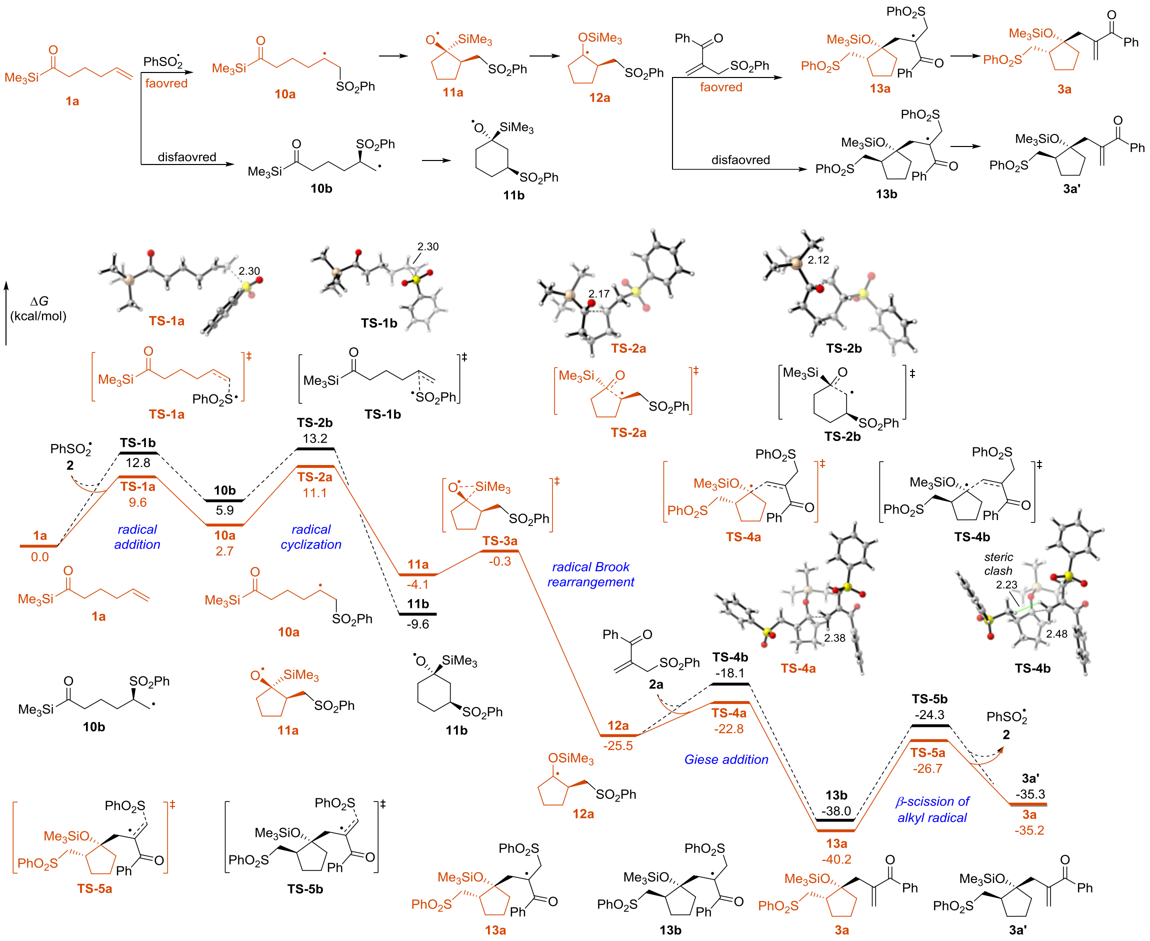

Supplementary Figure 18: DFT study of **1f** involved Cascade Cyclization of Alkene-tethered Acylsilanes and Allyic Sulfones

Cartesian coordinates (Å) and energies of optimized structures

Supplementary Table 7: Cartesian coordinates of **1a**

M062X SCF energy: -718.25357354 a.u.

M062X enthalpy: -717.984996 a.u.

M062X free energy: -718.048885 a.u.

M062X SCF energy in solution: -718.42153480 a.u.

M062X enthalpy in solution: -718.152957 a.u.

M062X free energy in solution: -718.216846 a.u.

Cartesian coordinates

ATOM	X	Y	Z
C	-0.590713	-0.542117	-0.053078
O	-0.440958	-1.749113	0.021584
Si	-2.395180	0.150987	0.036134
C	-2.888733	0.095453	1.851290
H	-3.927356	0.415682	1.984629
H	-2.795887	-0.923690	2.239078
H	-2.256093	0.748160	2.461467
C	-3.462294	-1.009553	-0.980843
H	-4.528128	-0.815752	-0.824538
H	-3.253241	-0.912499	-2.050625
H	-3.252432	-2.043406	-0.690486
C	-2.449131	1.918296	-0.613804
H	-3.464191	2.324426	-0.550235
H	-1.792987	2.577055	-0.035077
H	-2.137643	1.970145	-1.662254
C	0.609561	0.384968	-0.171898
H	0.484893	0.975368	-1.093047
H	0.546454	1.120802	0.645493
C	1.948310	-0.340443	-0.157819
H	1.962317	-1.080029	-0.966789
H	2.043195	-0.912259	0.771442
C	3.135305	0.618062	-0.300456
H	3.020472	1.189788	-1.232552
H	3.128973	1.346729	0.520065
C	4.449051	-0.108583	-0.315484
H	4.580110	-0.837951	-1.115576
C	5.420697	0.061078	0.575988
H	5.322229	0.775517	1.389844
H	6.347171	-0.501976	0.527239

Supplementary Table 8: Cartesian coordinates of **2**

M062X SCF energy: -779.98659512 a.u.
M062X enthalpy: -779.877940 a.u.
M062X free energy: -779.919945 a.u.
M062X SCF energy in solution: -780.14285358 a.u.
M062X enthalpy in solution: -780.034198 a.u.
M062X free energy in solution: -780.076203 a.u.

Cartesian coordinates

ATOM	X	Y	Z
C	0.757061	-1.220539	-0.054153
C	0.091394	0.000004	-0.084172
C	0.757140	1.220601	-0.054318
C	2.145977	1.210668	0.032037
C	2.835818	-0.000048	0.073159
C	2.145989	-1.210680	0.032182
H	0.193521	-2.147138	-0.077284
H	0.193391	2.147072	-0.077576
H	2.689093	2.149063	0.070296
H	3.919070	0.000029	0.138405
H	2.688914	-2.149180	0.070543
S	-1.696862	0.000023	-0.253260
O	-2.183423	-1.289617	0.266034
O	-2.183385	1.289586	0.266386

Supplementary Table 9: Cartesian coordinates of **TS-1a**

M062X SCF energy: -1498.23957830 a.u.
M062X enthalpy: -1497.861593 a.u.
M062X free energy: -1497.946251 a.u.
M062X SCF energy in solution: -1498.57100884 a.u.
M062X enthalpy in solution: -1498.193024 a.u.
M062X free energy in solution: -1498.277682 a.u.
Imaginary frequency: -361.7383 cm-1

Cartesian coordinates

ATOM	X	Y	Z
C	3.445214	-0.773429	0.372141
O	3.616108	-1.788454	1.023831
Si	5.000784	0.220143	-0.209536

C	5.581328	-0.594293	-1.804182
H	6.492675	-0.114827	-2.176565
H	5.801177	-1.653173	-1.636697
H	4.822870	-0.526191	-2.590861
C	6.285176	0.044411	1.144653
H	7.264767	0.408212	0.819318
H	5.997120	0.600286	2.042166
H	6.380765	-1.009845	1.421082
C	4.530776	2.017496	-0.522757
H	5.395748	2.588701	-0.875931
H	3.748550	2.097809	-1.285190
H	4.164629	2.501297	0.388706
C	2.041038	-0.326820	-0.011275
H	1.886786	0.683685	0.397755
H	2.015615	-0.198190	-1.104897
C	0.951488	-1.282083	0.456775
H	1.028376	-1.408236	1.542653
H	1.126516	-2.273676	0.026202
C	-0.452181	-0.787203	0.083986
H	-0.602218	0.214872	0.517110
H	-0.535015	-0.670152	-1.004408
C	-1.521963	-1.709343	0.574240
H	-1.553862	-1.901984	1.645292
C	-2.494922	-2.259004	-0.228838
H	-2.371749	-2.230908	-1.310734
H	-3.122431	-3.059618	0.155785
C	-3.229302	1.157425	1.290393
C	-3.446760	0.752339	-0.022689
C	-2.918828	1.442383	-1.108788
C	-2.155407	2.581267	-0.867611
C	-1.924909	3.003008	0.441520
C	-2.463793	2.298155	1.517654
H	-3.667737	0.591260	2.106091
H	-3.119702	1.092394	-2.116541
H	-1.742778	3.141684	-1.700252
H	-1.327152	3.890482	0.624159
H	-2.289991	2.638895	2.533226
S	-4.277330	-0.802240	-0.311125
O	-5.133138	-1.068618	0.852503
O	-4.824018	-0.772280	-1.675995

Supplementary Table 10: Cartesian coordinates of **TS-1f**

M062X SCF energy: -1498.23563088 a.u.
M062X enthalpy: -1497.858045 a.u.
M062X free energy: -1497.943373 a.u.
M062X SCF energy in solution: -1498.56491357 a.u.
M062X enthalpy in solution: -1498.187328 a.u.
M062X free energy in solution: -1498.272656 a.u.
Imaginary frequency: -408.3714 cm-1

Cartesian coordinates

ATOM	X	Y	Z
C	3.291929	-0.606298	0.134470
O	3.449677	-1.753825	-0.242563
Si	4.770669	0.626281	-0.055499
C	4.561315	2.077142	1.129011
H	4.558008	1.746519	2.172830
H	5.380585	2.794304	1.011524
H	3.624431	2.612580	0.941845
C	4.733383	1.221413	-1.840134
H	4.799636	0.371882	-2.526779
H	3.809329	1.764015	-2.064231
H	5.574684	1.891196	-2.046900
C	6.331549	-0.353008	0.295094
H	7.229336	0.209707	0.020825
H	6.407307	-0.618558	1.354030
H	6.314574	-1.281963	-0.282653
C	1.954496	-0.151686	0.701602
H	2.131331	0.174183	1.739075
H	1.647216	0.757233	0.161343
C	0.869945	-1.216314	0.625707
H	1.242810	-2.145997	1.069664
H	0.661894	-1.433186	-0.427124
C	-0.414742	-0.783874	1.330158
H	-0.236258	-0.696355	2.409199
H	-0.711528	0.219930	0.992860
S	-2.319414	-0.993729	-0.929321
O	-1.216273	-0.451687	-1.742052
O	-3.206219	-2.044977	-1.442161
C	-3.335167	0.391324	-0.438168
C	-4.627614	0.142262	0.015150
C	-2.786711	1.670191	-0.445358
C	-5.392041	1.212412	0.470341
H	-5.023322	-0.868010	-0.016179
C	-3.563931	2.731596	0.011307
H	-1.781178	1.819840	-0.825599

C	-4.859310	2.501113	0.470677
H	-6.405749	1.042538	0.818114
H	-3.160072	3.738711	0.003656
H	-5.460461	3.331931	0.826244
C	-1.555977	-1.761146	1.103892
H	-1.289662	-2.707624	0.628591
C	-2.686516	-1.759966	1.891883
H	-2.917387	-0.918305	2.538105
H	-3.415408	-2.559892	1.828120

Supplementary Table 11: Cartesian coordinates of **10a**

M062X SCF energy: -1498.25337970 a.u.

M062X enthalpy: -1497.873907 a.u.

M062X free energy: -1497.960304 a.u.

M062X SCF energy in solution: -1498.58188403 a.u.

M062X enthalpy in solution: -1498.202411 a.u.

M062X free energy in solution: -1498.288808 a.u.

Cartesian coordinates

ATOM	X	Y	Z
C	4.258159	-0.530398	-0.337391
O	4.403963	-1.614172	-0.875293
Si	5.843791	0.491034	0.094879
C	6.386209	1.320303	-1.505765
H	7.320991	1.872309	-1.362402
H	6.551674	0.571447	-2.286501
H	5.632726	2.027002	-1.868405
C	7.130858	-0.742244	0.678564
H	8.120125	-0.281273	0.760991
H	6.868690	-1.160283	1.655309
H	7.192180	-1.569764	-0.034529
C	5.446385	1.782382	1.407140
H	6.331010	2.384352	1.640292
H	4.660878	2.466473	1.068825
H	5.108072	1.316740	2.338563
C	2.866785	0.016649	-0.057171
H	2.797220	0.224811	1.021417
H	2.792761	1.003724	-0.541035
C	1.744898	-0.906016	-0.513031
H	1.857915	-1.876489	-0.016108
H	1.850311	-1.103942	-1.585182
C	0.359766	-0.327535	-0.216979
H	0.279197	-0.096559	0.858746

H	0.232628	0.638680	-0.725782
C	-0.743417	-1.249466	-0.612227
H	-0.630290	-2.311170	-0.417631
C	-2.103162	-0.745964	-0.897159
S	-2.966151	-0.382779	0.677056
O	-3.124275	-1.646783	1.392589
O	-2.279672	0.751249	1.295860
C	-4.580306	0.167415	0.158142
C	-5.593507	-0.773266	-0.003600
C	-4.780334	1.520170	-0.101535
C	-6.840848	-0.342440	-0.445703
H	-5.402626	-1.814316	0.237673
C	-6.032305	1.937821	-0.543302
H	-3.971037	2.224026	0.065656
C	-7.056160	1.007889	-0.717266
H	-7.646629	-1.058181	-0.570977
H	-6.211737	2.988889	-0.744197
H	-8.031383	1.339059	-1.059892
H	-2.735057	-1.473935	-1.414464
H	-2.100546	0.204246	-1.443125

Supplementary Table 12: Cartesian coordinates of **10b**

M062X SCF energy: -1498.24996091 a.u.

M062X enthalpy: -1497.871137 a.u.

M062X free energy: -1497.955147 a.u.

M062X SCF energy in solution: -1498.57848026 a.u.

M062X enthalpy in solution: -1498.199656 a.u.

M062X free energy in solution: -1498.283666 a.u.

Cartesian coordinates

ATOM	X	Y	Z
C	3.244037	-0.592946	-0.037108
O	3.334544	-1.656085	-0.624171
Si	4.797128	0.559750	0.016527
C	4.788252	1.533536	-1.594340
H	5.668711	2.180875	-1.664177
H	4.798641	0.854286	-2.452305
H	3.899365	2.167437	-1.676500
C	6.295167	-0.563563	0.117138
H	7.225243	-0.009548	-0.043698
H	6.357961	-1.057533	1.091614
H	6.217951	-1.342013	-0.647739
C	4.680299	1.727229	1.491207

H	5.542532	2.401716	1.521455
H	3.778417	2.346437	1.438792
H	4.656944	1.177286	2.437727
C	1.929928	-0.169157	0.605071
H	2.116258	-0.052214	1.684686
H	1.688779	0.843683	0.246065
C	0.783446	-1.135543	0.338248
H	1.097169	-2.146196	0.623796
H	0.572976	-1.168535	-0.734108
C	-0.474231	-0.750131	1.115178
H	-0.264126	-0.802964	2.190915
H	-0.750265	0.294690	0.914710
C	-1.681151	-1.657737	0.845539
C	-2.666536	-1.706154	1.947573
H	-2.730175	-0.885464	2.651658
H	-3.422005	-2.479959	1.971152
S	-2.551369	-1.177012	-0.710965
O	-1.556446	-0.971565	-1.764037
O	-3.641543	-2.134982	-0.883338
C	-3.272768	0.413679	-0.340892
C	-4.503050	0.463676	0.309922
C	-2.594450	1.568264	-0.720570
C	-5.054423	1.706747	0.605118
H	-5.018776	-0.459613	0.554326
C	-3.157473	2.806129	-0.421221
H	-1.656020	1.482585	-1.259739
C	-4.380141	2.872608	0.243917
H	-6.014448	1.766553	1.107153
H	-2.646579	3.717407	-0.714278
H	-4.816548	3.839645	0.473231
H	-1.353781	-2.671625	0.573024

Supplementary Table 13: Cartesian coordinates of **TS-2a**

M062X SCF energy: -1498.24928606 a.u.

M062X enthalpy: -1497.870211 a.u.

M062X free energy: -1497.947014 a.u.

M062X SCF energy in solution: -1498.57758325 a.u.

M062X enthalpy in solution: -1498.198508 a.u.

M062X free energy in solution: -1498.275311 a.u.

Imaginary frequency: -523.3122 cm-1

Cartesian coordinates

ATOM	X	Y	Z
C	-0.965107	1.018566	0.171900

C	-2.423311	0.084033	-1.136063
C	-3.232924	1.359408	-1.397538
C	-2.916837	2.417232	-0.338728
C	-1.395032	2.418427	-0.147658
H	-1.222004	0.666676	1.171236
H	-2.955921	1.713613	-2.397112
H	-4.302891	1.113375	-1.410297
H	-3.282568	3.404098	-0.636371
H	-3.400687	2.164626	0.612158
H	-0.906558	2.754597	-1.069934
H	-1.088756	3.102089	0.654615
O	-1.742323	-0.412167	-2.054605
Si	-2.976543	-1.198875	0.213352
C	-1.496890	-2.216086	0.762606
H	-0.789331	-1.656142	1.384414
H	-1.840551	-3.079118	1.343216
H	-0.965839	-2.599971	-0.115257
C	-3.863740	-0.399479	1.671735
H	-4.708073	0.211607	1.334106
H	-4.268302	-1.184702	2.320331
H	-3.211303	0.228612	2.285946
C	-4.193107	-2.301700	-0.709417
H	-3.710327	-2.739345	-1.588514
H	-4.547929	-3.117136	-0.069968
H	-5.068240	-1.738844	-1.050677
C	0.319875	0.474553	-0.354591
H	0.271531	-0.604556	-0.533905
H	0.636346	0.972205	-1.275138
S	1.601926	0.739793	0.890841
O	1.834992	2.179286	0.997795
O	1.214929	-0.043345	2.066186
C	3.048070	-0.012027	0.175445
C	3.936567	0.782716	-0.541929
C	3.239113	-1.382388	0.331116
C	5.047867	0.180608	-1.125178
H	3.759864	1.850927	-0.617208
C	4.352471	-1.971578	-0.259429
H	2.534622	-1.961229	0.920747
C	5.250789	-1.191225	-0.986307
H	5.757619	0.782239	-1.683218
H	4.523492	-3.037051	-0.147190
H	6.119000	-1.655565	-1.443140

Supplementary Table 14: Cartesian coordinates of **TS-2b**
M062X SCF energy: -1498.24643096 a.u.
M062X enthalpy: -1497.867567 a.u.
M062X free energy: -1497.944563 a.u.
M062X SCF energy in solution: -1498.57393452 a.u.
M062X enthalpy in solution: -1498.195071 a.u.
M062X free energy in solution: -1498.272067 a.u.
Imaginary frequency: -498.7734 cm-1

Cartesian coordinates

ATOM	X	Y	Z
C	0.570637	2.398891	-0.521486
C	2.013600	1.912732	-0.648758
C	2.349596	0.843839	0.399198
C	0.983823	-0.667162	-0.331641
C	-0.305924	0.051898	-0.112250
C	-0.446521	1.331407	-0.936303
H	2.218133	1.538562	-1.660029
H	2.706326	2.746783	-0.470141
H	0.401034	2.685210	0.521861
H	0.417291	3.290033	-1.138145
H	-0.445202	0.244641	0.958244
H	-0.319355	1.071356	-1.994636
H	-1.460157	1.731527	-0.821178
O	1.927268	1.003517	1.560457
Si	3.930527	-0.261492	0.219971
C	5.346217	0.915955	0.621333
H	6.306147	0.388649	0.623901
H	5.413289	1.727288	-0.111123
H	5.201428	1.362999	1.609884
C	3.805148	-1.616762	1.511251
H	3.285490	-1.219897	2.389134
H	3.249761	-2.486769	1.147661
H	4.796481	-1.959902	1.822949
C	4.154957	-0.943904	-1.522025
H	4.044065	-0.162557	-2.281674
H	5.162356	-1.360893	-1.628676
H	3.443616	-1.744000	-1.748737
S	-1.630943	-1.136223	-0.549566
O	-1.693605	-1.212526	-2.009262
O	-1.409837	-2.333812	0.259008
C	-3.127124	-0.356511	0.025339
C	-3.918602	0.344917	-0.879369
C	-3.465945	-0.468700	1.371306

C	-5.078659	0.959340	-0.415772
H	-3.627887	0.383886	-1.924495
C	-4.626002	0.152987	1.822188
H	-2.839766	-1.053162	2.038425
C	-5.426469	0.866198	0.930596
H	-5.713323	1.505602	-1.105683
H	-4.910296	0.074160	2.866245
H	-6.332081	1.346731	1.287013
H	1.200342	-1.496633	0.333845
H	1.307015	-0.781327	-1.364432

Supplementary Table 15: Cartesian coordinates of **11a**

M062X SCF energy: -1498.27995893 a.u.

M062X enthalpy: -1497.898070 a.u.

M062X free energy: -1497.974397 a.u.

M062X SCF energy in solution: -1498.60512700 a.u.

M062X enthalpy in solution: -1498.223238 a.u.

M062X free energy in solution: -1498.299565 a.u.

Cartesian coordinates

ATOM	X	Y	Z
C	-1.046500	0.923594	-0.027684
C	-2.174641	0.249946	-0.895215
C	-3.237365	1.355119	-1.161453
C	-2.841186	2.511901	-0.238434
C	-1.310793	2.426253	-0.184765
H	-1.172143	0.663700	1.032963
H	-3.129991	1.641813	-2.213661
H	-4.270226	1.019694	-1.020279
H	-3.200621	3.479810	-0.597013
H	-3.253581	2.357094	0.766287
H	-0.888033	2.771348	-1.137468
H	-0.859498	3.017924	0.614705
O	-1.803926	-0.498447	-1.908980
Si	-2.953306	-1.237536	0.181598
C	-1.529178	-2.322498	0.733660
H	-0.787917	-1.758904	1.312842
H	-1.898846	-3.135729	1.367988
H	-1.032537	-2.769169	-0.133283
C	-3.814376	-0.444715	1.656439
H	-4.607382	0.242190	1.343999
H	-4.276561	-1.230913	2.264675
H	-3.115762	0.100886	2.298057

C	-4.180672	-2.146991	-0.900414
H	-3.668632	-2.602012	-1.751927
H	-4.688872	-2.934490	-0.333862
H	-4.945335	-1.468258	-1.291141
C	0.321222	0.433381	-0.477249
H	0.313539	-0.643038	-0.673254
H	0.669443	0.947755	-1.378112
S	1.525334	0.719912	0.819816
O	1.742911	2.160592	0.949855
O	1.103831	-0.075020	1.977172
C	3.016283	-0.007808	0.169543
C	3.922268	0.802679	-0.506864
C	3.229157	-1.373092	0.339351
C	5.071669	0.222215	-1.036000
H	3.728319	1.867114	-0.592718
C	4.380764	-1.941119	-0.196035
H	2.509972	-1.964415	0.898038
C	5.296034	-1.144687	-0.883250
H	5.794437	0.837136	-1.562005
H	4.568393	-3.002455	-0.071353
H	6.194133	-1.592230	-1.297068

Supplementary Table 16: Cartesian coordinates of **11b**

M062X SCF energy: -1498.28761133 a.u.

M062X enthalpy: -1497.905109 a.u.

M062X free energy: -1497.981681 a.u.

M062X SCF energy in solution: -1498.61426597 a.u.

M062X enthalpy in solution: -1498.231764 a.u.

M062X free energy in solution: -1498.308336 a.u.

Cartesian coordinates

ATOM	X	Y	Z
C	-0.608903	2.450631	0.177562
C	-2.009354	1.864282	0.398152
C	-2.094997	0.518579	-0.346384
C	-1.048017	-0.482565	0.182799
C	0.338119	0.131723	-0.048312
C	0.483544	1.484211	0.647914
H	-2.189888	1.723647	1.472116
H	-2.769997	2.545900	0.005106
H	-0.487603	2.651514	-0.893973
H	-0.506192	3.405193	0.703711
H	-1.181377	-0.664538	1.256627

H	-1.124404	-1.433392	-0.352070
H	0.512550	0.218381	-1.128473
H	0.403240	1.323180	1.730537
H	1.473991	1.909351	0.451282
O	-2.139739	0.621912	-1.664226
Si	-3.912102	-0.267614	-0.122934
C	-5.145902	1.039814	-0.651663
H	-6.154651	0.615914	-0.699093
H	-5.169909	1.881284	0.047454
H	-4.885593	1.421784	-1.642796
C	-4.009128	-1.802097	-1.189898
H	-3.274393	-2.552753	-0.882960
H	-5.002314	-2.257822	-1.115231
H	-3.819114	-1.548558	-2.235993
C	-4.102626	-0.695166	1.700449
H	-3.956238	0.177507	2.344329
H	-5.116179	-1.072872	1.878141
H	-3.398704	-1.473772	2.009201
S	1.569363	-1.052389	0.545457
O	1.536391	-1.033621	2.009230
O	1.366166	-2.298871	-0.192224
C	3.128881	-0.359716	0.025868
C	3.867795	0.401554	0.926506
C	3.567835	-0.596108	-1.274087
C	5.075712	0.950737	0.504940
H	3.499161	0.537031	1.938476
C	4.775478	-0.039633	-1.683932
H	2.978524	-1.224039	-1.935289
C	5.523644	0.733245	-0.796758
H	5.669741	1.542665	1.193448
H	5.137424	-0.215741	-2.691495
H	6.466510	1.162635	-1.120470

Supplementary Table 17: Cartesian coordinates of **TS-3a**

M062X SCF energy: -1498.27715880 a.u.

M062X enthalpy: -1497.896196 a.u.

M062X free energy: -1497.971249 a.u.

M062X SCF energy in solution: -1498.59949439 a.u.

M062X enthalpy in solution: -1498.218532 a.u.

M062X free energy in solution: -1498.293585 a.u.

Imaginary frequency: -246.5142 cm-1

Cartesian coordinates

ATOM	X	Y	Z
C	-1.026771	0.973408	0.006960
C	-2.170518	0.317957	-0.822442
C	-3.143490	1.430378	-1.235102
C	-2.788522	2.596249	-0.307786
C	-1.264845	2.482692	-0.158054
H	-1.142044	0.715349	1.069945
H	-2.924360	1.681601	-2.281579
H	-4.194964	1.128064	-1.186586
H	-3.105200	3.565643	-0.700059
H	-3.268811	2.458482	0.668740
H	-0.777889	2.828387	-1.079036
H	-0.854541	3.064820	0.669599
O	-1.910398	-0.692062	-1.639247
Si	-2.966217	-1.262071	0.125244
C	-1.578952	-2.315193	0.810321
H	-0.834007	-1.705290	1.336450
H	-1.978137	-3.035407	1.533052
H	-1.077747	-2.868860	0.011895
C	-3.884609	-0.443210	1.570264
H	-4.671352	0.231467	1.216429
H	-4.360316	-1.219648	2.181566
H	-3.208443	0.123765	2.217861
C	-4.226871	-2.173944	-0.913580
H	-3.736014	-2.812600	-1.650997
H	-4.876984	-2.789073	-0.282582
H	-4.860318	-1.464042	-1.455640
C	0.326378	0.453348	-0.458290
H	0.294382	-0.627857	-0.625227
H	0.665354	0.942957	-1.376642
S	1.558487	0.754333	0.809165
O	1.802995	2.194198	0.890347
O	1.141088	0.002439	1.995629
C	3.024100	-0.020368	0.155946
C	3.935433	0.753257	-0.555511
C	3.212500	-1.384610	0.360102
C	5.065077	0.135668	-1.085237
H	3.761369	1.818725	-0.667340
C	4.344463	-1.989877	-0.176367
H	2.490417	-1.946138	0.945187
C	5.264747	-1.230827	-0.898361
H	5.791956	0.721455	-1.638151
H	4.513075	-3.050969	-0.025287
H	6.147609	-1.707316	-1.312658

Supplementary Table 18: Cartesian coordinates of **TS-3b**

M062X SCF energy: -1498.28435507 a.u.

M062X enthalpy: -1497.902739 a.u.

M062X free energy: -1497.978298 a.u.

M062X SCF energy in solution: -1498.60831168 a.u.

M062X enthalpy in solution: -1498.226696 a.u.

M062X free energy in solution: -1498.302255 a.u.

Imaginary frequency: -245.6018 cm-1

Cartesian coordinates

ATOM	X	Y	Z
C	-0.586288	2.471164	0.053189
C	-1.971593	1.908859	0.410833
C	-2.074674	0.503945	-0.172489
C	-1.018926	-0.443038	0.393584
C	0.356772	0.139154	0.030207
C	0.532661	1.558955	0.567880
H	-2.091137	1.875223	1.501274
H	-2.757696	2.544961	-0.006954
H	-0.521210	2.556842	-1.038367
H	-0.463430	3.476487	0.468477
H	-1.102165	-0.510626	1.485466
H	-1.120634	-1.443226	-0.035766
H	0.480048	0.107964	-1.059921
H	0.509589	1.516567	1.664225
H	1.509868	1.957958	0.274431
O	-2.324368	0.409990	-1.477811
Si	-3.921385	-0.272454	-0.240722
C	-5.145448	0.949946	-0.955172
H	-6.167969	0.596619	-0.782164
H	-5.053751	1.931973	-0.480449
H	-4.992164	1.073304	-2.029314
C	-3.948748	-1.949721	-1.067852
H	-3.137676	-2.587298	-0.702257
H	-4.892962	-2.460461	-0.849391
H	-3.841062	-1.852080	-2.150172
C	-4.312158	-0.507819	1.602217
H	-4.275040	0.440781	2.147236
H	-5.322499	-0.920221	1.711359
H	-3.612159	-1.203992	2.075001
S	1.615030	-0.980575	0.689989
O	1.649833	-0.802345	2.142580

O	1.374492	-2.297403	0.101270
C	3.148398	-0.357176	0.025876
C	3.923236	0.502790	0.798658
C	3.531736	-0.745400	-1.255067
C	5.109926	0.996595	0.263816
H	3.599104	0.756081	1.803220
C	4.719106	-0.243369	-1.778757
H	2.916682	-1.445503	-1.812067
C	5.502169	0.626921	-1.021485
H	5.731318	1.663695	0.852135
H	5.038187	-0.537650	-2.773122
H	6.428995	1.013385	-1.433523

Supplementary Table 19: Cartesian coordinates of **12a**

M062X SCF energy: -1498.31652454 a.u.

M062X enthalpy: -1497.934514 a.u.

M062X free energy: -1498.013398 a.u.

M062X SCF energy in solution: -1498.63684444 a.u.

M062X enthalpy in solution: -1498.254834 a.u.

M062X free energy in solution: -1498.333718 a.u.

Cartesian coordinates

ATOM	X	Y	Z
C	1.871536	2.939651	1.143207
C	0.854590	2.936147	-0.012082
C	0.820962	1.473149	-0.508006
C	2.135165	0.910388	-0.026495
C	2.952226	1.952980	0.680615
H	2.262423	3.937494	1.355772
H	1.403014	2.564763	2.060942
H	1.228088	3.570394	-0.822864
H	-0.130279	3.311608	0.274300
H	0.758189	1.409355	-1.602151
H	3.561274	1.556760	1.500400
H	3.644774	2.436995	-0.028904
O	2.703021	-0.102734	-0.737020
Si	3.256188	-1.519449	-0.011943
C	4.536335	-1.134075	1.304044
H	5.023246	-2.056950	1.638508
H	5.313450	-0.466505	0.917975
H	4.087251	-0.660971	2.183204
C	1.795372	-2.406493	0.762218
H	1.059160	-2.696747	0.005146

H	2.115716	-3.314634	1.284009
H	1.294967	-1.761357	1.493189
C	4.002847	-2.507633	-1.409726
H	4.852355	-1.978464	-1.851985
H	4.356948	-3.482477	-1.058873
H	3.266465	-2.679009	-2.200649
C	-0.332043	0.637357	0.065894
H	-0.433363	0.760137	1.149616
H	-0.186210	-0.422978	-0.166646
S	-1.915887	1.091492	-0.665250
O	-2.363315	2.352827	-0.072885
O	-1.785996	0.948881	-2.114767
C	-3.008528	-0.194003	-0.083866
C	-3.701022	-0.003612	1.107825
C	-3.131951	-1.362371	-0.830460
C	-4.531765	-1.021274	1.568502
H	-3.599897	0.935752	1.642276
C	-3.965447	-2.372120	-0.359240
H	-2.598046	-1.456593	-1.771098
C	-4.659136	-2.201506	0.838115
H	-5.085511	-0.890415	2.492395
H	-4.080975	-3.287984	-0.929501
H	-5.310022	-2.991055	1.200244

Supplementary Table 20: Cartesian coordinates of **12b**

M062X SCF energy: -1498.32824986 a.u.

M062X enthalpy: -1497.945334 a.u.

M062X free energy: -1498.022582 a.u.

M062X SCF energy in solution: -1498.64745913 a.u.

M062X enthalpy in solution: -1498.264543 a.u.

M062X free energy in solution: -1498.341791 a.u.

Cartesian coordinates

ATOM	X	Y	Z
C	0.504190	2.142133	1.409275
C	1.787911	2.251146	0.560829
C	2.001388	0.952032	-0.156600
C	0.880253	0.524973	-1.057599
C	-0.394188	0.412968	-0.191227
C	-0.689428	1.723479	0.541022
H	1.670915	3.061005	-0.170227
H	2.650908	2.482797	1.193143
H	0.667204	1.394560	2.194874
H	0.285981	3.094846	1.902895

H	0.705727	1.277210	-1.836501
H	1.093884	-0.434587	-1.536381
H	-0.276716	-0.418264	0.515276
H	-0.898047	2.496602	-0.209719
H	-1.586719	1.617530	1.160821
O	2.508984	-0.055588	0.632537
Si	4.029044	-0.688420	0.260004
C	5.299104	0.682942	0.406527
H	6.302205	0.326465	0.149586
H	5.054226	1.509029	-0.269970
H	5.333074	1.080565	1.426064
C	4.319738	-2.033273	1.524709
H	3.553600	-2.811116	1.451727
H	5.295757	-2.506652	1.375683
H	4.293278	-1.627668	2.540672
C	4.001336	-1.374050	-1.483748
H	3.754154	-0.590766	-2.208469
H	4.977794	-1.786632	-1.759399
H	3.258329	-2.171875	-1.584223
S	-1.757209	-0.067529	-1.275615
O	-2.095575	1.090187	-2.105452
O	-1.394961	-1.347180	-1.884579
C	-3.125057	-0.360884	-0.167961
C	-4.045561	0.658087	0.058474
C	-3.236269	-1.604595	0.447669
C	-5.099591	0.424686	0.937618
H	-3.935126	1.602713	-0.464854
C	-4.293140	-1.824803	1.325696
H	-2.517353	-2.384797	0.217221
C	-5.218579	-0.811191	1.570782
H	-5.831139	1.204240	1.123190
H	-4.400431	-2.789060	1.811469
H	-6.043004	-0.989042	2.254071

Supplementary Table 21: Cartesian coordinates of **TS-4a**

M062X SCF energy: -2739.83894765 a.u.
M062X enthalpy: -2739.171321 a.u.
M062X free energy: -2739.290341 a.u.
M062X SCF energy in solution: -2740.46846123 a.u.
M062X enthalpy in solution: -2739.800835 a.u.
M062X free energy in solution: -2739.919855 a.u.
Imaginary frequency: -226.7798 cm-1

Cartesian coordinates

ATOM	X	Y	Z
C	-2.019714	1.183641	-0.205109
C	-0.818060	0.899714	0.680313
C	-0.469978	2.106277	1.505886
C	-1.073127	3.257154	0.692387
C	-2.369070	2.653127	0.127067
H	-1.758756	1.087144	-1.268882
H	-0.953631	2.048276	2.497182
H	0.607955	2.200689	1.682713
H	-1.256081	4.148683	1.296532
H	-0.385837	3.546785	-0.111301
H	-3.148944	2.670937	0.897465
H	-2.765698	3.191284	-0.736631
O	-0.625157	-0.364269	1.114700
Si	-0.195457	-0.912376	2.663170
C	0.271701	-2.703863	2.395640
H	0.338736	-3.224977	3.356796
H	-0.480623	-3.216525	1.788295
H	1.240447	-2.809410	1.896887
C	1.223057	0.055542	3.409650
H	0.886549	0.991390	3.866102
H	1.681717	-0.549232	4.201058
H	2.009547	0.303488	2.687789
C	-1.728071	-0.788833	3.739395
H	-2.540773	-1.400119	3.333104
H	-1.520433	-1.139508	4.756204
H	-2.087336	0.243412	3.814404
C	-3.145961	0.186756	0.069179
H	-2.817312	-0.837706	-0.128177
H	-3.517743	0.254955	1.097476
S	-4.544713	0.513317	-1.015889
O	-5.276694	1.665302	-0.490793
O	-4.038231	0.509258	-2.387607
C	-5.571443	-0.928881	-0.801724
C	-6.559735	-0.913478	0.177619
C	-5.351766	-2.038785	-1.612806
C	-7.341638	-2.051419	0.355499
H	-6.718135	-0.015180	0.765971
C	-6.140079	-3.169832	-1.424557
H	-4.591175	-1.996856	-2.386378
C	-7.128347	-3.175136	-0.440985
H	-8.122326	-2.058308	1.108977
H	-5.989955	-4.043328	-2.050403

H	-7.742580	-4.058910	-0.300262
C	2.558272	-0.628629	0.018552
H	3.267636	-0.639327	0.850425
H	1.749170	-1.346525	0.179799
C	2.823031	1.855916	0.377948
O	3.556000	1.638534	1.334729
C	2.626005	3.258027	-0.112600
C	2.563345	4.283060	0.835007
C	2.544659	3.564071	-1.473760
C	2.370668	5.598473	0.428926
H	2.665406	4.028133	1.885848
C	2.373360	4.885108	-1.879020
H	2.641105	2.769096	-2.207833
C	2.272061	5.899365	-0.929188
H	2.304447	6.390765	1.167874
H	2.321380	5.122455	-2.936720
H	2.126373	6.926907	-1.247618
S	3.445258	-1.204314	-1.452466
O	2.444609	-1.564319	-2.456848
O	4.491774	-0.231091	-1.754788
C	4.223019	-2.704121	-0.885319
C	3.524085	-3.904112	-0.978269
C	5.497872	-2.631977	-0.331524
C	4.121375	-5.064352	-0.493758
H	2.543477	-3.919297	-1.443750
C	6.084163	-3.799704	0.147536
H	6.014710	-1.677884	-0.298298
C	5.395649	-5.009418	0.068751
H	3.597054	-6.011838	-0.562831
H	7.080081	-3.767199	0.576879
H	5.858422	-5.917316	0.442596
C	2.061520	0.755969	-0.254458
C	0.911876	0.948371	-0.950818
H	0.411563	0.101647	-1.412960
H	0.588460	1.937119	-1.256922

Supplementary Table 22: Cartesian coordinates of **TS-4b**

M062X SCF energy: -2739.83740962 a.u.

M062X enthalpy: -2739.168777 a.u.

M062X free energy: -2739.293792 a.u.

M062X SCF energy in solution: -2740.45590295 a.u.

M062X enthalpy in solution: -2739.787270 a.u.

M062X free energy in solution: -2739.912285 a.u.

Imaginary frequency: -116.4562 cm-1

Cartesian coordinates

ATOM	X	Y	Z
O	0.387454	-0.151967	1.933279
Si	-0.511892	-0.020385	3.368315
C	-0.918078	1.802791	3.470996
H	-1.657061	2.102130	2.720620
H	-1.332216	2.045918	4.455380
H	-0.020204	2.411155	3.325384
C	-2.072030	-1.055252	3.333383
H	-2.749245	-0.693714	4.116523
H	-2.610643	-0.997888	2.380926
H	-1.871171	-2.111475	3.537377
C	0.596833	-0.537280	4.788921
H	0.910074	-1.582749	4.697629
H	1.499799	0.080048	4.826055
H	0.074886	-0.431997	5.746376
C	2.758722	-0.172986	0.128593
H	2.384493	-0.110186	-0.897704
H	2.521656	0.751235	0.665713
S	4.551131	-0.270736	0.008892
O	5.083773	-0.291875	1.370988
O	4.881635	-1.333897	-0.939443
C	4.993496	1.287368	-0.734286
C	5.278949	2.371519	0.090442
C	5.027806	1.385136	-2.122140
C	5.596969	3.591996	-0.497644
H	5.269122	2.242794	1.168219
C	5.345360	2.611914	-2.697501
H	4.827865	0.507187	-2.728374
C	5.625294	3.710407	-1.886377
H	5.829086	4.448561	0.126599
H	5.380537	2.709269	-3.777453
H	5.874126	4.664461	-2.340160
C	-0.433836	-0.823539	-0.855762
C	-1.727853	-0.720093	-0.470385
C	-2.280334	0.599947	-0.027786
H	-3.220118	0.471961	0.515864
H	-1.564173	1.183026	0.558359
C	-2.648082	-1.876931	-0.363297
O	-3.642993	-1.817738	0.346743
C	-2.313829	-3.151662	-1.076689
C	-2.560773	-4.356014	-0.411945

C	-1.812517	-3.168856	-2.381160
C	-2.260847	-5.566718	-1.025435
H	-2.984679	-4.321784	0.587408
C	-1.532592	-4.383630	-3.001510
H	-1.670050	-2.232455	-2.912803
C	-1.742280	-5.580501	-2.320077
H	-2.436413	-6.499914	-0.499763
H	-1.153676	-4.395335	-4.018398
H	-1.511759	-6.525648	-2.801692
S	-2.639100	1.585638	-1.502967
O	-1.362027	2.069764	-2.029177
O	-3.550507	0.817643	-2.347313
C	-3.527913	2.981173	-0.841981
C	-2.813191	4.097015	-0.416498
C	-4.913658	2.905841	-0.735857
C	-3.511389	5.165714	0.138447
H	-1.735469	4.124671	-0.543410
C	-5.599458	3.981887	-0.180366
H	-5.432494	2.024476	-1.099866
C	-4.898906	5.104956	0.257332
H	-2.974553	6.048186	0.470677
H	-6.680427	3.946754	-0.094045
H	-5.439162	5.941784	0.688623
C	0.771062	-1.258580	1.264845
C	0.219077	-2.624086	1.538368
C	2.218151	-1.388969	0.872343
C	1.050627	-3.554145	0.626101
H	-0.859035	-2.684576	1.347367
H	0.362233	-2.884241	2.601503
C	2.255917	-2.721542	0.103912
H	2.800857	-1.479024	1.807853
H	1.387300	-4.434776	1.177435
H	0.439046	-3.917659	-0.206216
H	3.210830	-3.232635	0.236033
H	2.160149	-2.526751	-0.969773
H	0.166759	0.075851	-0.948958
H	-0.025136	-1.746430	-1.253063

Supplementary Table 23: Cartesian coordinates of **13a**

M062X SCF energy: -2739.88391899 a.u.

M062X enthalpy: -2739.212161 a.u.

M062X free energy: -2739.332941 a.u.

M062X SCF energy in solution: -2740.49855632 a.u.

M062X enthalpy in solution: -2739.826798 a.u.
M062X free energy in solution: -2739.947578 a.u.

Cartesian coordinates

ATOM	X	Y	Z
C	-1.781511	0.846691	-0.927599
C	-0.578912	0.676986	0.040629
C	-0.792175	1.828047	1.038304
C	-1.272901	3.006658	0.175141
C	-1.898489	2.376021	-1.101906
H	-1.583157	0.334394	-1.875317
H	-1.569002	1.532436	1.755372
H	0.104945	2.061970	1.618584
H	-1.991064	3.626056	0.717512
H	-0.429986	3.655995	-0.084476
H	-2.936929	2.679392	-1.248784
H	-1.350601	2.691379	-1.994167
O	-0.535511	-0.616639	0.600707
Si	-0.585237	-1.155905	2.186776
C	0.035593	-2.920956	2.080213
H	1.107424	-2.961280	1.860197
H	-0.126450	-3.445967	3.027713
H	-0.490543	-3.472891	1.295037
C	0.506381	-0.155888	3.341721
H	0.036566	0.783278	3.649582
H	0.688250	-0.743448	4.249602
H	1.482994	0.085748	2.906500
C	-2.350842	-1.165409	2.838493
H	-3.011869	-1.774589	2.211931
H	-2.367724	-1.592365	3.847976
H	-2.782393	-0.160823	2.903824
C	-3.026632	0.210759	-0.314185
H	-2.824567	-0.828766	-0.042674
H	-3.399288	0.748769	0.564676
S	-4.396227	0.153829	-1.480860
O	-5.015111	1.477758	-1.557488
O	-3.921558	-0.522202	-2.686815
C	-5.546726	-0.931251	-0.653684
C	-6.517497	-0.378880	0.175623
C	-5.430286	-2.305956	-0.840505
C	-7.389555	-1.233015	0.845315
H	-6.591193	0.699943	0.269604
C	-6.307363	-3.148835	-0.164812
H	-4.677791	-2.693587	-1.520503

C	-7.280585	-2.612115	0.677348
H	-8.157941	-0.822370	1.492018
H	-6.238142	-4.222984	-0.301639
H	-7.963995	-3.273823	1.200068
C	0.728579	0.874231	-0.775616
H	0.699782	1.864526	-1.235952
H	0.736951	0.125494	-1.576013
C	1.948978	0.701209	0.067592
C	2.547366	-0.650820	0.188258
H	3.164107	-0.733976	1.087328
H	1.807413	-1.452524	0.117738
C	2.590535	1.764611	0.841227
O	3.262766	1.486957	1.834030
C	2.376056	3.193026	0.449601
C	2.120211	4.133426	1.449292
C	2.470974	3.599512	-0.883698
C	1.905579	5.465559	1.112138
H	2.086625	3.803082	2.483530
C	2.276399	4.937035	-1.216629
H	2.726500	2.870586	-1.649181
C	1.978493	5.866280	-0.221462
H	1.687648	6.193169	1.887399
H	2.360501	5.254332	-2.250984
H	1.813926	6.906575	-0.484126
S	3.663268	-0.865337	-1.241994
O	2.835985	-1.202081	-2.399926
O	4.549358	0.296414	-1.273038
C	4.623344	-2.297150	-0.802822
C	4.193523	-3.551703	-1.223666
C	5.767227	-2.124129	-0.028083
C	4.934883	-4.668896	-0.849007
H	3.308942	-3.635486	-1.846973
C	6.497225	-3.249859	0.339574
H	6.077489	-1.124118	0.259338
C	6.079329	-4.516242	-0.068468
H	4.623090	-5.656800	-1.171405
H	7.395273	-3.139188	0.938136
H	6.654222	-5.390657	0.219619

Supplementary Table 24: Cartesian coordinates of **13b**

M062X SCF energy: -2739.87905137 a.u.

M062X enthalpy: -2739.207395 a.u.

M062X free energy: -2739.328823 a.u.

M062X SCF energy in solution: -2740.49424004 a.u.
M062X enthalpy in solution: -2739.822584 a.u.
M062X free energy in solution: -2739.944012 a.u.

Cartesian coordinates

ATOM	X	Y	Z
O	-0.501024	1.583027	-1.622095
Si	-1.452100	2.936670	-1.875985
C	-2.588083	2.407718	-3.268088
H	-3.252287	1.594940	-2.956157
H	-3.216532	3.242132	-3.597097
H	-2.011048	2.057498	-4.129262
C	-2.489344	3.443601	-0.391761
H	-1.899054	3.774961	0.468760
H	-3.117787	4.289972	-0.696187
H	-3.155033	2.652458	-0.035379
C	-0.442660	4.424768	-2.419594
H	0.139583	4.217086	-3.322653
H	-1.123157	5.253413	-2.647969
H	0.245594	4.773798	-1.643641
C	2.691566	-0.045930	-0.643845
H	2.956714	0.416972	0.313447
H	2.269691	-1.037027	-0.451408
S	4.254258	-0.336149	-1.496967
O	3.938766	-0.775402	-2.854903
O	5.130049	0.812868	-1.262962
C	4.940678	-1.723490	-0.610490
C	4.592895	-3.012950	-1.003551
C	5.799227	-1.482762	0.457465
C	5.112963	-4.090473	-0.293503
H	3.943626	-3.157480	-1.861639
C	6.313805	-2.569862	1.158668
H	6.067609	-0.461878	0.709970
C	5.967612	-3.867178	0.785466
H	4.857989	-5.103693	-0.585842
H	6.990107	-2.404759	1.990920
H	6.372439	-4.711400	1.334553
C	-0.091143	0.036435	0.157445
H	0.613023	-0.195014	0.959418
H	-0.159849	-0.839308	-0.500098
C	-1.452725	0.319514	0.707808
C	-2.630596	-0.139040	-0.070134
H	-3.546739	0.345571	0.275488
H	-2.493661	-0.041883	-1.151254

C	-1.738013	0.992599	1.972312
O	-2.855508	1.463309	2.184262
C	-0.658684	1.145062	2.998839
C	-0.476608	2.390450	3.602702
C	0.114380	0.054086	3.403524
C	0.510995	2.558000	4.567486
H	-1.113888	3.217548	3.303504
C	1.088415	0.219463	4.384291
H	-0.077228	-0.926332	2.973773
C	1.296877	1.473544	4.954967
H	0.663736	3.530733	5.024025
H	1.679082	-0.631472	4.708244
H	2.063735	1.603372	5.712214
S	-2.827394	-1.926848	0.259691
O	-1.914597	-2.650172	-0.626203
O	-2.749248	-2.103593	1.708800
C	-4.494269	-2.261889	-0.262426
C	-4.715100	-2.733947	-1.552205
C	-5.535393	-2.020610	0.630383
C	-6.025950	-2.966633	-1.959491
H	-3.870394	-2.928572	-2.205224
C	-6.839786	-2.255870	0.208554
H	-5.315352	-1.671695	1.634839
C	-7.081615	-2.725020	-1.082444
H	-6.222419	-3.341081	-2.958623
H	-7.666977	-2.079573	0.887993
H	-8.101514	-2.908540	-1.405196
C	0.481894	1.211706	-0.671663
C	0.990321	2.398067	0.186942
C	1.737681	0.806009	-1.488952
C	2.213728	2.984414	-0.564495
H	1.277706	2.039899	1.181131
H	0.196364	3.132392	0.352558
C	2.313496	2.177014	-1.868942
H	1.432968	0.222930	-2.362375
H	2.123710	4.057222	-0.749633
H	3.125007	2.843289	0.025648
H	1.667628	2.609293	-2.638010
H	3.329280	2.137736	-2.265435

Supplementary Table 25: Cartesian coordinates of **TS-5a**

M062X SCF energy: -2739.86217583 a.u.

M062X enthalpy: -2739.192517 a.u.

M062X free energy: -2739.313084 a.u.
M062X SCF energy in solution: -2740.47514580 a.u.
M062X enthalpy in solution: -2739.805487 a.u.
M062X free energy in solution: -2739.926054 a.u.
Imaginary frequency: -236.9438 cm-1

Cartesian coordinates

ATOM	X	Y	Z
C	-1.889675	0.856502	-0.777355
C	-0.761038	0.264980	0.112118
C	-0.717148	1.256468	1.288603
C	-0.920527	2.638125	0.643621
C	-1.663176	2.380736	-0.698547
H	-1.805275	0.476993	-1.801553
H	-1.544288	1.023882	1.972118
H	0.204172	1.187263	1.875319
H	-1.486466	3.301489	1.301984
H	0.045462	3.121693	0.464321
H	-2.607433	2.925357	-0.762045
H	-1.053127	2.710999	-1.543918
O	-1.021709	-1.074694	0.450213
Si	-1.142978	-1.875088	1.915799
C	-0.808243	-3.667249	1.488307
H	0.240409	-3.816179	1.211117
H	-1.023938	-4.320495	2.340507
H	-1.427805	-3.992704	0.646976
C	0.078098	-1.287815	3.215435
H	-0.228527	-0.348481	3.685619
H	0.130435	-2.046095	4.006227
H	1.090880	-1.154744	2.819114
C	-2.892687	-1.702574	2.589219
H	-3.640410	-2.066112	1.875479
H	-3.001415	-2.287758	3.509530
H	-3.141003	-0.663467	2.833096
C	-3.249176	0.418789	-0.237324
H	-3.282254	-0.669301	-0.137394
H	-3.501559	0.877964	0.725122
S	-4.585886	0.852464	-1.361994
O	-4.909024	2.270552	-1.197642
O	-4.250318	0.309200	-2.677168
C	-5.951456	-0.091354	-0.706692
C	-6.802556	0.504634	0.218257
C	-6.118864	-1.409675	-1.121923
C	-7.845754	-0.249204	0.749475

H	-6.652426	1.544117	0.492159
C	-7.164293	-2.152685	-0.582562
H	-5.450041	-1.826695	-1.868671
C	-8.021622	-1.573279	0.352302
H	-8.524783	0.198567	1.467619
H	-7.316065	-3.179882	-0.897367
H	-8.837271	-2.156327	0.768166
C	0.557840	0.286354	-0.701442
H	0.744540	1.306522	-1.043067
H	0.403695	-0.336494	-1.589116
C	1.713701	-0.256631	0.085371
C	2.051062	-1.578788	0.006758
H	2.790532	-1.987516	0.692072
H	1.412133	-2.276913	-0.528977
C	2.531364	0.594154	1.004929
O	3.059453	0.103830	1.991659
C	2.679562	2.058885	0.729428
C	2.720815	2.928776	1.822709
C	2.821000	2.560722	-0.569368
C	2.853672	4.297378	1.619234
H	2.638953	2.513651	2.822734
C	2.979468	3.930450	-0.766202
H	2.831976	1.878799	-1.417575
C	2.979854	4.798583	0.323755
H	2.863673	4.973985	2.467763
H	3.099387	4.319008	-1.772282
H	3.085778	5.867149	0.164554
S	3.619012	-1.467128	-1.864812
O	3.618396	-2.699077	-2.666673
O	3.363323	-0.153637	-2.475083
C	5.167781	-1.373086	-0.983271
C	5.882579	-2.546423	-0.761154
C	5.560303	-0.147569	-0.453620
C	7.045079	-2.478144	-0.000020
H	5.538142	-3.479307	-1.195169
C	6.721273	-0.098926	0.311771
H	4.978669	0.747516	-0.652220
C	7.458978	-1.259621	0.536690
H	7.628176	-3.376380	0.174176
H	7.047779	0.846285	0.731961
H	8.362720	-1.214998	1.135879

Supplementary Table 26: Cartesian coordinates of **TS-5b**

M062X SCF energy: -2739.85738844 a.u.
M062X enthalpy: -2739.187695 a.u.
M062X free energy: -2739.309179 a.u.
M062X SCF energy in solution: -2740.47046907 a.u.
M062X enthalpy in solution: -2739.800776 a.u.
M062X free energy in solution: -2739.922260 a.u.
Imaginary frequency: -237.9154 cm-1

Cartesian coordinates

ATOM	X	Y	Z
O	0.145965	2.711512	-0.497958
Si	-0.686878	4.084967	-0.041299
C	-1.834345	4.455025	-1.478503
H	-2.693065	3.776379	-1.495185
H	-2.222385	5.477079	-1.407791
H	-1.308333	4.361098	-2.433839
C	-1.707702	3.901114	1.524913
H	-1.101665	3.937905	2.436059
H	-2.412180	4.740621	1.570279
H	-2.295346	2.975721	1.535119
C	0.504249	5.526305	0.150895
H	1.113750	5.653362	-0.749919
H	-0.060178	6.453347	0.304286
H	1.181891	5.410163	1.002531
C	2.902207	0.245282	-0.087655
H	2.889346	-0.097052	0.953126
H	2.460537	-0.528230	-0.723526
S	4.642810	0.307618	-0.568573
O	4.707039	0.917714	-1.895218
O	5.424065	0.845587	0.545432
C	5.065592	-1.418022	-0.733915
C	4.865963	-2.046145	-1.960050
C	5.575509	-2.095932	0.368835
C	5.175272	-3.397787	-2.076433
H	4.495127	-1.472411	-2.803857
C	5.881347	-3.447897	0.239008
H	5.745223	-1.559546	1.297025
C	5.677680	-4.094953	-0.978422
H	5.032284	-3.905038	-3.024799
H	6.285837	-3.993963	1.084879
H	5.919889	-5.148582	-1.075147
C	-0.056668	0.337335	-0.424037
H	0.390634	-0.566964	-0.006348
H	0.045697	0.296992	-1.514794

C	-1.508937	0.432557	-0.058468
C	-2.421350	0.960812	-0.927592
H	-3.431620	1.173639	-0.584108
H	-2.090505	1.429960	-1.851234
C	-2.012107	0.011302	1.286843
O	-2.938636	0.611273	1.809881
C	-1.377534	-1.142824	2.000976
C	-1.349914	-1.112247	3.398443
C	-0.893384	-2.263982	1.317341
C	-0.809625	-2.177675	4.108237
H	-1.756059	-0.243996	3.908053
C	-0.375722	-3.339922	2.034772
H	-0.954776	-2.307951	0.231686
C	-0.322342	-3.292456	3.425921
H	-0.773931	-2.145015	5.192470
H	-0.014886	-4.215626	1.505031
H	0.093141	-4.127873	3.980908
S	-3.073020	-1.051436	-2.135085
O	-3.438703	-0.759481	-3.528393
O	-2.029194	-2.035551	-1.808367
C	-4.555279	-1.499918	-1.249080
C	-5.785564	-1.089618	-1.754238
C	-4.429292	-2.147360	-0.023634
C	-6.929579	-1.363322	-1.011371
H	-5.834649	-0.586520	-2.714330
C	-5.583053	-2.406076	0.709774
H	-3.452403	-2.455543	0.336371
C	-6.826658	-2.015060	0.216967
H	-7.901741	-1.066904	-1.391085
H	-5.508686	-2.912513	1.666162
H	-7.722446	-2.218011	0.794998
C	0.717721	1.566428	0.096408
C	0.773403	1.641518	1.630183
C	2.218009	1.598112	-0.295544
C	1.953083	2.587413	1.943417
H	0.970466	0.635634	2.020446
H	-0.178370	1.960130	2.062450
C	2.760586	2.709361	0.624919
H	2.307036	1.864496	-1.352798
H	1.599794	3.567474	2.279209
H	2.567958	2.184734	2.752727
H	2.563331	3.672119	0.147503
H	3.837249	2.635803	0.787678

Supplementary Table 27: Cartesian coordinates of **3a**

M062X SCF energy: -1959.87004392 a.u.

M062X enthalpy: -1959.309930 a.u.

M062X free energy: -1959.408241 a.u.

M062X SCF energy in solution: -1960.32519728 a.u.

M062X enthalpy in solution: -1959.765083 a.u.

M062X free energy in solution: -1959.863394 a.u.

Cartesian coordinates

ATOM	X	Y	Z
C	0.341703	-0.965350	-0.655970
C	-0.656403	0.199072	-0.397022
C	-1.117805	-0.083659	1.043923
C	-1.266391	-1.613007	1.112090
C	-0.323099	-2.185179	0.015977
H	0.482640	-1.109329	-1.733062
H	-0.333720	0.258541	1.732474
H	-2.031048	0.452465	1.319481
H	-1.015089	-1.991471	2.105804
H	-2.303940	-1.903943	0.918084
H	0.425591	-2.866474	0.425496
H	-0.893702	-2.755732	-0.722197
O	-0.048657	1.446613	-0.611473
Si	0.130876	2.815784	0.334078
C	0.319915	4.210310	-0.901373
H	-0.619671	4.383012	-1.436025
H	0.598966	5.144105	-0.401752
H	1.091645	3.978073	-1.641784
C	-1.312223	3.163556	1.480217
H	-1.304609	2.528561	2.371442
H	-1.238115	4.203662	1.820625
H	-2.282042	3.044321	0.984952
C	1.705764	2.656724	1.354955
H	2.585755	2.488477	0.723919
H	1.879981	3.573931	1.929007
H	1.646647	1.829965	2.072130
C	1.703686	-0.619710	-0.059094
H	2.030763	0.363220	-0.408076
H	1.713101	-0.636547	1.036561
S	2.974806	-1.777044	-0.589689
O	2.852762	-3.014955	0.181746
O	2.962139	-1.804687	-2.051729
C	4.471672	-0.958763	-0.065537

C	5.027358	-1.288521	1.166078
C	5.034188	0.008716	-0.894046
C	6.175867	-0.619710	1.581901
H	4.570748	-2.067930	1.767775
C	6.179702	0.671863	-0.465833
H	4.586310	0.214379	-1.861585
C	6.745289	0.358987	0.769966
H	6.628846	-0.866306	2.536514
H	6.636645	1.425619	-1.098540
H	7.640593	0.877261	1.098551
C	-1.815874	0.073048	-1.413250
H	-2.234540	-0.934652	-1.344422
H	-1.374253	0.183141	-2.409092
C	-3.955782	0.967038	-0.187841
O	-4.340284	1.918431	0.468227
C	-4.549606	-0.388261	0.047667
C	-5.001033	-0.693281	1.334528
C	-4.695683	-1.325084	-0.978910
C	-5.555596	-1.939431	1.601883
H	-4.902787	0.059017	2.111123
C	-5.270972	-2.564531	-0.715015
H	-4.377792	-1.075526	-1.987800
C	-5.688009	-2.876363	0.577418
H	-5.889153	-2.181114	2.605915
H	-5.392181	-3.286739	-1.515806
H	-6.122875	-3.848966	0.785348
C	-2.874127	1.130244	-1.222705
C	-2.849012	2.286008	-1.892756
H	-2.090825	2.481166	-2.646028
H	-3.589913	3.056025	-1.698777

Supplementary Table 28: Cartesian coordinates of **3a'**

M062X SCF energy: -1959.87314402 a.u.

M062X enthalpy: -1959.312840 a.u.

M062X free energy: -1959.412258 a.u.

M062X SCF energy in solution: -1960.32441451 a.u.

M062X enthalpy in solution: -1959.764110 a.u.

M062X free energy in solution: -1959.863528 a.u.

Cartesian coordinates

ATOM	X	Y	Z
C	1.111656	-1.388307	-1.654666
C	0.347387	-0.679407	-0.525918

C	-1.020804	-0.452824	-1.210239
C	-0.676126	0.010951	-2.632184
C	0.717156	-0.614682	-2.927295
H	2.191362	-1.418351	-1.485679
H	-1.477949	-1.450383	-1.260010
H	-1.444316	-0.295958	-3.344167
H	-0.632878	1.105196	-2.676250
H	0.690465	-1.274116	-3.797874
H	1.458808	0.161571	-3.125940
O	0.184997	-1.403403	0.672472
Si	0.107016	-3.052537	0.952745
C	-0.312272	-3.157043	2.775715
H	-1.270292	-2.670581	2.983836
H	-0.384588	-4.198692	3.105699
H	0.454454	-2.665419	3.383576
C	-1.240504	-3.876636	-0.062805
H	-0.993432	-3.935741	-1.128189
H	-1.397682	-4.901826	0.292161
H	-2.191351	-3.339166	0.028603
C	1.777542	-3.858873	0.648476
H	2.573194	-3.269875	1.117825
H	1.800362	-4.862717	1.087394
H	2.015561	-3.953989	-0.414847
C	1.011282	0.658158	-0.134038
H	0.383879	1.119788	0.636500
H	1.032940	1.323295	-1.002676
C	4.871793	1.285129	0.045901
C	5.993436	1.050725	-0.756213
C	5.025684	1.946585	1.268103
C	7.255954	1.438440	-0.326684
H	5.851279	0.563803	-1.715477
C	6.289729	2.351458	1.688500
H	4.155166	2.163417	1.878246
C	7.405305	2.089314	0.897834
H	8.124176	1.240803	-0.947187
H	6.402053	2.874643	2.632705
H	8.391004	2.398862	1.231160
C	2.411586	0.504999	0.414227
C	2.662508	-0.046551	1.607793
H	3.676443	-0.198939	1.964528
H	1.848905	-0.388693	2.239438
C	3.534279	0.885018	-0.502611
O	3.364420	0.857459	-1.710064
C	-1.955844	0.434528	-0.402690

H	-1.906719	0.179164	0.662380
H	-1.758946	1.502825	-0.540512
S	-3.655147	0.143199	-0.907505
O	-3.819593	0.632163	-2.275798
O	-3.977828	-1.243413	-0.565406
C	-4.595617	1.206473	0.171538
C	-4.894683	2.498120	-0.251019
C	-4.995771	0.725012	1.414662
C	-5.606190	3.333508	0.605318
H	-4.588958	2.822330	-1.240804
C	-5.705786	1.570445	2.261778
H	-4.768296	-0.299579	1.692060
C	-6.005897	2.870625	1.857807
H	-5.855289	4.342217	0.292709
H	-6.032211	1.213117	3.232886
H	-6.562439	3.525216	2.521069
H	0.748637	-2.421345	-1.718883

Supplementary Table 29: Cartesian coordinates of **1f**

M062X SCF energy: -678.96162613 a.u.

M062X enthalpy: -678.723221 a.u.

M062X free energy: -678.782717 a.u.

M062X SCF energy in solution: -679.11804772 a.u.

M062X enthalpy in solution: -678.879643 a.u.

M062X free energy in solution: -678.939139 a.u.

Cartesian coordinates

ATOM	X	Y	Z
C	-0.098085	-0.594420	0.016424
O	-0.025100	-1.809996	-0.027017
Si	-1.859904	0.204573	-0.043784
C	-1.732691	2.042250	-0.436256
H	-2.724848	2.505764	-0.447174
H	-1.129665	2.569903	0.310263
H	-1.277386	2.212327	-1.417397
C	-2.612983	-0.063988	1.659509
H	-3.648440	0.290769	1.690229
H	-2.611769	-1.129280	1.910217
H	-2.053600	0.467331	2.436204
C	-2.822966	-0.732010	-1.353529
H	-3.886701	-0.475337	-1.328324
H	-2.446030	-0.517131	-2.358174
H	-2.722503	-1.807614	-1.179869

C	1.156455	0.254472	0.152953
H	1.180582	0.970187	-0.681564
H	1.043217	0.874396	1.056408
C	2.443445	-0.568761	0.205184
H	2.535516	-1.154885	-0.715001
H	2.357324	-1.293110	1.024004
C	3.653446	0.297928	0.394030
H	3.690300	0.876087	1.318399
C	4.645780	0.415354	-0.483381
H	4.643364	-0.144117	-1.415707
H	5.495690	1.065718	-0.302999

Supplementary Table 30: Cartesian coordinates of **TS-6a**

M062X SCF energy: -1458.94390364 a.u.

M062X enthalpy: -1458.596475 a.u.

M062X free energy: -1458.678407 a.u.

M062X SCF energy in solution: -1459.26267550 a.u.

M062X enthalpy in solution: -1458.915247 a.u.

M062X free energy in solution: -1458.997179 a.u.

Imaginary frequency: -358.1462 cm-1

Cartesian coordinates

ATOM	X	Y	Z
C	-3.162592	-0.380808	-0.178725
O	-3.077824	-1.556580	-0.482808
Si	-4.919704	0.357552	0.159192
C	-4.884986	2.227520	-0.066353
H	-5.868618	2.661189	0.142681
H	-4.165709	2.698342	0.612267
H	-4.612796	2.503437	-1.090411
C	-5.341479	-0.084392	1.939237
H	-6.350777	0.253859	2.196181
H	-5.300812	-1.168301	2.084424
H	-4.644056	0.377898	2.645353
C	-6.089418	-0.483168	-1.040668
H	-7.135362	-0.274650	-0.794960
H	-5.909861	-0.154752	-2.068955
H	-5.933118	-1.565381	-1.003190
C	-1.910760	0.472771	-0.027351
H	-1.988951	1.321533	-0.722313
H	-1.927160	0.921722	0.977929
C	-0.619162	-0.309698	-0.261996
H	-0.619900	-0.731332	-1.272726

H	-0.582936	-1.157887	0.430081
C	0.587648	0.583069	-0.080301
H	0.821688	0.865434	0.948582
C	1.015939	1.420959	-1.082678
H	0.699499	1.257697	-2.108784
H	1.742291	2.204882	-0.899823
S	2.161231	-1.111696	-0.046764
O	1.985800	-1.900632	1.182907
O	2.127853	-1.737725	-1.373765
C	3.698578	-0.215614	0.095449
C	4.216011	0.038971	1.361515
C	4.289504	0.281525	-1.062469
C	5.373149	0.805852	1.465198
H	3.726922	-0.378044	2.235974
C	5.444091	1.049569	-0.942551
H	3.857532	0.046383	-2.030029
C	5.981493	1.310998	0.317184
H	5.802284	1.005673	2.441603
H	5.928411	1.438565	-1.832255
H	6.882866	1.909329	0.404302

Supplementary Table 31: Cartesian coordinates of **TS-6b**

M062X SCF energy: -1458.94796654 a.u.
M062X enthalpy: -1458.600232 a.u.
M062X free energy: -1458.681181 a.u.
M062X SCF energy in solution: -1459.26786562 a.u.
M062X enthalpy in solution: -1458.920131 a.u.
M062X free energy in solution: -1459.001080 a.u.
Imaginary frequency: -370.9533 cm-1

Cartesian coordinates

ATOM	X	Y	Z
C	-2.508260	0.085021	0.136397
O	-2.091214	1.103811	0.658064
Si	-4.422412	-0.172061	-0.000090
C	-4.804703	-1.346960	-1.421015
H	-5.882984	-1.520646	-1.500390
H	-4.463619	-0.942698	-2.379702
H	-4.323796	-2.320035	-1.274854
C	-5.171047	1.527566	-0.256110
H	-6.260326	1.506108	-0.152320
H	-4.765651	2.221022	0.486664
H	-4.932310	1.922612	-1.248293

C	-4.971259	-0.917410	1.638567
H	-6.057286	-1.056340	1.657487
H	-4.507043	-1.893613	1.812395
H	-4.700613	-0.260520	2.470931
C	-1.539768	-0.981881	-0.359018
H	-1.823600	-1.944239	0.091862
H	-1.705889	-1.108733	-1.439499
C	-0.080499	-0.634650	-0.059047
H	0.057191	-0.545111	1.024040
H	0.120502	0.365355	-0.469740
C	0.875222	-1.631971	-0.626607
H	0.847307	-1.790454	-1.703598
C	1.843798	-2.274623	0.111930
H	1.771524	-2.273645	1.198874
H	2.390456	-3.107284	-0.324619
S	3.722014	-0.950564	0.164040
O	4.339248	-1.049111	1.495009
O	4.496009	-1.197543	-1.059880
C	2.973009	0.661903	0.018360
C	2.481315	1.275288	1.166319
C	2.750510	1.180468	-1.253219
C	1.743207	2.447383	1.032177
H	2.680745	0.835862	2.138857
C	2.017593	2.358670	-1.372169
H	3.156179	0.668019	-2.119695
C	1.507934	2.982445	-0.233882
H	1.344446	2.939647	1.912721
H	1.839398	2.787230	-2.353198
H	0.922261	3.890597	-0.332949

Supplementary Table 32: Cartesian coordinates of **14a**

M062X SCF energy: -1458.95924292 a.u.

M062X enthalpy: -1458.610484 a.u.

M062X free energy: -1458.691132 a.u.

M062X SCF energy in solution: -1459.27693951 a.u.

M062X enthalpy in solution: -1458.928181 a.u.

M062X free energy in solution: -1459.008829 a.u.

Cartesian coordinates

ATOM	X	Y	Z
C	-3.106840	-0.462736	-0.067542
O	-3.139291	-1.676193	-0.162101
Si	-4.787372	0.483407	0.095132

C	-4.549369	2.303587	-0.328621
H	-5.490273	2.852823	-0.217508
H	-3.812559	2.775116	0.330383
H	-4.210496	2.434487	-1.361559
C	-5.328048	0.282864	1.885947
H	-6.312260	0.734012	2.049705
H	-5.394016	-0.777970	2.146564
H	-4.623121	0.757482	2.576140
C	-5.987898	-0.364540	-1.069362
H	-7.014644	-0.023054	-0.904748
H	-5.731056	-0.175521	-2.116168
H	-5.951928	-1.445672	-0.905199
C	-1.776170	0.276644	-0.040741
H	-1.767708	0.988162	-0.880197
H	-1.754989	0.901318	0.865888
C	-0.573760	-0.657994	-0.094981
H	-0.625312	-1.279888	-0.994999
H	-0.592485	-1.339788	0.760536
C	0.739901	0.132809	-0.102579
H	0.841887	0.728048	0.812781
C	0.934178	0.953516	-1.316825
S	2.093104	-1.109775	0.039291
O	1.946004	-1.778380	1.331573
O	2.120396	-1.871139	-1.208494
C	3.568212	-0.109643	0.106199
C	4.039956	0.311215	1.345708
C	4.202453	0.239845	-1.082895
C	5.175113	1.115925	1.391163
H	3.532747	-0.010243	2.250031
C	5.336002	1.045036	-1.023809
H	3.815891	-0.134557	-2.025683
C	5.816663	1.483207	0.209537
H	5.563443	1.449202	2.347946
H	5.849117	1.324098	-1.938248
H	6.702830	2.108718	0.250236
H	1.438979	1.909422	-1.277159
H	0.710250	0.519796	-2.284276

Supplementary Table 33: Cartesian coordinates of **14b**

M062X SCF energy: -1458.96398855 a.u.

M062X enthalpy: -1458.614428 a.u.

M062X free energy: -1458.695455 a.u.

M062X SCF energy in solution: -1459.27894308 a.u.

M062X enthalpy in solution: -1458.929383 a.u.
M062X free energy in solution: -1459.010410 a.u.

Cartesian coordinates

ATOM	X	Y	Z
C	-3.172415	0.663779	-0.588933
O	-3.502502	1.118910	-1.669823
Si	-4.169340	-0.795109	0.195791
C	-4.192973	-0.597423	2.068560
H	-4.731913	-1.426760	2.538772
H	-3.176530	-0.589883	2.476052
H	-4.685863	0.332640	2.369661
C	-3.230408	-2.354667	-0.283674
H	-3.671758	-3.240256	0.185599
H	-3.251337	-2.500916	-1.368106
H	-2.181324	-2.294059	0.025955
C	-5.889397	-0.758215	-0.550584
H	-6.453008	-1.665387	-0.311419
H	-6.459202	0.102392	-0.186990
H	-5.815349	-0.671751	-1.638689
C	-1.925612	1.167063	0.122953
H	-2.249810	1.724325	1.014710
H	-1.363200	0.301550	0.495447
C	-1.039511	2.036219	-0.781217
H	-1.631378	2.898958	-1.112139
H	-0.797026	1.461349	-1.681598
C	0.206934	2.479139	-0.095785
H	0.127771	3.087703	0.799097
C	1.517671	1.862374	-0.387395
S	1.645684	0.232576	0.444455
O	1.532752	0.461601	1.883573
O	0.725499	-0.682157	-0.236429
C	3.308079	-0.298328	0.088993
C	4.328057	0.045517	0.971653
C	3.551552	-1.015128	-1.079148
C	5.631012	-0.337404	0.667793
H	4.089511	0.581466	1.885044
C	4.859295	-1.391107	-1.371260
H	2.723055	-1.286784	-1.725775
C	5.893750	-1.049749	-0.501419
H	6.439949	-0.086885	1.346076
H	5.070322	-1.956600	-2.272840
H	6.911573	-1.347203	-0.732967
H	2.362701	2.444899	-0.008688

H	1.660428	1.642253	-1.451356

Supplementary Table 34: Cartesian coordinates of **TS-7a**

M062X SCF energy: -1458.94780929 a.u.

M062X enthalpy: -1458.599229 a.u.

M062X free energy: -1458.674220 a.u.

M062X SCF energy in solution: -1459.26692907 a.u.

M062X enthalpy in solution: -1458.918349 a.u.

M062X free energy in solution: -1458.993340 a.u.

Imaginary frequency: -558.0129 cm-1

Cartesian coordinates

ATOM	X	Y	Z
C	-0.084919	0.256615	0.021639
C	-0.061875	-0.544400	-1.282532
C	1.287401	-1.262452	-1.310109
C	2.392368	-0.227919	-1.039780
C	1.117261	1.139397	0.083233
H	-0.135343	-0.405437	0.894850
H	-0.120799	0.157534	-2.122167
H	-0.904281	-1.239827	-1.345003
H	1.303473	-2.057943	-0.553842
H	1.479160	-1.708318	-2.290072
H	1.518794	1.390022	1.059542
H	1.174366	1.930264	-0.659591
O	2.808193	0.456711	-1.987449
Si	3.608609	-0.408166	0.462190
C	4.418636	1.259478	0.742225
H	5.418781	1.146166	1.171696
H	4.515605	1.764192	-0.224524
H	3.837345	1.904989	1.407135
C	4.872374	-1.662309	-0.153675
H	5.647907	-1.841921	0.598672
H	4.402719	-2.623808	-0.386904
H	5.356808	-1.294104	-1.063287
C	2.762000	-1.097829	1.999404
H	2.242079	-2.037194	1.781480
H	3.514754	-1.316646	2.764887
H	2.039918	-0.403249	2.440131
S	-1.565336	1.314660	0.148621
O	-1.515926	1.952563	1.462170
O	-1.645976	2.105739	-1.077483
C	-2.922796	0.159659	0.148229

C	-3.310985	-0.420426	1.352837
C	-3.557112	-0.140380	-1.054279
C	-4.357407	-1.337778	1.346315
H	-2.813946	-0.132010	2.273925
C	-4.603220	-1.058535	-1.046511
H	-3.239304	0.356919	-1.965430
C	-4.997278	-1.656694	0.149387
H	-4.679988	-1.796329	2.275201
H	-5.115217	-1.301773	-1.971636
H	-5.814984	-2.370439	0.150156

Supplementary Table 35: Cartesian coordinates of **TS-7b**

M062X SCF energy: -1458.94059880 a.u.
M062X enthalpy: -1458.592159 a.u.
M062X free energy: -1458.668180 a.u.
M062X SCF energy in solution: -1459.25827072 a.u.
M062X enthalpy in solution: -1458.909831 a.u.
M062X free energy in solution: -1458.985852 a.u.
Imaginary frequency: -568.9261 cm-1

Cartesian coordinates

ATOM	X	Y	Z
C	1.539308	2.010227	-0.335902
C	1.048112	0.695781	0.241678
C	2.324264	0.004683	-1.137749
C	2.320246	1.484152	-1.559381
H	0.713539	2.682881	-0.588469
H	2.192072	2.520306	0.376076
H	1.255955	0.475866	1.288466
H	1.774347	1.583544	-2.501943
H	3.316952	1.922601	-1.680972
O	1.852459	-0.886939	-1.866579
Si	3.556275	-0.687384	0.198615
C	2.684641	-2.104037	1.064057
H	2.148031	-2.694759	0.314440
H	3.402348	-2.761465	1.564735
H	1.963617	-1.759574	1.811887
C	4.161484	0.653085	1.374691
H	3.382316	1.018042	2.050528
H	4.973741	0.253828	1.992246
H	4.564747	1.509609	0.823134
C	5.006780	-1.328608	-0.813365
H	5.755611	-1.804520	-0.171111

H	4.658002	-2.066563	-1.541909
H	5.500055	-0.519414	-1.361754
C	-0.257241	0.136418	-0.227207
H	-0.459091	0.358915	-1.279098
H	-0.310607	-0.946528	-0.074035
S	-1.597564	0.870591	0.745310
O	-1.370832	0.501550	2.141864
O	-1.712537	2.274306	0.349119
C	-3.049435	0.011966	0.173872
C	-3.419704	-1.173195	0.803213
C	-3.764156	0.533685	-0.900321
C	-4.535692	-1.858139	0.332887
H	-2.850283	-1.529399	1.655934
C	-4.878848	-0.161263	-1.360348
H	-3.456295	1.475261	-1.343970
C	-5.258987	-1.353882	-0.747048
H	-4.845434	-2.780757	0.812432
H	-5.454160	0.230663	-2.192549
H	-6.129656	-1.891211	-1.109333

Supplementary Table 36: Cartesian coordinates of **15a**

M062X SCF energy: -1458.98293083 a.u.

M062X enthalpy: -1458.631308 a.u.

M062X free energy: -1458.705316 a.u.

M062X SCF energy in solution: -1459.30063746 a.u.

M062X enthalpy in solution: -1458.949015 a.u.

M062X free energy in solution: -1459.023023 a.u.

Cartesian coordinates

ATOM	X	Y	Z
C	0.090473	-0.269760	-0.020185
C	0.056281	0.253925	-1.459438
C	-1.409256	0.675401	-1.630748
C	-2.216811	-0.360750	-0.783672
C	-1.178984	-1.113958	0.105068
H	0.102148	0.551693	0.706437
H	0.295256	-0.576500	-2.133676
H	0.768497	1.064535	-1.634822
H	-1.539384	1.704594	-1.273313
H	-1.759547	0.634048	-2.664221
H	-1.480877	-1.268453	1.144205
H	-1.017669	-2.098443	-0.349931
O	-3.113106	-1.096259	-1.405046

Si	-3.527789	0.589656	0.367745
C	-4.535029	-0.690892	1.287130
H	-3.895960	-1.360563	1.871019
H	-5.235043	-0.209717	1.978407
H	-5.104080	-1.300370	0.580666
C	-4.579842	1.654194	-0.757655
H	-5.337911	2.195695	-0.181962
H	-3.978533	2.392880	-1.296702
H	-5.088240	1.026230	-1.494306
C	-2.519834	1.636985	1.568388
H	-1.883250	2.364024	1.053964
H	-3.201035	2.197398	2.218919
H	-1.887555	1.015808	2.211572
S	1.538218	-1.267748	0.361906
O	1.446759	-1.634558	1.774288
O	1.666339	-2.289227	-0.676773
C	2.894687	-0.125507	0.175173
C	3.270804	0.654690	1.264814
C	3.536774	-0.030812	-1.056420
C	4.311821	1.565045	1.108466
H	2.766627	0.524866	2.217436
C	4.576303	0.883783	-1.199828
H	3.231285	-0.680463	-1.870697
C	4.958111	1.680216	-0.121391
H	4.624982	2.177622	1.947484
H	5.093683	0.969108	-2.149727
H	5.771228	2.389773	-0.237649

Supplementary Table 37: Cartesian coordinates of **15b**

M062X SCF energy: -1458.95454573 a.u.

M062X enthalpy: -1458.604075 a.u.

M062X free energy: -1458.679642 a.u.

M062X SCF energy in solution: -1459.27189048 a.u.

M062X enthalpy in solution: -1458.921420 a.u.

M062X free energy in solution: -1458.996987 a.u.

Cartesian coordinates

ATOM	X	Y	Z
C	1.700870	2.560684	0.064067
C	1.016890	1.185960	0.289016
C	2.011835	0.580015	-0.770759
C	2.604802	1.999063	-1.057884
H	1.021518	3.356020	-0.254611

H	2.252213	2.903117	0.942915
H	1.110084	0.816580	1.316660
H	2.325011	2.326830	-2.062273
H	3.681750	2.140713	-0.928565
O	1.597182	-0.180747	-1.739759
Si	3.298658	-0.619508	0.127939
C	2.329106	-2.061286	0.810559
H	3.000602	-2.794293	1.271142
H	1.606857	-1.736417	1.567396
H	1.758751	-2.545279	0.013723
C	4.085573	0.399758	1.504636
H	3.344257	0.728890	2.240019
H	4.820751	-0.216470	2.034694
H	4.608605	1.281485	1.121065
C	4.565304	-1.106532	-1.162498
H	5.350157	-1.734122	-0.727730
H	4.080963	-1.662428	-1.969659
H	5.042737	-0.223447	-1.599398
C	-0.439469	1.091557	-0.137288
H	-1.035492	1.909962	0.280789
H	-0.551526	1.060492	-1.225806
S	-1.184639	-0.417163	0.517152
O	-0.686037	-1.575907	-0.215355
O	-1.051676	-0.350366	1.974517
C	-2.908341	-0.210925	0.109389
C	-3.365034	-0.678929	-1.119175
C	-3.750663	0.420450	1.019575
C	-4.705297	-0.495354	-1.446872
H	-2.679370	-1.191983	-1.786505
C	-5.088943	0.596123	0.680277
H	-3.357928	0.742947	1.978875
C	-5.561324	0.142488	-0.550479
H	-5.083387	-0.856759	-2.397507
H	-5.764810	1.079344	1.378193
H	-6.606345	0.280273	-0.809602

Supplementary Table 38: Cartesian coordinates of **TS-8a**

M062X SCF energy: -1458.98085079 a.u.

M062X enthalpy: -1458.630242 a.u.

M062X free energy: -1458.703528 a.u.

M062X SCF energy in solution: -1459.29576989 a.u.

M062X enthalpy in solution: -1458.945161 a.u.

M062X free energy in solution: -1459.018447 a.u.

Imaginary frequency: -235.5897 cm-1

Cartesian coordinates

ATOM	X	Y	Z
C	-0.116847	0.284367	-0.056674
C	-0.098885	-0.371165	-1.443085
C	1.379491	-0.744813	-1.642935
C	2.167375	0.287763	-0.800730
C	1.144678	1.147894	-0.034116
H	-0.104344	-0.462192	0.746312
H	-0.401712	0.381259	-2.179987
H	-0.779181	-1.223216	-1.517155
H	1.560786	-1.773676	-1.307600
H	1.704707	-0.676993	-2.684259
H	1.451718	1.433303	0.975438
H	0.977265	2.067887	-0.610594
O	3.267322	0.843832	-1.277838
Si	3.584978	-0.513865	0.365177
C	4.536923	0.791253	1.306030
H	3.862937	1.556966	1.703448
H	5.069001	0.344531	2.152638
H	5.258624	1.287815	0.653955
C	4.643055	-1.673845	-0.651456
H	5.224823	-2.334627	-0.000039
H	4.027088	-2.305883	-1.299591
H	5.330257	-1.109596	-1.285770
C	2.572894	-1.524394	1.614182
H	1.944189	-2.276570	1.126263
H	3.254679	-2.050121	2.293383
H	1.931503	-0.878732	2.223916
S	-1.570859	1.298902	0.249208
O	-1.472465	1.788418	1.623105
O	-1.714127	2.224762	-0.874133
C	-2.917424	0.133206	0.171364
C	-3.272636	-0.561247	1.324358
C	-3.572708	-0.065147	-1.040704
C	-4.306067	-1.490675	1.253774
H	-2.758996	-0.351383	2.257463
C	-4.604721	-0.997531	-1.097862
H	-3.283501	0.520503	-1.907719
C	-4.965673	-1.709101	0.045158
H	-4.602888	-2.037521	2.142654
H	-5.132449	-1.162669	-2.031378
H	-5.773024	-2.432890	-0.004075

Supplementary Table 39: Cartesian coordinates of **TS-8b**

M062X SCF energy: -1458.95067370 a.u.

M062X enthalpy: -1458.601067 a.u.

M062X free energy: -1458.675386 a.u.

M062X SCF energy in solution: -1459.26457270 a.u.

M062X enthalpy in solution: -1458.914966 a.u.

M062X free energy in solution: -1458.989285 a.u.

Imaginary frequency: -243.3426 cm-1

Cartesian coordinates

ATOM	X	Y	Z
C	1.588772	2.068385	0.729178
C	1.066876	1.231494	-0.462103
C	2.492459	0.663306	-0.594661
C	3.040778	1.839031	0.246074
H	1.234294	3.097570	0.786167
H	1.387283	1.571455	1.682193
H	3.393550	2.634872	-0.419557
H	3.809236	1.640528	0.999371
O	3.019899	0.089632	-1.652164
Si	3.064908	-1.141587	0.059261
C	2.148661	-2.540931	-0.782430
H	2.350018	-3.484190	-0.262830
H	1.065684	-2.382825	-0.766038
H	2.462373	-2.639239	-1.823908
C	2.591125	-1.137043	1.901999
H	1.512303	-1.081184	2.075108
H	2.952861	-2.069200	2.353810
H	3.063885	-0.305022	2.434149
C	4.929386	-1.273392	-0.019168
H	5.295816	-2.051267	0.658828
H	5.259894	-1.500306	-1.035120
H	5.391859	-0.326109	0.277620
C	-0.116637	0.298833	-0.286110
H	-0.300094	-0.290188	-1.190661
H	-0.002325	-0.369638	0.573175
S	-1.598455	1.274861	0.024466
O	-1.493245	1.819849	1.377423
O	-1.793692	2.153955	-1.126726
C	-2.902996	0.060160	0.020937
C	-3.261704	-0.547509	1.220397
C	-3.521864	-0.260374	-1.184076

C	-4.263241	-1.514065	1.206128
H	-2.774761	-0.244501	2.142017
C	-4.521375	-1.228491	-1.184575
H	-3.232899	0.260471	-2.091713
C	-4.886557	-1.854176	0.006692
H	-4.562819	-1.995649	2.131098
H	-5.020952	-1.489064	-2.111785
H	-5.668689	-2.606726	0.001227
H	0.884266	1.875802	-1.332222

Supplementary Table 40: Cartesian coordinates of **16a**

M062X SCF energy: -1459.02671650 a.u.

M062X enthalpy: -1458.675177 a.u.

M062X free energy: -1458.749820 a.u.

M062X SCF energy in solution: -1459.33735261 a.u.

M062X enthalpy in solution: -1458.985813 a.u.

M062X free energy in solution: -1459.060456 a.u.

Cartesian coordinates

ATOM	X	Y	Z
C	-0.082261	-0.237299	1.799101
C	1.002312	-1.331550	1.861703
C	1.098462	-1.917469	0.437306
C	-0.296175	-1.744342	-0.184951
H	-0.664660	-0.190238	2.728109
H	0.345798	0.764301	1.648103
H	0.688436	-2.136490	2.533178
H	1.969171	-0.970369	2.223425
H	1.448541	-2.954965	0.435993
H	-0.256616	-1.527826	-1.259255
H	-0.850266	-2.689054	-0.082777
C	-0.914194	-0.625303	0.612273
O	-1.693831	0.313359	0.004007
Si	-3.359806	0.076053	-0.123714
C	-3.974165	1.537195	-1.113203
H	-3.527141	1.549192	-2.112075
H	-5.062066	1.505455	-1.231678
H	-3.716401	2.477839	-0.616814
C	-4.089082	0.042167	1.601957
H	-3.631749	-0.756495	2.196203
H	-3.912916	0.989928	2.120416
H	-5.169306	-0.135706	1.577875
C	-3.691142	-1.543404	-1.009080

H	-3.172088	-1.580593	-1.972520
H	-3.358281	-2.399986	-0.413406
H	-4.762275	-1.670401	-1.200034
S	2.399618	-1.123159	-0.550053
O	3.668680	-1.438301	0.105867
O	2.165680	-1.474051	-1.950071
C	2.115151	0.624122	-0.358753
C	2.865548	1.324988	0.581745
C	1.102711	1.232224	-1.095413
C	2.577928	2.669087	0.800155
H	3.660614	0.814327	1.115680
C	0.813532	2.571868	-0.855826
H	0.549204	0.664091	-1.835744
C	1.547063	3.284977	0.091049
H	3.154547	3.234884	1.524588
H	0.014548	3.056152	-1.407105
H	1.317935	4.330141	0.273878

Supplementary Table 41: Cartesian coordinates of **16b**

M062X SCF energy: -1458.99257145 a.u.

M062X enthalpy: -1458.641533 a.u.

M062X free energy: -1458.718589 a.u.

M062X SCF energy in solution: -1459.30160370 a.u.

M062X enthalpy in solution: -1458.950565 a.u.

M062X free energy in solution: -1459.027621 a.u.

Cartesian coordinates

ATOM	X	Y	Z
C	-1.041144	-2.886573	-0.386798
C	-1.004890	-1.463574	0.237214
C	-2.240908	-1.172641	-0.591180
C	-2.078649	-2.402973	-1.440628
H	-0.085241	-3.248922	-0.767529
H	-1.453334	-3.624195	0.303053
H	-1.095253	-1.400516	1.327995
H	-1.628966	-2.183413	-2.419469
H	-2.959885	-3.031620	-1.603086
O	-2.601417	0.049717	-1.052300
Si	-3.354221	1.164462	-0.022609
C	-3.118862	2.819544	-0.858557
H	-3.630566	3.614752	-0.306654
H	-2.058070	3.081783	-0.921392
H	-3.521212	2.801891	-1.876021

C	-2.554506	1.123054	1.673658
H	-1.463689	1.219185	1.640441
H	-2.940134	1.946497	2.285465
H	-2.786082	0.189524	2.196980
C	-5.162566	0.699540	0.109444
H	-5.697157	1.360822	0.799424
H	-5.655646	0.754467	-0.865859
H	-5.263760	-0.326066	0.480141
C	0.175970	-0.606940	-0.224917
H	0.472351	-0.840226	-1.253257
H	-0.049762	0.462536	-0.153977
S	1.595223	-0.898164	0.835662
O	1.250844	-0.385969	2.162302
O	2.021479	-2.285087	0.653711
C	2.856350	0.159328	0.149957
C	2.960889	1.469882	0.606797
C	3.696027	-0.347162	-0.837629
C	3.929030	2.297893	0.046443
H	2.306357	1.814687	1.401242
C	4.659902	0.491089	-1.390052
H	3.600732	-1.384619	-1.142520
C	4.771777	1.809313	-0.950728
H	4.031753	3.320951	0.392984
H	5.328606	0.114246	-2.156906
H	5.526706	2.458425	-1.382975

Supplementary Table 42: Cartesian coordinates of **TS-9a**

M062X SCF energy: -2700.54889401 a.u.
M062X enthalpy: -2699.910300 a.u.
M062X free energy: -2700.025635 a.u.
M062X SCF energy in solution: -2701.16428893 a.u.
M062X enthalpy in solution: -2700.525695 a.u.
M062X free energy in solution: -2700.641030 a.u.
Imaginary frequency: -234.0747 cm-1

Cartesian coordinates

ATOM	X	Y	Z
C	1.902522	0.746749	-0.413675
C	0.692960	0.898708	0.487220
C	0.890419	0.106331	1.759855
C	2.302519	-0.497821	1.626935
C	2.504918	-0.559417	0.110866
H	1.682578	0.710728	-1.484442

H	0.790619	0.762713	2.629845
H	0.129985	-0.680831	1.859850
H	3.039977	0.184947	2.066195
H	2.415194	-1.477745	2.093263
H	1.971397	-1.423043	-0.308168
O	0.061084	2.078434	0.620561
Si	-0.378958	3.300991	-0.467936
C	-2.247601	3.325785	-0.521122
H	-2.660340	2.452965	-1.041219
H	-2.657186	3.350872	0.495082
H	-2.600120	4.221833	-1.044615
C	0.297910	4.880145	0.270830
H	1.390924	4.852939	0.324814
H	0.011581	5.748278	-0.332282
H	-0.084827	5.032878	1.284517
C	0.335537	3.023672	-2.179283
H	0.010541	2.080809	-2.630244
H	-0.017507	3.831034	-2.831540
H	1.429530	3.050945	-2.185593
S	4.188600	-0.886917	-0.420996
O	4.184181	-0.863019	-1.883318
O	4.658151	-2.059554	0.316695
C	5.147843	0.511052	0.139293
C	5.732819	0.467970	1.402283
C	5.311318	1.604042	-0.708432
C	6.480316	1.560452	1.833311
H	5.617487	-0.420557	2.015145
C	6.061988	2.689692	-0.266694
H	4.874092	1.579783	-1.702039
C	6.639051	2.667987	1.002270
H	6.947183	1.543006	2.812543
H	6.205964	3.547240	-0.915667
H	7.226186	3.515632	1.341204
H	2.592582	1.589553	-0.238087
C	-0.840386	-0.373083	-0.815043
C	-1.652088	-1.093373	0.007025
C	-2.924475	-0.494438	0.525033
H	-2.809493	0.551461	0.830107
H	-3.326391	-1.093363	1.345941
C	-1.299939	-2.423425	0.558381
O	-1.940215	-2.911596	1.477943
C	-0.056575	-3.099889	0.059555
C	0.238576	-3.245532	-1.299111
C	0.851525	-3.561967	1.016717

C	1.459282	-3.789650	-1.692564
H	-0.486986	-2.929434	-2.043334
C	2.080467	-4.081105	0.622726
H	0.588569	-3.477842	2.067793
C	2.391725	-4.180572	-0.733043
H	1.691769	-3.888621	-2.747759
H	2.808973	-4.386564	1.366607
H	3.364695	-4.551084	-1.037373
S	-4.158338	-0.496104	-0.796508
O	-4.378734	-1.874979	-1.219731
O	-3.760414	0.525577	-1.770823
C	-5.628687	0.081586	0.026729
C	-6.483529	-0.853334	0.603040
C	-5.871565	1.450118	0.099729
C	-7.612576	-0.398091	1.277479
H	-6.265958	-1.912202	0.503449
C	-7.003805	1.891678	0.778064
H	-5.192150	2.144829	-0.385189
C	-7.868234	0.969394	1.365860
H	-8.295447	-1.110046	1.729109
H	-7.215915	2.953886	0.841916
H	-8.751042	1.318847	1.891880
H	0.015673	-0.820990	-1.303659
H	-1.206732	0.560719	-1.232909

Supplementary Table 43: Cartesian coordinates of **TS-9b**

M062X SCF energy: -2700.55129857 a.u.

M062X enthalpy: -2699.912814 a.u.

M062X free energy: -2700.030295 a.u.

M062X SCF energy in solution: -2701.15926507 a.u.

M062X enthalpy in solution: -2700.520780 a.u.

M062X free energy in solution: -2700.638261 a.u.

Imaginary frequency: -265.2724 cm-1

Cartesian coordinates

ATOM	X	Y	Z
C	1.059150	1.568915	1.226424
C	0.402947	1.950754	-0.069398
C	1.436791	2.273901	-1.119480
C	2.760401	1.771931	-0.495665
C	2.396761	0.976233	0.775169
H	0.463386	0.860060	1.813101
H	1.475508	3.353367	-1.309495

H	1.216420	1.792870	-2.081592
H	3.430494	2.606645	-0.270317
H	2.256432	-0.081734	0.528018
O	-0.770219	2.611558	-0.118856
Si	-1.701797	3.267903	1.138602
C	-3.383719	3.541520	0.368011
H	-3.287782	4.033421	-0.604706
H	-3.993849	4.184698	1.011476
H	-3.934322	2.606614	0.222479
C	-0.929803	4.907458	1.622527
H	0.092906	4.778821	1.992897
H	-1.510105	5.388071	2.417863
H	-0.894987	5.591994	0.769354
C	-1.806206	2.137646	2.631968
H	-1.860803	1.072337	2.381474
H	-2.710387	2.389824	3.198737
H	-0.952193	2.273596	3.303130
H	1.217681	2.460415	1.858203
S	3.656679	0.759216	-1.689357
O	2.806424	-0.391215	-2.007671
O	4.144514	1.643308	-2.745148
C	5.050046	0.161471	-0.756741
C	6.211294	0.927404	-0.709690
C	4.928804	-1.043654	-0.070255
C	7.278431	0.473195	0.060027
H	6.274543	1.845670	-1.285404
C	6.001532	-1.482507	0.699572
H	4.016129	-1.626567	-0.154539
C	7.169833	-0.724842	0.765447
H	8.196315	1.049981	0.104968
H	5.923601	-2.418728	1.243424
H	8.005213	-1.073131	1.364709
H	3.177271	1.038311	1.536295
C	-0.315531	-0.152681	-0.903956
C	-1.134952	-0.644304	0.062083
C	-2.589315	-0.293208	0.073347
H	-3.031033	-0.436912	1.062817
H	-2.787942	0.713572	-0.304960
C	-0.649231	-1.429811	1.216592
O	-1.250632	-1.402854	2.284158
C	0.619842	-2.221062	1.099101
C	1.418758	-2.351721	2.239735
C	0.981250	-2.879600	-0.079836
C	2.582316	-3.110858	2.196214

H	1.103866	-1.856564	3.153373
C	2.136231	-3.657468	-0.115811
H	0.341606	-2.809256	-0.953987
C	2.937494	-3.771083	1.018702
H	3.203784	-3.203465	3.081638
H	2.406736	-4.175937	-1.029777
H	3.836769	-4.380050	0.986860
S	-3.440365	-1.424889	-1.057557
O	-3.138904	-0.998351	-2.423216
O	-3.155778	-2.789168	-0.618832
C	-5.160147	-1.081839	-0.739105
C	-5.791756	-0.073097	-1.460729
C	-5.819171	-1.807894	0.248618
C	-7.122733	0.219060	-1.175929
H	-5.248058	0.448876	-2.242145
C	-7.150189	-1.506974	0.522059
H	-5.293386	-2.600550	0.771866
C	-7.796519	-0.494920	-0.186227
H	-7.636323	0.996917	-1.731404
H	-7.684853	-2.065585	1.283251
H	-8.835174	-0.265512	0.030150
H	0.712849	-0.479129	-1.027926
H	-0.745060	0.409188	-1.729779

Supplementary Table 44: Cartesian coordinates of **17a**

M062X SCF energy: -2700.59026980 a.u.

M062X enthalpy: -2699.948730 a.u.

M062X free energy: -2700.064798 a.u.

M062X SCF energy in solution: -2701.19617395 a.u.

M062X enthalpy in solution: -2700.554634 a.u.

M062X free energy in solution: -2700.670702 a.u.

Cartesian coordinates

ATOM	X	Y	Z
C	1.790271	-0.203455	-1.087173
C	0.778321	0.330936	-0.052592
C	0.785671	-0.794564	0.993928
C	0.732910	-2.076223	0.157856
C	1.492609	-1.712105	-1.145292
H	1.718785	0.284778	-2.062249
H	1.735211	-0.741496	1.538891
H	-0.016354	-0.693933	1.729777
H	1.165532	-2.944149	0.660749

H	-0.295800	-2.344744	-0.105415
H	0.905568	-1.976166	-2.031766
O	1.079887	1.587534	0.498710
Si	2.554244	2.277128	0.901246
C	2.106339	3.852594	1.801222
H	1.430098	3.646051	2.636872
H	3.002125	4.340048	2.200947
H	1.606278	4.555158	1.127701
C	3.552342	1.147064	2.023245
H	3.800211	0.192801	1.545671
H	4.500651	1.628136	2.290767
H	3.012387	0.931932	2.951597
C	3.554243	2.691658	-0.636801
H	2.914582	3.138189	-1.405358
H	4.329166	3.424870	-0.385252
H	4.054366	1.821781	-1.074419
S	2.978970	-2.700390	-1.427305
O	3.516880	-2.277189	-2.719168
O	2.636491	-4.096235	-1.164660
C	4.161405	-2.197880	-0.181228
C	4.112401	-2.773244	1.086581
C	5.151708	-1.281818	-0.530554
C	5.064710	-2.399025	2.031726
H	3.365498	-3.528507	1.309613
C	6.104673	-0.923987	0.419239
H	5.176464	-0.887207	-1.541836
C	6.056490	-1.478180	1.697486
H	5.042661	-2.839314	3.023094
H	6.886093	-0.217101	0.160180
H	6.800140	-1.196267	2.436122
H	2.800920	-0.034386	-0.698814
C	-0.593817	0.492555	-0.757466
H	-0.456852	1.310168	-1.469607
H	-0.828779	-0.414528	-1.325003
C	-1.707081	0.800583	0.189755
C	-2.560871	-0.297390	0.721079
H	-3.255872	0.087805	1.471142
H	-2.010275	-1.156111	1.119634
C	-1.994982	2.143546	0.700535
O	-2.544466	2.273868	1.791652
C	-1.636698	3.347065	-0.111364
C	-1.157816	4.483137	0.541781
C	-1.881839	3.384355	-1.486179
C	-0.873557	5.634852	-0.183840

H	-1.012161	4.446279	1.616914
C	-1.615470	4.544881	-2.207613
H	-2.315074	2.516588	-1.978852
C	-1.098493	5.664612	-1.559909
H	-0.486062	6.513231	0.323410
H	-1.817739	4.576703	-3.273388
H	-0.883044	6.566159	-2.125016
S	-3.528559	-0.979339	-0.665676
O	-2.683459	-1.972242	-1.336375
O	-4.078505	0.153093	-1.408630
C	-4.864569	-1.841993	0.129971
C	-4.731391	-3.203368	0.383917
C	-6.002282	-1.125369	0.492404
C	-5.772980	-3.864113	1.029464
H	-3.837678	-3.726496	0.059397
C	-7.033709	-1.798653	1.138396
H	-6.075176	-0.069096	0.252257
C	-6.916010	-3.162141	1.407053
H	-5.694646	-4.927122	1.231506
H	-7.931765	-1.262174	1.425743
H	-7.725250	-3.682614	1.909246

Supplementary Table 45: Cartesian coordinates of **17b**

M062X SCF energy: -2700.59216076 a.u.

M062X enthalpy: -2699.950790 a.u.

M062X free energy: -2700.065595 a.u.

M062X SCF energy in solution: -2701.19455209 a.u.

M062X enthalpy in solution: -2700.553181 a.u.

M062X free energy in solution: -2700.667986 a.u.

Cartesian coordinates

ATOM	X	Y	Z
C	-0.083816	-1.131584	-2.011871
C	0.806419	-1.310525	-0.773569
C	0.012594	-2.348761	0.060012
C	-1.467937	-2.185018	-0.368037
C	-1.503848	-1.084533	-1.448178
H	0.196415	-0.266227	-2.617541
H	0.339708	-3.356665	-0.199906
H	0.160164	-2.220122	1.137889
H	-1.867452	-3.128800	-0.750993
H	-1.702532	-0.110999	-0.981173
O	2.083149	-1.728316	-1.187678

Si	3.153372	-2.847630	-0.538905
C	4.686877	-2.689301	-1.593907
H	5.072867	-1.667991	-1.587655
H	5.477029	-3.353911	-1.228039
H	4.467819	-2.963585	-2.630739
C	3.493534	-2.505501	1.272026
H	2.577893	-2.524029	1.874357
H	4.159765	-3.278185	1.673182
H	3.978779	-1.535461	1.417414
C	2.513630	-4.613314	-0.724571
H	1.991814	-4.750802	-1.678052
H	3.366606	-5.301942	-0.718833
H	1.843111	-4.923613	0.082790
C	0.953334	-0.005211	0.059207
H	1.613476	-0.259090	0.894844
H	-0.021951	0.259230	0.488479
C	1.507909	1.136559	-0.726992
C	0.607831	2.098049	-1.408474
H	1.107371	2.574791	-2.255906
H	-0.357965	1.683422	-1.708870
C	2.944849	1.383881	-0.908838
O	3.343715	1.916854	-1.939142
C	3.912242	1.023839	0.174707
C	5.200303	0.621102	-0.183503
C	3.588675	1.207001	1.522481
C	6.140129	0.332032	0.799852
H	5.448571	0.552678	-1.238219
C	4.540364	0.943840	2.504318
H	2.610391	1.597555	1.790948
C	5.808236	0.489711	2.145462
H	7.134470	-0.000953	0.519356
H	4.293401	1.098373	3.549730
H	6.543852	0.273061	2.913926
S	0.225777	3.499815	-0.256250
O	0.943602	3.240382	0.992611
O	0.416700	4.742955	-0.996622
C	-1.519783	3.342874	0.079953
C	-1.942412	2.480693	1.086308
C	-2.415527	4.092003	-0.679117
C	-3.307986	2.365636	1.336882
H	-1.222905	1.920433	1.674240
C	-3.776680	3.969445	-0.417139
H	-2.038812	4.769740	-1.438282
C	-4.219234	3.108483	0.587595

H	-3.648011	1.684536	2.109840
H	-4.490743	4.552988	-0.989118
H	-5.282297	3.018799	0.791493
H	0.046888	-2.023522	-2.635773
S	-2.496665	-1.823166	1.064715
O	-2.144085	-0.481315	1.542525
O	-2.417906	-2.970884	1.965300
C	-4.144910	-1.753800	0.392482
C	-4.924863	-2.906491	0.414343
C	-4.608791	-0.558411	-0.151184
C	-6.203956	-2.857798	-0.132904
H	-4.534587	-3.809610	0.872812
C	-5.888530	-0.524609	-0.696499
H	-3.984421	0.329514	-0.125763
C	-6.680884	-1.671752	-0.688683
H	-6.830795	-3.743206	-0.119267
H	-6.268143	0.399466	-1.120553
H	-7.679599	-1.639639	-1.112454
H	-2.281177	-1.264447	-2.193850

Supplementary Table 46: Cartesian coordinates of **TS-10a**

M062X SCF energy: -2700.56959603 a.u.

M062X enthalpy: -2699.930259 a.u.

M062X free energy: -2700.046688 a.u.

M062X SCF energy in solution: -2701.17588074 a.u.

M062X enthalpy in solution: -2700.536544 a.u.

M062X free energy in solution: -2700.652973 a.u.

Imaginary frequency: -250.4821 cm-1

Cartesian coordinates

ATOM	X	Y	Z
C	-2.114782	-0.318558	1.028083
C	-0.984584	-0.103248	0.003020
C	-1.448145	-0.975300	-1.175989
C	-1.982208	-2.253123	-0.519653
C	-2.484411	-1.801659	0.876478
H	-1.837811	-0.046697	2.049595
H	-2.260514	-0.451753	-1.690632
H	-0.651161	-1.151246	-1.903143
H	-2.766721	-2.739915	-1.103559
H	-1.192062	-2.998399	-0.390998
H	-2.048305	-2.410389	1.675988
O	-0.767095	1.240233	-0.352825

Si	-1.837530	2.494637	-0.659431
C	-0.759131	3.848893	-1.364046
H	-0.129133	4.288747	-0.584867
H	-0.102863	3.448227	-2.143822
H	-1.366530	4.647887	-1.802440
C	-3.159623	1.993979	-1.897063
H	-3.786724	1.173355	-1.531389
H	-3.824558	2.844346	-2.090201
H	-2.719406	1.688669	-2.852149
C	-2.658342	3.091263	0.924350
H	-1.932793	3.143944	1.742821
H	-3.059508	4.099999	0.772500
H	-3.487930	2.452749	1.244947
S	-4.243742	-2.108768	1.152336
O	-4.531513	-1.668019	2.516112
O	-4.523226	-3.475416	0.717824
C	-5.119997	-1.005771	0.049204
C	-5.366152	-1.399228	-1.264261
C	-5.594295	0.205113	0.550788
C	-6.081127	-0.543722	-2.099242
H	-5.038037	-2.375408	-1.606817
C	-6.314757	1.047876	-0.290863
H	-5.417491	0.454102	1.592910
C	-6.552092	0.674873	-1.613099
H	-6.284471	-0.836092	-3.124053
H	-6.694978	1.991454	0.086780
H	-7.113991	1.334210	-2.266977
H	-2.962571	0.309802	0.732321
C	0.357334	-0.591769	0.598007
H	0.529334	0.002616	1.499036
H	0.281563	-1.641863	0.903689
C	1.479830	-0.431685	-0.383775
C	1.974976	-1.494965	-1.086067
C	2.072580	0.915191	-0.685071
O	2.393815	1.187142	-1.828490
C	2.312150	1.900526	0.418210
C	2.393586	3.253103	0.075219
C	2.555962	1.504874	1.737742
C	2.663519	4.207492	1.048357
H	2.243484	3.533625	-0.962138
C	2.848881	2.462533	2.706651
H	2.561182	0.449804	2.001736
C	2.887786	3.812484	2.367437
H	2.709742	5.258321	0.779527

H	3.049095	2.150535	3.726620
H	3.104838	4.556760	3.127436
S	3.687081	-2.322211	0.401111
O	3.838027	-3.776676	0.249967
O	3.374934	-1.716756	1.704490
C	5.151514	-1.536051	-0.245507
C	5.940047	-2.236792	-1.153603
C	5.399467	-0.208591	0.090712
C	7.029771	-1.585795	-1.722923
H	5.707523	-3.270958	-1.385348
C	6.489283	0.429266	-0.494078
H	4.763997	0.304323	0.806003
C	7.299477	-0.257233	-1.396597
H	7.668531	-2.115553	-2.421876
H	6.703567	1.462753	-0.243529
H	8.146781	0.246706	-1.850413
H	1.483226	-2.465529	-1.048483
H	2.658052	-1.316296	-1.913327

Supplementary Table 47: Cartesian coordinates of **TS-10b**

M062X SCF energy: -2700.57253083 a.u.

M062X enthalpy: -2699.933178 a.u.

M062X free energy: -2700.047400 a.u.

M062X SCF energy in solution: -2701.17181321 a.u.

M062X enthalpy in solution: -2700.532460 a.u.

M062X free energy in solution: -2700.646682 a.u.

Imaginary frequency: -319.9239 cm-1

Cartesian coordinates

ATOM	X	Y	Z
C	-0.154291	-1.790840	1.809986
C	-0.815627	-1.642804	0.431766
C	0.093402	-2.521810	-0.465447
C	1.491270	-2.481340	0.203714
C	1.338386	-1.667880	1.505985
H	-0.520726	-1.063011	2.538550
H	-0.273263	-3.550636	-0.448899
H	0.110012	-2.186164	-1.507950
H	1.855558	-3.492386	0.407631
H	1.600819	-0.617435	1.331671
O	-2.154665	-2.071540	0.511135
Si	-3.126079	-2.743542	-0.685024
C	-4.878885	-2.541966	-0.074837

H	-5.230966	-1.519689	-0.241120
H	-5.553543	-3.224160	-0.603297
H	-4.950749	-2.752692	0.996648
C	-2.923596	-1.885669	-2.345229
H	-1.899505	-1.910794	-2.731369
H	-3.559540	-2.393290	-3.080443
H	-3.249179	-0.841355	-2.296128
C	-2.736645	-4.575393	-0.893024
H	-2.608319	-5.064421	0.078381
H	-3.569924	-5.070939	-1.404125
H	-1.837166	-4.757776	-1.489386
C	-0.779757	-0.177551	-0.071127
H	-1.438991	-0.119233	-0.945191
H	0.226996	0.069779	-0.423930
C	-1.176773	0.810662	0.983971
C	-0.286921	1.759015	1.428646
C	-2.510888	0.746732	1.677390
O	-2.517629	0.665096	2.893441
C	-3.785234	0.829037	0.906893
C	-4.978361	0.574172	1.592013
C	-3.819541	1.238922	-0.428910
C	-6.197241	0.708275	0.941705
H	-4.921882	0.278386	2.634336
C	-5.045919	1.365899	-1.079705
H	-2.902408	1.507992	-0.947082
C	-6.230942	1.098425	-0.398620
H	-7.122061	0.507152	1.473004
H	-5.073180	1.692596	-2.114343
H	-7.183820	1.203128	-0.908519
S	-0.380588	3.441092	-0.222562
O	-1.044098	2.790827	-1.362648
O	-0.843795	4.738389	0.288604
C	1.358568	3.581220	-0.604363
C	1.957410	2.538752	-1.305442
C	2.088193	4.644509	-0.081258
C	3.335960	2.571308	-1.497736
H	1.364105	1.719134	-1.697397
C	3.463213	4.672076	-0.297331
H	1.577962	5.430246	0.466165
C	4.083687	3.636867	-0.996930
H	3.812546	1.756541	-2.033127
H	4.051426	5.500232	0.084571
H	5.157934	3.661162	-1.151887
H	-0.389681	-2.794484	2.182616

S	2.717793	-1.806593	-0.932126
O	2.305299	-0.445322	-1.297852
O	2.953548	-2.805670	-1.971287
C	4.186969	-1.659188	0.062742
C	4.986961	-2.783986	0.245839
C	4.495224	-0.430390	0.640531
C	6.120535	-2.674649	1.045227
H	4.732289	-3.714754	-0.251960
C	5.634014	-0.334759	1.435495
H	3.861739	0.430706	0.446111
C	6.438714	-1.454273	1.639936
H	6.760072	-3.537743	1.197157
H	5.895274	0.614753	1.890903
H	7.325431	-1.373631	2.260589
H	1.985514	-2.042893	2.301523
H	-0.535340	2.369740	2.293222
H	0.771811	1.649072	1.196004

Supplementary Table 48: Cartesian coordinates of **7a**

M062X SCF energy: -1920.58424114 a.u.

M062X enthalpy: -1920.054331 a.u.

M062X free energy: -1920.149777 a.u.

M062X SCF energy in solution: -1920.58424114 a.u.

M062X enthalpy in solution: -1920.054331 a.u.

M062X free energy in solution: -1920.149777 a.u.

Cartesian coordinates

ATOM	X	Y	Z
C	-1.106100	-1.065656	-1.460423
C	0.253020	-1.062716	-0.724893
C	-0.087587	-0.362042	0.600560
C	-1.003004	0.788440	0.184130
C	-1.843418	0.203244	-0.974039
H	-0.998651	-1.089848	-2.547407
H	-0.658129	-1.054777	1.230217
H	0.797661	-0.030040	1.149404
H	-1.619765	1.153030	1.010127
H	-0.408582	1.630593	-0.176191
H	-2.028569	0.922761	-1.776579
O	0.761891	-2.370142	-0.602397
Si	0.569612	-3.497316	0.622696
C	1.528709	-4.978871	-0.006295
H	2.590900	-4.739427	-0.123251

H	1.450616	-5.821845	0.688474
H	1.148552	-5.306228	-0.978751
C	1.352499	-2.898473	2.225901
H	0.717822	-2.199651	2.777906
H	1.558077	-3.752118	2.881748
H	2.305848	-2.398636	2.020516
C	-1.234154	-3.944340	0.872734
H	-1.642629	-4.410438	-0.030877
H	-1.332101	-4.668810	1.689611
H	-1.867164	-3.082431	1.112067
C	1.289524	-0.276730	-1.557410
H	0.878594	0.701426	-1.825910
H	1.434286	-0.846487	-2.481591
C	2.900271	1.260676	-0.297736
O	1.970657	1.992398	0.002380
C	4.307717	1.725814	-0.072085
C	5.375955	1.338091	-0.886646
C	4.524382	2.651519	0.953852
C	6.648049	1.858392	-0.663899
H	5.207246	0.648019	-1.706688
C	5.797631	3.154842	1.186639
H	3.677990	2.963980	1.556717
C	6.861795	2.757656	0.377284
H	7.471416	1.562850	-1.306158
H	5.962269	3.861521	1.993711
H	7.856170	3.155680	0.554319
C	2.622260	-0.094634	-0.870743
C	3.469541	-1.110784	-0.665495
H	3.228133	-2.108566	-1.018041
H	4.398101	-0.979431	-0.118427
H	-1.644277	-1.973523	-1.165963
S	-3.483422	-0.300030	-0.414025
O	-4.190207	-0.861706	-1.563579
O	-3.310889	-1.087396	0.811139
C	-4.290436	1.225106	0.036770
C	-4.858195	2.003631	-0.968480
C	-4.344044	1.599083	1.375896
C	-5.477107	3.199835	-0.620264
H	-4.832238	1.658744	-1.997681
C	-4.969039	2.797039	1.711952
H	-3.918249	0.944563	2.129838
C	-5.527834	3.595225	0.715993
H	-5.928289	3.818688	-1.388795
H	-5.024707	3.103985	2.751128

H	-6.014380	4.528001	0.983030

Supplementary Table 49: Cartesian coordinates of **7a'**

M062X SCF energy: -1920.58267289 a.u.

M062X enthalpy: -1920.052744 a.u.

M062X free energy: -1920.148756 a.u.

M062X SCF energy in solution: -1921.02361389 a.u.

M062X enthalpy in solution: -1920.493685 a.u.

M062X free energy in solution: -1920.589697 a.u.

Cartesian coordinates

ATOM	X	Y	Z
C	-1.194407	1.853257	0.922425
C	0.216381	1.501679	0.393343
C	-0.070556	1.037396	-1.045089
C	-1.333486	0.186293	-0.907048
C	-2.176546	0.960532	0.127932
H	-1.272091	1.725272	2.006771
H	-0.287642	1.917419	-1.664451
H	0.762245	0.492776	-1.496465
H	-1.868051	0.046708	-1.849251
H	-1.053807	-0.800150	-0.525501
O	1.074464	2.616179	0.503968
Si	1.559510	3.758823	-0.610411
C	2.770419	4.822943	0.341625
H	3.645620	4.241122	0.648841
H	3.123671	5.661849	-0.267306
H	2.305757	5.231033	1.244287
C	2.432134	2.983571	-2.084938
H	1.741511	2.538965	-2.807179
H	3.020679	3.744317	-2.610353
H	3.120941	2.199374	-1.752034
C	0.108589	4.810056	-1.185087
H	-0.382579	5.290121	-0.331976
H	0.456054	5.602658	-1.857391
H	-0.647856	4.231075	-1.725421
C	0.835385	0.384316	1.259771
H	0.127299	-0.443233	1.356404
H	0.969468	0.816715	2.257255
C	2.165992	-1.428018	0.032012
O	1.162261	-1.792630	-0.558731
C	3.391957	-2.291343	0.008764
C	4.314346	-2.325495	1.058933

C	3.559624	-3.148953	-1.083412
C	5.399689	-3.195993	1.006629
H	4.169991	-1.688430	1.925081
C	4.653163	-4.002990	-1.142787
H	2.817864	-3.129717	-1.875214
C	5.575423	-4.026730	-0.096645
H	6.106022	-3.227088	1.829983
H	4.785814	-4.655786	-1.999658
H	6.426658	-4.699181	-0.138978
C	2.168228	-0.117139	0.757780
C	3.291613	0.605638	0.848492
H	3.278778	1.586642	1.313809
H	4.235345	0.252579	0.443432
H	-1.395370	2.905360	0.702276
H	-2.958346	1.559685	-0.348104
S	-3.077775	-0.123910	1.250185
O	-2.112542	-1.011813	1.901846
O	-3.970337	0.715014	2.048573
C	-4.075276	-1.116844	0.156725
C	-3.590429	-2.348730	-0.273891
C	-5.312537	-0.626905	-0.252464
C	-4.365596	-3.101349	-1.151738
H	-2.633430	-2.706852	0.092432
C	-6.077247	-1.389178	-1.130162
H	-5.669198	0.322625	0.134690
C	-5.601473	-2.620110	-1.580068
H	-4.007543	-4.065958	-1.495995
H	-7.047133	-1.028032	-1.456113
H	-6.202529	-3.212241	-2.262955

Computational methods of triplet energy

All density functional theory (DFT) calculations were carried out using the Gaussian 16 software package.[1] All geometries were optimized using the B3LYP functional[2] with a basis set of def2-TZVPP for all atoms with the SMD continuum solvation model. Frequencies were calculated for all the stationary points to confirm if each optimized structure is a local minimum on the respective potential energy surface or a transition state structure with only one imaginary frequency. [3] The 3D structures were plot using CYLview.[4]

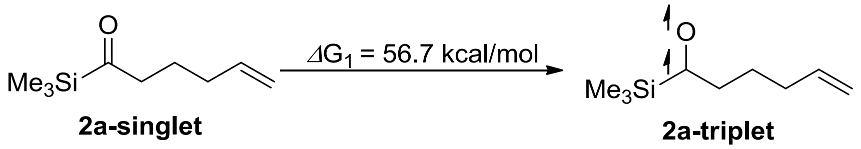

All energies were calculated at B3LYP/def2-TZVPP/SMD(acetonitrile) level of theory

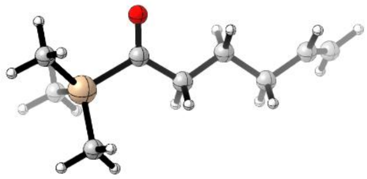

Supplementary Table 50: Cartesian coordinates of **2a-singlet**
B3LYP SCF energy in solution: -718.74555380 a.u.
B3LYP enthalpy in solution: -718.482242 a.u.
B3LYP free energy in solution: -718.544551 a.u.

Cartesian coordinates

ATOM	X	Y	Z
C	-0.589779	-0.566217	-0.003183
O	-0.475468	-1.782851	0.063850
Si	-2.398411	0.162605	0.035262
C	-3.174127	-0.427212	1.640112
H	-4.217945	-0.108026	1.700192
H	-3.152401	-1.516540	1.711524
H	-2.651382	-0.021350	2.509349
C	-3.291450	-0.565862	-1.447275
H	-4.337900	-0.249813	-1.456457
H	-2.837211	-0.240745	-2.386183
H	-3.271417	-1.657333	-1.422782
C	-2.390268	2.038292	-0.050341
H	-3.415294	2.418839	-0.028805
H	-1.857631	2.480658	0.794361
H	-1.924732	2.399854	-0.969820
C	0.619823	0.334276	-0.114799
H	0.479256	0.946848	-1.014447
H	0.561978	1.049963	0.715100
C	1.971726	-0.368417	-0.138584
H	2.003214	-1.069528	-0.976120
H	2.089368	-0.966869	0.767616
C	3.142574	0.618972	-0.252462
H	2.998183	1.225047	-1.154242
H	3.133650	1.306125	0.597772
C	4.472581	-0.066683	-0.335119
H	4.603602	-0.741698	-1.178106
C	5.469929	0.077669	0.532023
H	5.386052	0.736834	1.389762
H	6.406051	-0.454840	0.414589

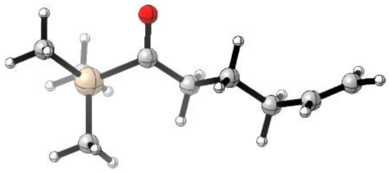

Supplementary Table 51: Cartesian coordinates of **2a-triplet**
B3LYP SCF energy in solution: -718.65230204 a.u.
B3LYP enthalpy in solution: -718.390455 a.u.
B3LYP free energy in solution: -718.454209 a.u.

Cartesian coordinates

ATOM	X	Y	Z
C	-0.590724	-0.645944	-0.064171
O	-0.650105	-1.913765	0.066810
Si	-2.350232	0.177126	-0.044350
C	-3.101056	0.069818	1.673963
H	-4.105165	0.504882	1.675059
H	-3.186267	-0.968066	2.002860
H	-2.500846	0.610911	2.408420
C	-3.405953	-0.764742	-1.271227
H	-4.399735	-0.312017	-1.330302
H	-2.970067	-0.749866	-2.272138
H	-3.530371	-1.806775	-0.969967
C	-2.117832	1.960928	-0.569382
H	-3.105960	2.410849	-0.706426
H	-1.588301	2.547663	0.182653
H	-1.579721	2.042382	-1.515235
C	0.609470	0.141538	0.467797
H	0.407089	1.205504	0.357128
H	0.715812	-0.064862	1.540745
C	1.913308	-0.220022	-0.255393
H	1.798788	-0.025581	-1.324735
H	2.109038	-1.288880	-0.144761
C	3.107381	0.579177	0.289014
H	2.879627	1.647130	0.196138
H	3.239615	0.370802	1.353648
C	4.378813	0.284703	-0.447741
H	4.369570	0.506695	-1.512479
C	5.481102	-0.225005	0.093115
H	5.536822	-0.465169	1.149666
H	6.367102	-0.417955	-0.499687

7. Spectroscopic Data

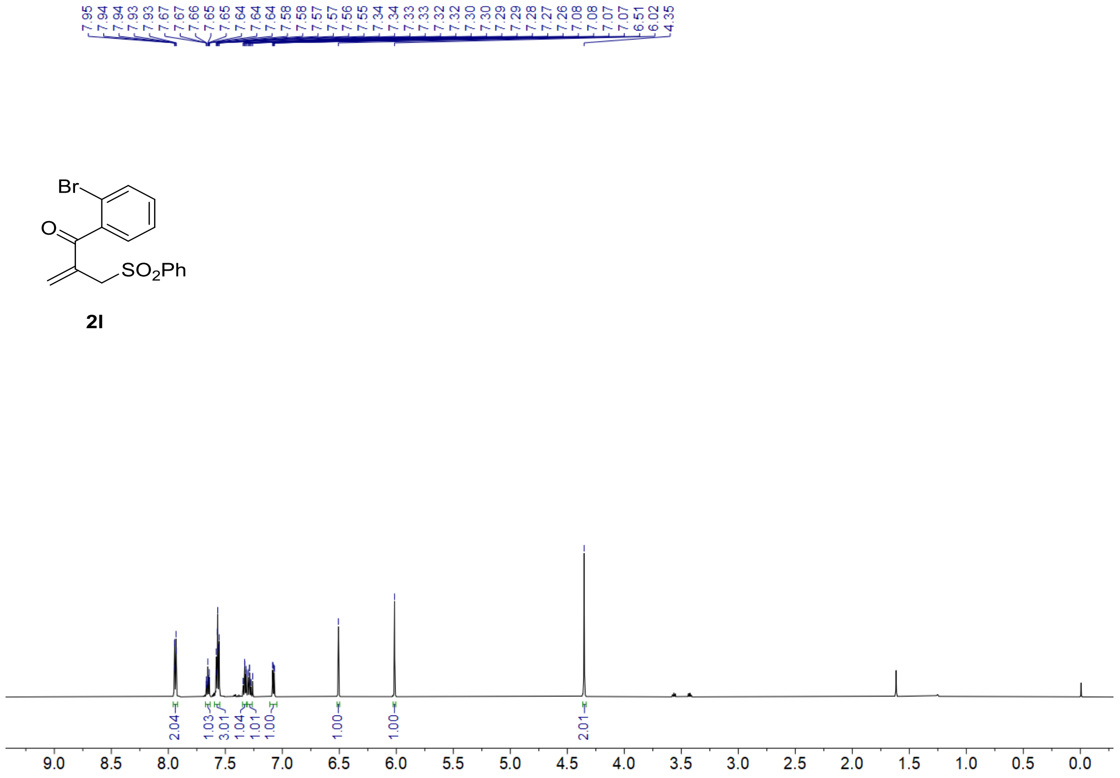

Supplementary Figure 19: ^{1}H NMR of **2l** (600 MHz, $CDCl_3$, 25 ºC)

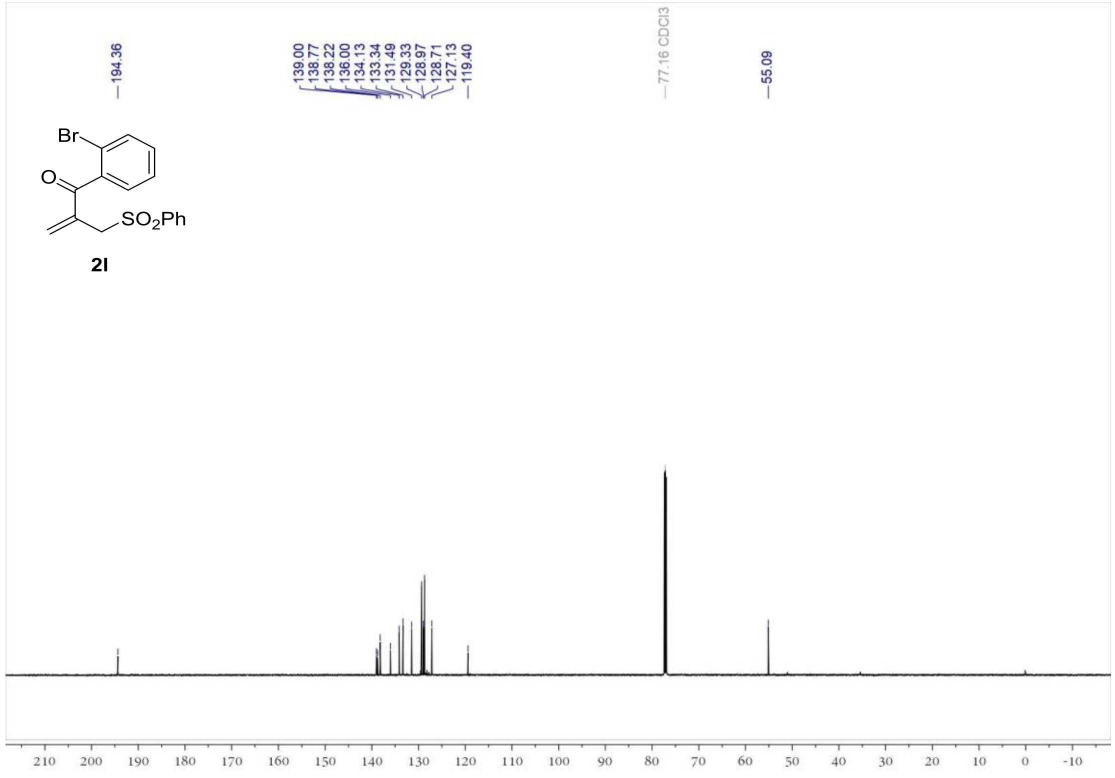

Supplementary Figure 20: ^{13}C NMR of **2l** (151 MHz, $CDCl_3$, 25 ºC)

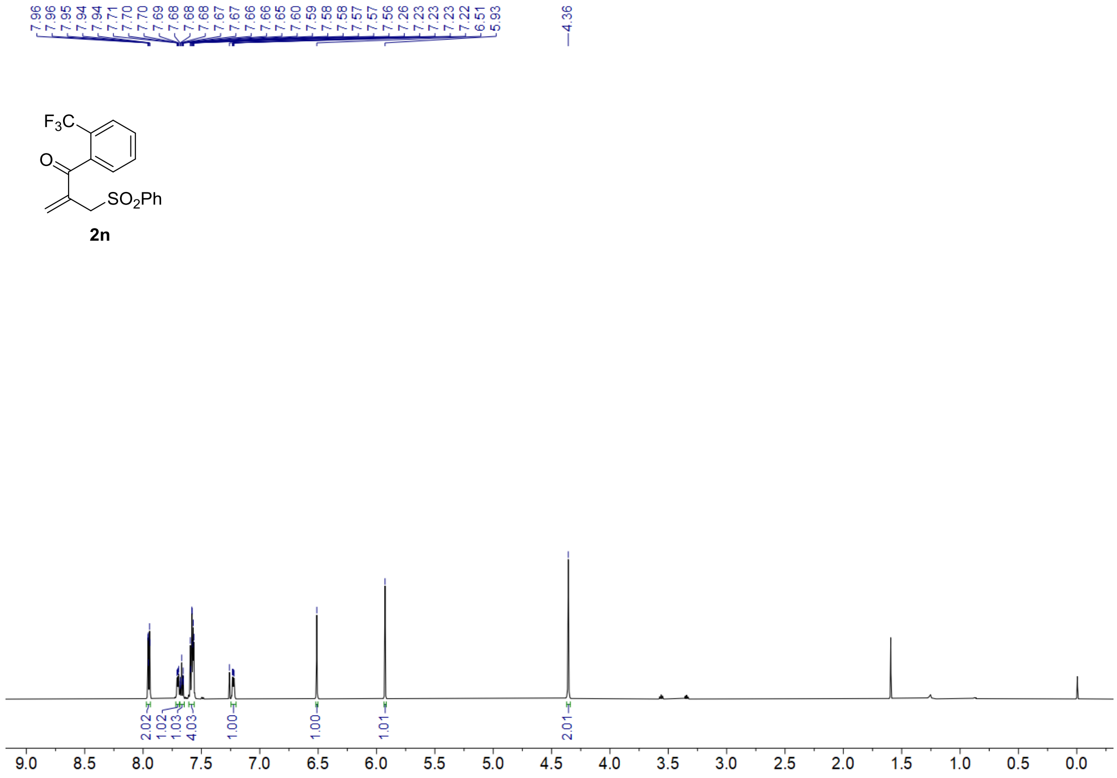

Supplementary Figure 21: ^{1}H NMR of **2n** (600 MHz, $CDCl_3$, 25 ºC)

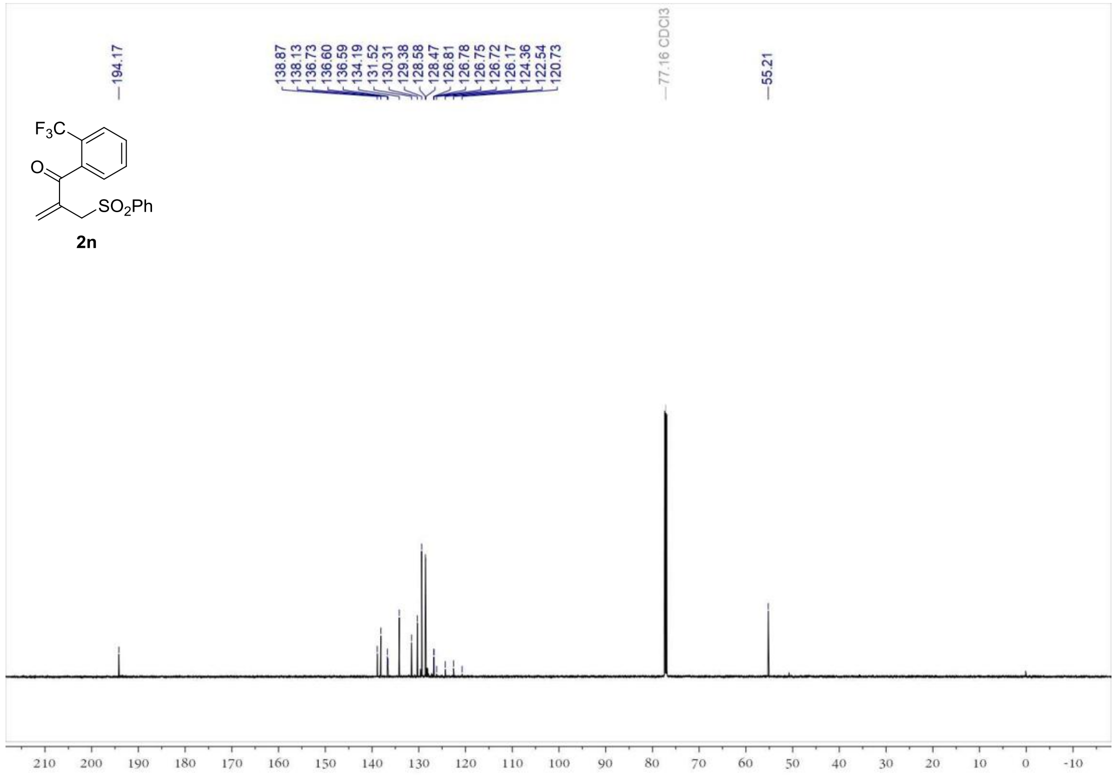

Supplementary Figure 22: ^{13}C NMR of **2n** (151 MHz, $CDCl_3$, 25 ºC)

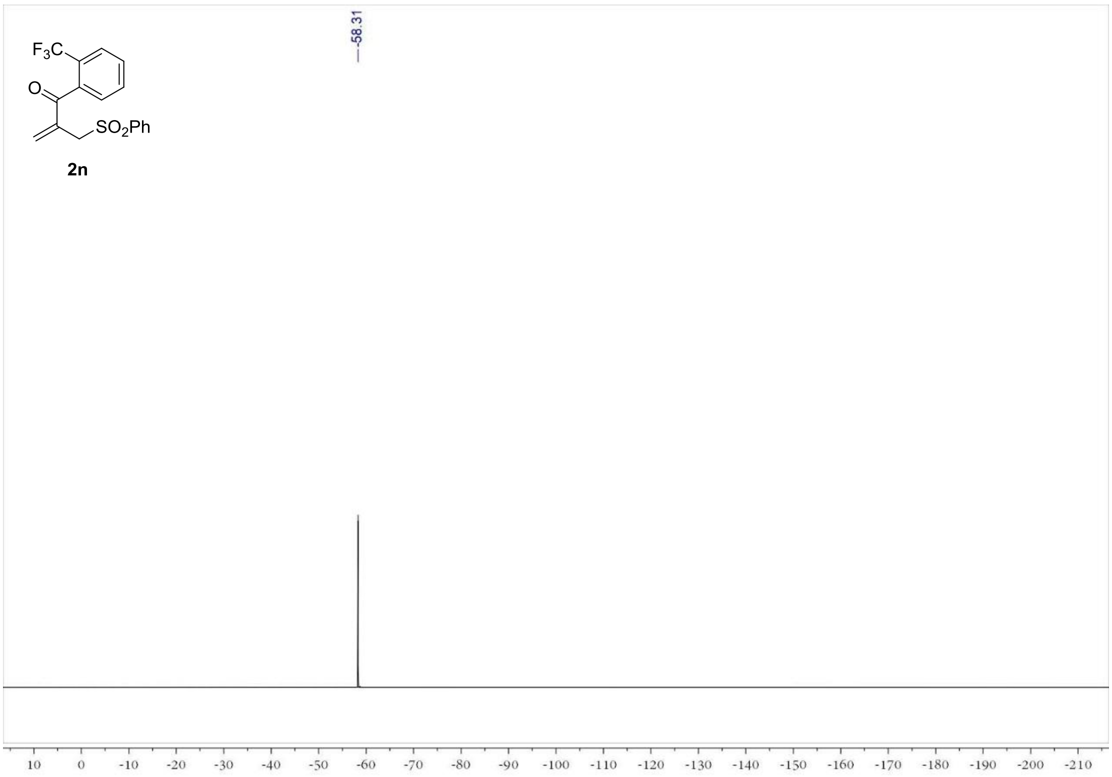

Supplementary Figure 23: ^{19}F NMR of **2n** (565 MHz, CDCl3, 25 ºC)

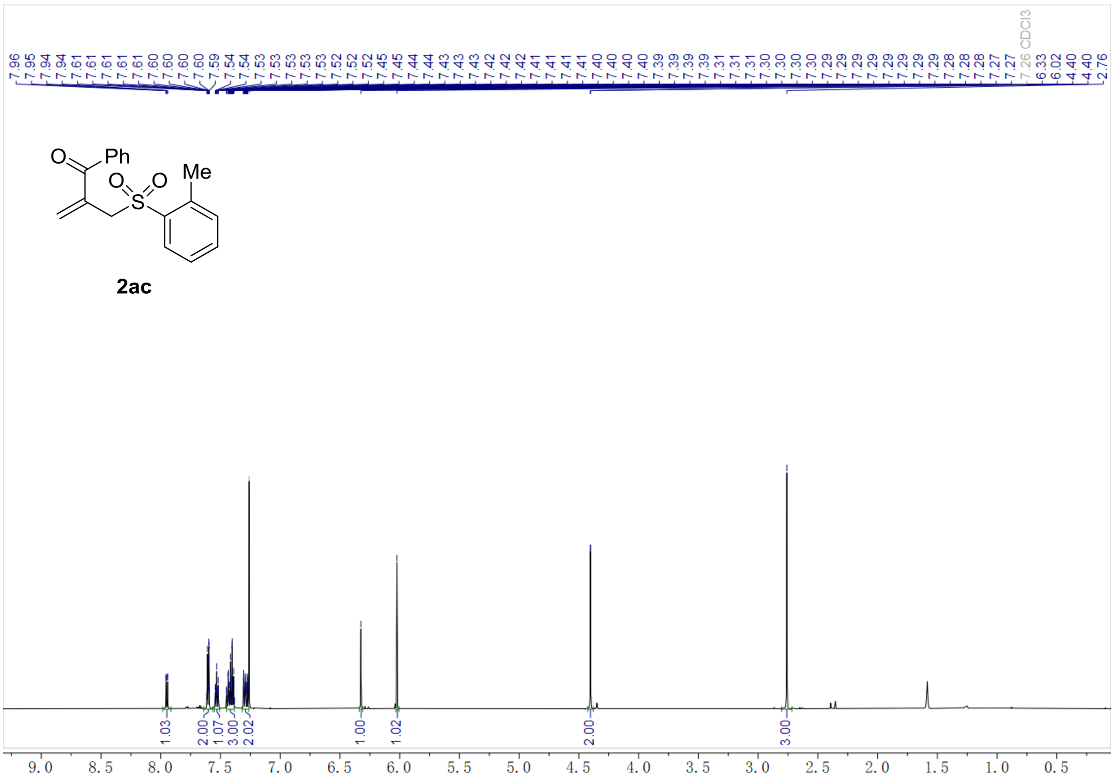

Supplementary Figure 24: ^{1}H NMR of **2ac** (600 MHz, $CDCl_3$, 25 ºC)

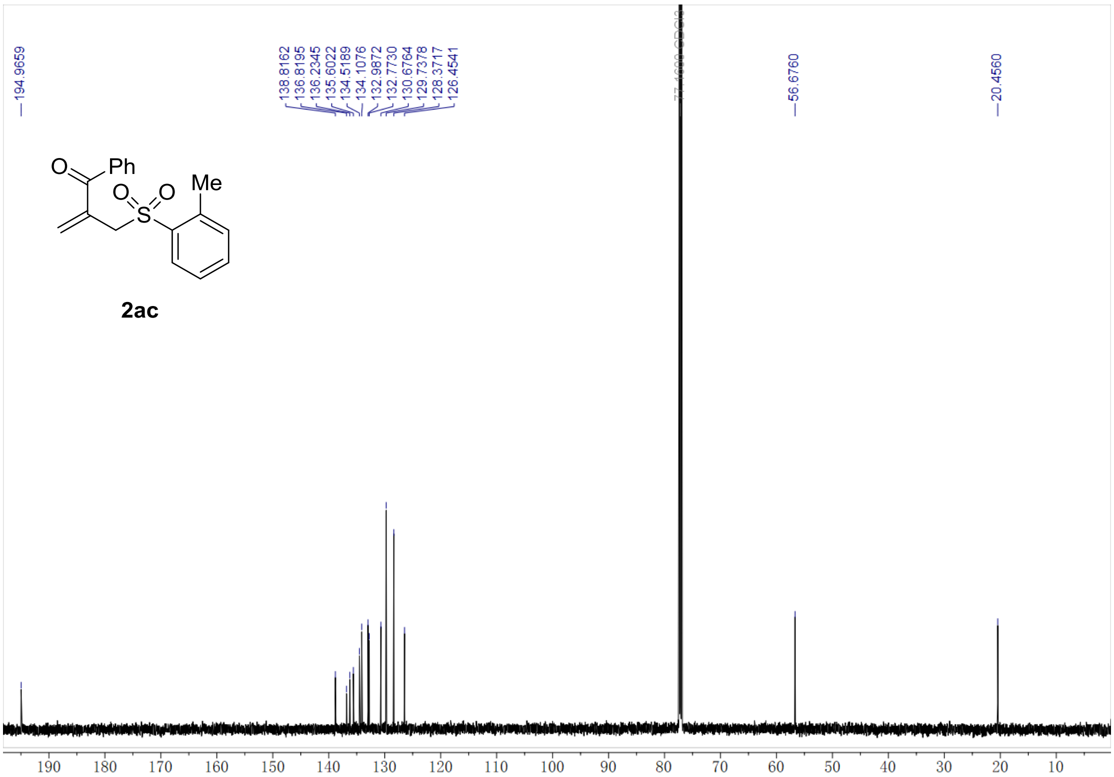

2ac

Supplementary Figure 25: ^{13}C NMR of **2ac** (151 MHz, $CDCl_3$, 25 ºC)

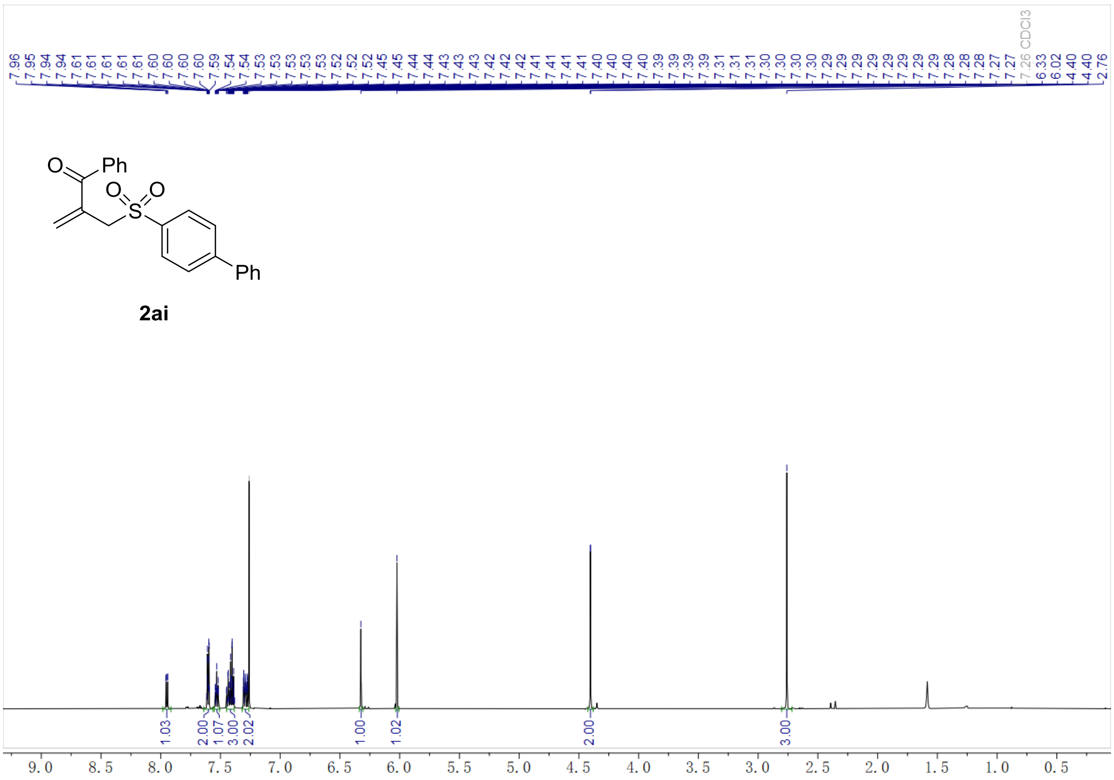

2ai

Supplementary Figure 26: ^{1}H NMR of **2ai** (600 MHz, $CDCl_3$, 25 ºC)

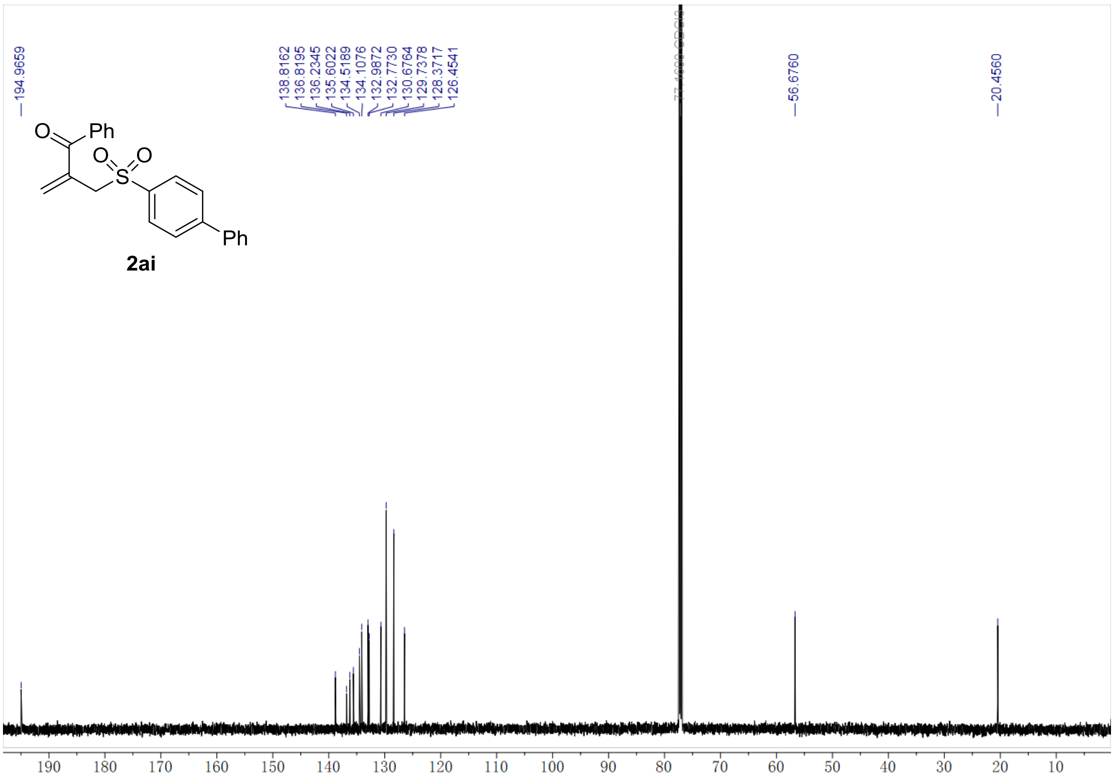

Supplementary Figure 27: ^{13}C NMR of **2ai** (101 MHz, $CDCl_3$, 25 ºC)

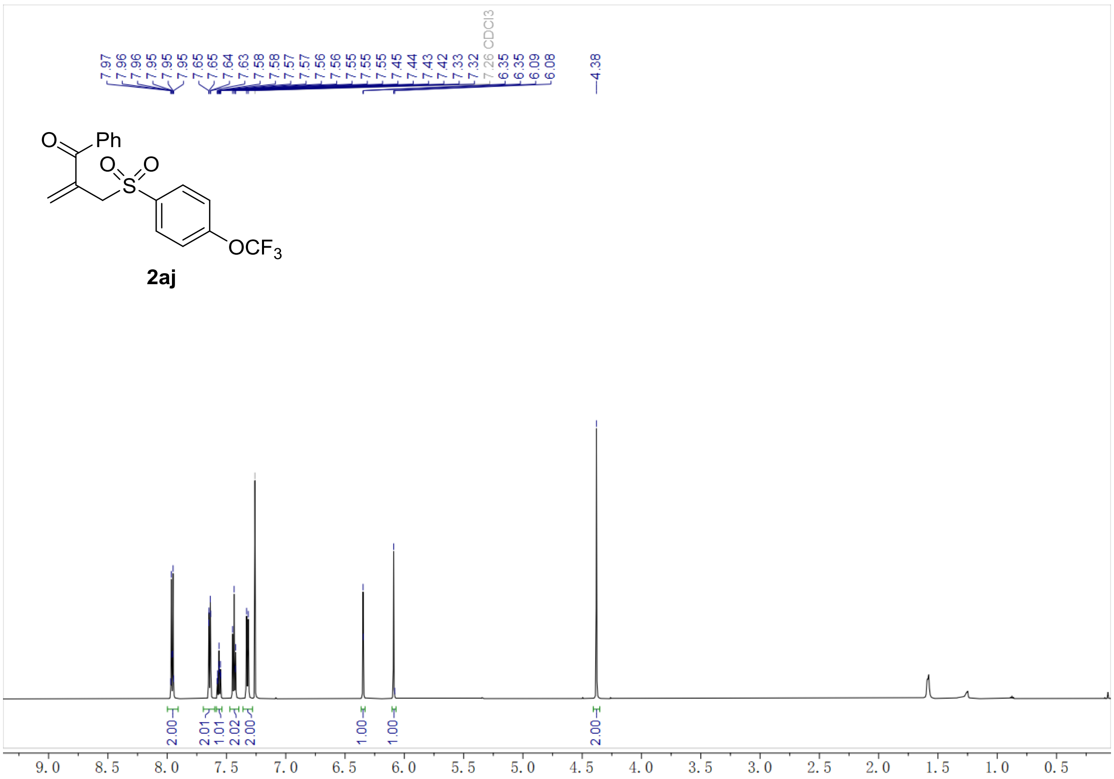

Supplementary Figure 28: ^{1}H NMR of **2aj** (600 MHz, $CDCl_3$, 25 ºC)

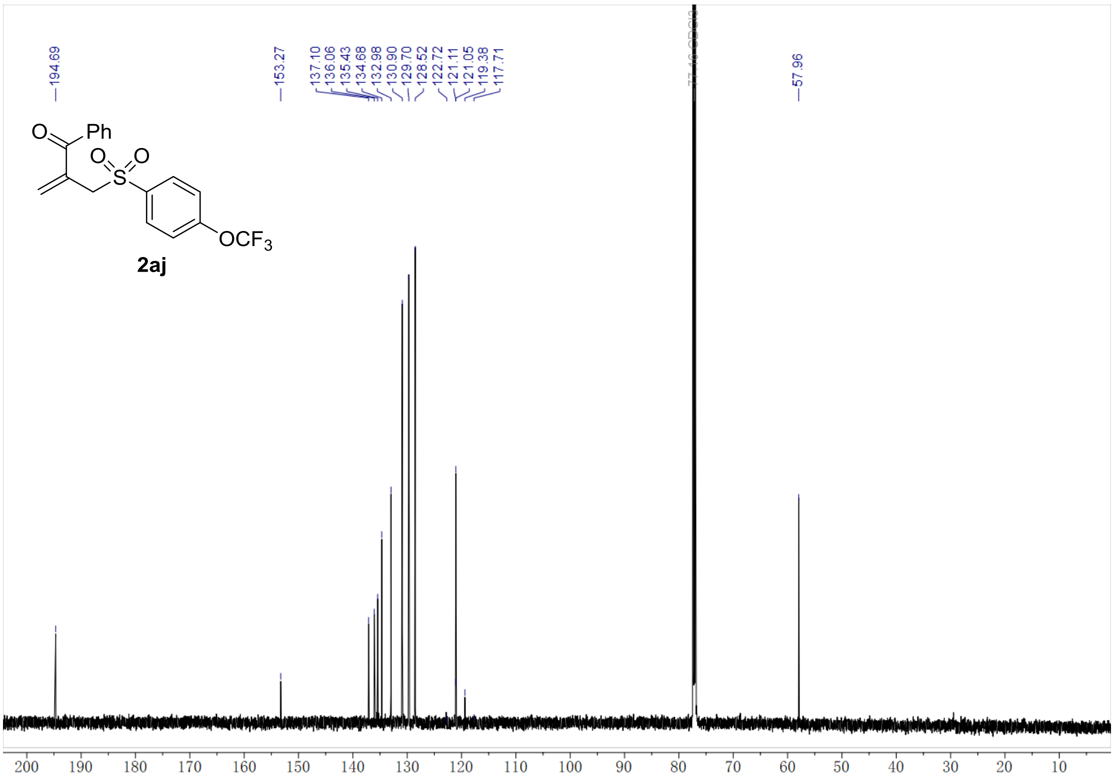

Supplementary Figure 29: ^{13}C NMR of **2aj** (151 MHz, $CDCl_3$, 25 ºC)

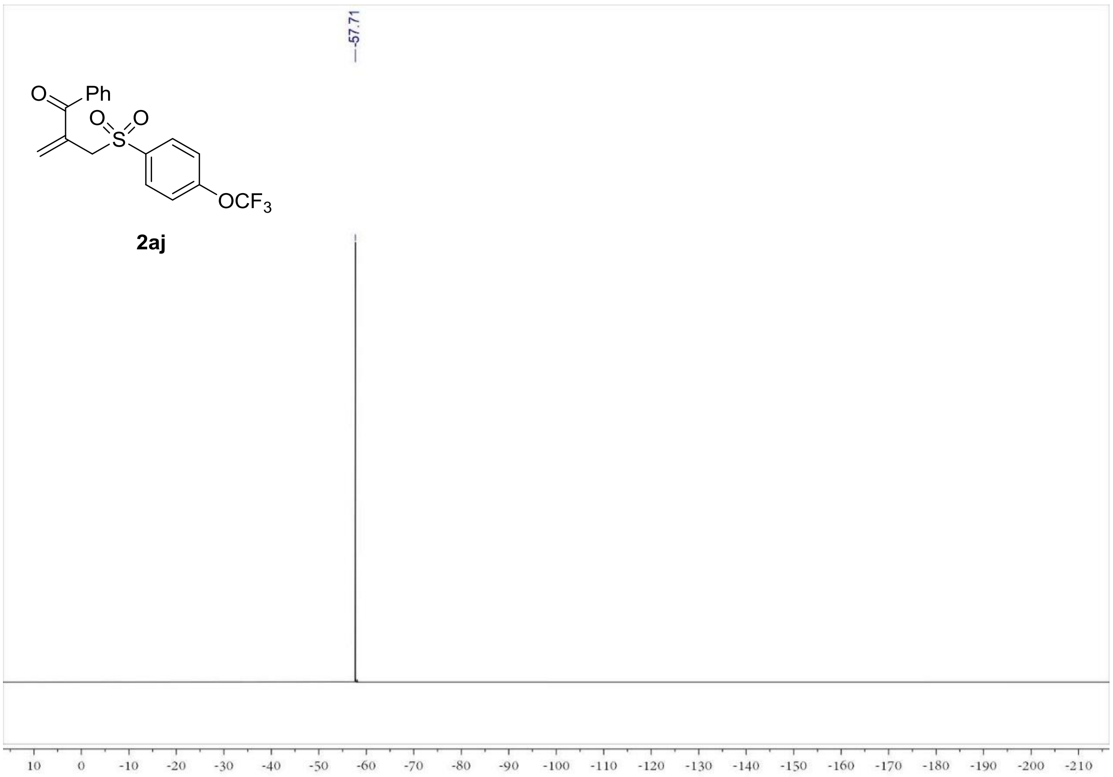

Supplementary Figure 30: ^{19}F NMR of **2aj** (565 MHz, $CDCl_3$, 25 ºC)

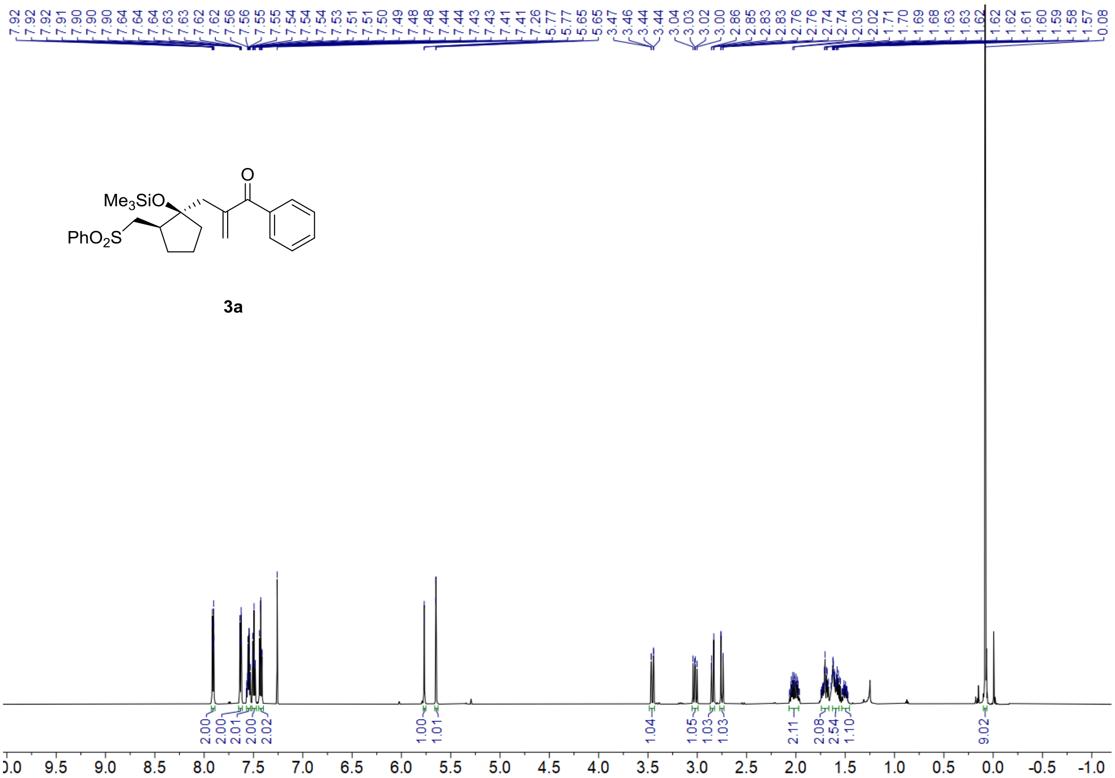

Supplementary Figure 31: ^{1}H NMR of **3a** (600 MHz, $CDCl_3$, 25 ºC)

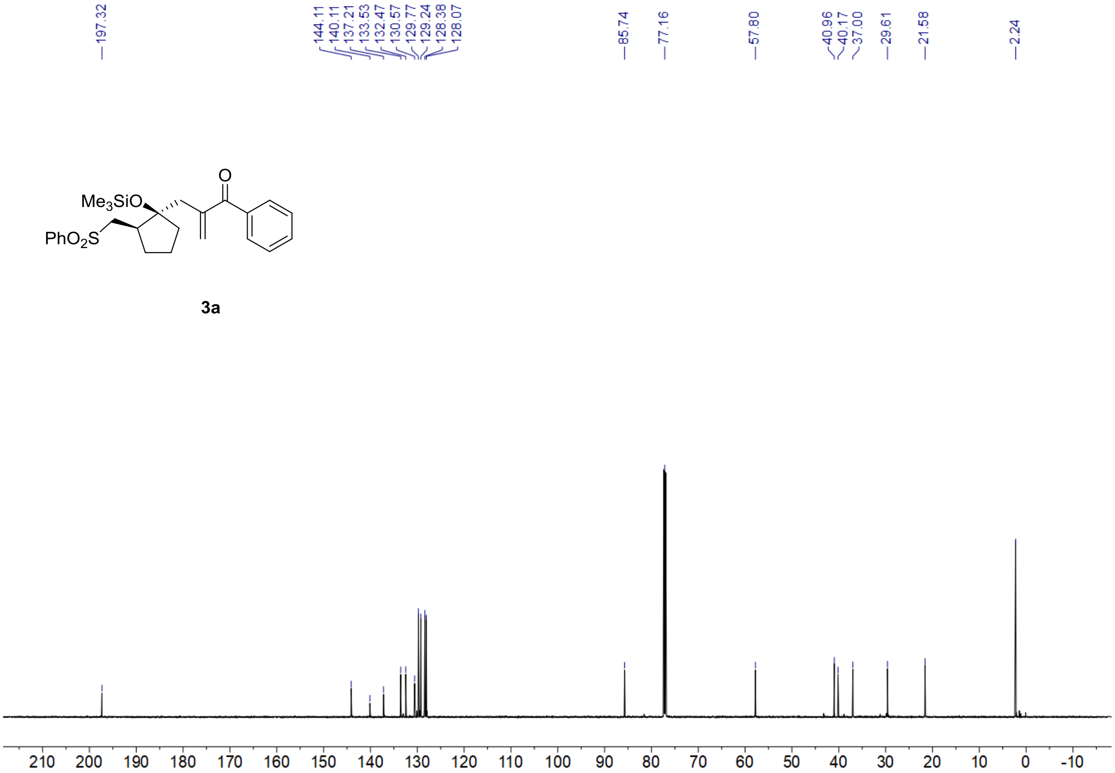

Supplementary Figure 32: ^{13}C NMR of **3a** (151 MHz, $CDCl_3$, 25 ºC)

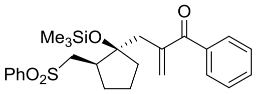

3a

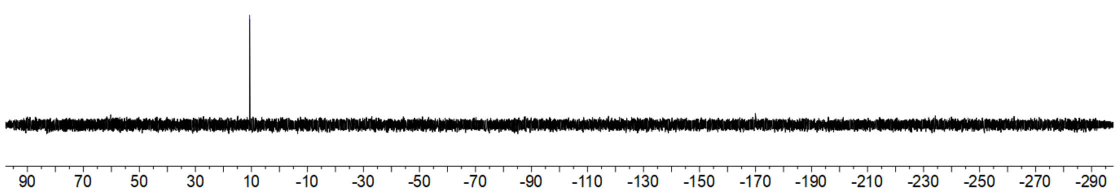

Supplementary Figure 33: ^{29}Si NMR of **3a** (119 MHz, $CDCl_3$, 25 ºC)

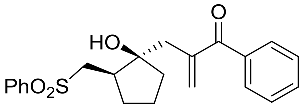

3a'

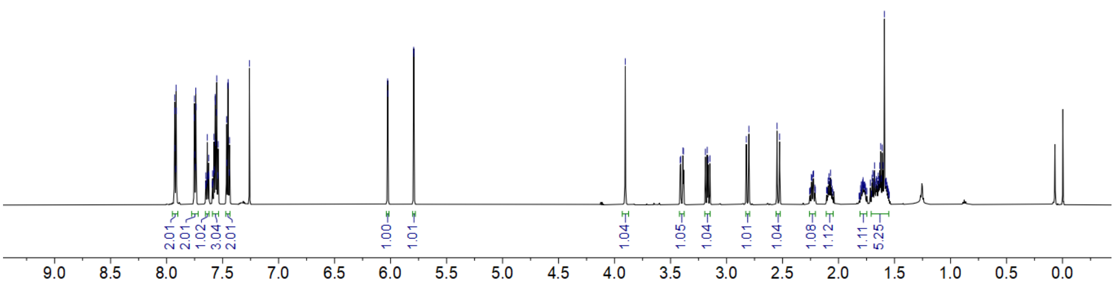

Supplementary Figure 34: ^{1}H NMR of **3a'** (600 MHz, $CDCl_3$, 25 ºC)

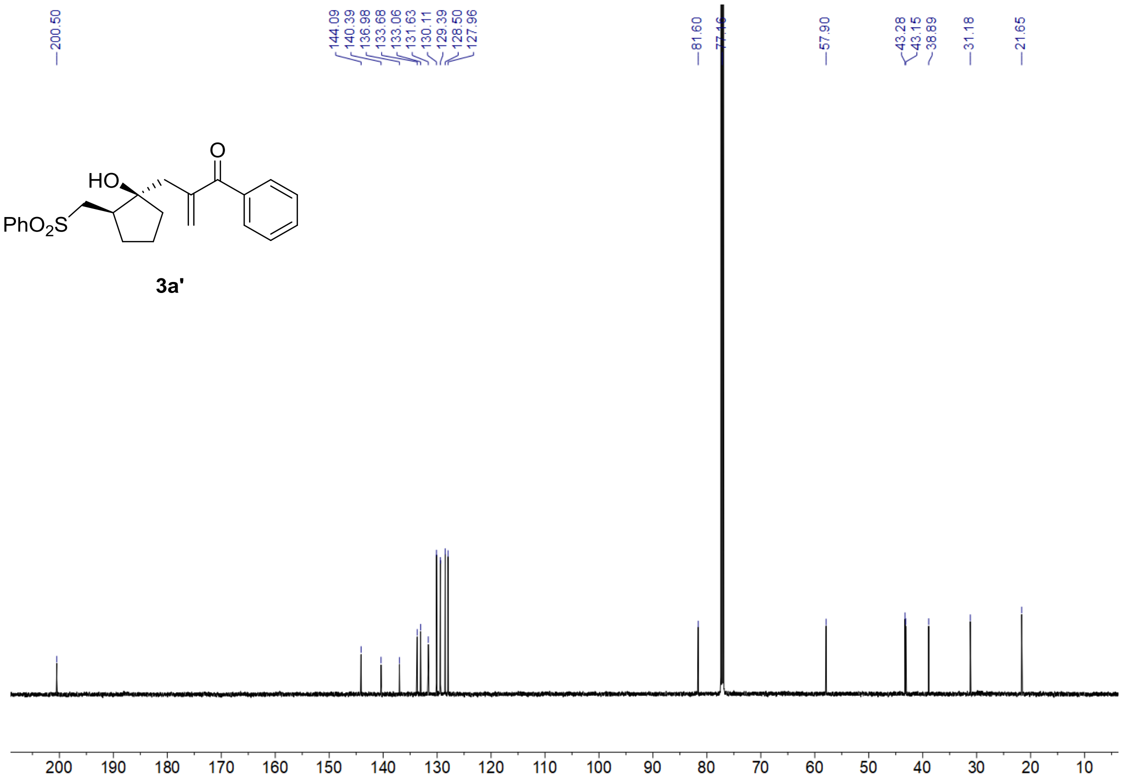

Supplementary Figure 35: ^{13}C NMR of **3a**‘ (151 MHz, CDCl$_3$, 25 ºC)

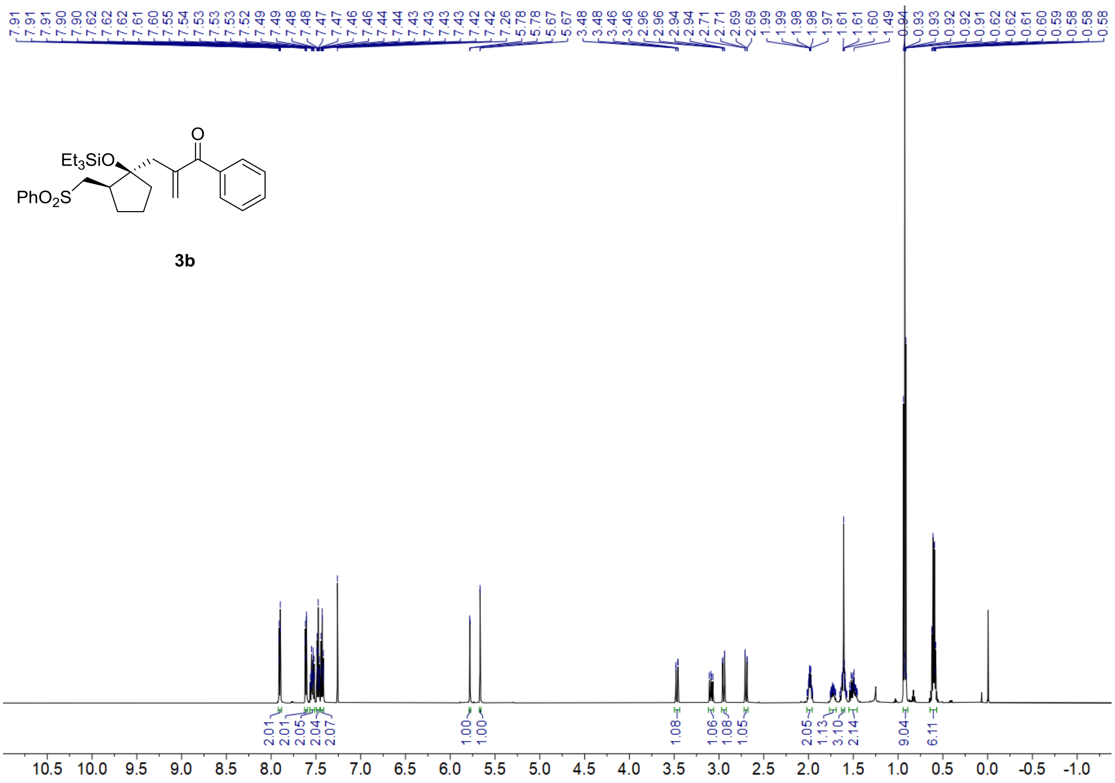

Supplementary Figure 36: ^{1}H NMR of **3b** (600 MHz, CDCl$_3$, 25 ºC)

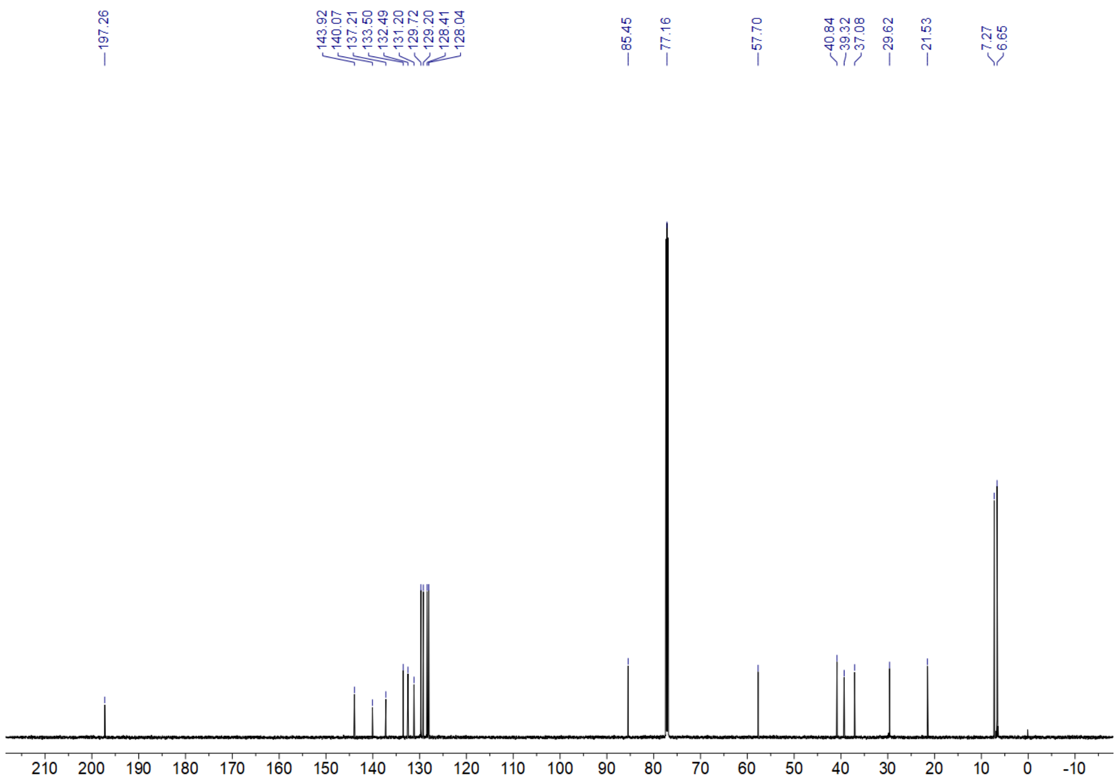

Supplementary Figure 37: ^{13}C NMR of **3b** (151 MHz, $CDCl_3$, 25 ºC)

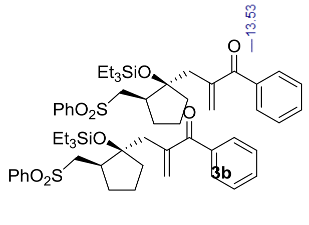

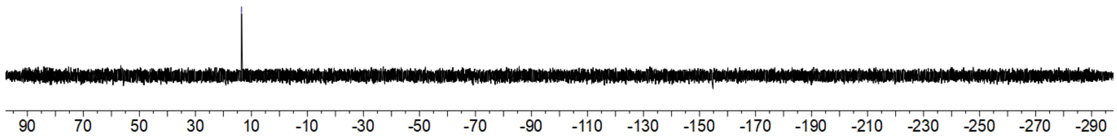

Supplementary Figure 38: ^{29}Si NMR of **3b** (119 MHz, $CDCl_3$, 25 ºC)

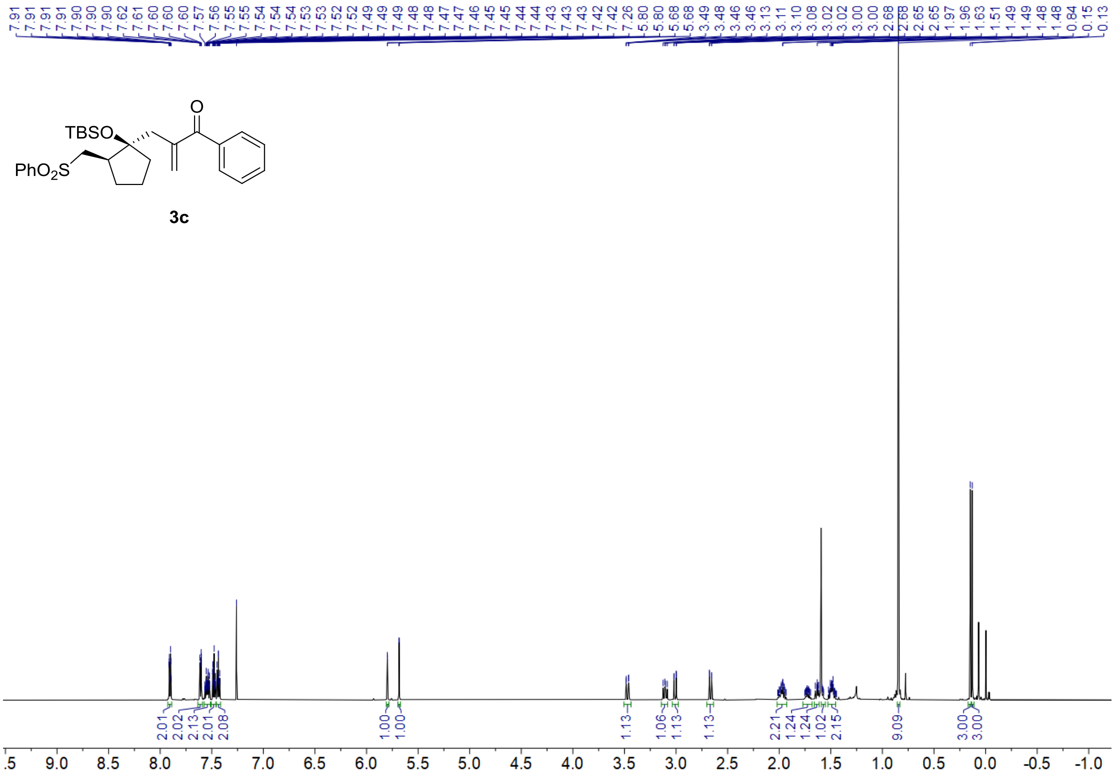

Supplementary Figure 39: ^{1}H NMR of **3c** (600 MHz, $CDCl_3$, 25 ºC)

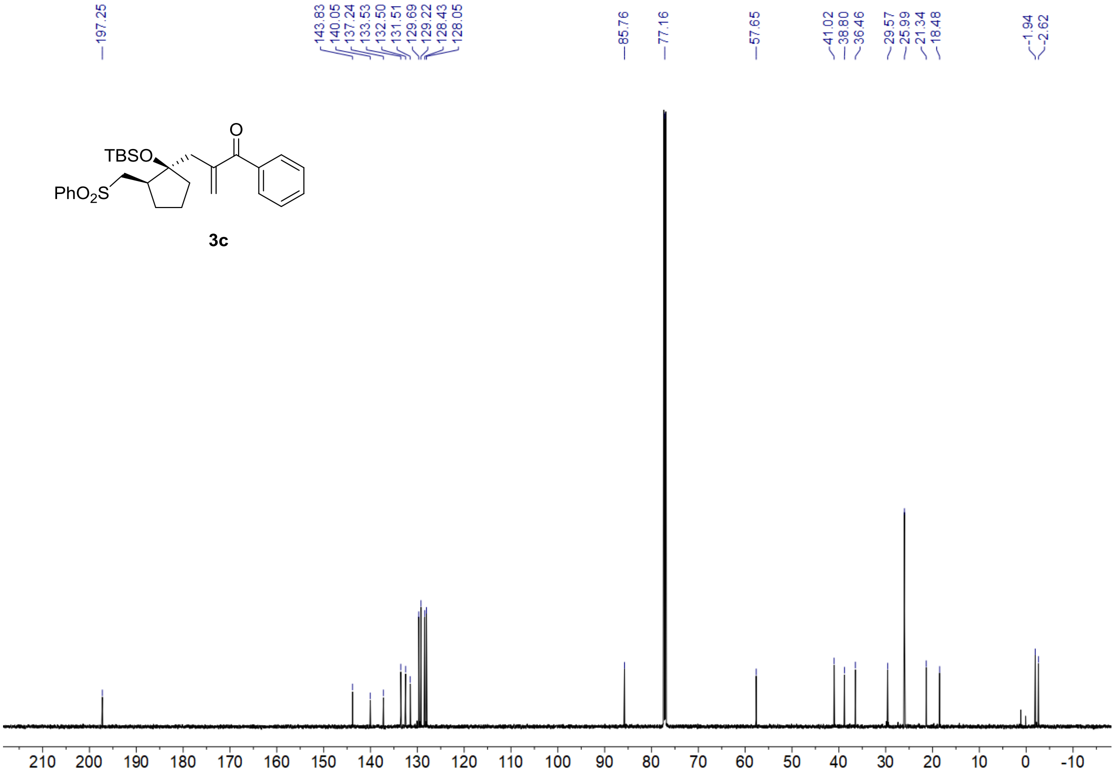

Supplementary Figure 40: ^{13}C NMR of **3c** (151 MHz, $CDCl_3$, 25 ºC)

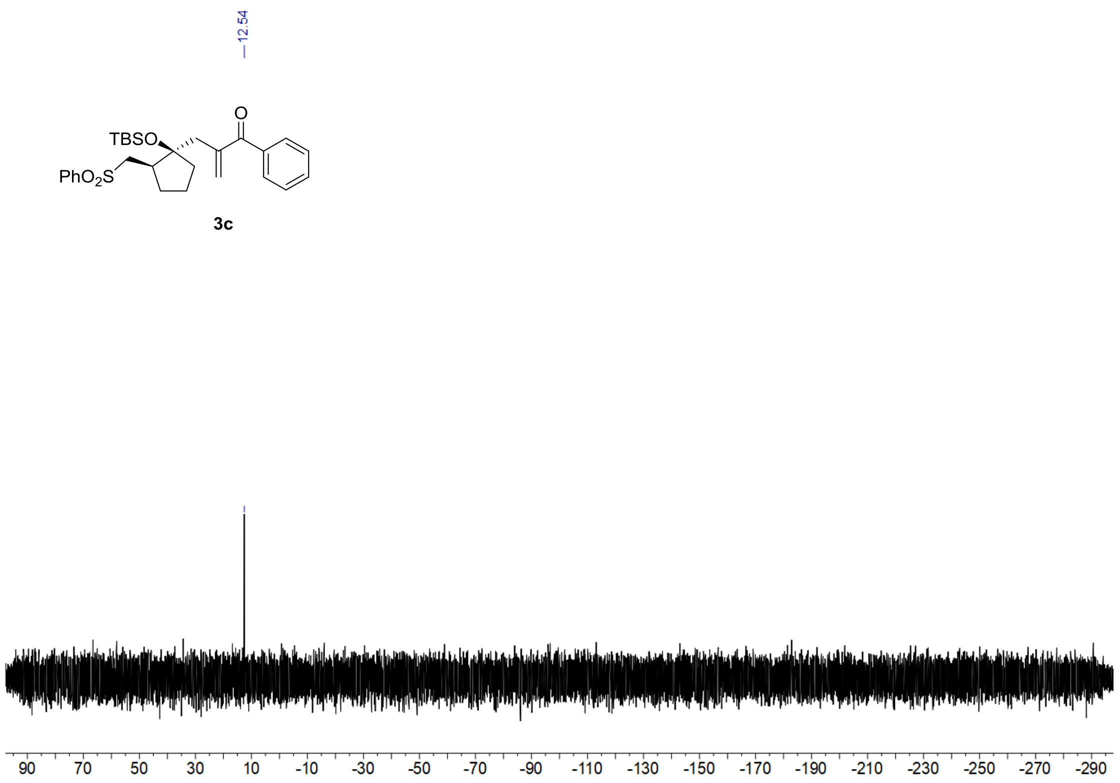

Supplementary Figure 41: ^{29}Si NMR of **3c** (119 MHz, $CDCl_3$, 25 ºC)

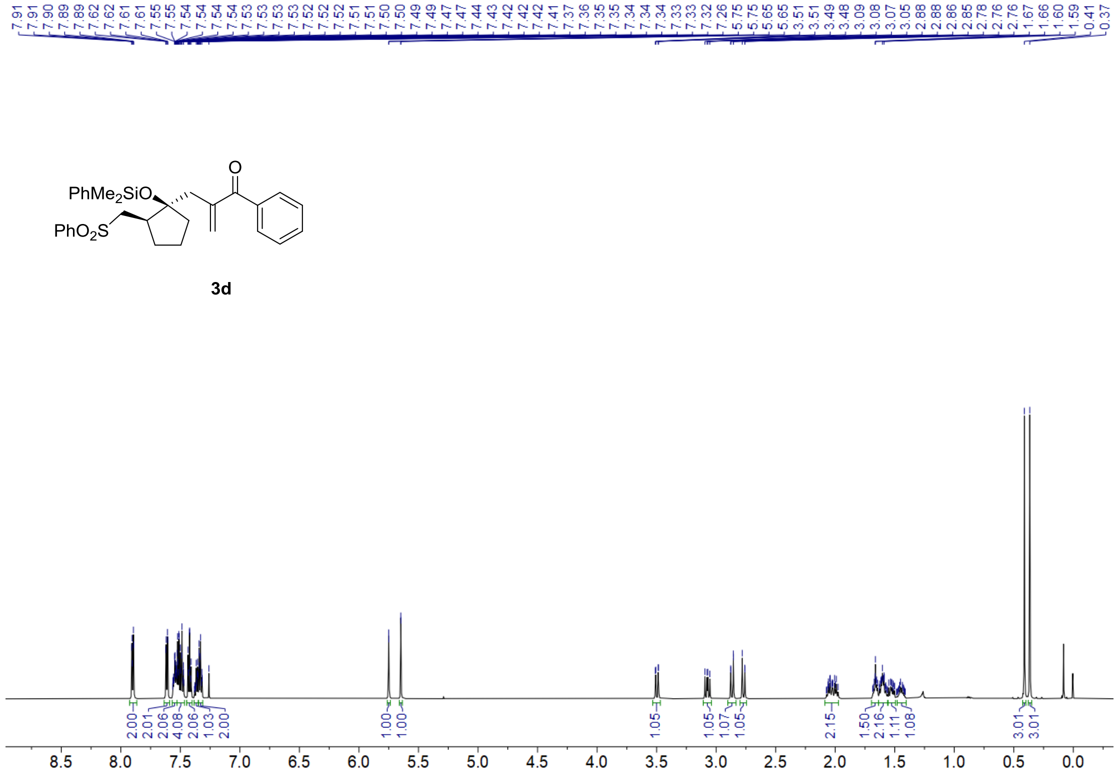

Supplementary Figure 42: ^{1}H NMR of **3d** (600 MHz, $CDCl_3$, 25 ºC)

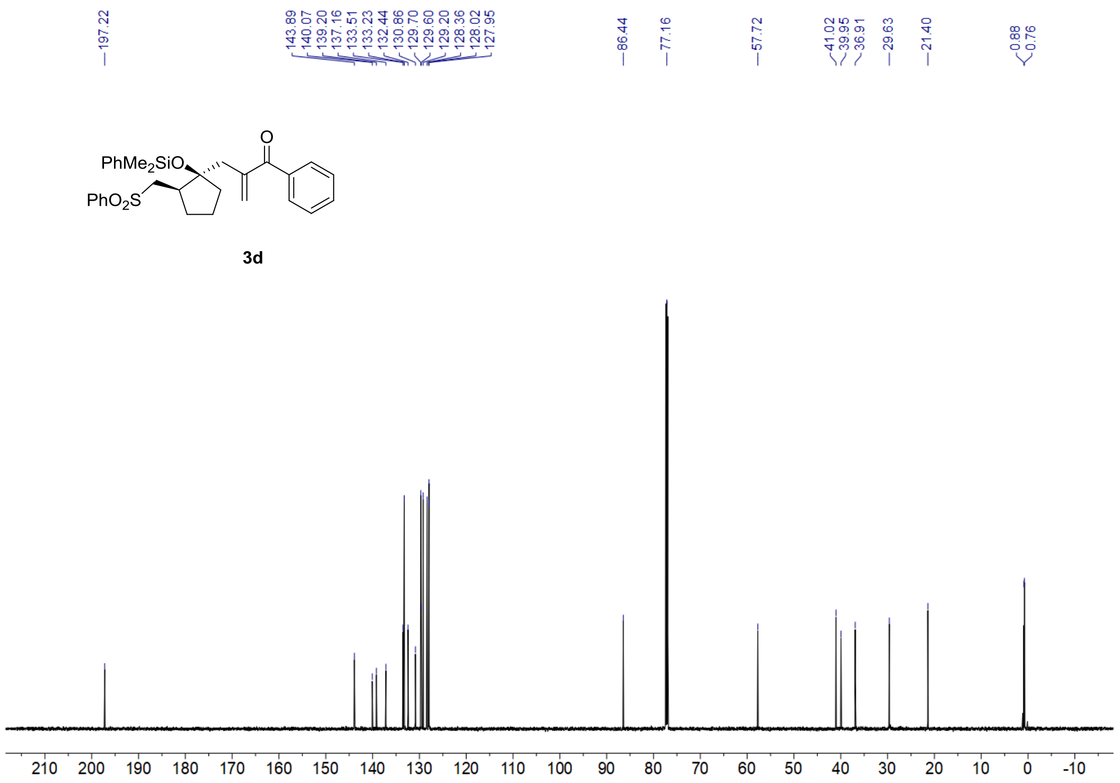

Supplementary Figure 43: ^{13}C NMR of **3d** (151 MHz, $CDCl_3$, 25 ºC)

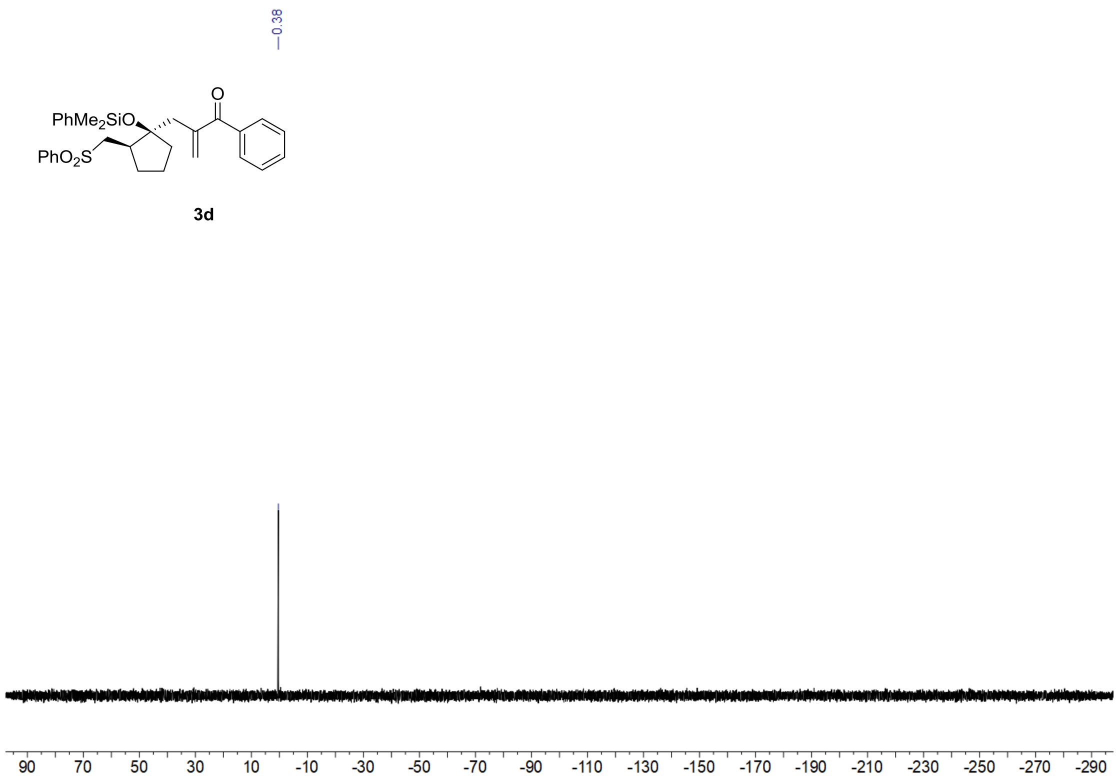

Supplementary Figure 44: ^{29}Si NMR of **3d** (119 MHz, $CDCl_3$, 25 ºC)

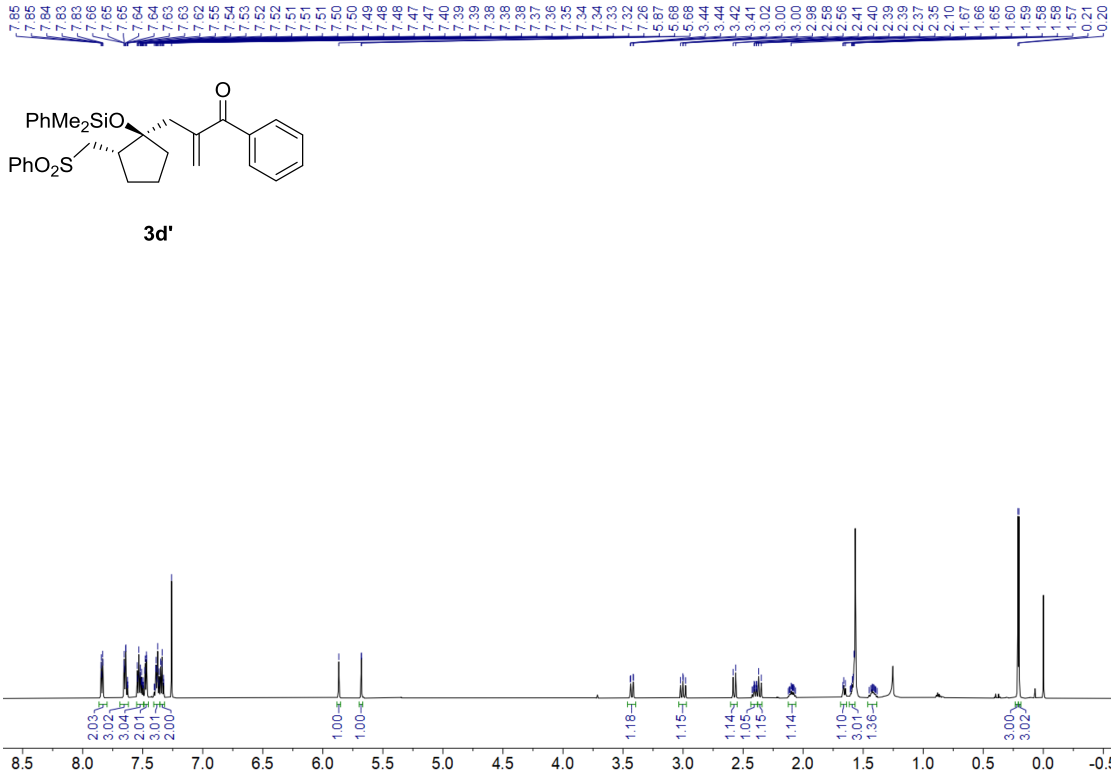

Supplementary Figure 45: ^{1}H NMR of **3d’** (600 MHz, $CDCl_3$, 25 ºC)

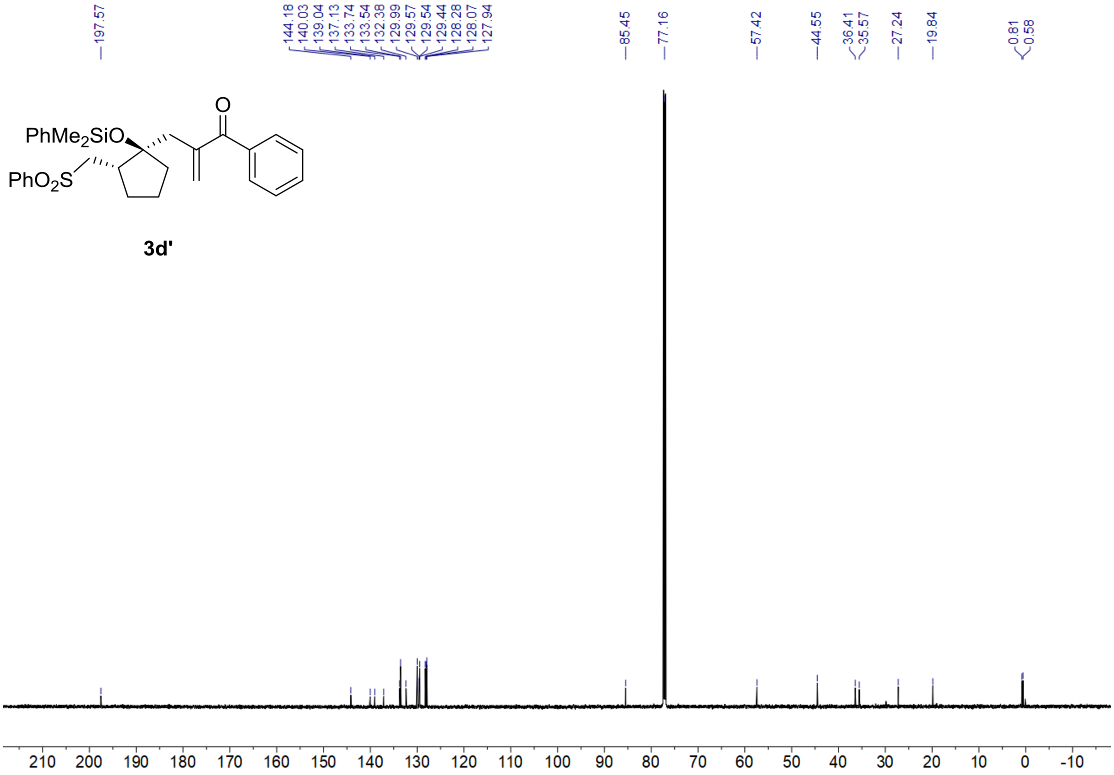

Supplementary Figure 46: ^{13}C NMR of **3d’** (151 MHz, $CDCl_3$, 25 ºC)

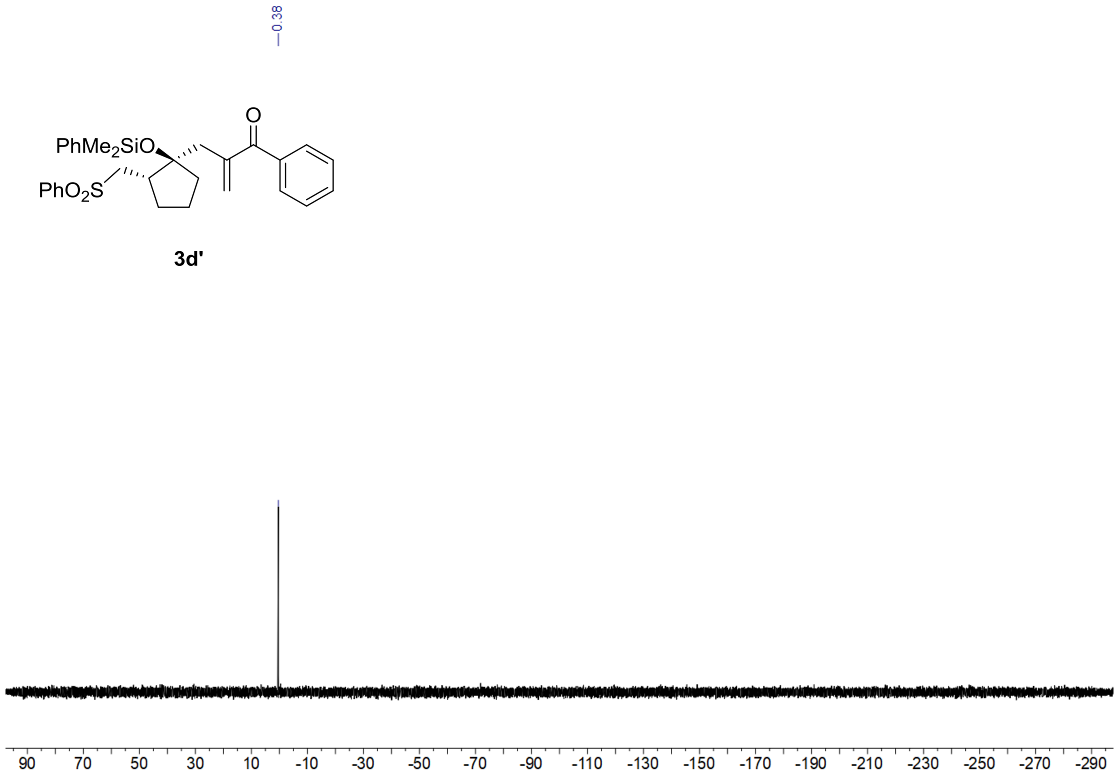

Supplementary Figure 47: ^{29}Si NMR of **3d'** (119 MHz, $CDCl_3$, 25 ºC)

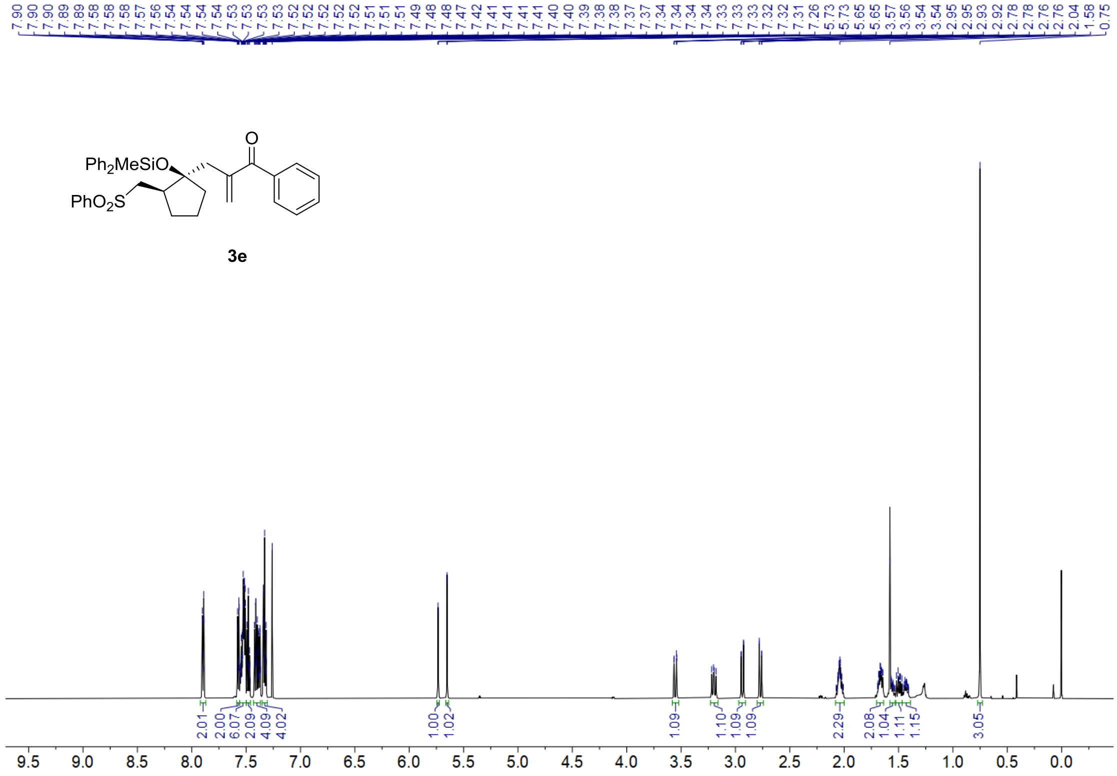

Supplementary Figure 48: ^{1}H NMR of **3e** (600 MHz, $CDCl_3$, 25 ºC)

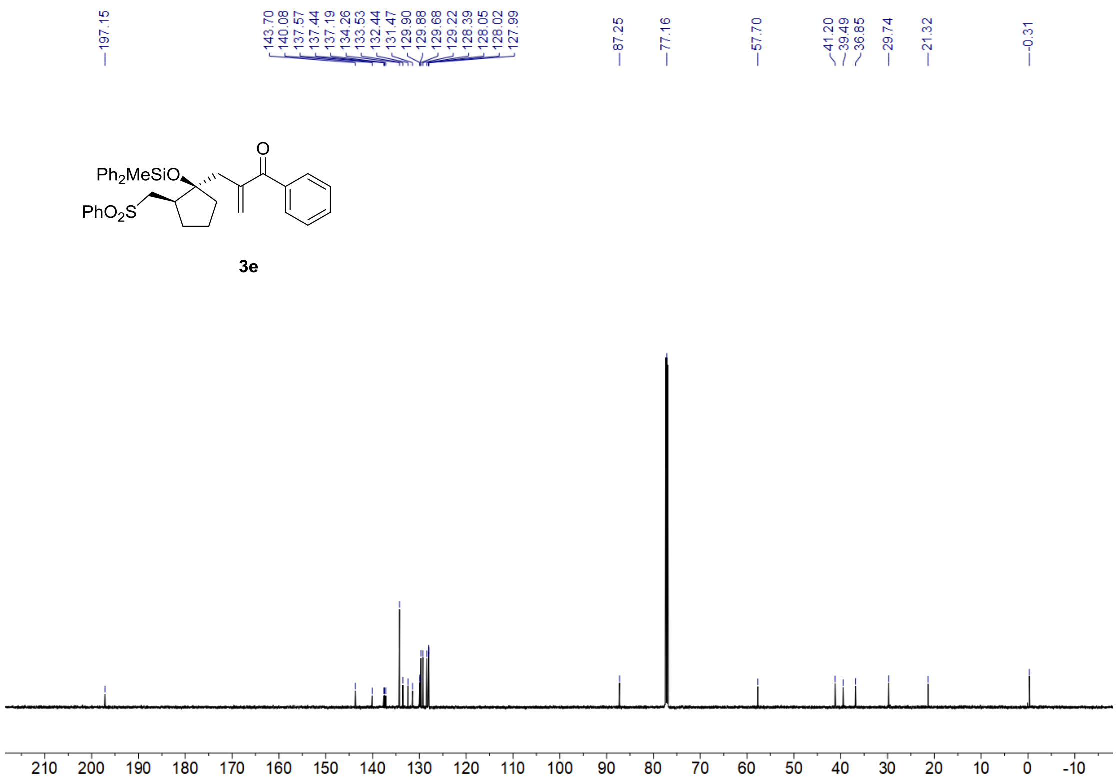

Supplementary Figure 49: ^{13}C NMR of **3e** (151 MHz, $CDCl_3$, 25 ºC)

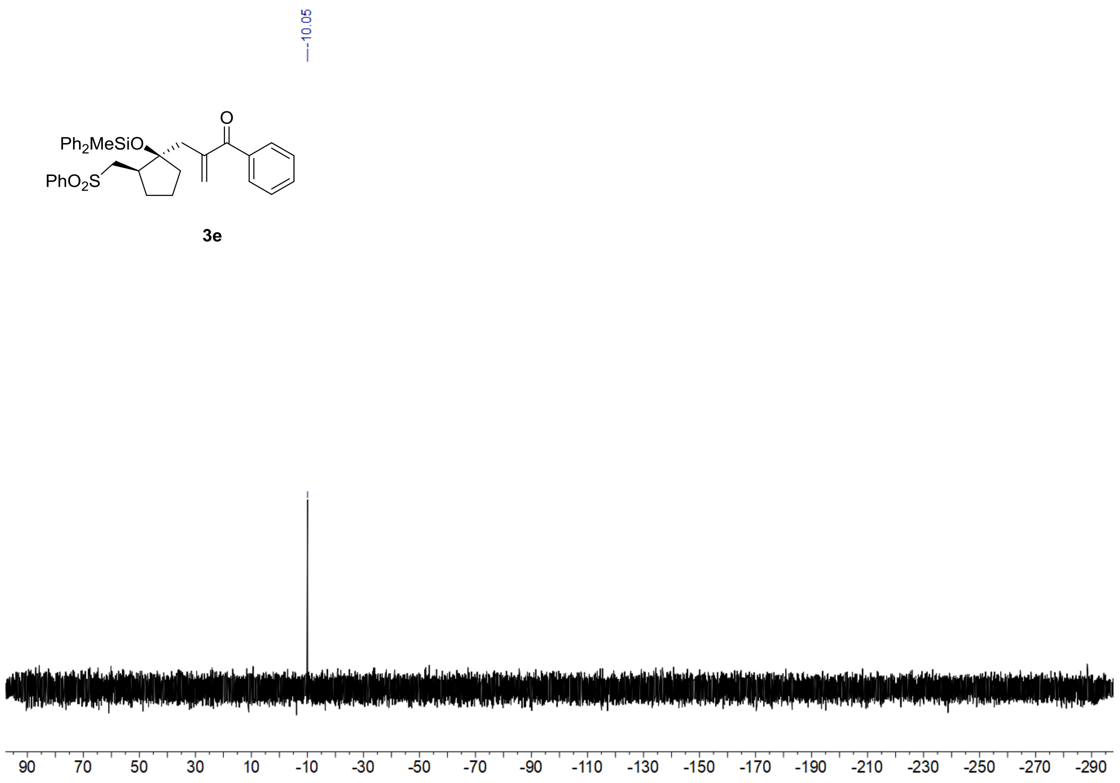

Supplementary Figure 50: ^{29}Si NMR of **3e** (119 MHz, $CDCl_3$, 25 ºC)

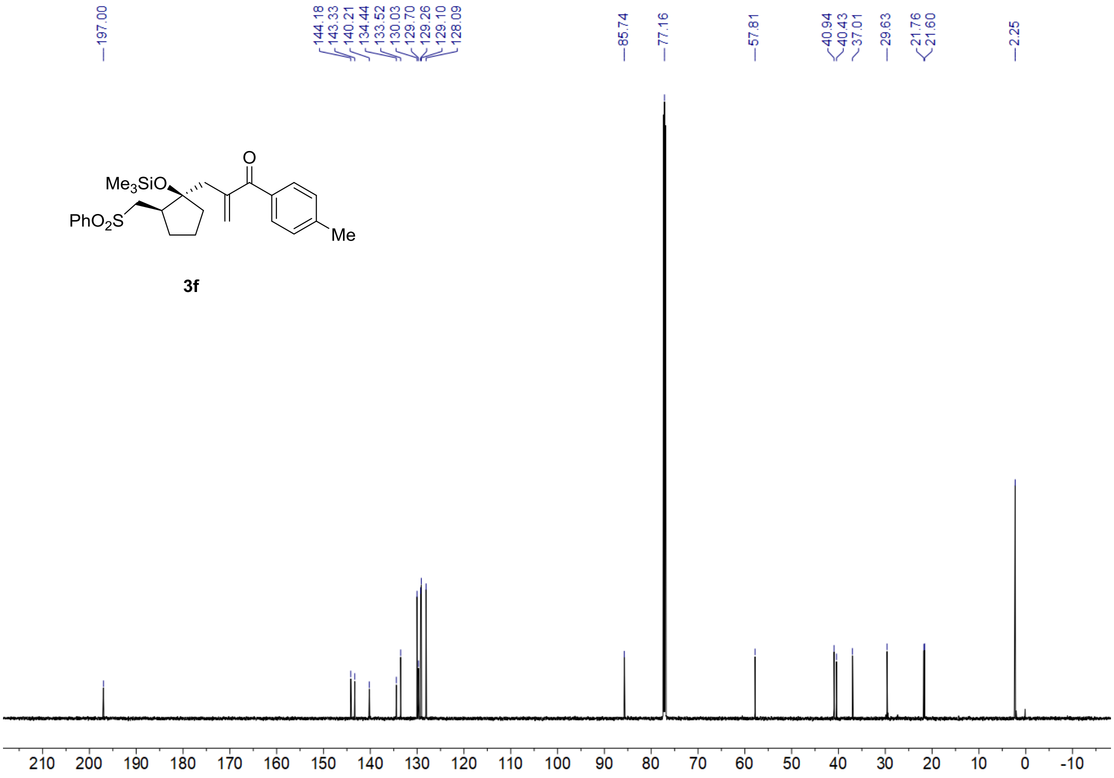

Supplementary Figure 51: ^{1}H NMR of **3f** (600 MHz, $CDCl_3$, 25 ºC)

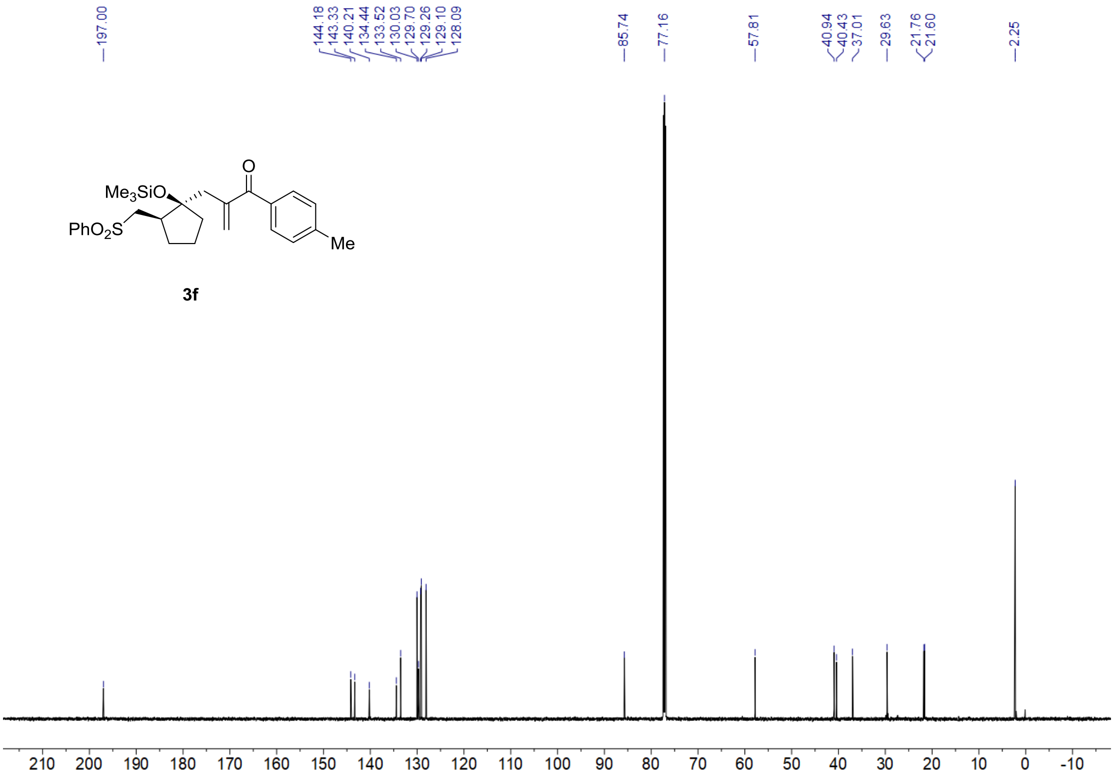

Supplementary Figure 52: ^{13}C NMR of **3f** (151 MHz, $CDCl_3$, 25 ºC)

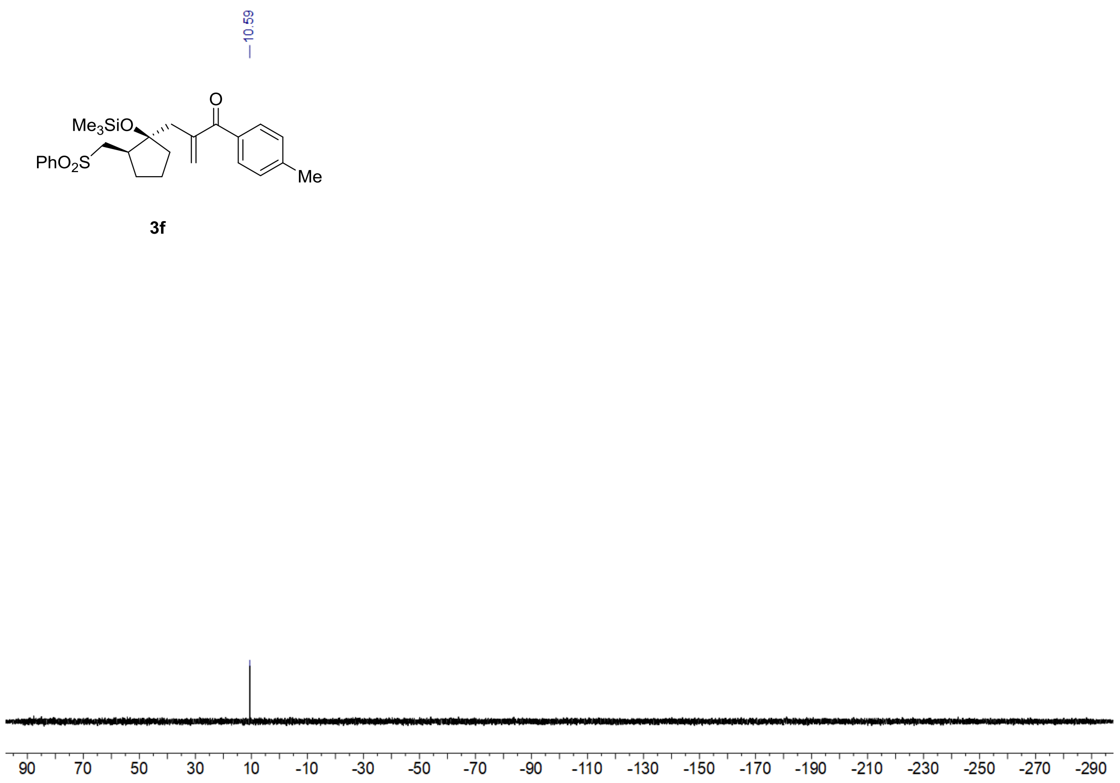

Supplementary Figure 53: ^{29}Si NMR of **3f** (119 MHz, $CDCl_3$, 25 ºC)

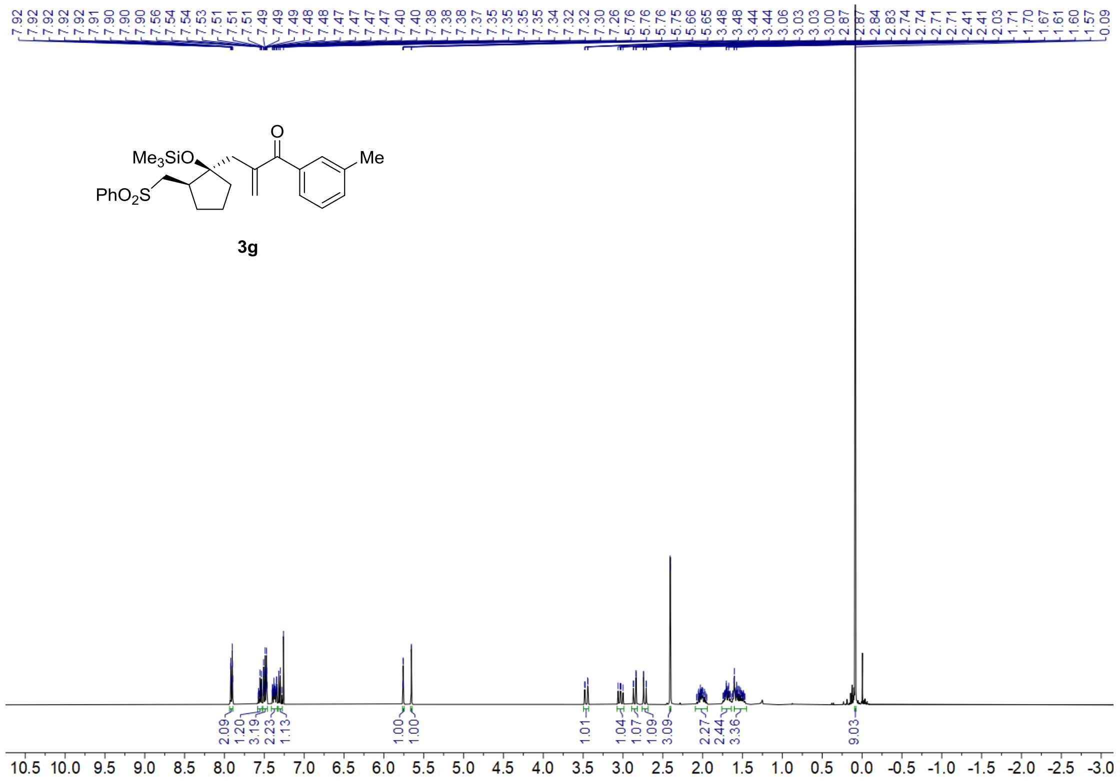

Supplementary Figure 54: ^{1}H NMR of **3g** (600 MHz, $CDCl_3$, 25 ºC)

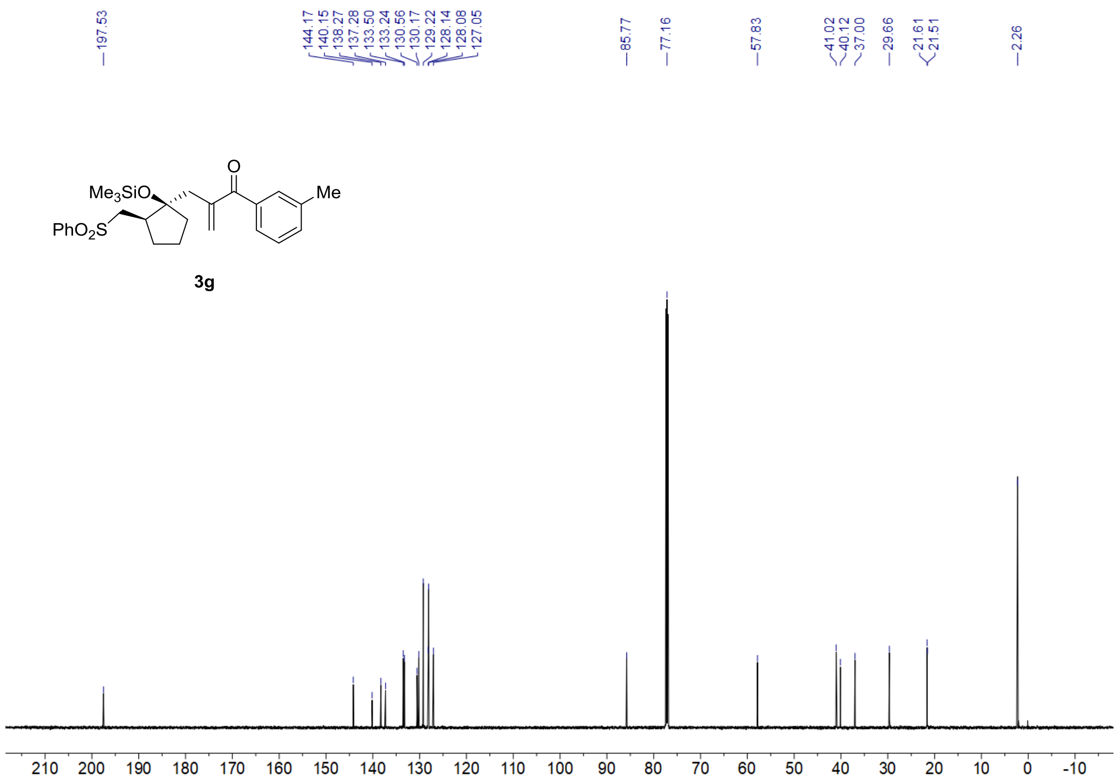

Supplementary Figure 55: ^{13}C NMR of **3g** (151 MHz, $CDCl_3$, 25 ºC)

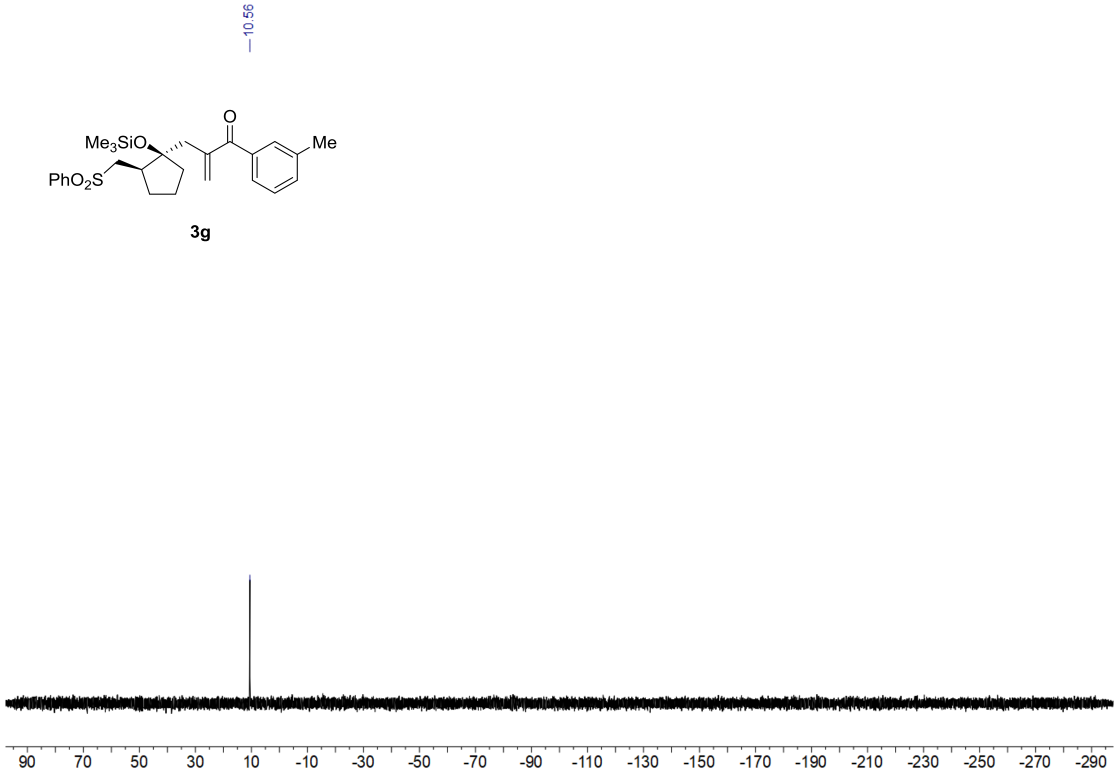

Supplementary Figure 56: ^{29}Si NMR of **3g** (119 MHz, $CDCl_3$, 25 ºC)

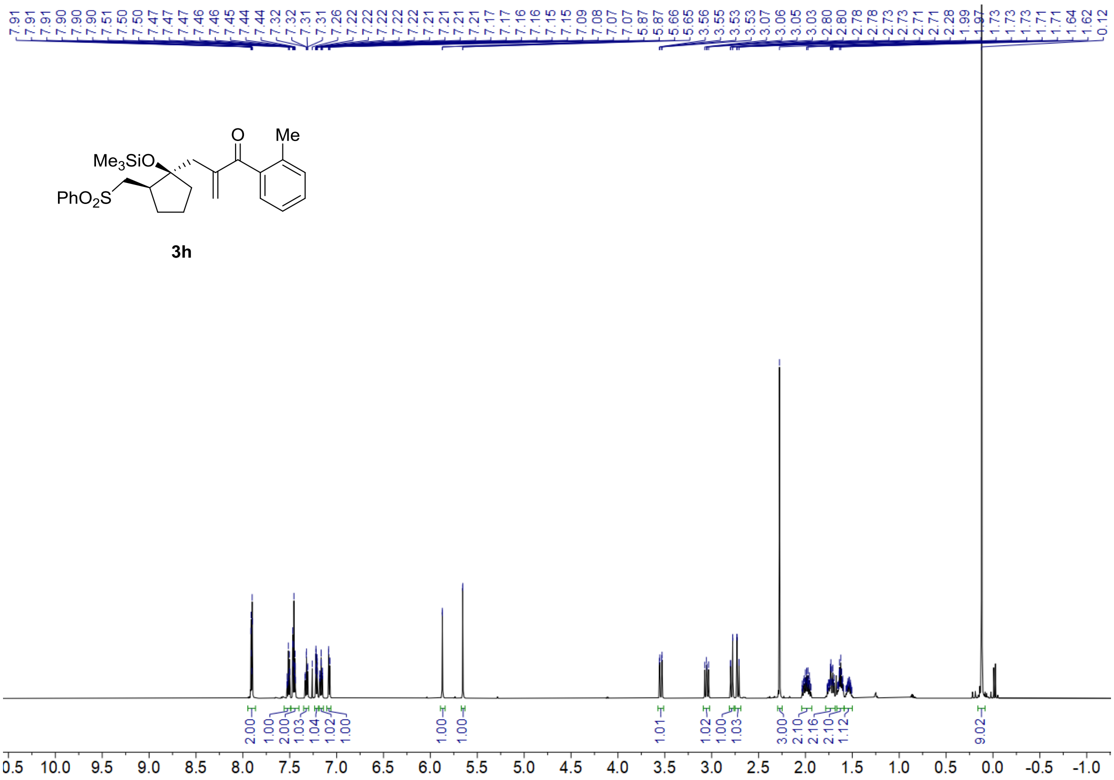

Supplementary Figure 57: ^{1}H NMR of **3h** (600 MHz, $CDCl_3$, 25 ºC)

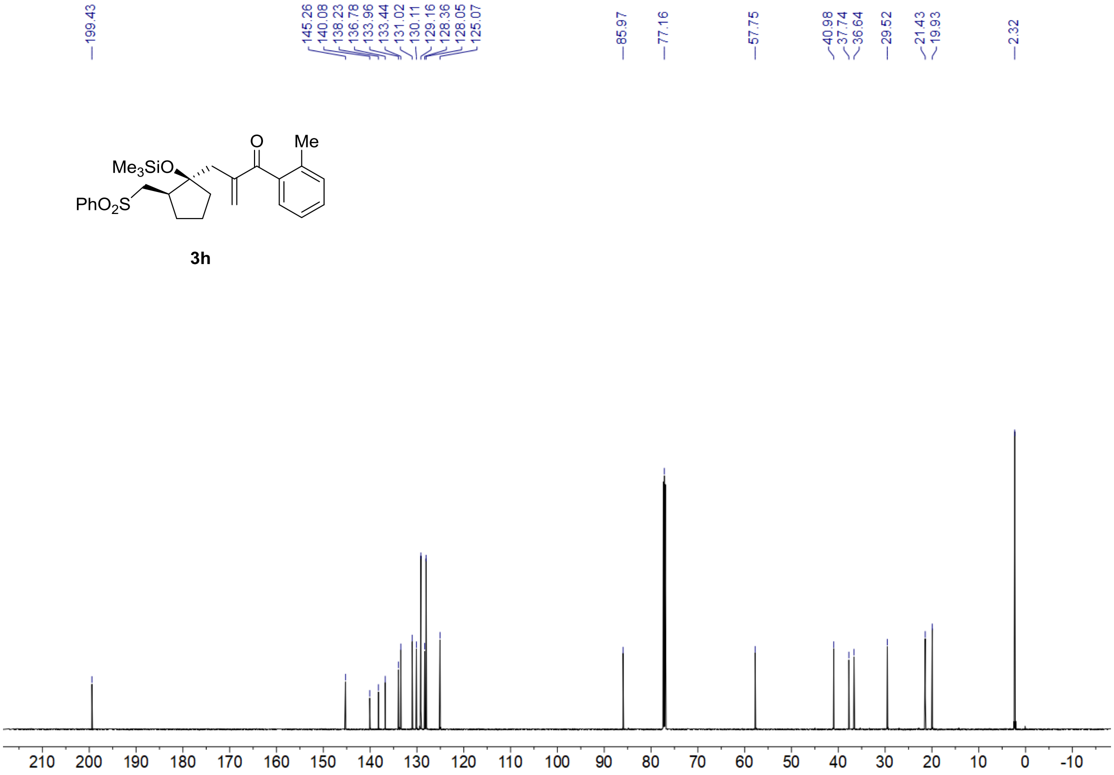

Supplementary Figure 58: ^{13}C NMR of **3h** (151 MHz, $CDCl_3$, 25 ºC)

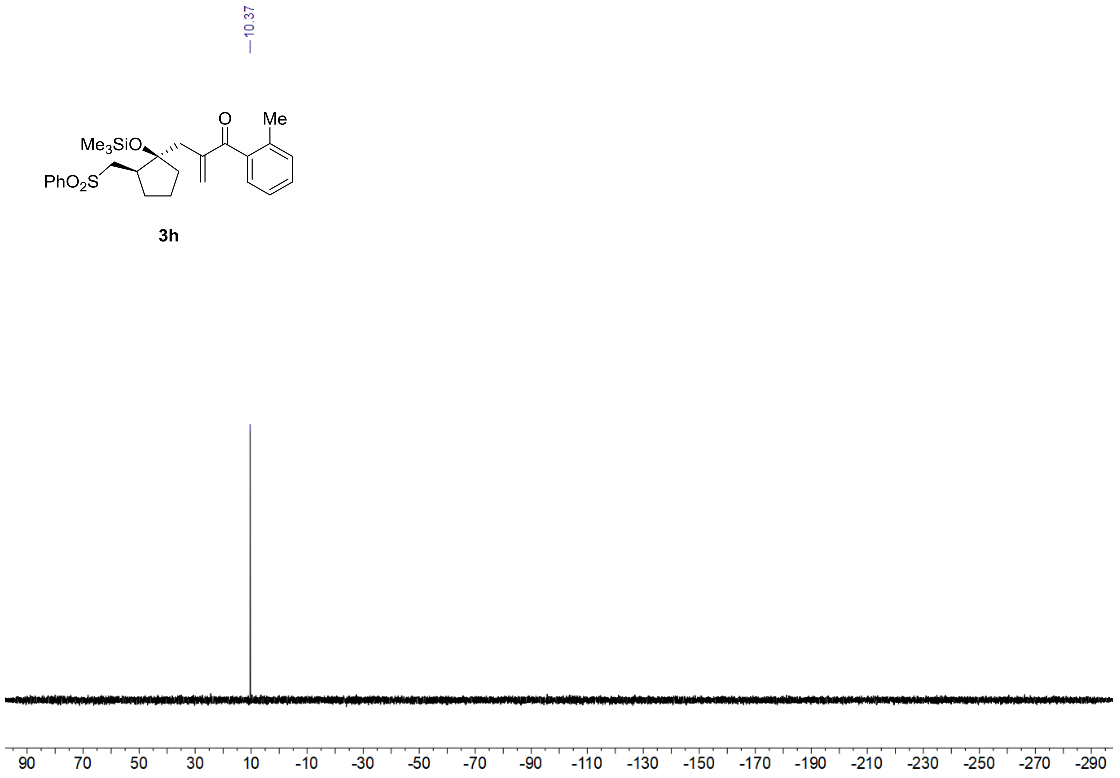

Supplementary Figure 59: ^{29}Si NMR of **3h** (119 MHz, $CDCl_3$, 25 ºC)

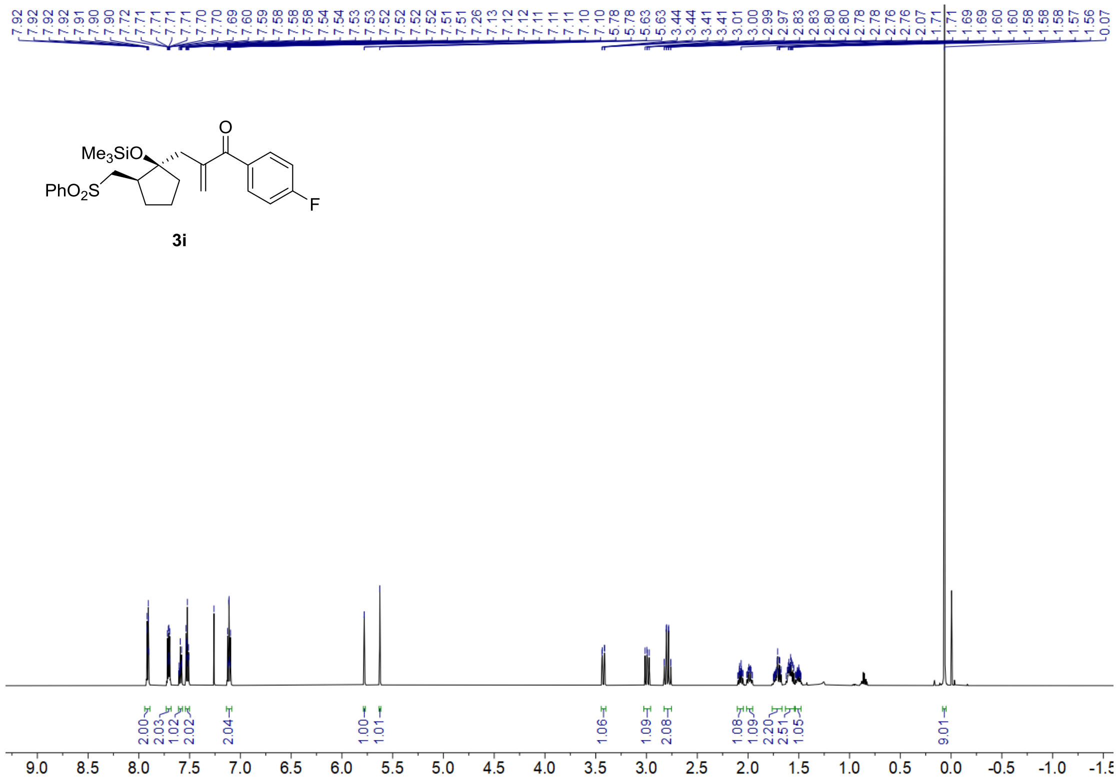

Supplementary Figure 60: ^{1}H NMR of **3i** (600 MHz, $CDCl_3$, 25 ºC)

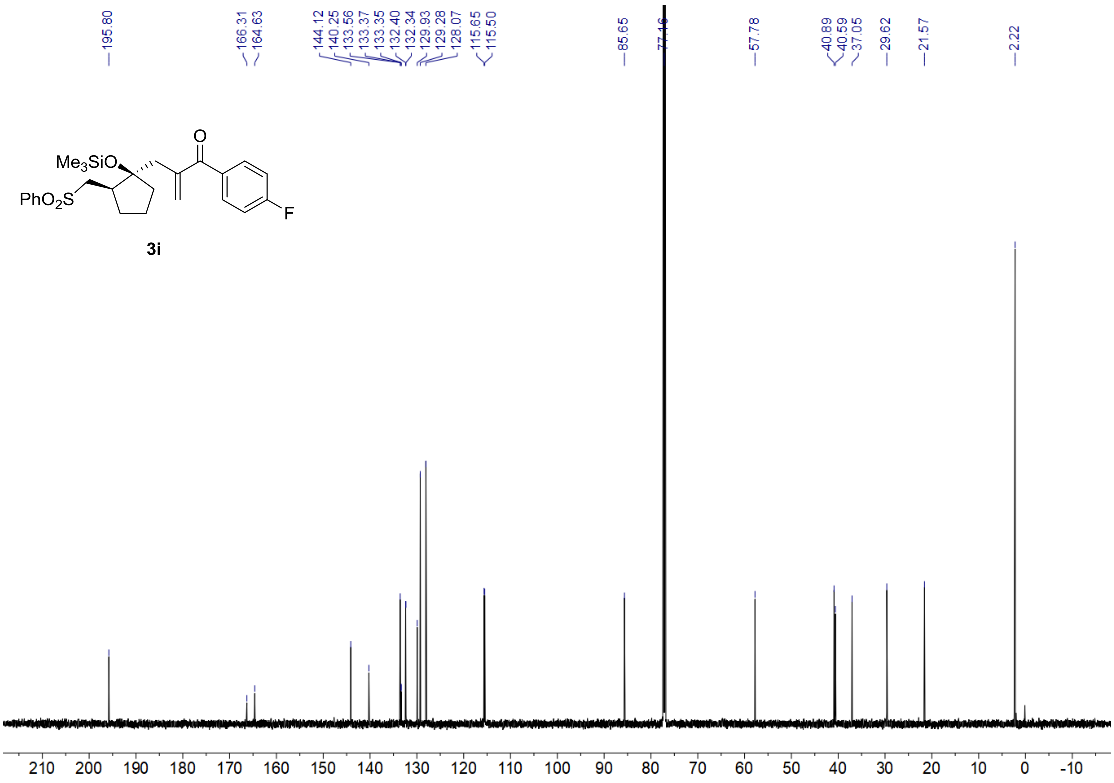

Supplementary Figure 61: ^{13}C NMR of **3i** (151 MHz, $CDCl_3$, 25 ºC)

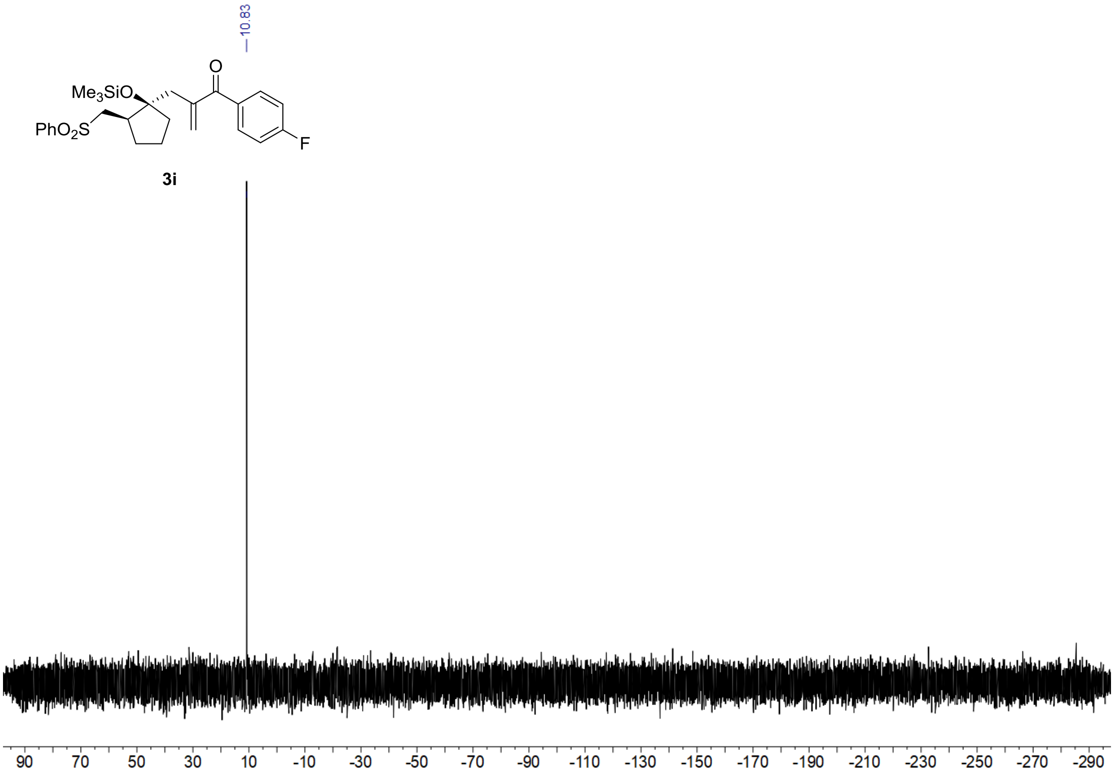

Supplementary Figure 62: ^{29}Si NMR of **3i** (119 MHz, $CDCl_3$, 25 ºC)

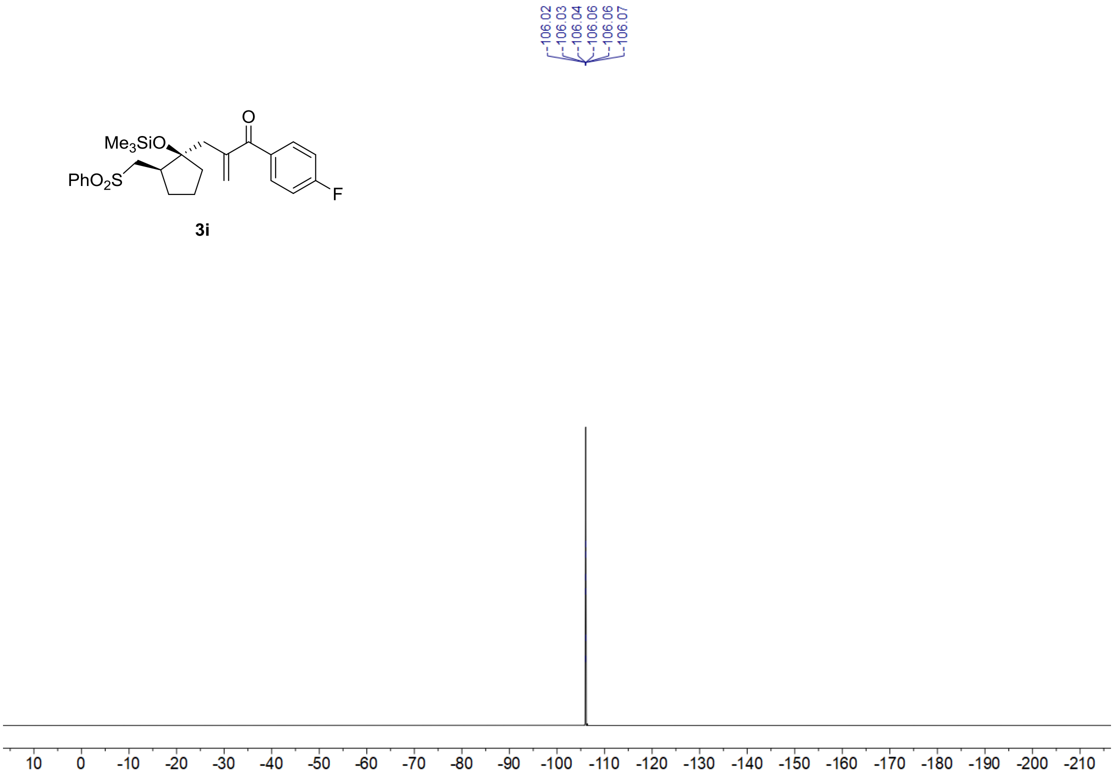

Supplementary Figure 63: ^{19}F NMR of **3i** (565 MHz, $CDCl_3$, 25 ºC)

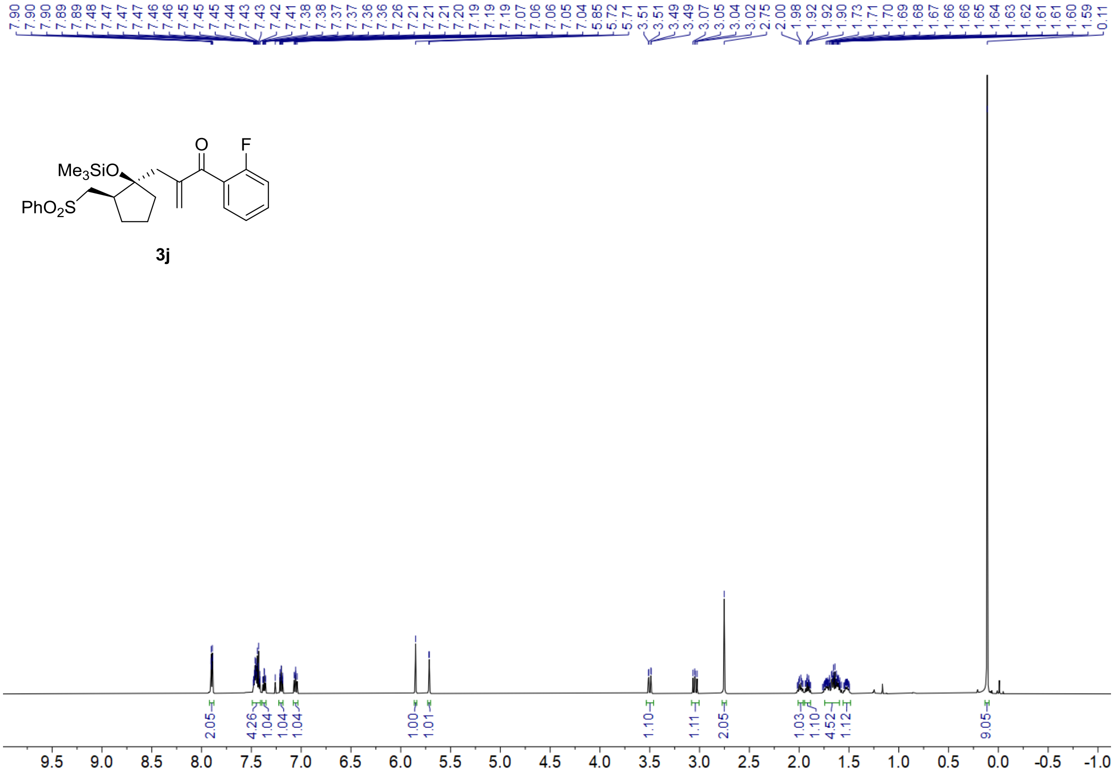

Supplementary Figure 64: ^{1}H NMR of **3j** (600 MHz, $CDCl_3$, 25 ºC)

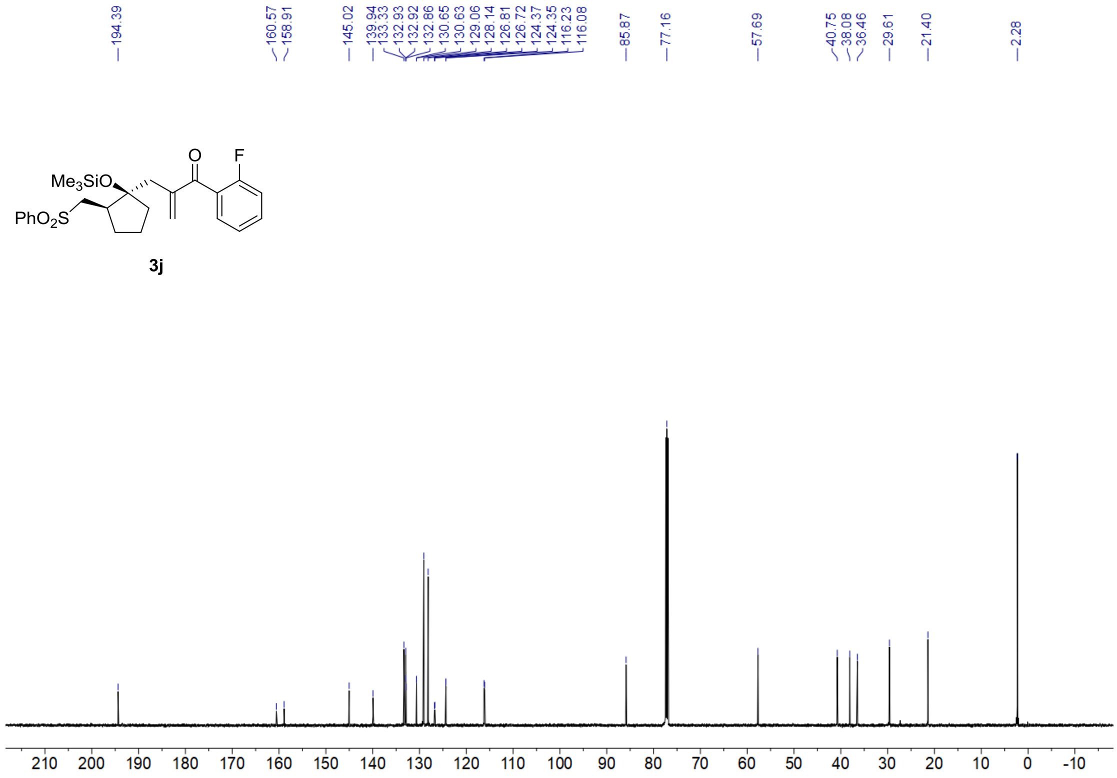

Supplementary Figure 65: ^{13}C NMR of **3j** (151 MHz, $CDCl_3$, 25 ºC)

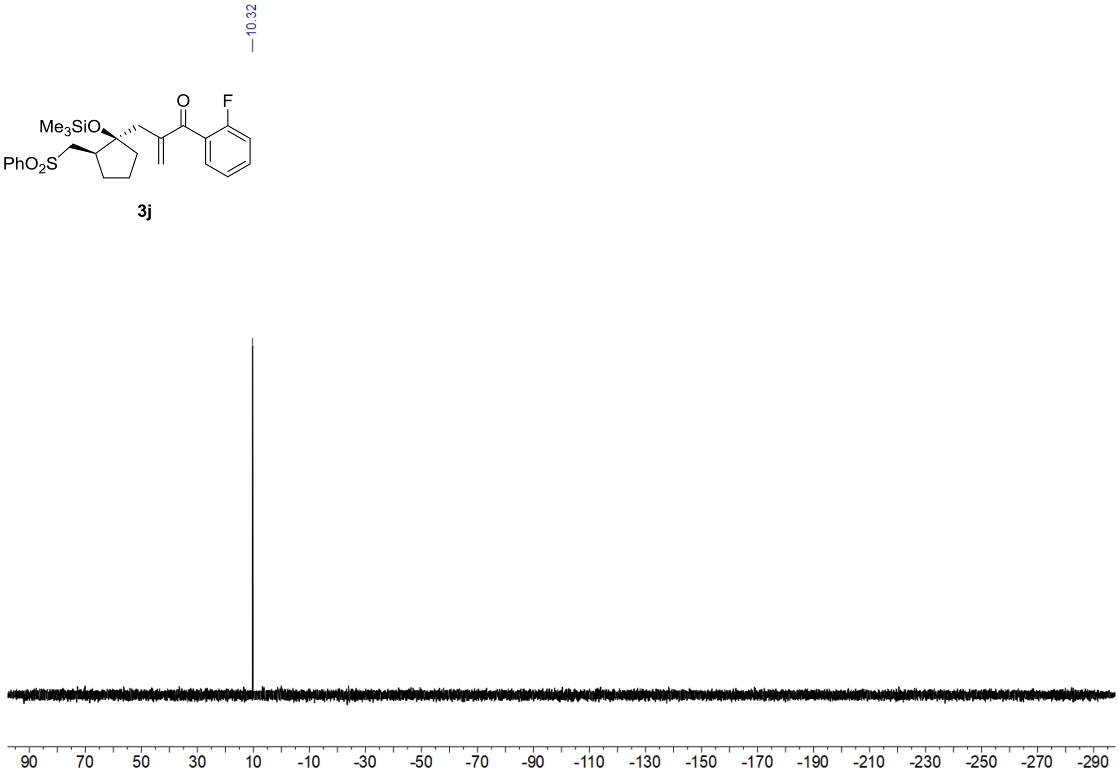

Supplementary Figure 66: ^{29}Si NMR of **3j** (119 MHz, $CDCl_3$, 25 ºC)

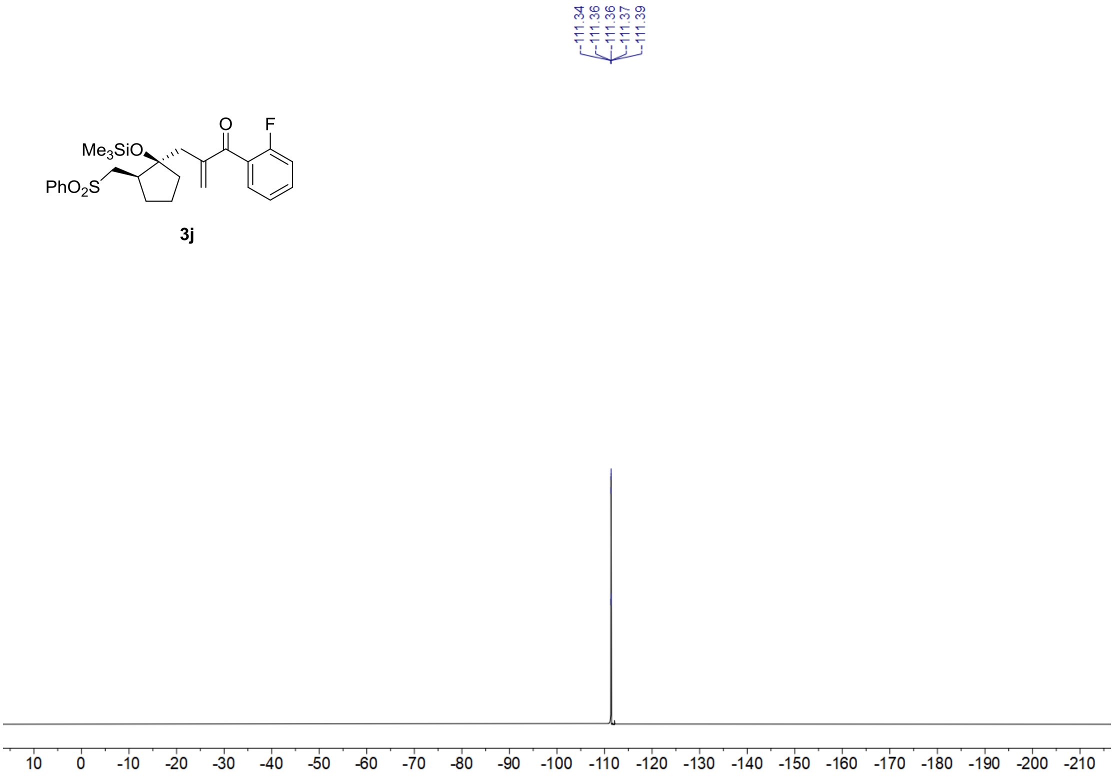

Supplementary Figure 67: ^{19}F NMR of **3j** (565 MHz, CDCl$_3$, 25 ºC)

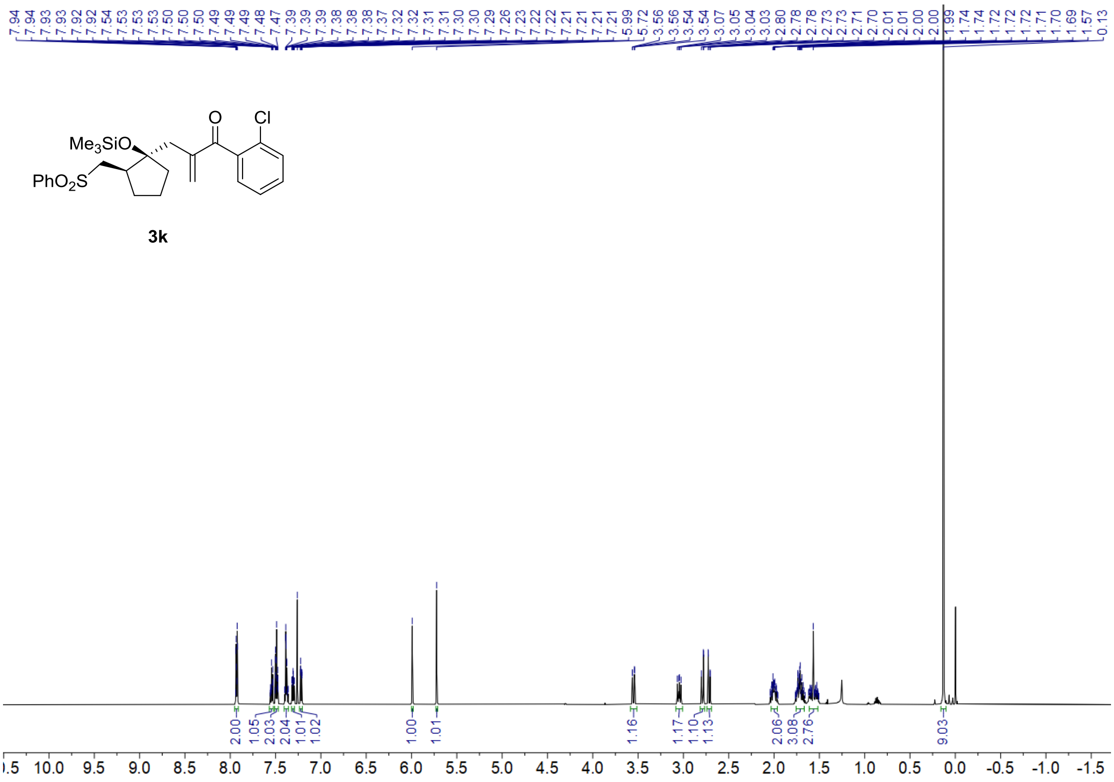

Supplementary Figure 68: ^{1}H NMR of **3k** (600 MHz, CDCl$_3$, 25 ºC)

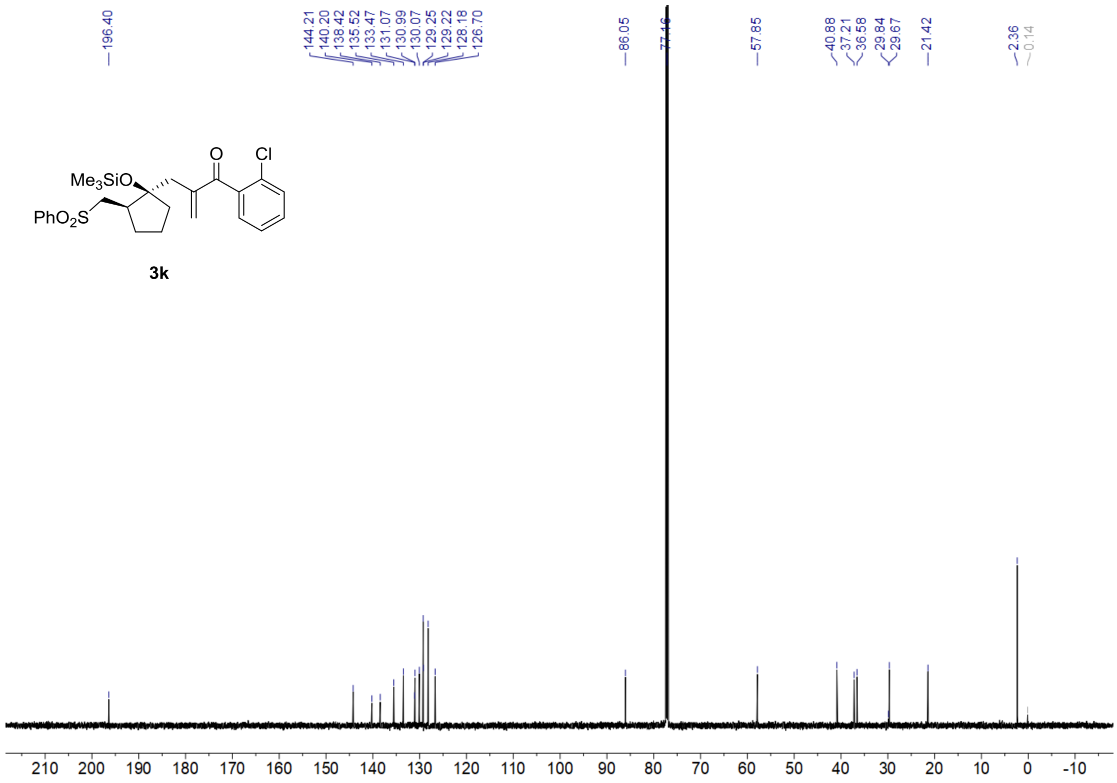

Supplementary Figure 69: ^{13}C NMR of **3k** (151 MHz, $CDCl_3$, 25 ºC)

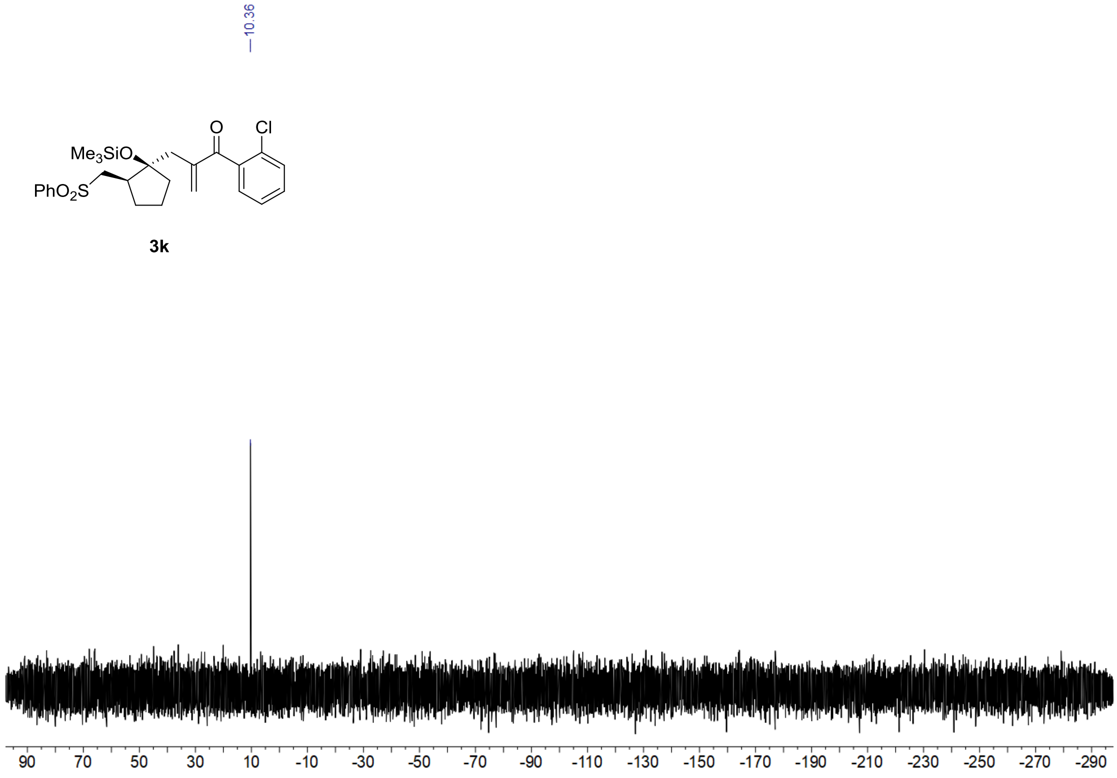

Supplementary Figure 70: ^{29}Si NMR of **3k** (119 MHz, $CDCl_3$, 25 ºC)

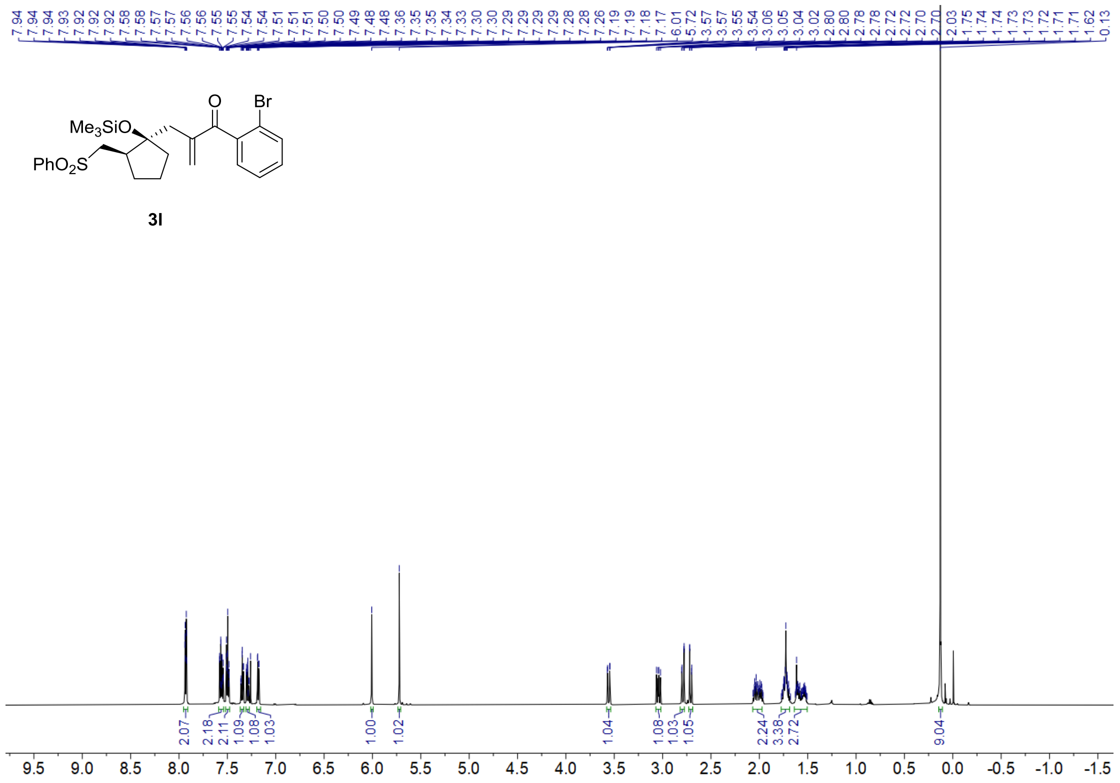

Supplementary Figure 71: ^{1}H NMR of **3l** (600 MHz, $CDCl_3$, 25 ºC)

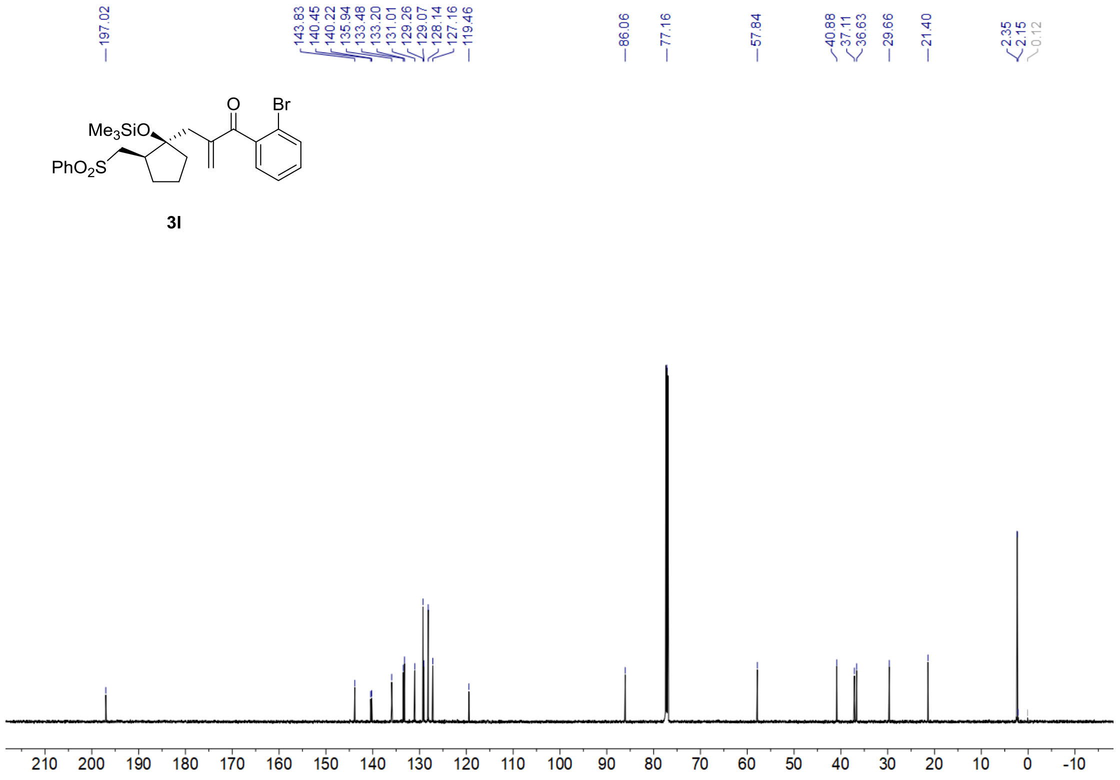

Supplementary Figure 72: ^{13}C NMR of **3l** (151 MHz, $CDCl_3$, 25 ºC)

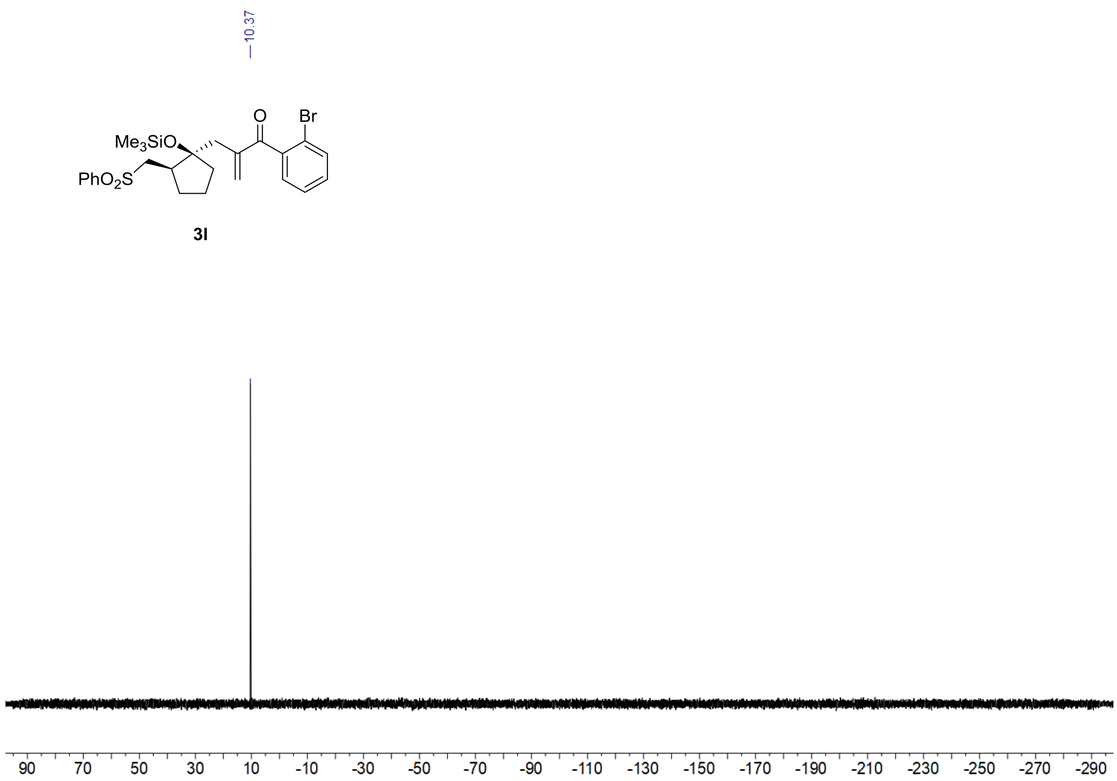

Supplementary Figure 73: ^{29}Si NMR of **3l** (119 MHz, $CDCl_3$, 25 ºC)

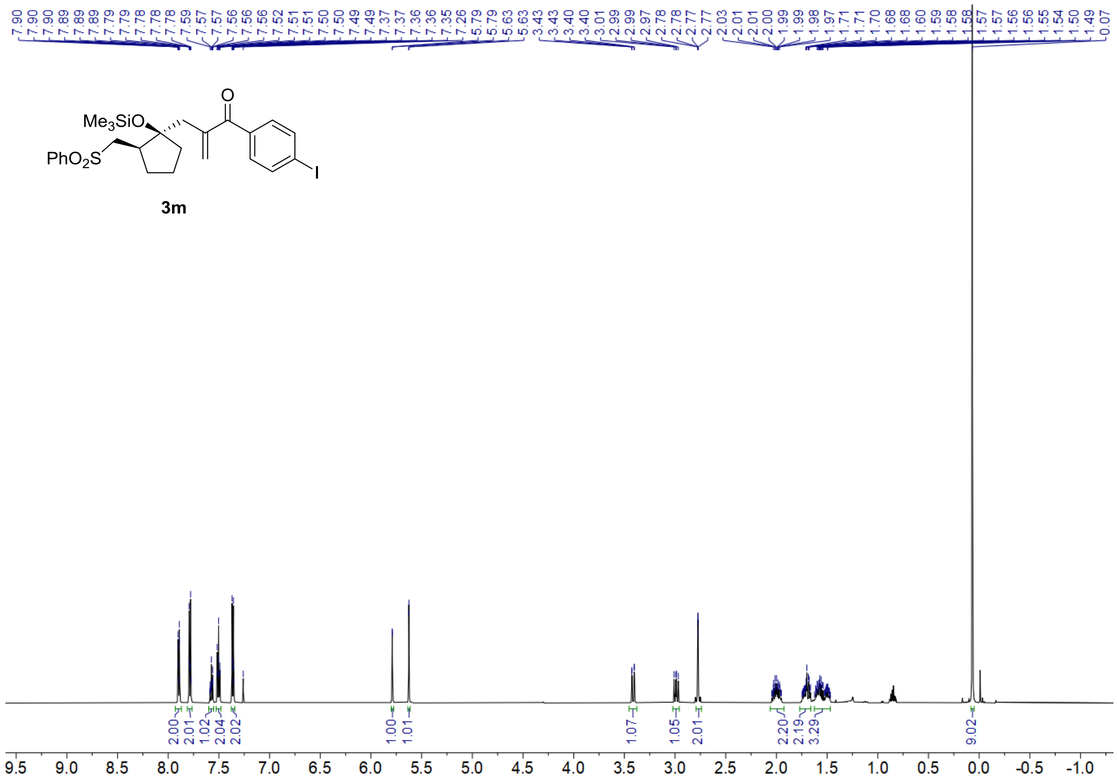

Supplementary Figure 74: ^{1}H NMR of **3m** (600 MHz, $CDCl_3$, 25 ºC)

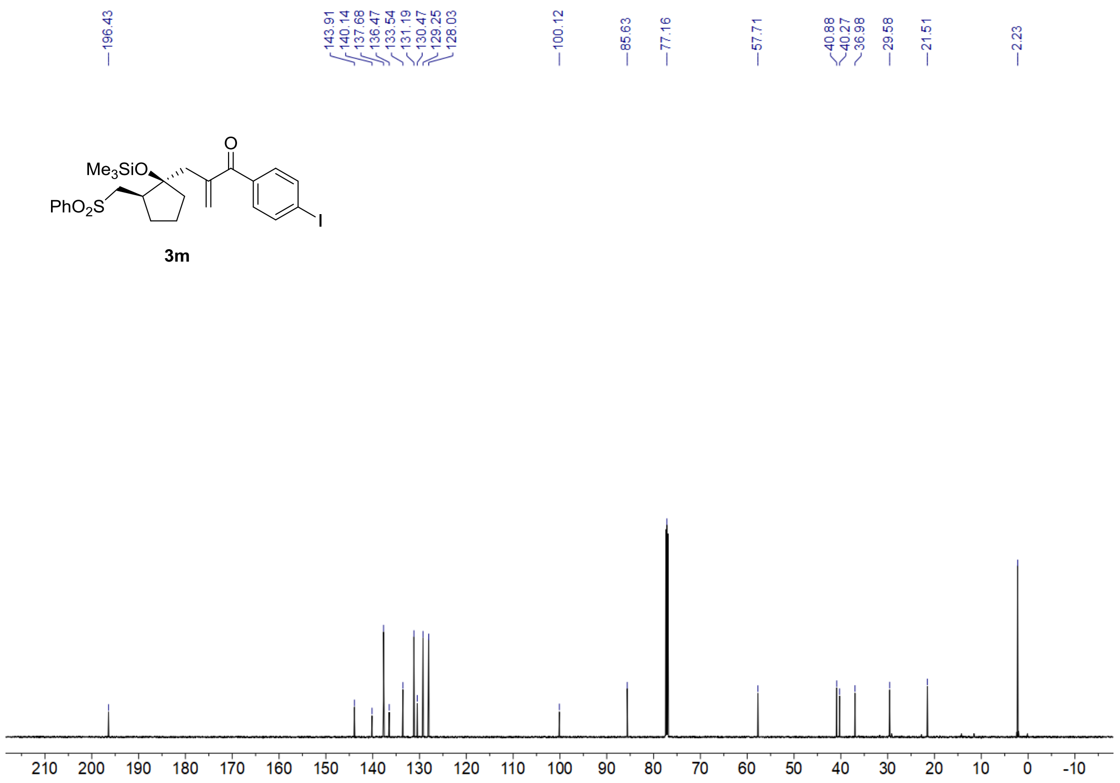

Supplementary Figure 75: ^{13}C NMR of **3m** (151 MHz, $CDCl_3$, 25 ºC)

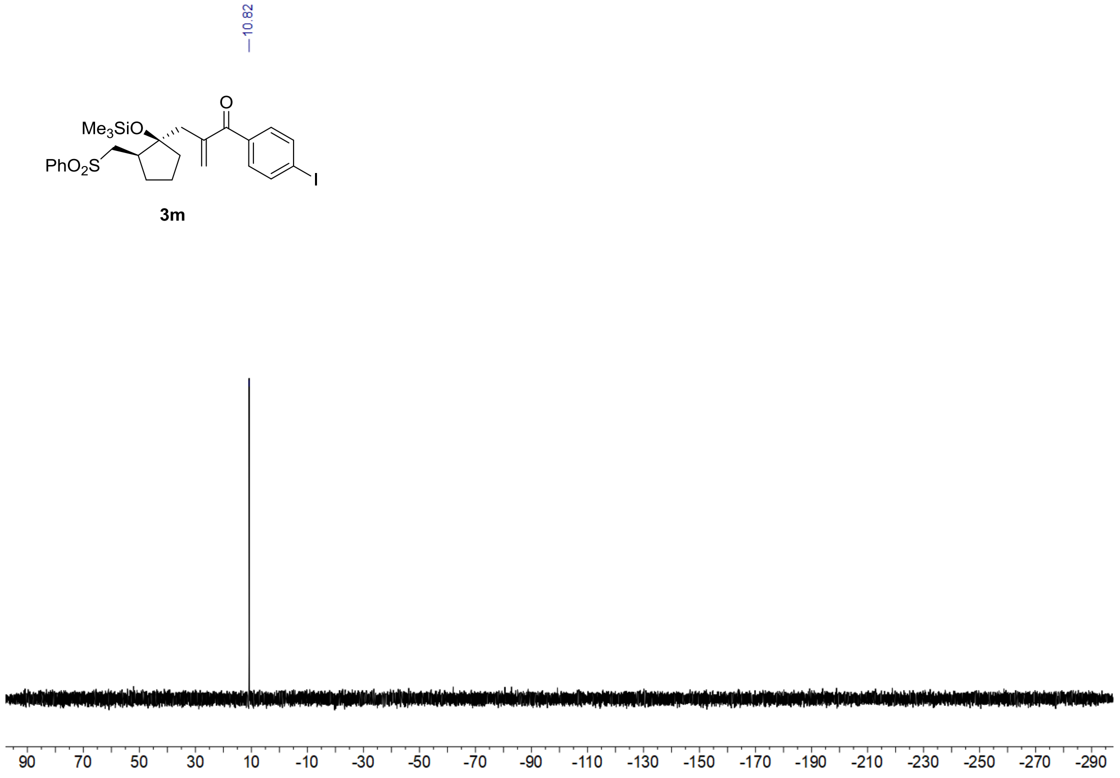

Supplementary Figure 76: ^{29}Si NMR of **3m** (119 MHz, $CDCl_3$, 25 ºC)

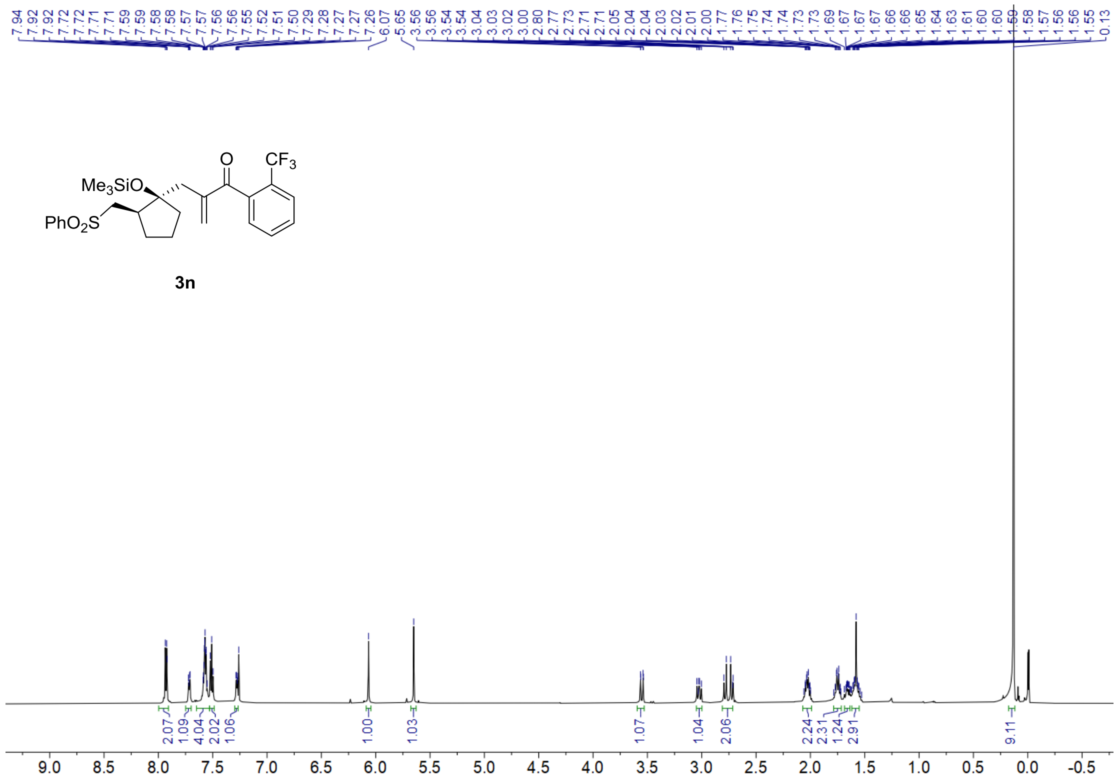

Supplementary Figure 77: ^{1}H NMR of **3n** (600 MHz, $CDCl_3$, 25 ºC)

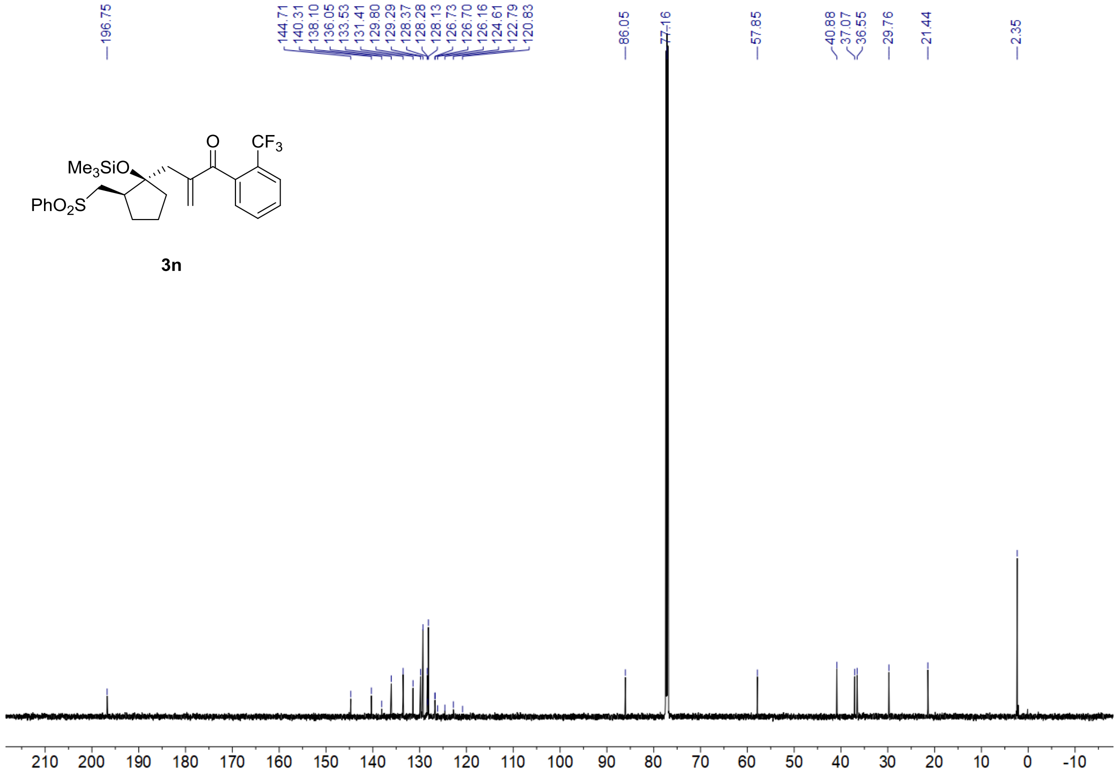

Supplementary Figure 78: ^{13}C NMR of **3n** (151 MHz, $CDCl_3$, 25 ºC)

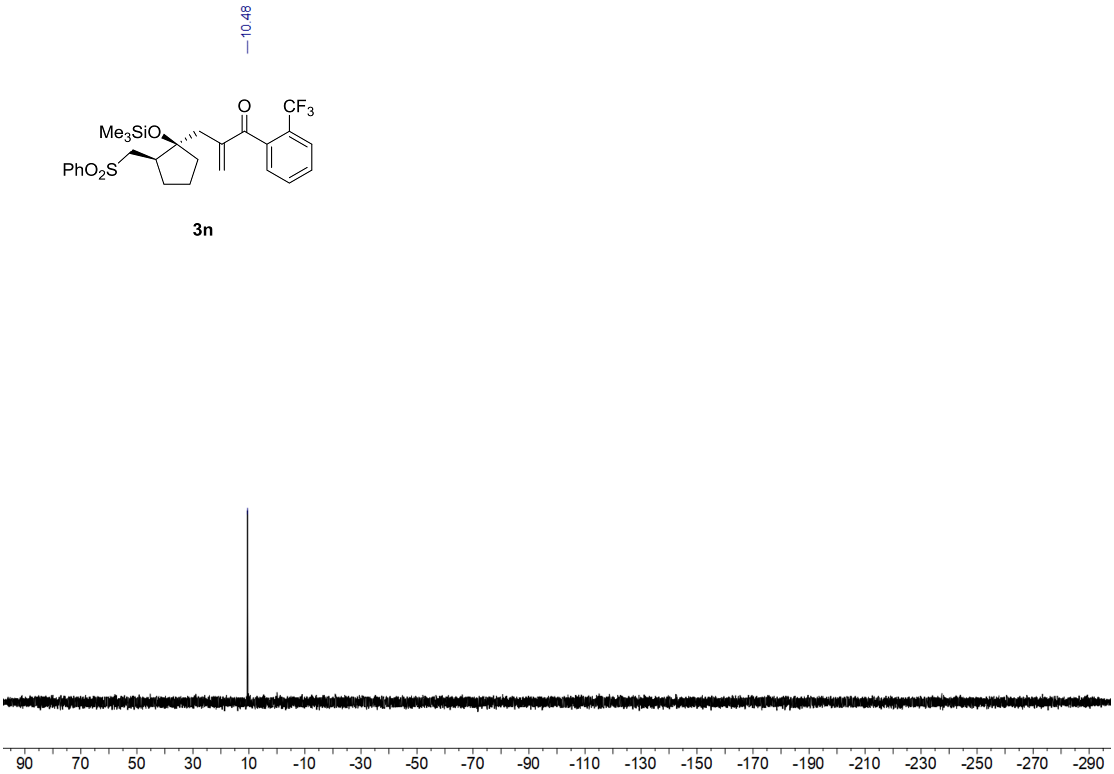

Supplementary Figure 79: ^{29}Si NMR of **3n** (119 MHz, $CDCl_3$, 25 ºC)

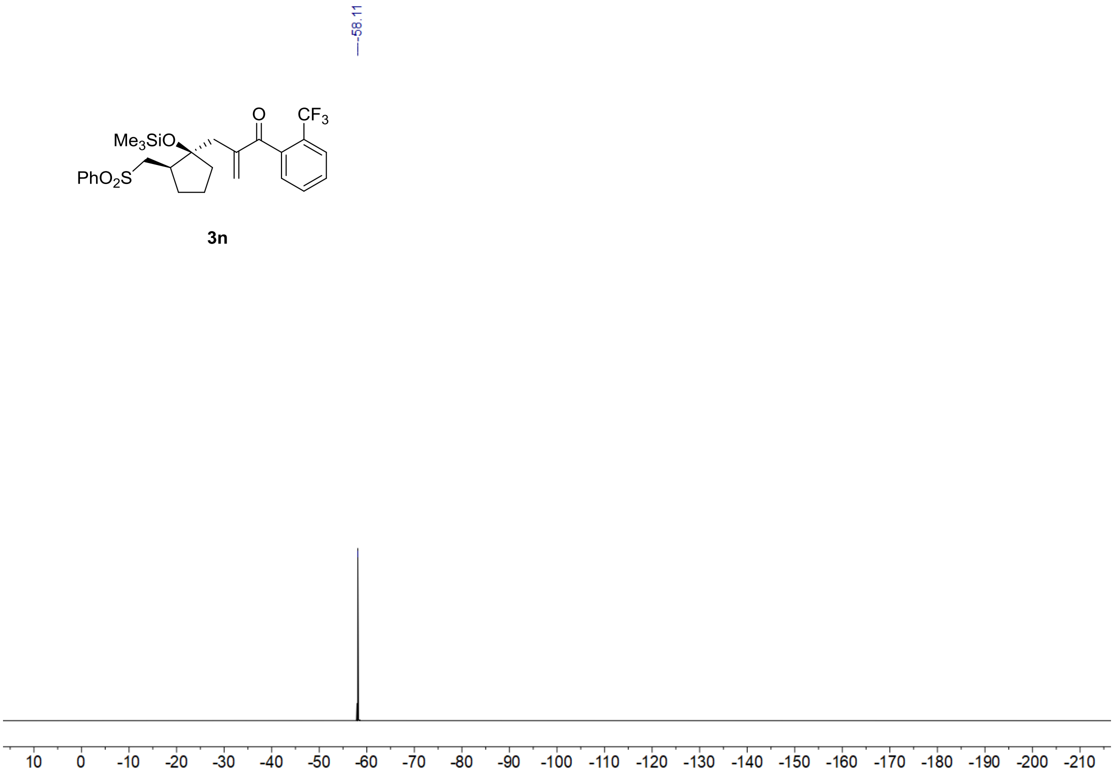

Supplementary Figure 80: ^{19}F NMR of **3n** (565 MHz, $CDCl_3$, 25 ºC)

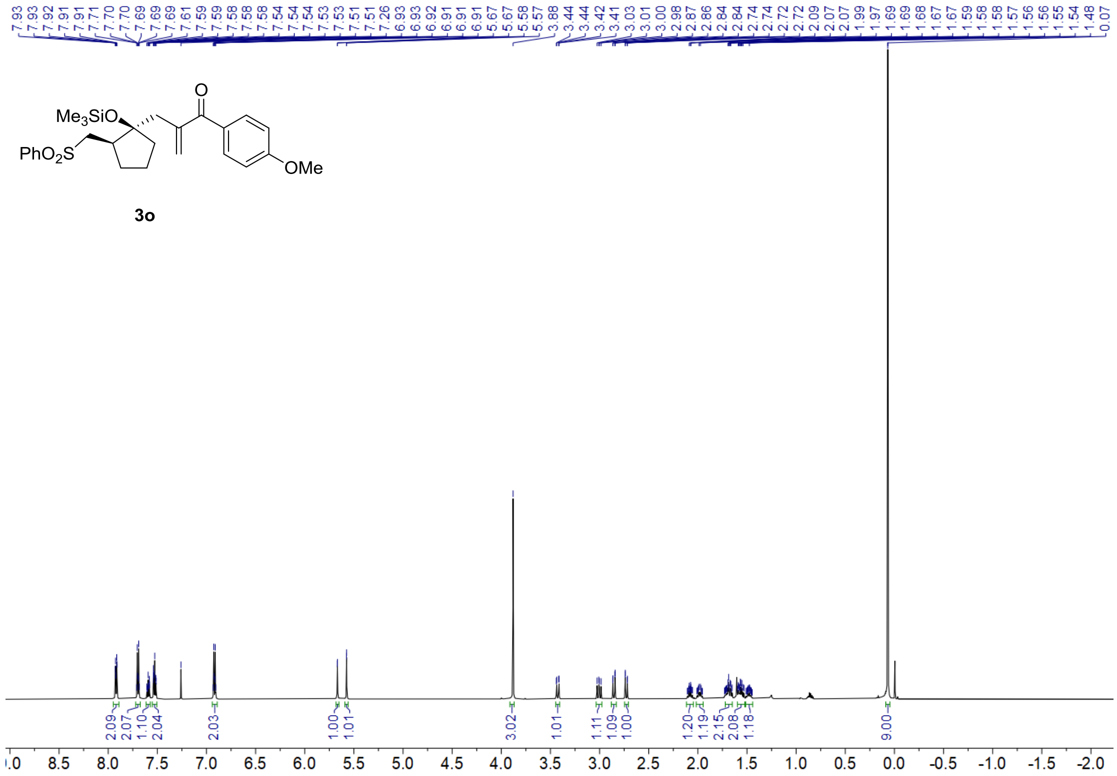

Supplementary Figure 81: ^{1}H NMR of **3o** (600 MHz, CDCl$_3$, 25 ºC)

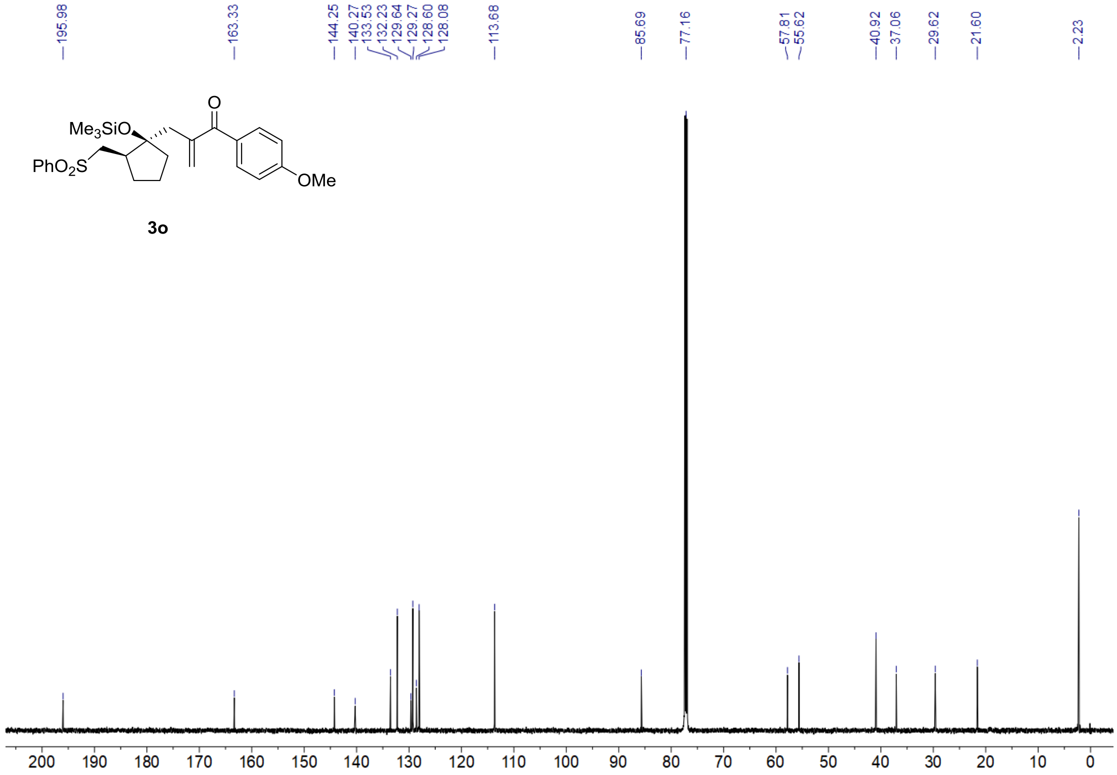

Supplementary Figure 82: ^{13}C NMR of **3o** (151 MHz, CDCl$_3$, 25 ºC)

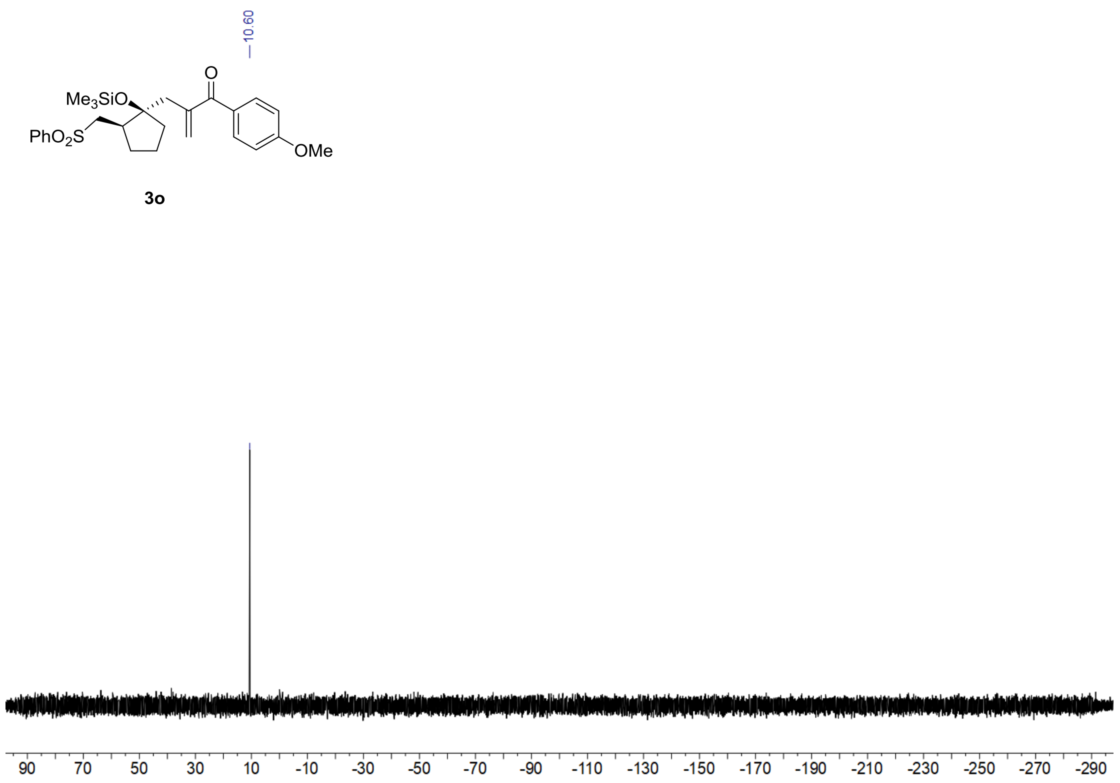

Supplementary Figure 83: ^{29}Si NMR of **3o** (119 MHz, $CDCl_3$, 25 ºC)

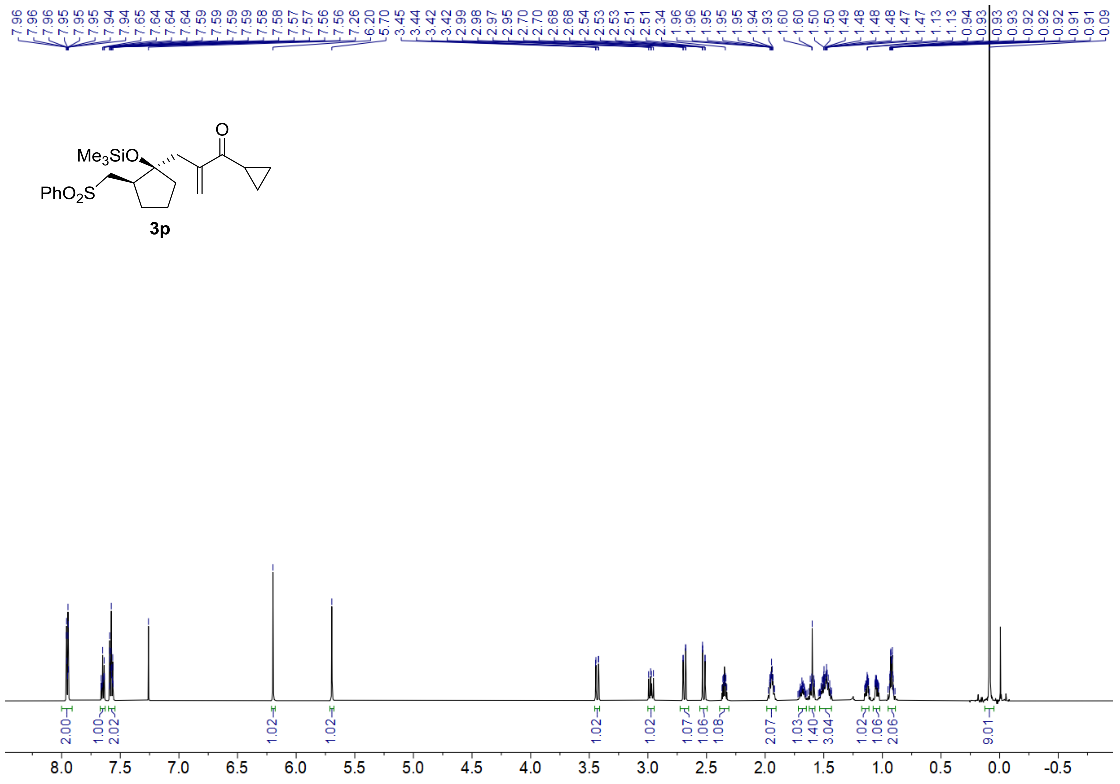

Supplementary Figure 84: ^{1}H NMR of **3p** (600 MHz, $CDCl_3$, 25 ºC)

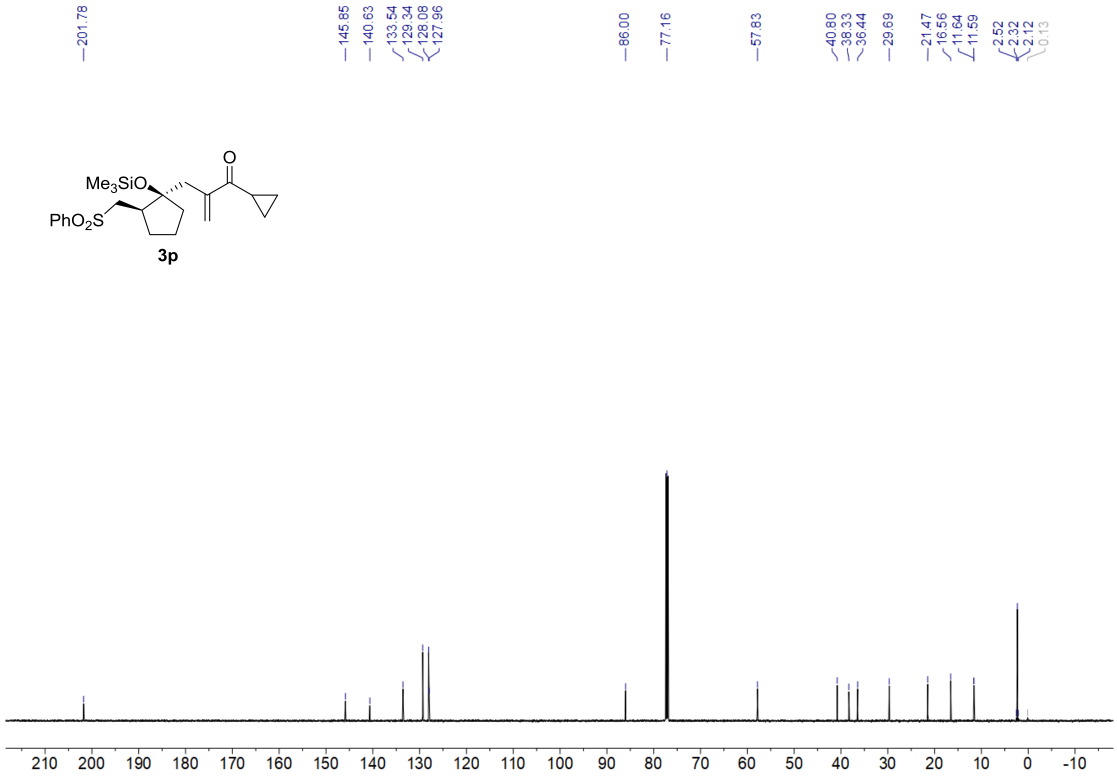

Supplementary Figure 85: ^{13}C NMR of **3p** (151 MHz, $CDCl_3$, 25 ºC)

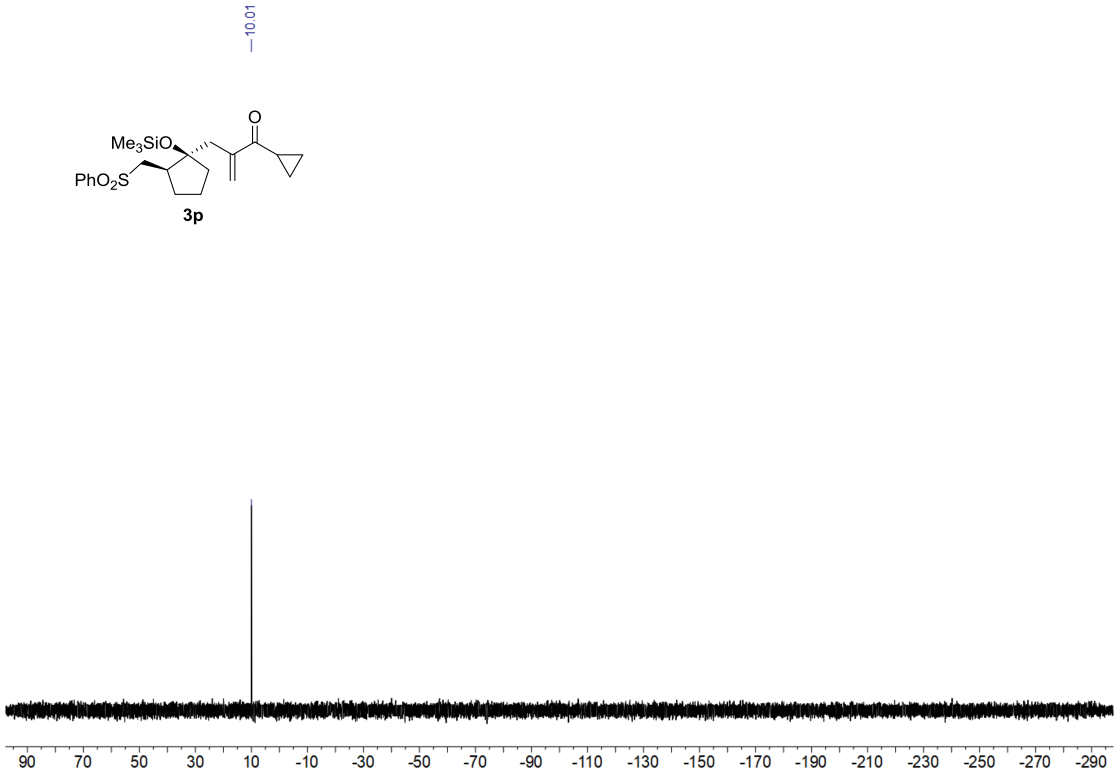

Supplementary Figure 86: ^{29}Si NMR of **3p** (119 MHz, $CDCl_3$, 25 ºC)

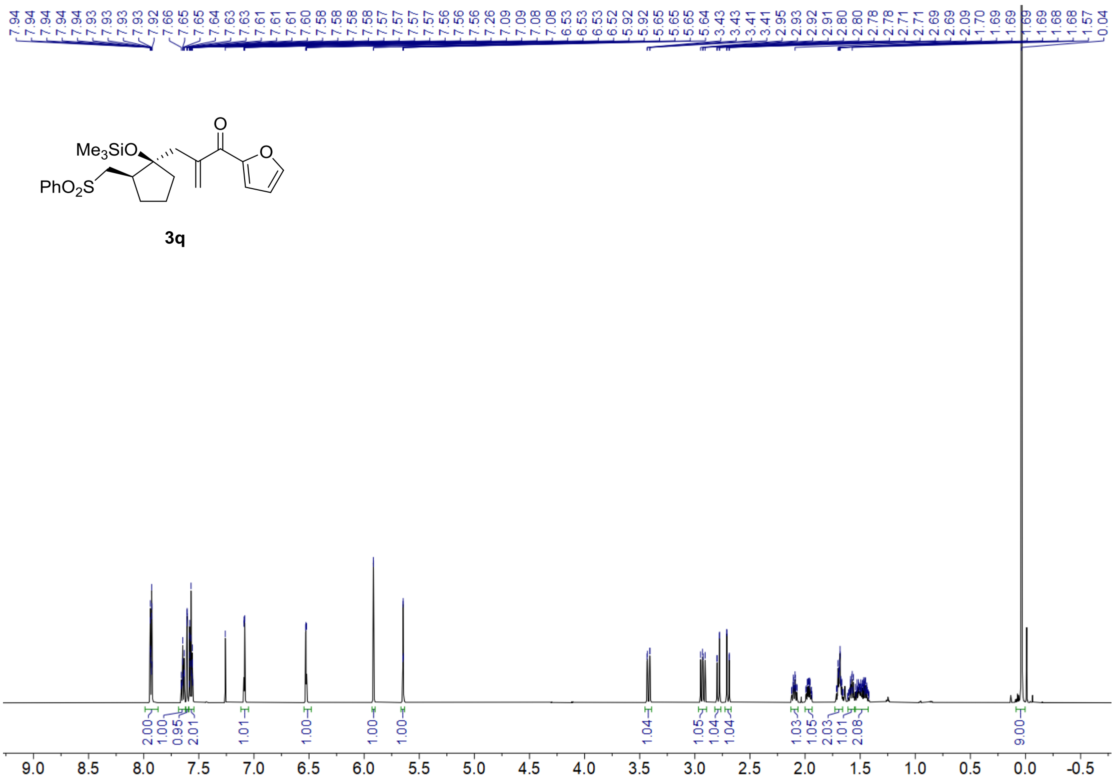

Supplementary Figure 87: ^{1}H NMR of **3q** (600 MHz, $CDCl_3$, 25 ºC)

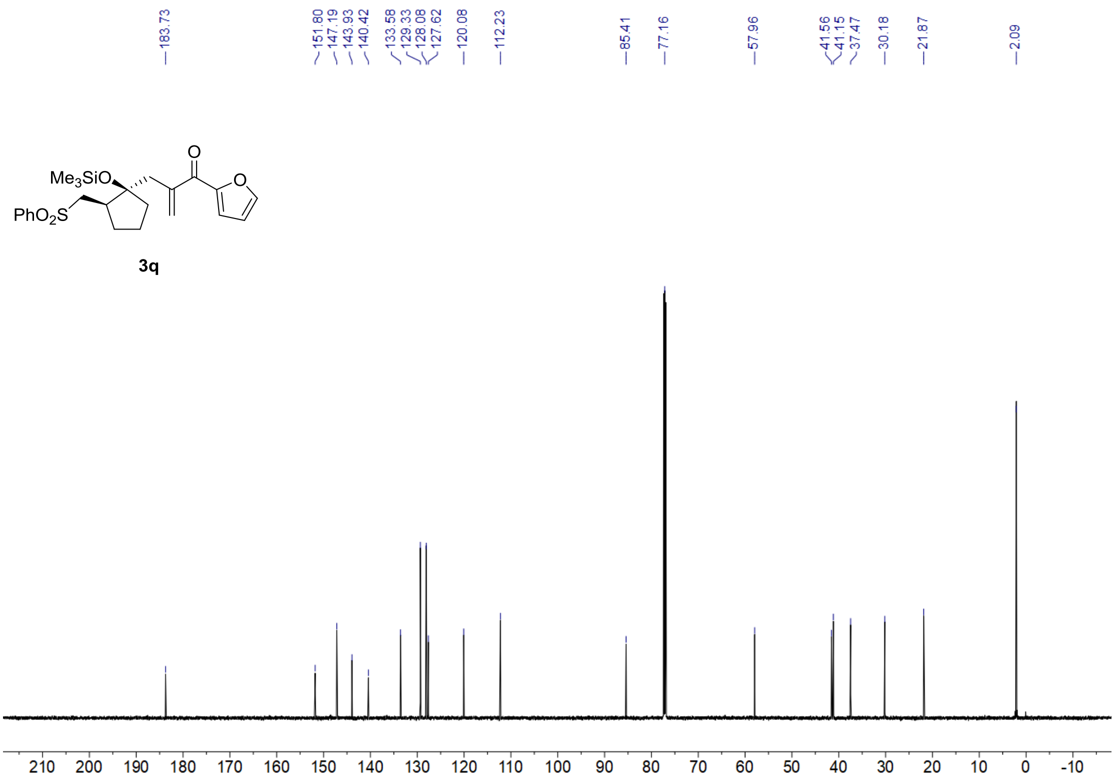

Supplementary Figure 88: ^{13}C NMR of **3q** (151 MHz, $CDCl_3$, 25 ºC)

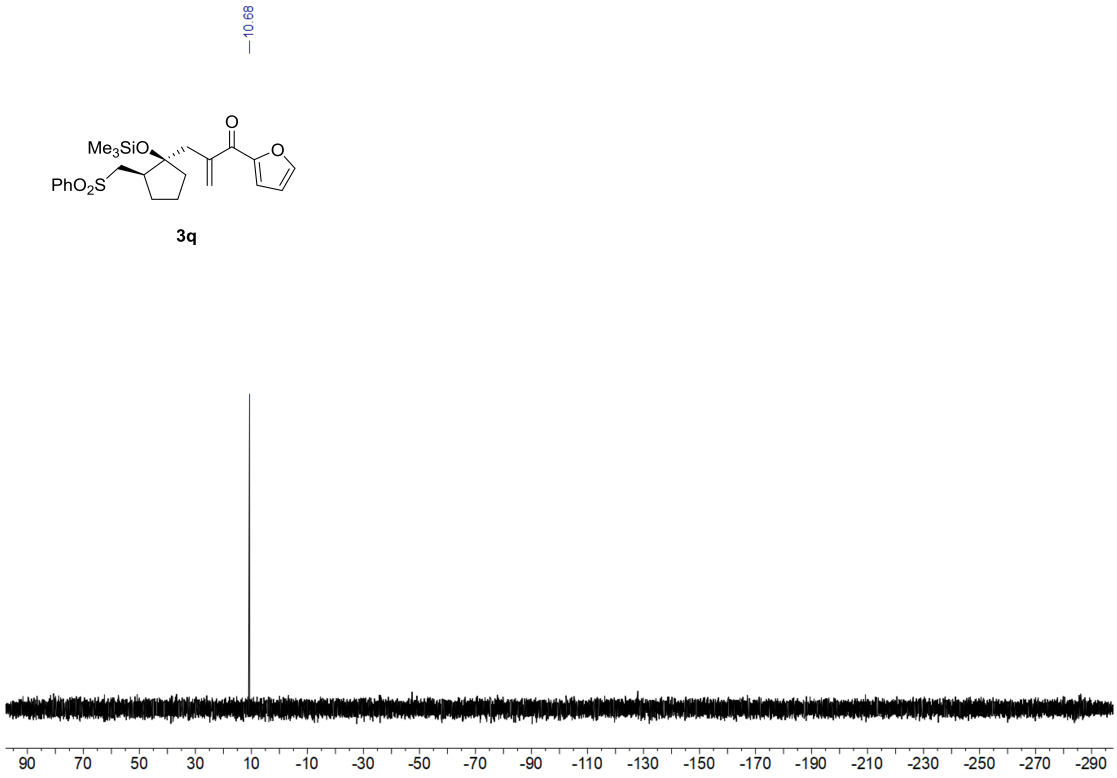

Supplementary Figure 89: ^{29}Si NMR of **3q** (119 MHz, $CDCl_3$, 25 ºC)

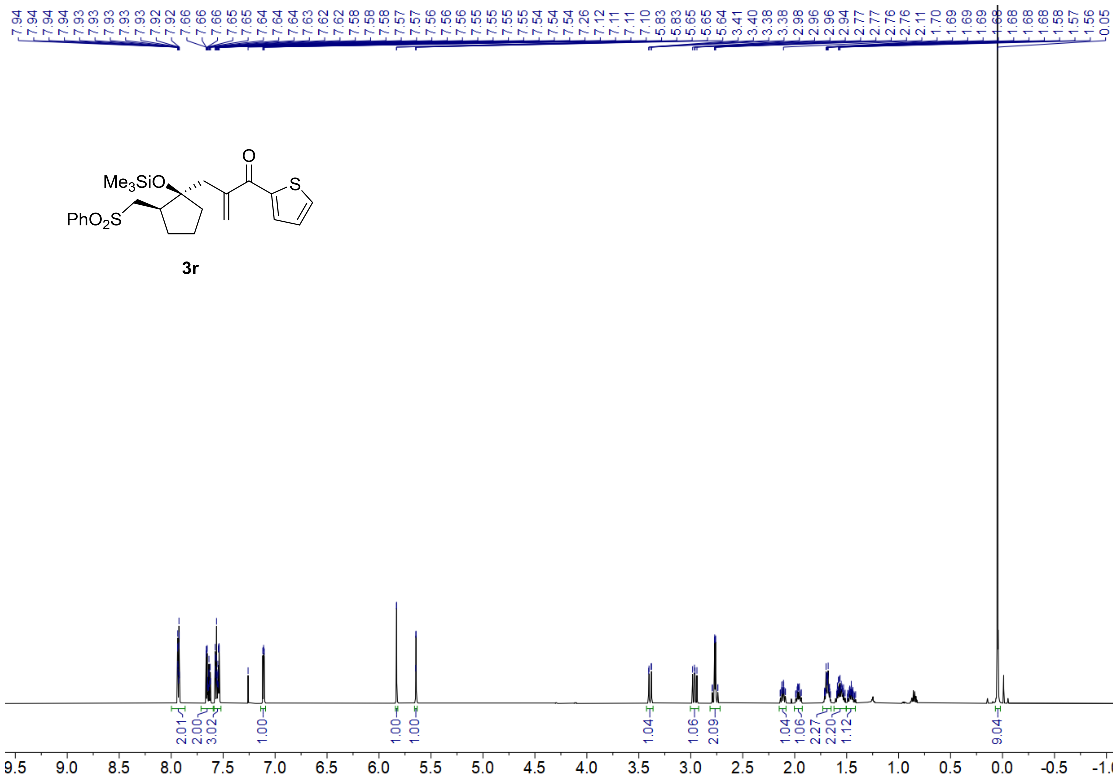

Supplementary Figure 90: ^{1}H NMR of **3r** (600 MHz, $CDCl_3$, 25 ºC)

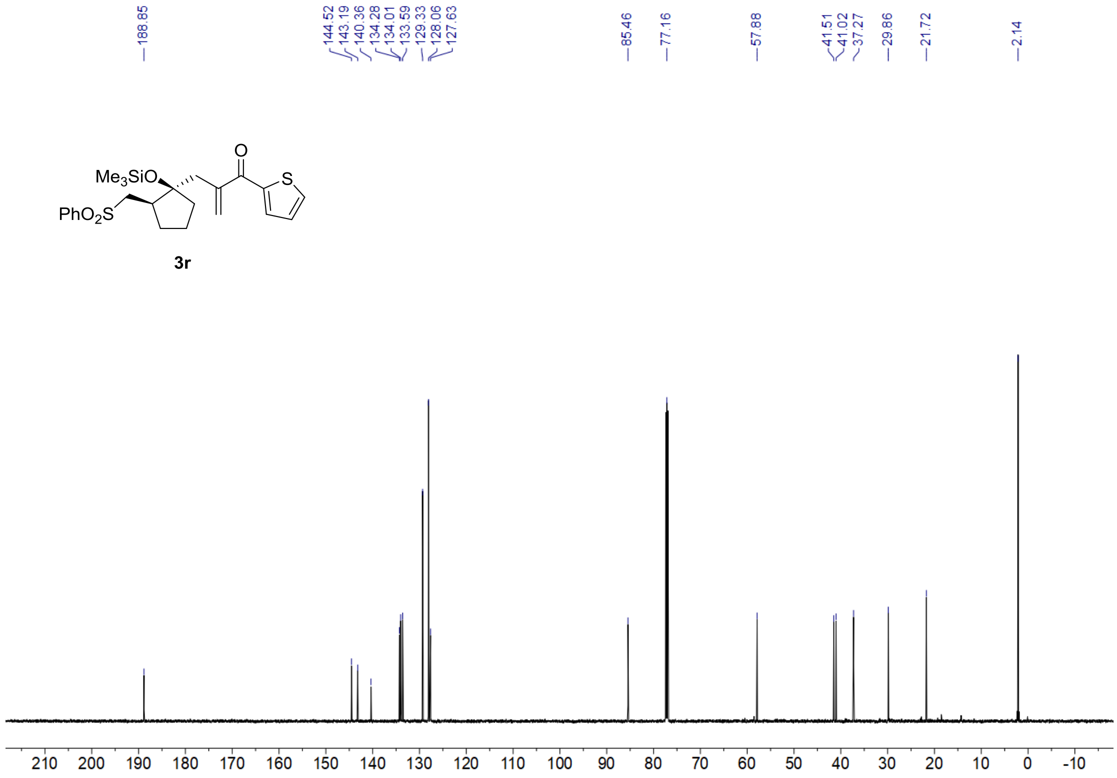

Supplementary Figure 91: ^{13}C NMR of **3r** (151 MHz, $CDCl_3$, 25 ºC)

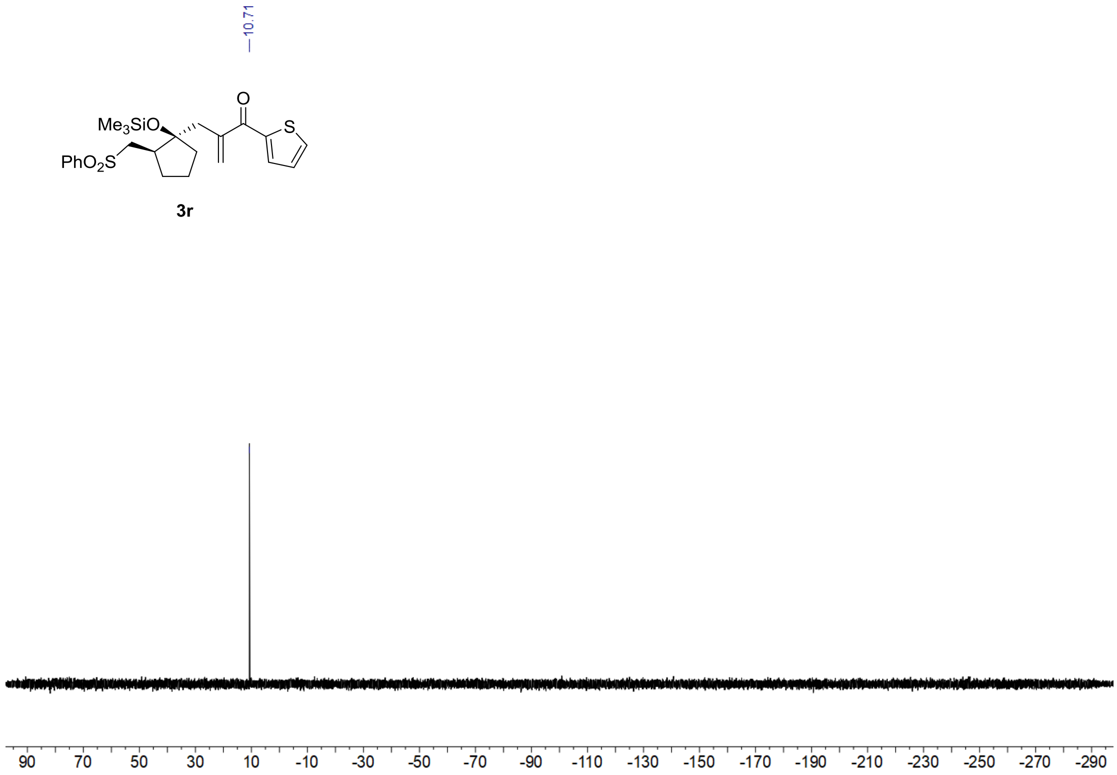

Supplementary Figure 92: ^{29}Si NMR of **3r** (119 MHz, $CDCl_3$, 25 ºC)

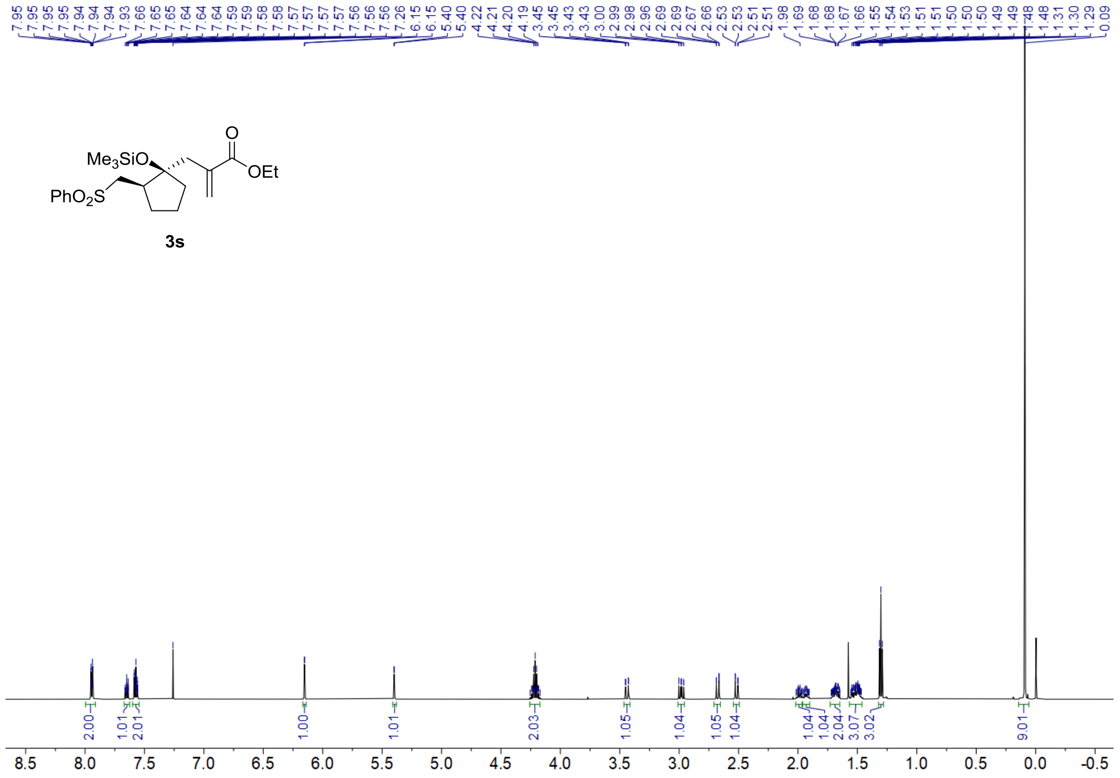

Supplementary Figure 93: ^{1}H NMR of **3s** (600 MHz, $CDCl_3$, 25 ºC)

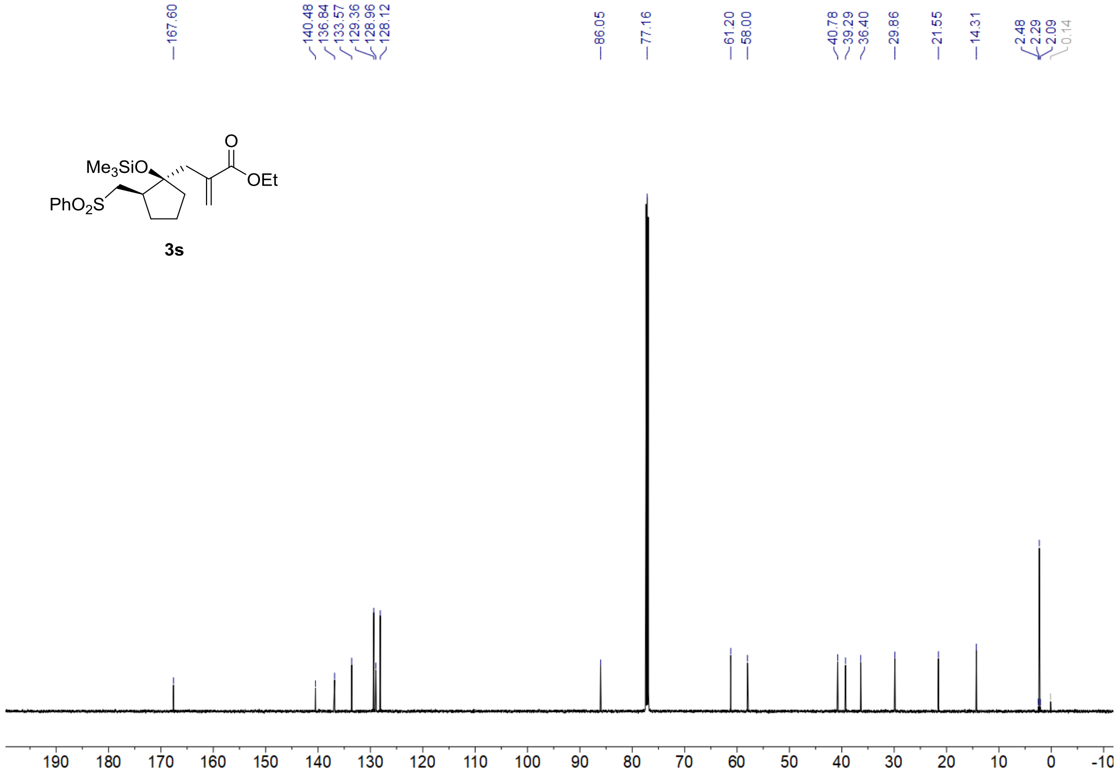

Supplementary Figure 94: ^{13}C NMR of **3s** (151 MHz, $CDCl_3$, 25 ºC)

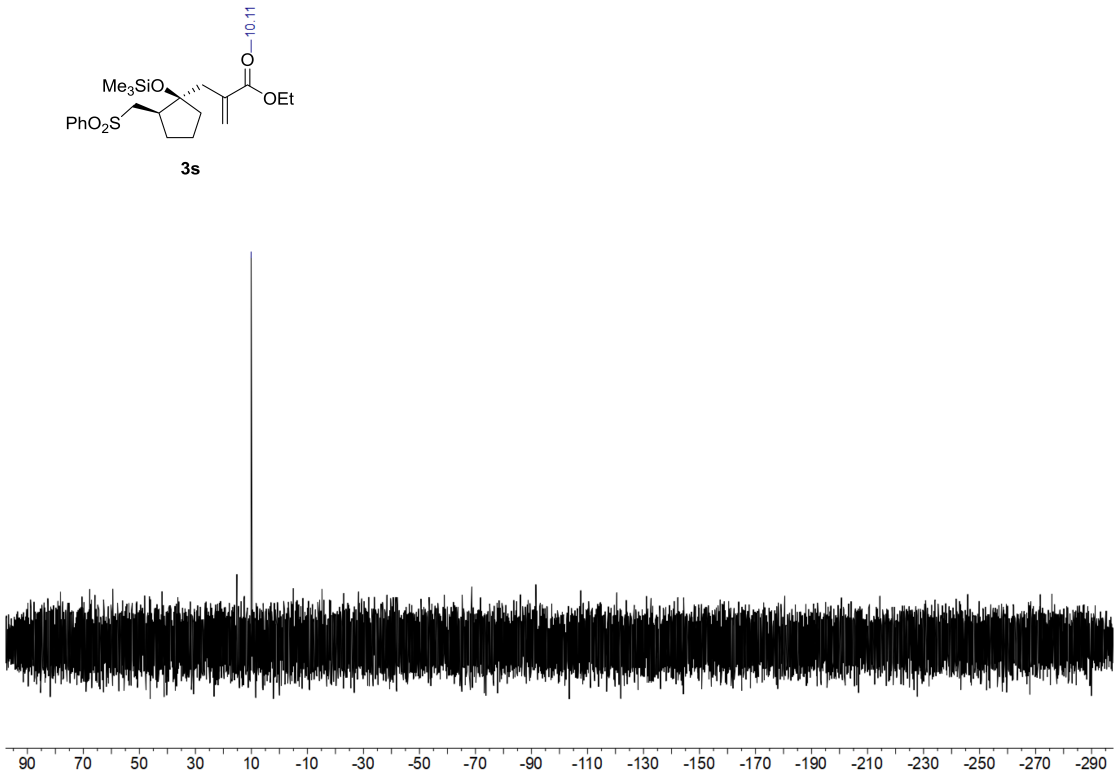

Supplementary Figure 95: ^{29}Si NMR of **3s** (119 MHz, $CDCl_3$, 25 ºC)

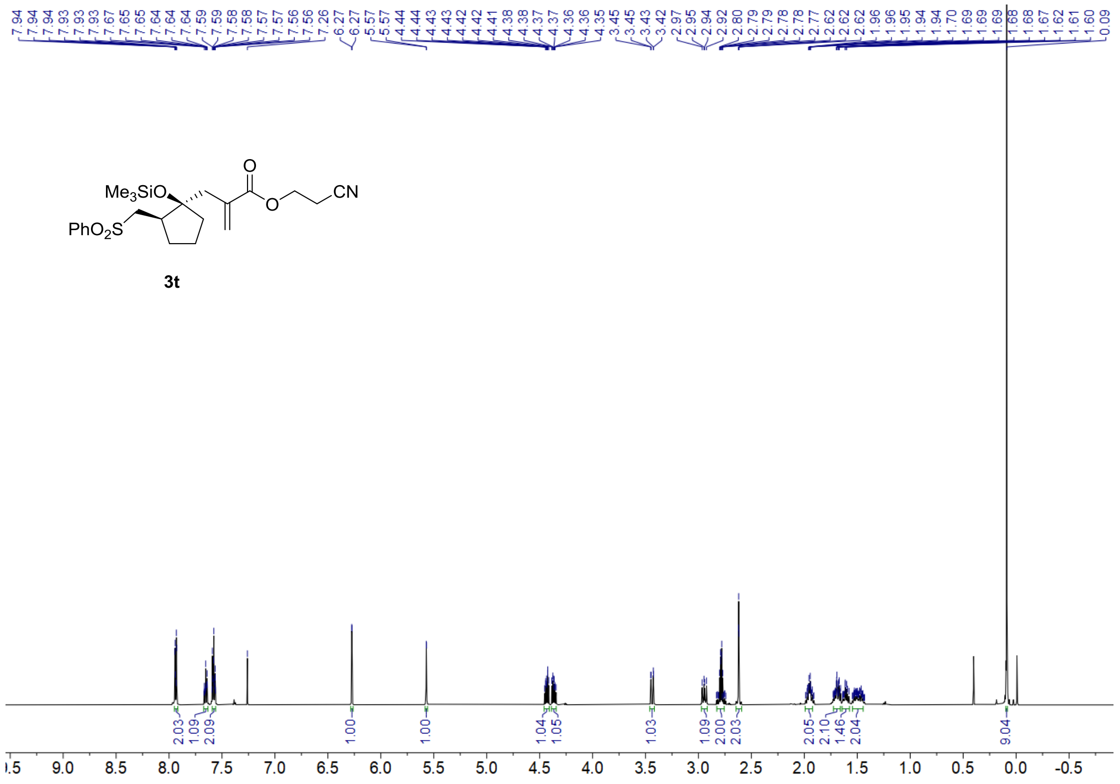

Supplementary Figure 96: ^{1}H NMR of **3t** (600 MHz, $CDCl_3$, 25 ºC)

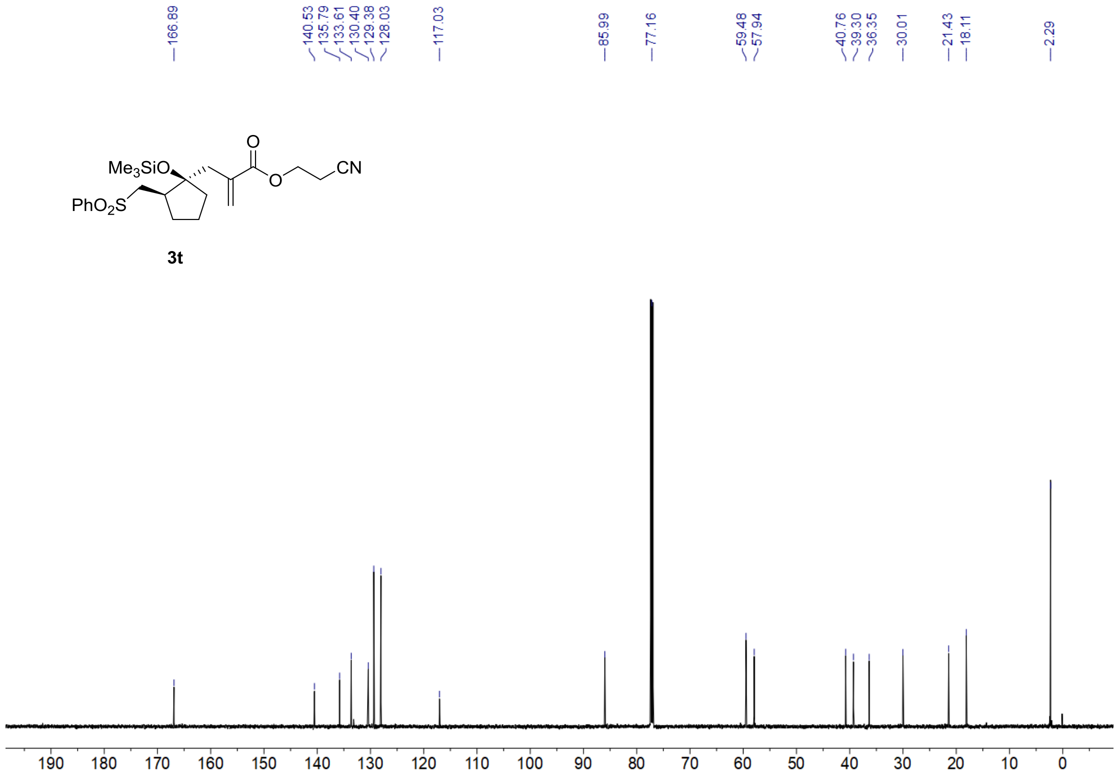

Supplementary Figure 97: ^{13}C NMR of **3t** (151 MHz, $CDCl_3$, 25 ºC)

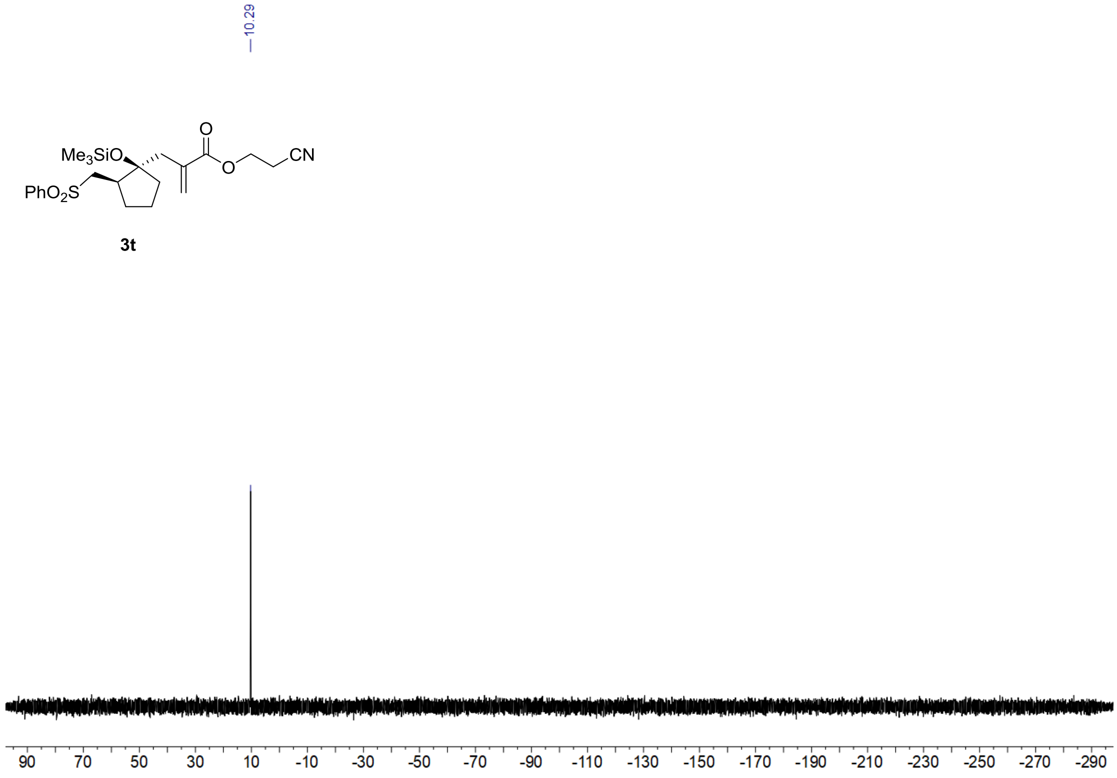

Supplementary Figure 98: ^{29}Si NMR of **3t** (119 MHz, $CDCl_3$, 25 ºC)

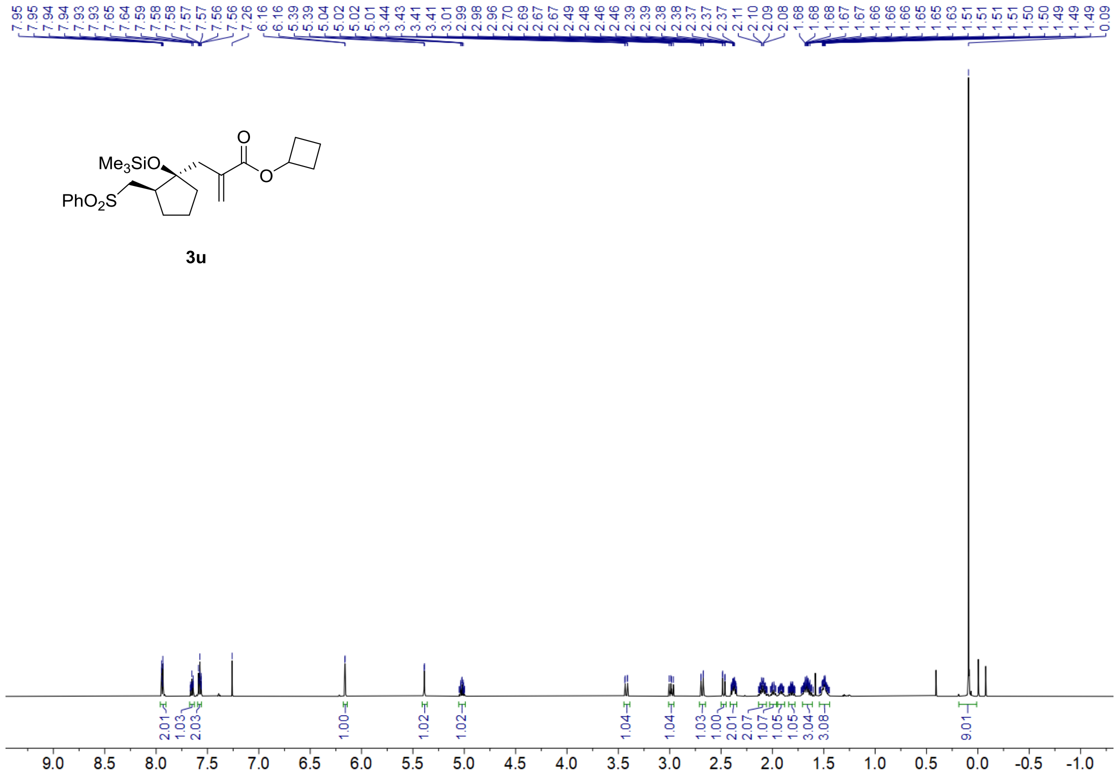

Supplementary Figure 99: ^{1}H NMR of **3u** (600 MHz, CDCl$_3$, 25 ºC)

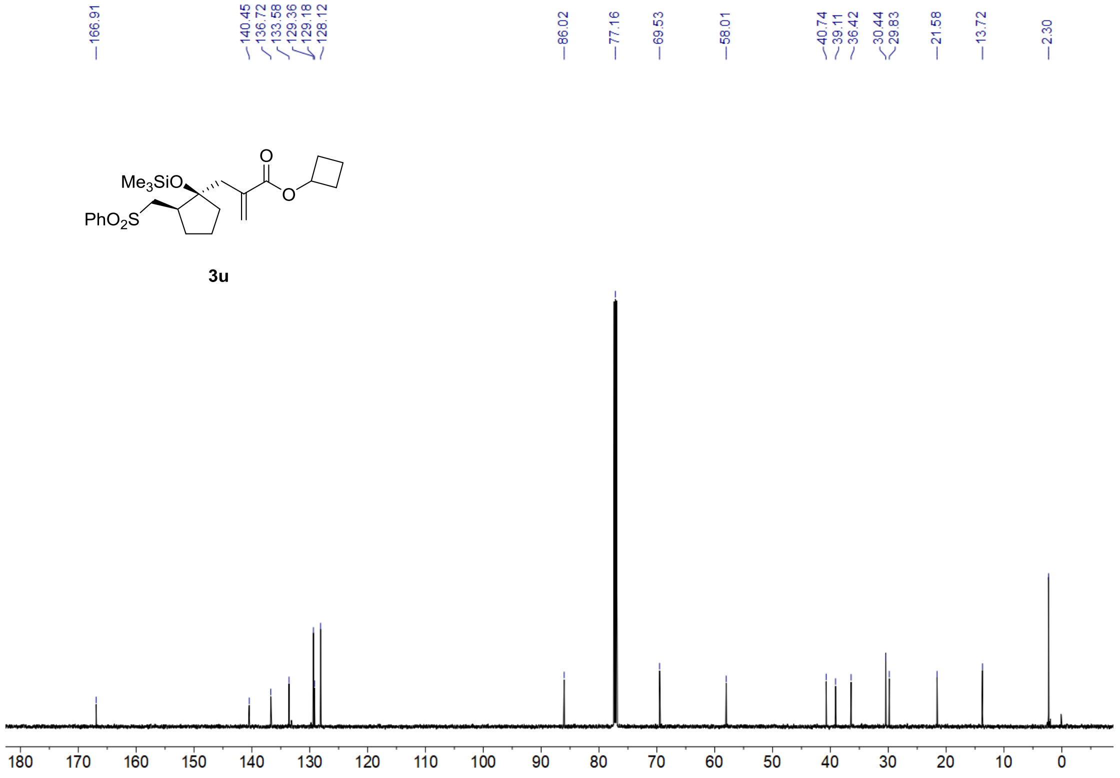

Supplementary Figure 100: ^{13}C NMR of **3u** (151 MHz, CDCl$_3$, 25 ºC)

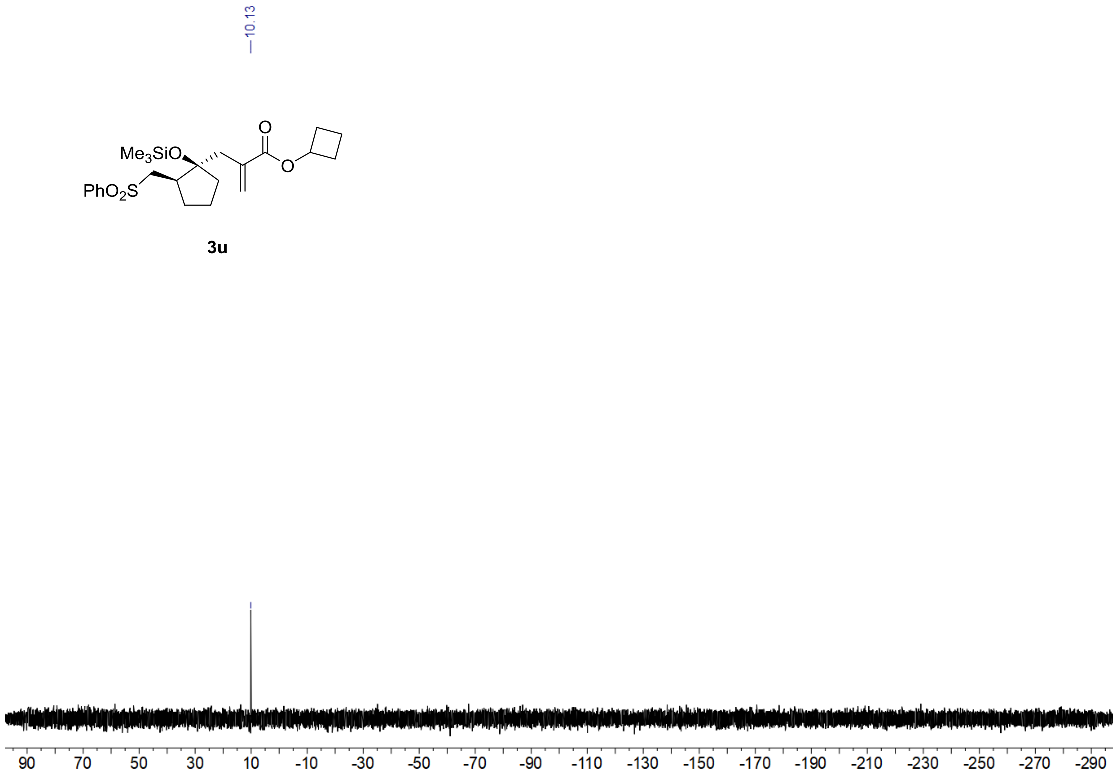

Supplementary Figure 101: ^{29}Si NMR of **3u** (119 MHz, $CDCl_3$, 25 ºC)

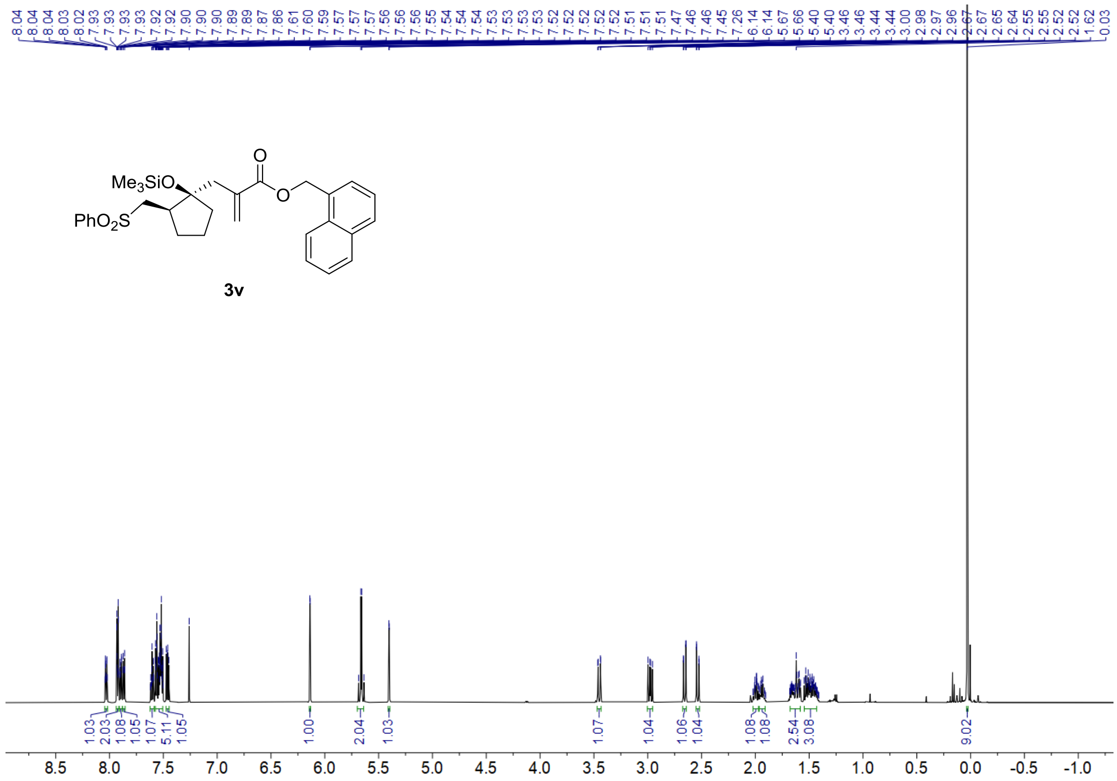

Supplementary Figure 102: ^{1}H NMR of **3v** (600 MHz, $CDCl_3$, 25 ºC)

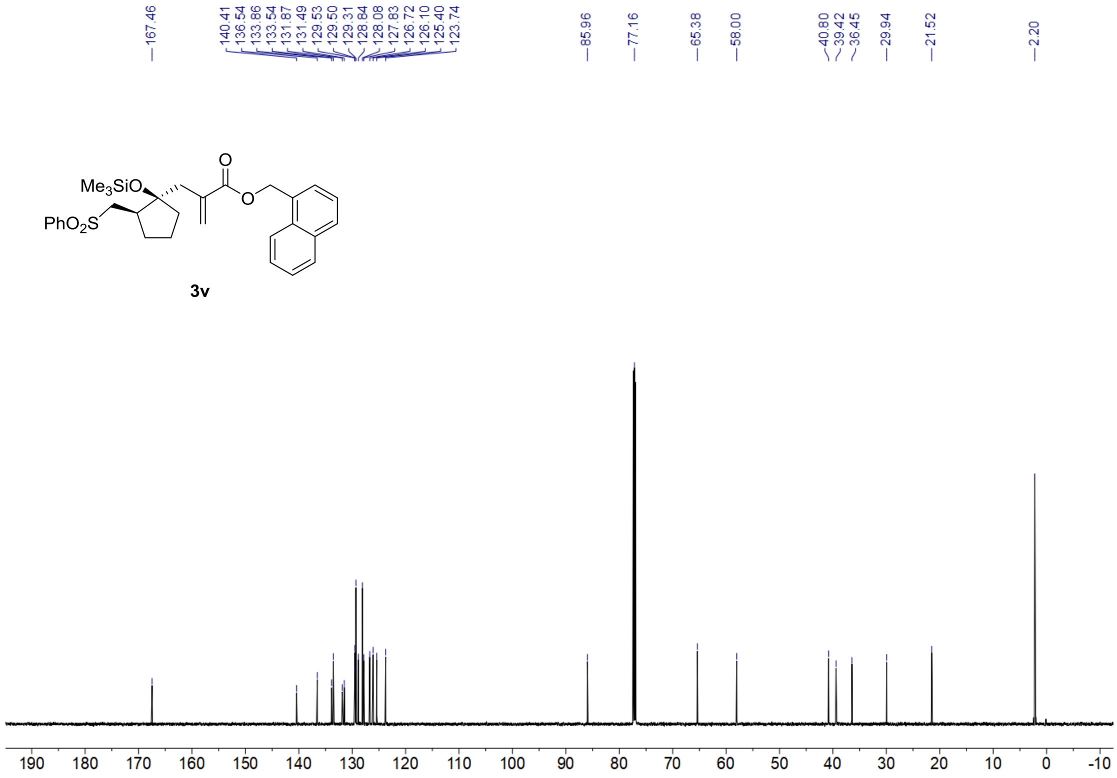

Supplementary Figure 103: ^{13}C NMR of **3v** (151 MHz, $CDCl_3$, 25 ºC)

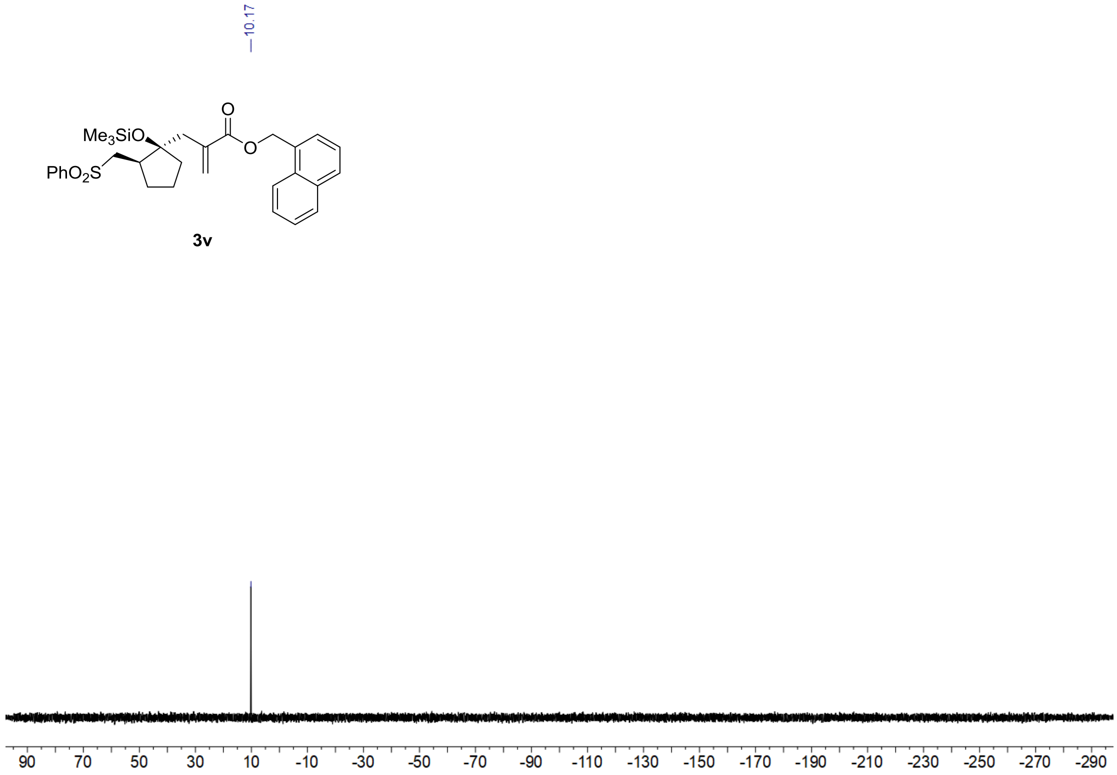

Supplementary Figure 104: ^{29}Si NMR of **3v** (119 MHz, $CDCl_3$, 25 ºC)

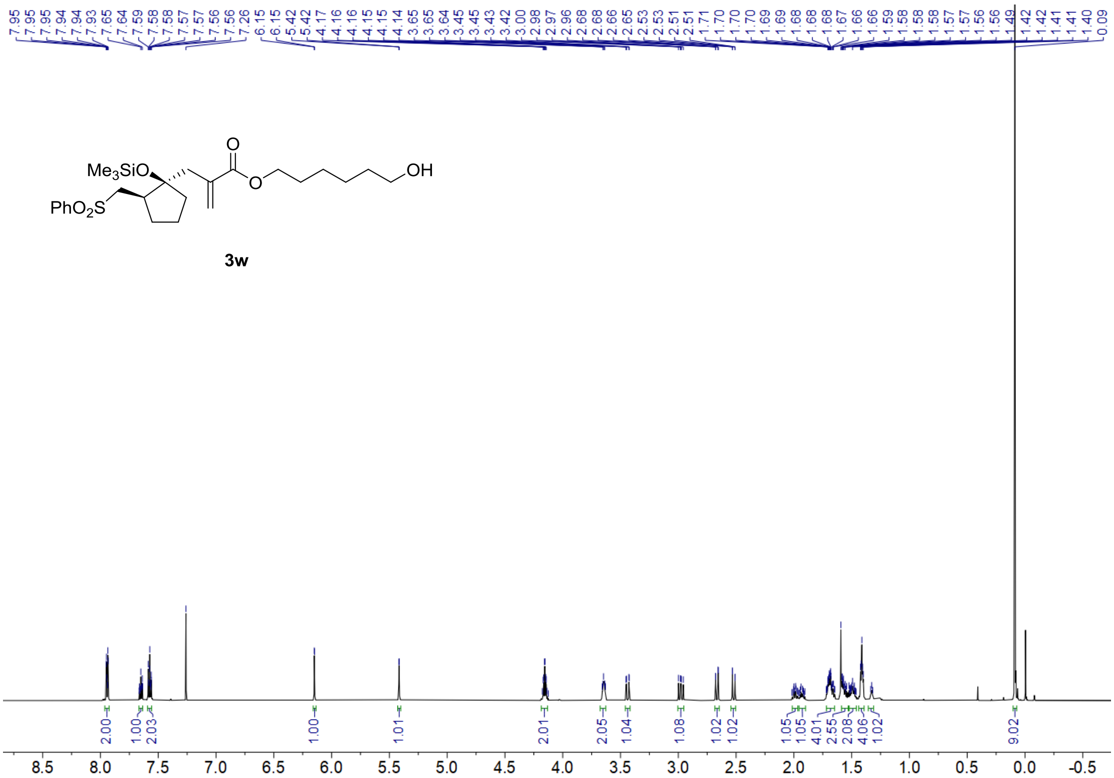

Supplementary Figure 105: ^{1}H NMR of **3w** (600 MHz, $CDCl_3$, 25 ºC)

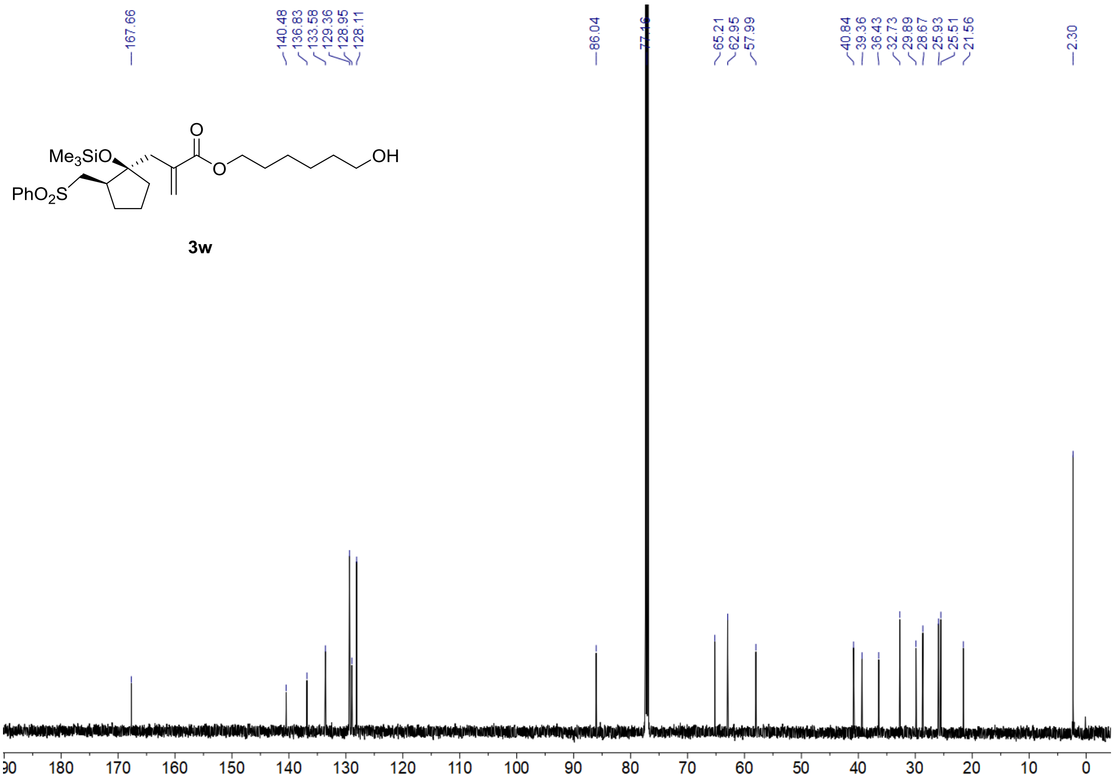

Supplementary Figure 106: ^{13}C NMR of **3w** (151 MHz, $CDCl_3$, 25 ºC)

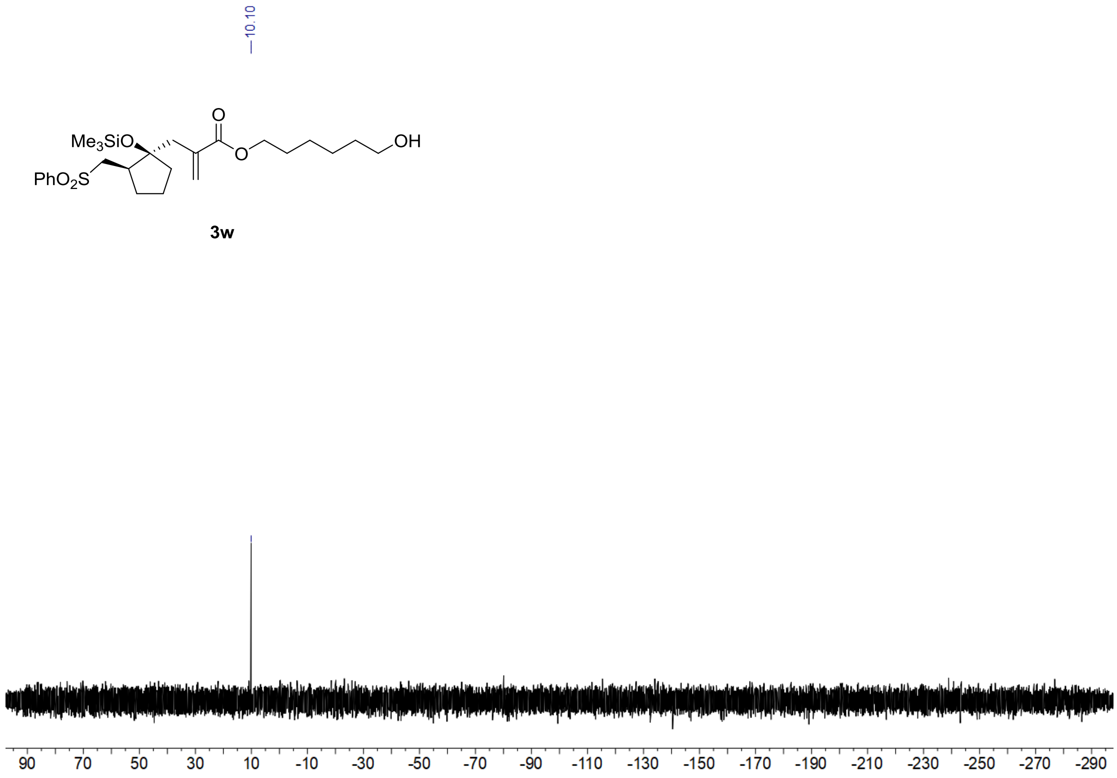

Supplementary Figure 107: ^{29}Si NMR of **3w** (119 MHz, CDCl$_3$, 25 ºC)

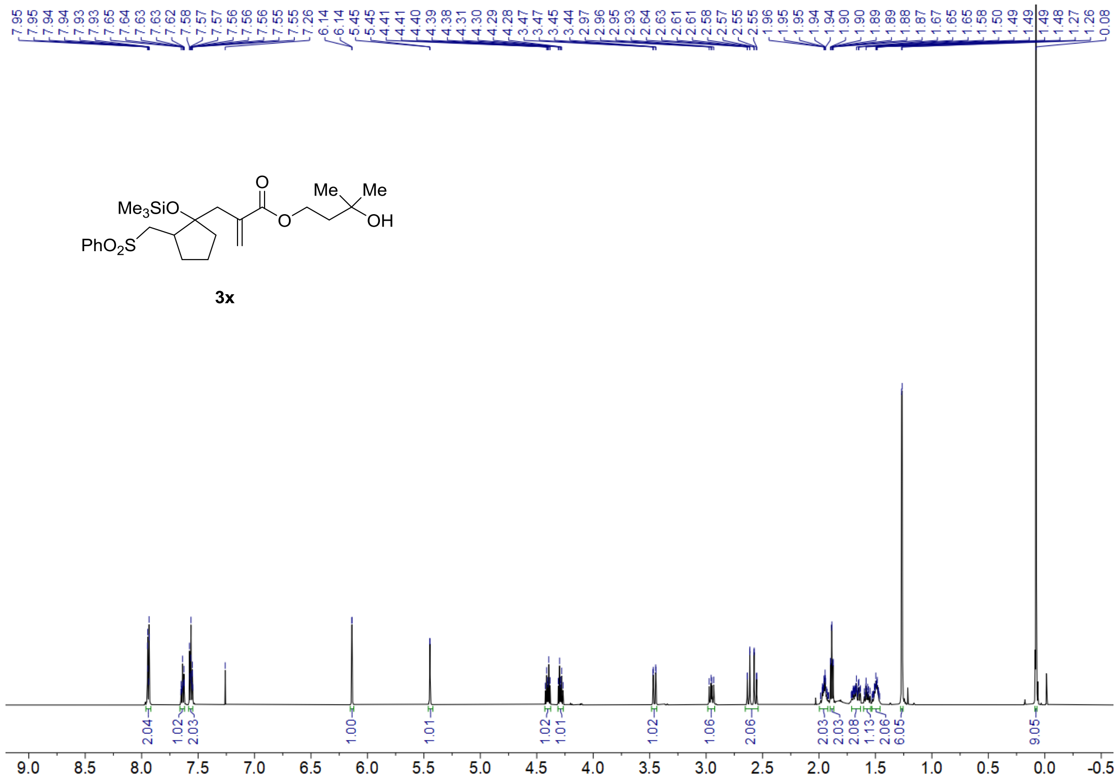

Supplementary Figure 108: ^{1}H NMR of **3x** (600 MHz, CDCl$_3$, 25 ºC)

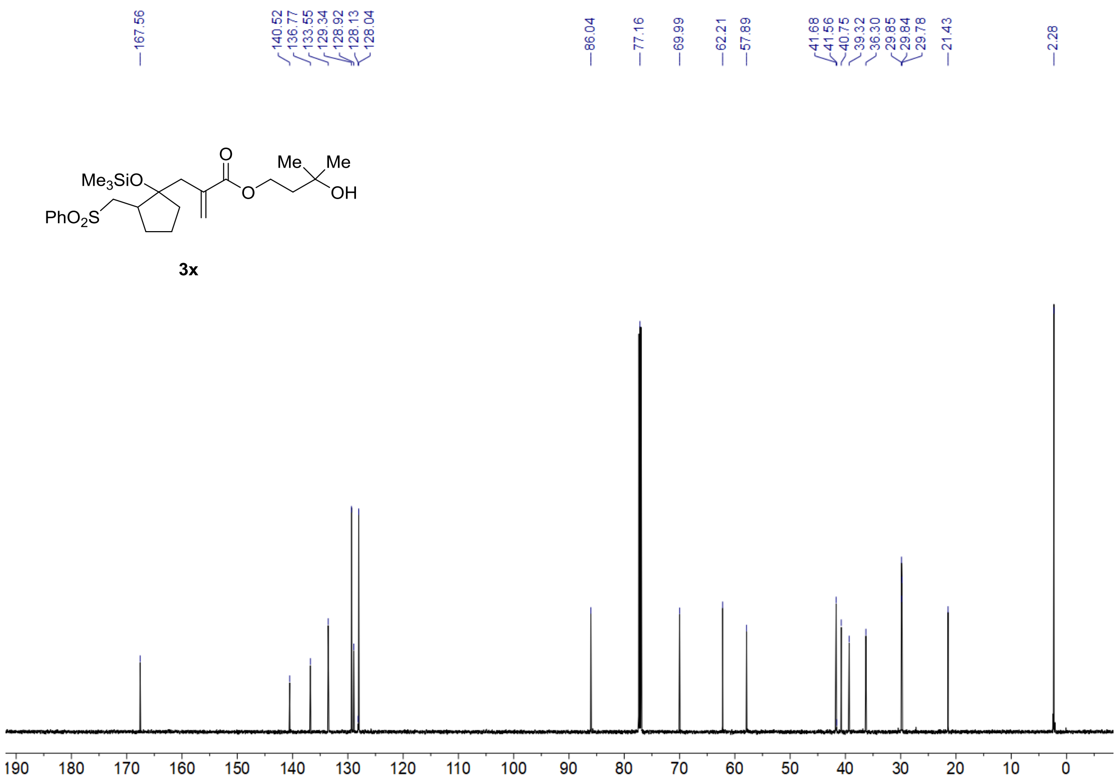

Supplementary Figure 109: ^{13}C NMR of **3x** (151 MHz, $CDCl_3$, 25 ºC)

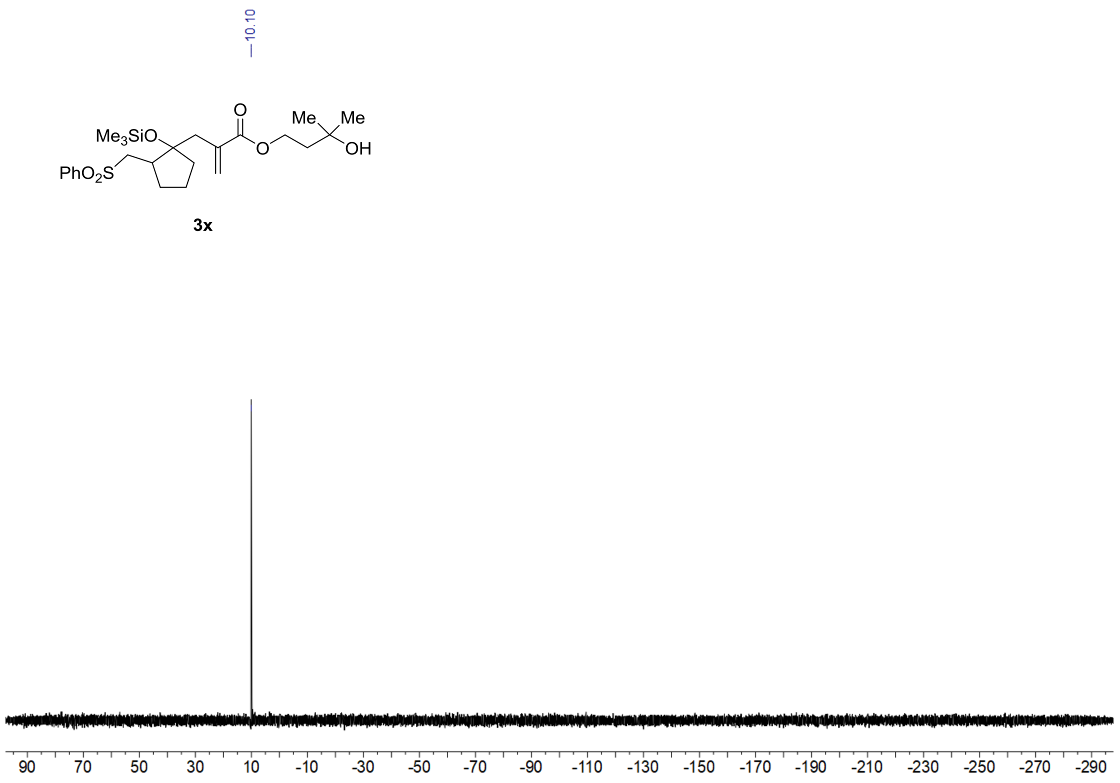

Supplementary Figure 110: ^{29}Si NMR of **3x** (119 MHz, $CDCl_3$, 25 ºC)

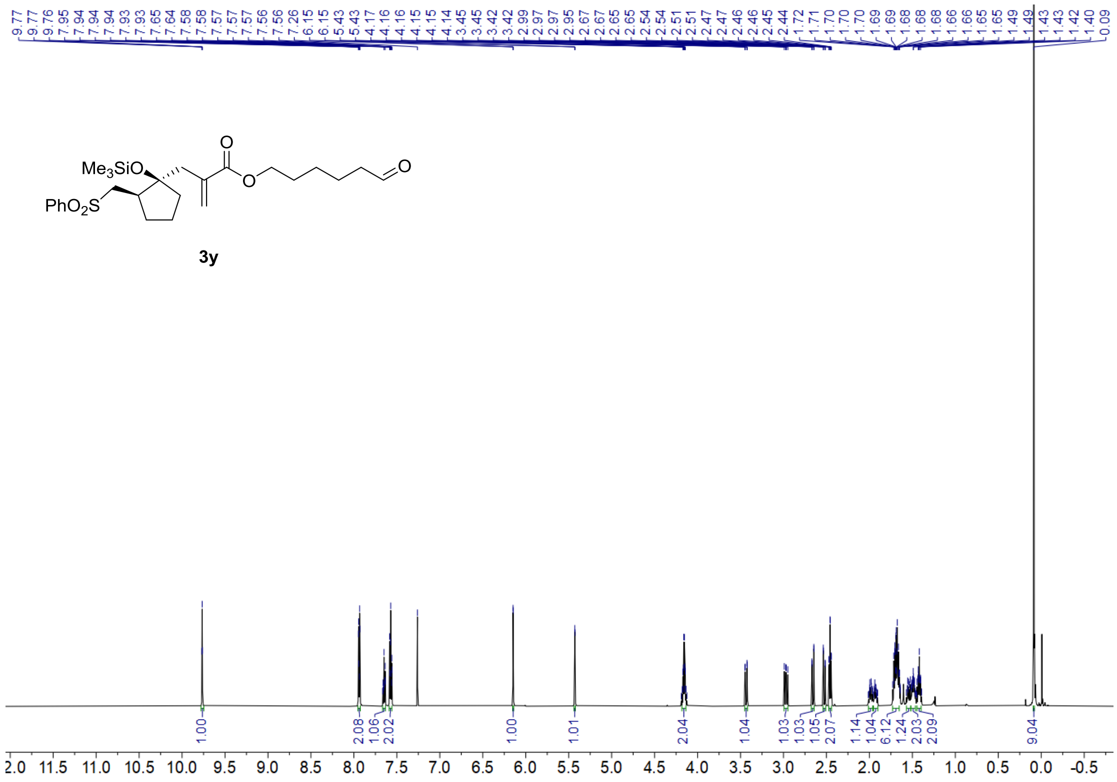

Supplementary Figure 111: ^{1}H NMR of **3y** (600 MHz, $CDCl_3$, 25 ºC)

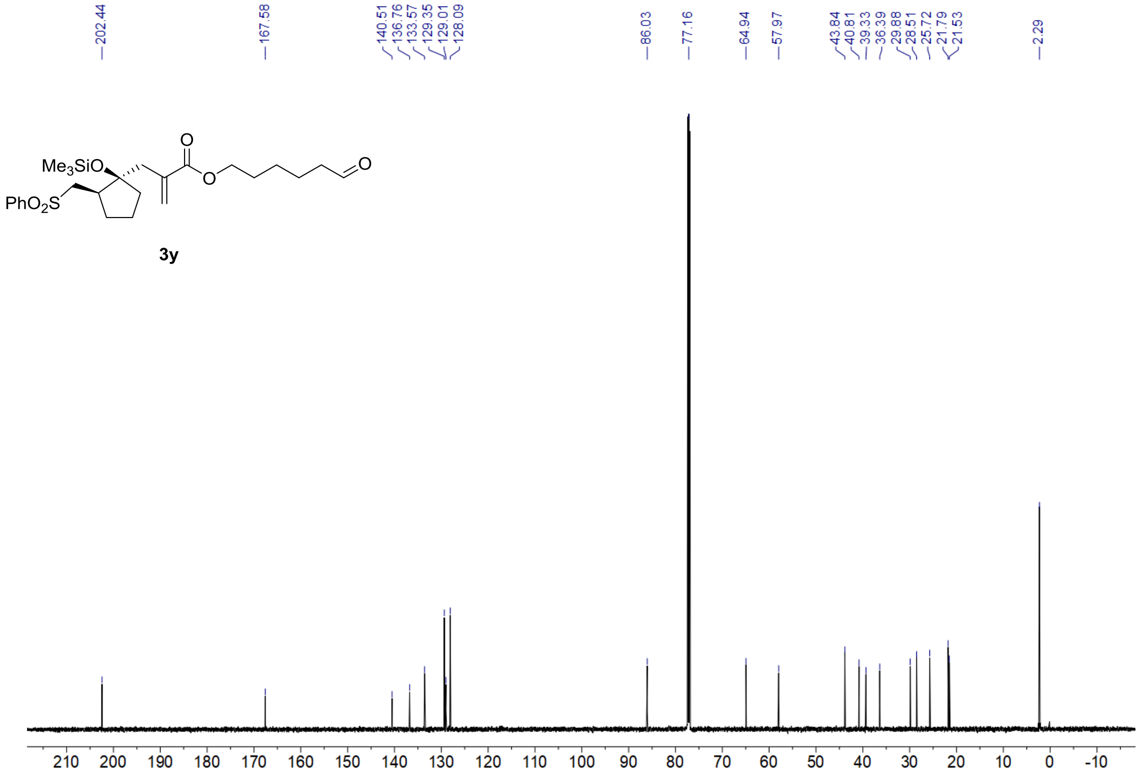

Supplementary Figure 112: ^{13}C NMR of **3y** (151 MHz, $CDCl_3$, 25 ºC)

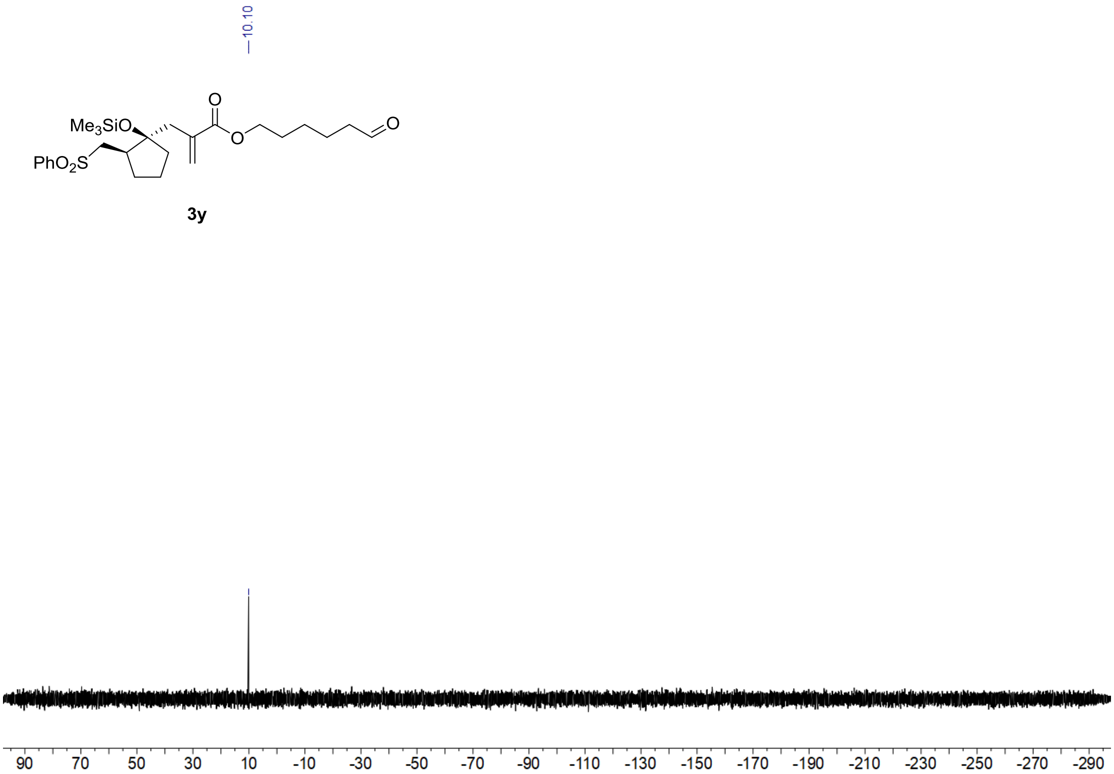

Supplementary Figure 113: ^{29}Si NMR of **3y** (119 MHz, $CDCl_3$, 25 ºC)

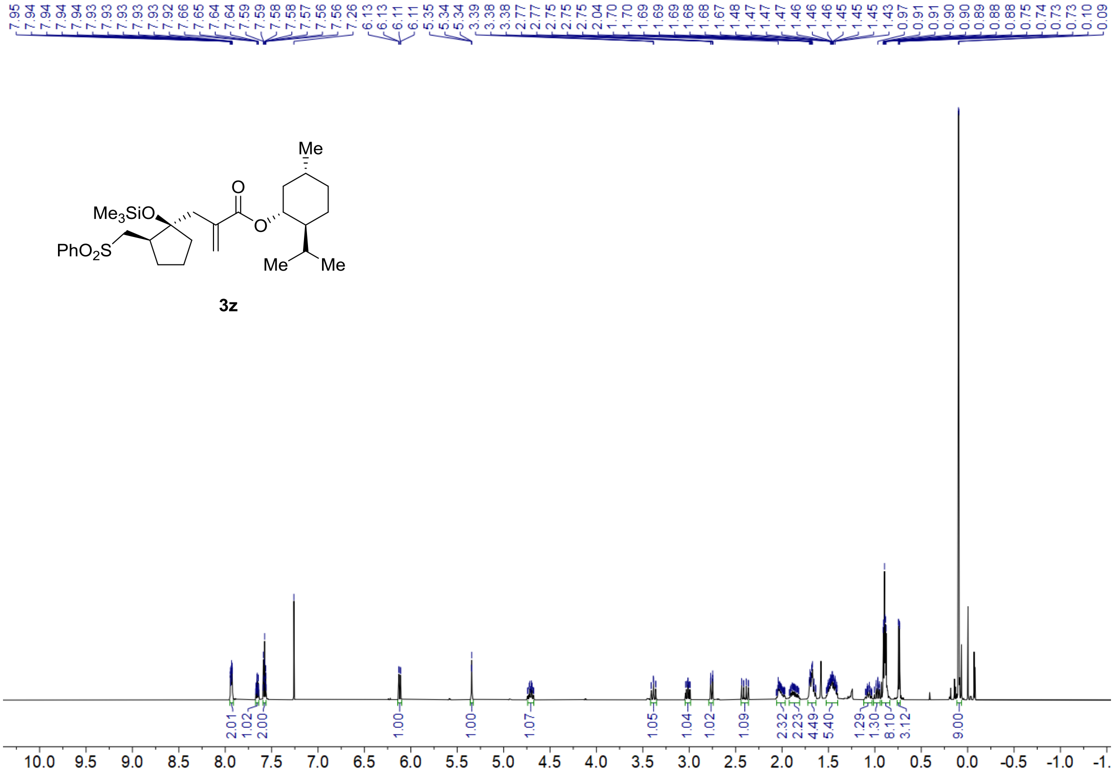

Supplementary Figure 114: ^{1}H NMR of **3z** (600 MHz, $CDCl_3$, 25 ºC)

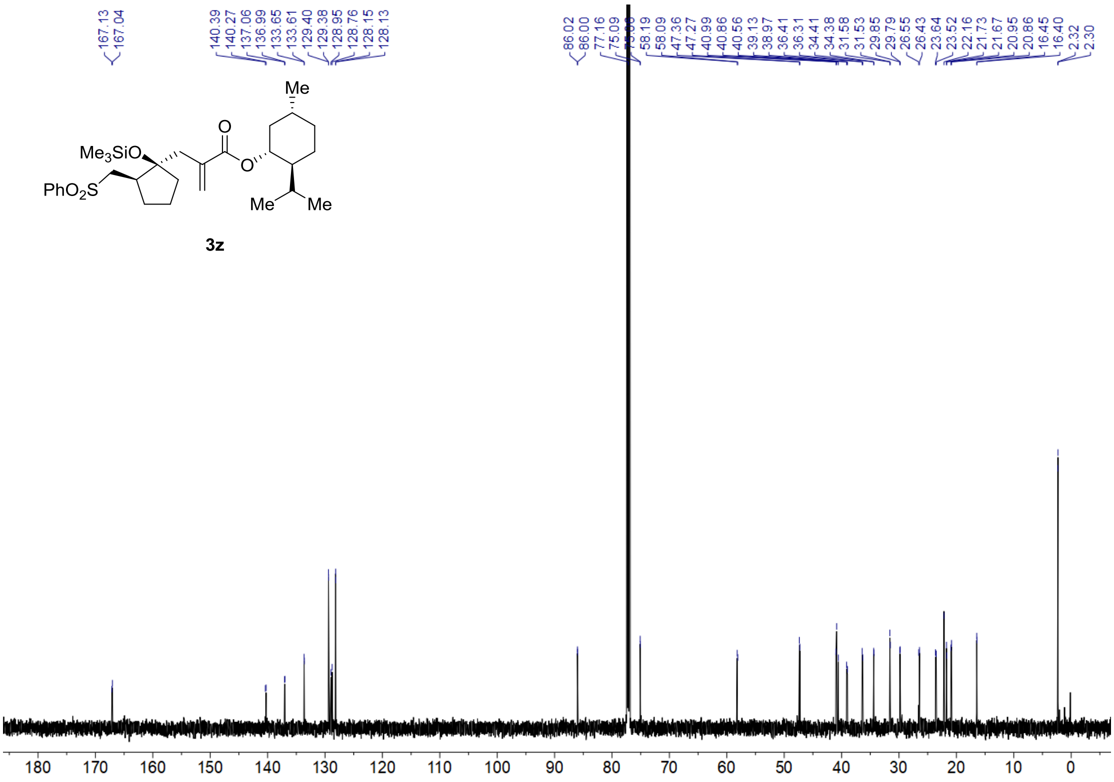

Supplementary Figure 115: ^{13}C NMR of **3z** (151 MHz, $CDCl_3$, 25 ºC)

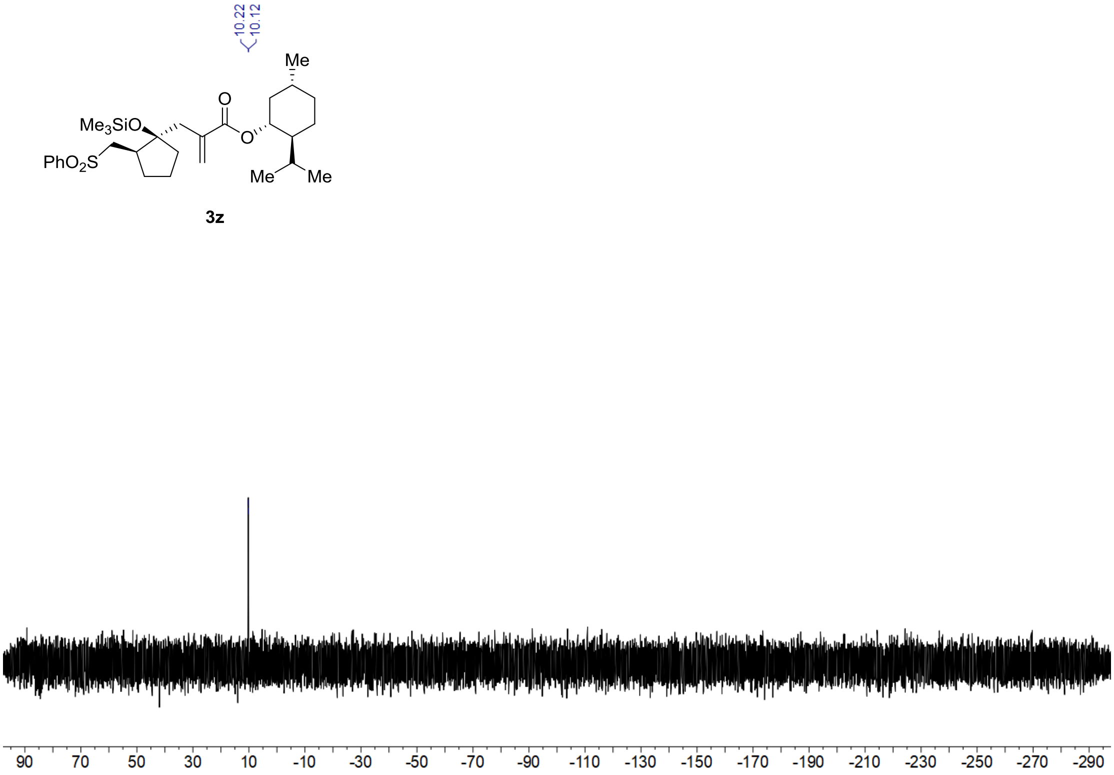

Supplementary Figure 116: ^{29}Si NMR of **3z** (119 MHz, $CDCl_3$, 25 ºC)

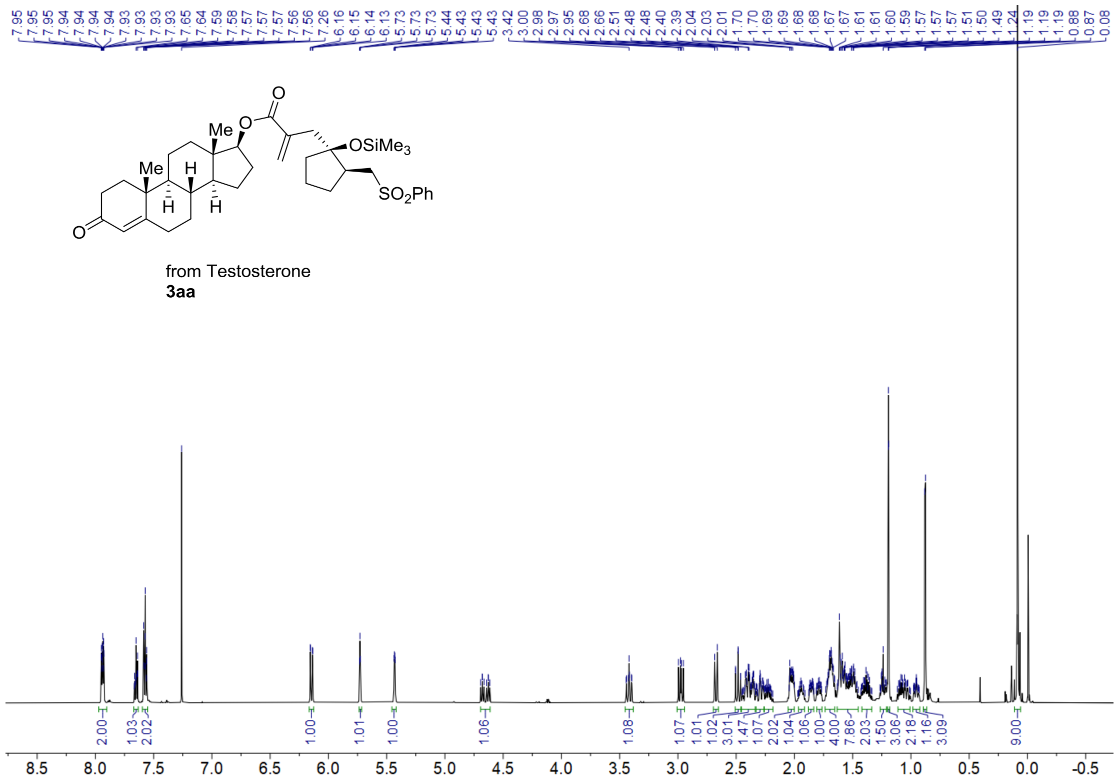

Supplementary Figure 117: ^{1}H NMR of **3aa** (600 MHz, CDCl$_3$, 25 ºC)

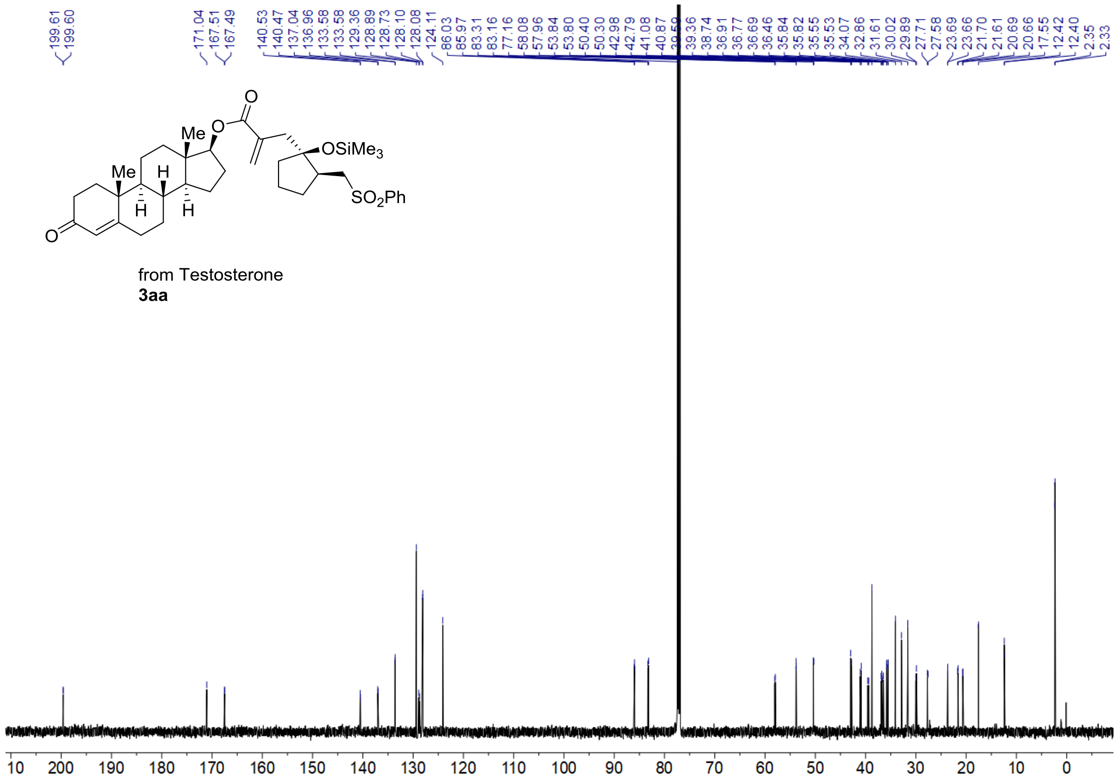

Supplementary Figure 118: ^{13}C NMR of **3aa** (151 MHz, CDCl$_3$, 25 ºC)

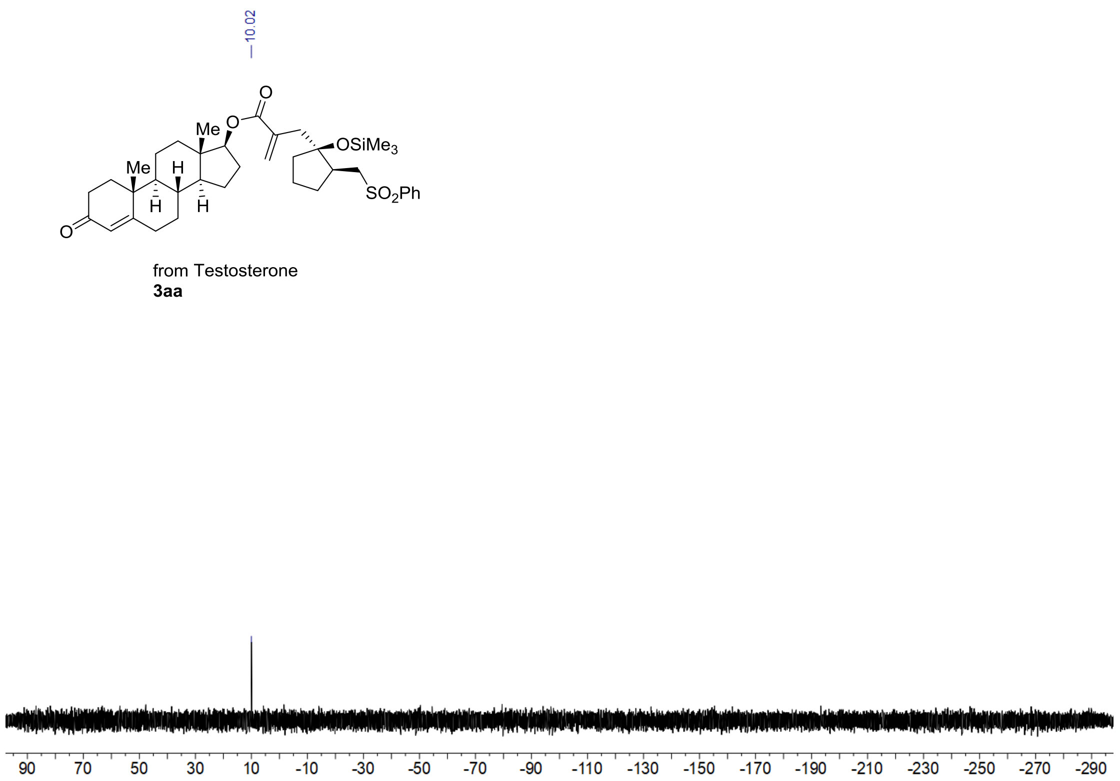

Supplementary Figure 119: ^{29}Si NMR of **3aa** (119 MHz, $CDCl_3$, 25 ºC)

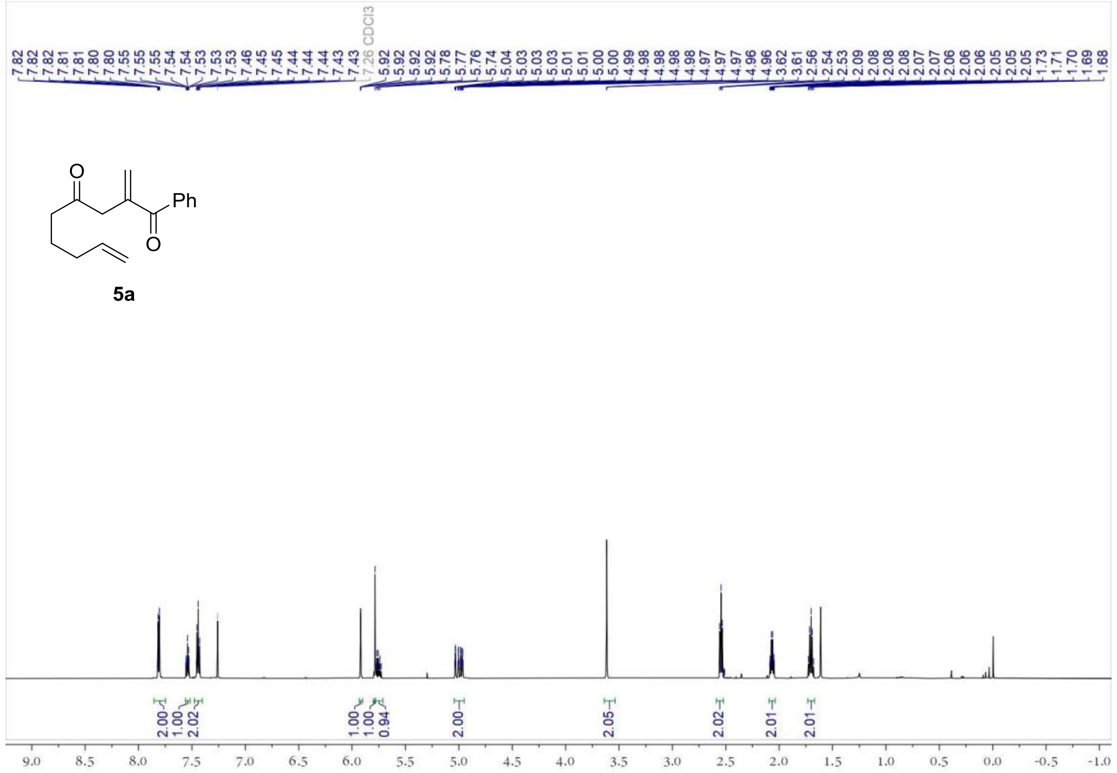

Supplementary Figure 120: ^{1}H NMR of **5a** (600 MHz, $CDCl_3$, 25 ºC)

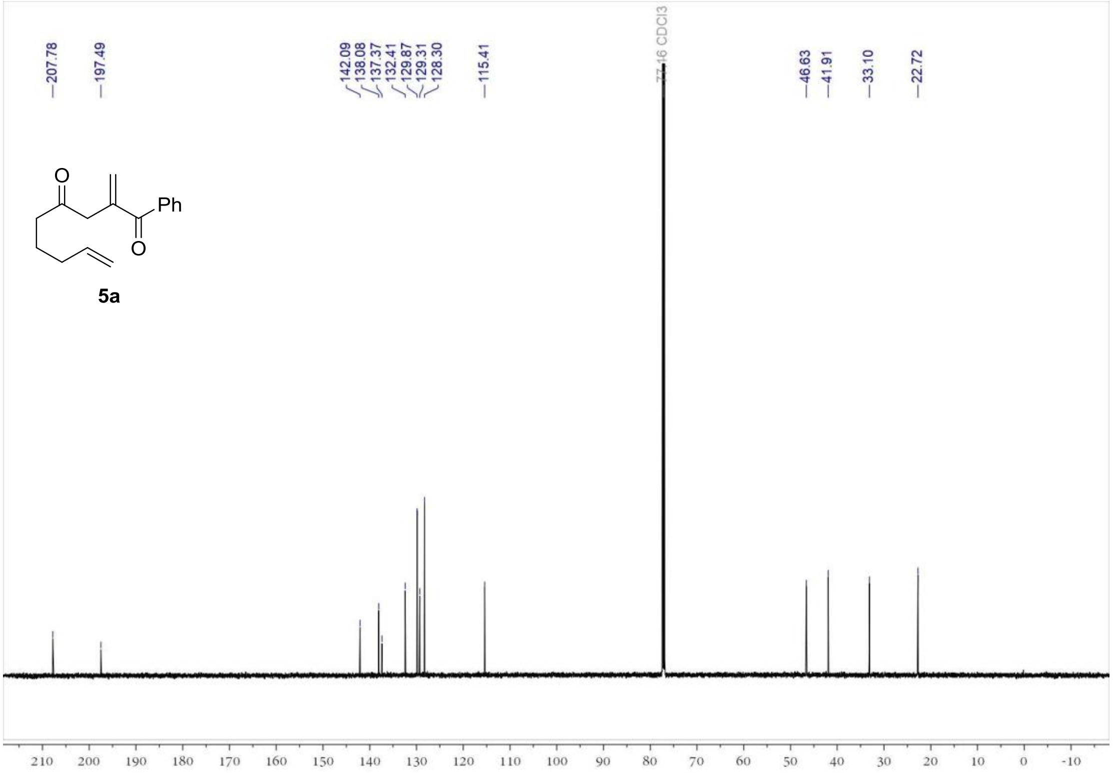

Supplementary Figure 121: ^{13}C NMR of **5a** (600 MHz, $CDCl_3$, 25 ºC)

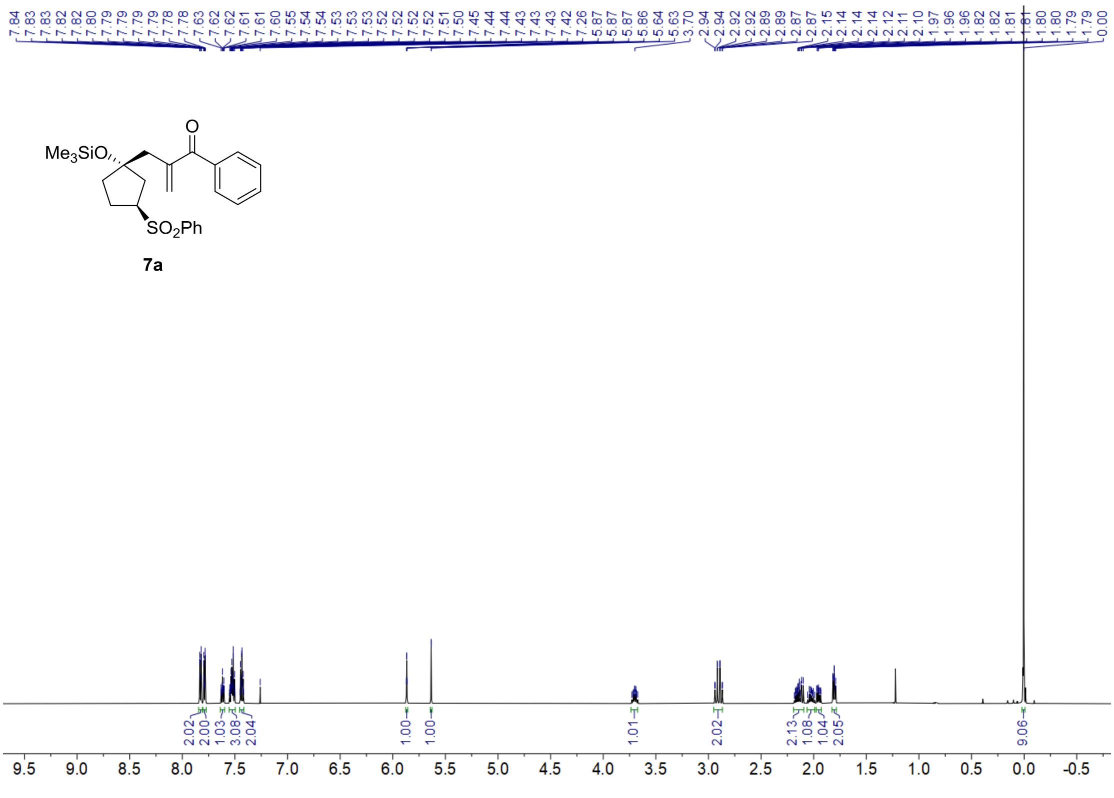

Supplementary Figure 122: ^{1}H NMR of **7a** (600 MHz, $CDCl_3$, 25 ºC)

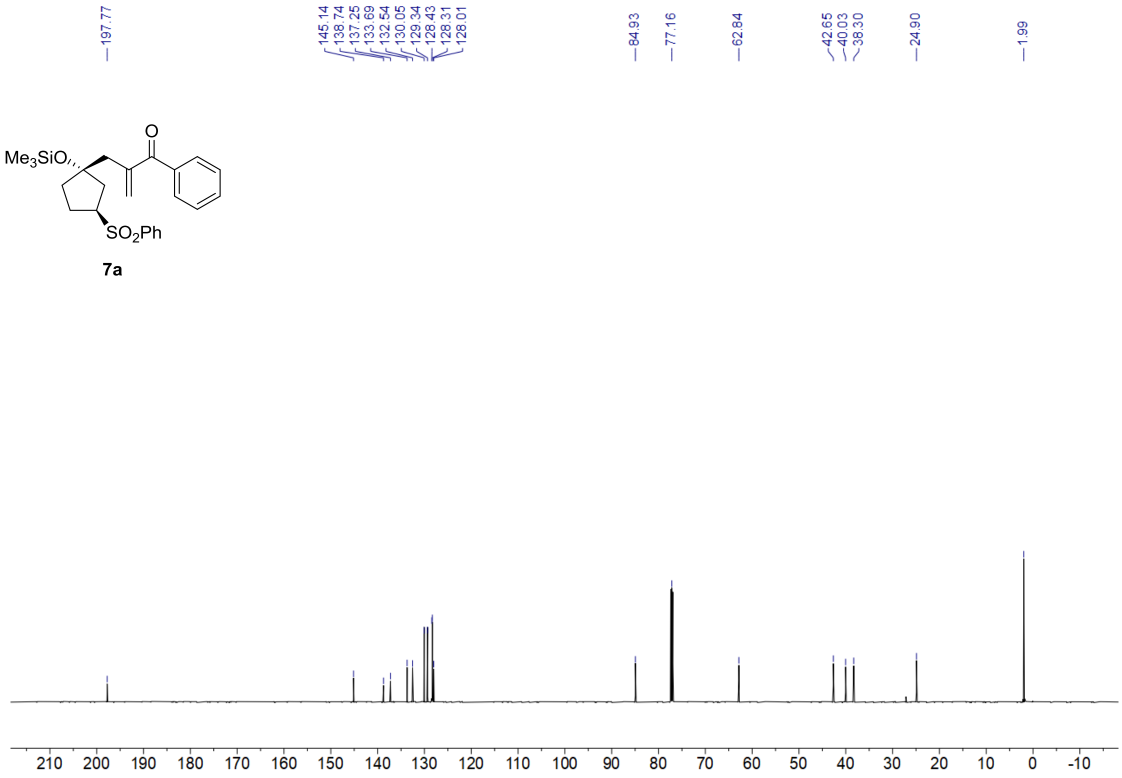

Supplementary Figure 123: ^{13}C NMR of **7a** (151 MHz, $CDCl_3$, 25 ºC)

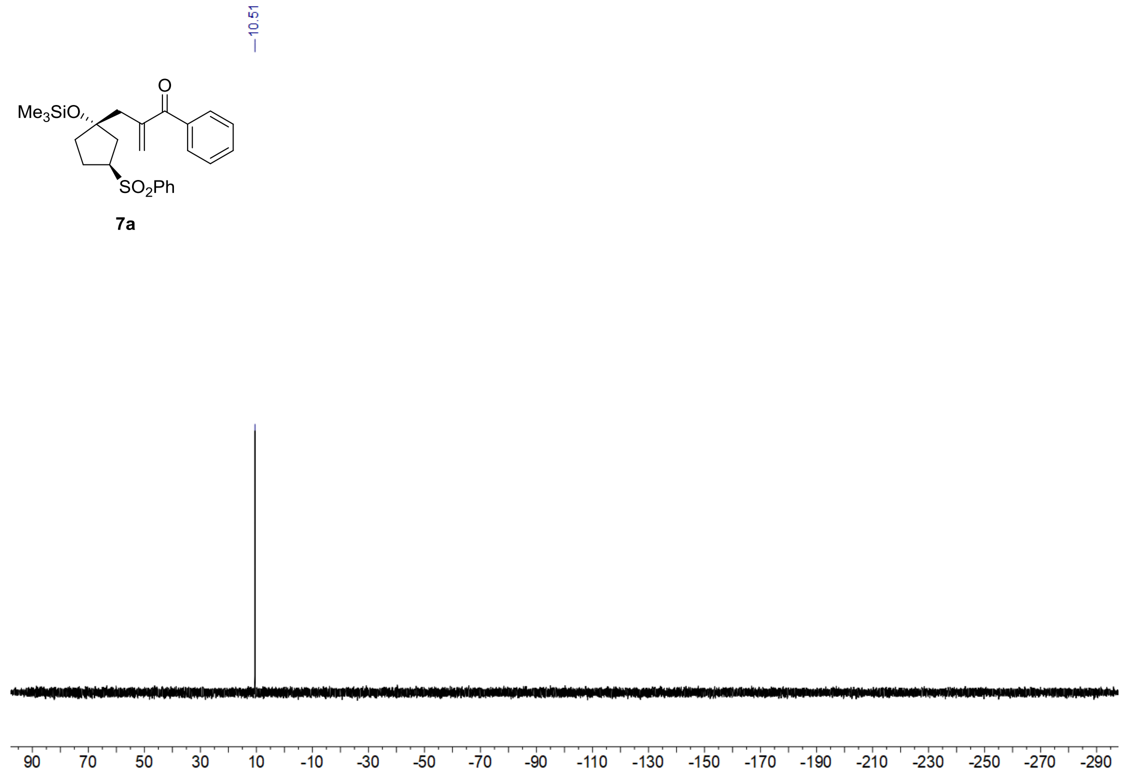

Supplementary Figure 124: ^{29}Si NMR of **7a** (119 MHz, $CDCl_3$, 25 ºC)

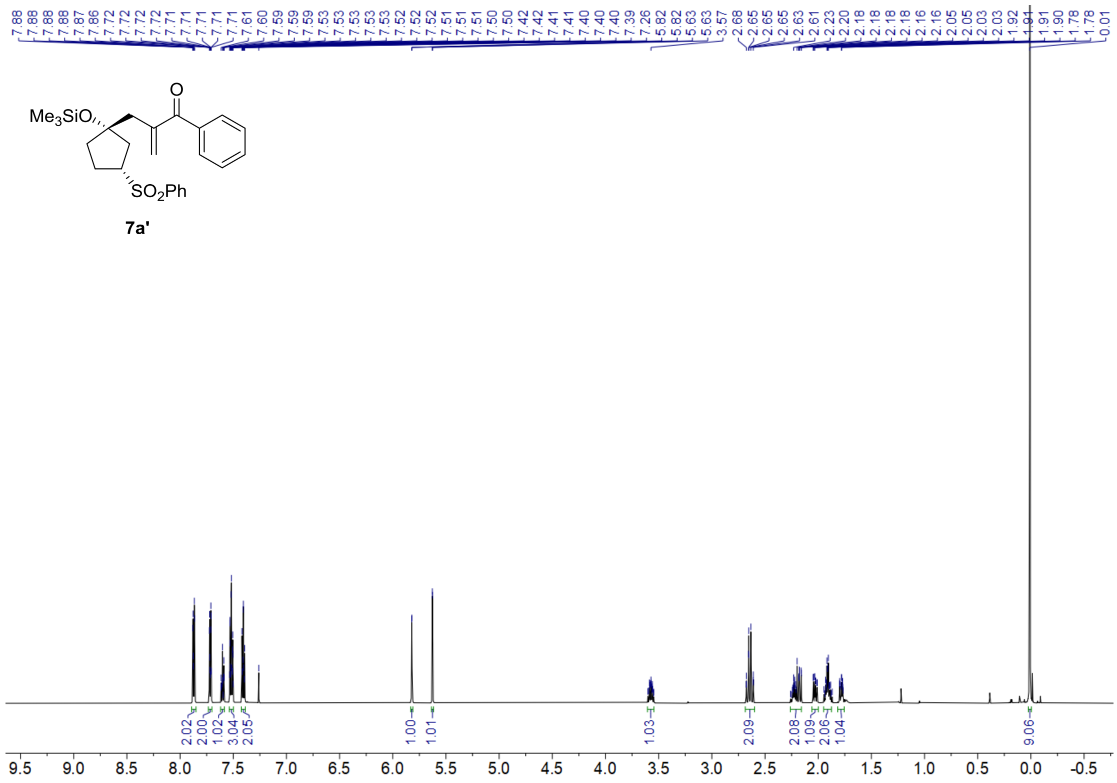

Supplementary Figure 125: ^{1}H NMR of **7a'** (600 MHz, $CDCl_3$, 25 ºC)

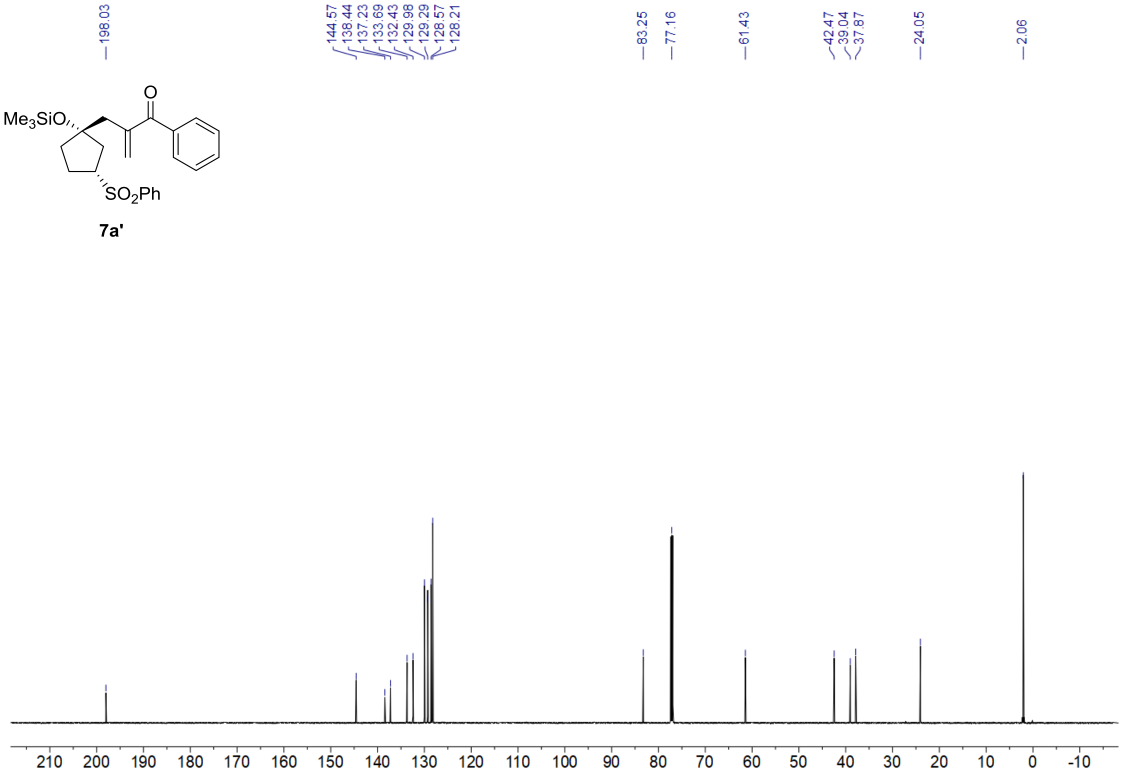

Supplementary Figure 126: ^{13}C NMR of **7a'** (151 MHz, $CDCl_3$, 25 ºC)

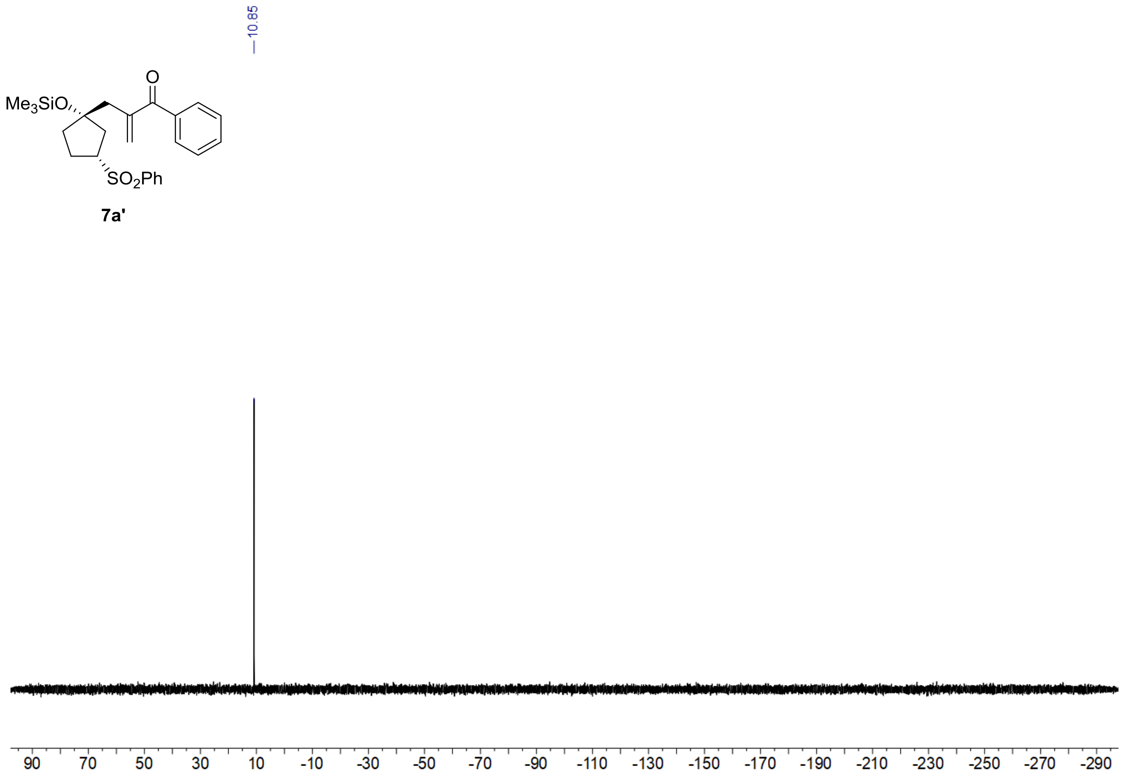

Supplementary Figure 127: ^{29}Si NMR of **7a’** (119 MHz, $CDCl_3$, 25 ºC)

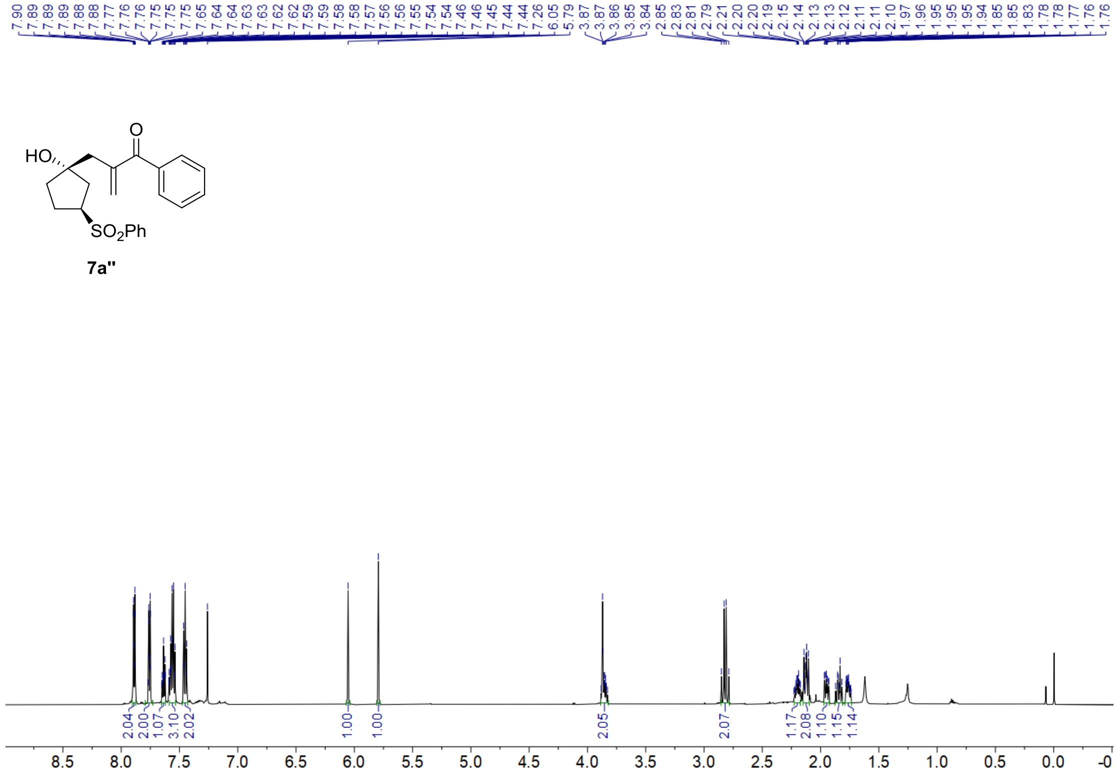

Supplementary Figure 128: ^{1}H NMR of **7a”** (600 MHz, $CDCl_3$, 25 ºC)

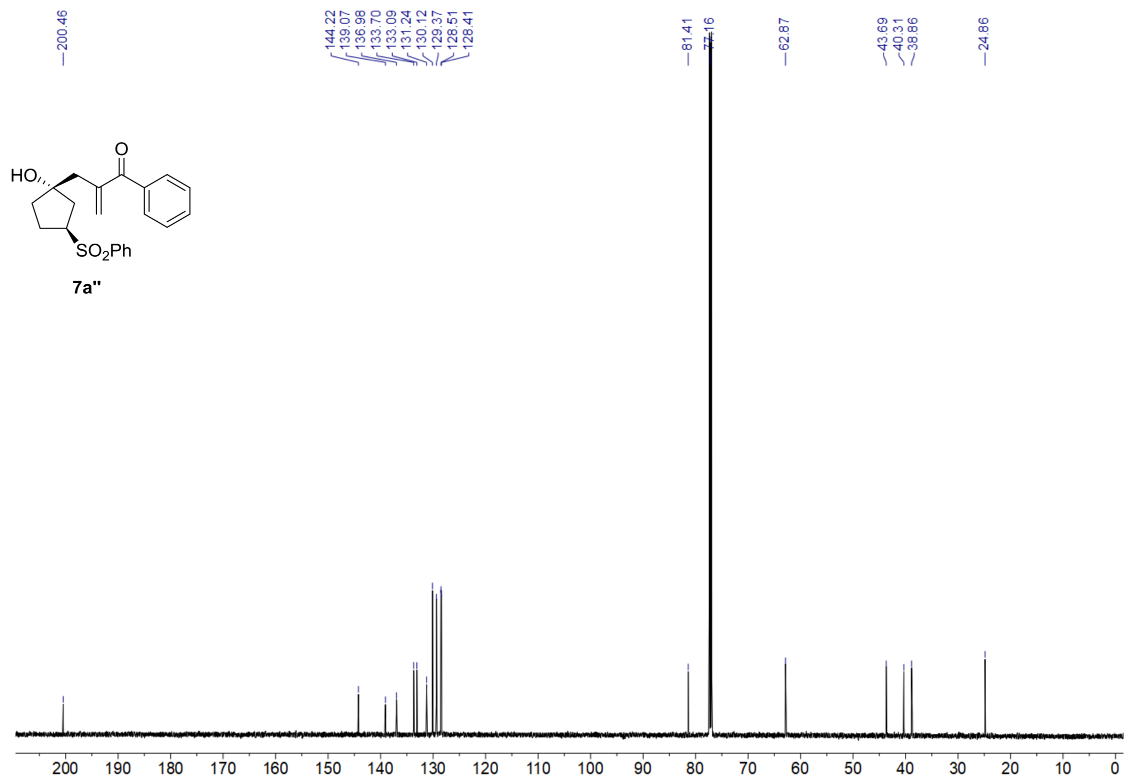

Supplementary Figure 129: ^{13}C NMR of **7a"** (151 MHz, $CDCl_3$, 25 ºC)

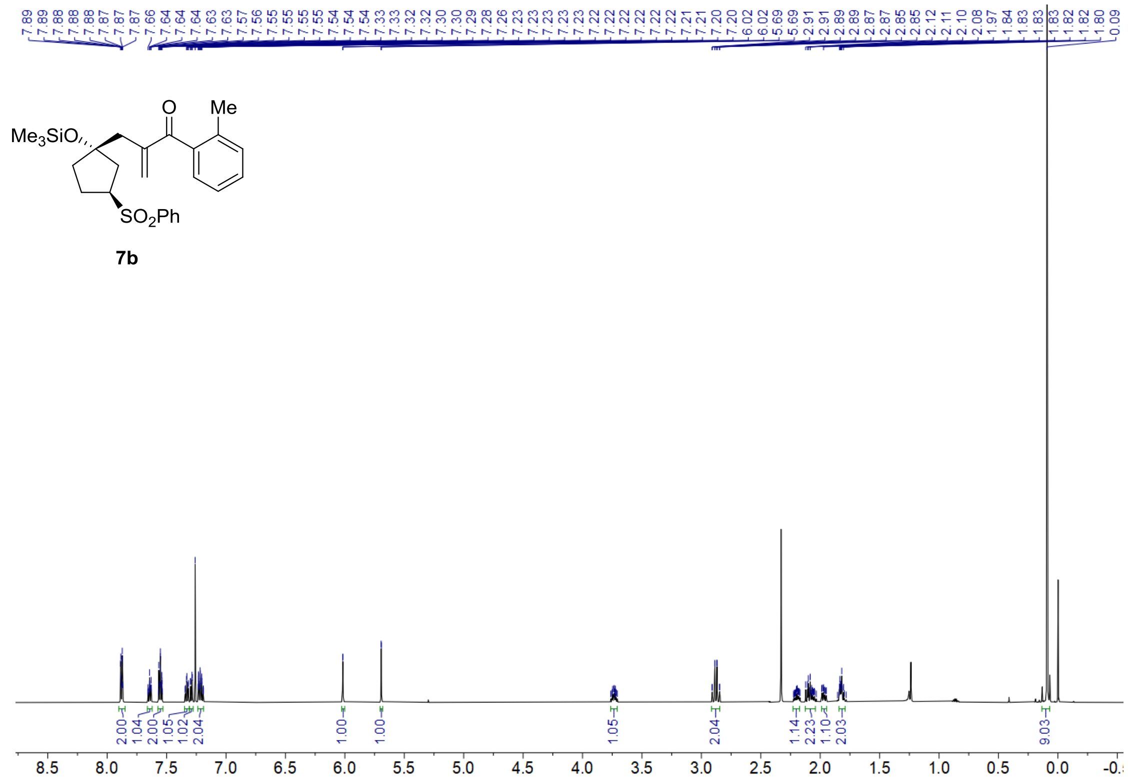

Supplementary Figure 130: ^{1}H NMR of **7b** (600 MHz, $CDCl_3$, 25 ºC)

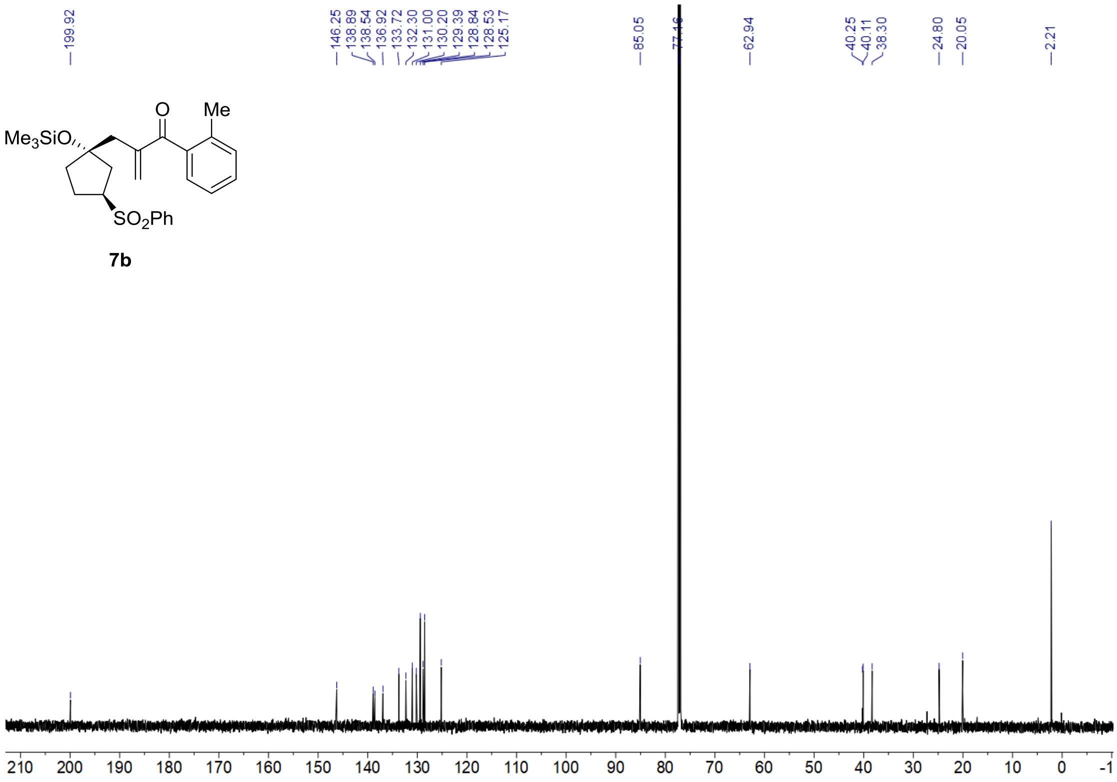

Supplementary Figure 131: ^{13}C NMR of **7b** (151 MHz, $CDCl_3$, 25 ºC)

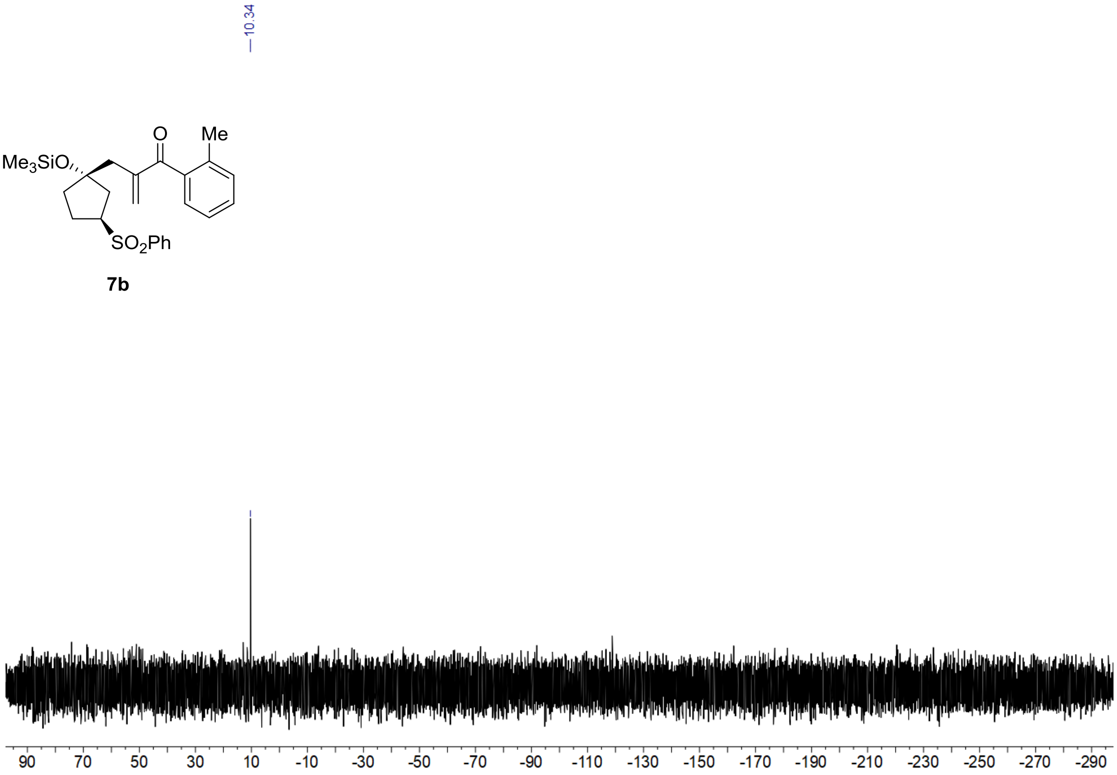

Supplementary Figure 132: ^{29}Si NMR of **7b** (119 MHz, $CDCl_3$, 25 ºC)

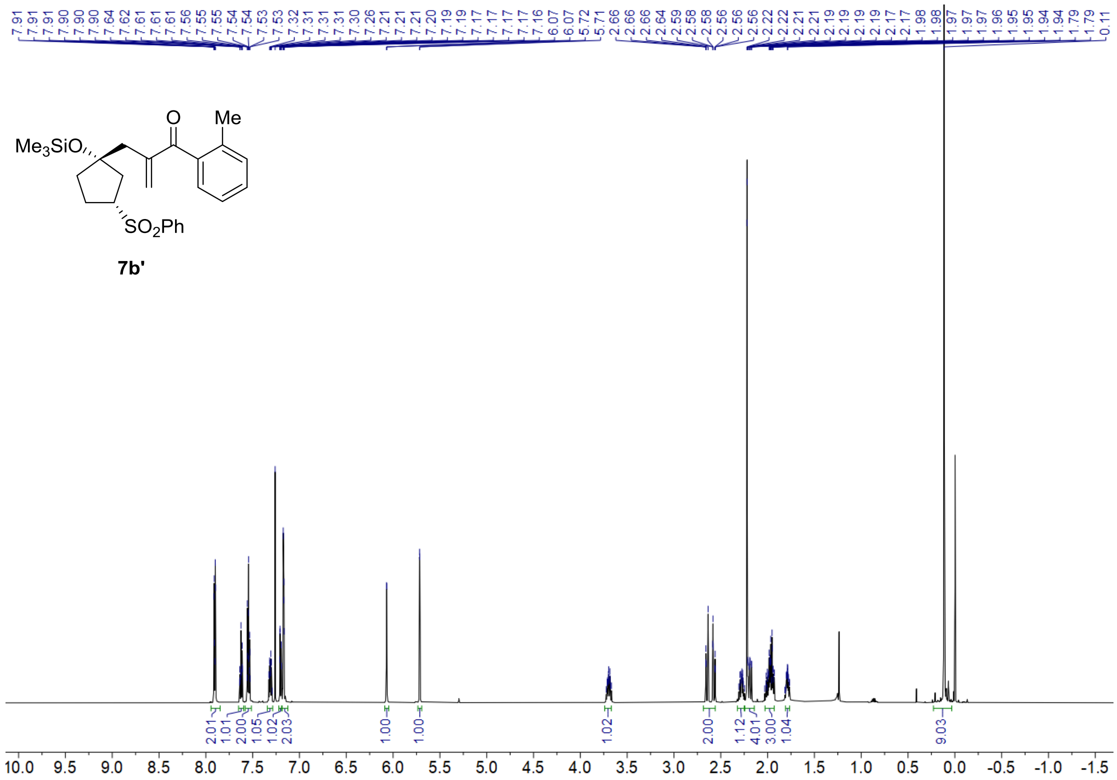

Supplementary Figure 133: ^{1}H NMR of 7b' (600 MHz, $CDCl_3$, 25 ºC)

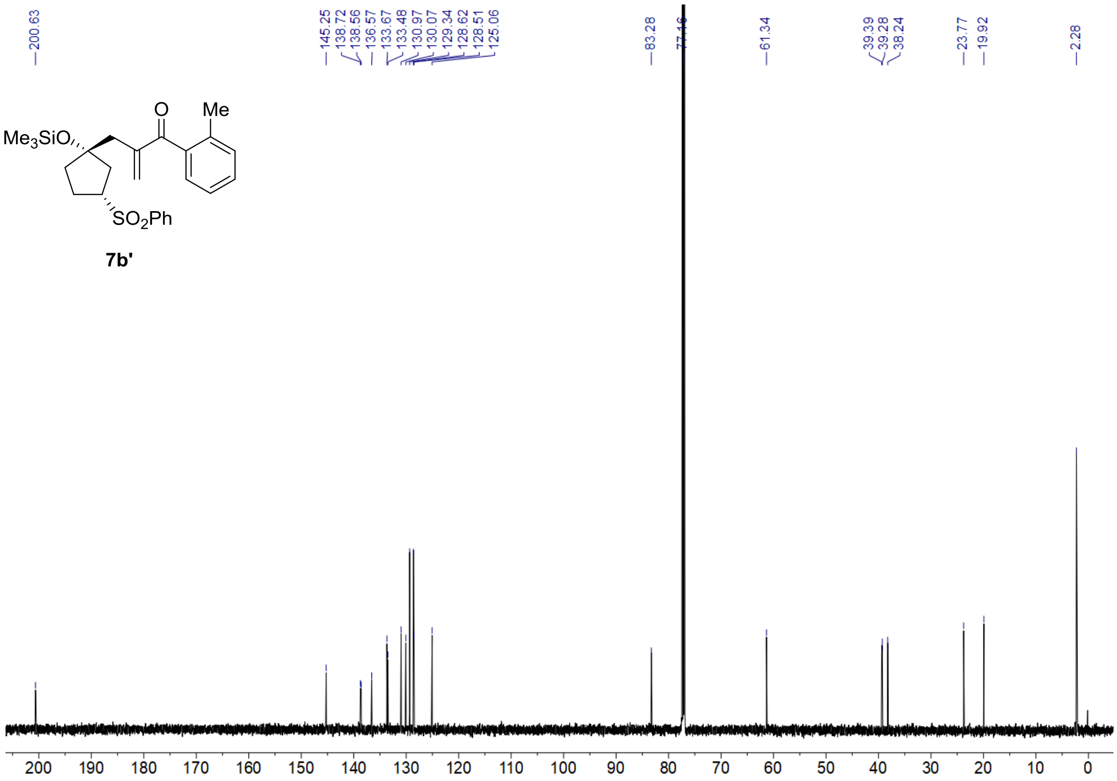

Supplementary Figure 134: ^{13}C NMR of 7b' (151 MHz, $CDCl_3$, 25 ºC)

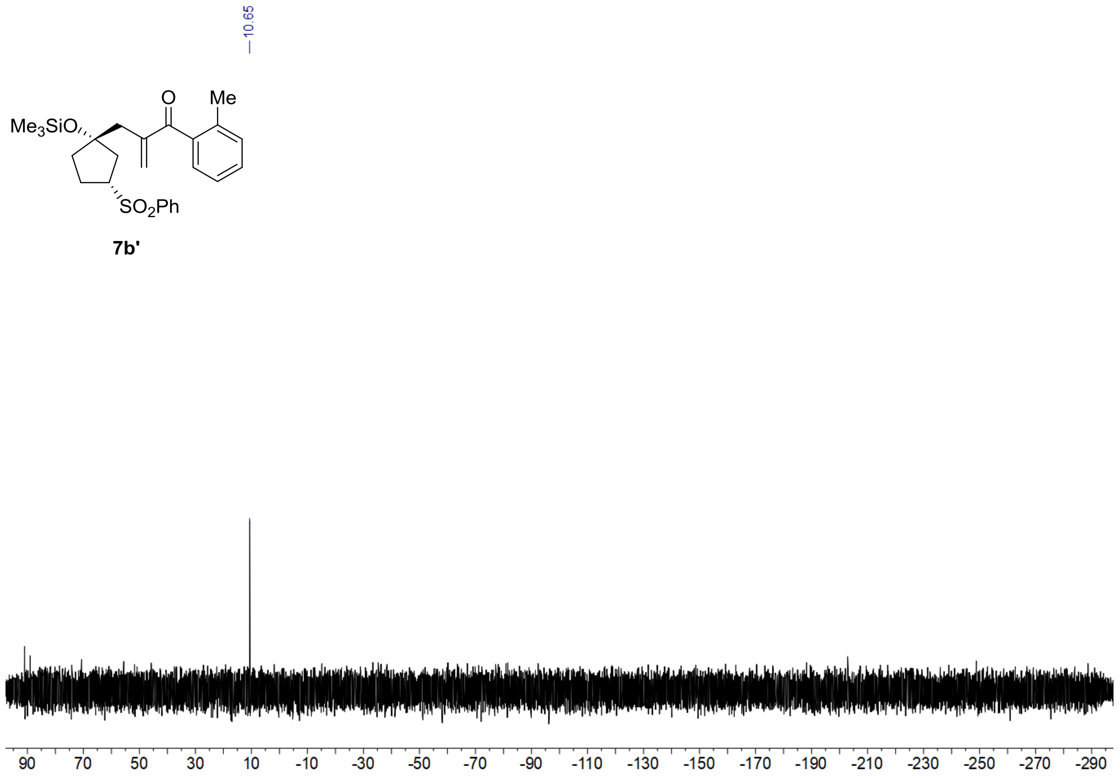

Supplementary Figure 135: ^{29}Si NMR of **7b'** (119 MHz, $CDCl_3$, 25 ºC)

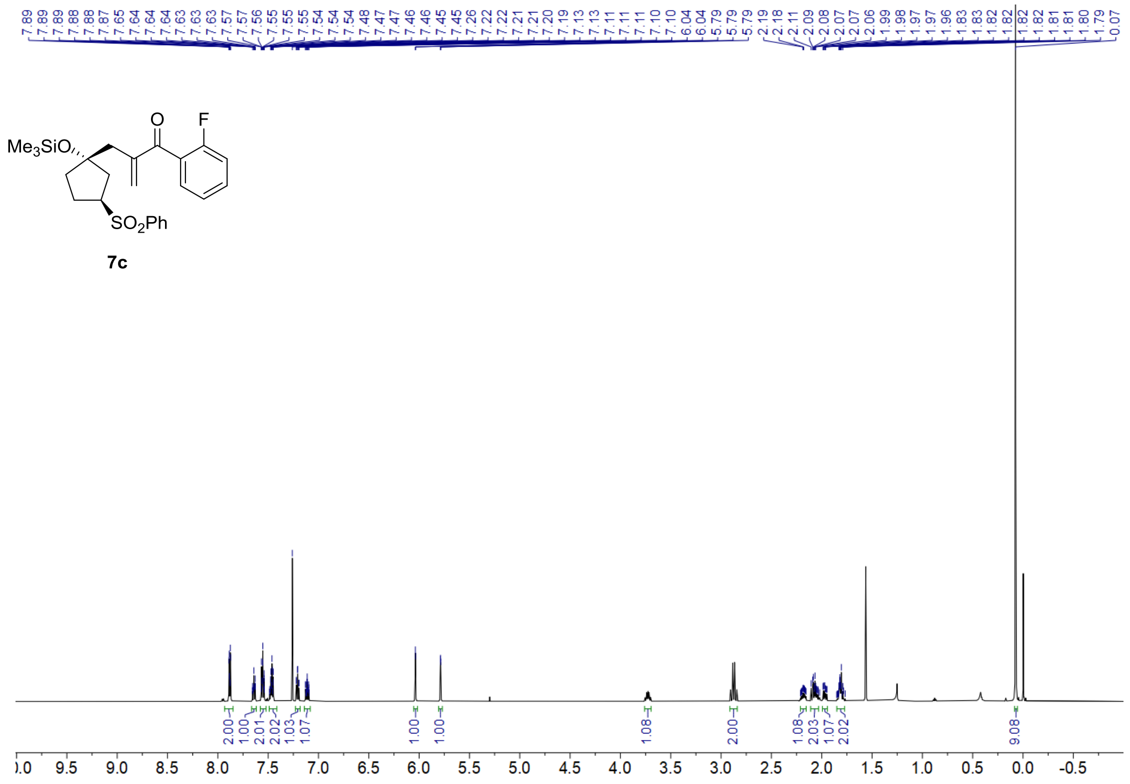

Supplementary Figure 136: ^{1}H NMR of **7c** (600 MHz, $CDCl_3$, 25 ºC)

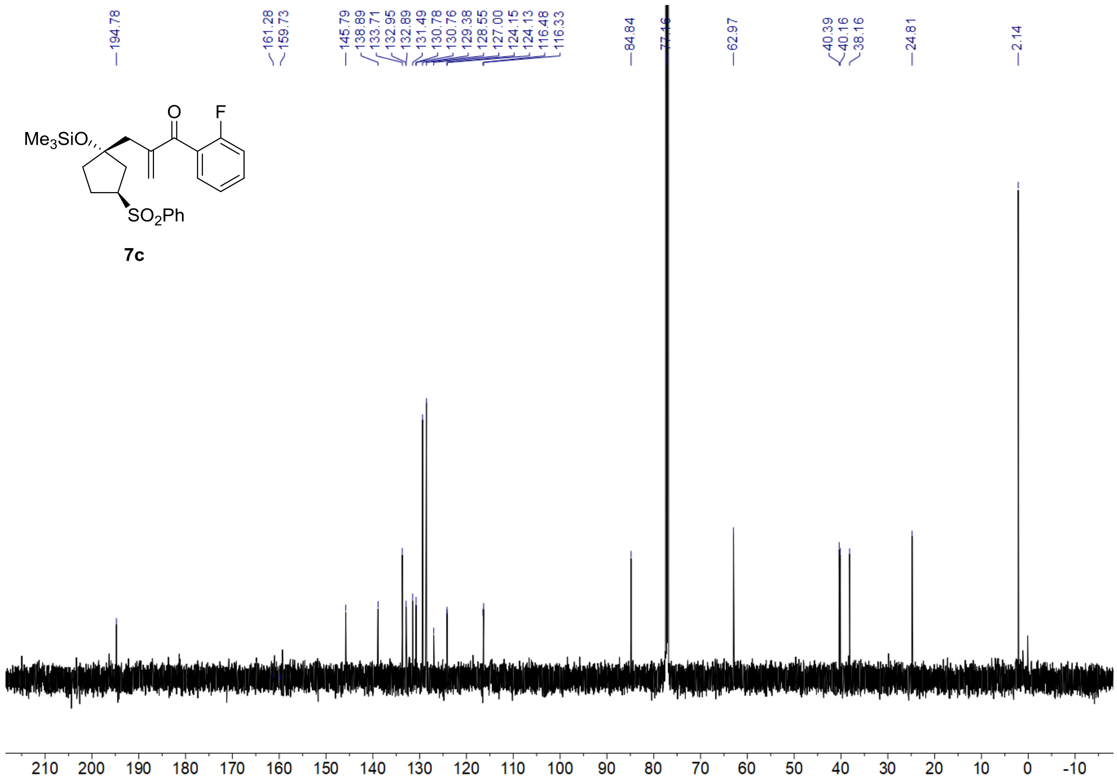

Supplementary Figure 137: ^{13}C NMR of **7c** (151 MHz, $CDCl_3$, 25 ºC)

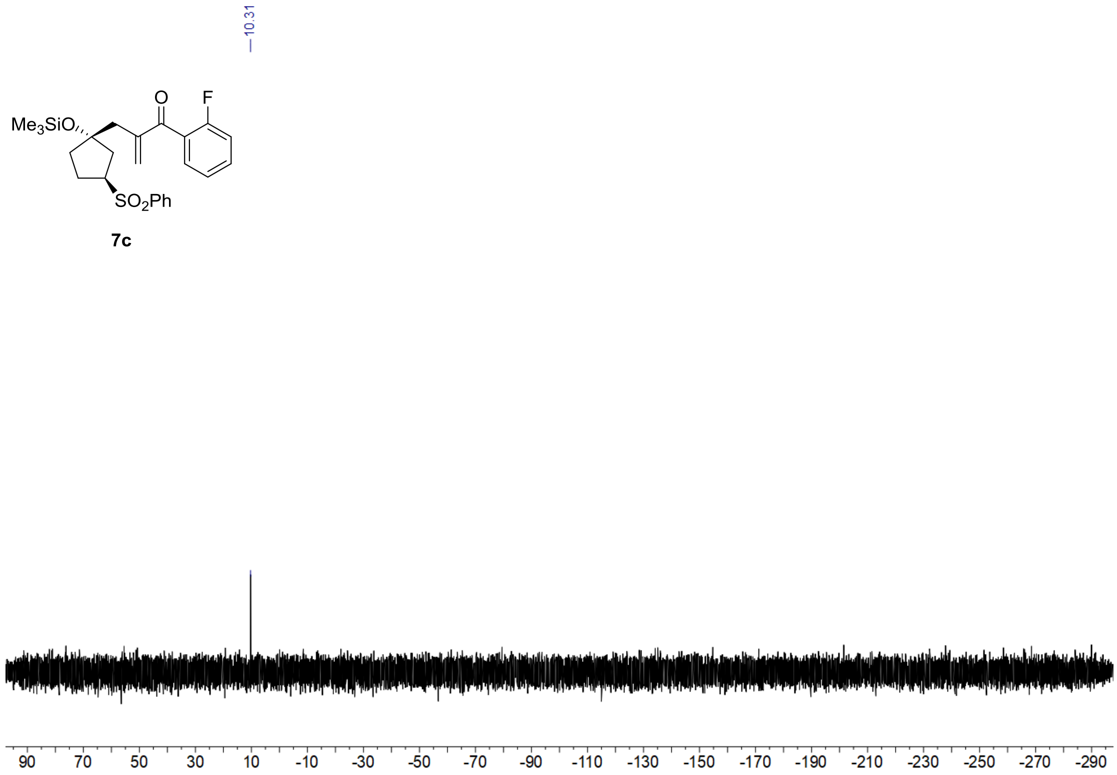

Supplementary Figure 138: ^{29}Si NMR of **7c** (119 MHz, $CDCl_3$, 25 ºC)

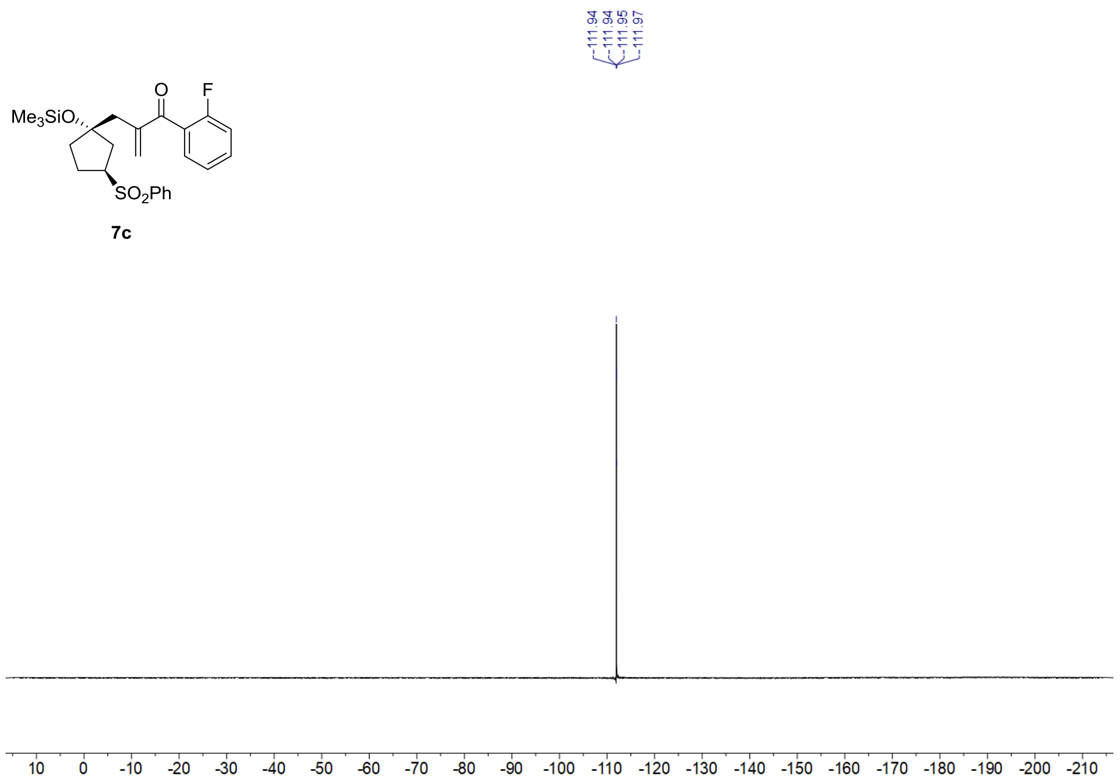

Supplementary Figure 139: ^{19}F NMR of **7c** (565 MHz, $CDCl_3$, 25 ºC)

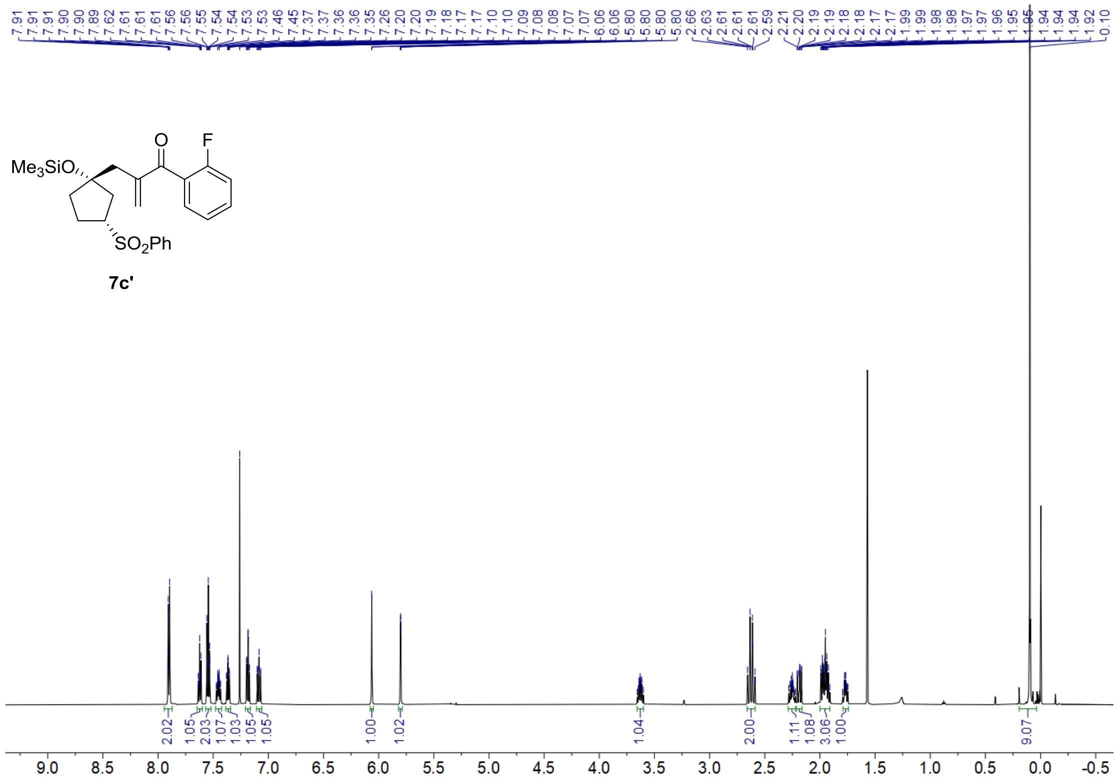

Supplementary Figure 140: ^{1}H NMR of **7c'** (600 MHz, $CDCl_3$, 25 ºC)

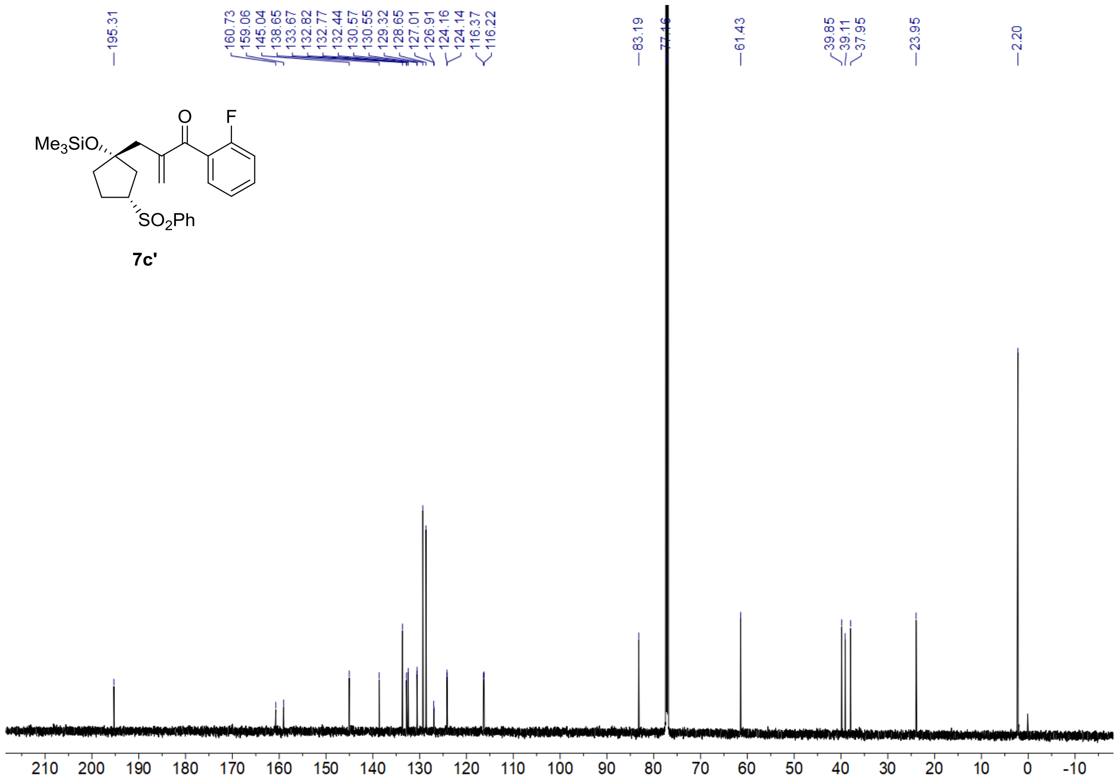

Supplementary Figure 141: ^{13}C NMR of **7c'** (151 MHz, $CDCl_3$, 25 ºC)

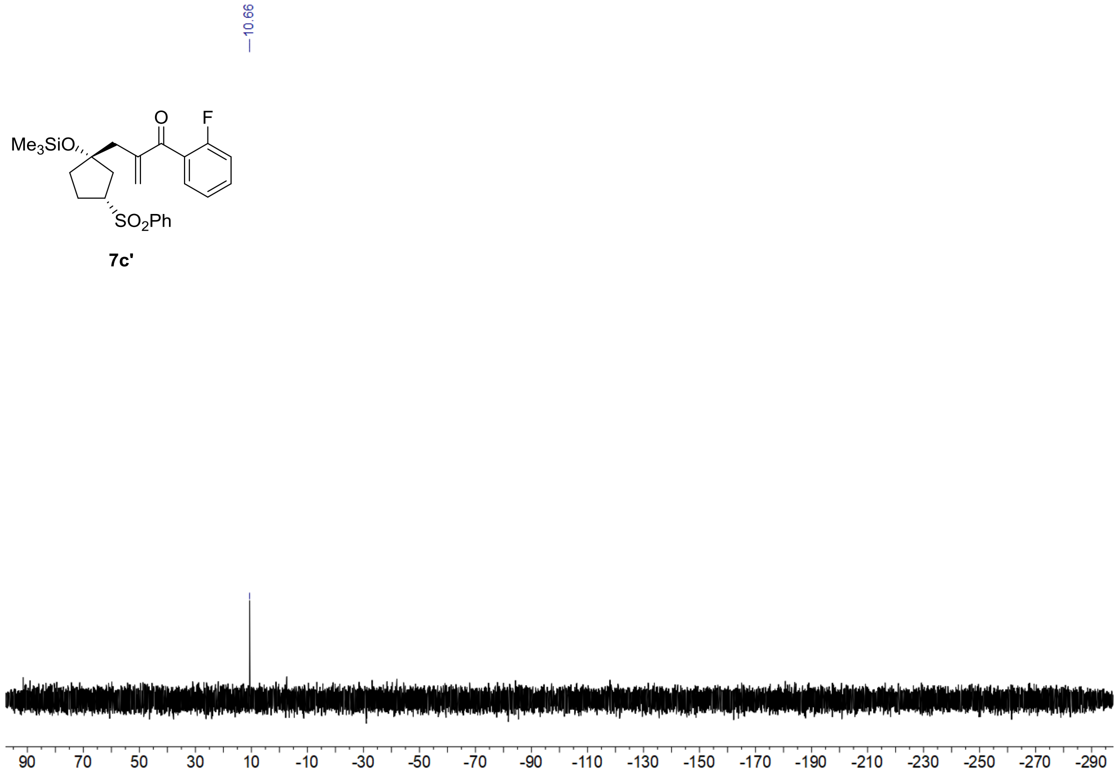

Supplementary Figure 142: ^{29}Si NMR of **7c'** (119 MHz, $CDCl_3$, 25 ºC)

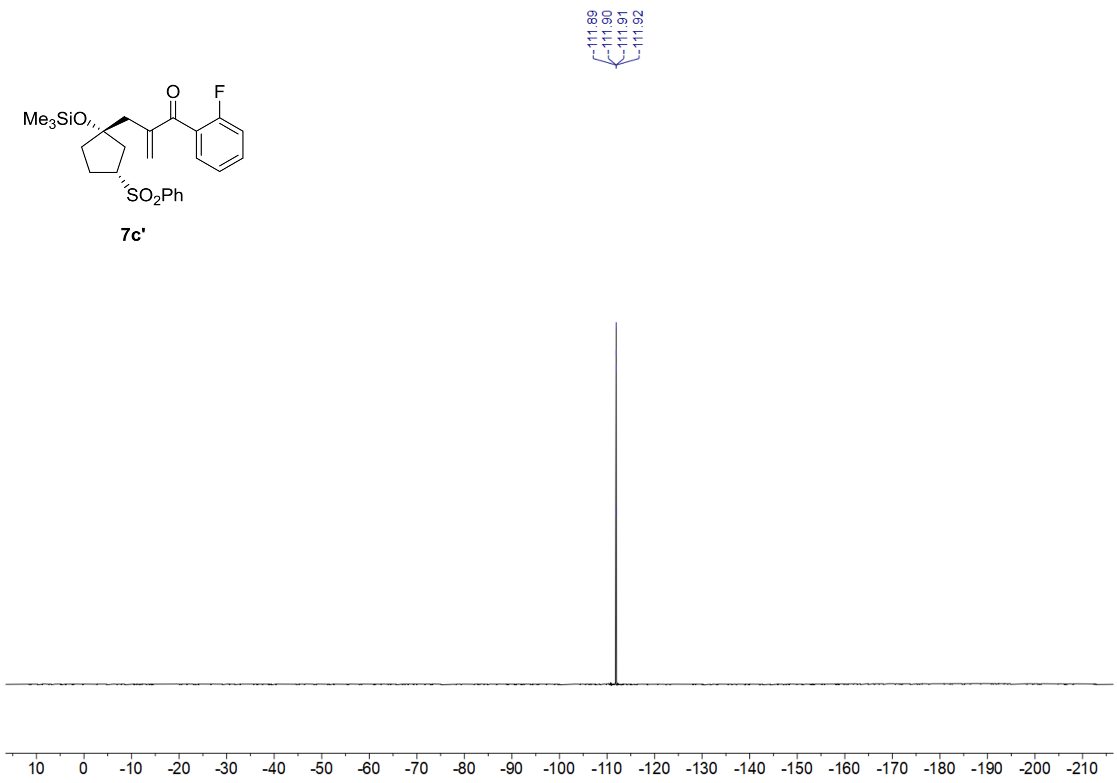

Supplementary Figure 143: ^{19}F NMR of **7c’** (565 MHz, $CDCl_3$, 25 ºC)

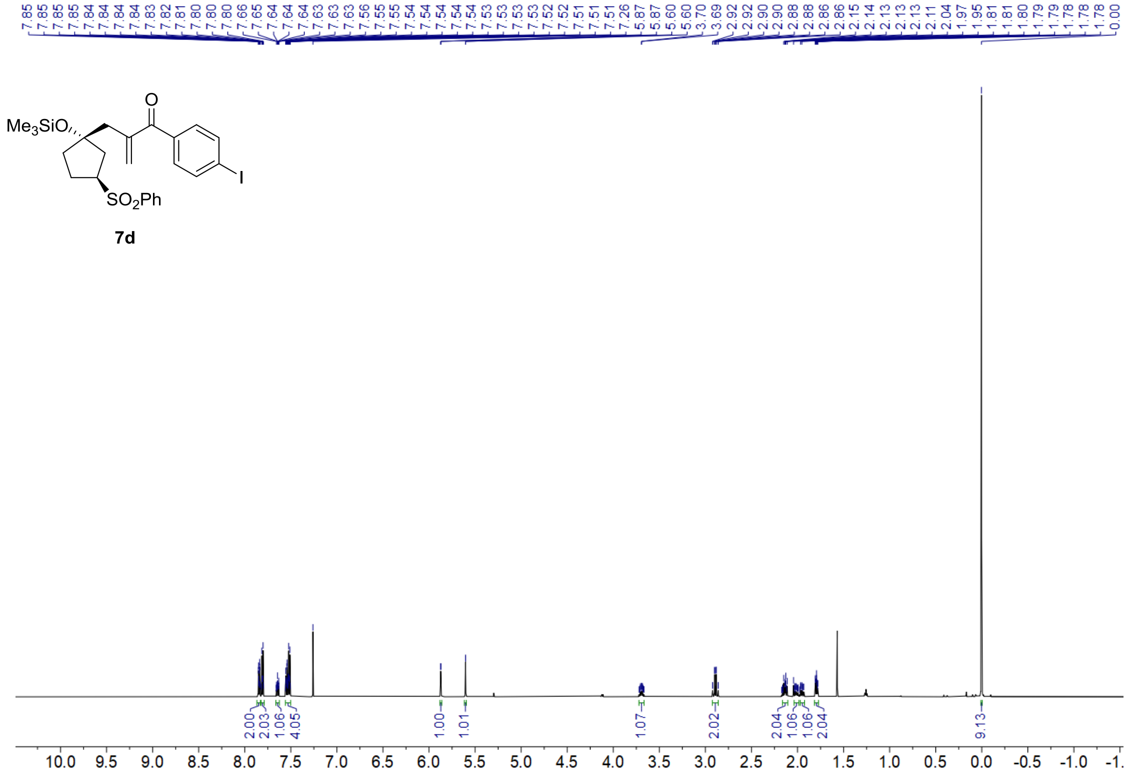

Supplementary Figure 144: ^{1}H NMR of **7d** (600 MHz, $CDCl_3$, 25 ºC)

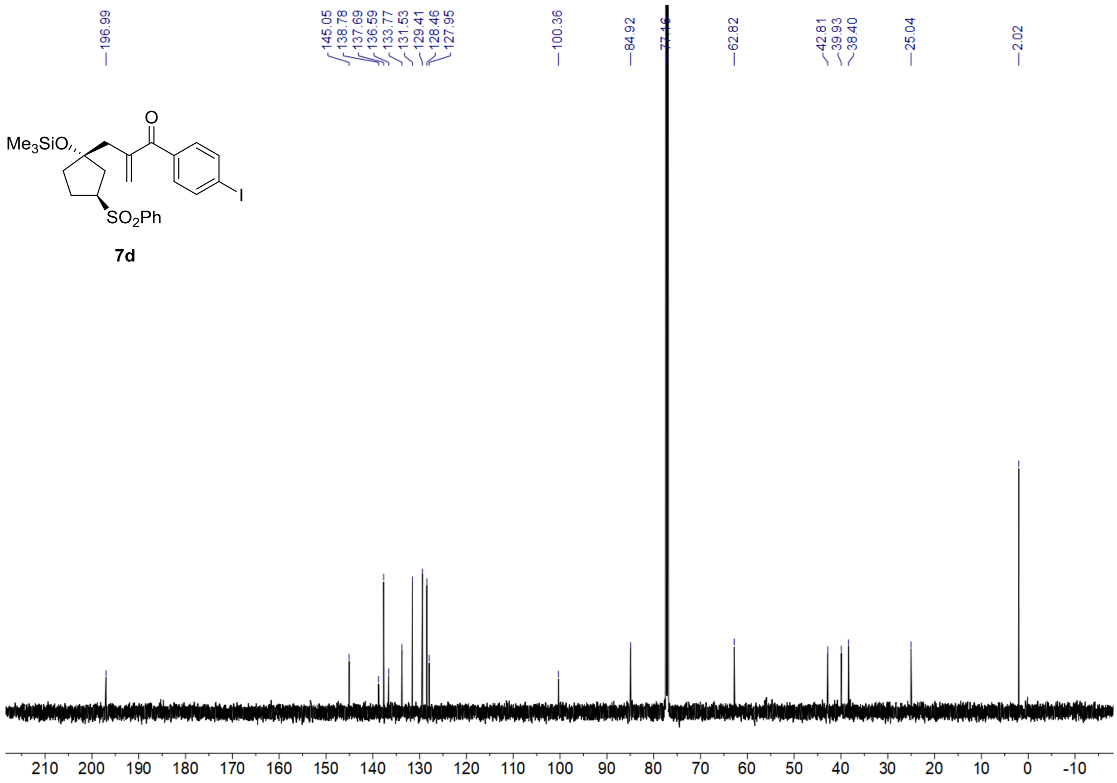

Supplementary Figure 145: ^{13}C NMR of **7d** (151 MHz, $CDCl_3$, 25 ºC)

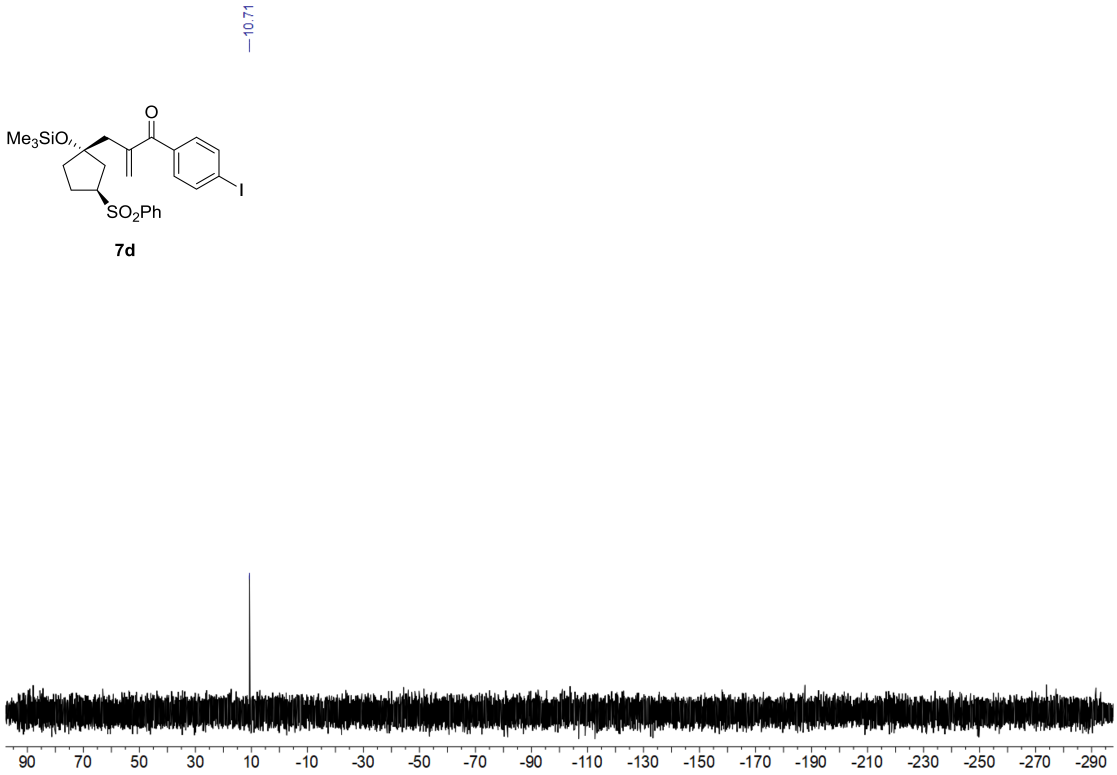

Supplementary Figure 146: ^{29}Si NMR of **7d** (119 MHz, $CDCl_3$, 25 ºC)

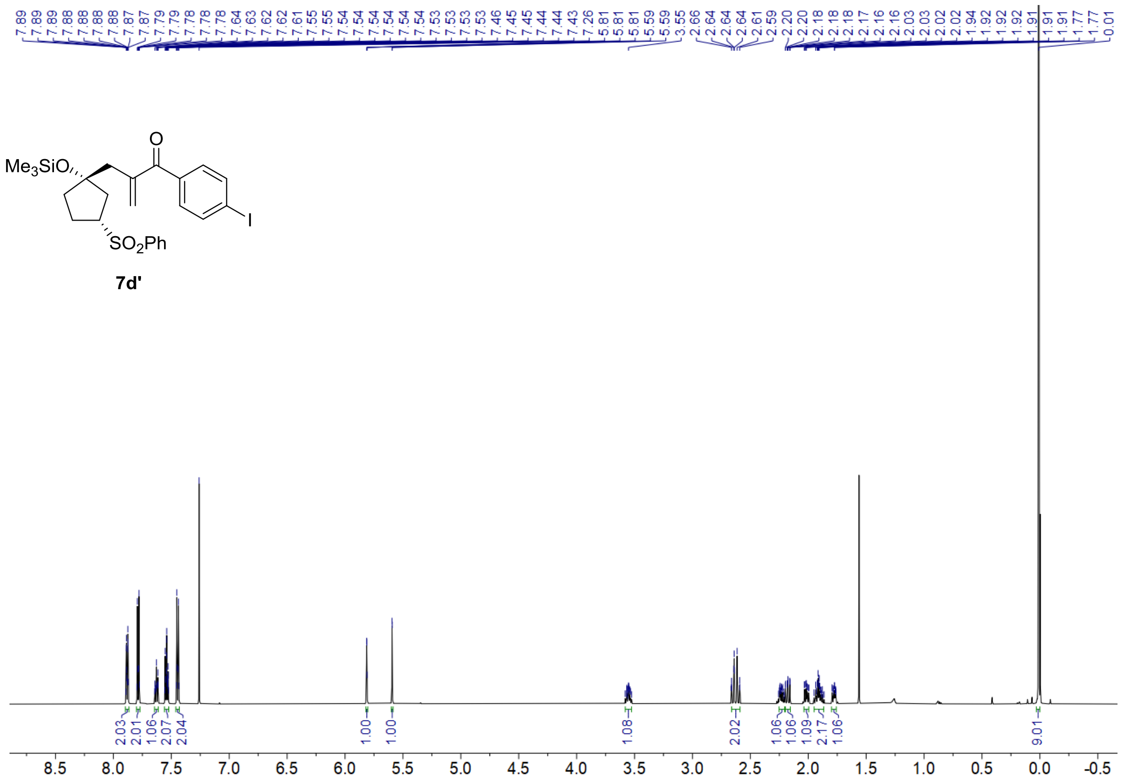

Supplementary Figure 147: ^{1}H NMR of **7d'** (600 MHz, $CDCl_3$, 25 ºC)

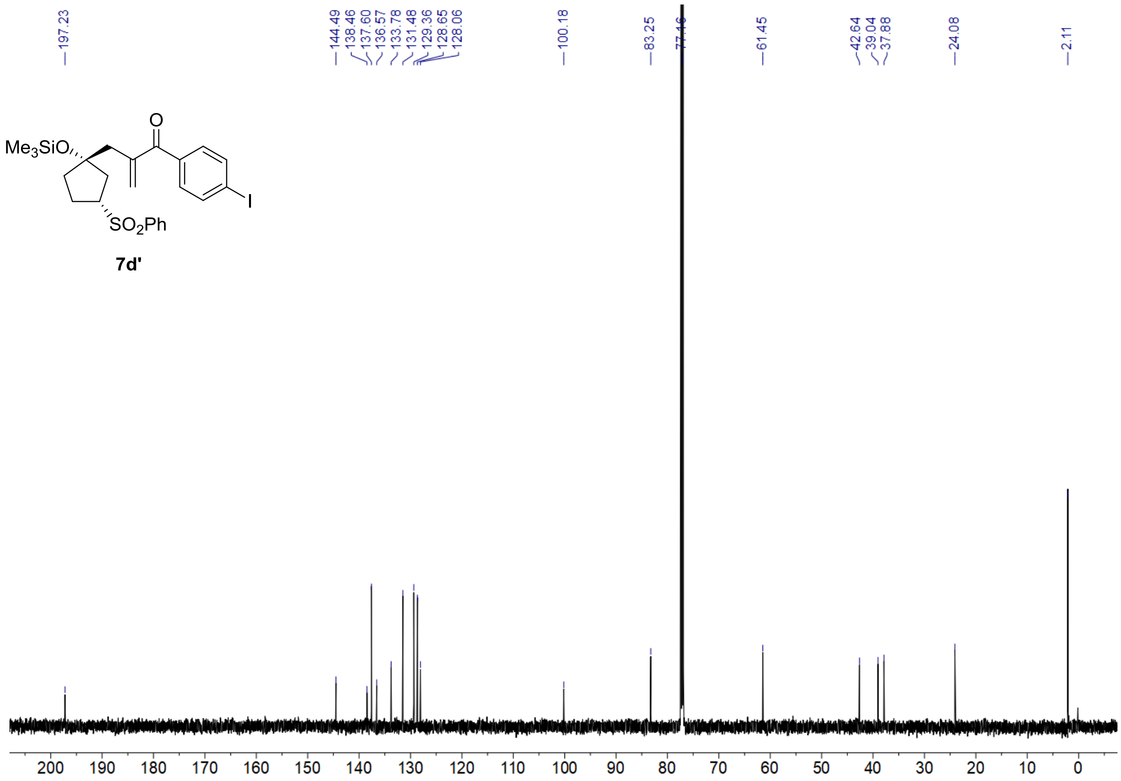

Supplementary Figure 148: ^{13}C NMR of **7d'** (151 MHz, $CDCl_3$, 25 ºC)

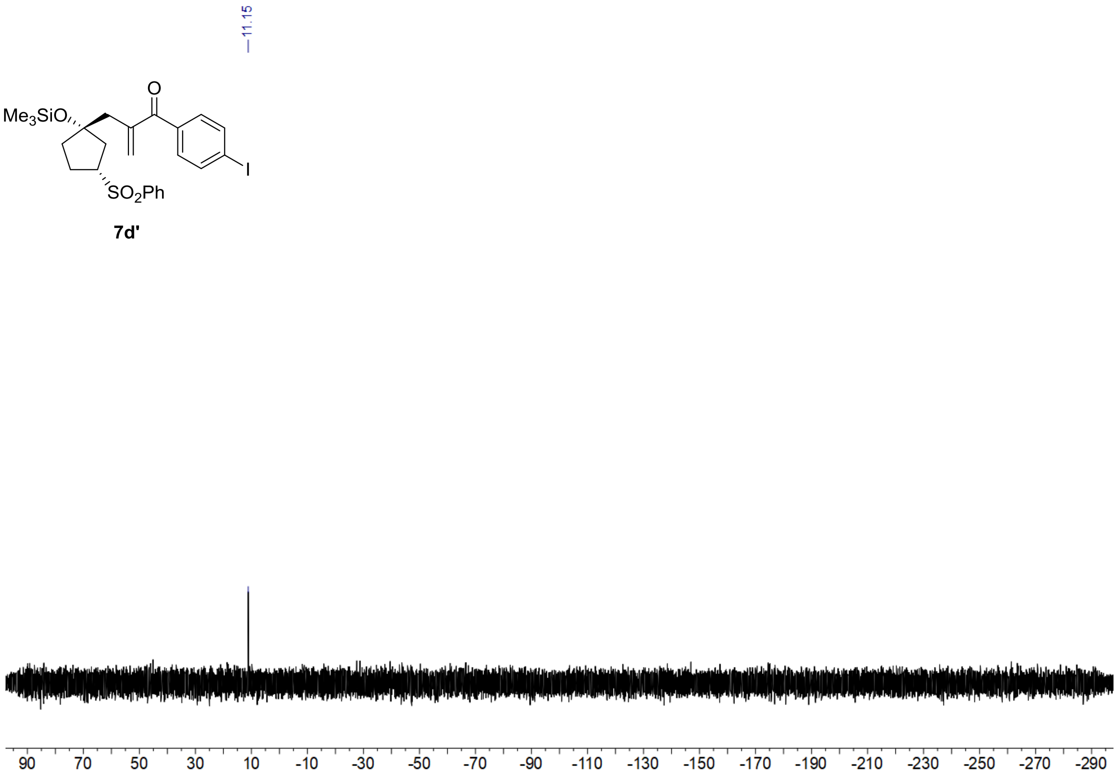

Supplementary Figure 149: ^{29}Si NMR of **7d’** (119 MHz, $CDCl_3$, 25 ºC)

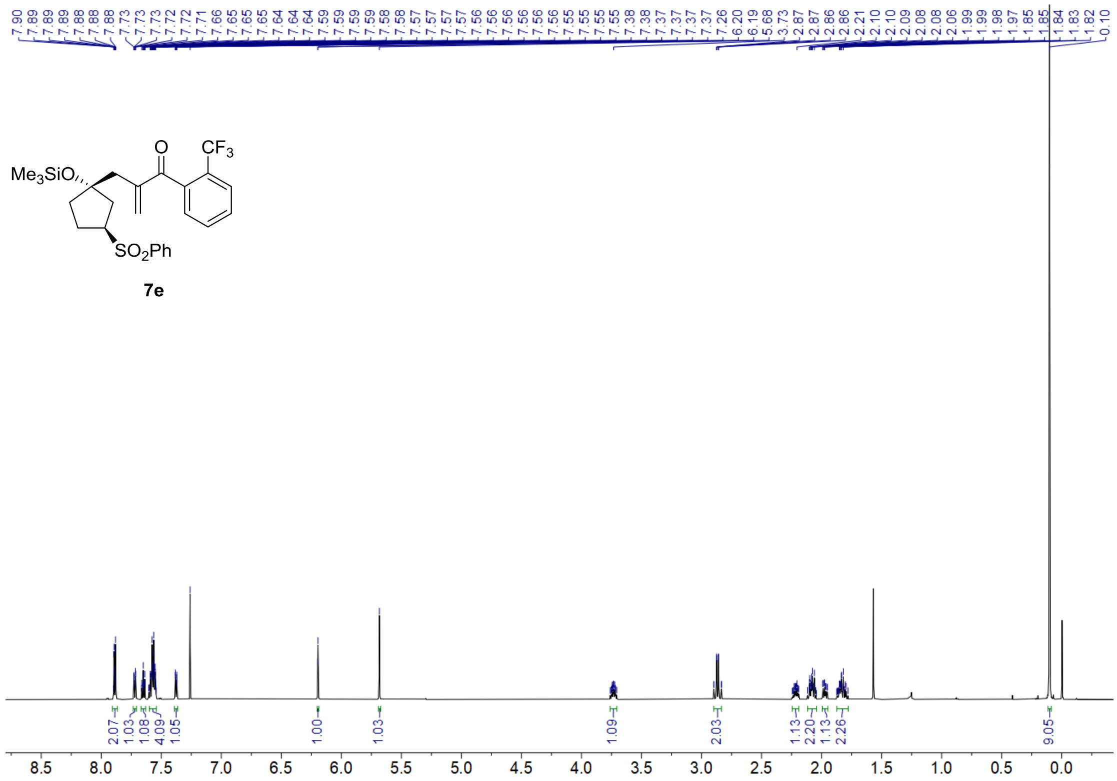

Supplementary Figure 150: ^{1}H NMR of **7e** (600 MHz, $CDCl_3$, 25 ºC)

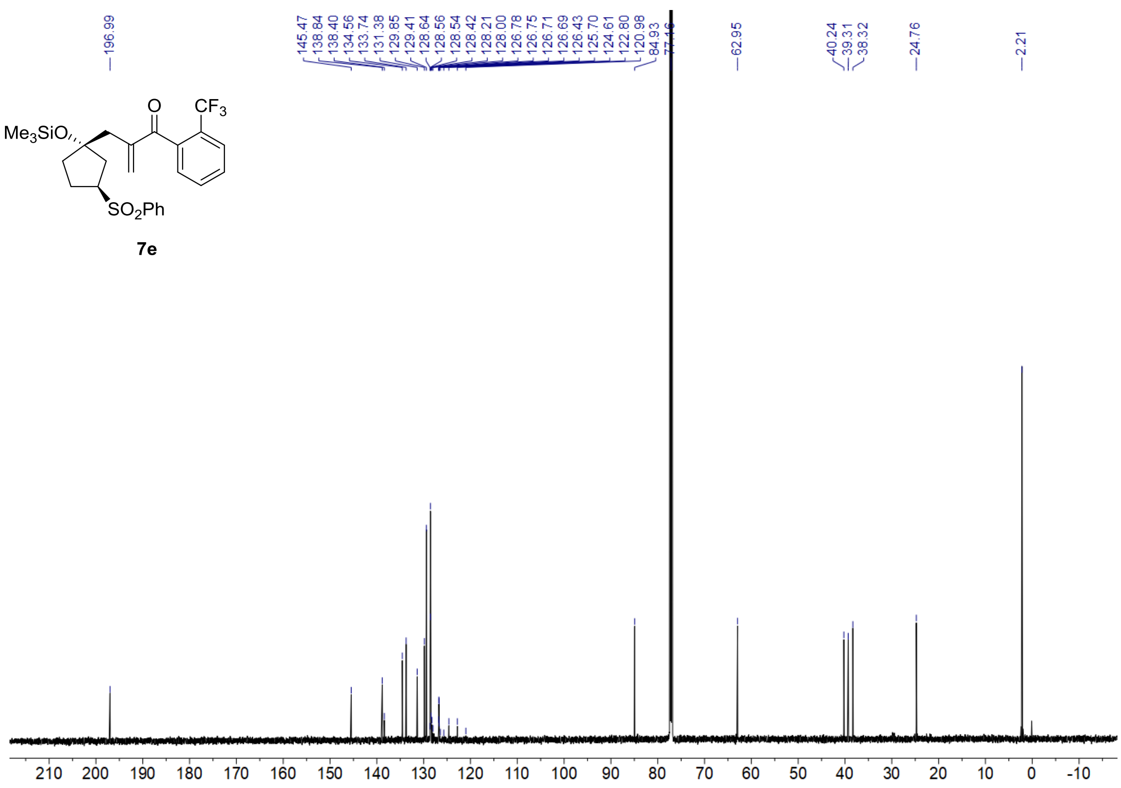

Supplementary Figure 151: ^{13}C NMR of **7e** (151 MHz, $CDCl_3$, 25 ºC)

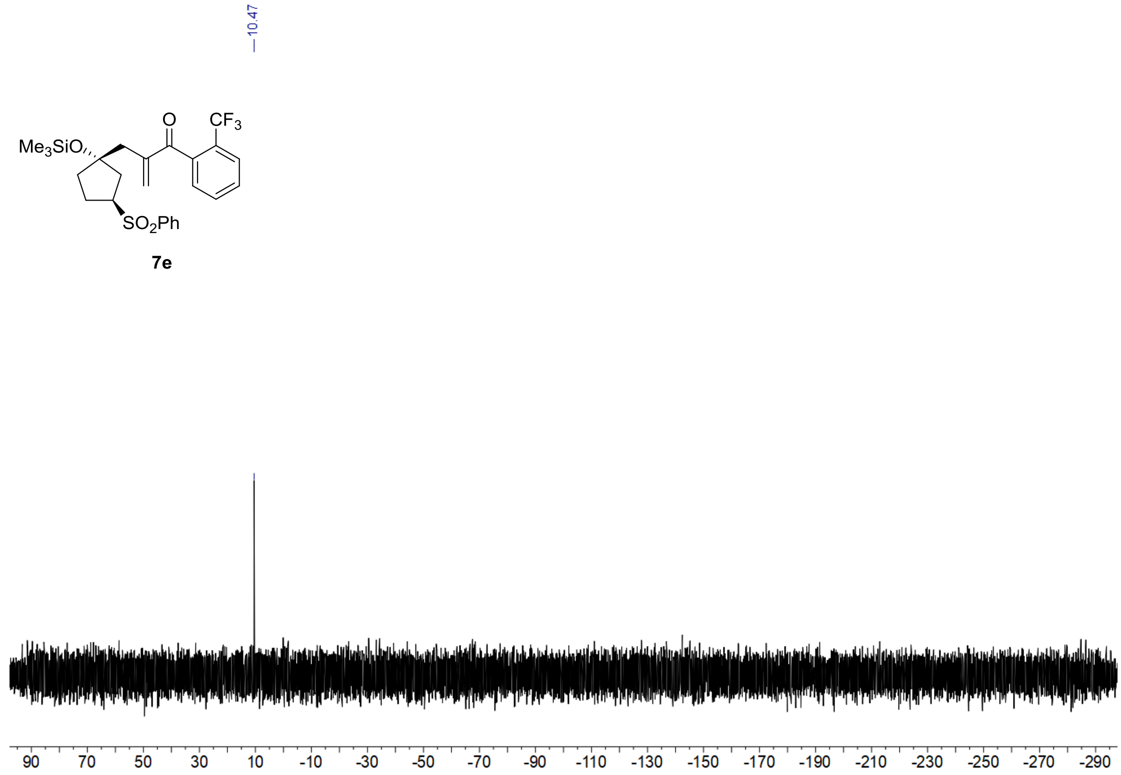

Supplementary Figure 152: ^{29}Si NMR of **7e** (119 MHz, $CDCl_3$, 25 ºC)

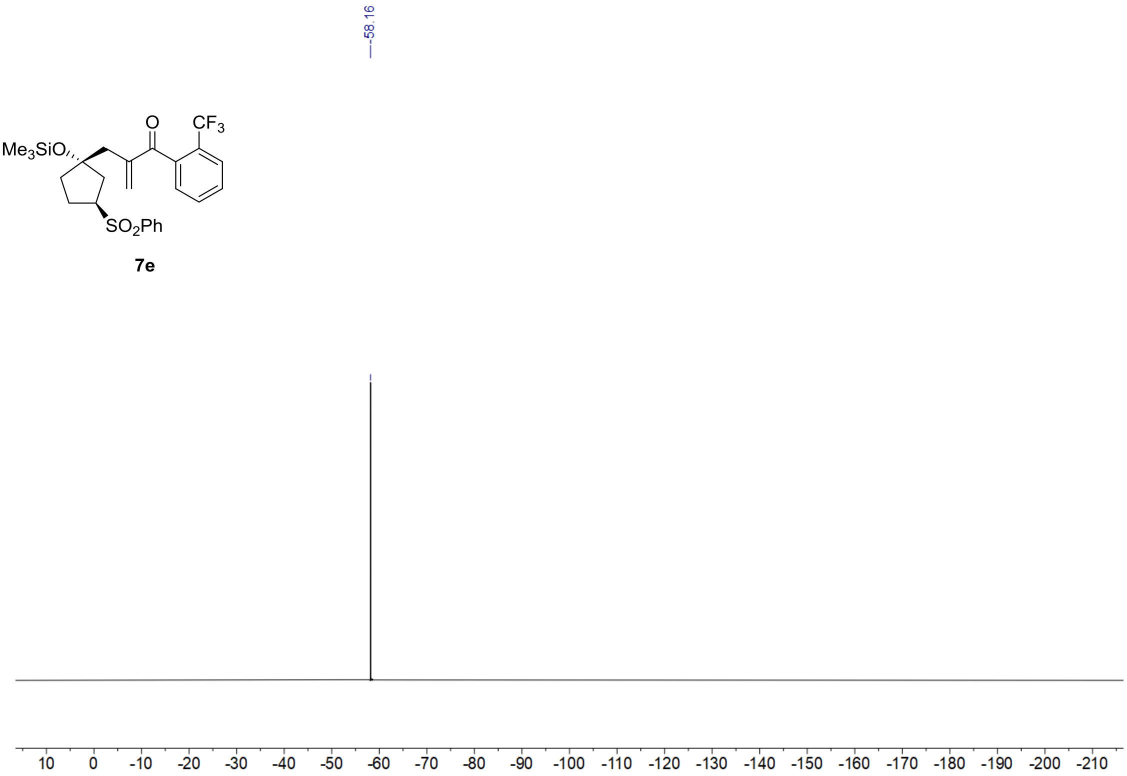

Supplementary Figure 153: ^{19}F NMR of **7e** (565 MHz, $CDCl_3$, 25 ºC)

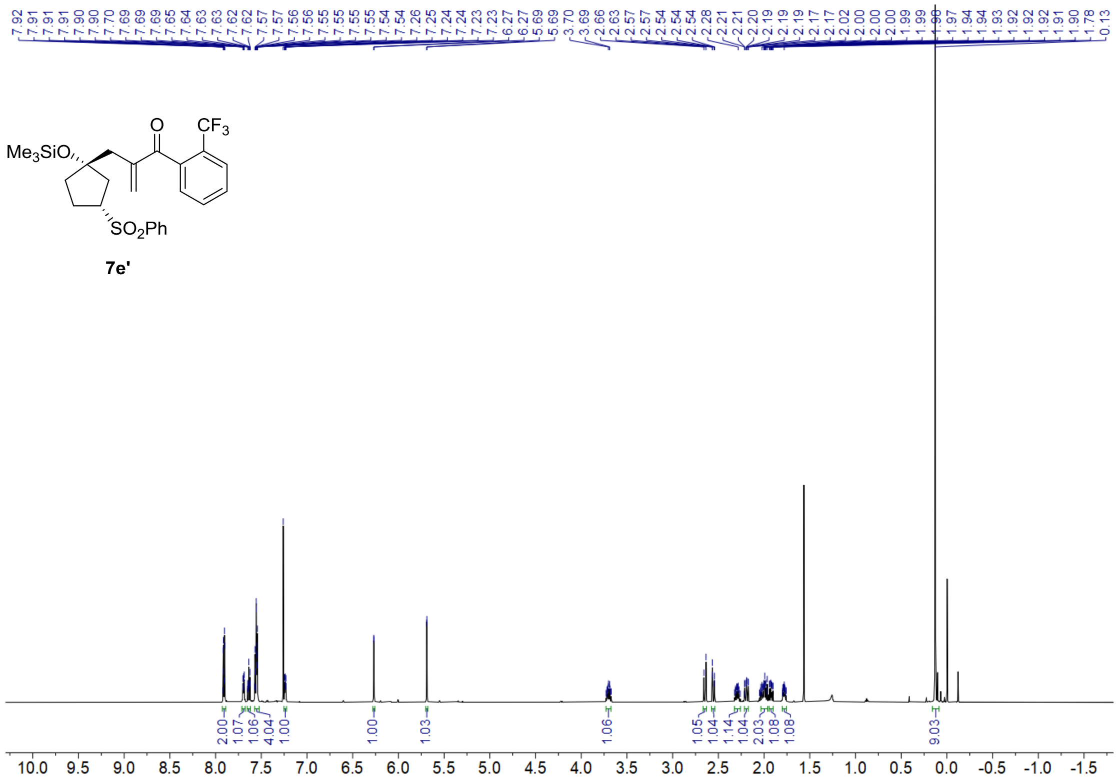

Supplementary Figure 154: ^{1}H NMR of **7e’** (600 MHz, $CDCl_3$, 25 ºC)

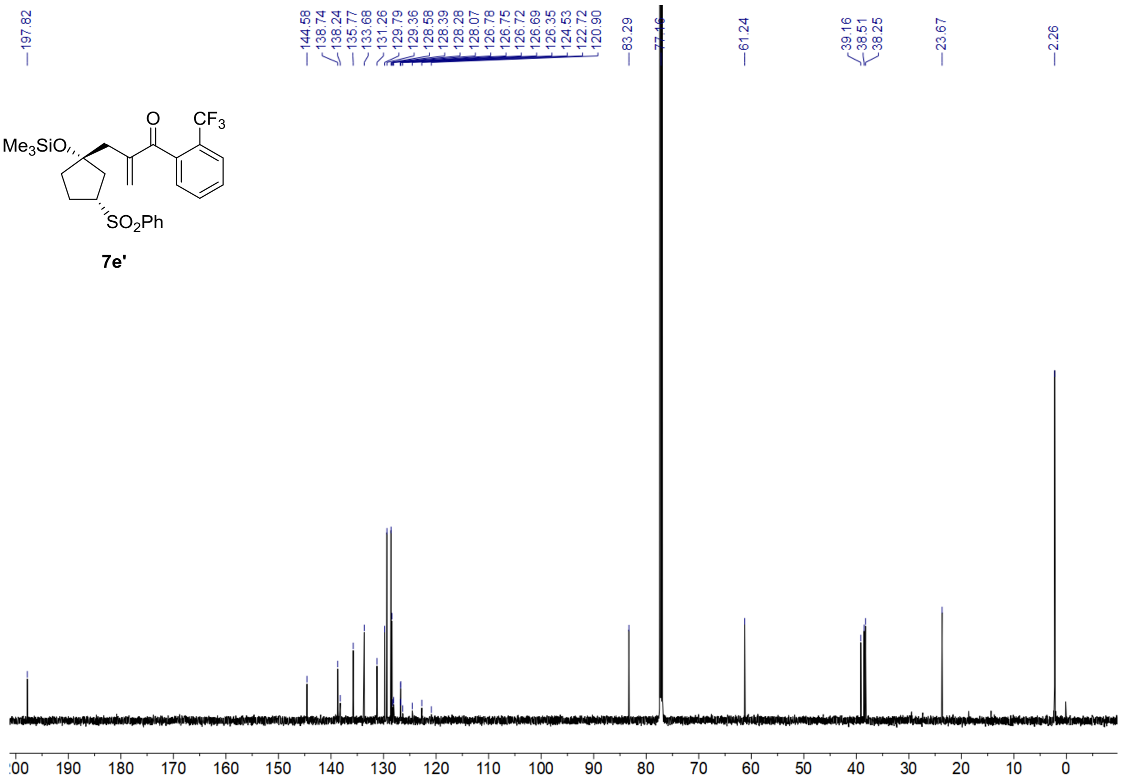

Supplementary Figure 155: ^{13}C NMR of **7e’** (151 MHz, $CDCl_3$, 25 ºC)

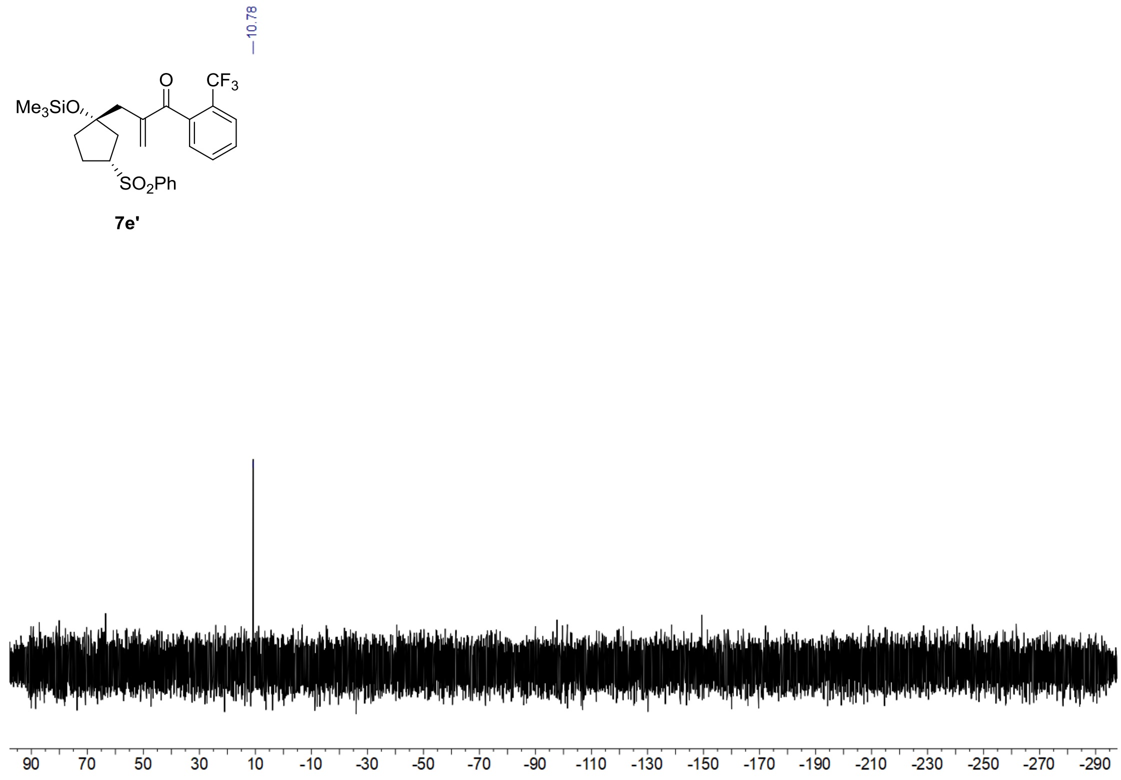

Supplementary Figure 156: ^{29}Si NMR of **7e’** (119 MHz, $CDCl_3$, 25 ºC)

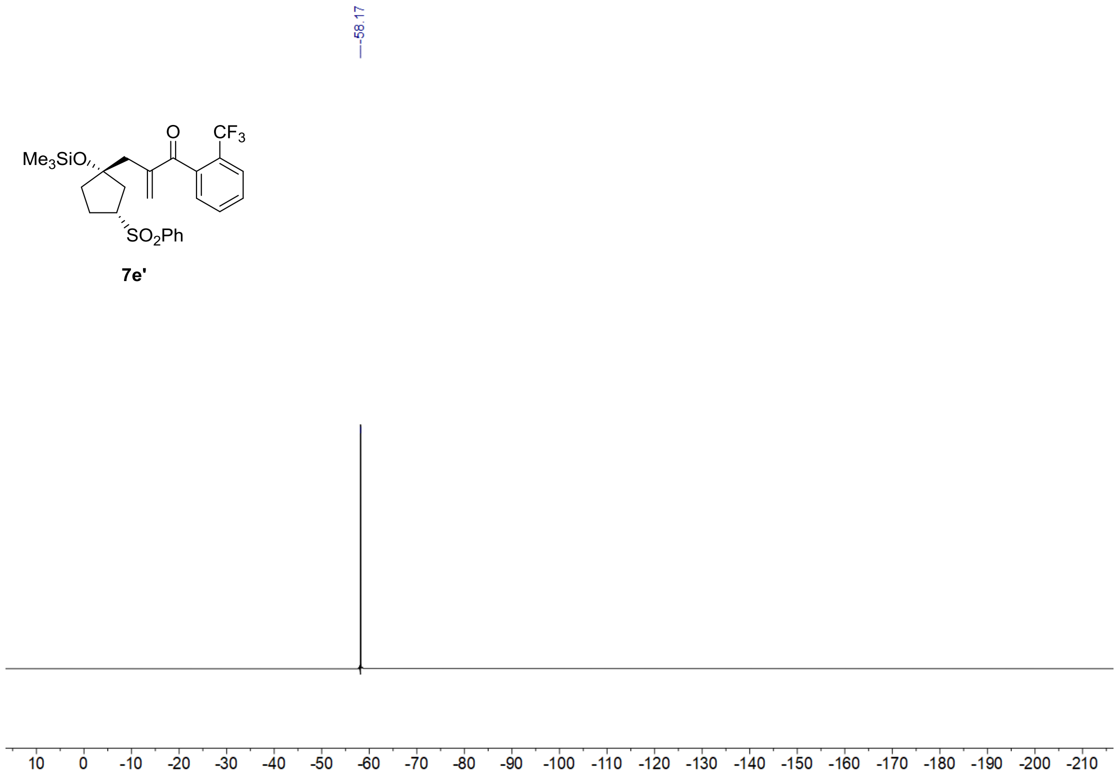

Supplementary Figure 157: ^{19}F NMR of **7e'** (565 MHz, $CDCl_3$, 25 ºC)

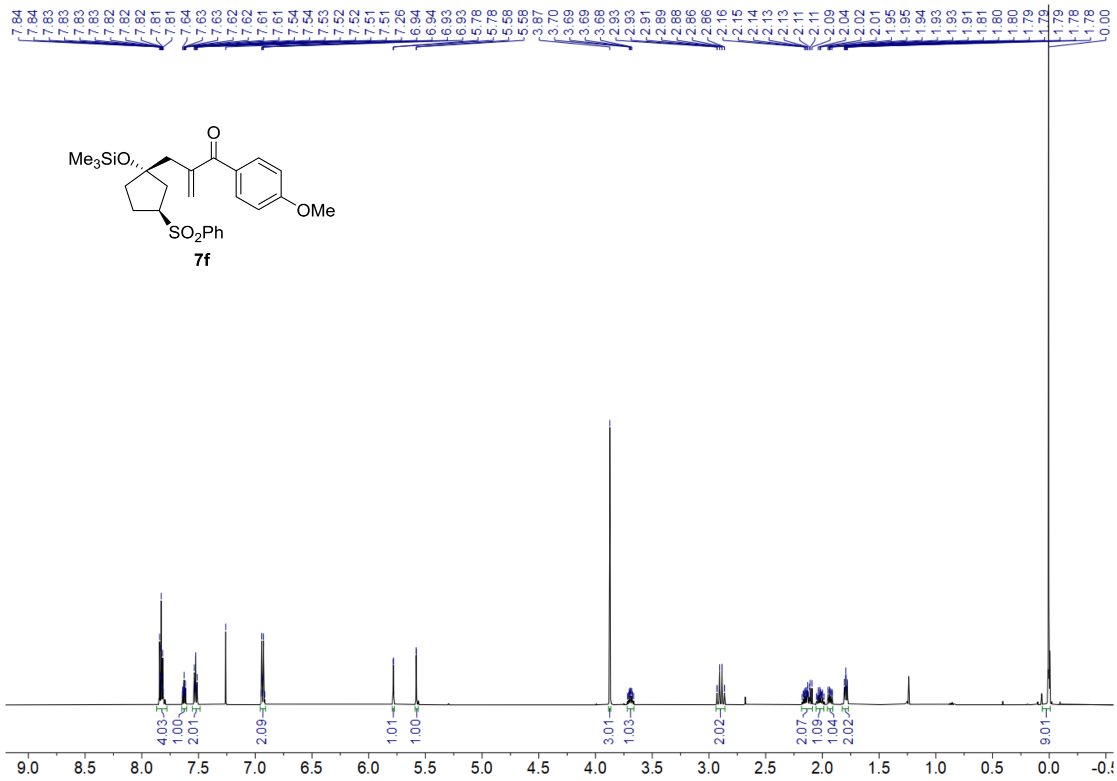

Supplementary Figure 158: ^{1}H NMR of **7f** (600 MHz, $CDCl_3$, 25 ºC)

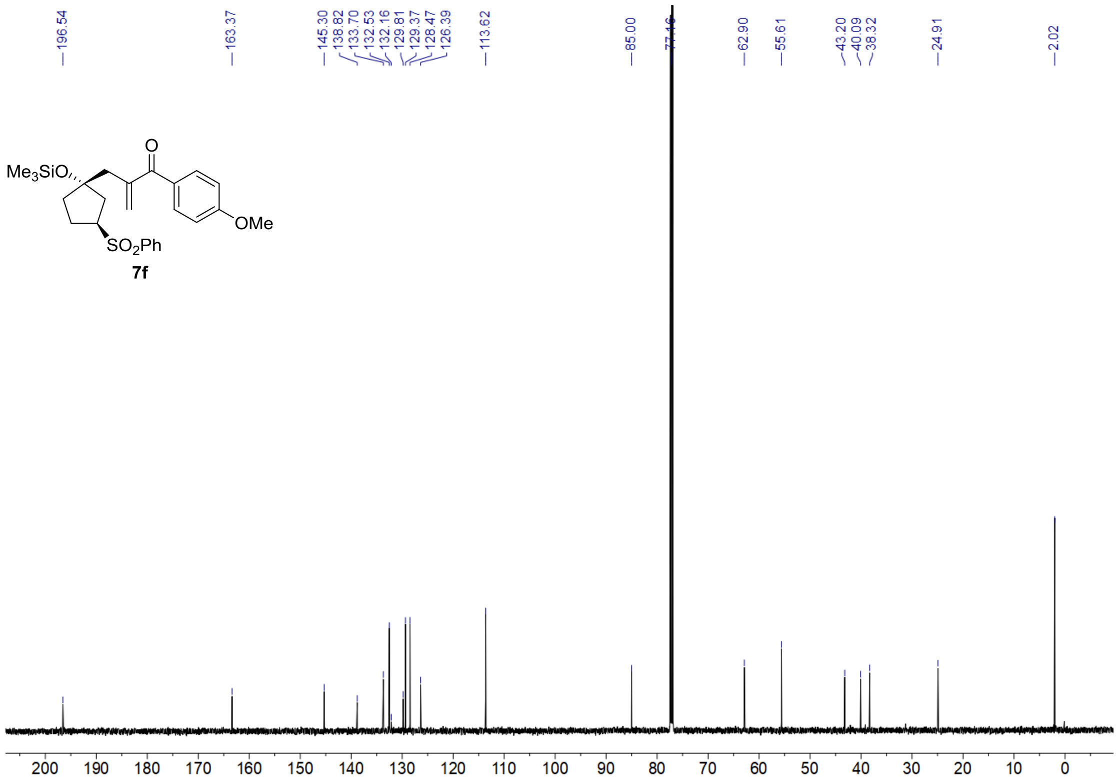

Supplementary Figure 159: ^{13}C NMR of **7f** (151 MHz, $CDCl_3$, 25 ºC)

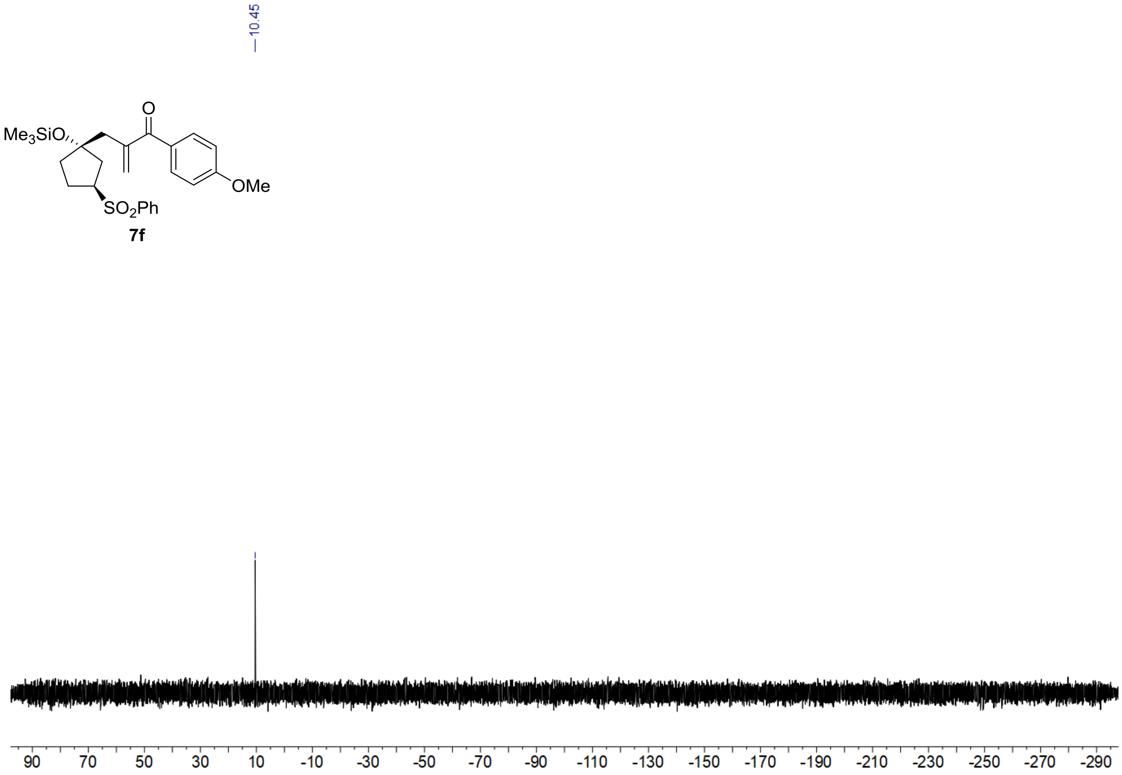

Supplementary Figure 160: ^{29}Si NMR of **7f** (119 MHz, $CDCl_3$, 25 ºC)

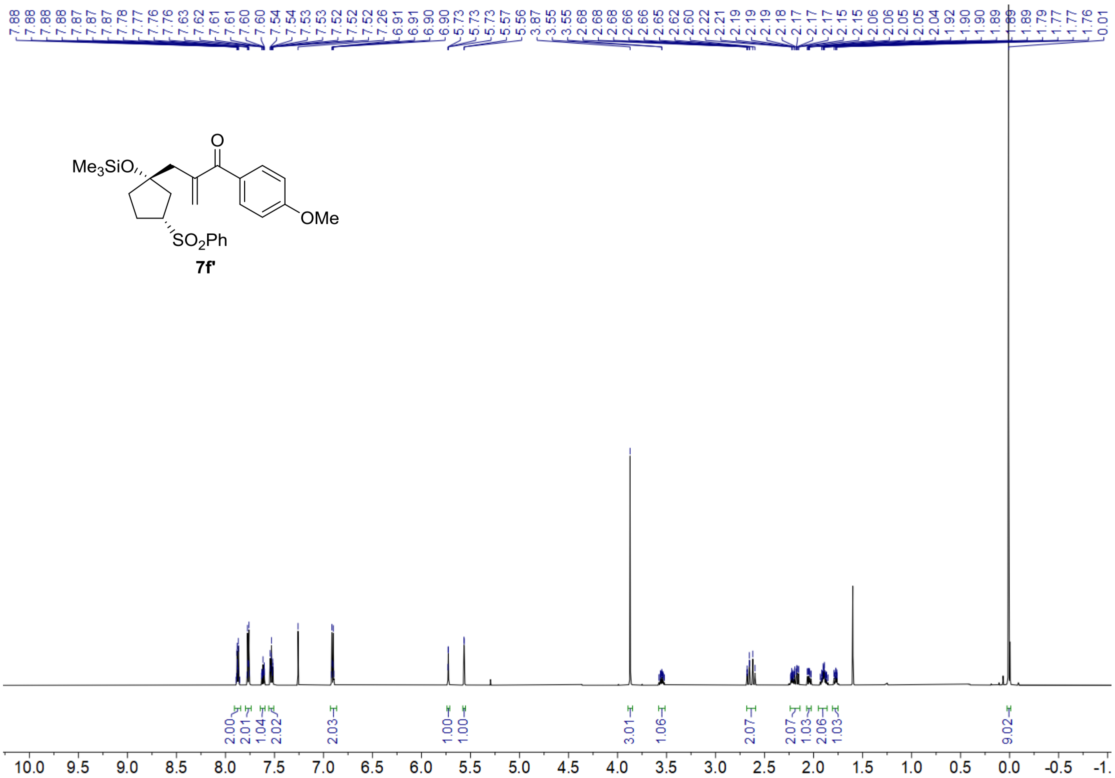

Supplementary Figure 161: ^{1}H NMR of **7f'** (600 MHz, $CDCl_3$, 25 ºC)

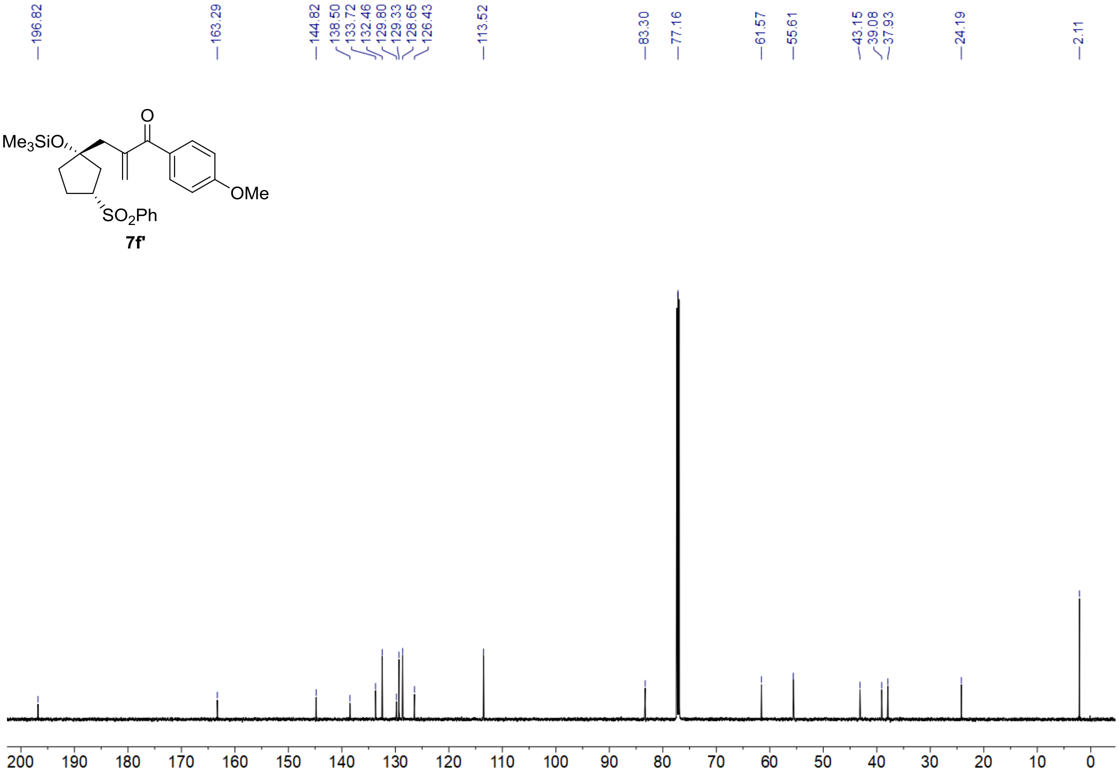

Supplementary Figure 162: ^{13}C NMR of **7f'** (151 MHz, $CDCl_3$, 25 ºC)

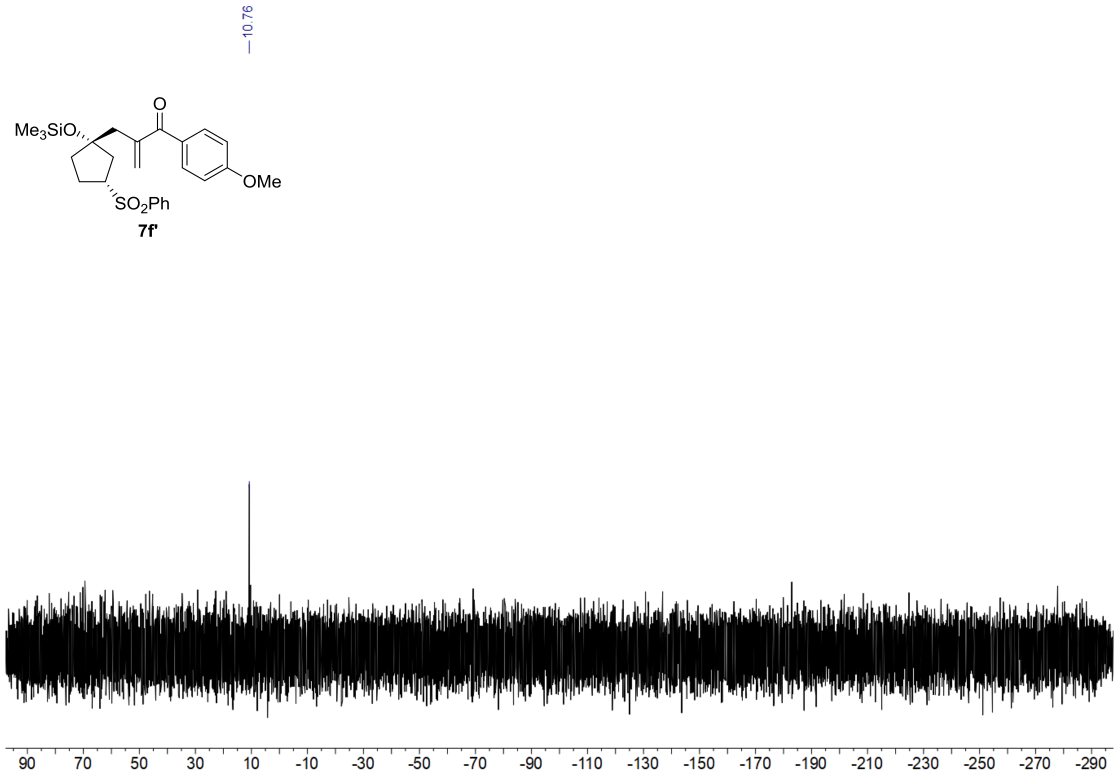

Supplementary Figure 163: ^{29}Si NMR of **7f’** (119 MHz, $CDCl_3$, 25 ºC)

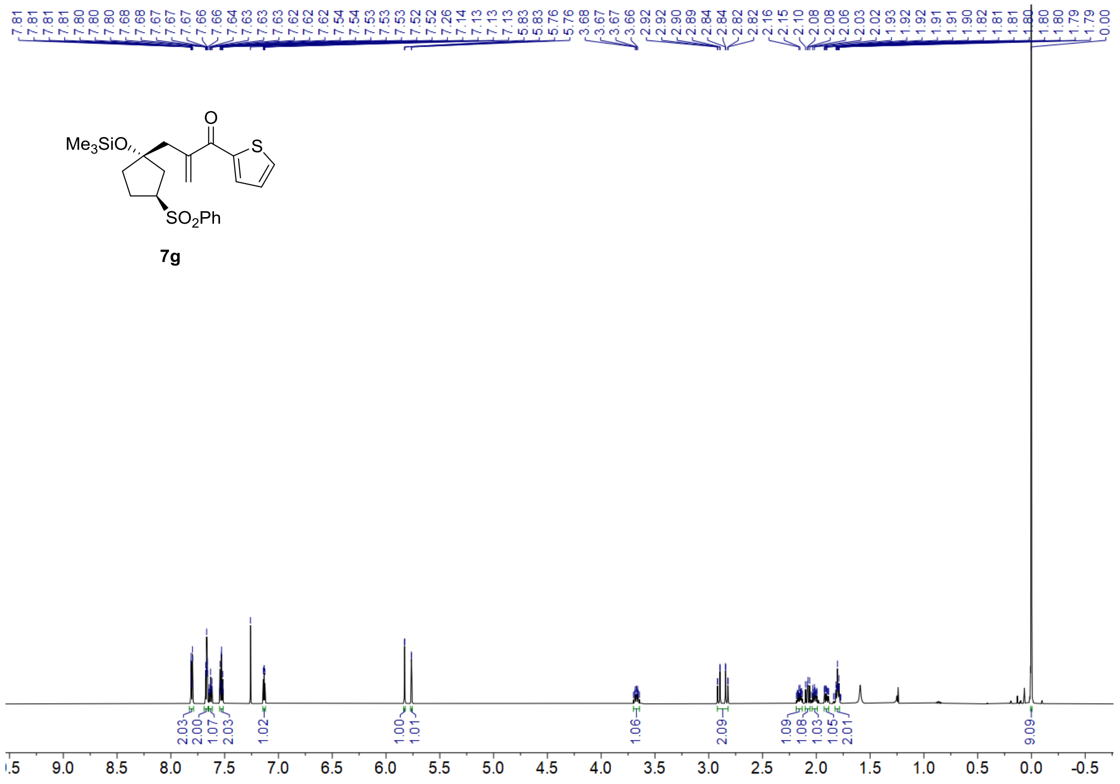

Supplementary Figure 164: ^{1}H NMR of **7g** (600 MHz, $CDCl_3$, 25 ºC)

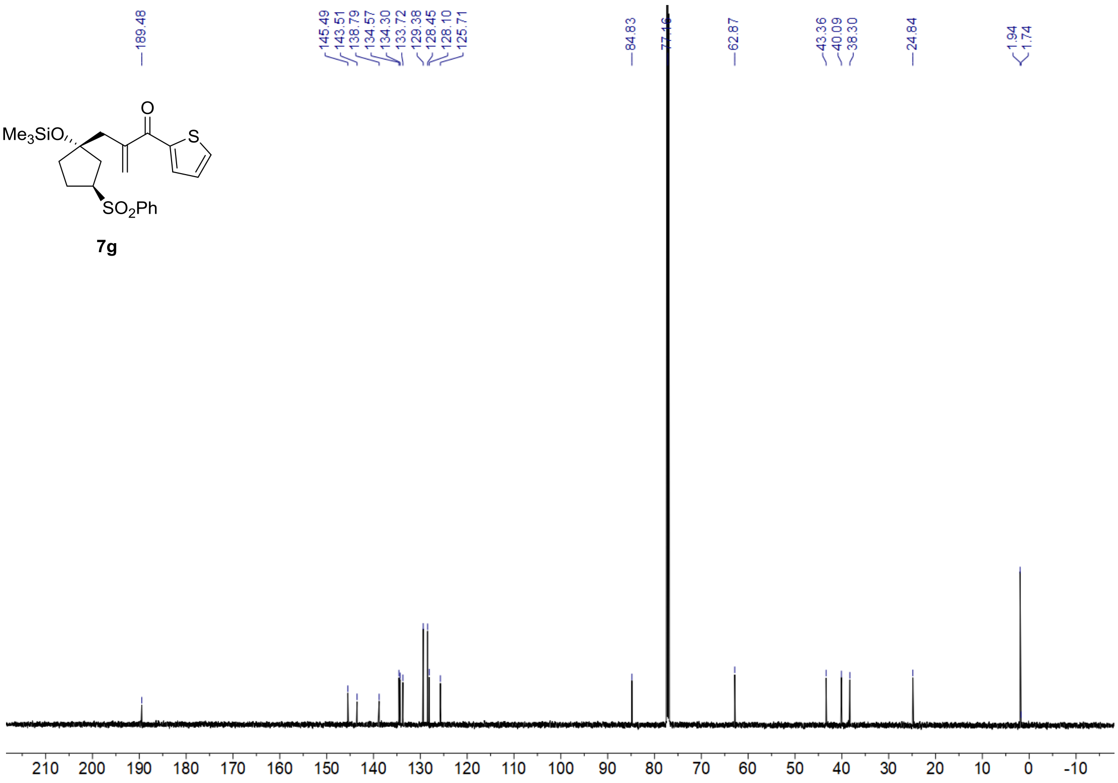

Supplementary Figure 165: ^{13}C NMR of **7g** (151 MHz, $CDCl_3$, 25 ºC)

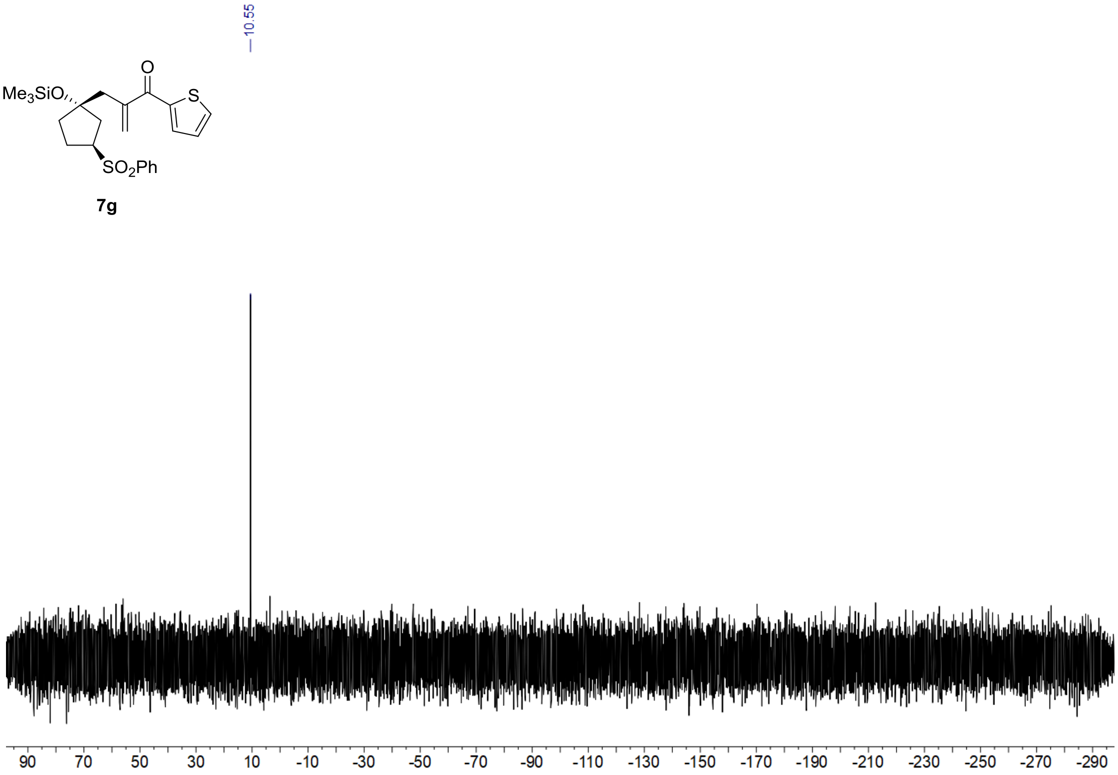

Supplementary Figure 166: ^{29}Si NMR of **7g** (119 MHz, $CDCl_3$, 25 ºC)

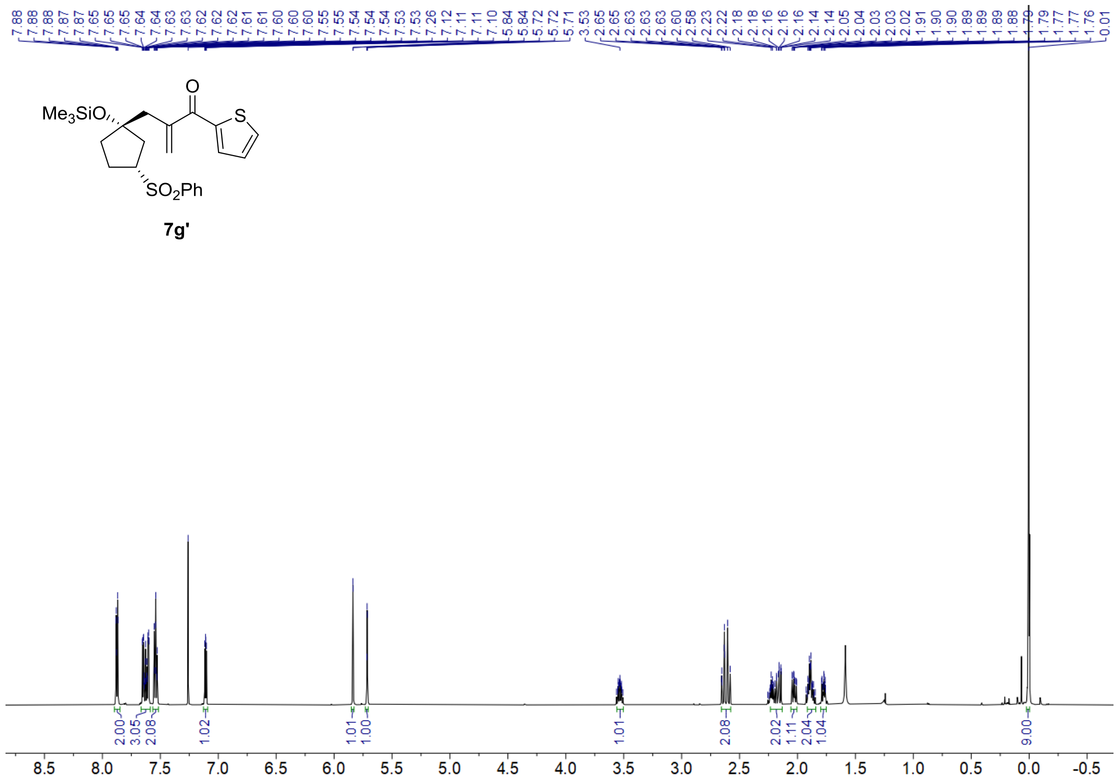

Supplementary Figure 167: ^{1}H NMR of **7g'** (600 MHz, $CDCl_3$, 25 ºC)

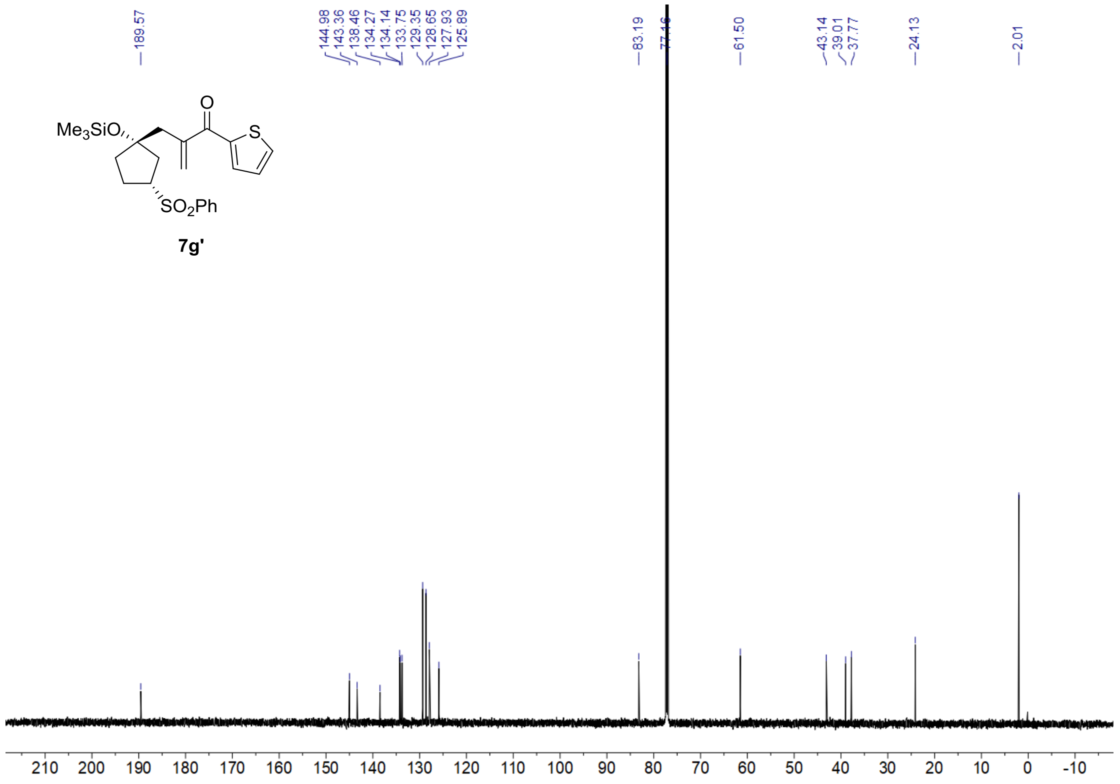

Supplementary Figure 168: ^{13}C NMR of **7g'** (151 MHz, $CDCl_3$, 25 ºC)

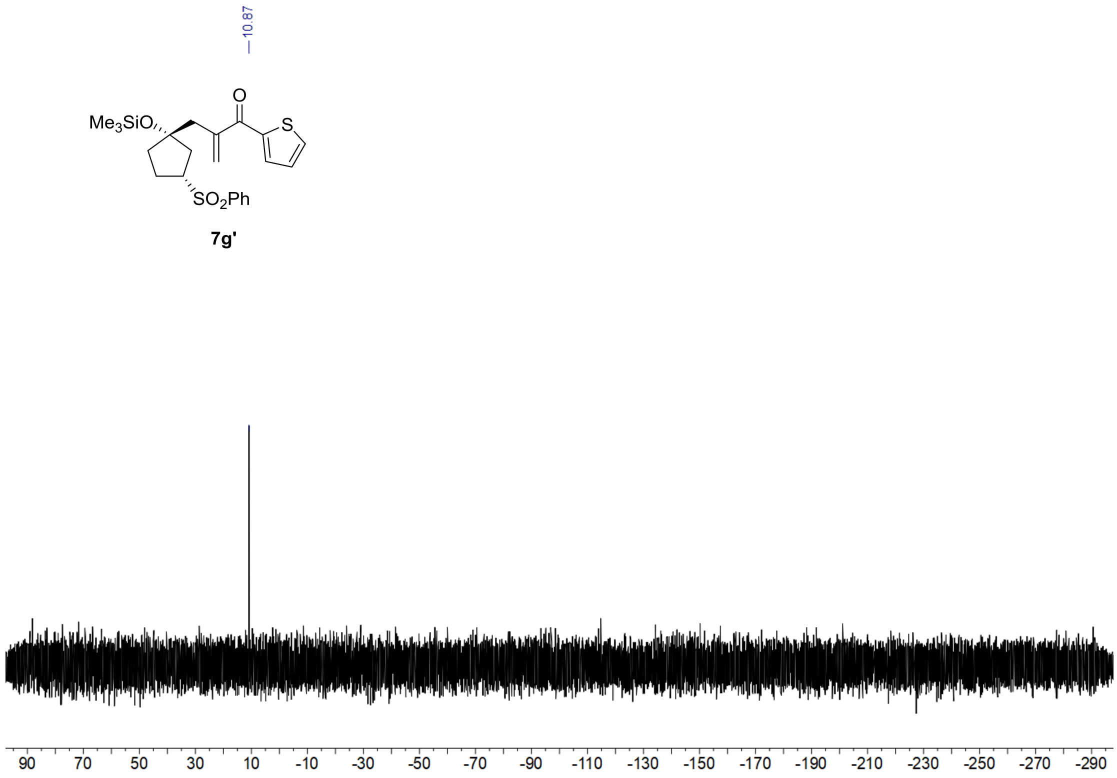

Supplementary Figure 169: ^{29}Si NMR of **7g'** (119 MHz, CDCl$_3$, 25 ºC)

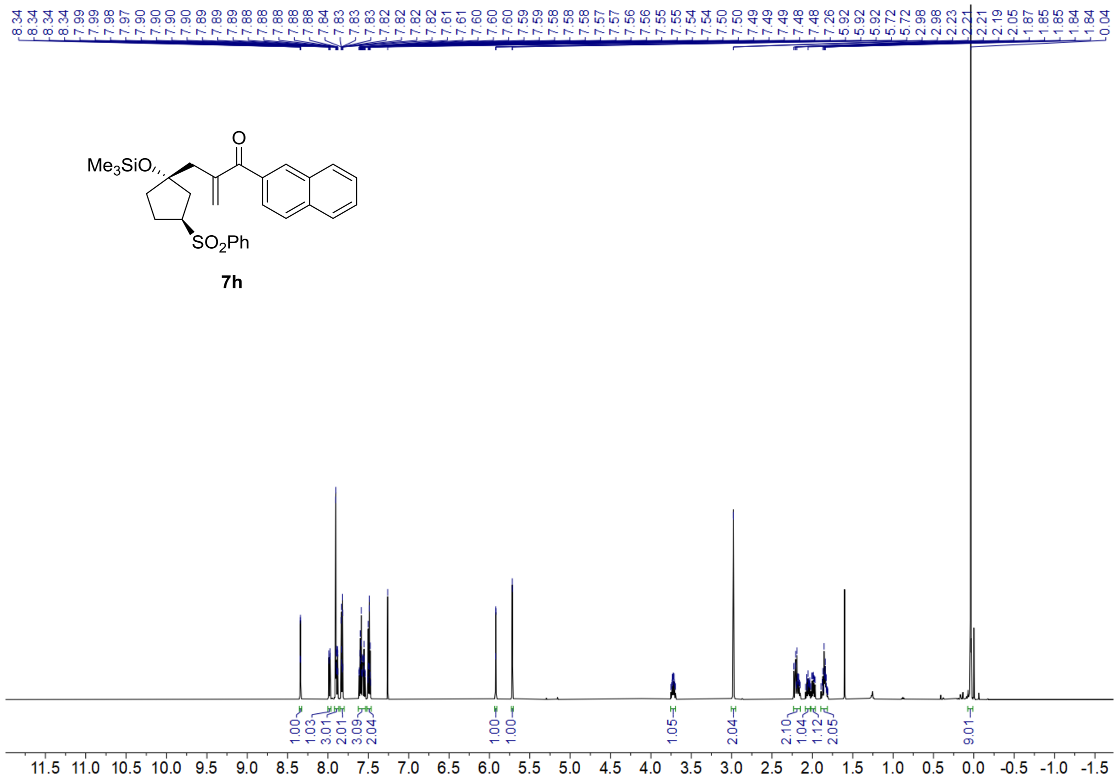

Supplementary Figure 170: ^{1}H NMR of **7h** (600 MHz, CDCl$_3$, 25 ºC)

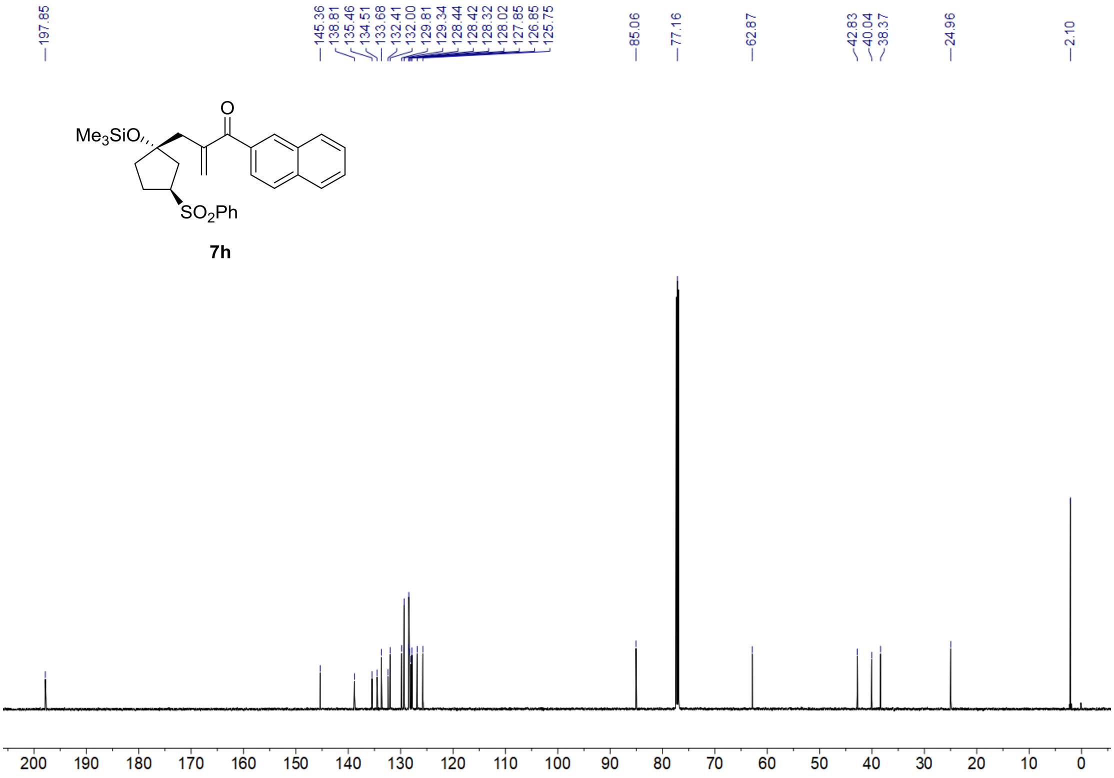

Supplementary Figure 171: ^{13}C NMR of **7h** (151 MHz, $CDCl_3$, 25 ºC)

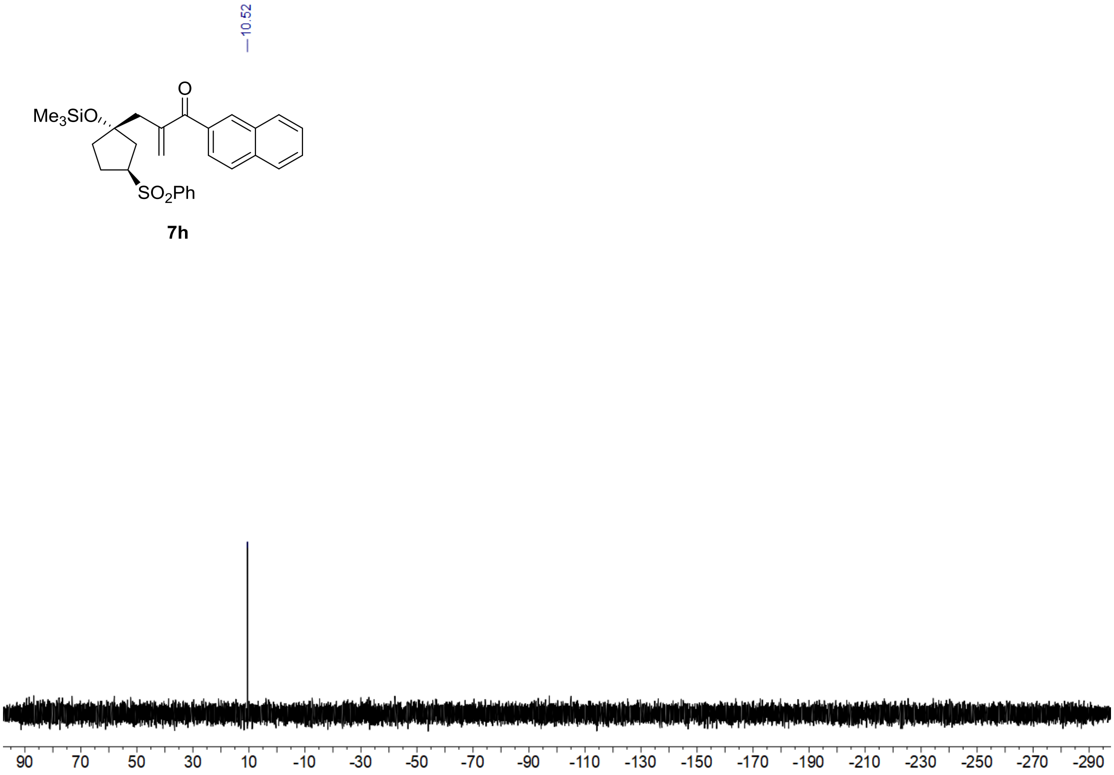

Supplementary Figure 172: ^{29}Si NMR of **7h** (119 MHz, $CDCl_3$, 25 ºC)

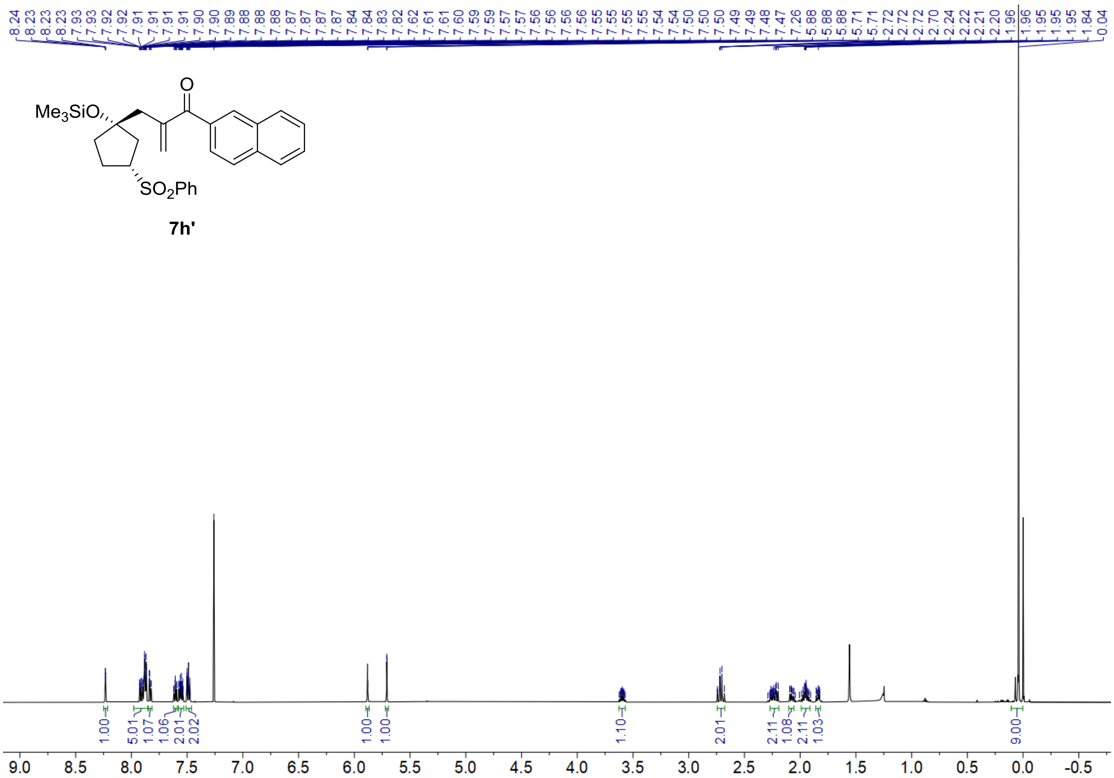

Supplementary Figure 173: ^{1}H NMR of **7h’** (600 MHz, $CDCl_3$, 25 ºC)

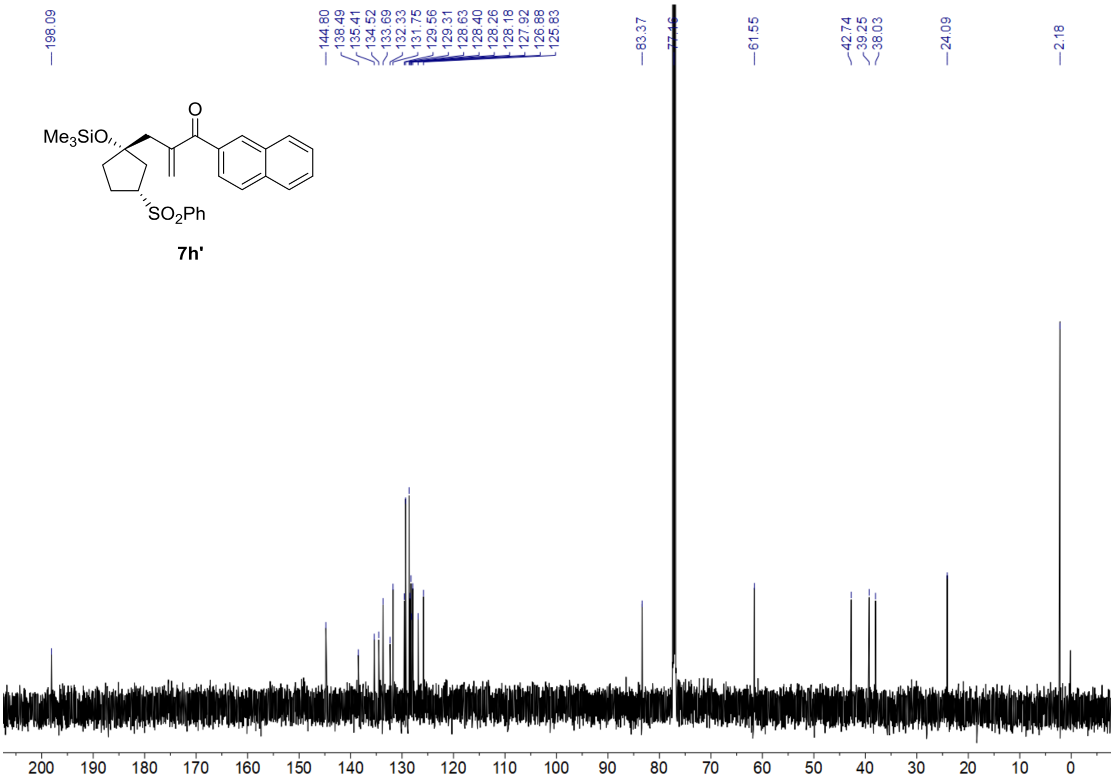

Supplementary Figure 174: ^{13}C NMR of **7h’** (151 MHz, $CDCl_3$, 25 ºC)

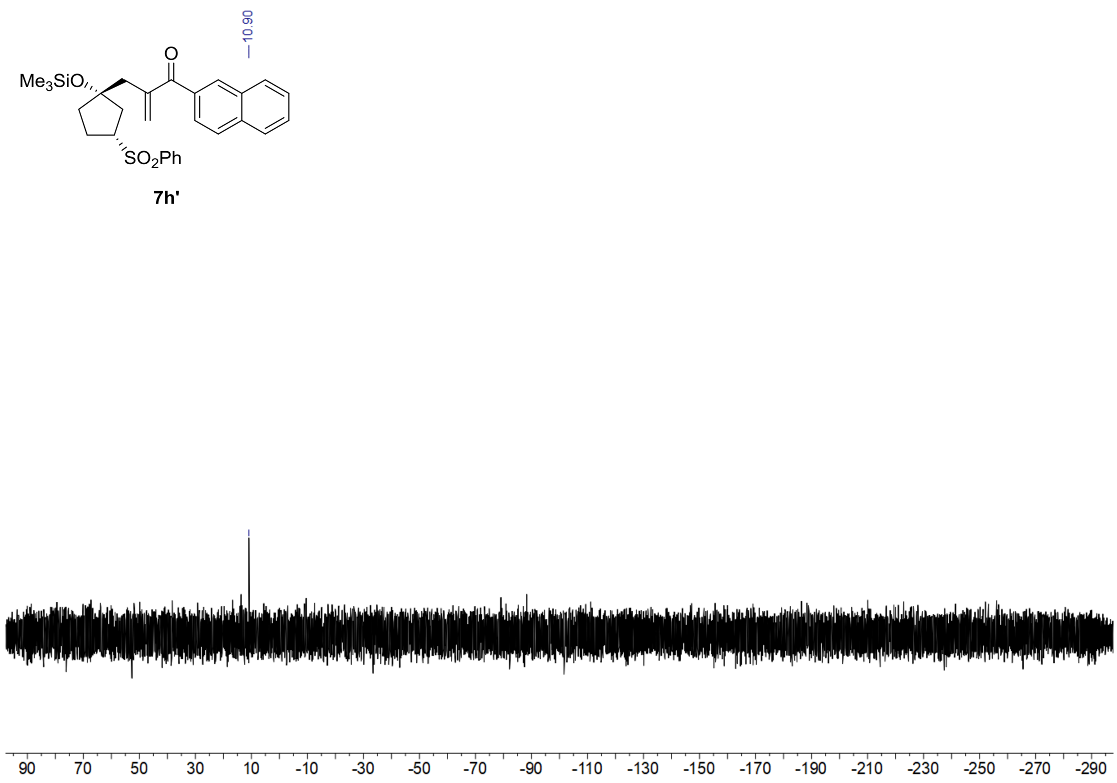

Supplementary Figure 175: ^{29}Si NMR of **7h’** (119 MHz, $CDCl_3$, 25 ºC)

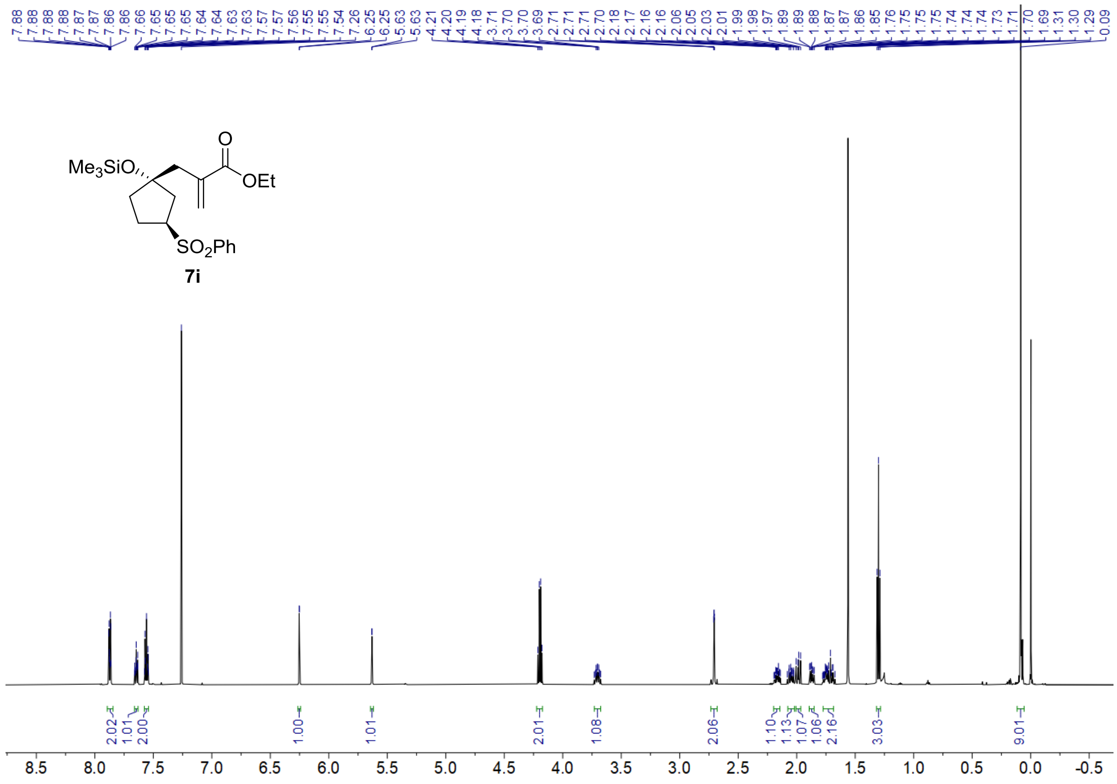

Supplementary Figure 176: ^{1}H NMR of **7i** (600 MHz, $CDCl_3$, 25 ºC)

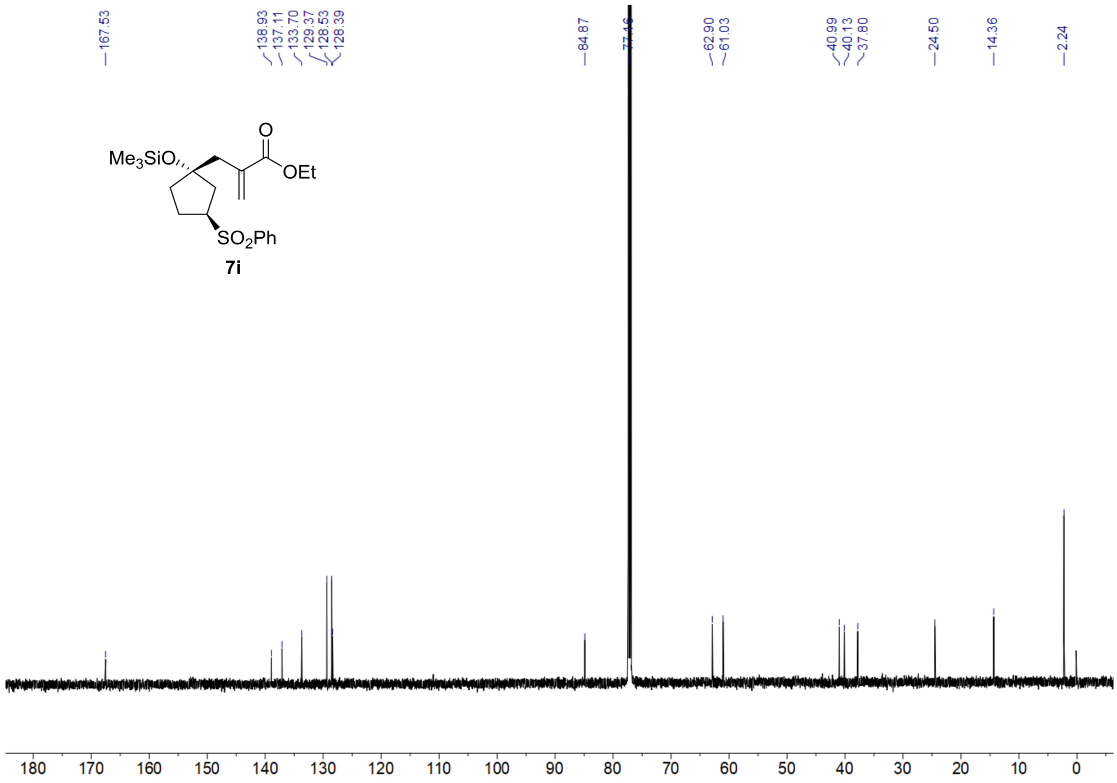

Supplementary Figure 177: ^{13}C NMR of **7i** (151 MHz, CDCl$_3$, 25 ºC)

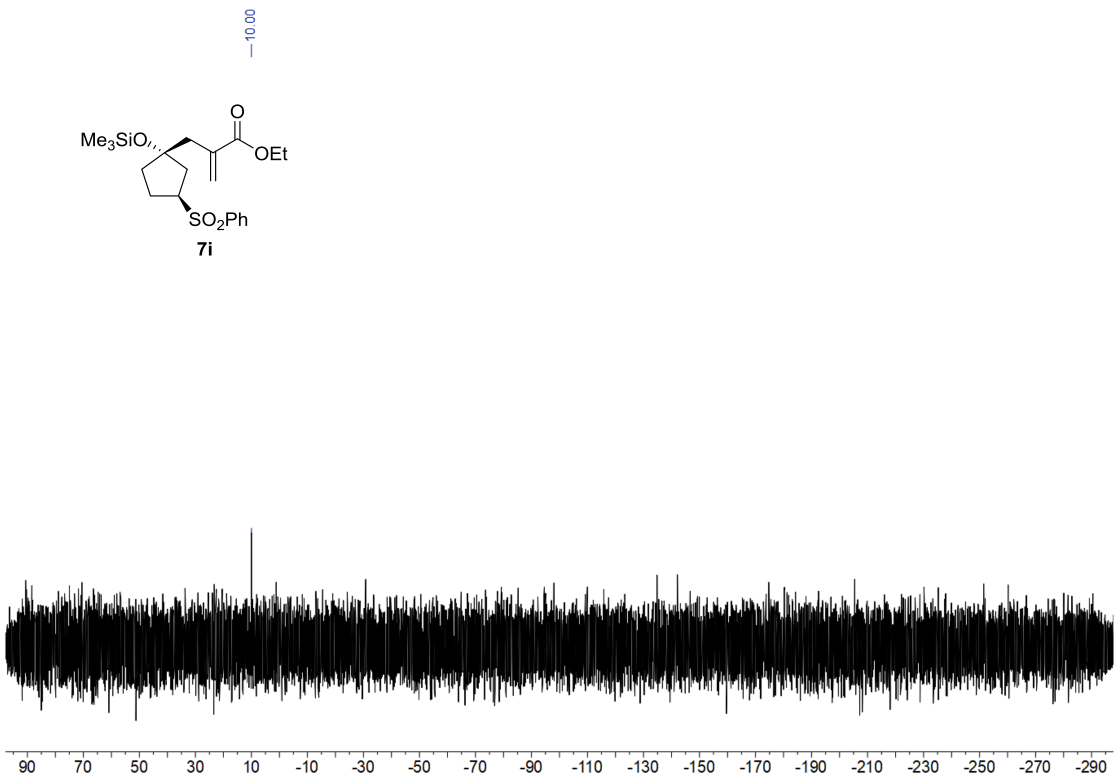

Supplementary Figure 178: ^{29}Si NMR of **7i** (119 MHz, CDCl$_3$, 25 ºC)

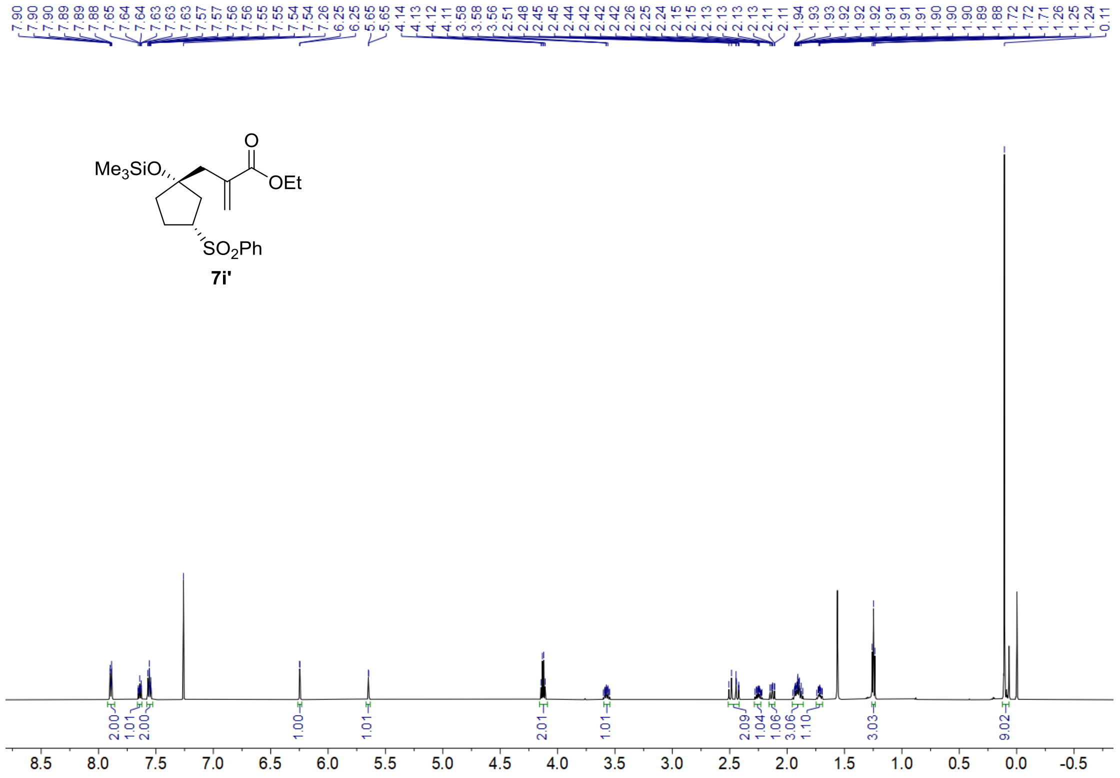

Supplementary Figure 179: ^{1}H NMR of **7i’** (600 MHz, $CDCl_3$, 25 ºC)

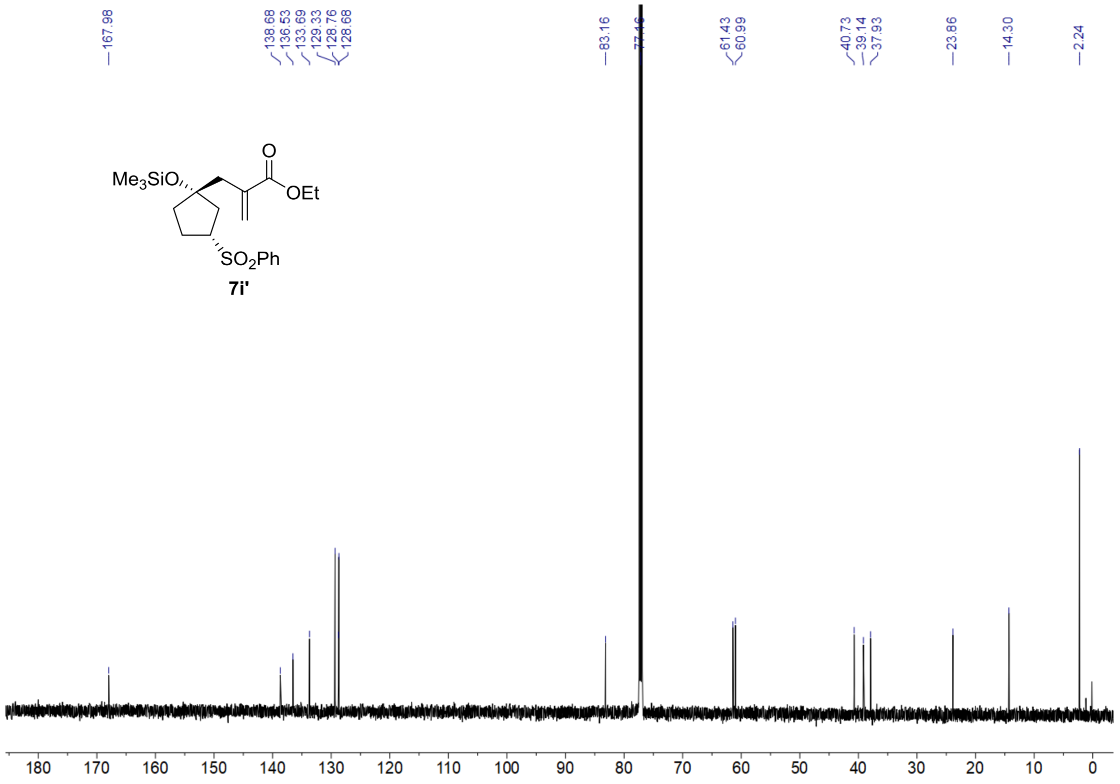

Supplementary Figure 180: ^{13}C NMR of **7i’** (151 MHz, $CDCl_3$, 25 ºC)

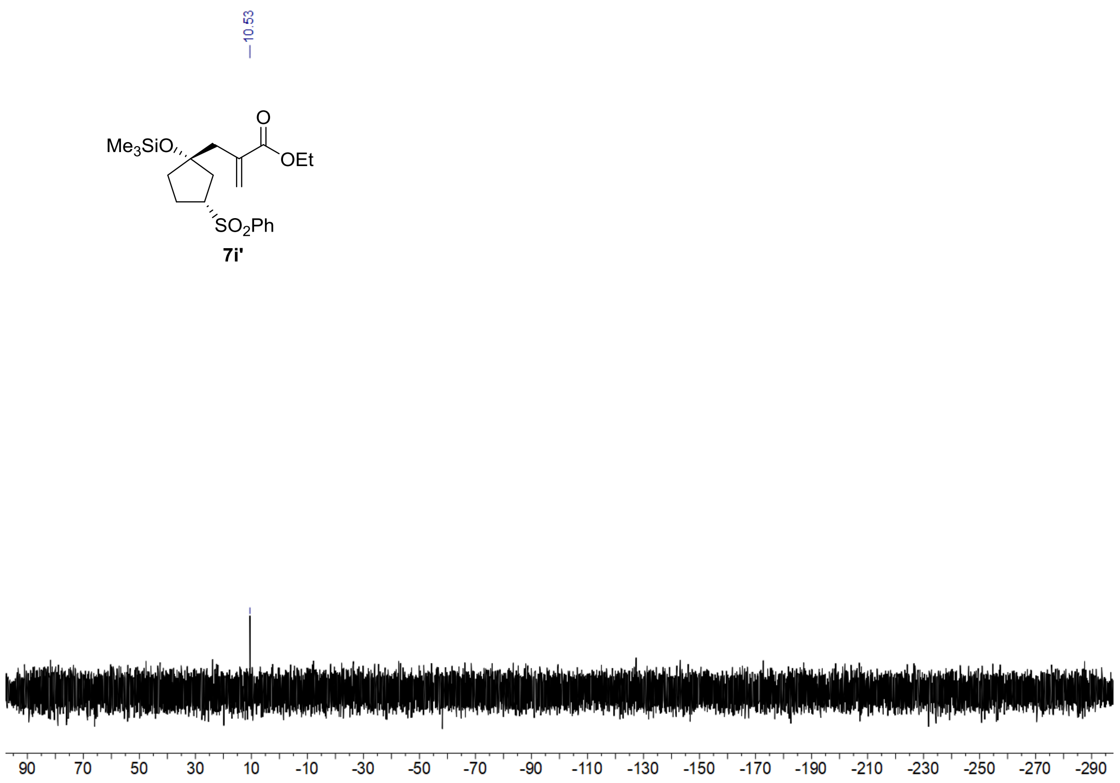

Supplementary Figure 181: ^{29}Si NMR of **7i’** (119 MHz, $CDCl_3$, 25 ºC)

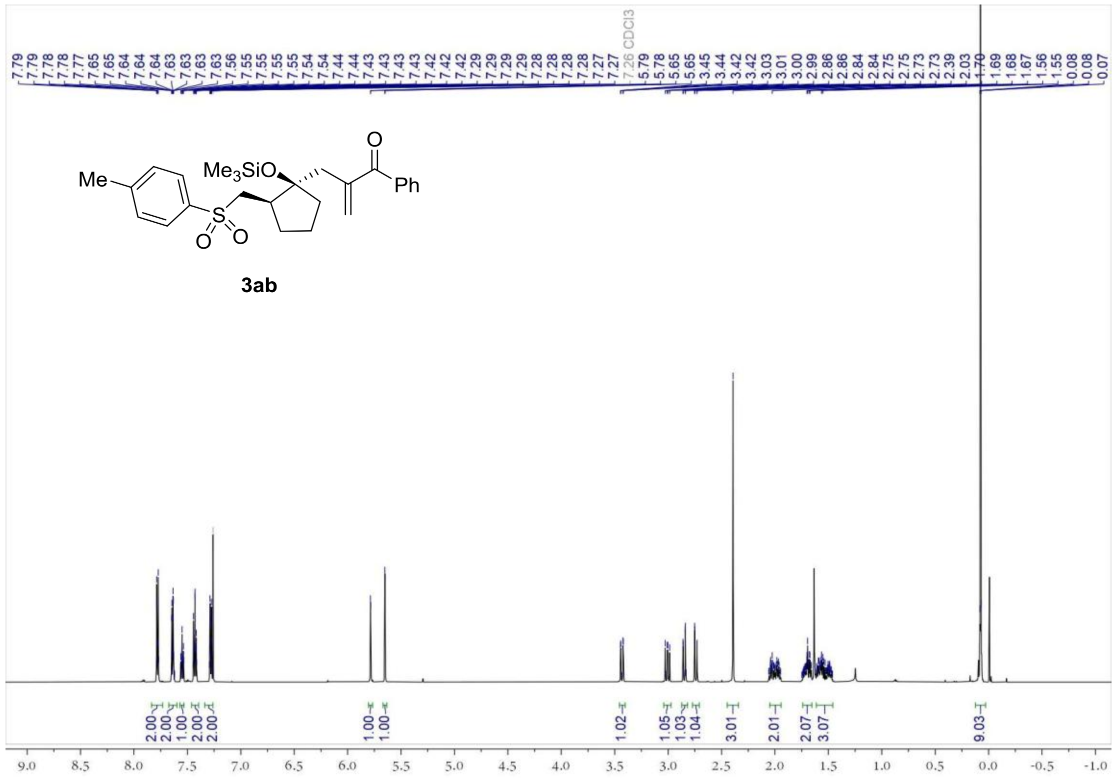

Supplementary Figure 182: ^{1}H NMR of **3ab** (600 MHz, $CDCl_3$, 25 ºC)

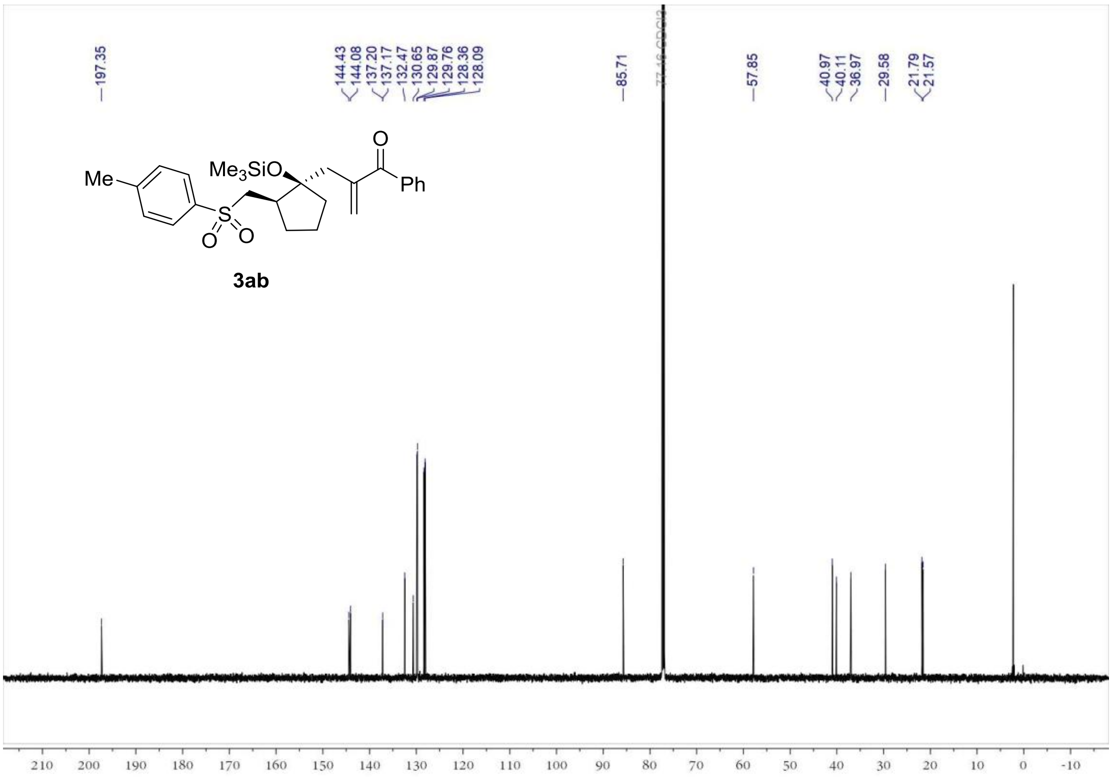

Supplementary Figure 183: ^{13}C NMR of **3ab** (151 MHz, $CDCl_3$, 25 ºC)

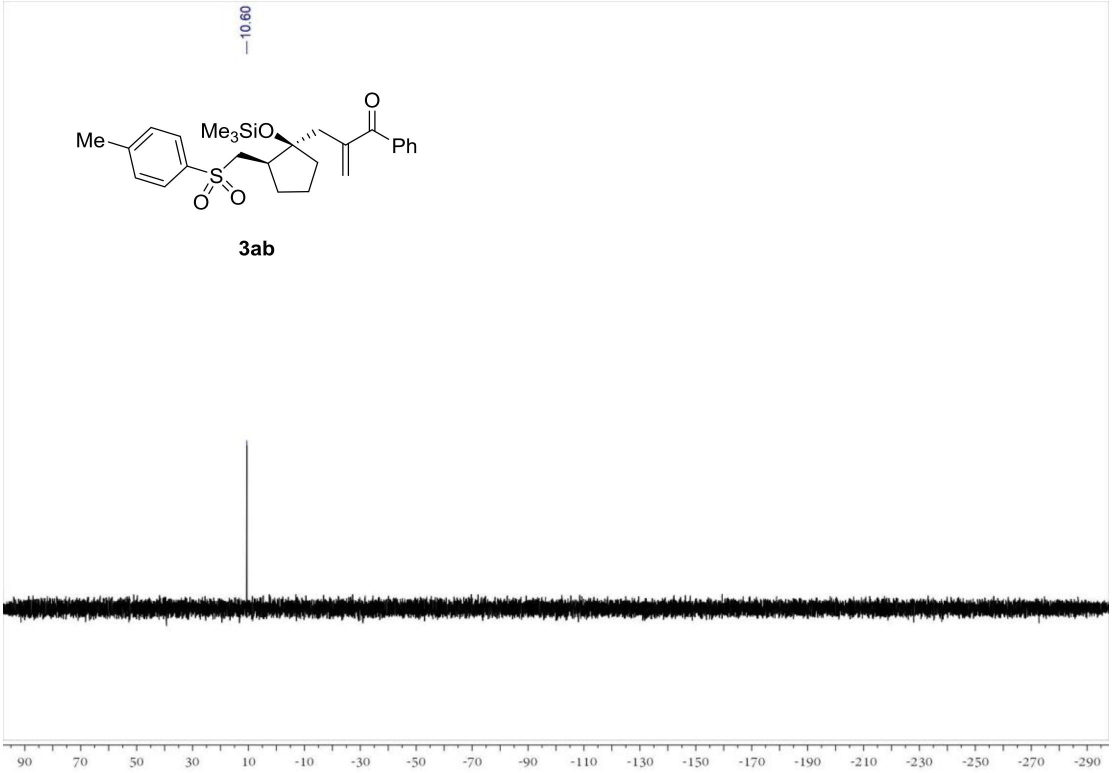

Supplementary Figure 184: ^{29}Si NMR of **3ab** (119 MHz, $CDCl_3$, 25 ºC)

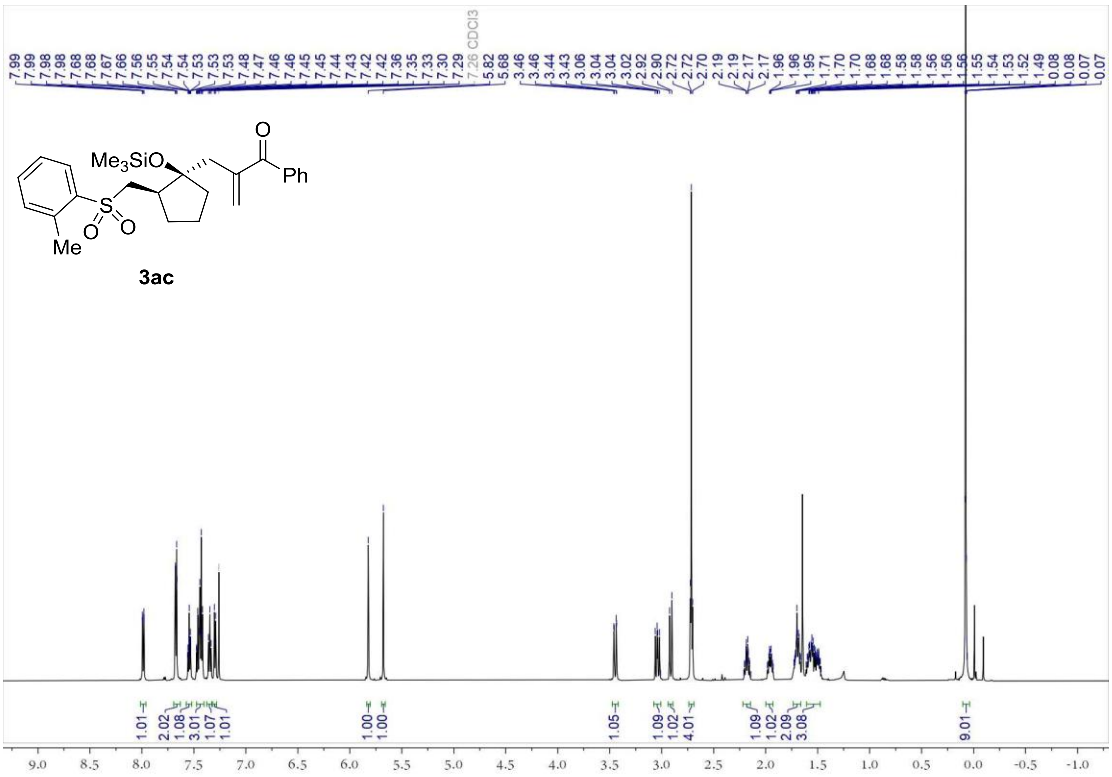

Supplementary Figure 185: ^{1}H NMR of **3ac** (600 MHz, $CDCl_3$, 25 ºC)

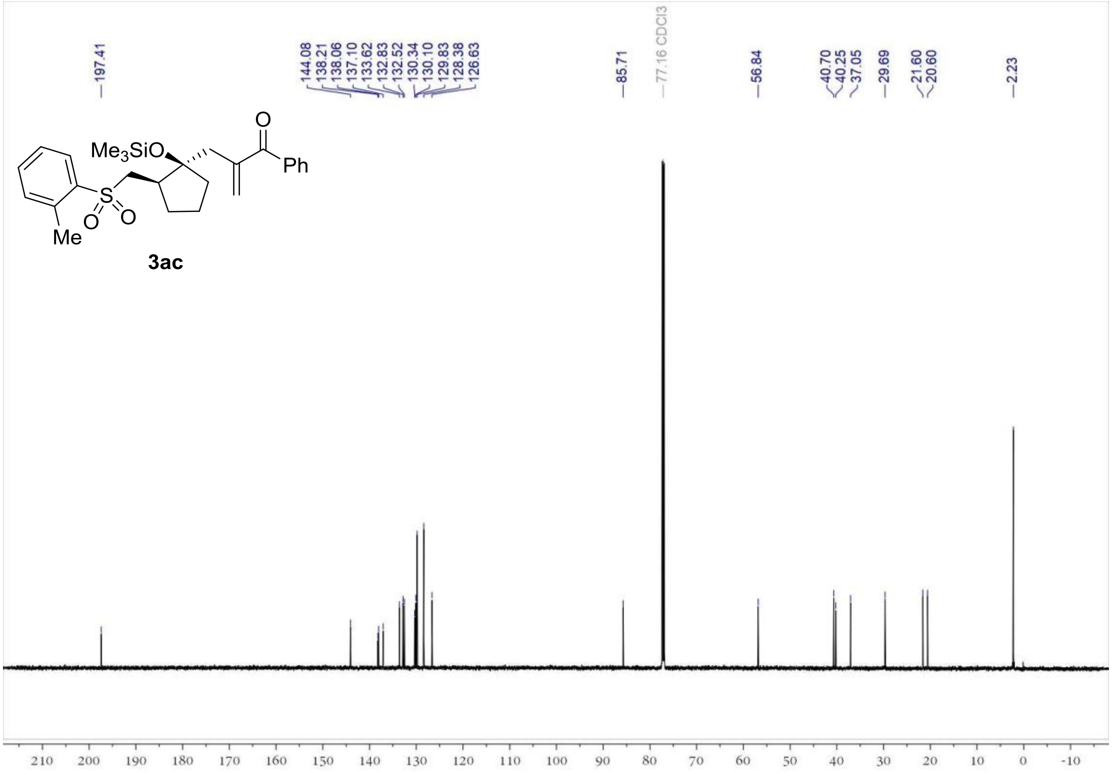

Supplementary Figure 186: ^{13}C NMR of **3ac** (151 MHz, $CDCl_3$, 25 ºC)

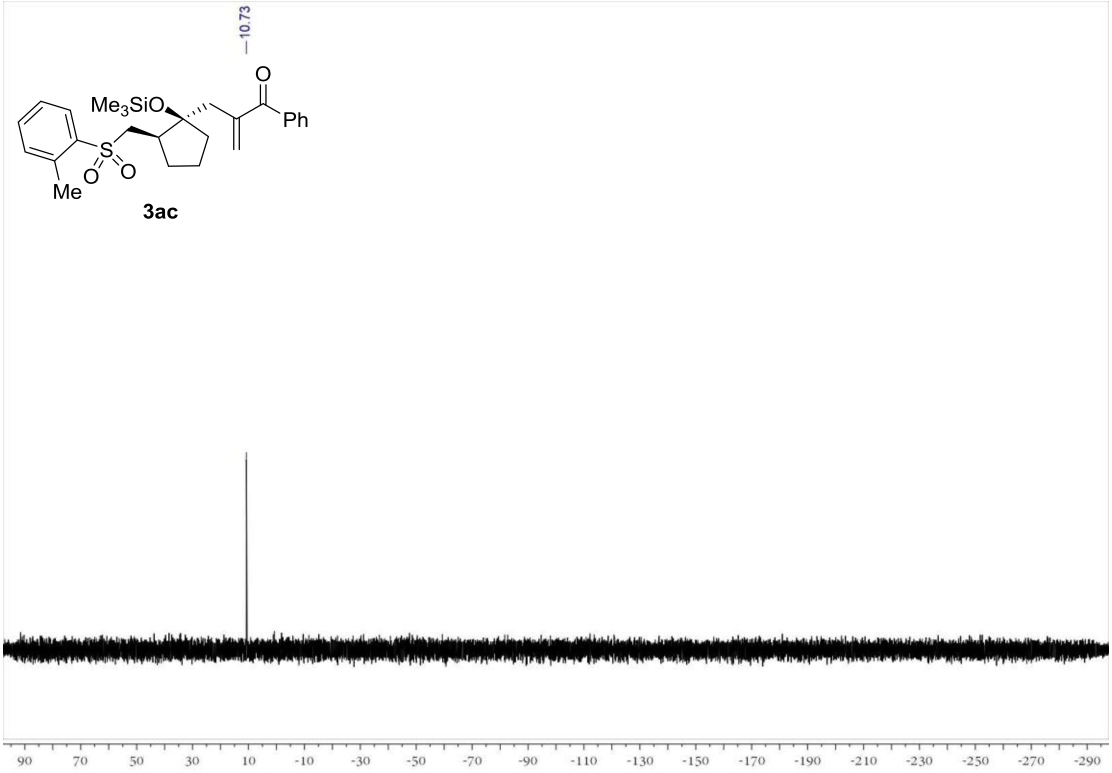

Supplementary Figure 187: ^{29}Si NMR of **3ac** (119 MHz, $CDCl_3$, 25 ºC)

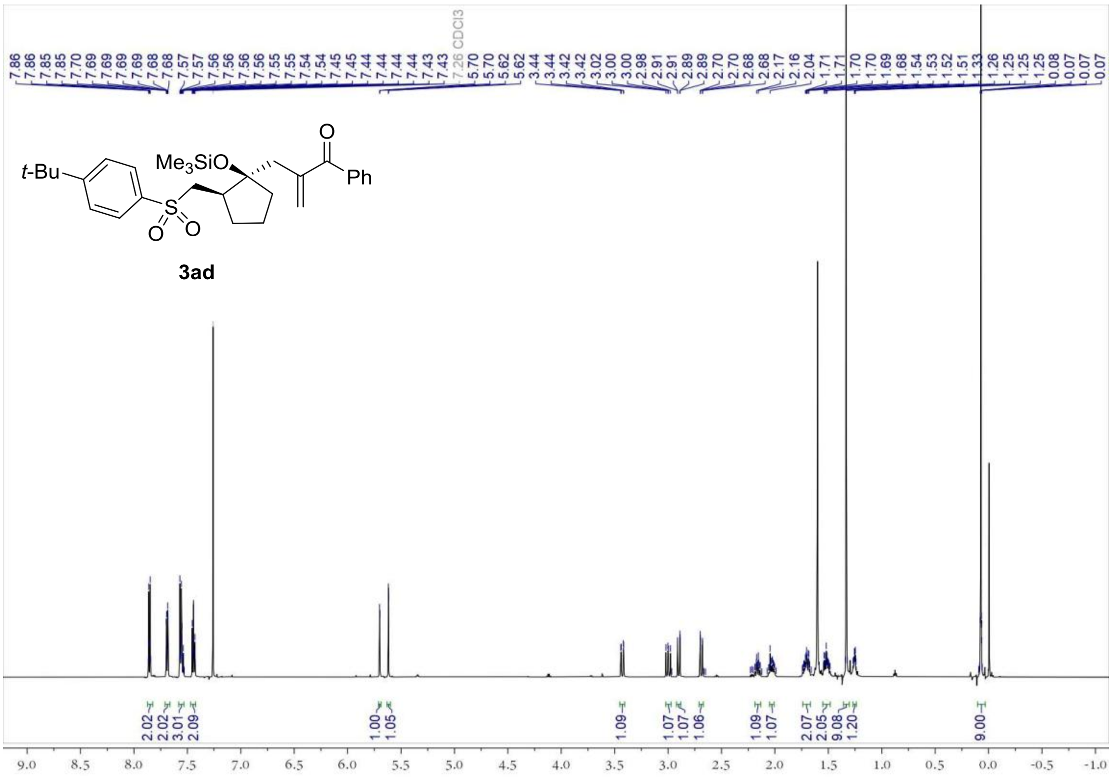

Supplementary Figure 188: ^{1}H NMR of **3ad** (600 MHz, $CDCl_3$, 25 ºC)

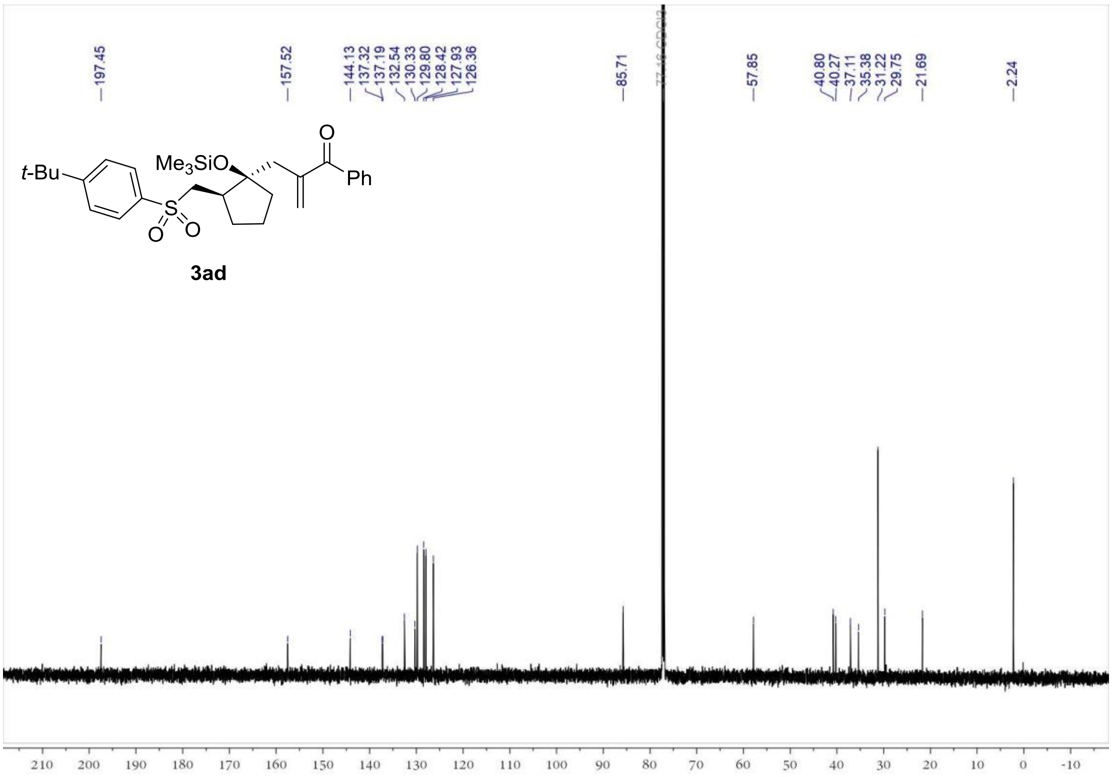

Supplementary Figure 189: ^{13}C NMR of **3ad** (151 MHz, $CDCl_3$, 25 ºC)

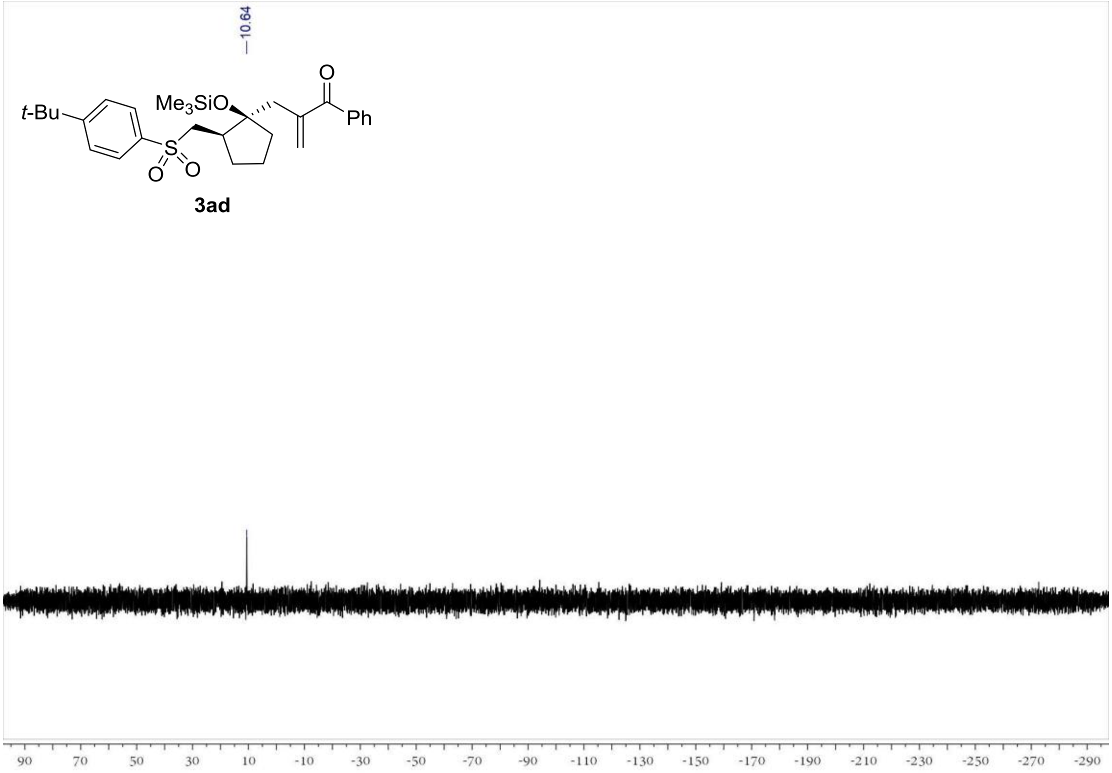

Supplementary Figure 190: ^{29}Si NMR of **3ad** (119 MHz, $CDCl_3$, 25 ºC)

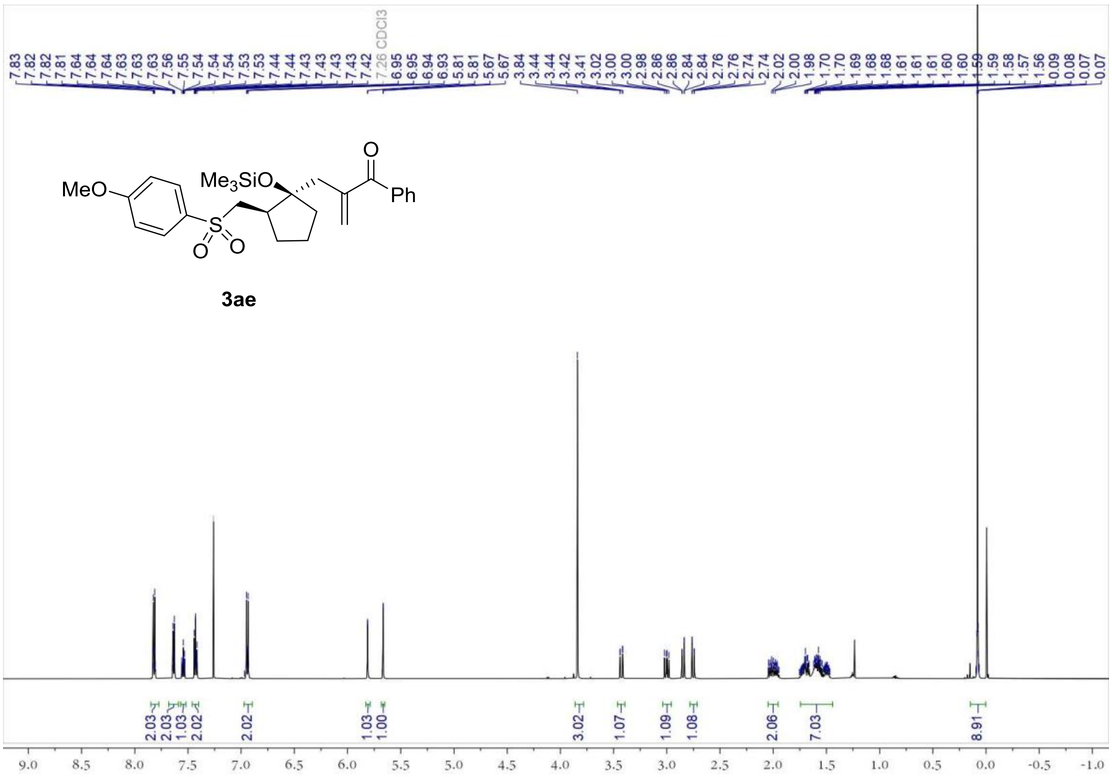

Supplementary Figure 191: ^{1}H NMR of **3ae** (600 MHz, $CDCl_3$, 25 ºC)

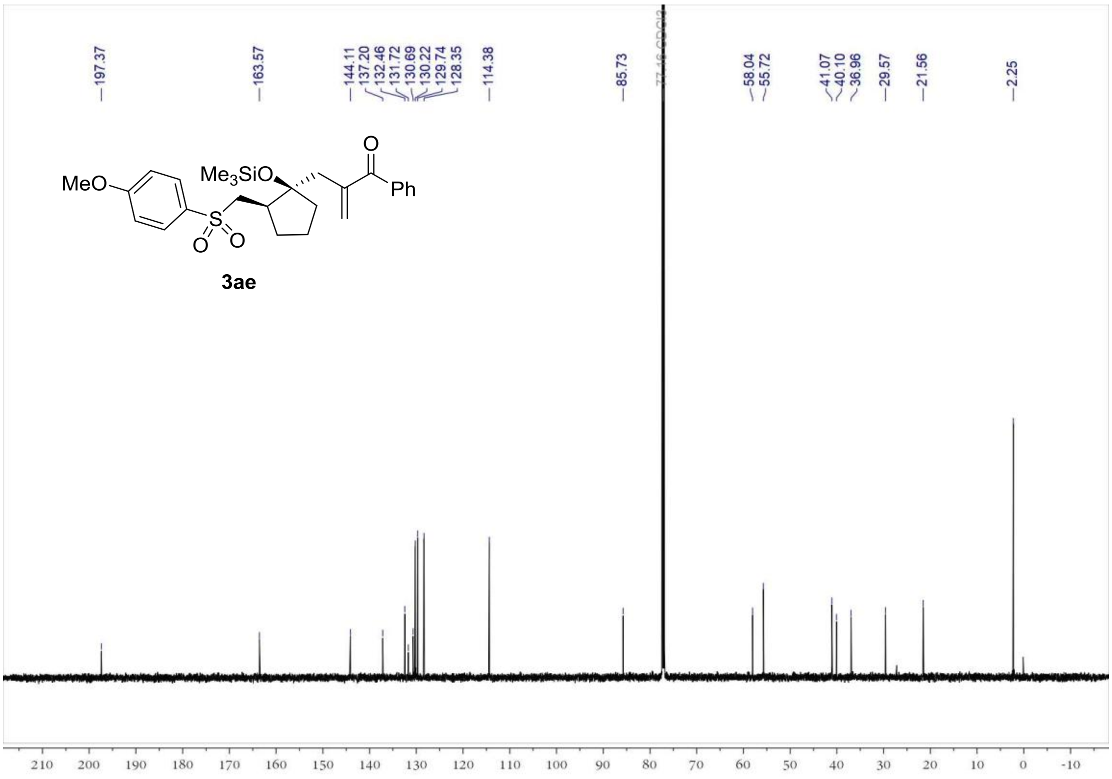

Supplementary Figure 192: ^{13}C NMR of **3ae** (151 MHz, $CDCl_3$, 25 ºC)

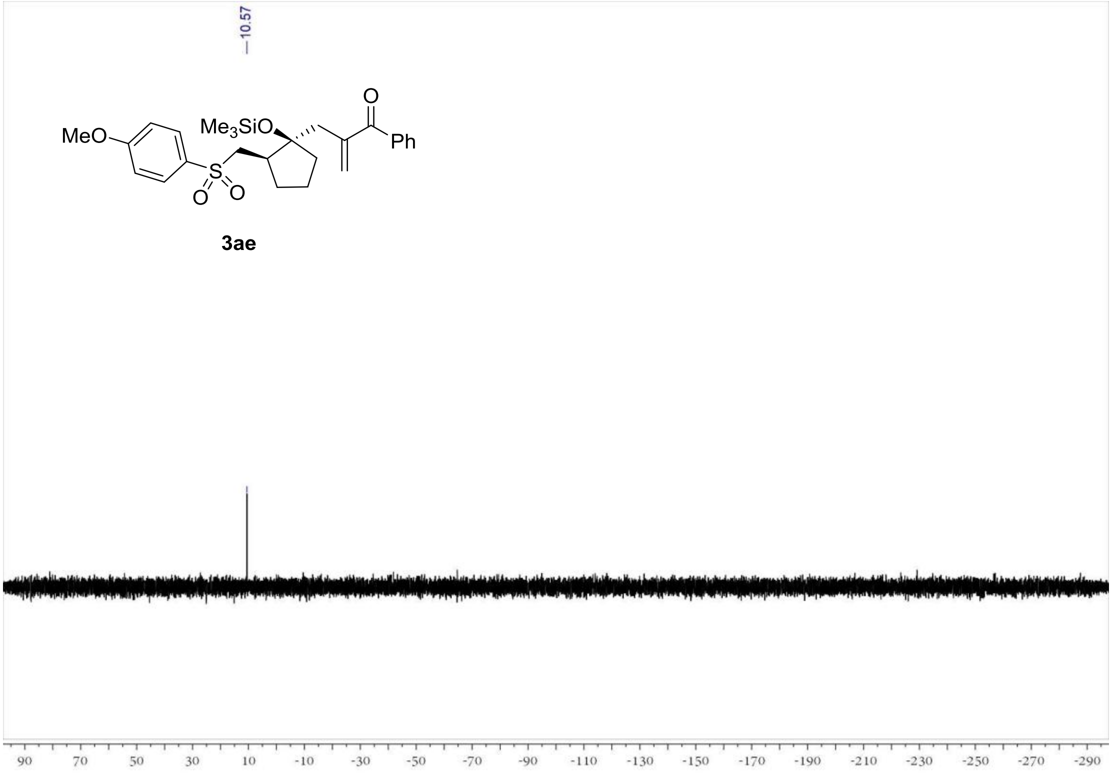

Supplementary Figure 193: ^{29}Si NMR of **3ae** (119 MHz, $CDCl_3$, 25 ºC)

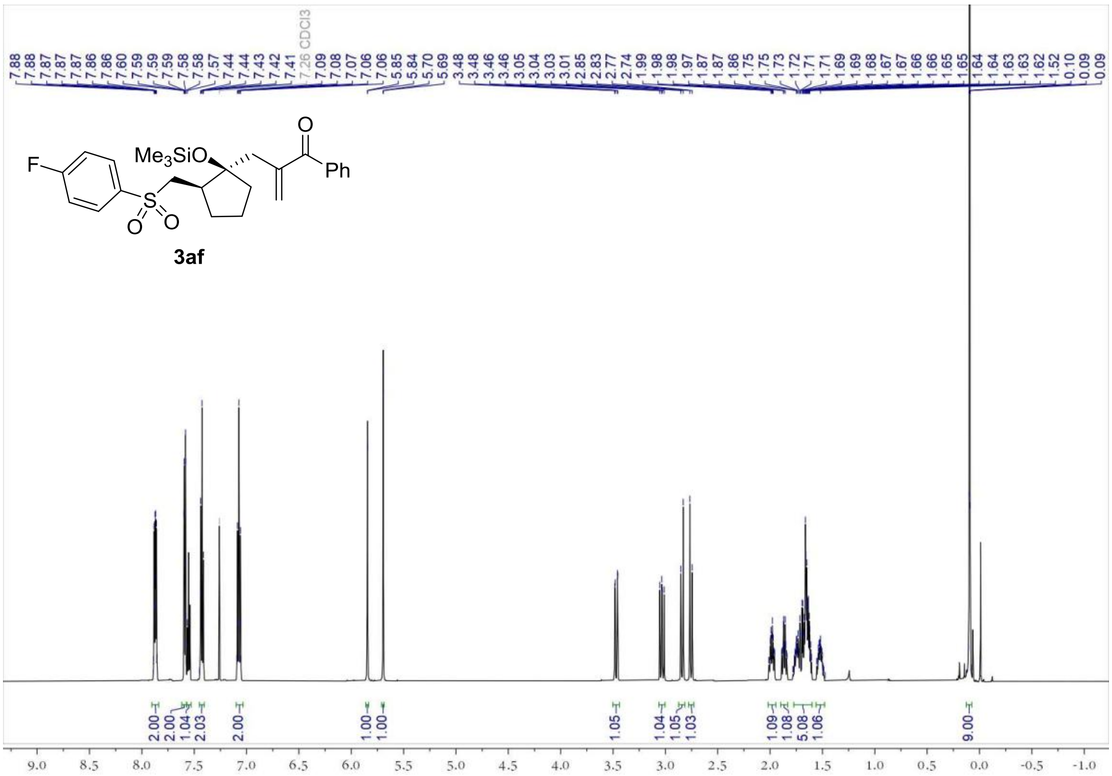

Supplementary Figure 194: ^{1}H NMR of **3af** (600 MHz, $CDCl_3$, 25 ºC)

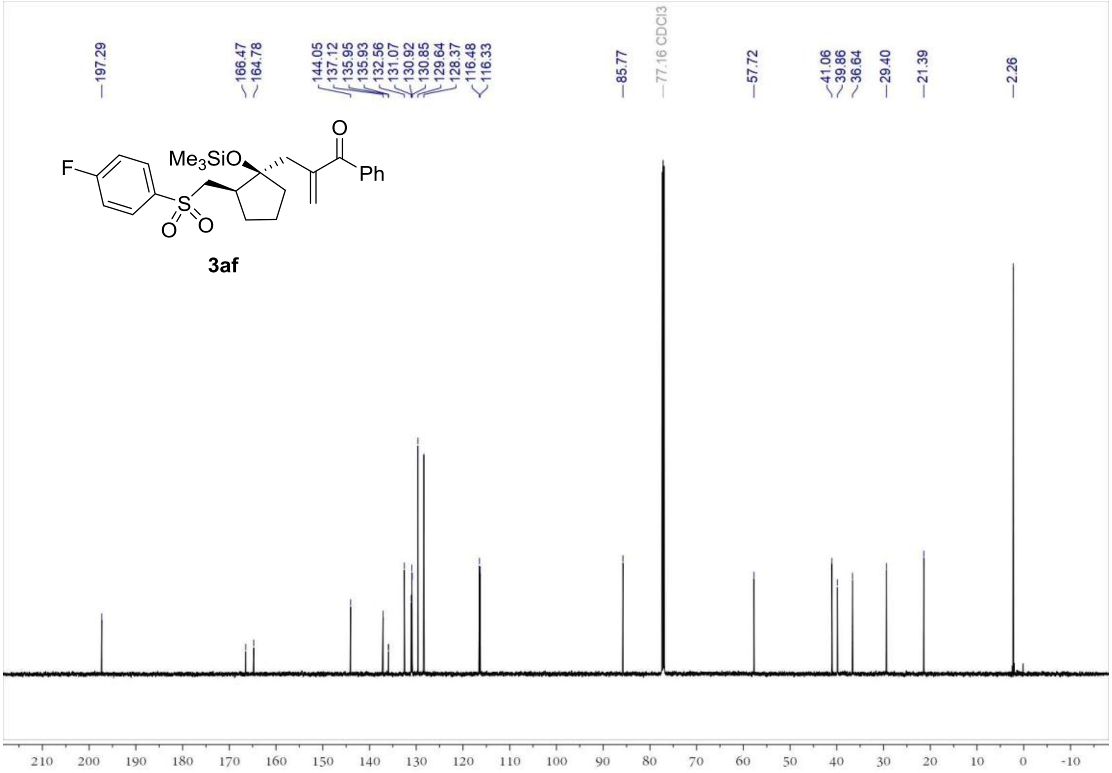

Supplementary Figure 195: ^{13}C NMR of **3af** (151 MHz, $CDCl_3$, 25 ºC)

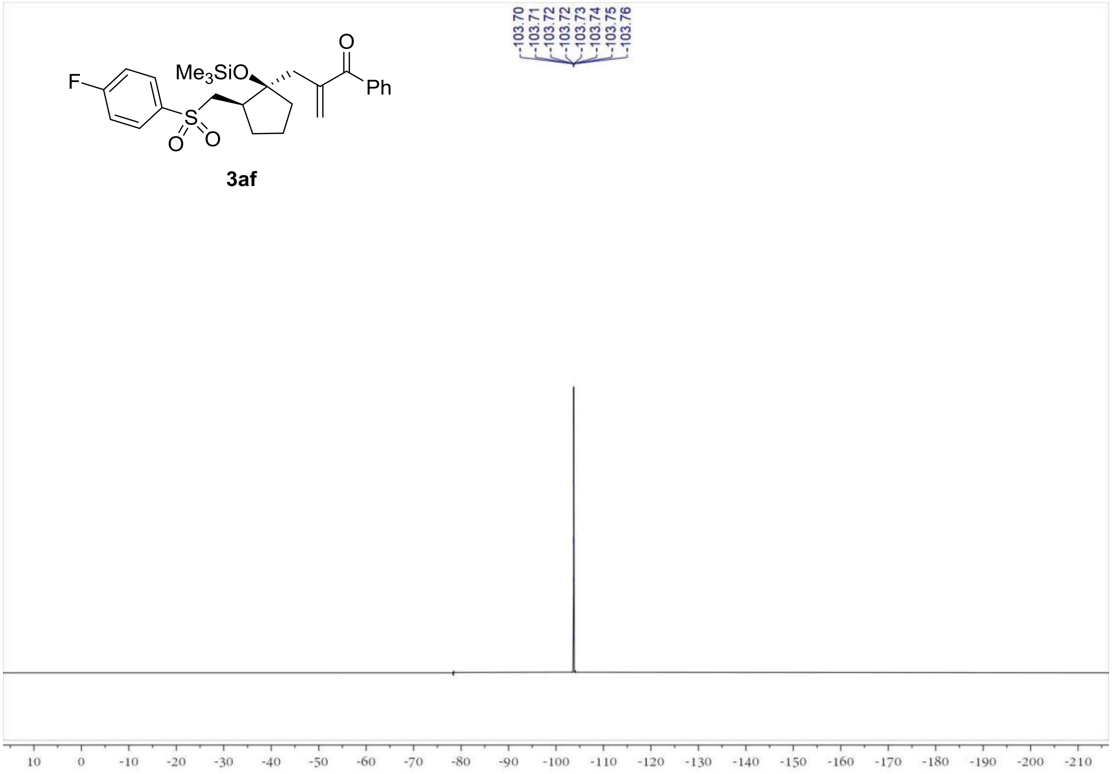

Supplementary Figure 196: ^{19}F NMR of **3af** (565 MHz, $CDCl_3$, 25 ºC)

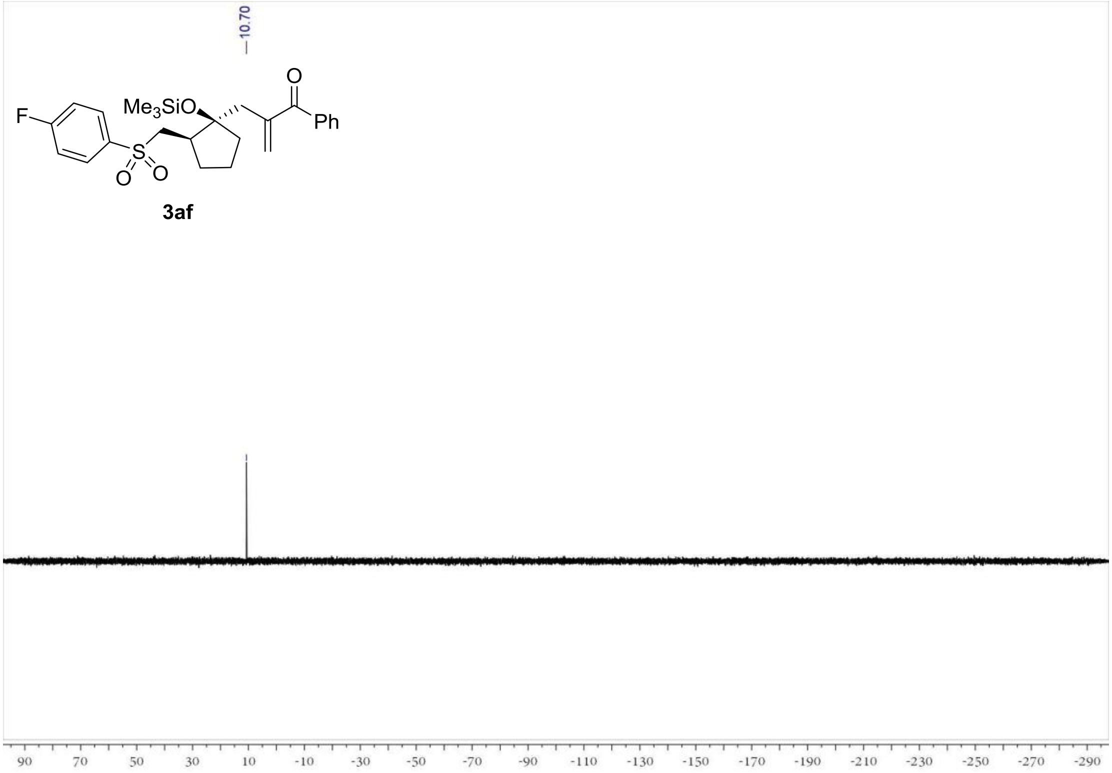

Supplementary Figure 197: ^{29}Si NMR of **3af** (119 MHz, $CDCl_3$, 25 ºC)

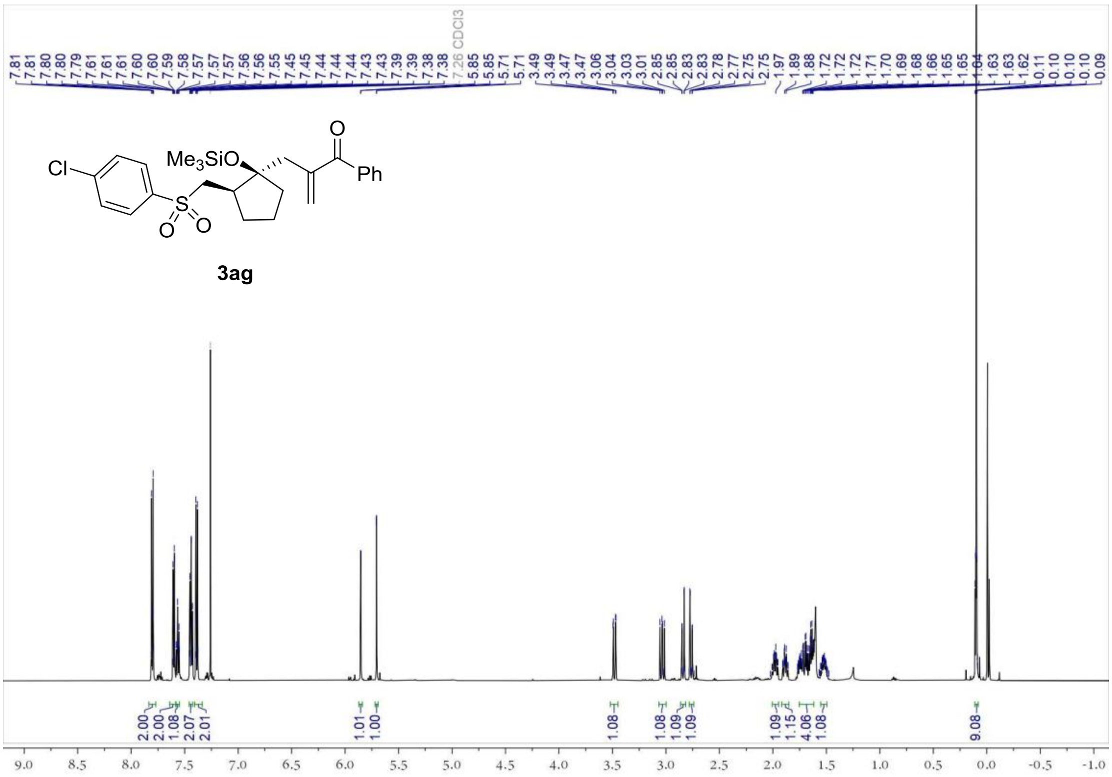

Supplementary Figure 198: ^{1}H NMR of **3ag** (600 MHz, $CDCl_3$, 25 ºC)

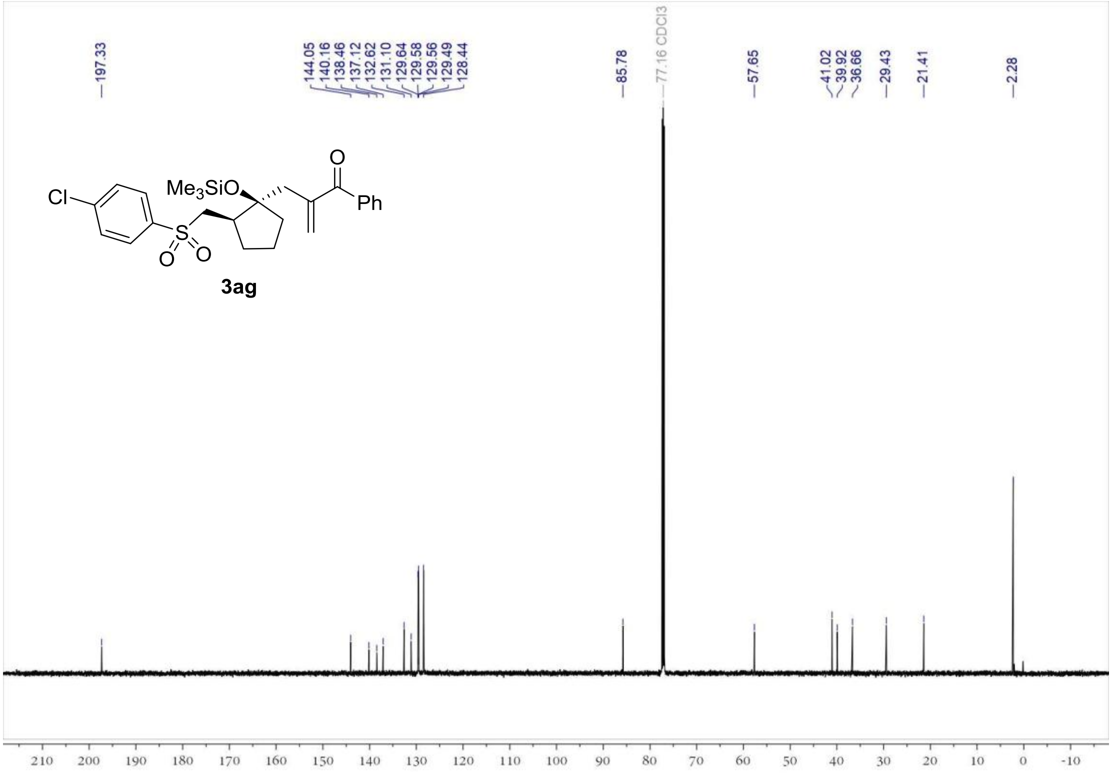

Supplementary Figure 199: ^{13}C NMR of **3ag** (151 MHz, $CDCl_3$, 25 ºC)

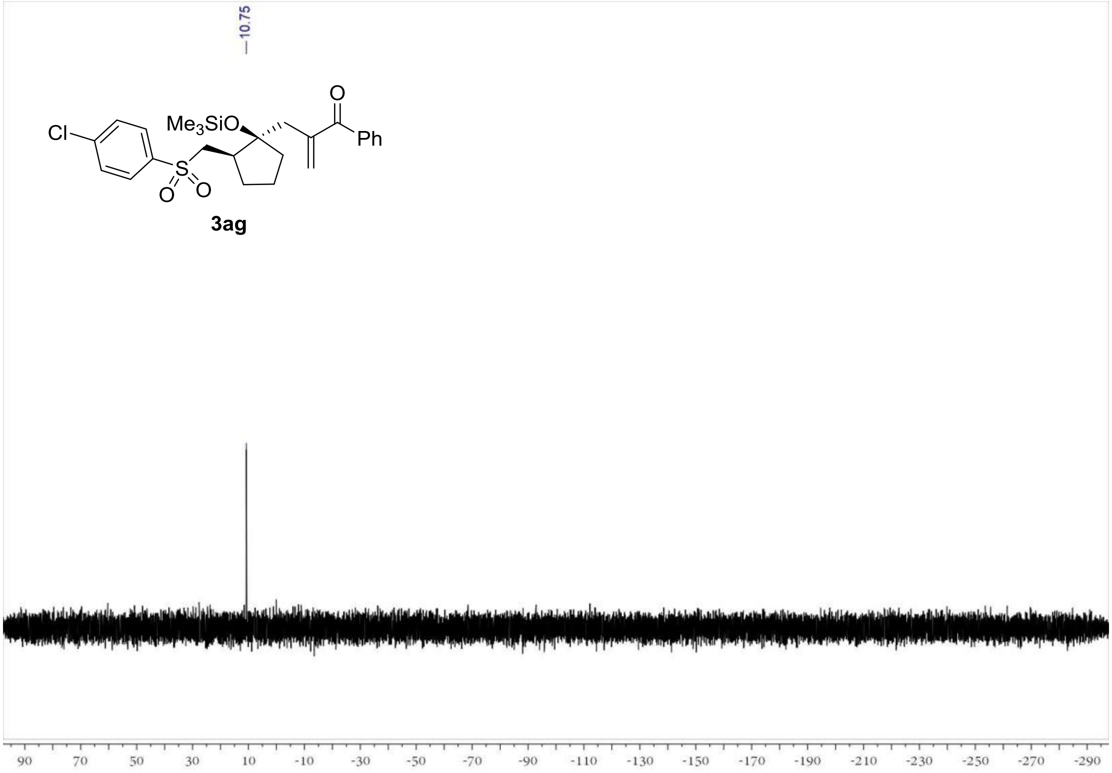

Supplementary Figure 200: ^{29}Si NMR of **3ag** (119 MHz, $CDCl_3$, 25 ºC)

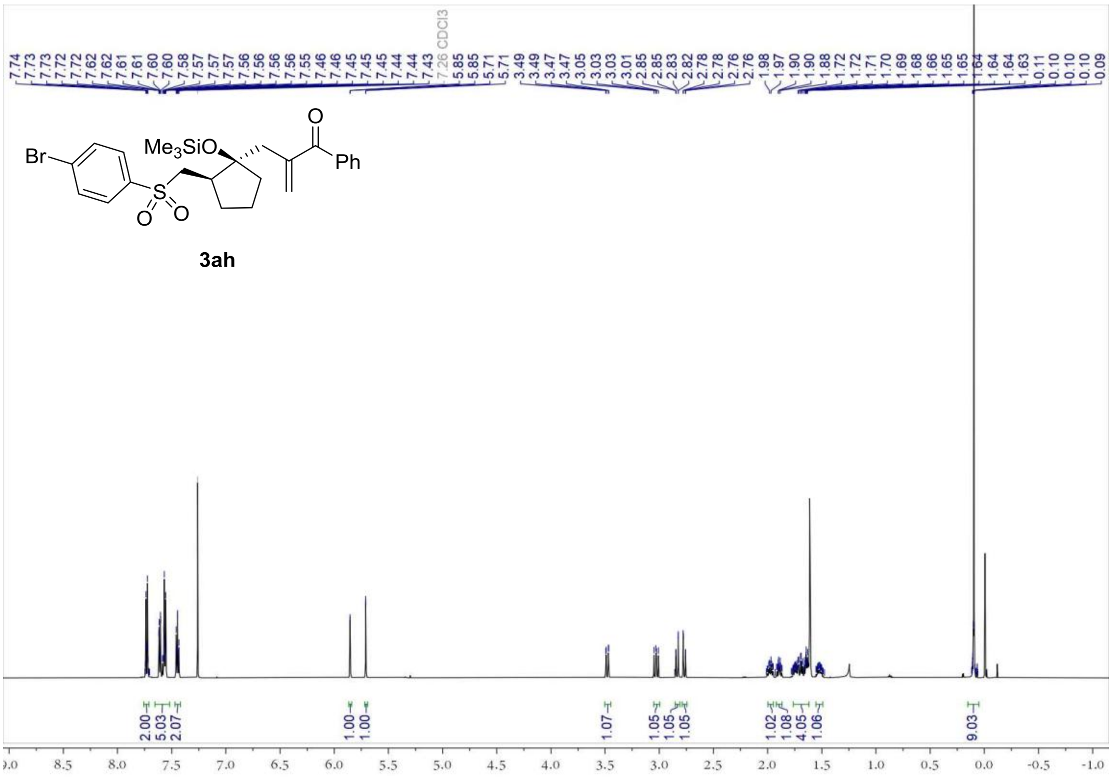

Supplementary Figure 201: ^{1}H NMR of **3ah** (600 MHz, $CDCl_3$, 25 ºC)

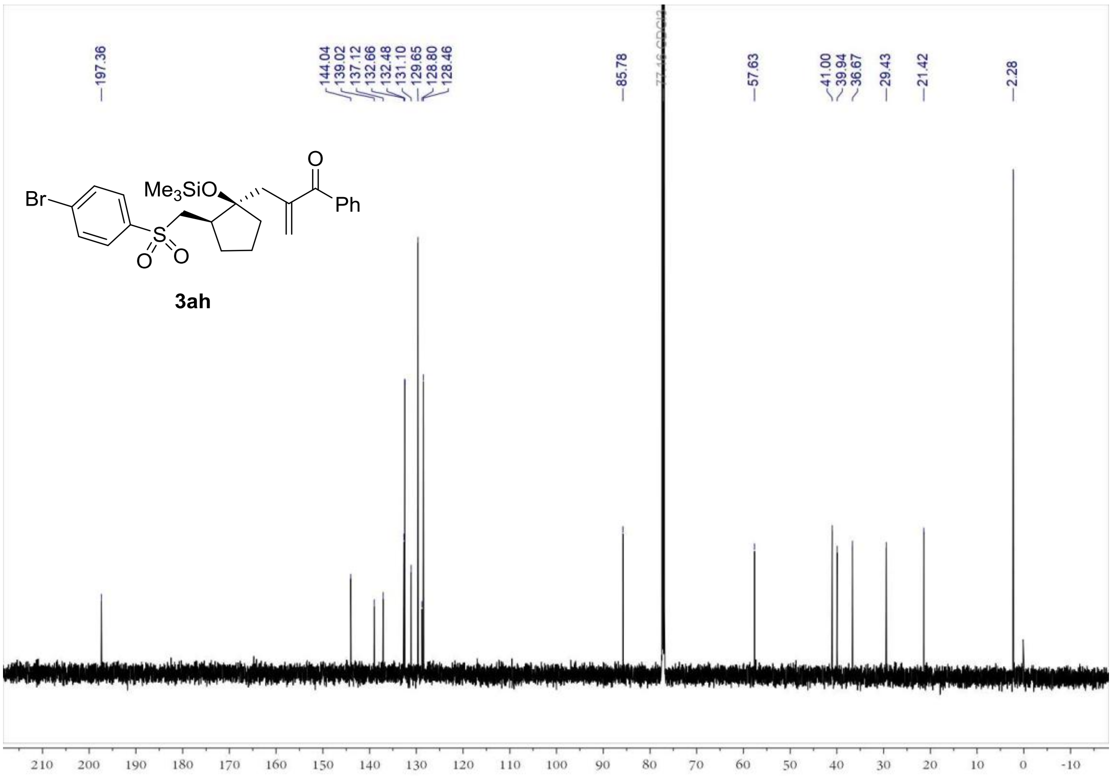

Supplementary Figure 202: ^{13}C NMR of **3ah** (151 MHz, $CDCl_3$, 25 ºC)

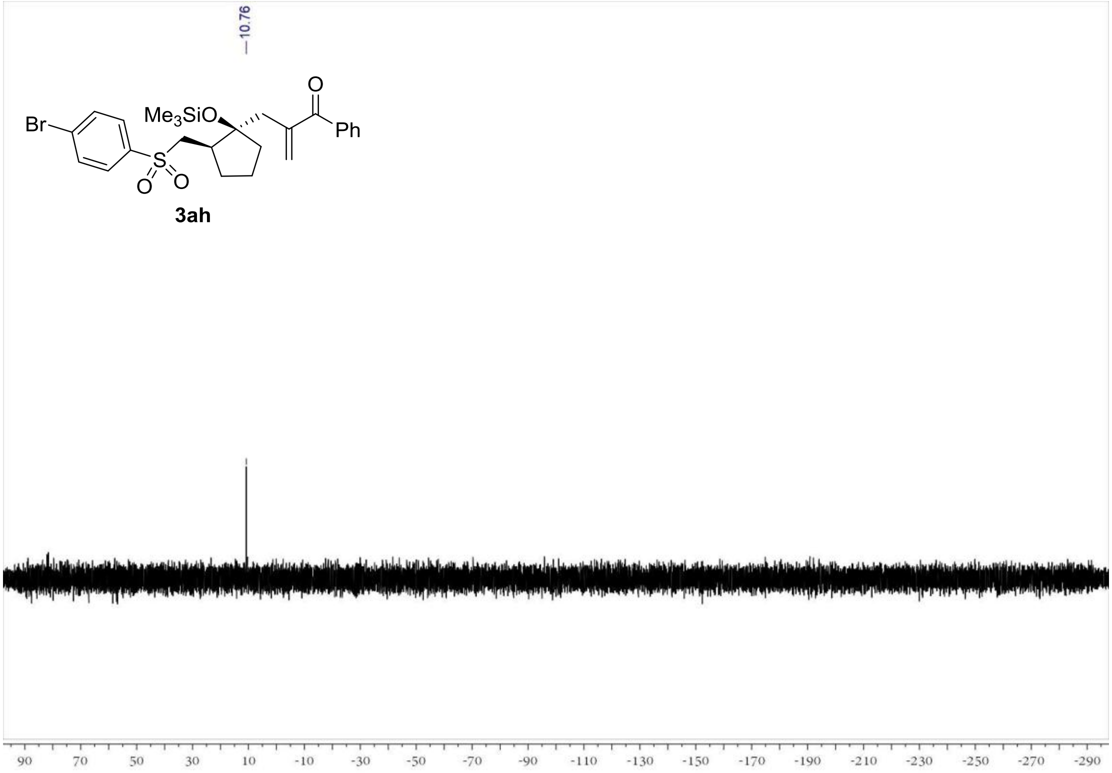

Supplementary Figure 203: ^{29}Si NMR of **3ah** (119 MHz, $CDCl_3$, 25 ºC)

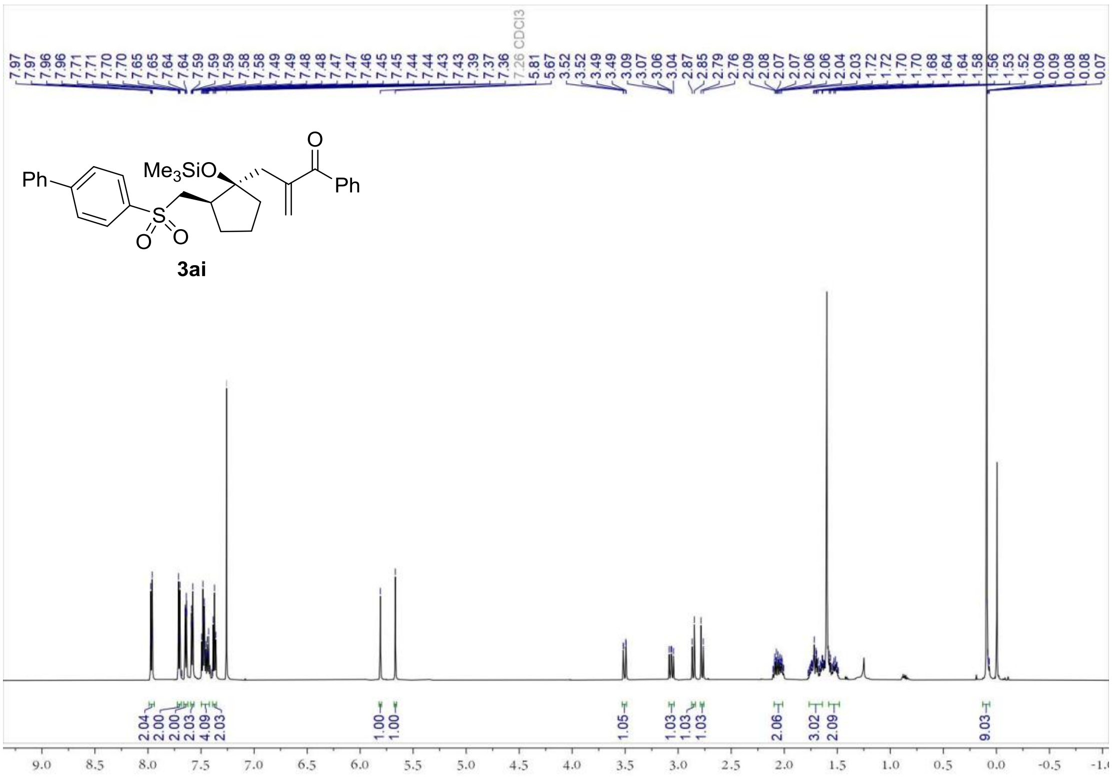

Supplementary Figure 204: ^{1}H NMR of **3ai** (600 MHz, $CDCl_3$, 25 ºC)

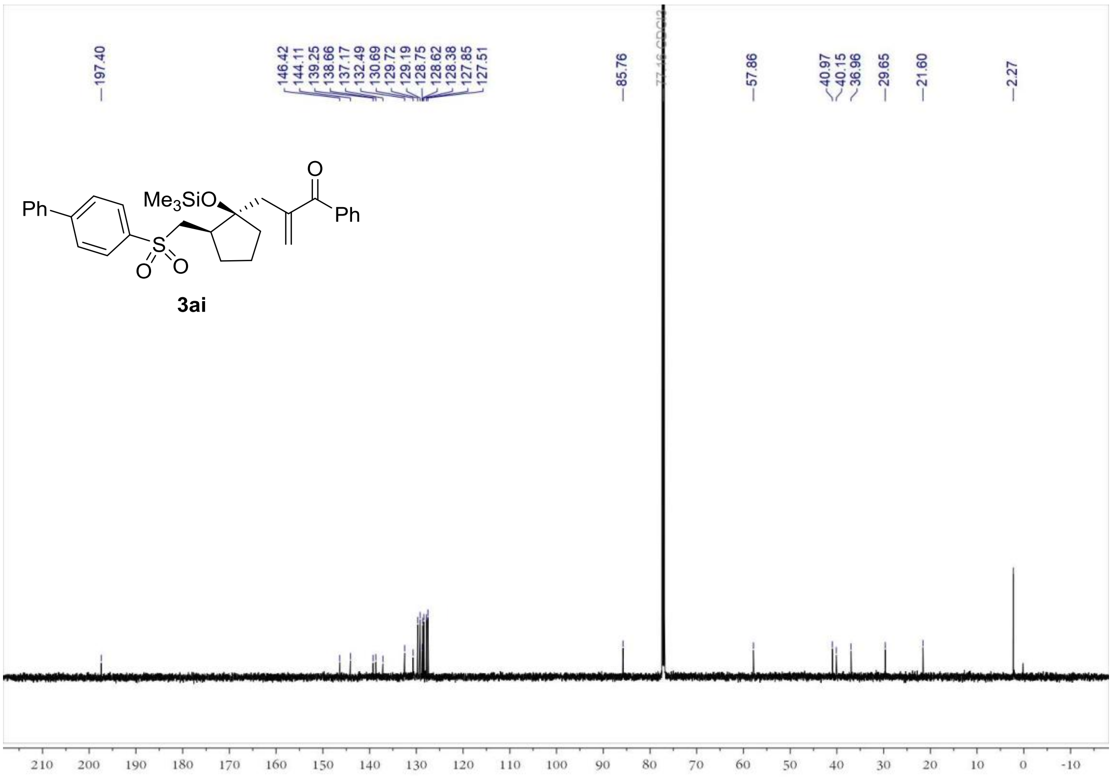

Supplementary Figure 205: ^{13}C NMR of **3ai** (151 MHz, $CDCl_3$, 25 ºC)

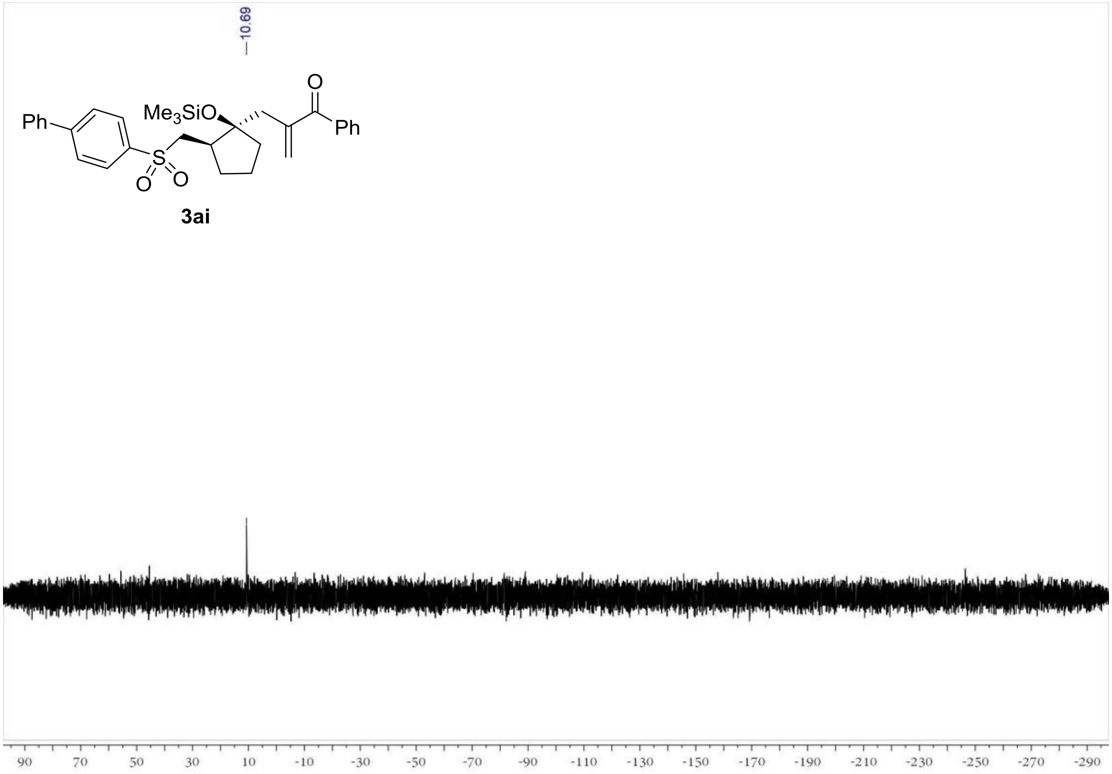

Supplementary Figure 206: ^{29}Si NMR of **3ai** (119 MHz, $CDCl_3$, 25 ºC)

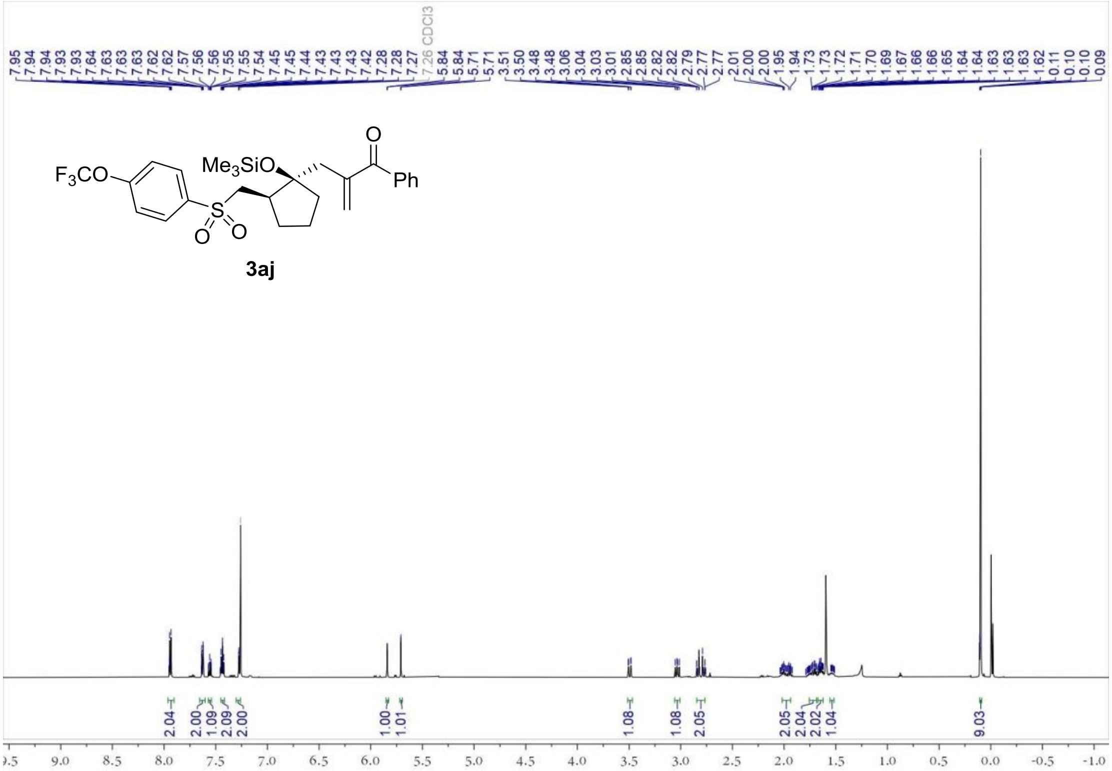

Supplementary Figure 207: ^{1}H NMR of **3aj** (600 MHz, $CDCl_3$, 25 ºC)

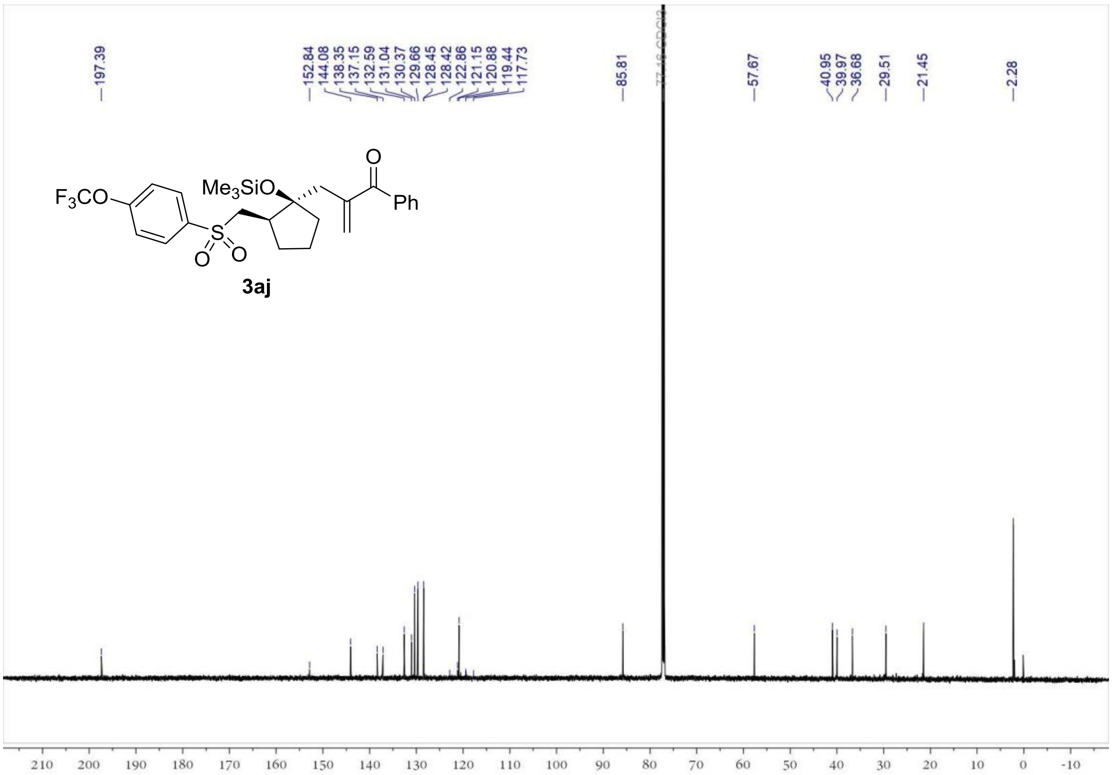

Supplementary Figure 208: ^{13}C NMR of **3aj** (151 MHz, $CDCl_3$, 25 ºC)

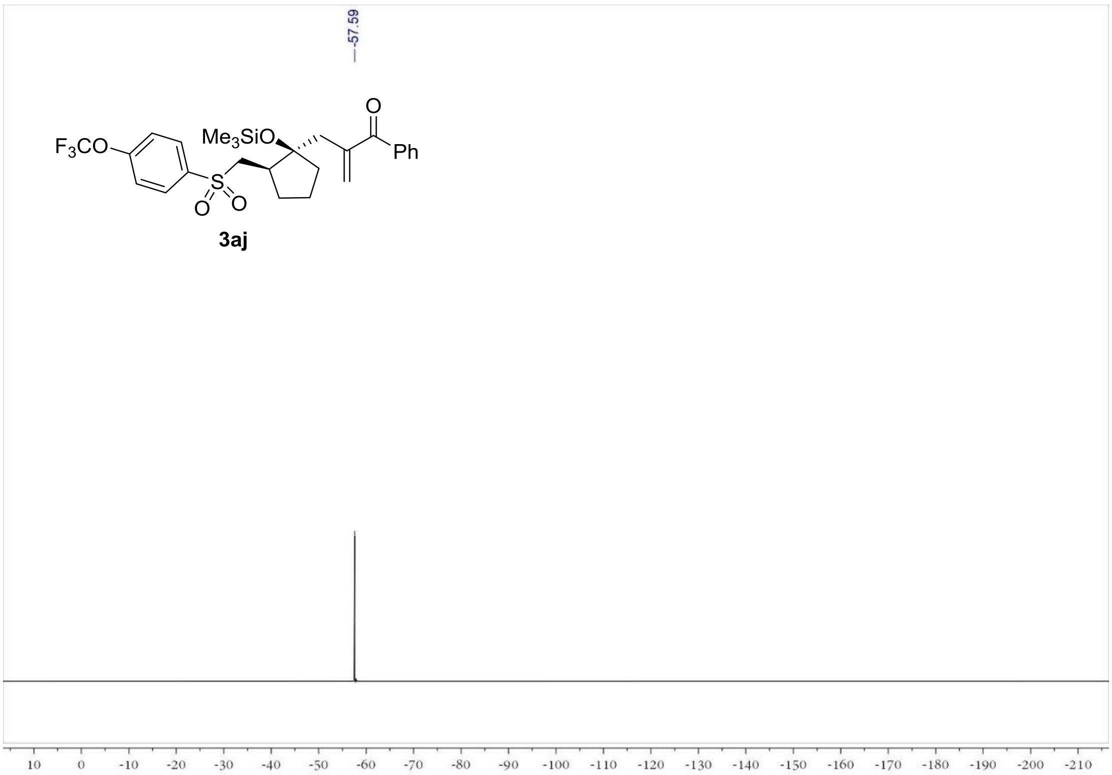

Supplementary Figure 209: ^{19}F NMR of **3aj** (565 MHz, $CDCl_3$, 25 ºC)

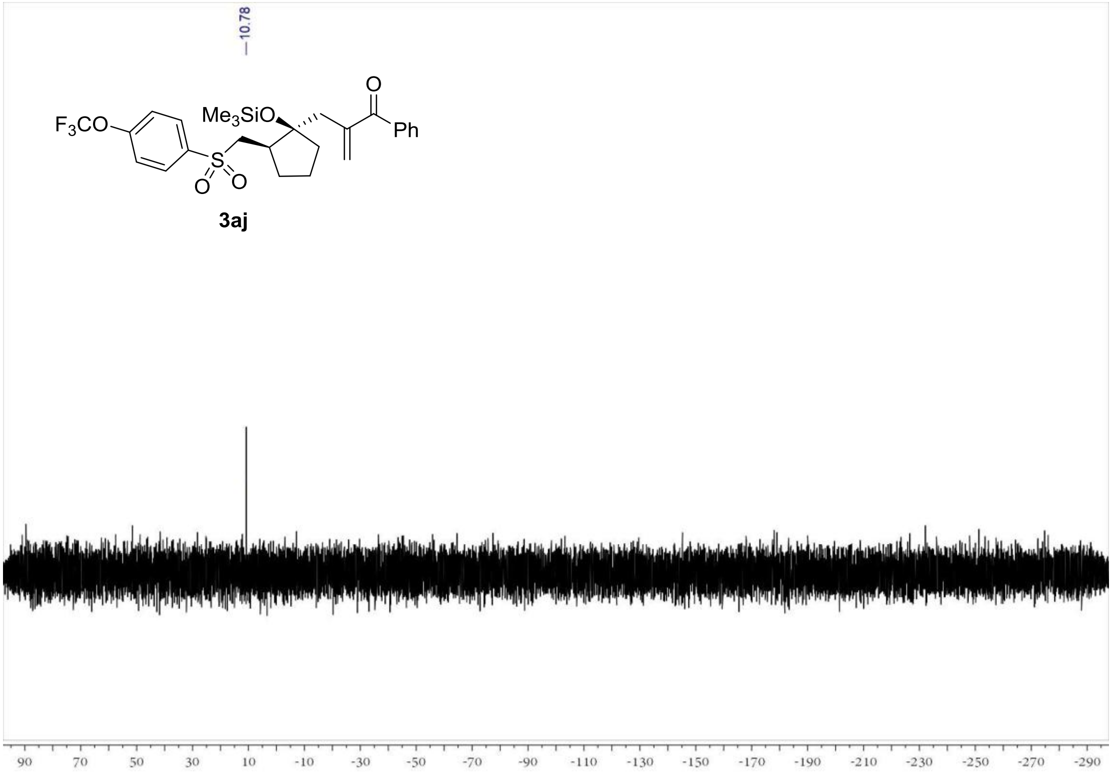

Supplementary Figure 210: ^{29}Si NMR of **3aj** (119 MHz, $CDCl_3$, 25 ºC)

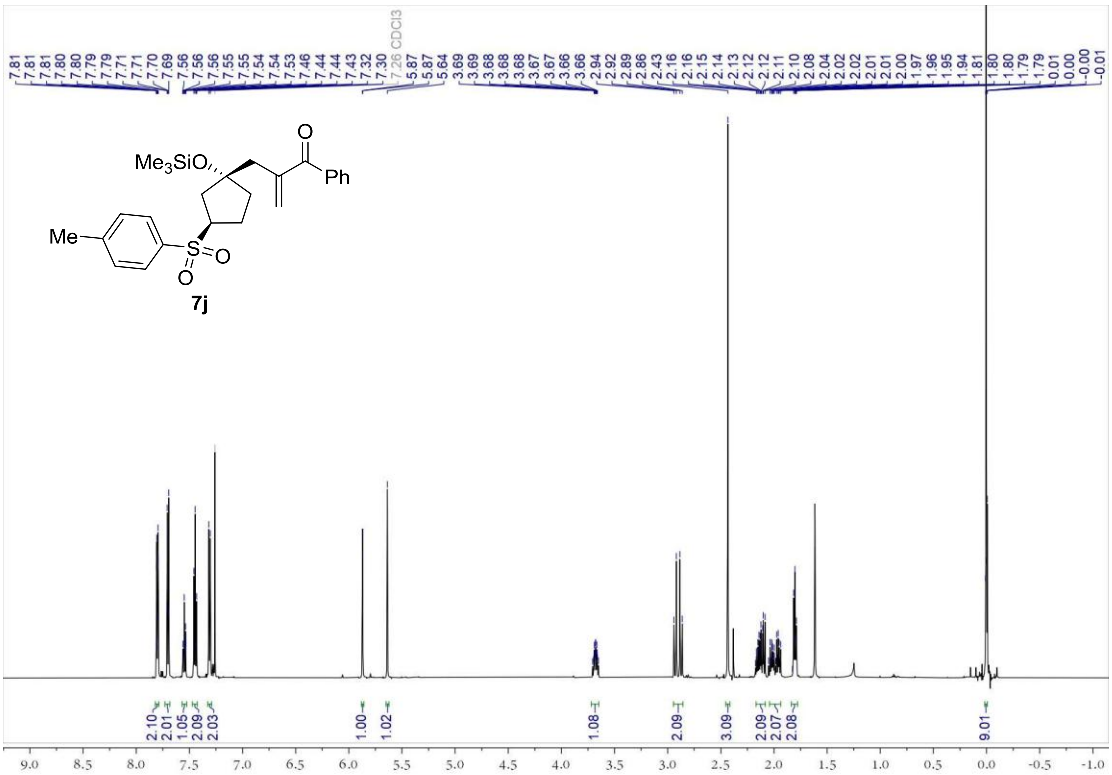

Supplementary Figure 211: ^{1}H NMR of **7j** (600 MHz, $CDCl_3$, 25 ºC)

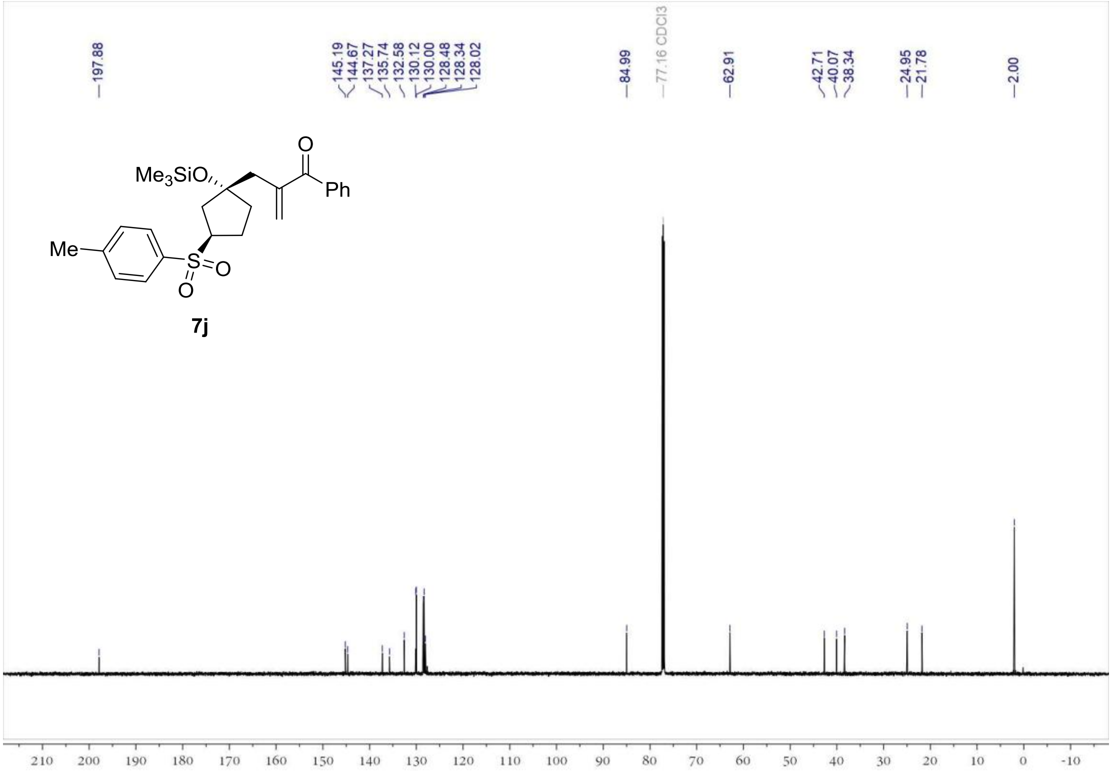

Supplementary Figure 212: ^{13}C NMR of **7j** (151 MHz, $CDCl_3$, 25 ºC)

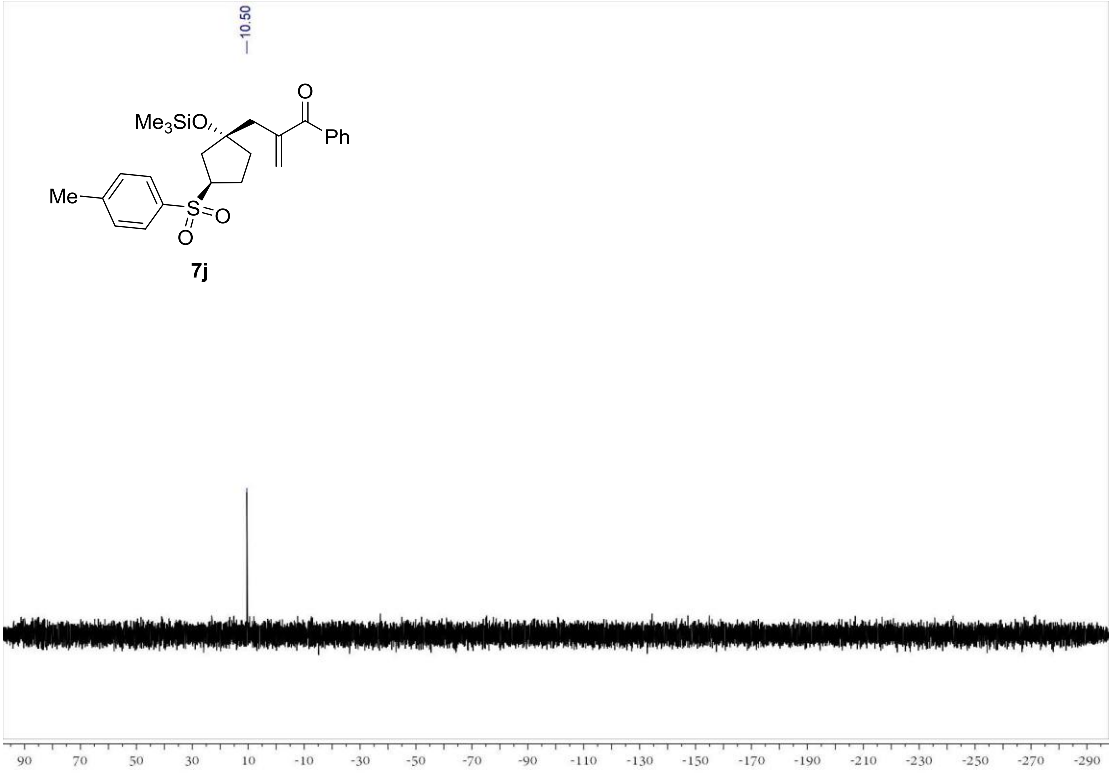

Supplementary Figure 213: ^{29}Si NMR of **7j** (119 MHz, $CDCl_3$, 25 ºC)

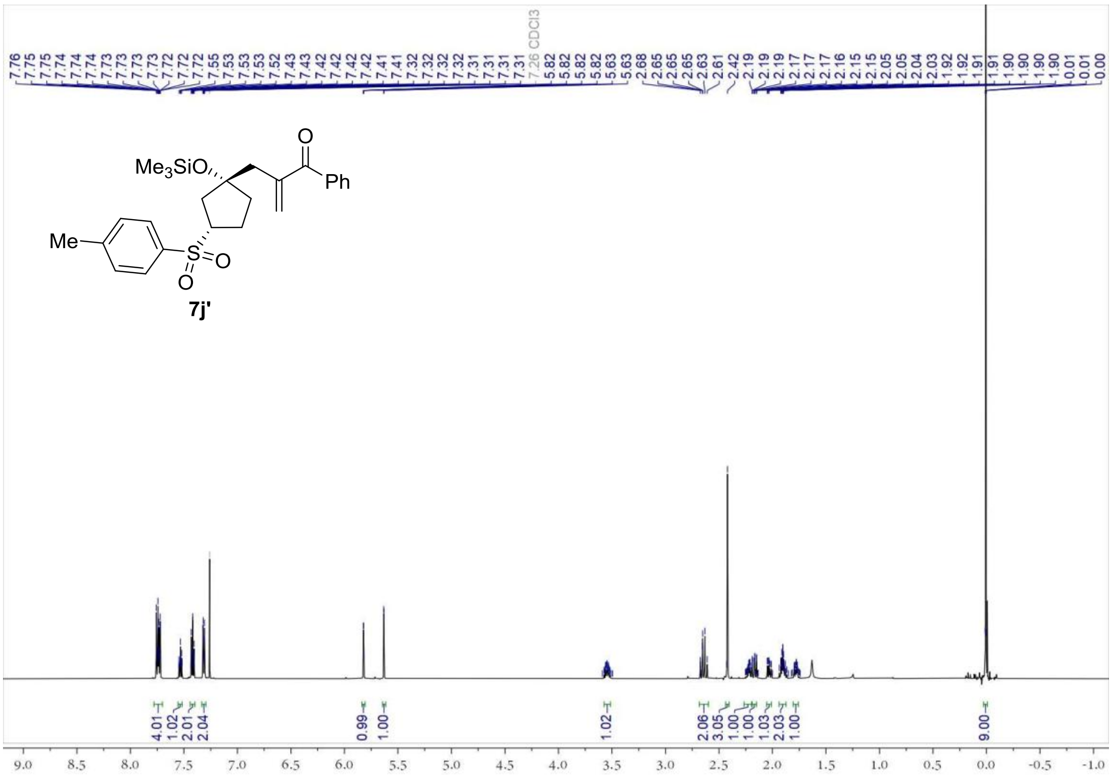

Supplementary Figure 214: ^{1}H NMR of **7j’** (600 MHz, $CDCl_3$, 25 ºC)

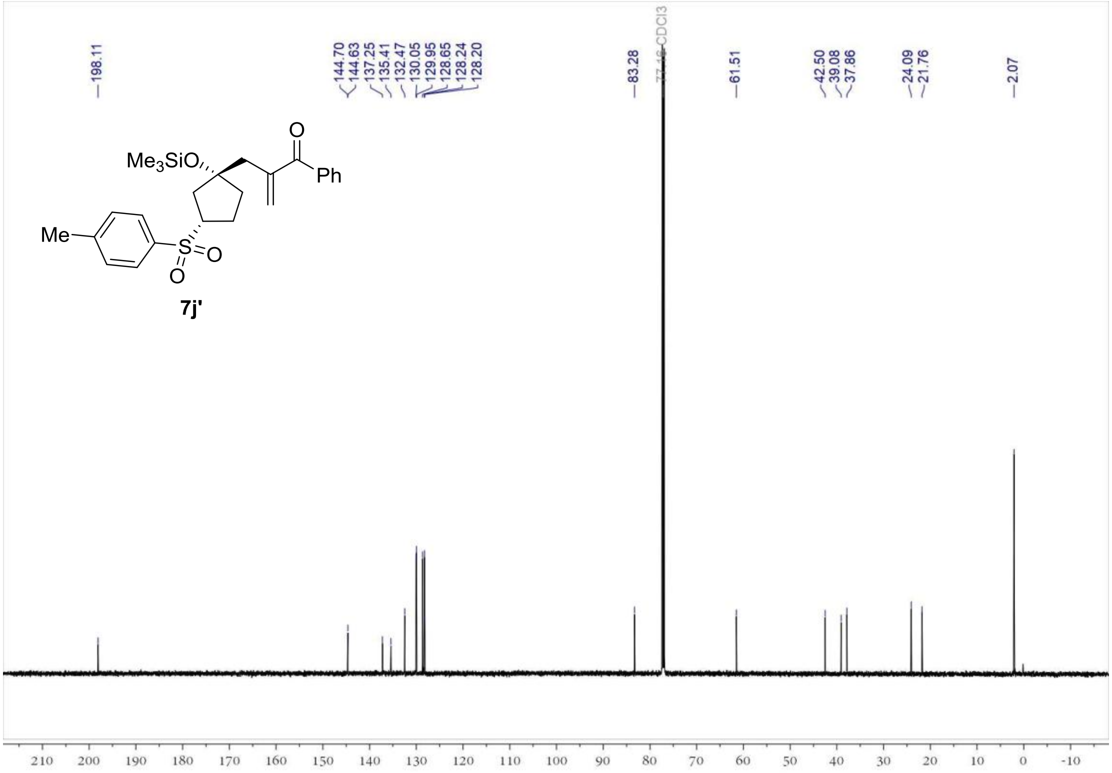

Supplementary Figure 215: ^{13}C NMR of **7j’** (151 MHz, $CDCl_3$, 25 ºC)

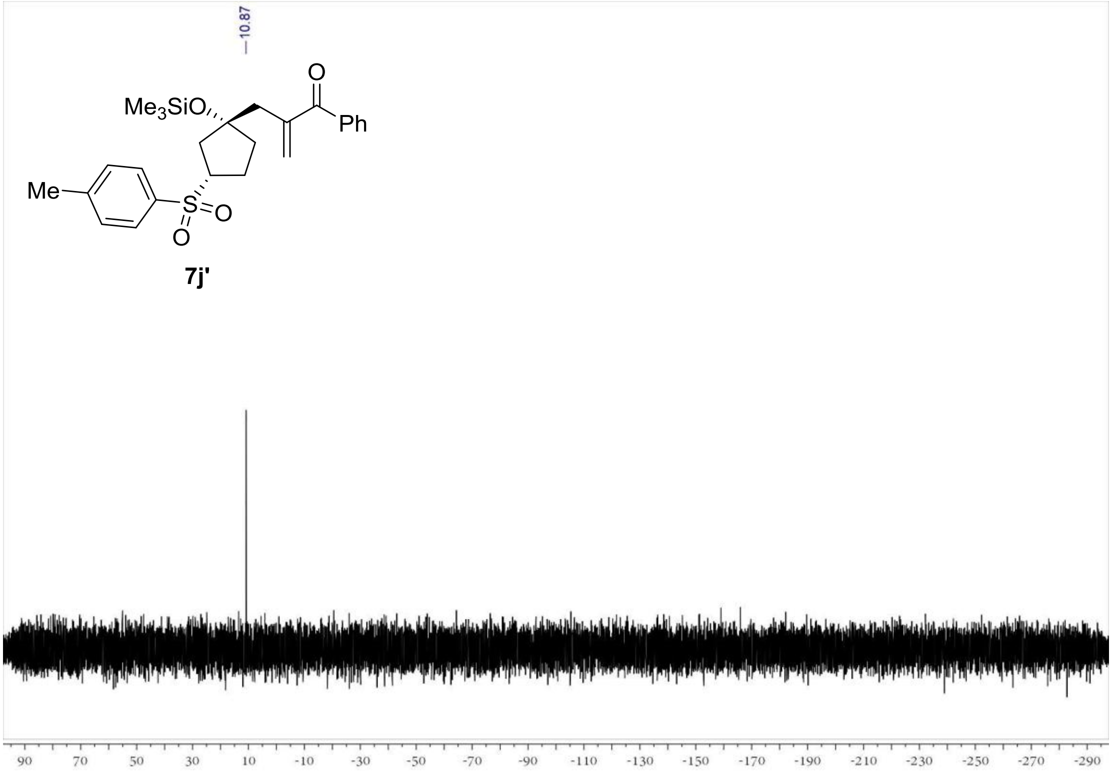

Supplementary Figure 216: ^{29}Si NMR of **7j’** (119 MHz, $CDCl_3$, 25 ºC)

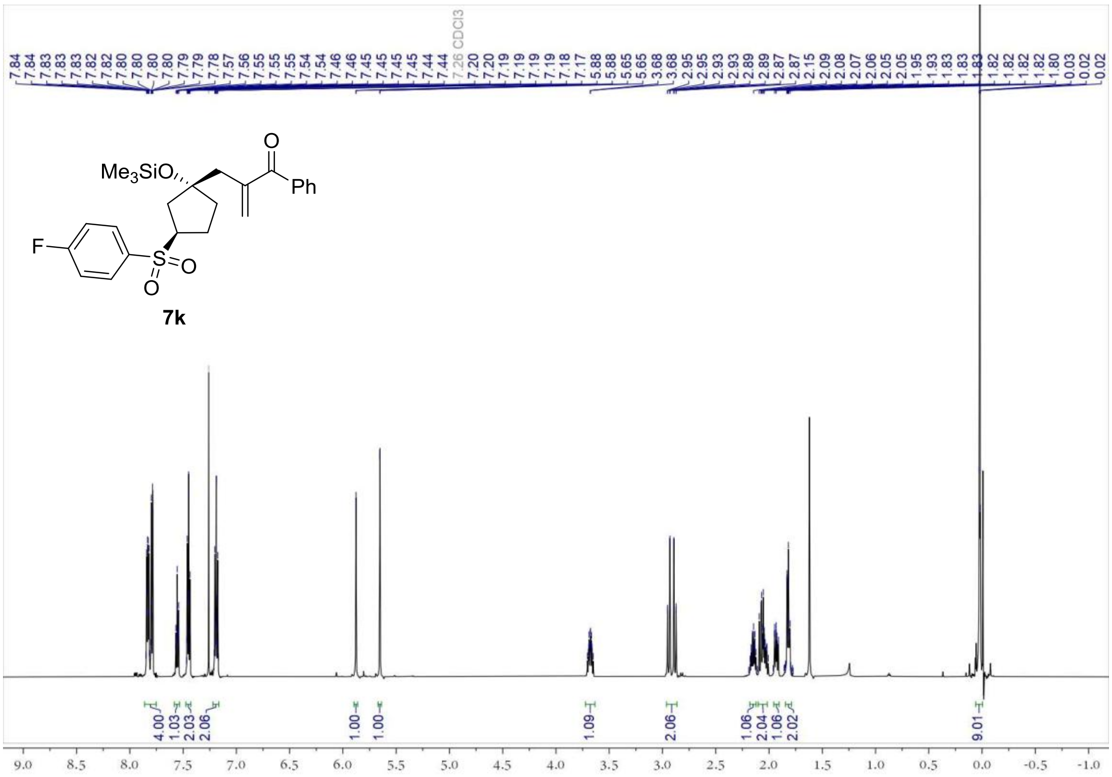

Supplementary Figure 217: ^{1}H NMR of **7k** (600 MHz, $CDCl_3$, 25 ºC)

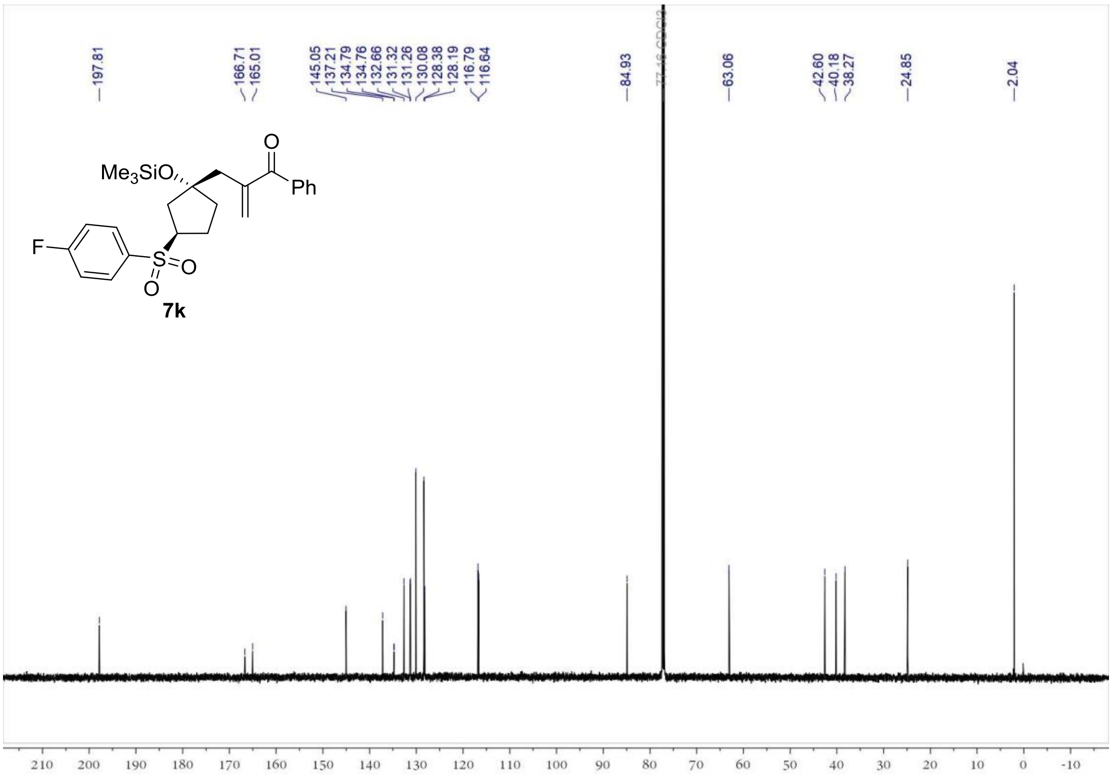

Supplementary Figure 218: ^{13}C NMR of **7k** (151 MHz, $CDCl_3$, 25 ºC)

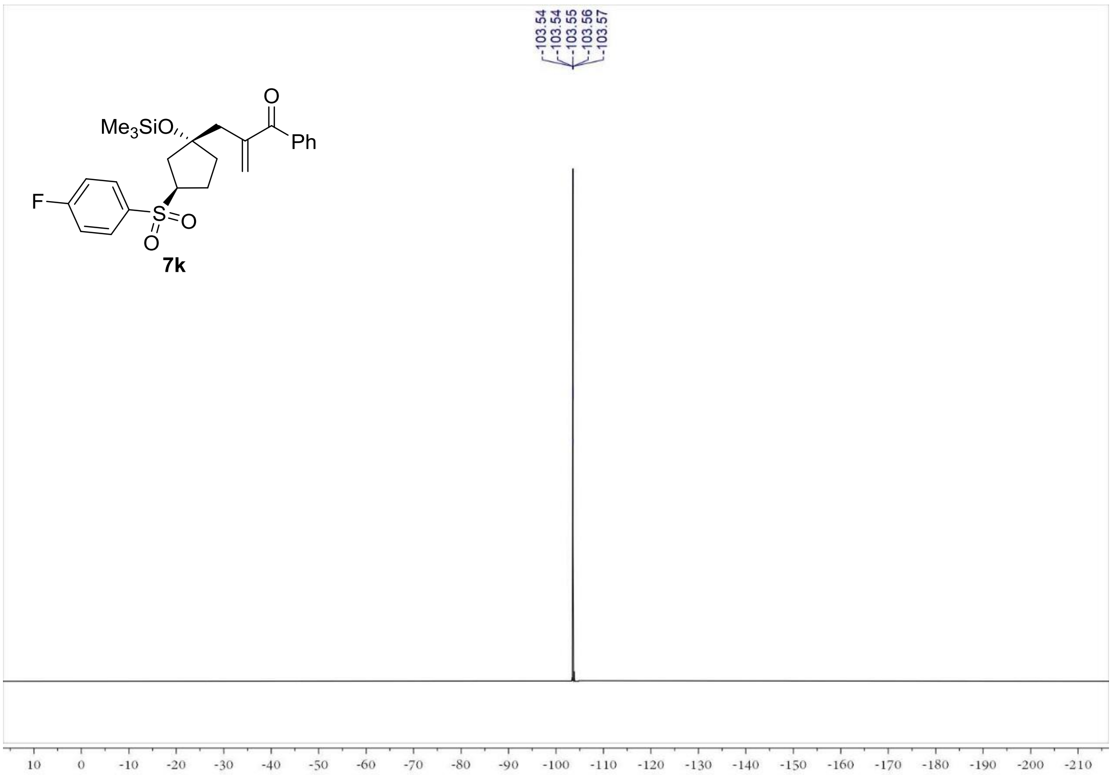

Supplementary Figure 219: ^{19}F NMR of **7k** (565 MHz, $CDCl_3$, 25 ºC)

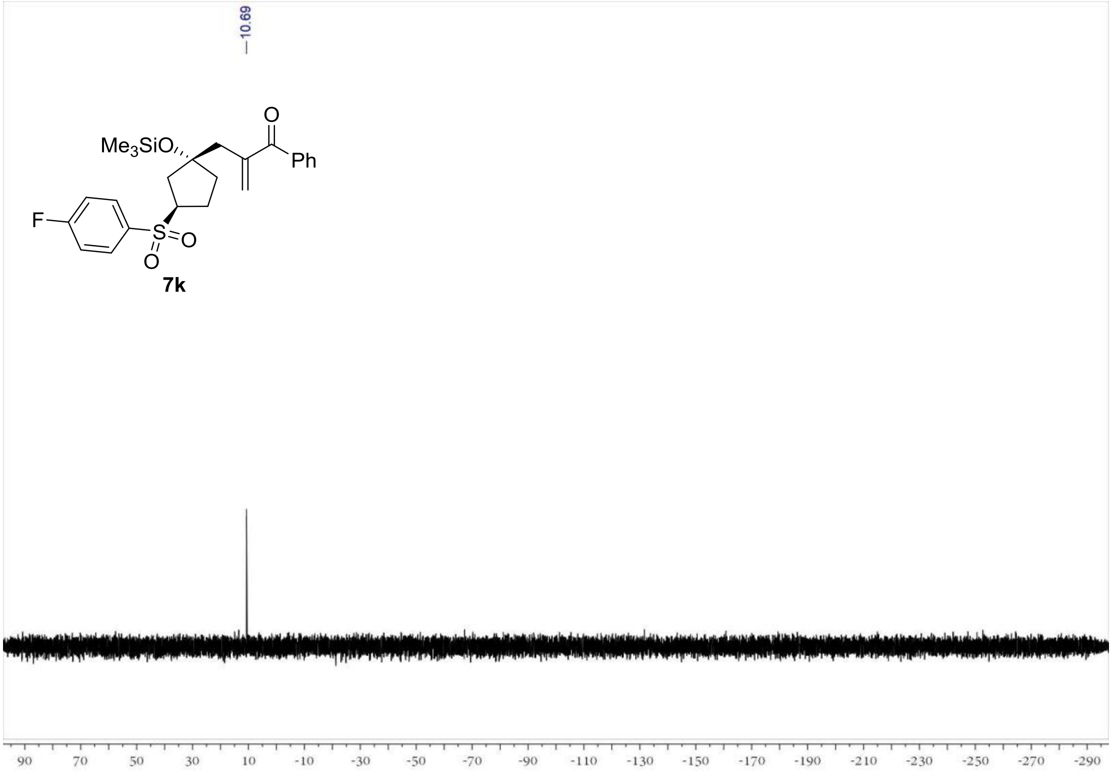

Supplementary Figure 220: ^{29}Si NMR of **7k** (119 MHz, $CDCl_3$, 25 ºC)

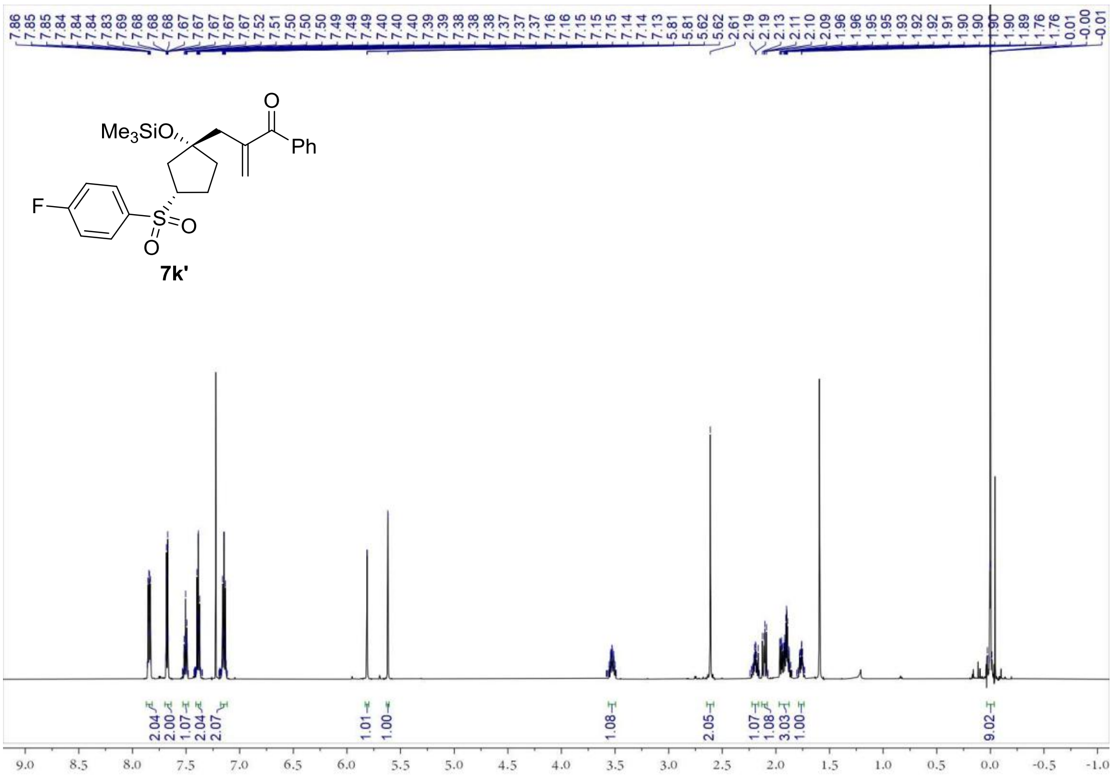

Supplementary Figure 221: ^{1}H NMR of **7k**' (600 MHz, CDCl$_3$, 25 ºC)

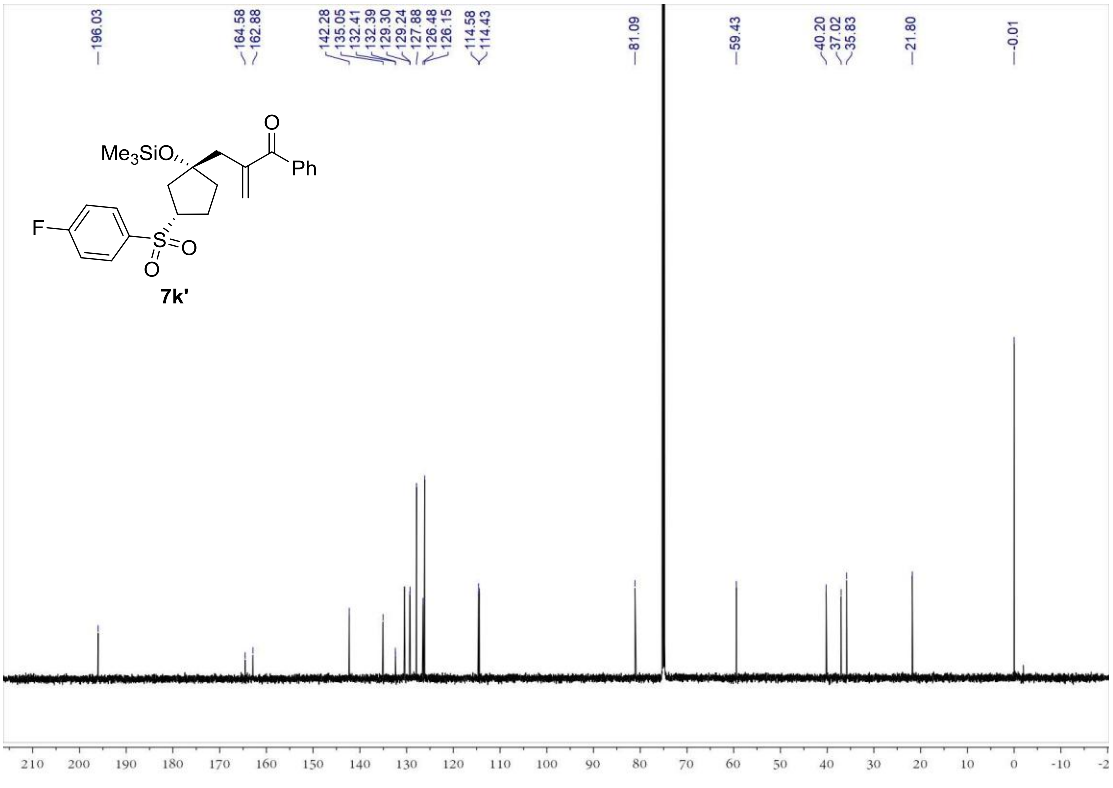

Supplementary Figure 222: ^{13}C NMR of **7k'** (151 MHz, CDCl$_3$, 25 ºC)

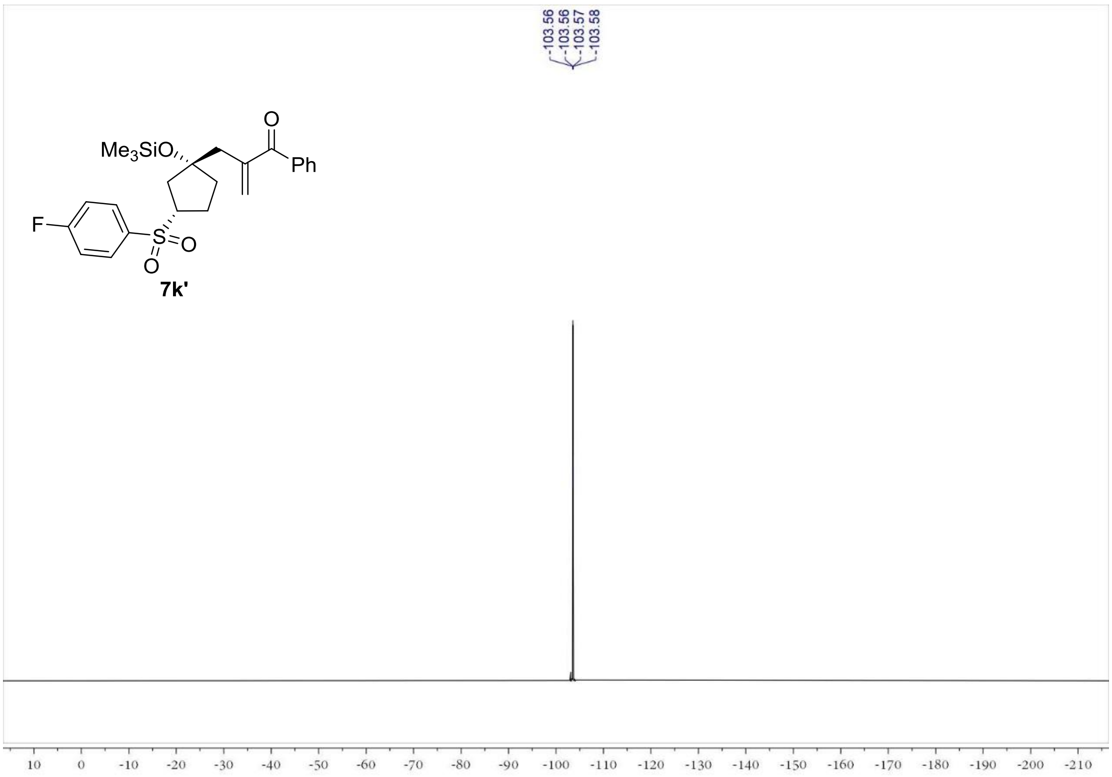

Supplementary Figure 223: ^{19}F NMR of **7k’** (565 MHz, $CDCl_3$, 25 ºC)

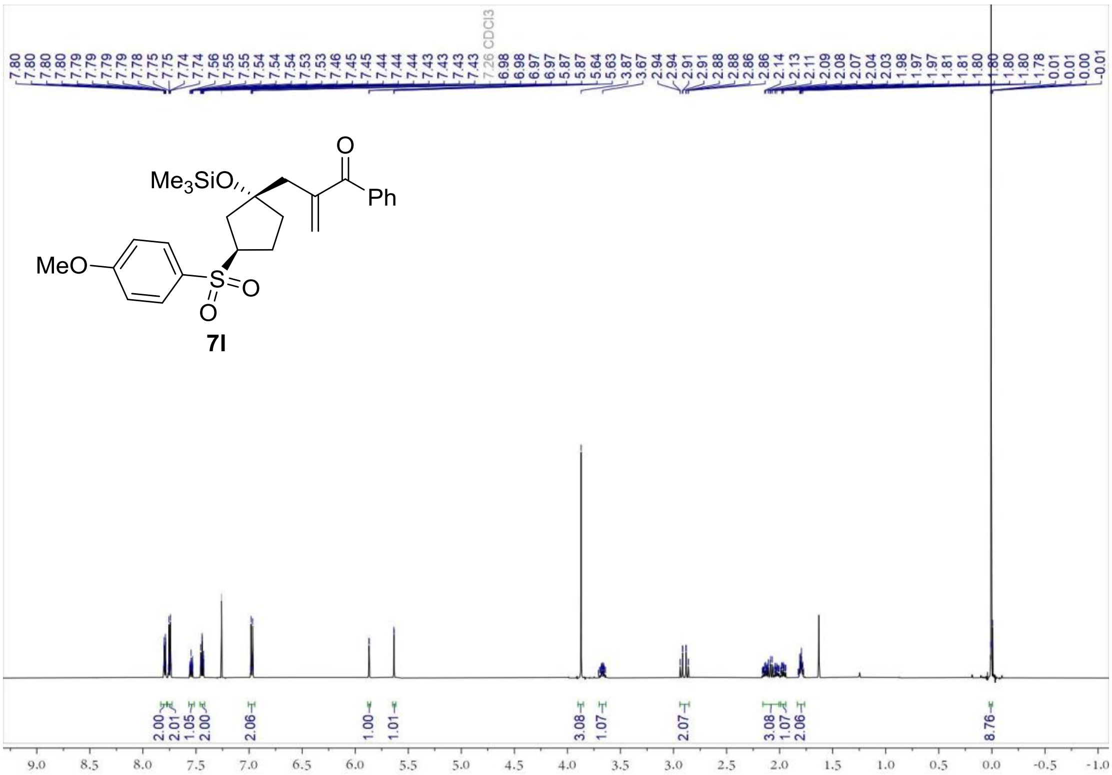

Supplementary Figure 224: ^{1}H NMR of **7l** (600 MHz, $CDCl_3$, 25 ºC)

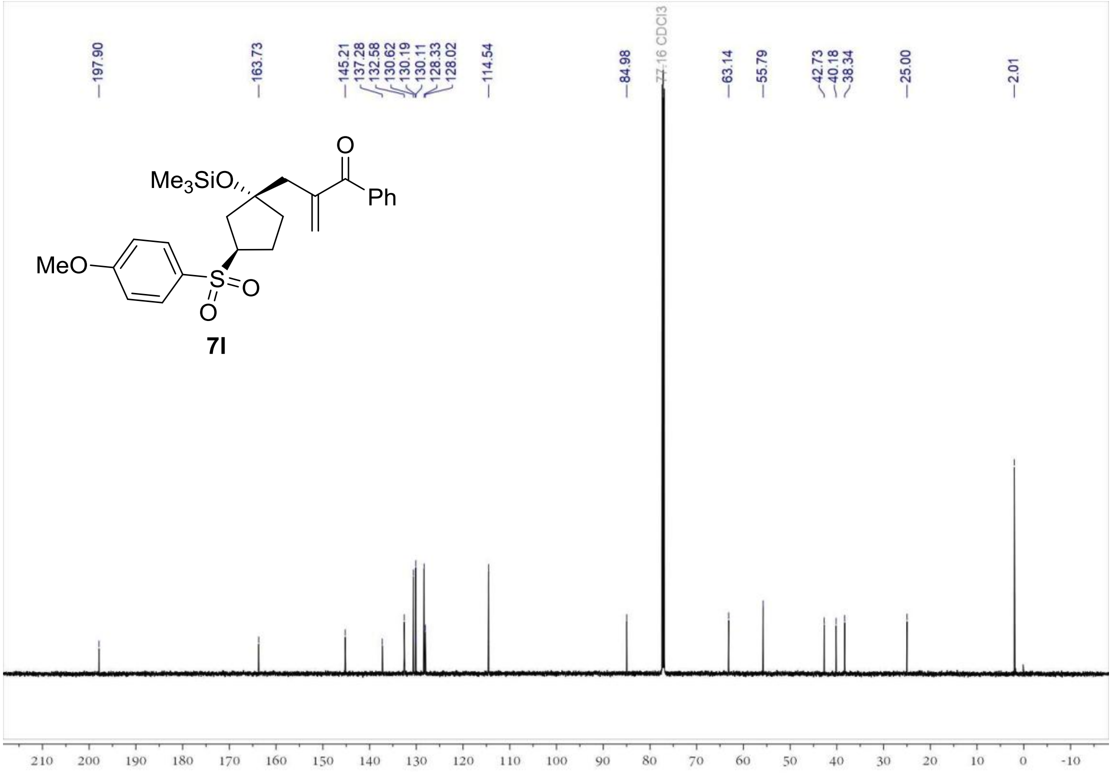

Supplementary Figure 225: ^{13}**C NMR of 7l (151 MHz, CDCl$_3$, 25 ºC)**

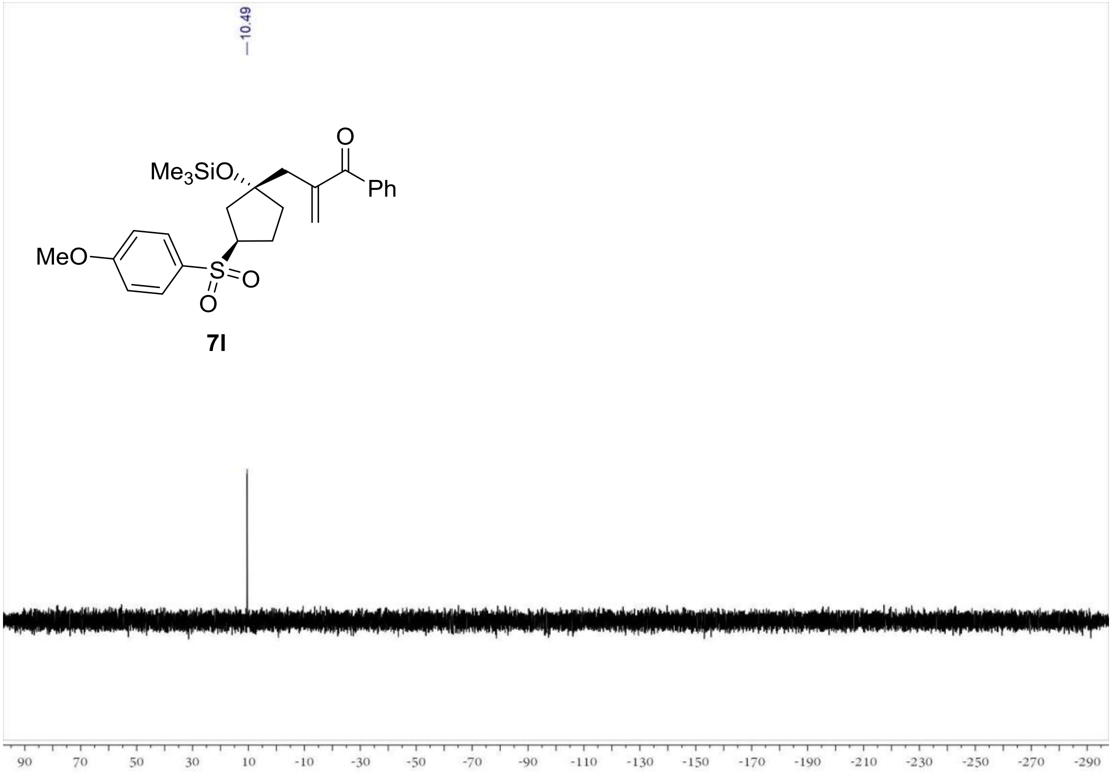

Supplementary Figure 226: ^{29}Si NMR of **7l** (119 MHz, CDCl$_3$, 25 ºC)

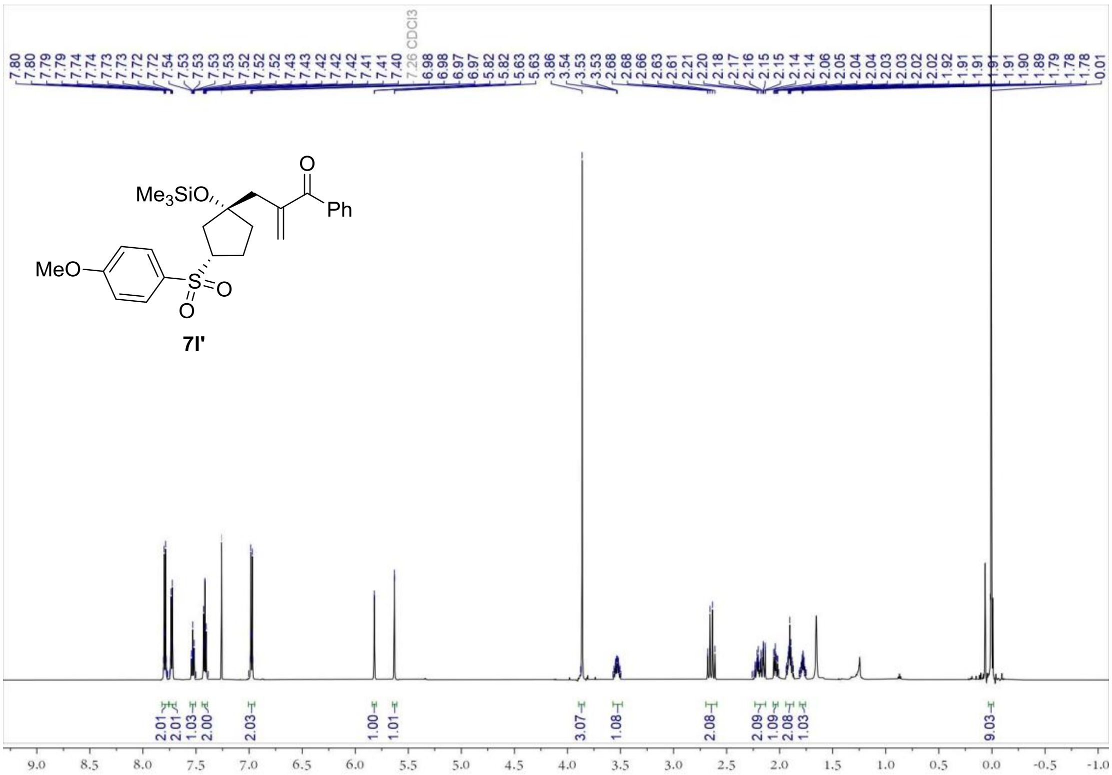

Supplementary Figure 227: ^{1}H NMR of **7l'** (600 MHz, $CDCl_3$, 25 ºC)

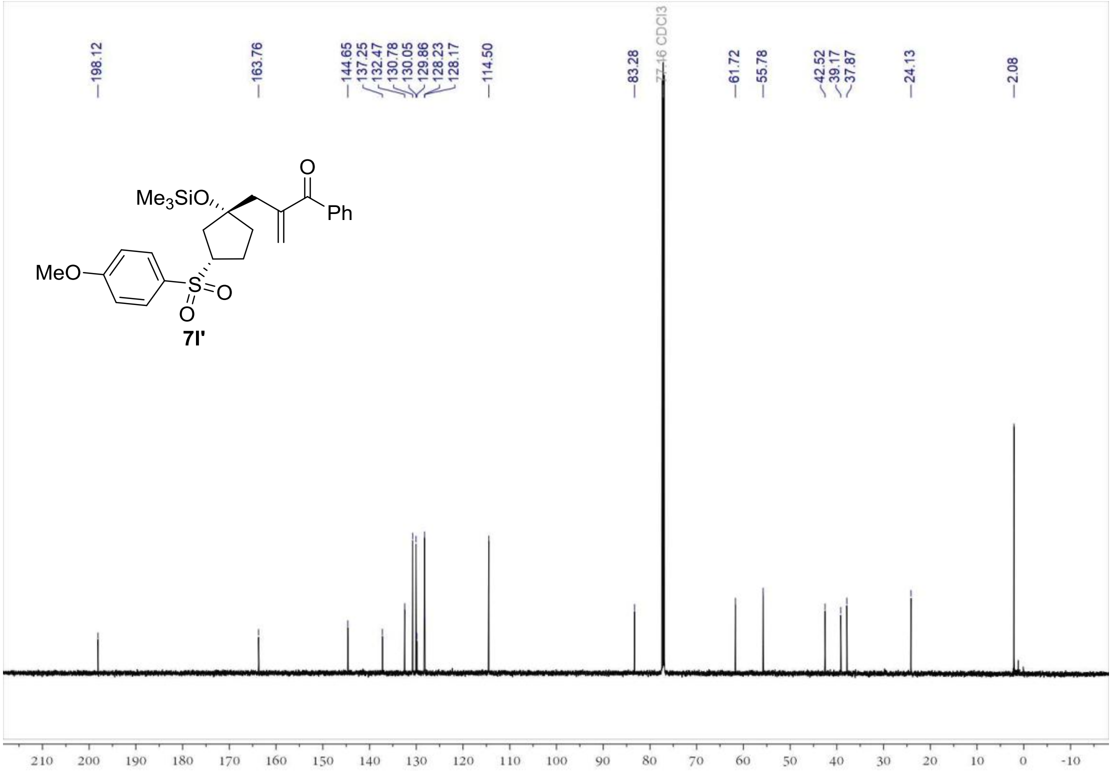

Supplementary Figure 228: ^{13}C NMR of **7l'** (151 MHz, $CDCl_3$, 25 ºC)

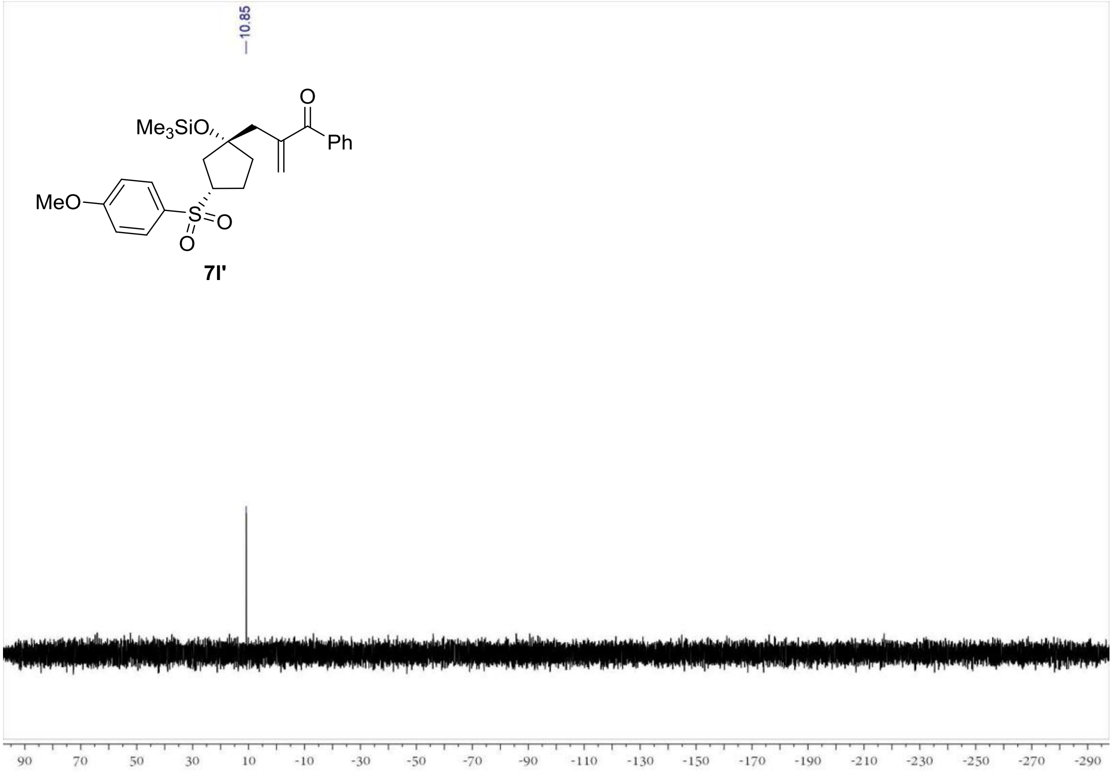

Supplementary Figure 229: ^{29}**Si NMR of 7l' (119 MHz, CDCl$_3$, 25 ºC)**

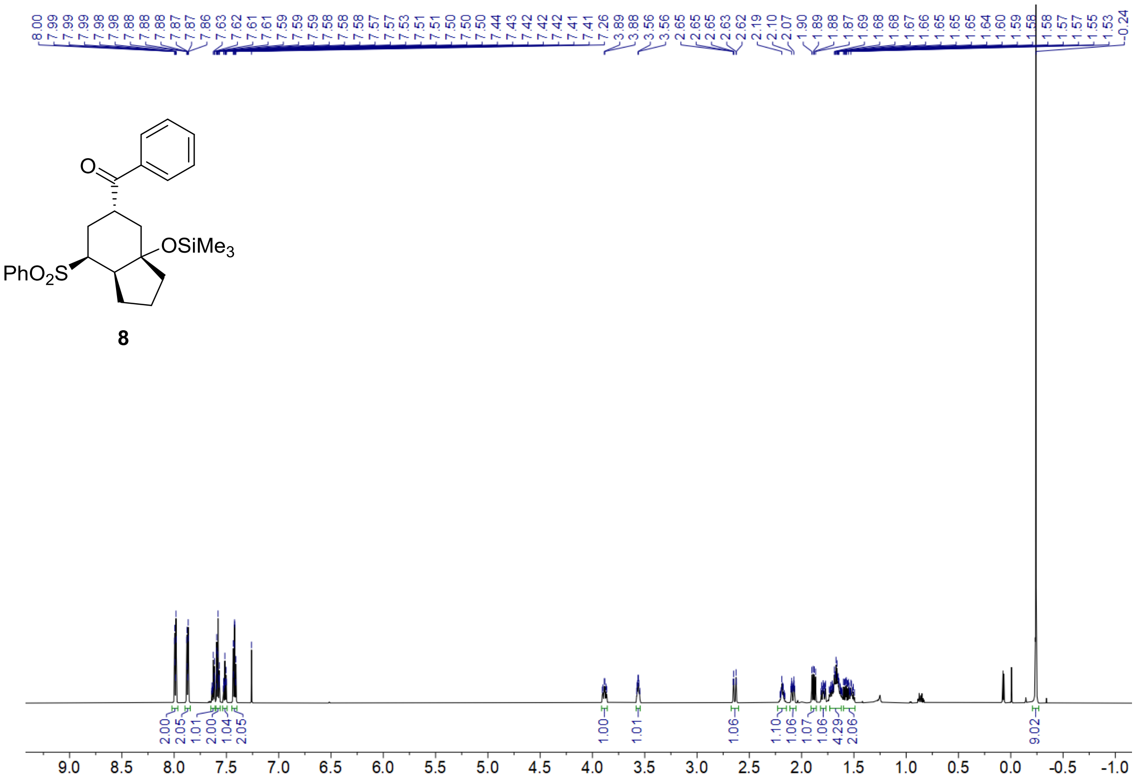

Supplementary Figure 230: ^{1}H NMR of **8** (600 MHz, CDCl$_3$, 25 ºC)

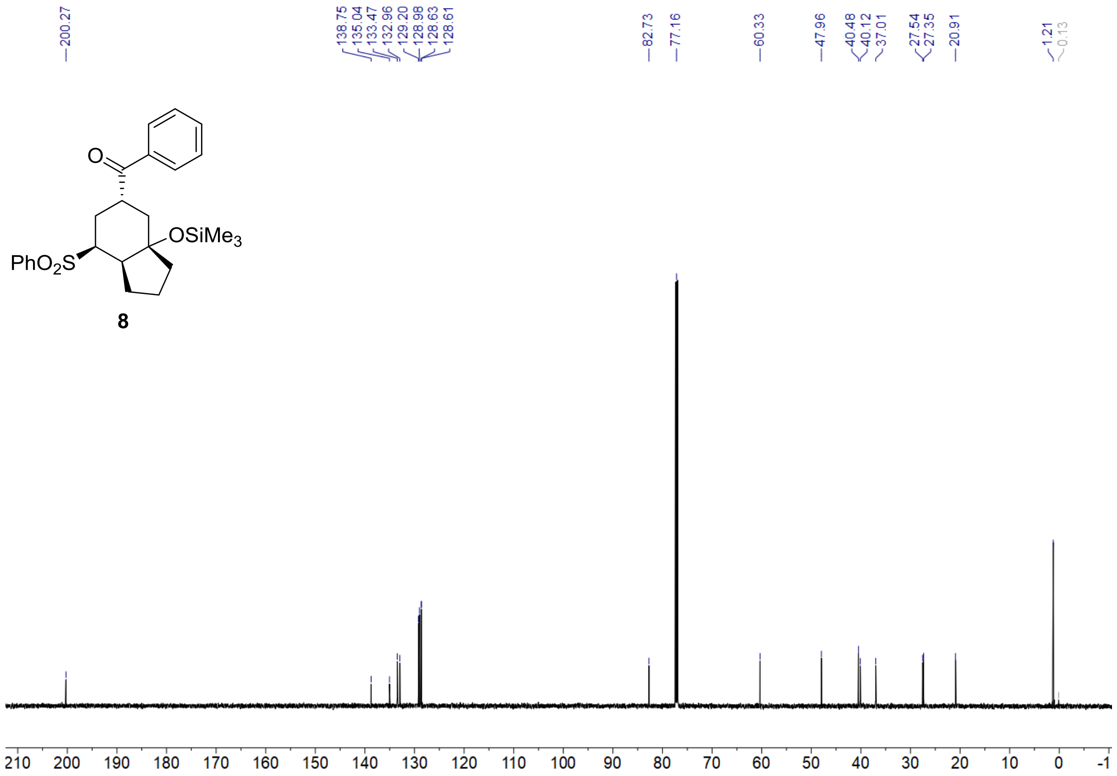

Supplementary Figure 231: ^{13}C NMR of **8** (151 MHz, $CDCl_3$, 25 ºC)

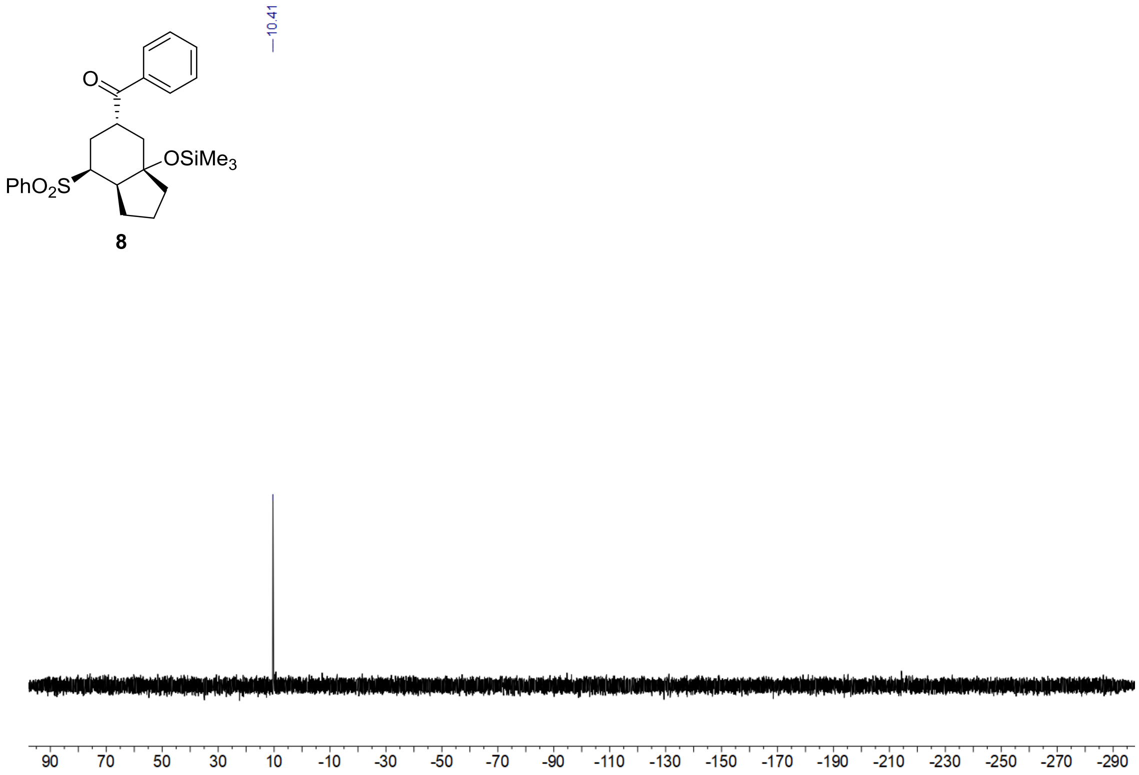

Supplementary Figure 232: ^{29}Si NMR of **8** (119 MHz, $CDCl_3$, 25 ºC)

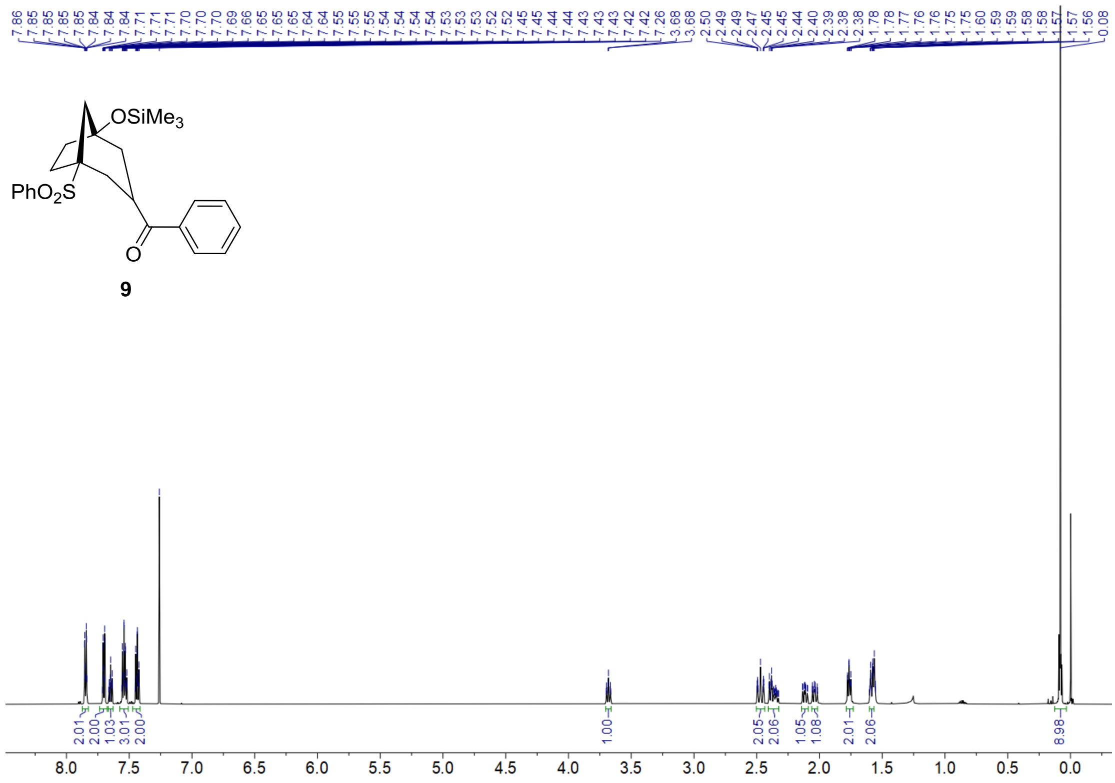

Supplementary Figure 233: ^{1}H NMR of **9** (600 MHz, $CDCl_3$, 25 ºC)

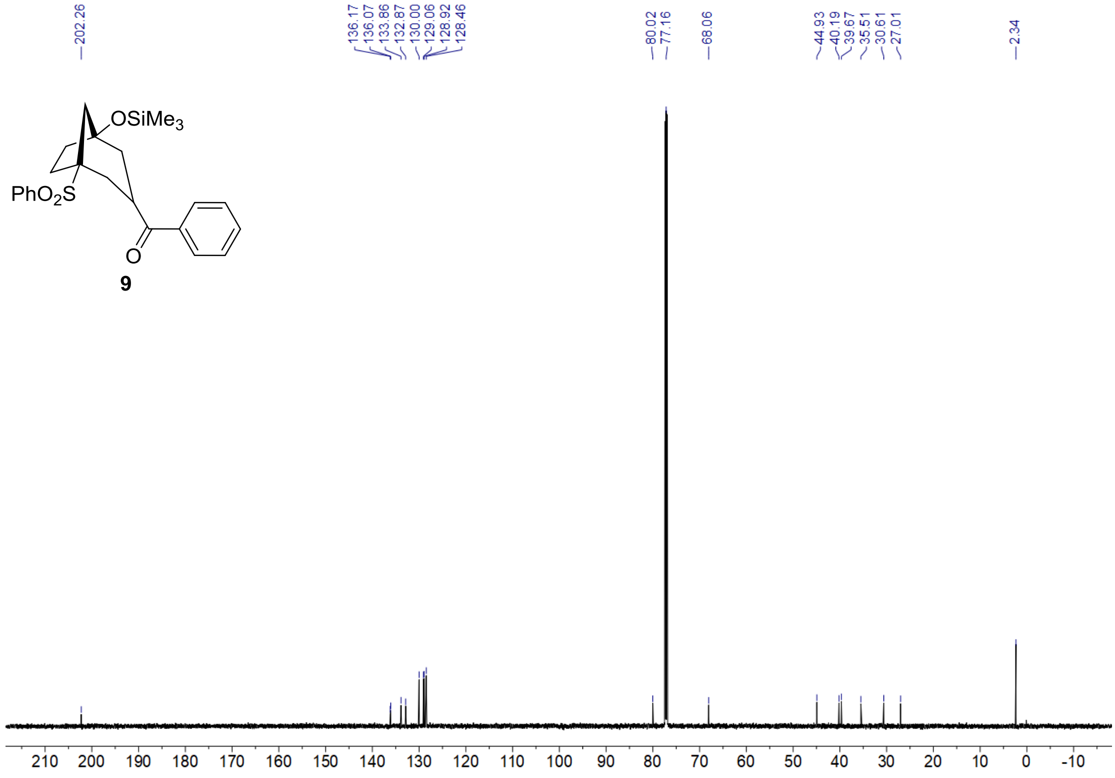

Supplementary Figure 234: ^{13}C NMR of **9** (151 MHz, $CDCl_3$, 25 ºC)

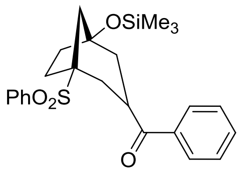

9

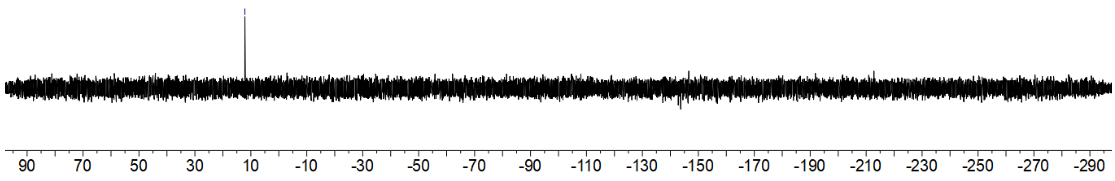

Supplementary Figure 235: ^{29}Si NMR of **9** (119 MHz, $CDCl_3$, 25 ºC)

8. Supplementary References

[1] Matsumoto, A., Asano, K. & Matsubara, S. Kinetic Resolution of Acylsilane Cyanohydrins via Organocatalytic Cycloetherification. *Chem. Asian J.* **14,** 116-120 (2019).

[2] Wilson, S. R., Haque, M. S. & Misra, R. N. Acyl silanes as sterically hindered aldehydes: additions, oxidations, and desilylations. *J. Org. Chem.* **47**, 747-748 (1982).

[3] Xiao, F., Liu, C., Wang, D., Huang, H. & Deng, G.-J. Concise synthesis of ketoallyl sulfones through an iron-catalyzed sequential four-component assembly. *Green Chem.* **20**, 973-977 (2018).

[4] Chen, X., Gong, X., Li, Z., Zhou, G., Zhu, Z., Zhang, W., Liu, S. & Shen, X. Direct transfer of tri- and di-fluoroethanol units enabled by radical activation of organosilicon reagents. *Nat. Commun.* **11**, 2756 (2020).

[5] Frisch, M. J., Trucks, G. W., Schlegel, H. B., Scuseria, G. E., Robb, M. A., Cheeseman, J. R., Scalmani, G., Barone, V., Mennucci, B., Petersson, G. A., Nakatsuji, H., Caricato, M., Li, X., Hratchian, H. P., Izmaylov, A. F., Bloino, J., Zheng, G., Sonnenberg, J. L., Hada, M., Ehara, M., Toyota, K., Fukuda, R., Hasegawa, J., Ishida, M., Nakajima, T., Honda, Y., Kitao, O., Nakai, H., Vreven, T., Montgomery, J. A., Jr., Peralta, J. E., Ogliaro, F., Bearpark, M., Heyd, J. J., Brothers, E., Kudin, K. N., Staroverov, V. N., Kobayashi, R., Normand, J., Raghavachari, K., Rendell, A., Burant, J. C., Iyengar, S. S., Tomasi, J., Cossi, M., Rega, N., Millam, N. J., Klene, M., Knox, J. E., Cross, J. B., Bakken, V., Adamo, C., Jaramillo, J., Gomperts, R., Stratmann, R. E., Yazyev, O., Austin, A. J., Cammi, R., Pomelli, C., Ochterski, J. W., Martin, R. L., Morokuma, K., Zakrzewski, V. G., Voth, G. A., Salvador, P., Dannenberg, J. J., Dapprich, S., Daniels, A. D., Farkas, O., Foresman, J. B., Ortiz, J. V., Cioslowski, J. & Fox, D. J., Gaussian 16, Revision C.01; Gaussian, Inc.: Wallingford, CT, 2016.

[6] Zhao Y. & Truhlar D G., The M06 suite of density functionals for main group thermochemistry, thermochemical kinetics, noncovalent interactions, excited states, and transition elements: two new functionals and systematic testing of four M06-class functionals and 12 other functionals. *Theor. Chem. Acc.* **120**, 215–241 (2008).

[7] Marenich A.V., Cramer C.G. & Truhlar D.G., Universal Solvation Model Based on Solute Electron Density and on a Continuum Model of the Solvent Defined by the Bulk Dielectric Constant and Atomic Surface Tensions. *J. Phy. Chem. B*, **113**, 6378–6396. (2009).

[8] CYLview, 1.0b; Legault, C. Y., Université de Sherbrooke, (2009).
